# Synthesis, In Silico Study, and In Vitro Antifungal Activity of New 5-(1,3-Diphenyl-1*H*-Pyrazol-4-yl)-4-Tosyl-4,5-Dihydrooxazoles

**DOI:** 10.3390/ijms25105091

**Published:** 2024-05-07

**Authors:** Neively Tlapale-Lara, Julio López, Elizabeth Gómez, Lourdes Villa-Tanaca, Edson Barrera, Carlos H. Escalante, Joaquín Tamariz, Francisco Delgado, Dulce Andrade-Pavón, Omar Gómez-García

**Affiliations:** 1 Departamento de Química Orgánica, Escuela Nacional de Ciencias Biológicas, Instituto Politécnico Nacional, Prol. Carpio y Plan de Ayala S/N, Mexico City 11340, Mexico; neively.tlapale@gmail.com (N.T.-L.); jclmtz@hotmail.com (J.L.); ebarrerac0800@alumno.ipn.mx (E.B.); jtamarizm@gmail.com (J.T.); jfdelgador@gmail.com (F.D.); 2 Instituto de Química, Universidad Nacional Autónoma de México, Circuito Exterior, Ciudad Universitaria, Coyoacán, Mexico City 04510, Mexico; eligom@iquimica.unam.mx (E.G.); cescalantep1700@alumno.ipn.mx (C.H.E.); 3 Departamento de Microbiología, Laboratorio de Biología Molecular de Bacterias y Levaduras, Escuela Nacional de Ciencias Biológicas, Instituto Politécnico Nacional, Prolongación de Carpio y Plan de Ayala S/N, Colonia Santo Tomás, Mexico City 11340, Mexico; mvillat@ipn.mx; 4 Departamento de Fisiología, Escuela Nacional de Ciencias Biológicas, Instituto Politécnico Nacional, Av. Wilfrido Massieu S/N, Unidad Adolfo López Mateos, Mexico City 07738, Mexico

**Keywords:** pyrazoles, 1,3-dihydrooxazoles, molecular modeling, molecular docking, antifungal activity

## Abstract

The increase in multi-drug resistant *Candida* strains has caused a sharp rise in life-threatening fungal infections in immunosuppressed patients, including those with SARS-CoV-2. Novel antifungal drugs are needed to combat multi-drug-resistant yeasts. This study aimed to synthesize a new series of 2-oxazolines and evaluate the ligands in vitro for the inhibition of six *Candida* species and in silico for affinity to the CYP51 enzymes (obtained with molecular modeling and protein homology) of the same species. The 5-(1,3-diphenyl-1*H*-pyrazol-4-yl)-4-tosyl-4,5-dihydrooxazoles **6a-j** were synthesized using the Van Leusen reaction between 1,3-diphenyl-4-formylpyrazoles **4a-j** and TosMIC **5** in the presence of K_2_CO_3_ or KOH without heating, resulting in short reaction times, high compound purity, and high yields. The docking studies revealed good affinity for the active site of the CYP51 enzymes of the *Candida* species in the following order: **6a-j** > **4a-j** > fluconazole (the reference drug). The in vitro testing of the compounds against the *Candida* species showed lower MIC values for **6a-j** than **4a-j**, and for **4a-j** than fluconazole, thus correlating well with the in silico findings. According to growth rescue assays, **6a-j** and **4a-j** (like fluconazole) inhibit ergosterol synthesis. The in silico toxicity assessment evidenced the safety of compounds **6a-j**, which merit further research as possible antifungal drugs.

## 1. Introduction

In recent years, yeasts of the genus *Candida* have been responsible for a sharp increase in invasive fungal infections in patients with an immunosuppressed system, including those with the SARS-CoV-2 virus. As a result, there have been significant complications in a considerable number of critically ill hospitalized patients, sometimes leading to death [1,2]. This situation is due in large part to the rise in multi-drug-resistant fungi. Prior to the extensive clinical administration of antifungal drugs such as azoles, studies had found a prevalence of fungal species susceptible to all classes of antifungal drugs. Unfortunately, the widespread use of antifungals over the years has gradually undermined the effectiveness of the current treatments for many kinds of invasive fungal infections. It is more common each year to find species of fungi with resistance to one or more types of drugs, leading to therapeutic failure in many cases [3,4].

Candidemia is one of the most common types of invasive fungal infections [5,6,7]. The main species involved is *C. albicans*, followed by *C. glabrata*, *C. parapsilosis*, *C. tropicalis*, and *C. krusei*. The frequency of incidence depends on the population, geographic region, patient age, and previous exposure to antifungal drugs [8,9]. Relatively new species, such as *C. auris* and *C. haemulonii*, have been emerging as pathogens worldwide and some of their strains have become multi-drug-resistant. These species are considered a public health problem because of their high mortality, tendency to provoke nosocomial outbreaks, and lack of susceptibility to drugs [10,11,12,13]. Hence, new compounds are needed that can effectively treat multi-drug resistant fungal infections.

The lanosterol 14α demethylase enzyme (CYP51) is an important target for combatting fungal infections. In fungal cells, it catalyzes lanosterol 14α demethylation through a series of successive oxidation reactions to form a key intermediate in ergosterol synthesis. Ergosterol is an essential component of the fungal cell membrane, maintaining its fluidity, integrity, and permeability. Thus, the inhibition of CYP51 has become a key strategy for the development of new antifungal drugs [14,15].

In the search for new antifungal agents, interest has been shown in 2-oxazolines (also known as 4,5-dihydrooxazoles), which are partially saturated analogues of oxazoles [16]. 1,3-oxazoles are aromatic five-membered heterocyclic compounds moderately rich in π-electrons [17]. The attractiveness of 2-oxazolines for medicinal chemistry owes itself to their presence in the structure of a variety of compounds with antifungal [18], antiviral [19], anticancer [20], anti-inflammatory [21], antibacterial [22], antidiabetic [23], and/or antioxidant activity [24]. The potential antifungal activity of 2-oxazolines has recently been described in the literature in relation to various *Candida* species, including multi-drug-resistant species (e.g., *C. auris* and *C. haemulonii*). Other reports indicate that they are active against *Cryptococcus neoformans*, *Aspergillus fumigatus*, *Tilletia indica*, *Trichoderma*, *Psilocybe cubensis*, *Sphaerotheca fuliginea*, and *Phytophthora infestans* [18,25,26,27,28].

Other compounds of interest in the search for new antifungal agents are pyrazoles, a type of 1,2-diazole with two adjacent nitrogen atoms. These aromatic heterocycles are moderately rich in π-electrons [17] and are regarded as privileged structures since they are found in a large number of drugs and naturally occurring molecules with antifungal [29], antiparasitic [30], antimicrobial [31], anti-inflammatory [31], anticancer [32], antihypertensive [33], antiviral [34], and antidiabetic activity [35].

Due to the great therapeutic relevance of the 2-oxazoline and 1*H*-pyrazole systems, several methods have been developed to synthesize them. One method for achieving the 2-oxazoline system involves an intramolecular cyclization reaction of the *5-exo-trig* type in accordance with Baldwin’s rules. It takes place in derivatives of salicylic acid and is promoted by thionyl chloride. [18,25,26,27]. A characteristic example for the other methodologies is the reaction between benzaldehydes, with 4-phenyl-thiosemicarbazide involving a hydrazone intermediate which reacts with benzoin to obtain 2-oxazolines [28]; another methodology is based on the (3 + 2) reaction between TosMIC **5** and an aromatic aldehyde in the presence of a base. This synthetic strategy generally leads to the corresponding oxazole via the oxidation of the primary oxazoline formed [36,37,38].

The aim of the current contribution was first to synthesize new 2-oxazolines with the Van Leusen reaction between a series of 1,3-diphenyl-4-formylpyrazoles **4a-j** and TosMIC **5** in the presence of K_2_CO_3_, and then evaluate the new compounds in silico and in vitro in relation to six *Candida* species. The series of hybrid molecules of 5-(1,3-diphenyl-1*H*-pyrazol-4-yl)-4-tosyl-4,5-dihydrooxazoles **6a-j** contained both the 2-oxazoline and 1*H*-pyrazole systems. Molecular docking studies conducted at the active site of the CYP51 enzymes of *Candida* spp. showed better binding energy values for ligands **3a-j** and **5a-j** than for the reference drug fluconazole. According to the in vitro antifungal tests, the MIC values were much lower (better inhibition of the yeasts) for both series of test compounds than for fluconazole **20**. Also, the MIC values were lower for **6a-j** than **4a-j**. Thus, the in silico and in vitro data correlated well.

## 2. Results and Discussion

### 2.1. General Strategy for Obtaining Compounds ***6a-j***

**Scheme 1:**
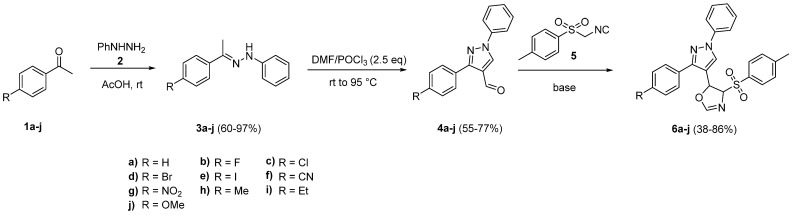
Synthesis of (4S*, 5S*)-5-(1,3-diphenyl-1*H*-pyrazol-4-yl)-4-tosyl-4,5-dihydrooxazoles **6a-j**.

The general synthetic strategy for obtaining (4S*, 5S*)-5-(1,3-diphenyl-1*H*-pyrazol-4-yl)-4-tosyl-4,5-dihydrooxazoles **6a-j** was based on three steps: (a) a series of the 1-phenyl-2-(1-phenylethylidene)hydrazines **3a-j** was prepared from the nucleophilic addition of phenylhydrazine **2** with the respective substituted acetophenones **1a-j**, which is a reaction catalyzed by glacial acetic acid; (b) then, the pyrazole ring was formed, followed by a formylation at C-4 of the heterocyclic system in a single step by using 2.5 equivalents of POCl_3_/DMF (in accordance with the Vilsmeier–Haack conditions) to obtain 1,3-diphenyl-1*H*-pyrazole-4-carbaldehydes **4a-j**; (c) finally, a cycloaddition (3 + 2) was carried out between the series of aldehydes **4a-j** and the anion of para-toluenesulfonylmethylisocyanide **5** under basic conditions to furnish (4S*, 5S*)-5-(1,3-diphenyl-1*H*-pyrazol-4-yl)-4-tosyl-4,5-dihydrooxazoles **6a-j** in good yields (Scheme 1). To our knowledge, the synthesis of this type of dihydrooxazole has not yet been reported.

### 2.2. (E)-1-Phenyl-2-(1-Phenylethylidene)Hydrazones ***3a-j***

Phenylhydrazones **3a-j** were synthesized in good yields and with very short reaction times (Table 1). As expected, the reaction took less time for molecules with the electron-withdrawing groups NO_2_ and CN in the *para* position of the aromatic ring, given that these groups exert a negative mesomeric effect and make the ketone more electrophilic. With high-resolution mass spectrometry (HRMS), it was possible to detect the molecular ion in all compounds of the series **3a-j** (see Supplementary Material, Figures S1–S250, Tables S1–S6). In the IR spectra, the C=N signal for **3a-j** was observed between 1640 and 1690 cm^−1^.

### 2.3. 1,3-Arylphenyl-1H-Pyrazole-4-Carbaldehydes ***4a-j***

The preparation of the 1,3-diphenyl-1*H*-pyrazole-4-carbaldehyde **4** system was achieved through the reaction of (*E*)-1-phenyl-2-(1-phenylethylidene)hydrazones **3** with an excess of POCl_3_ (3.0 equiv) and DMF. This process involves an attack by the tautomeric hydrazone **7** on the chloroiminium ion **8** to give intermediate **9**, which undergoes a 5-exo-trig cyclization to afford **11**. The subsequent aromatization of **11** allows for the formation of the pyrazole **14**, which reacts at C-4 with a second chloroiminium ion to provide **17**. With an aqueous workup, **17** is hydrolyzed to deliver the 1,3-diphenyl-1*H*-pyrazole-4-carbaldehydes **4a-j** (see Scheme S1 in Supplementary Material).

As expected, the highest yields (72–76%) were obtained with electron-withdrawing substituents at the *para* position (e.g., F, Cl, Br, I, and NO_2_; Table 2). Since these substituents increase the acidity of the alpha hydrogens, they favor tautomerization to furnish **7**. The subsequent reaction between **7** and **8** results in **4**. On the other hand, the electron-donating substituents (e.g., Me, Et, and OMe) decrease the acidity of the alpha hydrogens, thus generating lower yields (55–68%).

Compounds **4a-j** were fully characterized. In the ^1^H NMR spectra, the singlet signal of the formyl group was observed from 10.03 to 10.09 ppm. The ^13^C assignment was made using heteronuclear single quantum coherence (HSQC) spectroscopy and heteronuclear multiple-bond correlation (HMBC) spectroscopy. Key correlations were found in all compounds, including a triple bond interaction of C3′ with protons H5′, H1, and H2‴, and double bond interactions of C4′ with H1 and H5′. There was also a double bond interaction of C1‴ with H2‴ and C1″ with H2″, and a triple bond interaction of C1‴ with H3‴ and C1″ with H3″. The IR spectroscopy revealed the formyl group signal at 1668-1682 cm^−1^ for all compounds of the series **4a-j**. By means of HRMS, molecular ions were identified for all compounds.

### 2.4. (4S*, 5S*)-5-(1,3-Diphenyl-1H-Pyrazol-4-yl)-4-Tosyl-4,5-Dihydrooxazoles ***6a-j***

In view of the biological importance of dihydrooxazoles, a synthetic route to access 5-(1,3-diphenyl-1*H*-pyrazol-4-yl)-4-tosyl-4,5-dihydrooxazoles **6a-j** was explored. Compounds **6a-j** are herein reported for the first time. The synthesis took place through a (3 + 2) cycloaddition of 1,3-diphenyl-1*H*-pyrazole-4-carbaldehydes **4a-j** with the anion of *para*-toluenesulfonylmethylisocyanide **5**, promoted by a base. Cycloaddition reactions represent a powerful tool that can be used for the construction of new and complex molecules by reacting a dipole with a dipolarophile. In this case, the 1,3-dipolar cycloaddition involved the para-toluenesulfonylmethylisocyanide TosMIC **5** as the dipole and 1,3-diphenyl-1*H*-pyrazole-4-carbaldehydes **4a-j** as dipolarophiles. The reactivity and selectivity of each carbaldehyde were examined within the context of the overall efficiency of the process.

Initially, molar equivalents of the aldehydes **4a-j** were reacted with TosMIC **5** and K_2_CO_3_, finding quite low yields and the formation of byproducts. Therefore, the proportion of the reactants was modified to 1.0 equiv mol of **4**, 1.8 equiv mol of TosMIC **5**, and an excess of the base (2.5 equiv mol of K_2_CO_3_). The reaction was carried out at room temperature (rt) for 3 h to furnish the series of compounds **6a-j**. Nevertheless, the yields were still low. Other bases were tested as well (e.g., morpholine, DBU, and K_2_HPO_4_), but good yields were not achieved. Consequently, it was decided to conduct different tests involving a protic solvent (MeOH) and the modification of the equivalents of the base. It was found that higher yields were obtained in the same reaction time by reacting **4g** with a slight excess of TosMIC **5** (1.2 equiv mol) and 1.2 equiv mol of KOH. Once the reaction conditions were optimized, a variety of formyl pyrazoles (some with electron-donating groups and others with electron-withdrawing groups) were employed to determine the scope of the methodology, resulting in ten new derivatives of the 5-(1,3-diphenyl-1*H*-pyrazol-4-yl)-4-tosyl-4,5-dihydrooxazole system **6a-j** (Table 3).

Compounds **6a-j** were fully characterized. Although there was no evidence of a formyl signal in the ^1^H NMR spectra, the signals of the methyl group and tosyl group were identified at 2.40 to 2.47 ppm. The characteristic signals of the H4 and H5 protons of the dihydrooxazole ring were found between 5.79 and 6.07 ppm. Based on NOESY experiments performed on **6d**, **6g**, and **6h**, the general tendency for the H4 and H5 protons to adopt a *trans* configuration could be observed (see Figures S167 and S200 in Supplementary Material). Furthermore, the H2 signal of the oxazole ring was observed in all cases from 7.41 to 7.72 ppm. The ^13^C NMR spectra displayed signals of the methyl group from 21.2 to 21.4 ppm and of C4, C5 from 71.7 to 90.8 ppm. The other aromatic carbons were also observed. In the HMBC experiment, the triple bond interaction of C2 with the H4 and H5 protons of the dihydrooxazole ring can be appreciated.

A plausible mechanism for the formation of 5-(1,3-diphenyl-1*H*-pyrazol-4-yl)-4-tosyl-4,5-dihydrooxazole **6a** is based on the initial deprotonation of para-toluenesulfonylmethylisocyanide **5** promoted by KOH to provide carbanion **18**, which reacts with the formyl group of **4a** via a (3 + 2) cycloaddition to generate intermediate **19a**. Finally, the latter is protonated with the protic solvent in a subsequent stage to deliver dihydrooxazoles **6a** (see Scheme S2 in Supplementary Material).

### 2.5. Physicochemical, Drug-Likeness, Pharmacokinetic, and Toxicological Properties of 1,3-Diphenyl-1H-Pyrazole-4-Carbaldehydes ***4a-j***, (4S*, 5S*)-5-(1,3-Diphenyl-1H-Pyrazol-4-yl)-4-Tosyl-4,5-Dihydrooxazoles ***6a-j***, and Fluconazole ***20***

With the Osiris DataWarrior version 6.1.0 (Allschwil, Switzerland) [39] program and the SwissADME (Laussane, Switzerland) [40] server, several pharmacological properties were determined for 1,3-diphenyl-1*H*-pyrazole-4-carbaldehydes **4a-j**, (4S*, 5S*)-5-(1,3-diphenyl-1*H*-pyrazol-4-yl)-4-tosyl-4,5-dihydrooxazoles **6a-j**, and fluconazole **20**, with the aim of evaluating their efficacy and behavior in the human body (Table 4 and Table 5). The first descriptor to be examined was lipophilicity (the octanol/water partition coefficient, Log P) [41]. This parameter provides evidence of many aspects of the performance of a drug, including solubility, membrane permeability, absorption in the gut, distribution through the bloodstream by binding to plasma proteins, the crossing of the blood–brain barrier (BBB), entry into organs, metabolism, and clearance from the body (ADME properties). The partition coefficient (P = [organic]/[aqueous]) is defined as the ability of a compound to differentially dissolve in a mixture of water and lipids/organic solvents [42]. Permeability is high with Log P values close to 5 and low with negative values. The Log P values of **4a-j** and **6a-j** are acceptable; all values are under 5.0 and none of them are negative. On the other hand, fluconazole **20** has a negative log P value (−0.1089). The highest values (3.23 and 4.06) correspond to **4i** and **6i** (R = Et). Compounds **4f** and **6f**, containing the polar substituent (R = NO_2_), had Log P values of 1.59 and 2.41, respectively.

Aqueous solubility constitutes a key property of chemical substances because it governs important phenomena in drug design, agrochemical design, and protein–ligand binding. It is quantified as the maximum amount of a compound (i.e., the solute) that can be dissolved in each volume of water. It depends on physical conditions such as temperature and pressure. According to the calculated values, all the 5-(1,3-diphenyl-1*H*-pyrazol-4-yl)-4-tosyl-4,5-dihydrooxazoles **6a-j** and 1,3-diphenyl-1*H*-pyrazole-4-carbaldehydes **4c-f** are moderately soluble in water (−6 > Log S > −4), while a few of them (**4a**, **4b**, and **4g-j**) have only a slight tendency to solubilize (−4 > Log S > −2) [43].

Another effective descriptor for predicting the drug solubility and transport properties of a molecule is the polar surface area (PSA), which has been widely used in the study of drug transport properties such as intestinal absorption [44] and penetration of the BBB [45]. It is the sum of the contributions to the molecular surface area (usually van der Waals forces) of polar atoms (e.g., oxygen and nitrogen) and slightly polar atoms (S and P), and the hydrogen atoms attached to them. PSA values of 34.89 to 127.75 Å^2^ were found in **4a-j** and **5a-j**, suggesting acceptable permeability. Compounds **4g** and **6g**, with nitro substituents and thus a greater number of electronegative atoms, showed the highest PSA values (80.71 and 127.75 Å^2^), and consequently the lowest permeability of cell membranes [46].

Ligand efficiency (LE) is the binding energy per hydrogen atom and is interpreted as a measurement of the goodness of interaction between a given compound and its target protein. It is calculated by dividing the free binding energy of each molecule by the number of heavy non-hydrogen atoms in the structure (LE = ΔG_interaction_/[number of heavy non-hydrogen atoms]) [47]. Therefore, it takes the affinity and size of the ligand molecule into account, but not the size and topological properties of the molecular target [48].

High gastrointestinal absorption was evidenced for the entire series of compounds **4a-j** along with **6a-f** and **6h-j**, which coincides with the values obtained for the polar surface area (in all cases being < 140 Å^2^). Only **6g** seems to have limited gastrointestinal absorption, which is caused by the polarity of the nitro group.

According to the results, **4a-j** can cross the BBB (except for **4g** due to the electronegativity of the nitro group), but compounds **6a-j** could not cross the BBB because of their sulfone group. Likewise, fluconazole **20** does not pass through this barrier.

A comparative study of the possible risk of toxicity of compounds **4a-j** and **6a-j** was made through computational tools (DataWarrior) (Table 6 and Table 7), finding no evidence of tumorigenicity, mutagenicity, irritation, or reproductive effects. The results demonstrate a wide margin of safety between the effective dose and the dose that could cause any serious risk to human health.

### 2.6. Homology Modeling of Lanosterol 14-Alpha Demethylase (CYP51) from C. auris, C. dubliniensis, C. glabrata, C. haemulonii, and C. krusei

**Figure 1:**
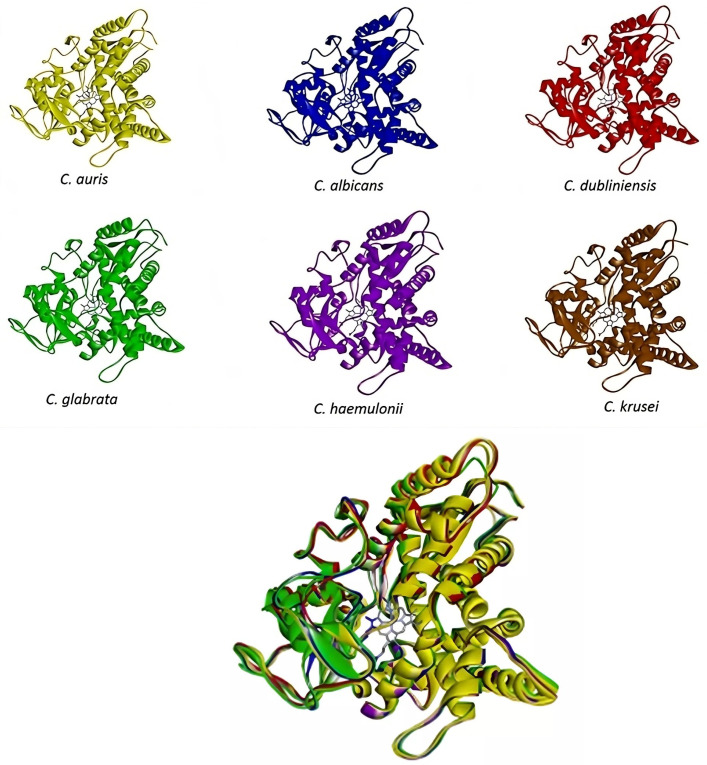
**Above**: 3D representation of the CYP51 enzymes of *C. albicans* (blue), *C. glabrata* (green), *C. auris* (yellow), *C. dubliniensis* (red), *C. haemulonii* (purple), and *C. Krusei* (brown). **Below**: the overlap of each of the modeled CYP51 enzymes with the CYP51 of *C. albicans*.

Once the azoles **4a-j** and **6a-j** were obtained, an evaluation was made of the recognition of and affinity for the active site of the CYP51 enzyme of different *Candida* species: *C. auris*, *C. dubliniensis*, *C. glabrata*, *C. haemulonii*, and *C. krusei*. The homology modeling of the CYP51 proteins of the aforementioned species was carried out, using the 3D structure of CYP51 *C. albicans* (PDB code 5FSA) as the template [14]. The identity of the protein of each *Candida* strain with the CYP51 *C. albicans* was greater than 50%. According to the Ramachandran plot [49] for all the CYP51 proteins, including CYP51Cau (from *C. auris*), CYP51Cdu (from *C. dubliniensis*), CYP51Cha (from *C. haemulonii*), and CYP51Ckr (from *C. krusei*) (Figures S242–S246, Supplementary Materials), over 90% of the amino acid residues fall within the allowed regions with respect to the amino acids of the CYP51 enzyme of *C. albicans*; the close structural similarity between the 3D structures was noted. Figure 1 illustrates the models obtained from these five CYP51 enzymes (above), as well as the overlap of each of the modeled CYP51 enzymes with the CYP51 of *C. albicans* (below). The RMSD values were also determined to evaluate the structural alignment of the 3D models of the CYP51 enzymes of the *Candida* spp. that were tested. In all cases, the values were less than 1.2 Å, thus evidencing the high quality of the 3D models obtained. The results are summarized in Table S4 (see Supplementary Material).

### 2.7. Molecular Docking

To determine whether the compounds have affinity with the active site of the lanosterol 14-alpha demethylase enzyme of the different *Candida* species, an exhaustive docking analysis was carried out for all the compounds (**4a-j**, **6a-j**, and fluconazole **20**) at the active site of the CYP51 enzyme of *C. albicans*, *C. glabrata*, *C. auris*, *C. dubliniensis*, *C. haemulonii*, and *C. krusei* (Table 8). A higher affinity with the active site of *C. albicans* was displayed by pyrazoles **4a-j** versus **20**, which was evidenced by better interaction free energy values (−8.92 to −9.81 kcal/mol vs. −7.29 kcal/mol). This same trend was observed for compounds **4a-j** on the other CYP51 proteins (of *C. auris*, *C. dubliniensis*, *C. glabrata*, *C. haemulonii*, and *C. krusei*). Hence, these pyrazole derivatives form enzyme–ligand complexes with greater stability than the enzyme–fluconazole complex.

The affinity for the CYP51Ca enzyme (from *C. albicans*) was even greater for (4S*, 5S*)-5-(1,3-pheny-1*H*-pyrazol-4-yl)-4-tosyl-4,5-dihydrooxazoles **6a-j** than for compounds **4a-j** (−12.44 to −14.23 kcal/mol vs. −8.92 to −9.81 kcal/mol, respectively; Table 8). The difference in affinity is due to the hydrophilic and hydrophobic intermolecular interactions presented by both the dihydrooxazole ring and the tosyl group of **6a-j**, causing a strong affinity with the CYP51 active site of the distinct *Candida* species. Such interactions with the amino acid residues of CYP51 are not found with the 4-formylpyrazoles **4a-j**, which only interact through the formyl group.

In a complementary manner, we carried out the molecular docking analysis using the GOLD v.5.6.3 program. As a scoring function, we use ChemScore fitness DG, which represents the total free energy change that occurs on ligand binding. The docking results are summarized in Table S3. As we can see, a similar trend is observed with respect to the results obtained by Autodock4; in all cases compounds **6a-j** and **4a-j** showed better binding energies when compared with the reference drug fluconazole **20**. It is important to note that in general, compounds **6a-j** showed even greater affinity to the active site of the CYP51 of *Candida* spp. with respect to derivatives **4a-j**. This is due to a greater number of interactions with the amino acids of the active site of the enzyme. These results confirm that the compounds obtained, **6a-j** and **4a-j**, act at the same level as fluconazole.

Table 9 shows the residues involved in the ligand–receptor interaction as well as the different hydrophobic and hydrophilic interactions found for 1,3-diaryl-1*H*-pyrazole-4-carbaldehydes **4a-j**, (4S*, 5S*)-5-(1,3-diaryl-1*H*-pyrazol-4-yl)-4-tosyl-4,5-dihydrooxazoles **6a-j**, and fluconazole **20**. The two series of test compounds and the reference drug all interact with key amino acids of the active site of the CYP51Ca enzyme, such as Thr122, Phe126, Ile131, Tyr132, Phe228, Gly307, and Thr311. All of them also interact with the prosthetic group Hem580, which is known to be essential for the catalytic activity of the enzyme, as previously reported for azole derivatives [14,50,51].

**Figure 2:**
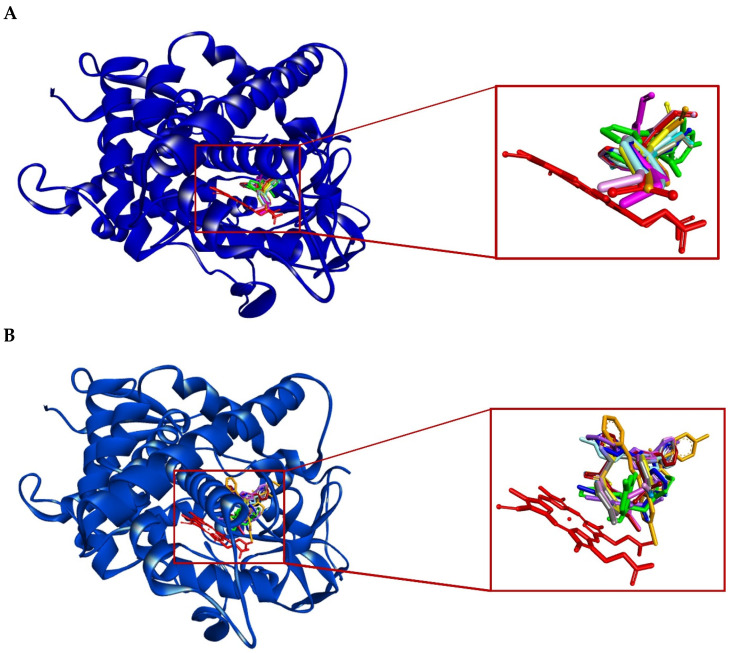
The binding mode at the active site of the CYP51 enzyme of *C. albicans*: (**A**) for fluconazole **20** and the 1,3-diaryl-1*H*-pyrazole-4-carbaldehydes. The heme group is in red, fluconazole **20** (green), **4a** (blue), **4b** (cyan), **4c** (yellow), **4d** (orange), **4e** (purple), **4f** (turquoise), **4g** (red), **4h** (gray), **4i** (fuchsia) and **4j** (pink)., and (**B**) for the (4S*, 5S*)-5-(1,3-diaryl-1*H*-pyrazol-4-yl)-4-tosyl-4,5-dihydrooxazoles the heme group is in red, fluconazole **20** (green), **6a** (blue), **6b** (cyan), **6c** (yellow), **6d** (orange), **6e** (purple), **6f** (turquoise), **6g** (red), **6h** (gray), **6i** (fuchsia) and **6j** (pink).

A greater number of hydrophobic versus hydrophilic interactions are observed (Figure 2A,B, Figures S247 and S248). The most common interactions for **4a-j** are as follows: π-sigma with Ile131, π-alkyl with Ile304 and Ile376, π-cation with Hem580, and π-sigma with Hem580. Compounds **6a-d** and **6f-i** exhibit a T-shaped π–π interaction with the hydrophobic amino acids Phe228 and Phe233. For **6c-j**, there is a π–alkyl-type interaction with Pro230, while **6a-b**, **6d-e**, and **6i** interact with Ile376 through a π–alkyl-type interaction. Meanwhile, **6a-b** and **6f-i** show a π–sulfur-type interaction with Met508, and **6c**, **6f**, **6g-h**, and **6j** display an alkyl-type interaction with Hem580.

Regarding hydrophilic interactions, a conventional hydrogen bond was observed between the hydroxyl group of fluconazole and the oxygen of the carbonyl group of Tyr132. There were also carbon–hydrogen bond interactions with Gly307. Of the series of pyrazoles **4a-j**, only compound **4j** exhibited a carbon–hydrogen bond interaction between the methyl of the OMe group and Gln142. 1,3-dihydrooxazoles **6a-b** and **6e** exhibited a conventional hydrogen bond with the hydroxyl group in C-4 of Tyr118. This interaction could also be appreciated between the oxygen of the sulfone group of **6a-b** and the hydroxyl group of the aromatic ring of Tyr132. A carbon–hydrogen bond type interaction is also present between the C-H bond in C5 of the pyrazole ring of compounds **6g-j** and the oxygen of the carbonyl group of Met508.

Compounds **4a-j**, **6a-j**, and **20** also interact with key residues of the active site of CYP51Cg (from *C. glabrata*) (Table S2, Figures S249 and S250 of Supplementary Material), such as Thr78, Phe82, Tyr88, Phe184, Gly258, Gly262, and Hem478. Several hydrophobic interactions were found. An amide–π-stacking-type interaction with Gly258 was observed for **4a-j**, **6a-e**, and **6g-i**. An π–alkyl-type interaction was evident for **4a-c**, **4e-i**, and **6a-i** with Val259 as well as for **4a-e**, **4g-j**, **6a-e**, and **6g-j** with Hem478. The latter prosthetic group also interacts with the aromatic rings in **4a-j** through a π–π stacking interaction.

As shown in Figure 2A,B, and Figures S247–S250, compounds **4a-j**, **6a-j**, and fluconazole **20** bind to the active site of CYP51Ca and CYP51Cg, as described in other reports on azole derivatives. All the compounds display the same binding mode. Hence, (4S*, 5S*)-5-(1,3-diaryl-1*H*-pyrazol-4-yl)-4-tosyl-4,5-dihydrooxazoles **6a-j** and 1,3-diaryl-1*H*-pyrazole-4-carbaldehydes **4a-j** may act with the same mechanism of action as **20**. To our knowledge, there are no reports on the binding mode of the series of azoles **6a-j** and **4a-j** with the active site of any of the CYP51 enzymes of *Candida* species.

### 2.8. Antifungal Activity

#### 2.8.1. Antifungal Effect of the 1,3-Diaryl-1*H*-Pyrazole-4-Carbaldehydes **4a-j** and (4S*, 5S*)-5-(1,3-Diaryl-1*H*-Pyrazol-4-yl)-4-Tosyl-4,5-Dihydrooxazoles 6a-j on *Candida* spp.

The susceptibility of six *Candida* species to the 1,3-diaryl-1*H*-pyrazole-4-carbaldehydes **4a-j** and (4S*, 5S*)-5-(1,3-diphenyl-1*H*-pyrazol-4-yl)-4-tosyl-4,5-dihydrooxazoles **6a-j** was examined in vitro. The MIC_70_ and MIC_90_ values were lower for **4a-j** and **6a-j** than for fluconazole **20** (Table 10 and Table 11). It is well known that some pyrazole derivatives distinct from those proposed herein have antifungal activity against *Candida* species. For example, the MIC values of pyrazole derivatives have previously been reported at values similar to or a little higher than those determined in this study when tested on *C. albicans*, *C. glabrata*, *C. parapsilosis*, *C. krusei*, *C. tropicalis*, and *C. famata* [52,53,54,55,56]. The importance of the present pyrazoles is that they are new and are used as intermediates for the synthesis of dihydrooxazoles.

Likewise, good antifungal activity against *Candida* spp. (*C. albicans*, *C. tropicalis*, and *C. krusei*) has been described for other derivatives of dihydrooxazoles in three recent works [26,27,28]. However, there are few reports dealing with the effect of dihydrooxazoles on a wide spectrum of *Candida* spp., despite the importance of such a study given the multi-drug resistance that has developed in many such species (including the relatively new species of *C. auris* and *C. haemulonii*) [57].

Hence, the current contribution is quite relevant because **4a-j** and **6a-j** exhibited better antifungal activity than **20** against a large number of *Candida* species. In this study, the best inhibitory activity on the greatest number of the *Candida* spp. was found for the compounds with halogenated substituents. The results demonstrate the merit of continuing to design structures analogous to these series that could possibly improve therapeutic antifungal activity on *Candida* species.

#### 2.8.2. Rescue of the Growth of *Candida* spp. by Adding Ergosterol

**Figure 3:**
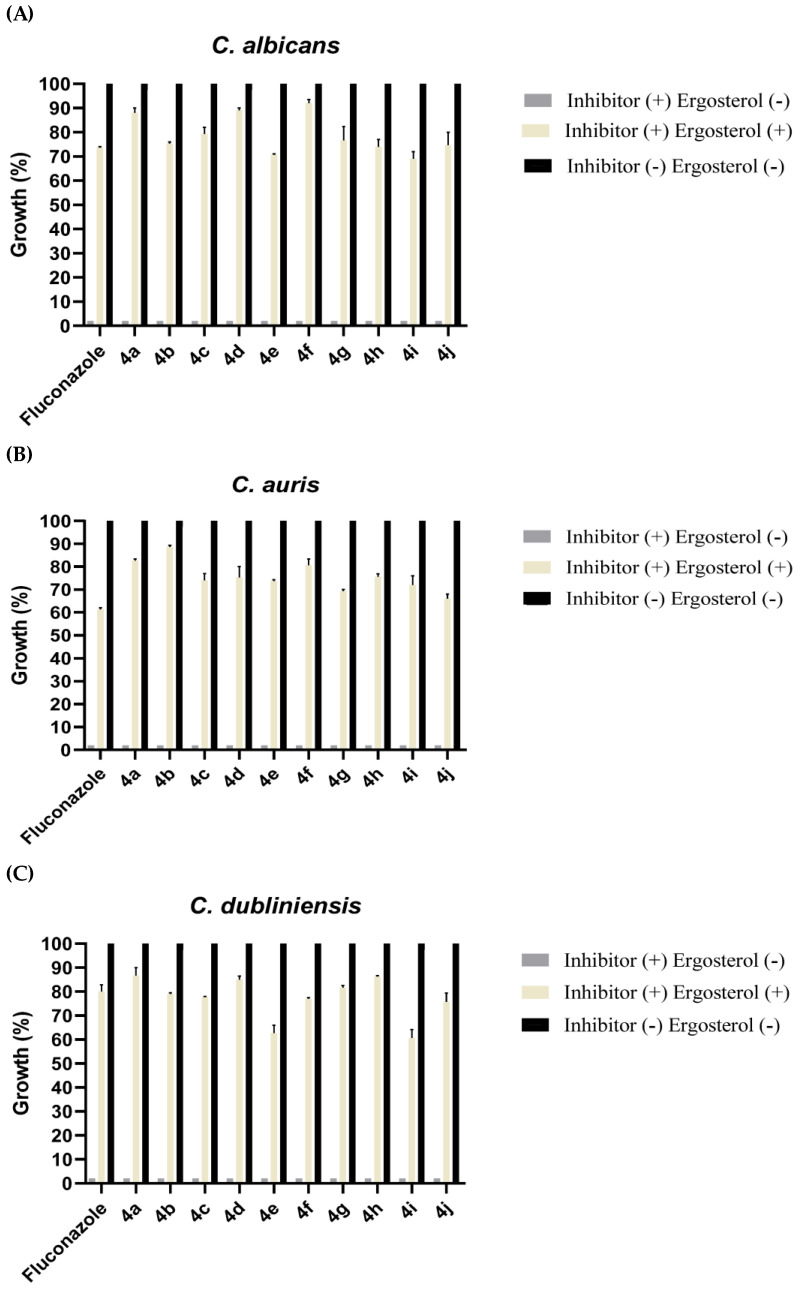

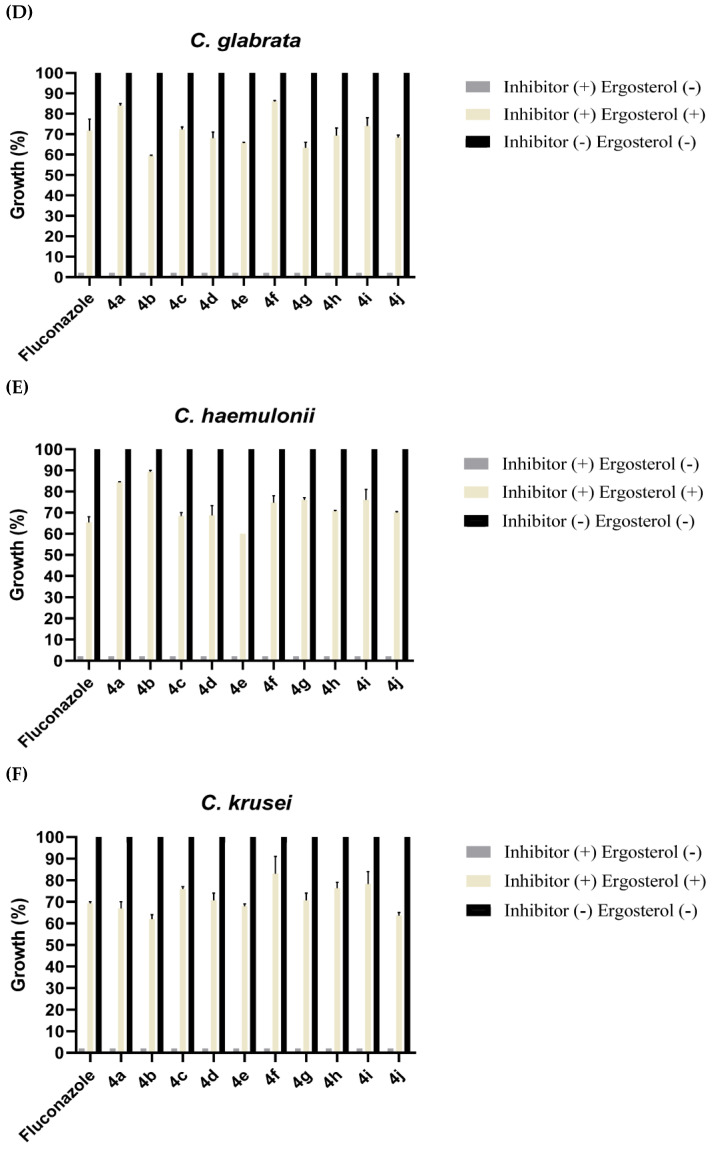
(**A**–**F**) Growth rescue of the six *Candida* species evaluated herein, promoted by adding ergosterol after yeast growth had been stopped by treatment with one of the 1,3-diaryl-1*H*-pyrazole-4-carbaldehydes **4a-j** or fluconazole **20**. The gray bars illustrate the mean ± standard error (SE) of the growth of yeasts cultured in the presence of an inhibitor (+) and in the absence of ergosterol (−). The cream-colored bars portray the growth of yeasts in the presence of an inhibitor (+) and with the posterior addition of ergosterol (+). The black bars indicate the 100% growth of yeasts cultured in the absence of both an inhibitor (−) and ergosterol (−). The yeasts were cultured at 37 °C for 24 h and the optical density was determined in a microplate photometer at 620 nm. Values are expressed as the average of three independent assays. In all cases, *p* < 0.001 compared to the growth observed without any inhibitor (Student’s *t*-test).

**Figure 4:**
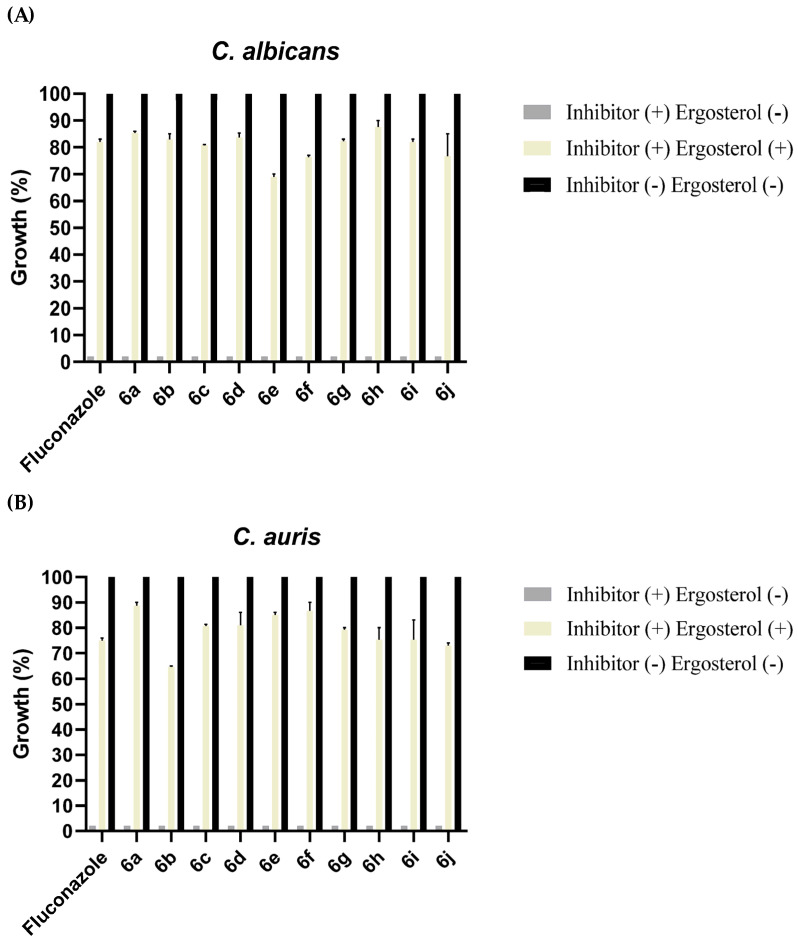

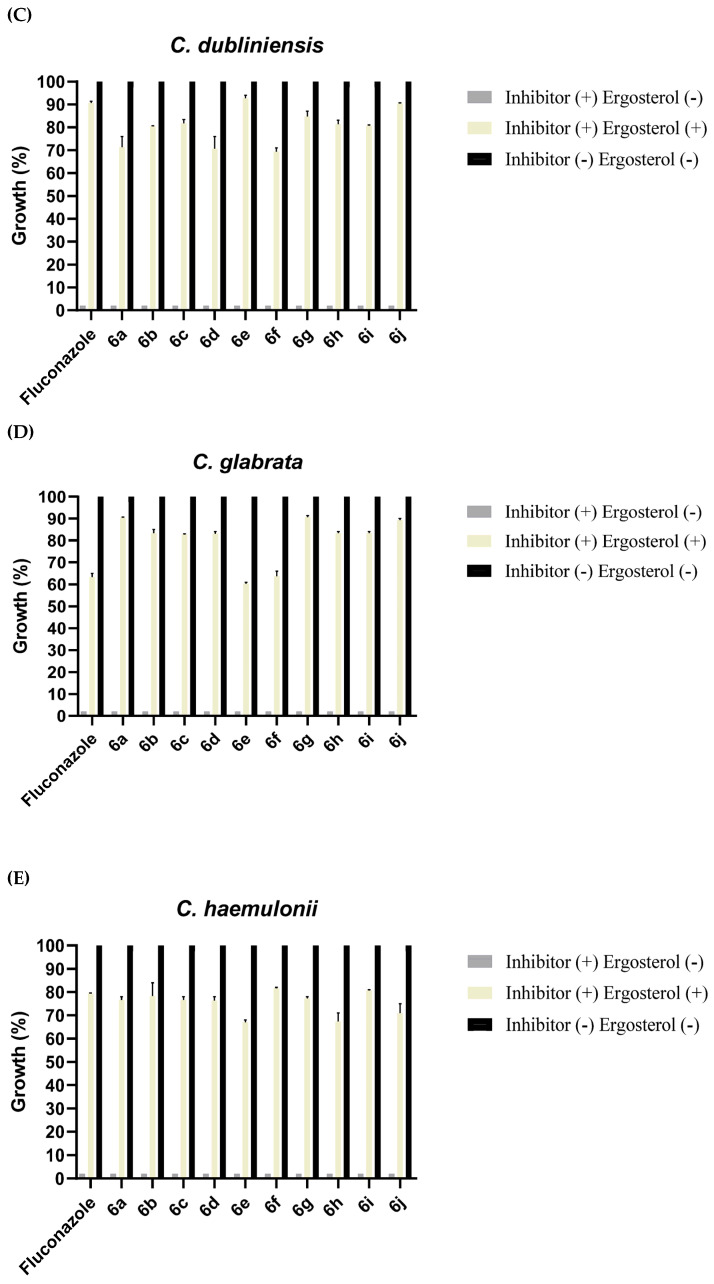

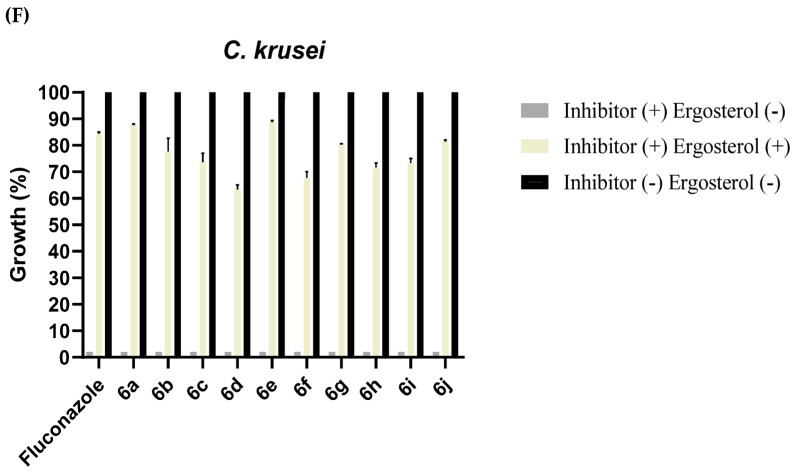
(**A**–**F**) Growth rescue of the six *Candida* species evaluated herein, promoted by adding ergosterol after yeast growth had been stopped by treatment with any of the (4S*, 5S*)-5-(1,3-diphenyl-1*H*-pyrazol-4-yl)-4-tosyl-4,5-dihydrooxazoles **6a-j** or fluconazole **20**. The gray bars illustrate the mean ± standard error (SE) of the growth of yeasts cultured in the presence of an inhibitor (+) and in the absence of ergosterol (−). The cream-colored bars portray the growth of yeasts in the presence of an inhibitor (+) and with the posterior addition of ergosterol (+). The black bars indicate the 100% growth of yeasts cultured in the absence of both an inhibitor (−) and ergosterol (−). The yeasts were cultured at 37 °C for 24 h and the optical density was determined in a microplate photometer at 620 nm. Values are expressed as the average of three independent assays. In all cases, *p* < 0.001 compared to the growth observed without any inhibitor (Student’s *t*-test).

To explore whether (4S*, 5S*)-5-(1,3-diphenyl-1*H*-pyrazol-4-yl)-4-tosyl-4,5-dihydrooxazoles **6a-j** and 1,3-diaryl-1*H*-pyrazole-4-carbaldehydes **4a-j**, like fluconazole **20**, inhibit the biosynthesis of ergosterol, a growth rescue assay was carried out with six *Candida* species (Figure 3 and Figure 4). After yeast growth was inhibited by the test compounds, ergosterol was added to the yeast culture, and an increase in the growth of each well was found (treated with any of the compounds from the **4a-j** or **6a-j** series). Thus, **4a-j** and **6a-j** appear to interfere with ergosterol synthesis at some level (probably by CYP51 inhibition) in the six fungal species tested, as has been described in various studies on **20** and other inhibitors of *Candida* spp. [28,58,59,60,61].

## 3. Materials and Methods

### 3.1. Chemicals and Instruments

All glassware was oven-dried. Chemicals and solvents were purchased from commercial sources. Thin-layer chromatography (TLC) was performed with silica plates and visualized by using a UV lamp at 254 nm or iodine. The synthesized compounds were purified using flash column chromatography. Melting points were determined in an electrothermal capillary melting point apparatus. ^1^H (500 or 600 MHz) and ^13^C (125 or 150 MHz) NMR as well as HSQC, HMBC, and correlation spectroscopy (COSY) experiments were conducted on a Varian VNMR System (Santa Clara, CA, USA) or Bruker Avance III HD (Karlsruhe, Germany) with chloroform-*d* or dimethylsulfoxide (DMSO)*-d_6_* and C_6_D_6_ as a solvent and TMS as an internal standard. Most of the NMR assignments are based on extensive 2D homonuclear and heteronuclear experiments. HRMS analyses were recorded with electron ionization (70 eV) on a JEOL JSM-GC spectrometer (Akishima, Tokyo). IR spectra were acquired on a Bruker Tensor 27 spectrophotometer (Karlsruhe, Germany) with of the ATR technique.

### 3.2. General Procedure for the Synthesis of (E)-1-Phenyl-2-(1-Phenylethylidene)Hydrazones ***3a-j***

In a 50 mL balloon flask equipped with magnetic stirring, the corresponding acetophenone **1** (16.66 mmol) and 8 mL of glacial acetic acid were combined and stirred at rt for 10 min. Subsequently, phenylhydrazine **2** (16.66 mmol) was added and the stirring continued for 20 min. The reaction was monitored with TLC by using a 7:3 hexane–AcOEt system. After the reaction was completed, within a period between 10 to 40 min, the crude was treated with a saturated solution of NaHCO_3_. Then, the reaction crude was filtered under vacuum and washed with isopropanol/water (1:1 solution), followed by drying of the title compound under vacuum.

#### 3.2.1. (*E*)-1-Phenyl-2-(1-Phenylethylidene)Hydrazone **3a**

According to the general method, acetophenone **1a** (0.5 g, 4.16 mmol) and 8 mL of glacial acetic acid were combined and stirred, and then phenylhydrazine **2** (0.45 g, 4.16 mmol) was added. After stirring again, the reaction crude was filtered, washed, and dried under vacuum and 3.0 g (86%) was obtained as a light-yellow solid; m.p. = 87–88 °C (Lit. 104 °C [62]), Rf = 0.7 (hexane/AcOEt, 7:3). FT-IR (ATR) ν_max_ 3352, 3027, 1938, 1361, 1266, 1250, 749, 687 cm^−1^. HRMS (EI): *m/z* [M]^+^ calcd for C_14_H_14_N_2_: 210.1157; found: 210.1156.

#### 3.2.2. (*E*)-1-(1-(4-Fluorophenyl)Ethylidene)-2-Phenylhydrazone **3b**

Following the general method, 4-fluoroacetophenone **1b** (0.574 g, 4.16 mmol) and 8 mL of glacial acetic acid were combined and stirred, and then **2** (0.45 g, 4.16 mmol) was added. After stirring again, the reaction crude was filtered, washed, and dried under vacuum, and 0.76 g (92%) of **3b** was furnished as a clear white solid; m.p. 83–84 °C. FT-IR (ATR) *ν*_max_ 3347, 3054, 1678, 1598, 1503, 1230, 1142, 832, 749, 690 cm^−1^. ^1^H NMR (600 MHz, C_6_D_6_): *δ* = 1.39 (s, 3H), 6.85–6.89 (m, 4H), 7.15–7.16 (d, *J* = 6.0 Hz, 1H), 7.23–7.26 (m, 2H), 7.51–7.53 (m, 2H). ^13^C NMR (150 MHz, C_6_D_6_**)** δ 11.4, 114.0, 115.7 (*J* = 22.5 Hz), 120.9, 123.4, 127.8 (*J* = 10.5 Hz), 129.9, 136.2, 146.2, 163.4 (*J* = 244.5 Hz). HRMS (EI): *m/z* [M]^+^ calcd for C_14_H_13_N_2_F: 228.1063; found: 228.1066.

#### 3.2.3. (*E*)-1-(1-(4-Chlorophenyl)Ethylidene)-2-Phenylhydrazone **3c**

According to the general method, 4-chloroacetophenone **1c** (0.64 g, 4.16 mmol) and 8 mL of glacial acetic acid were combined and stirred, and then **2** (0.45 g, 4.16 mmol) was added. After stirring again, the reaction crude was filtered, washed, and dried under vacuum, and 0.90 g (89%) of **3c** was obtained as a light-yellow solid; m.p. 103–104 °C (Lit. 114 °C [63]). FT-IR (ATR) *ν*_max_ 3349, 3018, 1939, 1861, 1597, 1483, 1244, 1095, 810, 751, 695 cm^−1^. ^1^H NMR (600 MHz, C_6_D_6_): *δ* = 1.32 (s, 3H), 6.84-6.89 (m, 2H), 7.12 (d, *J* = 7.8 Hz, 2H), 7.15–7.18 (m, 2H), 7.22–7.26 (m, 2H), 7.43–7.46 (m, 2H). ^13^C NMR (150 MHz, C_6_D_6_): *δ* = 11.1, 114.1, 121.1, 127.4, 128.9, 129.9, 134.1, 138.4, 139.9, 145.9. HRMS (EI): *m/z* [M]^+^ calcd for C_14_H_13_N_2_Cl: 244.0767; found: 244.0756.

#### 3.2.4. (*E*)-1-(1-(4-Bromophenyl)Ethylidene)-2-Phenylhydrazone **3d**

Following the general method, 4-bromoacetophenone **1d** (0.82, 4.16 mmol) and 8 mL of glacial acetic acid were combined and stirred, and then **2** (0.45 g, 4.16 mmol) was added. After stirring again, the reaction crude was filtered, washed, and dried under vacuum, and 1.13 g (93%) of **3d** was obtained as a yellow solid; m.p. 116-117 °C (Lit. 126 °C [63]). FT-IR (ATR) *ν*_max_ 3339, 1592, 1573, 1494, 1479, 1395, 1247, 1151, 823, 750, 692 cm^−1^. ^1^H NMR (600 MHz, C_6_D_6_): *δ* = 1.30 (s, 3H), 6.83 (bs, 1H), 6.89 (t, *J* = 7.3 Hz, 1H), 7.11–7.14 (m, 2H), 7.25 (t, *J* = 7.9 Hz, 2H), 7.33–7.36 (m, 2H), 7.38–7.40 (m, 2H). ^13^C NMR (150 MHz, C_6_D_6_): *δ* = 11.1, 114.1, 121.1, 122.5, 127.7, 129.9, 131.9, 138.8, 139.8, 145.9. HRMS (EI): *m/z* [M]^+^ calcd for C_14_H_13_N_2_Br: 288.0262; found: 288.0261.

#### 3.2.5. (*E*)-1-(1-(4-Iodophenyl)Ethylidene)-2-Phenylhydrazone **3e**

According to the general method, 4-iodoacetophenone **1e** (1.02 g, 4.16 mmol) and 8 mL of glacial acetic acid were combined and stirred, and then **2** (0.45 g, 4.16 mmol) was added. After stirring again, the reaction crude was filtered, washed, and dried under vacuum, and 1.27 g (92%) of **3e** was obtained as an orange solid; m.p. 111-112 °C. FT-IR (ATR) *ν*_max_ 3342, 1596, 1494, 1478, 1391, 1248, 1147, 818, 750, 691 cm^−1^. ^1^H NMR (600 MHz, C_6_D_6_): *δ* = 1.30 (s, 3H), 6.85–6.89 (m, 2H), 7.13–7.14 (m, 2H), 7.23–7.26 (m, 2H), 7.27–7.28 (m, 2H), 7.54–7.56 (m, 2H). ^13^C NMR (151 MHz, C_6_D_6_): *δ* = 10.9, 94.0, 114.1, 121.1, 127.8, 129.9, 137.6, 137.9, 139.4, 145.9. HRMS (EI): *m/z* [M]^+^ calcd for C_14_H_13_N_2_I: 336.0124; found: 336.0126.

#### 3.2.6. (*E*)-4-(1-(2-Phenylhydrazono)Ethyl)Benzonitrile **3f**

Following the general method, 4-acetylbenzonitrile **1f** (0.82, 4.16 mmol) and 8 mL of glacial acetic acid were combined and stirred, and then **2** (0.45 g, 4.16 mmol) was added. After stirring again, the reaction crude was filtered, washed, and dried under vacuum, and 0.57 g (59%) of **3f** was produced as a yellow solid; m.p. 160-161 °C. FT-IR (ATR) *ν*_max_ 3332, 2218, 1599, 1568, 1490, 1252, 1154, 831, 746, 689 cm^−1^. ^1^H NMR (600 MHz, C_6_D_6_): *δ* = 1.23 (s, 3H), 6.90 (t, *J* = 7.3 Hz, 1H), 6.96 (bs, 1H, N*H*), 7.11–7.16 (m, 4H), 7.24–7.26 (m, 2H), 7.36–7.38 (m, 2H). ^13^C NMR (151 MHz, C_6_D_6_): *δ* = 10.8, 111.6, 114.2, 119.6, 121.7, 126.1, 130.0, 132.4, 138.6, 143.4, 145.4. HRMS (EI): *m/z* [M]^+^ calcd for C_15_H_13_N_3_: 235.1109; found: 235.1108.

#### 3.2.7. (*E*)-1-(1-(4-Nitrophenyl)Ethylidene)-2-Phenylhydrazone **3g**

According to the general method, 4-nitroacetophenone **1g** (0.6869 g, 4.16 mmol) and 8 mL of glacial acetic acid were combined and stirred, and then **2** (0.45 g, 4.16 mmol) was added. After stirring again, the reaction crude was filtered, washed, and dried under vacuum, and 0.90 g (85%) of **3g** was obtained as a red solid; m.p. 137-138 °C (Lit. 183 °C [62]). FT-IR (ATR) *ν*_max_ 3337, 1590, 1543, 1518, 1487, 1316, 1244, 1165, 1105, 1063, 848, 746, 689 cm^−1^. NMR (600 MHz, CDCl_3_): *δ* = 2.27 (s, 3H, H2″), 6.95 (t, *J* = 7.3 Hz, 1H, H4′), 7.21 (d, *J* = 7.3 Hz, 2H, H2′), 7.29-7.36 (m, 2H, H3′), 7.61 (bs, 1H, NH), 7.93 (d, *J* = 8.0 Hz, 2H, H2‴), 8.22 (d, *J* = 8.0 Hz, 2H, H3‴). ^13^C NMR (150 MHz, CDCl_3_): *δ* = 11.6 (C2″), 113.6 (C2′), 121.3 (C4′), 123.8 (C3‴), 125.9 (C2‴), 129.5 (C3′), 138.0 (C1″), 144.4 (C1′), 145.2 (C1‴), 147.0 (C4‴). HRMS (EI): *m/z* [M]^+^ calcd for C_14_H_13_N_3_O_2_: 255.1005; found: 255.1008.

#### 3.2.8. (*E*)-1-Phenyl-2-(1-(p-Tolyl)Ethylidene)Hydrazone **3h**

Following the general method, *p*-tolylacetophenone **1h** (0.55, 4.16 mmol) and 8 mL of glacial acetic acid were combined and stirred, and then **2** (0.45 g, 4.16 mmol) was added. After stirring again, the reaction crude was filtered, washed, and dried under vacuum, and 0.90 g (97%) of **3h** was generated as a white solid; m.p. 81-82 °C (Lit. 96 °C [63]). FT-IR (ATR) *ν*_max_ 3349, 1670, 1597,1499, 1480, 1250, 1141, 1110, 809, 748, 690 cm^−1^. ^1^H NMR (600 MHz, C_6_D_6_)**:** *δ* = 1.50 (s, 3H), 2.10 (s, 3H), 6.81–6.84 (m, 2H), 7.02 (d, *J* = 8.2 Hz, 2H), 7.15 (d, *J* = 7.4 Hz, 2H), 7.19–7.22 (m, 2H), 7.68 (d, *J* = 8.1 Hz, 2H). ^13^C NMR (150 MHz, C_6_D_6_): *δ* = 11.5, 21.5, 114.1, 120.7, 126.2, 129.6, 129.9, 137.4, 138.0, 141.5, 146.4. HRMS (EI): *m/z* [M]^+^ calcd for C_15_H_16_N_2_: 224.1314; found: 224.1313.

#### 3.2.9. (*E*)-1-(1-(4-Ethylphenyl)Ethylidene)-2-Phenylhydrazone **3i**

According to the general method, 4-ethylacetophenone **1i** (0.61 g, 4.16 mmol) and 8 mL of glacial acetic were combined and stirred, and then **2** (0.45 g, 4.16 mmol) was added. After stirring again, the reaction crude was filtered, washed, and dried under vacuum, and 0.96 g (95%) of **3i** was obtained as a yellow solid; m.p. 88–89 °C. FT-IR (ATR) *ν*_max_ 3348, 2960, 1598, 1496, 1251, 1139, 827, 749, 691 cm^−1^. ^1^H NMR (600 MHz, C_6_D_6_): *δ* = 1.12 (t, *J* = 7.6 Hz, 3H), 1.51 (s, 3H), 2.49 (q, *J* = 7.6 Hz, 2H), 6.87–6.90 (m, 2H), 7.12 (d, *J* = 8.0 Hz, 2H), 7.21 (d, *J* = 8.0 Hz, 2H), 7.26 (t, *J* = 8.0 Hz, 2H), 7.75–7.78 (m, 2H). ^13^C NMR (150 MHz, C_6_D_6_): *δ* = 11.54, 16.18, 29.3, 114.1, 120.7, 126.4, 128.4, 129.9, 137.7, 141.6, 144.5, 146.4. HRMS (EI): *m/z* [M]^+^ calcd for C_16_H_18_N_2_: 238.1470; found: 238.1474.

#### 3.2.10. (*E*)-1-(1-(4-Methoxyphenyl)Ethylidene)-2-Phenylhydrazone **3j**

Following the general method, *p*-methoxyacetophenone **1j** (0.62, 4.16 mmol) and 8 mL of glacial acetic were combined and stirred, and then **2** (0.45 g, 4.16 mmol) was added. After stirring again, the reaction crude was filtered, washed, and dried under vacuum, resulting in 0.85 g (86%) of **3j** as a white solid; m.p. 97–98 °C (Lit. 142 °C [63]). FT-IR (ATR) *ν*_max_ 3336, 1598, 1498, 1251, 1140, 1115, 1029, 834, 751, 696 cm^−1^. ^1^H NMR (600 MHz, C_6_D_6_): *δ* = 1.50 (s, 3H), 3.34 (s, 3H), 5.24 (bs, 1H), 6.85 (d, *J* = 8.9 Hz, 2H), 6.88 (t, *J* = 7.26 Hz, 1H), 7.22 (d, *J* = 7.26 Hz, 2H), 7.27 (f, *J* = 7.26 Hz, 2H), 7.74 (d, *J* = 8.9 Hz, 2H). **^1^**^3^C NMR (151 MHz, C_6_D_6_): *δ* = 11.5, 55.19, 114.0, 114.4, 120.6, 127.6, 129.9, 132.8, 141.4, 146.6, 160.5. HRMS (EI): *m/z* [M]^+^ calcd for C_15_H_16_N_2_O: 240.1263; found: 240.1262.

### 3.3. General Procedure for the Synthesis of 1,3-Diphenyl-1H-Pyrazole-4-Carbaldehydes ***4a-j***

In a two-necked balloon flask adapted to a reflux system, equipped with magnetic stirring and under N_2_ atmosphere, the corresponding hydrazone **3a-j** (6.0 mmol, 1.0 eq) was dissolved in 10.0 mL of anhydrous *N,N′*-dimethylformamide (DMF) and phosphorus oxychloride (POCl_3_) (18.0 mmol, 3.0 eq) was slowly added. The mixture was heated at 95 °C for 12 h before allowing the reaction crude to cool to rt. Subsequently, 10 mL of a solution of 5% NH_4_Cl was added and a saturated solution of NaHCO_3_ was poured into the mixture until a pH of 7 was reached. Eventually, the reaction crude was vacuum filtered and the solid was washed with water. Finally, the product was purified through recrystallization with a mixture of isopropanol/acetone/water (7:2:1).

#### 3.3.1. 1,3-Diphenyl-1*H*-Pyrazole-4-Carbaldehyde **4a**

Yellow solid (87%). *Rf* = 0.48 (hexane/EtOAc, 7:3), m.p. 141–142 °C (Lit. 140 °C [64]). FT-IR (ATR) *ν*_max_ 3125, 3060.42, 1669, 1523, 1452, 1226, 1044, 956, 813, 771, 753, 686 cm^−1^. NMR (600 MHz, CDCl_3_): *δ* = 7.39 (t, *J* = 7.0 Hz, 1H, H4″), 7.46-7.52 (m, 5H, H-3″, H-3‴, H4‴), 7.81 (d, *J* = 9.0 Hz, 2H, H2″), 7.83 (d, *J* = 8.4 Hz, 2H, H2‴), 8.55 (s, 1H, H5′), 10.06 (s, 1H, CHO). NMR (150 MHz, CDCl_3_): *δ* 119.9 (C2″), 122.6 (C4′), 128.1 (C4″), 128.9 (C2‴), 129.1 (C3‴), 129.4 (C-4‴), 129.8 (C3″), 131.1 (C5′), 131.5 (C1‴), 139.1 (C1″), 154.9 (C3′), 185.3 (CHO). HRMS (EI): *m/z* [M]+ calcd for C_16_H_12_N_2_O: 248.0950; found: 248.0951.

#### 3.3.2. 3-(4-Fluorophenyl)-1-Phenyl-1*H*-Pyrazole-4-Carbaldehyde **4b**

Light brown solid (82%). *Rf* = 0.44 (hexane/EtOAc, 7:3), m.p. 156–157 °C. FT-IR (ATR) *ν*_max_ 3125, 1668, 1594, 1519, 1505, 1454, 1223, 957, 840, 794, 751, 68 cm^−1^. NMR (600 MHz, CDCl_3_): *δ* 7.17–7.20 (m, 2H, H3‴), 7.40 (t, *J* = 7.7 Hz, 1H, H4″), 7.52 (t, *J* = 7.7 Hz, 2H, H3″), 7.78 (d, *J* = 7.7 Hz, 2H, H2″), 7.83–7.89 (m, 2H, H2‴), 8.53 (s, 1H, H5′), 10.03 (s, 1H, CHO). NMR (150 MHz, CDCl_3_): *δ* 115.7 (*J* = 25.0 Hz, C3‴), 119.7 (C2″), 122.4 (C4′), 127.5 (C1‴), 128.0 (C4″), 129.7 (C3″), 130.8 (*J* = 12.5 Hz, C2‴), 131.8 (C5′), 138.9 (C1″), 153.4 (C3′), 164.4 (*J* = 237.5 Hz, C4‴), 184.6 (CHO). HRMS (EI): *m/z* [M]+ calcd for C_16_H_11N2_OF: 266.0855; found: 266.0855.

#### 3.3.3. 3-(4-Chlorophenyl)-1-Phenyl-1*H*-Pyrazole-4-Carbaldehyde **4c**

Yellow solid (89%). *Rf* = 0.48 (hexane/EtOAc, 7:3), m.p. 138–140 °C. FT-IR (ATR) *ν*_max_ 3124, 1668, 1519, 1505, 1225, 1092, 1012, 957, 836, 813, 751, 727, 684 cm^−1^. NMR (600 MHz, CDCl_3_): *δ* 7.40 (t, *J* = 7.7 Hz, 1H, H-4″), 7.47 (d, *J* = 8.7 Hz, 2H, H-3‴), 7.52 (t, *J* = 7.7 Hz, 2H. H-3″), 7.78 (d, *J* = 7.7 Hz, 2H, H-2″), 7.83 (d, *J* = 8.7 Hz, 2H, H-2‴), 8.53 (s, 1H, H-5), 10.03 (s, 1H, CHO). NMR (150 MHz, CDCl_3_): *δ* 119.7 (C2″), 122.5 (C4′), 128.1 (C4″), 128.9 (C3‴), 129.7 (C3″), 129.8 (C1‴), 130.2 (C2‴), 132.0 (C5′), 135.4 (C4‴), 138.9 (C1″), 153.2 (C3′), 184.4 (CHO). HRMS (EI): *m/z* [M]+ calcd for C_16_H_11N2_OCl: 282.0560; found: 282.0560.

#### 3.3.4. 3-(4-Bromophenyl)-1-Phenyl-1*H*-Pyrazole-4-Carbaldehyde **4d**

Yellow solid (74%). *Rf* = 0.44 (hexane/EtOAc, 7:3), m.p. 140–141 °C. FT-IR (ATR) *ν*_max_ 3126, 1672, 1522, 1503, 1225, 1074, 1008, 813, 758, 684 cm^−1^. NMR (600 MHz, CDCl_3_): *δ* 7.40 (t, *J* = 7.8 Hz, 1H, H4″), 7.51 (t, *J* = 7.8 Hz, 2H, H3″), 7.62 (d, *J* = 8.4 Hz, 2H, H3‴), 7.74–7.80 (m, 4H, H2″, H2‴), 8.52 (s, 1H, H5′), 10.03 (s, 1H, CHO). NMR (150 MHz, CDCl_3_): *δ* 119.9 (C2″), 122.7 (C4′), 123.8 (C4‴), 128.2 (C4″), 129.8 (C3″), 130.5 (C1‴), 130.6 (C2‴), 132.0 (C3‴), 132.2 (C5′), 139.0 (C1″), 153.3 (C3′), 184.5 (CHO). HRMS (EI): *m/z* [M]+ calcd for C_16_H_11_N_2_OBr: 326.0055; found: 326.0050.

#### 3.3.5. 3-(4-Iodophenyl)-1-Phenyl-1*H*-Pyrazole-4-Carbaldehyde **4e**

Light brown solid (73%). *Rf* = 0.55 (hexane/EtOAc, 7:3), m.p. 161–162 °C. FT-IR (ATR) *ν*_max_ 3114, 1669, 1597, 1519, 1216, 1056, 1003, 975, 812, 757, 684 cm^−1^. NMR (600 MHz, CDCl_3_): *δ* 7.41 (t, *J* = 8.1 Hz, 1H, H4″), 7.52 (t, *J* = 8.1 Hz, 2H, H3″), 7.63 (d, *J* = 8.4 Hz, 2H, H2‴), 7.78 (d, *J* = 8.1 Hz, 2H, H2″), 7.84 (d, *J* = 8.4 Hz, 2H, H3‴), 8.53 (s, 1H, H5′), 10.04 (s, 1H, CHO). NMR (150 MHz, CDCl_3_): *δ* 95.5 (C4‴), 119.8 (C2″), 122.5 (C4′), 128.1 (C4″), 129.7 (C3″), 130.6 (C2‴), 130.9 (C1‴), 132.0 (C5′), 137.9 (C3‴), 138.9 (C1″), 153.3 (C3′), 184.4 (CHO). HRMS (EI): *m/z* [M]+ calcd for C_16_H_11_N_2_OI: 373.9916; found: 373.9907.

#### 3.3.6. 4-(4-Formyl-1-Phenyl-1*H*-Pyrazol-3-yl)Benzonitrile **4f**

Light brown solid (80%). *Rf* = 0.66 (hexane/EtOAc, 1:1), m.p. 157–159 °C. FT-IR (ATR) *ν*_max_ 3125, 2831, 2224, 1683, 1519, 1505, 1455, 1399, 1210, 1055, 841, 752, 684 cm^−1^. NMR (600 MHz, CDCl_3_): *δ* 7.43 (t, *J* = 7.7 Hz, 1H, H4″), 7.54 (t, *J* = 7.7 Hz, 2H, H3″), 7.76 (d, *J* = 8.5 Hz, 2H, H3‴), 7.78 (d, *J* = 7.7 Hz, 2H, H2″), 8.08 (d, *J* = 8.5 Hz, 2H, H2‴), 8.56 (s, 1H, H5′), 10.05 (s, 1H, CHO). NMR (150 MHz, CDCl_3_): *δ* 112.7 (C4‴), 118.6 (C1‴), 119.7 (C2″), 122.7 (C4′), 128.3 (C4″), 129.4 (C3‴), 129.8 (C3″), 132.3 (C2‴), 133.4 (C5′), 135.8 (CN), 138.6 (C1″), 151.7 (C3′), 183.6 (CHO). HRMS (EI): *m/z* [M]+ calcd for C_17_H_11_N_3_O: 273.0902; found: 273.0904.

#### 3.3.7. 3-(4-Nitrophenyl)-1-Phenyl-1*H*-Pyrazole-4-Carbaldehyde **4g**

Light brown solid (90%). *Rf* = 0.34 (hexane/EtOAc, 7:3), m.p. 163–165 °C (Lit. 163–164 °C [64]). FT-IR (ATR) *ν*_max_ 3127, 1677, 1597, 1525, 1506, 1339, 1206, 1070, 854, 757, 709, 685 cm^−1^. NMR (600 MHz, CDCl_3_): δ 7.44 (t, *J* = 7.6 Hz, 1H, H4″), 7.55 (t, *J* = 7.6 Hz, 2H, H3″), 7.81 (d, *J* = 7.6 Hz, 2H, H2″), 8.17 (d, *J* = 8.7 Hz, 2H, H2‴), 8.35 (d, *J* = 8.7 Hz, 2H, H3‴), 8.58 (s, 1H, H5′), 10.09 (s, 1H, CHO). NMR (150 MHz, CDCl3): *δ* 119.7 (C2″), 122.9 (C4′), 123.8 (C3‴), 128.4 (C4″), 129.7 (C2‴), 129.8 (C3″), 133.6 (C5′), 137.7 (C1‴), 138.7 (C1″), 148.1 (C4‴), 151.3 (C3′), 183.6 (CHO). HRMS (EI): *m/z* [M]+ calcd for C_16_H_11_N_3_O_3_: 293.0800; found: 293.0798.

#### 3.3.8. 1-Phenyl-3-(p-Tolyl)-1*H*-Pyrazole-4-Carbaldehyde **4h**

Light brown solid (67%). *Rf* = 0.55 (hexane/EtOAc, 7:3), m.p. 115–117 °C. FT-IR (ATR) *ν*_max_ 3122, 2834, 2782, 1737, 1668, 1599, 1517, 1218 cm^−1^. NMR (500 MHz, CDCl_3_): δ 2.44 (s, 3H, Me), 7.32 (d, *J* = 7.2 Hz, 2H, H3‴), 7.39 (t, *J* = 7.5 Hz, 1H, H4″), 7.51 (t, *J* = 7.5 Hz, 2H, H3″), 7.72 (d, *J* = 7.2 Hz, 2H, H2‴), 7.80 (d, *J* = 7.5 Hz, 2H, H2″), 8.54 (s, 1H, H5′), 10.05 (s, 1H, CHO). NMR (125 MHz, CDCl_3_): δ 21.3 (Me), 119.8 (C2″), 122.5 (C4′), 127.9 (C4″), 128.5 (C1‴), 128.8 (C2‴), 129.4 (C3‴), 129.7 (C3″), 130.8 (C5′), 139.1 (C1″), 139.3 (C4‴), 154.9 (C3′), 185.3 (CHO). HRMS (EI): *m/z* [M]+ calcd for C_17_H_14_N_2_O: 262.1106; found: 262.1109.

#### 3.3.9. 3-(4-Ethylphenyl)-1-Phenyl-1*H*-Pyrazole-4-Carbaldehyde **4i**

Light brown solid (55%). Rf = 0.55 (hexane/EtOAc, 7:3), m.p. 116–117 °C (Lit. 120–122 °C [64]). FT-IR (ATR) ν_max_ 3123, 2964, 2823, 1738, 1670, 1598, 1506, 1216, 754 cm^−1^. NMR (500 MHz, CDCl_3_): δ 1.29 (t, J = 7.7 Hz, 3H, CH_2_CH_3_), 2.73 (c, J = 7.7 Hz, 2H, CH_2_CH_3_), 7.34 (d, J = 8.5 Hz, 2H, H3‴), 7.38 (t, J = 7.4 Hz, 1H, H4″), 7.51 (tm, J = 7.7 Hz, 2H, H3″), 7.74 (dm, J = 8.5 Hz, 2H, H2‴), 7.79 (dm, J = 7.7 Hz, 2H, H2″), 8.54 (s, 1H, H5′), 10.06 (s, 1H, CHO). NMR (125 MHz, CDCl_3_): δ 15.5 (CH_2_CH_3_), 28.7 (CH_2_CH_3_), 119.7 (C2″), 122.4 (C4′), 127.8 (C4″), 128.3 (C3‴), 128.6 (C1‴), 128.9 (C2‴), 129.6 (C3″), 130.8 (C5′), 139.0 (C1″), 145.6 (C4‴), 154.9 (C3′), 185.3 (CHO). HRMS (EI): m/z [M]+ calcd for C_18_H_16_N_2_O: 276.1263; found: 276.1261.

#### 3.3.10. 3-(4-Methoxyphenyl)-1-Phenyl-1*H*-Pyrazole-4-Carbaldehyde **4j**

Brown solid (68%). *Rf* = 0.42 (hexane/EtOAc, 7:3), m.p. 132–134 °C (Lit. 100–102 °C [64]). FT-IR (ATR) *ν*_max_ 3123, 1669, 1600, 1519, 1455, 1258, 1226, 1174, 1042, 956, 838, 784, 752, 685 cm^−1^. NMR (600 MHz, CDCl_3_): δ 3.88 (s, 3H, OMe), 7.03 (d, *J* = 8.6 Hz, 2H, H3‴), 7.39 (t, *J* = 7.5 Hz, 1H, H4″), 7.51 (t, *J* = 7.8 hz, 2H, H3″), 7.77–7.82 (m, 4H, H2″, H2‴), 8.52 (s, 1H, H5′), 10.04 (s, 1H, CHO). NMR (150 MHz, CDCl_3_): δ 55.3 (OMe), 114.2 (C3‴), 119.7 (C2″), 122.4 (C4′), 123.9 (C1‴), 127.8 (C4″), 129.6 (C3″), 130.3 (C2‴), 131.2 (C5′), 139.1 (C1″), 154.5 (C3′), 160.6 (C4‴), 185.1 (CHO). HRMS (EI): m/z [M]+ calcd for C_17_H_14_N_2_O_2_: 278.1055; found: 278.1054.

### 3.4. General Procedure for the Synthesis of (4S*, 5S*)-5-(1,3-Diphenyl-1H-Pyrazol-4-yl)-4-Tosyl-4,5-Dihydrooxazoles ***6a-j***

The corresponding 1,3-diphenyl-1*H*-pyrazole-4-carbaldehyde (0.806 mmol) was added to a solution of TosMIC (0.969 mmol) and KOH (0.969 mmol) under nitrogen atmosphere at rt. The mixture was stirred at 25 °C for 3 h. The residue was filtered under vacuum and washed with isopropanol-water (1:1). Finally, it was recrystallized with acetone/ethyl acetate (1:1) to form the title compound **6**.

#### 3.4.1. (4S*, 5S*)-5-(1,3-Diphenyl-1*H*-Pyrazol-4-yl)-4-Tosyl-4,5-Dihydrooxazole **6a**

White solid (81%). *Rf* = 0.48 (hexane/EtOAc, 1:1), m.p. 184–185 °C. FT-IR (ATR) *ν*_max_ 3090, 1610, 1502, 1453, 1315, 1294, 1149, 1121, 902, 753, 658 cm^−1^. NMR ^1^H (600 MHz, DMSO-*d_6_*): δ 2.41 (s, 3H, H5⁗), 5.81 (d, *J* = 6.5 Hz, 1H, H5), 5.93 (dd, *J* = 6.5, 1.7 Hz, 1H, H4), 7.37 (t, *J* = 7.5 Hz, 1H, H4″), 7.44–7.60 (m, 7H, H3″, H3‴, H4‴, H3⁗), 7.63 (d, *J* = 1.7 Hz, 1H, H2), 7.70–7.75 (m, 2H, H2‴), 7.77 (d, *J* = 8.0 Hz, 2H, H2⁗), 7.88 (d, *J* = 8.0 Hz, 2H, H2″), 8.87 (s, 1H, H5′). NMR ^13^C (150 MHz, DMSO-*d_6_*): δ 21.2 (C5⁗), 71.7 (C5), 89.3 (C4), 117.3 (C4′), 118.6 (C2″), 127.0 (C4″), 128.0 (C2‴), 128.8 (C4‴), 129.0 (C2⁗), 129.2 (C5′), 129.5 (C3‴), 129.7 (C3″), 129.9 (C3⁗), 131.7 (C1‴), 132.9 (C1⁗), 139.1 (C1″), 145.5 (C4⁗), 151.5 (C3′), 159.4 (C2). HRMS (EI): *m/z* [M]+ calcd for C_25_H_21_N_3_O_3_S: 443.1304; found: 443.1306.

#### 3.4.2. (4S*, 5S*)-5-(3-(4-Fluorophenyl)-1-Phenyl-1*H*-Pyrazol-4-yl)-4-Tosyl-4,5-Dihydrooxazole **6b**

White solid (86%). *Rf* = 0.48 (hexane/EtOAc, 1:1), m.p. 228–230 °C. FT-IR (ATR) *ν*_max_ 1610, 1502, 1303, 1292, 1219, 1145, 1119, 839, 762, 669 cm^−1^. NMR ^1^H (600 MHz, DMSO-*d_6_*): δ 2.41 (s, 3H, H5⁗), 5.79 (d, *J* = 6.5 Hz, 1H, H5), 5.92 (dd, *J* = 6.5, 1.7 Hz, 1H, H4), 7.37 (t, *J* = 8.0 Hz, 1H, H4″), 7.41 (tm, *J* = 8.0 Hz, 2H, H3‴), 7.47 (d, *J* = 8.0 Hz, 2H, H3⁗), 7.52 (t, *J* = 8.0 Hz, 2H, H3″), 7.61 (d, *J* = 1.7 Hz, 1H, H2), 7.71-7.79 (m, 4H, H2‴, H2⁗), 7.86 (d, *J* = 8.0 Hz, 2H, H2″), 8.86 (s, 1H, H5′). NMR ^13^C (150 MHz, DMSO-*d_6_*): δ 21.2 (C5⁗), 71.8 (C5), 89.3 (C4), 116.1 (*J* = 25.0 Hz, C3‴), 117.3 (C4′), 118.6 (C2″), 127.1 (C4″), 128.2 (C1‴), 129.3 (C2⁗), 129.5 (C5′), 129.7 (C3″), 129.9 (C3⁗), 130.1 (*J* = 12.5 Hz, C2‴), 132.9 (C1⁗), 139.1 (C1″), 145.6 (C4⁗), 150.5 (C3′), 159.5 (C2), 163.5 (J = 250.0 Hz, C4‴). HRMS (EI): *m/z* [M]+ calcd for C_25_H_20_N_3_O_3_FS: 461.1209; found: 461.1209.

#### 3.4.3. (4S*, 5S*)-5-(3-(4-Chlorophenyl)-1-Phenyl-1*H*-Pyrazol-4-yl)-4-Tosyl-4,5-Dihydrooxazole **6c**

White solid (85%). *Rf* = 0.48 (hexane/EtOAc, 1:1), m.p. 200–203 °C. FT-IR (ATR) *ν*_max_ 2921, 1610, 1504, 1454, 1302, 1292, 1144, 1121, 811, 757, 688 cm^−1^. NMR ^1^H (600 MHz, DMSO-*d_6_*): δ 2.41 (s, 3H, H5⁗), 5.82 (d, *J* = 6.3 Hz, 1H, H5), 5.94 (dd, *J* = 6.3, 1.7 Hz, 1H, H4), 7.37 (t, *J* = 7.5 Hz, 1H, H4″), 7.47 (d, *J* = 8.5 Hz, 2H, H3⁗), 7.54 (t, *J* = 7.5 Hz, 2H, H3″), 7.62–7.66 (m, 2H, H3‴), 7.64 (s, 1H, H2), 7.75 (d, *J* = 8.5 Hz, 2H, H2‴), 7.78 (d, *J* = 8.5 Hz, 2H, H2⁗), 7.87 (d, *J* = 7.5 Hz, 2H, H-2″), 8.89 (s, 1H, H5′). NMR ^13^C (150 MHz, DMSO-*d_6_*): δ 21.2 (C5⁗), 71.7 (C5), 89.2 (C4), 117.4 (C4′), 118.6 (C2″), 127.1 (C4″), 129.1 (C3‴), 129.2 (C2⁗), 129.6 (C5′), 129.7 (C2‴), 129.8 (C3″), 129.9 (C3⁗), 130.6 (C1‴), 132.8 (C1⁗), 133.6 (C4‴), 139.0 (C1″), 145.5 (C4⁗), 150.2 (C3′), 159.4 (C2). HRMS (EI): *m/z* [M]+ calcd for C_25_H_20_N_3_O_3_ClS: 477.0914; found: 447.0912.

#### 3.4.4. (4S*, 5S*)-5-(3-(4-Bromophenyl)-1-Phenyl-1*H*-Pyrazol-4-yl)-4-Tosyl-4,5-Dihydrooxazole **6d**

White solid (81%). *Rf* = 0.48 (hexane/EtOAc, 1:1), m.p. 224–226 °C. FT-IR (ATR) *ν*_max_ 3084, 2922, 1608, 1502, 1317, 1245, 1148, 1122, 1084, 907, 813, 753, 658 cm^−1^. NMR ^1^H (600 MHz, DMSO-*d_6_*): *δ* 2.41 (s, 3H, H5⁗), 5.80 (d, *J* = 6.5 Hz, 1H, H5), 5.93 (dd, *J* = 6.5, 1.7 Hz, 1H, H4), 7.37 (t, *J* = 7.5 Hz, 1H, H4″), 7.47 (d, *J* = 7.5 Hz, 2H, H3⁗), 7.54 (t, *J* = 7.5 Hz, 2H, H3″), 7.61 (d, *J* = 1.5 Hz, 1H, H2), 7.67 (d, *J* = 8.5 Hz, 2H, H2‴), 7.75–7.80 (m, 4H, H2⁗, H3‴), 7.86 (d, *J* = 8.0 Hz, 2H, H2″), 8.87 (s, 1H, H5′). NMR ^13^C (150 MHz, DMSO-*d_6_*): δ 21.2 (C5⁗), 71.7 (C5), 89.3 (C4), 117.4 (C4′), 118.7 (C2″), 122.3 (C4‴), 127.2 (C4″), 129.3 (C2⁗), 129.7 (C5′), 129.8 (C3″), 129.9 (C3⁗), 130.0 (C2‴), 131.0 (C1‴), 132.1 (C3‴), 132.9 (C1⁗), 139.1 (C1″), 145.6 (C4⁗), 150.3 (C3′), 159.5 (C2). HRMS (EI): *m/z* [M]+ calcd for C_25_H_20_N_3_O_3_BrS: 521.0409; found: 521.0397.

#### 3.4.5. (4S*, 5S*)-5-(3-(4-Iodophenyl)-1-Phenyl-1*H*-Pyrazol-4-yl)-4-Tosyl-4,5-Dihydrooxazole **6e**

White solid (35%). *Rf* = 0.48 (hexane/EtOAc, 1:1), m.p. 209–211 °C. FT-IR (ATR) *ν*_max_ 1610, 1504, 1316, 1146, 1124, 1081, 1064, 812, 752 cm^−1^. NMR ^1^H (500 MHz, DMSO-*d_6_*): *δ* 2.41 (s, 3H, H5⁗), 5.80 (d, J = 6.5 Hz, 1H, H5), 5.92 (da, J = 6.5 Hz, 1H, H4), 7.37 (t, J = 7.5 Hz, 1H, H4″), 7.47 (d, J = 8.0 Hz, 2H, H3⁗), 7.50–7.56 (m, 4H, H3″, H2‴), 7.62 (s, 1H, H2), 7.77 (d, J = 8.0 Hz, 2H, H2⁗), 7.86 (d, J = 8.0 Hz, 2H, H2″), 7.93 (d, J = 8.0 Hz, 2H, H3‴), 8.87 (s, 1H, H5′). NMR ^13^C (125 MHz, DMSO-*d_6_*): δ 21.2 (C5⁗), 71.7 (C5), 89.2 (C4), 95.5 (C4‴), 117.3 (C4′), 118.6 (C2″), 127.1 (C4″), 129.2 (C2⁗), 129.6 (C5′), 129.7 (C2‴), 129.8 (C3″), 129.9 (C3⁗), 131.2 (C1‴), 132.8 (C1⁗), 137.8 (C3‴), 139.0 (C1″), 145.5 (C4⁗), 150.4 (C3′), 159.4 (C2). HRMS (EI): *m/z* [M]+ calcd for C_25_H_20_N_3_O_3_IS: 569.0270; found: 569.0265.

#### 3.4.6. (4S*, 5S*)-4-(1-Phenyl-4-(4-Tosyl-4,5-Dihydrooxazol-5-yl)-1*H*-Pyrazol-3-yl)Benzonitrile **6f**

White solid (69%). *Rf* = 0.51 (hexane/EtOAc, 1:1), m.p. 191–193 °C. FT-IR (ATR) *ν*_max_ 2.47 (s, 3H, H5⁗), 6.03 (dd, *J* = 6.5, 2.0 Hz, 1H, H4), 6.07 (d, *J* = 6.5 Hz, 1H, H5), 7.43 (t, *J* = 7.5 Hz, 1H, H4″), 7.54 (d, *J* = 8.0 Hz, 2H, H3⁗), 7.60 (t, *J* = 7.5 Hz, 2H, H3″), 7.72 (d, *J* = 2.0 Hz, 1H, H2), 7.88 (d, *J* = 8.0 Hz, 2H, H2⁗), 8.01–8.04 (m, 2H, H2″), 8.10 (m, 4H, H2‴, H3‴), 9.06 (s, 1H, H5′). NMR ^13^C (125 MHz, DMSO-*d_6_*): δ 21.4 (C5‘‘‘‘), 72.7 (C5), 90.8 (C4), 112.4 (C4‘‘‘‘), 119.2 (C4‘), 119.4 (CN), 119.4 (C2″), 127.9 (C4″), 129.6 (C2‴), 130.1 (C2⁗), 130.40 (C5′), 130.45 (C3″), 130.6 (C3⁗), 133.6 (C3‴), 134.3 (C1⁗), 137.4 (C1‴), 140.1 (C1″), 146.4 (C4⁗), 150.5 (C3′), 160.0 (C2). HRMS (EI): *m/z* [M+] calcd para C_26_H_20_N_4_O_3_S: 468.1256; found: 468.1245.

#### 3.4.7. (4S*, 5S*)-5-(3-(4-Nitrophenyl)-1-Phenyl-1*H*-Pyrazol-4-yl)-4-Tosyl-4,5-Dihydrooxazole **6g**

White solid (63%). *Rf* = 0.52 (hexane/EtOAc, 1:1), m.p. 235–236 °C. FT-IR (ATR) *ν*_max_ 3174, 1618, 1598, 1547, 1504, 1303, 1245, 1150, 1118, 1076, 909, 845, 752, 724, 681 cm^−1^. NMR ^13^C (125 MHz, DMSO-*d_6_*): δ 2.40 (s, 3H, H5⁗), 5.91 (d, *J* = 6.5 Hz, 1H, H5), 5.96 (dd, *J* = 6.5, 1.7 Hz, 1H, H4), 7.40 (t, *J* = 7.5 Hz, 1H, H4″), 7.46 (d, *J* = 8.0 Hz, 2H, H3⁗), 7.56 (t, *J* = 7.5 Hz, 2H, H3″), 7.64 (d, *J* = 1.5 Hz, 1H, H2), 7.78 (d, *J* = 8.0 Hz, 2H, H2⁗), 7.89 (d, *J* = 7.5 Hz, 2H, H-2″), 8.02 (d, *J* = 8.9 Hz, 2H, H2‴), 8.41 (d, *J* = 8.9 Hz, 2H, H3‴), 8.93 (s, 1H, H5′). NMR ^13^C (125 MHz, DMSO-*d_6_*): δ 21.2 (C5⁗), 71.7 (C5), 89.3 (C4), 118.1 (C4′), 118.8 (C2″), 124.3 (C3‴), 127.5 (C4″), 129.0 (C2‴), 129.3 (C2⁗), 129.8 (C3″), 129.9 (C3⁗), 130.0 (C5′), 132.8 (C1⁗), 138.2 (C1‴), 138.9 (C1″), 145.6 (C4⁗), 147.5 (C4‴), 149.1 (C3′), 159.5 (C2). HRMS (EI): *m/z* [M+] calcd para C_25_H_20_N_4_O_5_S: 488.1154; found: 488.1157.

#### 3.4.8. (4S*, 5S*)-5-(1-Phenyl-3-(p-Tolyl)-1*H*-Pyrazol-4-yl)-4-Tosyl-4,5-Dihydrooxazole **6h**

White solid (71%). *Rf* = 0.55 (hexane/EtOAc, 1:1), m.p. 177–178 °C. FT-IR (ATR) *ν*_max_ 2920, 1612, 1547, 1504, 1316, 1296, 1149, 1117, 903, 814, 754, 659 cm^−1^. NMR ^13^C (125 MHz, DMSO-*d_6_*): δ 2.40 (s, 3H, Me), 2.42 (s, 3H, H5⁗), 5.81 (d, *J* = 6.5 Hz, 1H, H5), 5.89 (dd, *J* = 6.5, 1.7 Hz, 1H, H4), 7.31–7.38 (m, 3H, H4″, H3‴), 7.45 (d, *J* = 8.0 Hz, 2H, H3⁗), 7.51 (t, *J* = 7.7 Hz, 2H, H3″), 7.58 (d, *J* = 1.5 Hz, 1H, H2), 7.62 (d, *J* = 7.5 Hz, 2H, H2‴), 7.77 (d, *J* = 8.0 Hz, 2H, H2⁗), 7.86 (d, *J* = 7.7 Hz, 2H, H2″), 8.83 (s, 1H, H5′). NMR ^13^C (125 MHz, DMSO-*d_6_*): δ 20.9 (Me-4‴), 21.1 (C5⁗), 71.8 (C5), 89.3 (C4), 117.2 (C4′), 118.4 (C2″), 126.7 (C4″), 127.8 (C2‴), 128.9 (C5′), 129.2 (C2⁗), 129.3 (C1‴), 129.4 (C3″), 129.5 (C3‴), 129.7 (C3⁗), 132.9 (C1⁗), 138.1 (C4‴), 139.1 (C1″), 145.3 (C4⁗), 151.5 (C3′), 159.3 (C2). HRMS (EI): *m/z* [M+] calcd para C_26_H_23_N_3_O_3_S: 457.1460; found: 457.1465.

#### 3.4.9. (4S*, 5S*)-5-(3-(4-Ethylphenyl)-1-Phenyl-1*H*-Pyrazol-4-yl)-4-Tosyl-4,5-Dihydrooxazole **6i**

White solid (67%). *Rf* = 0.59 (hexane/EtOAc, 1:1), m.p. 176–178 °C. FT-IR (ATR) *ν*_max_ 3130, 2969, 1737, 1618, 1307, 1148, 1114, 758, 658 cm^−1^. NMR ^1^H (500 MHz, DMSO-*d_6_*): δ 1.24 (t, *J* = 7.7 Hz, 3H, CH_3_CH_2_), 2.41 (s, 3H, H5⁗), 2.69 (q, *J* = 7.7 Hz, 2H, CH_3_CH_2_), 5.80 (d, *J* = 6.5 Hz, 1H, H5), 5.92 (dd, *J* = 6.5, 1.7 Hz, 1H, H4), 7.36 (t, *J* = 7.7 Hz, 1H, H4″), 7.40 (d, *J* = 7.8 Hz, 2H, H3‴), 7.47 (d, *J* = 8.0 Hz, 2H, H3⁗), 7.53 (tm, *J* = 7.7 Hz, 2H, H3″), 7.62 (sa, 1H, H2), 7.62–7.64 (m, 2H, H2‴), 7.77 (d, *J* = 8.0 Hz, 2H, H2⁗), 7.86 (d, *J* = 7.7 Hz, 2H, H2″), 8.84 (s, 1H, H5′). NMR ^13^C (125 MHz, DMSO-*d_6_*): δ 15.6 (CH_3_CH_2_), 21.2 (C5⁗), 28.1 (CH_3_CH_2_), 71.8 (C5), 89.3 (C4), 117.3 (C4′), 118.6 (C2″), 127.0 (C4″), 128.0 (C2‴), 128.5 (C3‴), 129.2 (C1‴), 129.3 (C2⁗), 129.4 (C5′), 129.7 (C3″), 129.9 (C3⁗), 132.9 (C1⁗), 139.2 (C1″), 144.6 (C4‴), 145.6 (C4⁗), 151.6 (C3′), 159.5 (C2). HRMS (EI): *m/z* [M+] calcd para C_27_H_25_N_3_O_3_S: 471.1617; found: 471.1614.

#### 3.4.10. (4S*, 5S*)-5-(3-(4-Methoxyphenyl)-1-Phenyl-1*H*-Pyrazol-4-yl)-4-Tosyl-4,5-Dihydrooxazole **6j**

White solid (62%). *Rf* = 0.51 (hexane/EtOAc, 1:1), m.p. 188–190 °C. FT-IR (ATR) *ν*_max_ 1614, 1530, 1505, 1456, 1294, 1258, 1147, 1120, 901, 833, 756 cm^−1^. NMR ^1^H (500 MHz, DMSO-*d_6_*): δ 2.41 (s, 3H, H5⁗), 3.84 (s, 3H, OMe), 5.79 (d, *J* = 6.5 Hz, 1H, H5), 5.91 (dd, *J* = 6.5, 2.0 Hz, 1H, H4), 7.12 (d, *J* = 8.0 Hz, 2H, H3‴), 7.35 (t, *J* = 7.5 Hz, 1H, H4″), 7.47 (d, *J* = 8.0 Hz, 2H, H3⁗), 7.52 (t, *J* = 7.5 Hz, 2H, H3″), 7.62 (d, *J* = 2.0 Hz, 1H, H2), 7.65 (d, *J* = 8.0 Hz, 2H, H2‴), 7.77 (d, *J* = 8.0 Hz, 2H, H2⁗), 7.86 (d, *J* = 7.5 Hz, 2H, H2″), 8.83 (s, 1H, H5′). NMR ^13^C (125 MHz, DMSO-*d_6_*): δ 21.2 (C5⁗), 55.3 (OMe), 71.9 (C5), 89.2 (C4), 114.5 (C4′), 117.1 (C3‴), 118.5 (C4″), 124.1 (C2‴), 126.9 (C3‴), 129.2 (C1‴), 129.3 (C2⁗), 129.4 (C5′), 129.7 (C3″), 129.9 (C3⁗), 132.9 (C1⁗), 139.2 (C1″), 145.6 (C4⁗), 151.4 (C3′), 159.4 (C2), 159.7. HRMS (EI): *m/z* [M+] calcd para C_26_H_23_N_3_O_4_S: 473.1409; found: 473.1413.

### 3.5. In Silico Analysis of 1,3-Diphenyl-1H-Pyrazole-4-Carbaldehydes 4a-j and (4S*, 5S*)-5-(1,3-Diphenyl-1H-Pyrazol-4-yl)-4-Tosyl-4,5-Dihydrooxazoles ***6a-j***

The toxicological and physicochemical properties of 1,3-diphenyl-1*H*-pyrazole-4-carbaldehydes **4a-j** and (4S*, 5S*)-5-(1,3-diphenyl-1*H*-pyrazol-4-yl)-4-tosyl-4,5-dihydrooxazoles **6a-j** were evaluated on the OSIRIS DataWarrior V.4.7.2 program (http://www.organicchemistry.org/prog/peo/ accessed on 2 February 2024) [39]. The drug-like and pharmacokinetic properties were assessed with the SwissADME server platform [40]. The physicochemical properties were analyzed based on Lipinski’s rules, considering Log P value, molecular weight, hydrogen bond donors, and hydrogen bond acceptors [65].

### 3.6. Multiple Sequence Alignment and Generation of a 3D Model of the CYP51 of Candida spp. through Homology Modeling

The sequences of the lanosterol 14α-demethylase enzymes (CYP51) were downloaded from the NCBI database (http://www.ncbi.nlm.nih.gov accessed on 2 February 2024) [66] for *C. albicans* AATCC 10,231 (CYP51Ca), *C. auris* (CYP51Cau), *C. dubliniensis* CD36 (CYP51Cdu), *C. glabrata* CBS138 (CYP51Cg), *C. haemulonii* (CYP51Cha), and *C. krusei* ATCC 6358 (CYP51Ckr). The percentage of identity of each of the CYP51 sequences of *Candida* spp. with the CYP51 protein of *C. albicans* (CYP51Ca) complexed with posaconazole at the active site (PDB code: 5FSA) was determined with the BLASTp (protein query-protein database) server. Three-dimensional models were elaborated with the sequences of CYP51 proteins of *Candida* spp. by using the homology modeling technique on the Modeller 10.4 program [67]. The crystallized structure of the CYP51 enzyme from *C. albicans* (PDB code: 5FSA) served as a template for the construction of the models. Once the amino acid sequences were aligned to build 15 3D models for each of the different *Candida* species, the model with the lowest energy was selected. The quality of the model chosen for each *Candida* species was validated on the PROCHECK [49] online server. The 3D models of CYP51 were validated with the VERIFY3D [68] and PROCHECK [49] programs in order to check the stereochemical quality of the Ramachandran plots that show the amino acid residues in the allowed regions. The analysis of the structural alignment of 3D models of CYP51Cau, CYP51Cdu, CYP51Cg, CYP51Cha and CYP51Ckr was carried out using Pymol Version 3.0 for windows https://pymol.org/ accessed on 22 February 2024. The six selected CYP51 models of *Candida* spp. were overlapped on the Discovery Studio Visualizer [69].

### 3.7. Molecular Docking of the Compounds on the CYP51 Enzymes of Candida spp.

On the AutoDock4 program, the docking of 5-(1,3-diphenyl-1*H*-pyrazol-4-yl)-4-tosyl-4,5-dihydrooxazoles **6a-j** and 1,3-diphenyl-1*H*-pyrazole-4-carbaldehydes **4a-j** was carried out at the active site of the CYP51 enzymes of *C. albicans*, *C. auris*, *C. dubliniensis*, *C glabrata*, *C. haemulonii*, and *C. krusei* [70]. The CYP51 enzymes of *C. auris*, *C. dubliniensis*, *C. glabrata*, *C. haemulonii*, and *C. krusei* were modeled by using the crystallized CYP51 protein of *C. albicans* (PDB code: 5FSA) as a template. The proteins were processed by adding hydrogen atoms to the polar atoms (considering a pH of 7.4) and assigning the Kollman charges. The water molecules were removed and the proteins were optimized on the Nanoscale Molecular Dynamics (NAMD) program [71]. The 3D structure of fluconazole **20** was downloaded from the ZINC 15 database [72]. The 5-(1,3-diphenyl-1*H*-pyrazol-4-yl)-4-tosyl-4,5-dihydrooxazoles **6a-j** and 1,3-diphenyl-1*H*-pyrazole-4-carbaldehydes **4a-j** were sketched in two dimensions with ChemSketch (https://www.acdlabs.com/resources/freeware/chemsketch/ accessed on 12 January 2024) and converted into 3D mol2 format on the Open Babel GUI program [73]. Fluconazole and the test ligands were optimized with PM6 on Gaussian 98 software to obtain the minimum energy conformation for the docking studies [74]. Molecular docking simulations were carried out on AutoDock version 4.2 [70] with the following grid dimensions: 62 × 54 × 62 Å^3^ for *C. albicans* and *C. dubliniensis*; 62 × 56 × 62 Å^3^ for *C. auris*, *C. glabrata*, and *C. haemulonii*; and 72 × 52 × 62 Å^3^ for *C. krusei.* The grid center values found for each of the CYP51 enzymes of the *Candida* spp. were as follows: *C. albicans* (X = 195.4, Y = −3.3, and Z = 33.3), *C. auris* (X = 99.0, Y = −4.5, and Z = 37.0), *C. dubliniensis* (X = 195.4, Y = −3.4, and Z = 33.3*), C. glabrata* (X = 195.4, Y = −3.3, and Z = 33.5), *C. haemulonii* (X = 195.4, Y = −3.4, and Z = 33.3), and *C. krusei* (X = 193.4, Y = −3.9, and Z = 33.40). The hybrid Lamarckian genetic algorithm was applied for minimization, utilizing default parameters. Out of the 100 docking runs performed, the conformation with the lowest binding energy (kcal/mol) was selected for all subsequent simulations. AutoDockTools was used to prepare the script and files as well as to visualize the docking results, which were edited on the Discovery Studio Visualizer [69].

GOLD program version 5.6.3 https://www.ccdc.cam.ac.uk/solutions/software/gold/ accessed on 29 March 2024, was used to prepare the receptor for docking. For this study, the protein binding site was identified within (15 Å) of the reference ligand. The number of produced poses was set to 50 and the number of predetermined postures was fixed. We employed the configuration model of the ChemScore kinase scoring function. As a scoring function, ChemScore fitness DG is employed, which represents the total free energy change that occurs on ligand binding. The findings were stored as .mol2 files.

### 3.8. In Vitro Experiments

#### 3.8.1. Strains for the Antifungal Susceptibility Tests

The strains for the antifungal susceptibility tests were *C. albicans* ATCC 10231, *C. glabrata* CBS138, *C. dubliniensis* CD36, *C. krusei* ATCC 14423, *C. auris* Monterrey, and *C. haemulonii* ENCB87. They were stored at –70 °C in 50% (*vol/vol*) of glycerol and recovered in yeast extract-peptone-dextrose (YPD) medium (1% yeast extract, 2% casein peptone, and 2% dextrose) under orbital shaking at 120 rpm and 37 °C, to serve as the inoculum in the assays.

#### 3.8.2. Antifungal Activity of the Pyrazole and Dihydrooxazole Derivatives on *Candida* spp.

The antifungal activity of the pyrazole derivatives **4a-j** against *Candida* spp. was evaluated with the CLSI M27-A3 microdilution method [75]. Fluconazole **20** (the antifungal reference drug) and the pyrazole **4a-j** and dihydrooxazole derivatives **6a-j** were examined at concentrations of 6.4 to 0.0125 μg/mL. The diluent was RPMI 1640 medium (Sigma-Aldrich, St. Louis, MO, USA) for **20** and DMSO for the two series of test compounds. To avoid an inhibitory effect by DMSO, it was employed at less than 10% of the total volume. For the preparation of the inoculum of *Candida* spp., the optical density was adjusted on a spectrophotometer to 620 nm, followed by a 1:1000 dilution with RPMI medium. The 96-well microplates were inoculated with 100 µL of yeast suspension. RPMI served as the sterility control and DMSO without any antifungal compound as the growth control. The microplates were incubated at 37 °C for 24 h, and upon completion of this, the time growth was quantified by optical density on a Multiskan™ GO microplate spectrophotometer at 620 nm. The reported values of yeast growth are expressed as the averages of three independent assays.

#### 3.8.3. Rescue of the Growth of *Candida* spp. by Adding Ergosterol

To verify that pyrazole **4a-j** and dihydrooxazole derivatives **6a-j** affect the viability of *Candida* spp. by inhibiting ergosterol synthesis, a growth rescue experiment was performed. A total 100 µL of one of the solutions of the compounds prepared in RPMI 1640 medium (Sigma-Aldrich) was added to each well of a 96-well microplate, followed by the addition of 80 µL of a yeast suspension adjusted to 1–5 × 10^6^ CFU/mL and diluted 1:1000 with RPMI 1640 medium (Sigma-Aldrich). Subsequently, of a stock ergosterol solution was added, which was prepared by dissolving 120 µg/mL in Tween 80/ethanol (1:1). The final ergosterol concentration in each well was 12 µg/mL. The controls used were yeast cells grown in the absence of an inhibitor and those grown in the presence of the inhibitor only (without adding ergosterol) [60,61].

## 4. Conclusions

In the current contribution, the synthesis of new 5-(1,3-diphenyl-1*H*-pyrazol-4-yl)-4-tosyl-4,5-dihydrooxazoles **6a-j** was described. The synthetic design focused on the Van Leusen reaction between a series of 1,3-diaryl-1*H*-pyrazole-4-carbaldehydes **4a-j** and TosMIC **5** in the presence of a base without heating, which resulted in short reaction times, high compound purity, and high yields. The molecular docking study revealed that compounds **4a-j** and **6a-j** have better binding energy values than fluconazole (the reference compound). The in vitro testing showed better antifungal activity for **4a-j** and **6a-j** versus fluconazole, which is evidenced by lower MIC_70_ and MIC_90_ values for the test compounds. Thus, the in silico and in vitro data correlated well. Growth rescue assays demonstrated that **4a-j** and **6a-j** interfere with ergosterol synthesis in the six *Candida* species analyzed, similar to what has been previously documented in the literature for fluconazole. This is the first report, to our knowledge, on the synthesis, in vitro antifungal activity, and in silico study of the binding affinity of the series of dihydrooxazoles **6a-j** in relation to *Candida* species. Additionally, it is one of the few reports dealing with the effect of dihydrooxazoles on a wide spectrum of *Candida* spp., despite the importance of such a study given the multi-drug resistance that has developed in many such species. The current findings suggest the merit of continuing to design new inhibitors of the lanosterol 14-⍺ demethylase enzyme, taking **6a-j** as lead compounds in order to propose a more effective antifungal therapy against multi-drug-resistant species of the *Candida* genus.

## Figures and Tables

**Table 1:** Reaction conditions and yields for the synthesis of (*E*)-1-phenyl-2-(1-phenylethylidene)hydrazones **3a-j** ^a^.

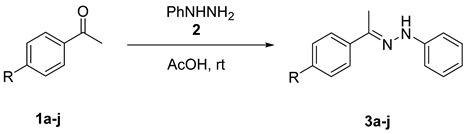					
Entry	Ketone	R	T (°C)	t (min)	3a-j (%)
1	**1a**	H	rt	10	86
2	**1b**	F	rt	15	92
3	**1c**	Cl	rt	8	89
4	**1d**	Br	rt	20	94
5	**1e**	I	rt	40	92
6	**1f**	CN	rt	5	60
7	**1g**	NO_2_	rt	5	85
8	**1h**	Me	rt	30	97
9	**1i**	Et	rt	15	85
10	**1j**	OMe	rt	30	95

^a^ Reaction conditions: **1a**-**j** and phenylhydrazine **2** were reacted (1:1 equiv. mol) in 8 mL of glacial acetic acid at room temperature.

**Table 2:** Reaction conditions and yields for the synthesis of 1,3-diphenyl-1*H*-pyrazole-4-carbaldehydes **4a-j** ^a^.

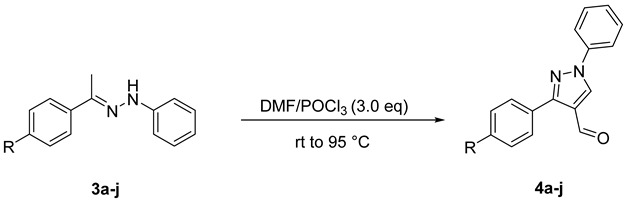					
Entry	Hydrazone	R	T (°C)	t (h)	4a-j (%) ^b^
1	**3a**	H	25–95	12	60
2	**3b**	F	25–95	12	72
3	**3c**	Cl	25–95	12	77
4	**3d**	Br	25–95	12	74
5	**3e**	I	25–95	12	73
6	**3f**	CN	25–95	12	60
7	**3g**	NO_2_	25–95	12	76
8	**3h**	Me	25–95	12	67
9	**3i**	Et	25–95	12	55
10	**3j**	OMe	25–95	12	68

^a^ All reactions were performed with 1.0 equiv mol of **3** and 3.0 equiv mol of POCl_3_ in 7 mL in DMF at 95 °C. ^b^ Isolated yield after column chromatography.

**Table 3:** Reaction conditions and yields for the synthesis of (4S*, 5S*)-5-(1,3-diphenyl-1*H*-pyrazol-4-yl)-4-tosyl-4,5-dihydrooxazoles **6a-j** ^a^.

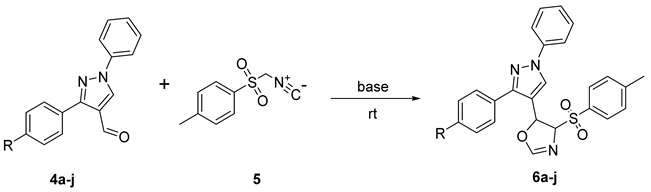			
Entry ^a,b^	4a-j	R	6a-j (%) ^c^
1	**4a**	H	46
2	**4b**	F	54
3	**4c**	Cl	25
4	**4d**	Br	27
5	**4e**	I	25
6	**4f**	CN	12
7	**4g**	NO_2_	66
8	**4h**	Me	48
9	**4i**	Et	55
10	**4j**	OMe	68
11	**4a**	H	81
12	**4b**	F	86
13	**4c**	Cl	85
14	**4d**	Br	80
15	**4e**	I	38
16	**4f**	CN	69
17	**4g**	NO_2_	63
18	**4h**	Me	71
19	**4i**	Et	66
20	**4j**	OMe	62

^a^ Reactions of entries 1–10 were performed with 1.0 equiv mol of **4**, 1.8 equiv mol of TosMIC **5**, and 2.5 equiv mol of K_2_CO_3_ in THF at rt for 3 h. ^b^ Reactions of entries 11-20 were performed with 1.0 equiv mol of **4**, 1.2 equiv mol of TosMIC **5**, and 1.2 equiv mol of KOH in MeOH at rt for 3 h. ^c^ Isolated yield after column chromatography.

**Table 4:** Pharmacokinetics and physicochemical properties of 1,3-diphenyl-1*H*-pyrazole-4-carbaldehydes **4a-j** and fluconazole **20**.

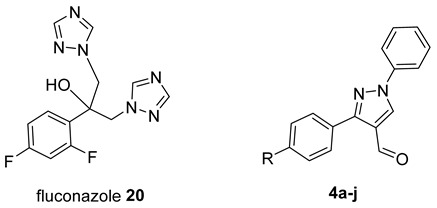										
Compound	R	MW (g/mol)	Log P	Log S	PSA	LE	GI Absorption	BBB Permeant	H-A	H-D
**Fluconazole**	-	306.27	−0.1089	−2.170	81.65	0.406	High	No	7	1
**4a**	H	248.284	2.5119	−3.453	34.89	0.476	High	Yes	3	0
**4b**	F	266.274	2.6127	−3.767	34.89	0.450	High	Yes	3	0
**4c**	cl	282.729	3.1179	−4.189	34.89	0.449	High	Yes	3	0
**4d**	Br	327.18	3.2371	−4.287	34.89	0.444	High	Yes	3	0
**4e**	I	374.176	2.949	−4.469	34.89	0.440	High	Yes	3	0
**4f**	CN	273.294	2.3475	−4.226	58.58	0.428	High	Yes	4	0
**4g**	NO_2_	293.281	1.5903	−3.913	80.71	0.407	High	No	6	0
**4h**	Me	262.311	2.8558	−3.797	34.89	0.451	High	Yes	3	0
**4i**	Et	276.338	3.2714	−3.956	34.89	0.428	High	Yes	3	0
**4j**	OMe	278.31	2.4419	−3.471	44.12	0.428	High	Yes	4	0

**Table 5:** Pharmacokinetics and physicochemical properties of (4S*, 5S*)-5-(1,3-diphenyl-1*H*-pyrazol-4-yl)-4-tosyl-4,5-dihydrooxazoles **6a-j** and fluconazole **20**.

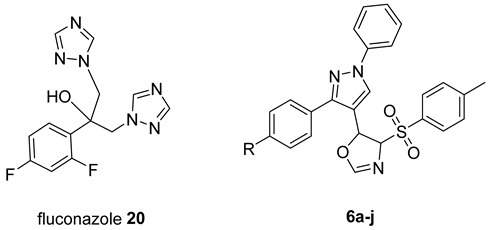										
Compound	R	MW (g/mol)	Log P	Log S	PSA	LE	GI Absorption	BBB Permeant	H-A	H-D
**Fluconazole**	-	306.27	−0.1089	−2.170	81.65	0.406	High	No	7	1
**6a**	H	443.526	3.3355	−5.328	81.93	0.264	High	No	6	0
**6b**	F	461.516	3.4363	−5.642	81.93	0.37	High	No	6	0
**6c**	cl	477.971	3.9415	−6.064	81.93	0.369	High	No	6	0
**6d**	Br	522.422	4.0607	−6.162	81.93	0.366	High	No	6	0
**6e**	I	569.418	3.7726	−6.344	81.93	0.355	High	No	6	0
**6f**	CN	468.536	3.1711	−6.101	105.72	0.34	High	No	7	0
**6g**	NO_2_	488.523	2.4139	−5.788	127.75	0.371	Low	No	9	0
**6h**	Me	457.553	3.6794	−5.672	81.93	0.355	High	No	6	0
**6i**	Et	471.58	4.095	−5.095	81.93	0.377	High	No	6	0
**6j**	OMe	473.552	3.2655	−5.346	91.16	0.2588	High		7	0

**Table 6:** Estimated risk of toxicity for 1,3-diphenyl-1*H*-pyrazole-4-carbaldehydes **4a-j** and the reference drug, fluconazole **20** (inhibitors of CYP51).

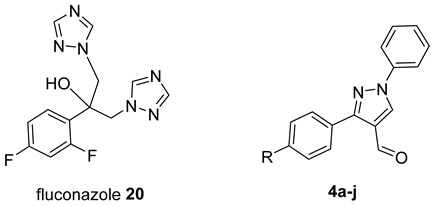					
Compound	R	Tumorigenic	Mutagenic	Reproductive Effects	Irritant
**Fluconazole**	-	No	No	No	No
**4a**	H	No	No	No	No
**4b**	F	No	No	No	No
**4c**	Cl	No	No	No	No
**4d**	Br	No	No	No	No
**4e**	I	No	No	No	No
**4f**	CN	No	No	No	No
**4g**	NO_2_	No	No	No	No
**4h**	Me	No	No	No	No
**4i**	Et	No	No	No	No
**4j**	OMe	No	No	No	No

**Table 7:** Estimated risk of toxicity for (4S*, 5S*)-5-(1,3-diphenyl-1*H*-pyrazol-4-yl)-4-tosyl-4,5-dihydrooxazoles **6a-j** and the reference drug, fluconazole **20** (inhibitors of CYP51).

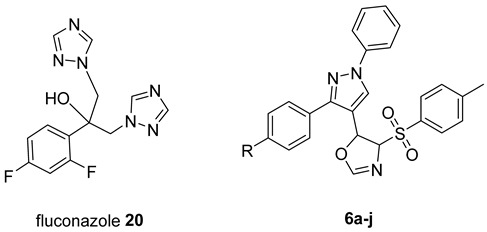					
Compound	R	Tumorigenic	Mutagenic	Reproductive Effects	Irritant
**Fluconazole**	-	No	No	No	No
**6a**	H	No	No	No	No
**6b**	F	No	No	No	No
**6c**	Cl	No	No	No	No
**6d**	Br	No	No	No	No
**6e**	I	No	No	No	No
**6f**	CN	No	No	No	No
**6g**	NO_2_	No	No	No	No
**6h**	Me	No	No	No	No
**6i**	Et	No	No	No	No
**6j**	OMe	No	No	No	No

**Table 8:** Binding energy of 1,3-diaryl-1*H*-pyrazole-4-carbaldehydes **4a-j**, (4S*, 5S*)-5-(1,3-diaryl-1*H*-pyrazol-4-yl)-4-tosyl-4,5-dihydrooxazoles **6a-j**, and fluconazole **20** at the active site of lanosterol 14-alpha demethylase CYP51 from *C. albicans*.

Compound		Binding Energy ΔG (kcal/mol)					
	R	*C. albicans*	*C. auris*	*C. dubliniensis*	*C. glabrata*	*C. haemulonii*	*C. krusei*
**Fluconazole**	-	−7.29	−7.48	−7.27	−7.5	−7.41	−6.93
**4a**	H	−8.94	−9.32	−9.15	−9.34	−9.22	−8.80
**4b**	F	−8.92	−9.34	−9.07	−9.21	−9.18	−8.87
**4c**	Cl	−9.55	−9.6	−9.45	−9.67	−9.63	−9.48
**4d**	Br	−9.81	−9.69	−9.31	−9.76	−9.56	−9.61
**4e**	I	−9.77	−9.99	−9.66	−9.81	−9.42	−9.34
**4f**	CN	−9.64	−9.91	−9.24	−9.98	−9.55	−9.77
**4g**	NO_2_	−9.27	−9.28	−9.18	−9.22	−9.16	−9.23
**4h**	Me	−9.40	−9.47	−9.44	−9.65	−9.61	−9.28
**4i**	Et	−9.76	−9.97	−9.97	−10.09	−9.74	−9.47
**4j**	OMe	−9.41	−9.75	−9.25	−9.65	−9.40	−9.09
**6a**	H	−13.28	−13.50	−13.19	−13.21	−13.06	−13.13
**6b**	F	−13.04	−13.47	−13.02	−13.10	−12.08	−12.67
**6c**	Cl	−13.10	−13.75	−13.20	−13.21	−13.06	−12.76
**6d**	Br	−14.23	−13.63	−12.97	−13.27	−13.24	−12.80
**6e**	I	−12.99	−13.63	−12.42	−13.33	−13.33	−12.49
**6f**	CN	−13.56	−13.83	−13.62	−13.31	−13.46	−13.05
**6g**	NO_2_	−12.44	−13.99	−12.98	−12.79	−12.53	−12.56
**6h**	Me	−13.21	−13.81	−13.15	−13.29	−12.98	−12.73
**6i**	Et	−13.10	−14.04	−13.42	−13.32	−13.46	−12.89
**6j**	OMe	−13.02	−9.75	−13.33	−13.19	−13.05	−12.31

**Table 9:** Interactions of 1,3-diaryl-1*H*-pyrazole-4-carbaldehydes **4a-j**, (4S*, 5S*)-5-(1,3-diaryl-1*H*-pyrazol-4-yl)-4-tosyl-4,5-dihydrooxazoles **6a-j**, and fluconazole **20** at the active site of the lanosterol 14-alpha demethylase CYP51 enzyme from *C. albicans*.

Compound	Interacting Residues	Interactions	
		Polar	Hydrophobic
**Fluconazole**	Tyr118, Leu121, Thr122, Phe126, Ile131, Tyr132, Phe228, Phe233, Gly303, Ile304, Gly307, Thr311, Leu376, Hem580.	O-H^… …^O (Tyr132) C-H^… …^O (Gly307) N^… …^H-C (Gly307)	π–π stacked (Tyr118) π–alkyl (Ile131) π–π T-shaped (Tyr132) π–alkyl (Ile304) π–cation (Hem580)
**4a**	Thr122, Phe126, Ile131, Tyr132, Leu139, Lys143, Leu300, Ile304, Gly307, Thr311, Leu376, Hem580.		π–alkyl (Ile131) π–sigma (Ile131) π–alkyl (Ile304) π–alkyl (Hem580) π–cation (Hem580) π–sigma (Hem580)
**4b**	Thr122, Phe126, Ile131, Tyr132, Leu139, Gln142, Lys143, Phe228, Leu300, Ile304, Gly307, Thr311, Leu376, Hem580.		π-sigma (Ile131) π–alkyl (Ile304) π–cation (Hem580) halogen (Hem580) π–sigma (Hem580)
**4c**	Thr122, Phe126, Ile131, Tyr132, Leu139, Gln142, Lys143, Phe228, Leu300, Ile304, Gly307, Thr311, Leu376, Hem580.		π–sigma (Ile131) π–alkyl (Ile304) π–alkyl (Leu376) halogen (Leu376) halogen (Hem580) π–sigma (Hem580) π–π stacked (Hem580)
**4d**	Phe126, Ile131, Leu139, Gln142, Lys143, Leu300, Gly303, Ile304, Gly307, Thr311, Leu376, Hem580.		halogen (Ile131) π–sigma (Ile131) halogen (Leu139) halogen (Lys143) halogen (Leu300) π–alkyl (Ile304) π–alkyl (Ile376) π–cation (Hem580) π–sigma (Hem580)
**4e**	Thr122, Phe126, Ile131, Tyr132, Leu139, Gln142, Lys143, Leu300, Ile304, Gly307, Thr311, Leu376, Hem580.		halogen (Ile131) π–sigma (Ile131) halogen (Leu139) halogen (Leu300) π–alkyl (Ile376) π–cation (Hem580) π–sigma (Hem580)
**4f**	Tyr118, Thr122, Phe126, Ile131, Tyr132, Leu139, Lys143, Leu300, Ile304, Gly307, Thr311, Pro375, Leu376, Hem580.		π–sigma (Ile131) π–alkyl (Ile304) π–alkyl (Ile376) π–cation (Hem580) π–sigma (Hem580)
**4g**	Thr122, Phe126, Ile131, Tyr132, Leu139, Gln142, Lys143, Ala146, Leu300, Ile304, Gly307, Thr311, Leu376, Hem580.		π–sigma (Ile131) π–alkyl (Ile304) π–alkyl (Ile376) π–cation (Hem580) π–sigma (Hem580)
**4h**	Tyr118, Thr122, Phe126, Ile131, Tyr132, Leu139, Lys143, Leu300, Ile304, Thr311, Leu376, Hem580.		π–sigma (Ile131) π–alkyl (Ile304) π–alkyl (Ile376) alkyl (Ile376) π–cation (Hem580) π–sigma (Hem580)
**4i**	Phe126, Ile131, Leu139, Gln142, Lys143, Leu300, Gly303, Ile304, Gly307, Thr311, Leu376, Hem580.		Π–sigma (Ile131) alkyl (Lys143) alkyl (Leu300) π–alkyl (Ile304) π–alkyl (Ile376) alkyl (Hem508) π–cation (Hem580) π–sigma (Hem580)
**4j**	Thr122, Phe126, Ile131, Tyr132, Leu139, Gln142, Lys143, Ala146, Leu300, Ile304, Gly307, Thr311, Leu376, Ile471, Hem580.	C-H^… …^O (Gln142)	π–sigma (Ile131) π–alkyl (Ile304) π–alkyl (Ile376) π–cation (Hem580) π–sigma (Hem580)
**6a**	Tyr118, Leu121, Thr122, Tyr132, Phe228, Pro230, Phe233, Met306, Gly307, Gly308, His310, Thr311, Leu376, Ser378, Ile379, Phe380, Met508, Val509, Hem580.	O^… …^H-O (Tyr118) O^… …^H-O (Tyr132)	π–alkyl (Leu121) π–π T-shaped (Phe228, Phe233) π–alkyl (Ile376) π–sigma (Ile376) π–alkyl (Met508) π–sulfur (Met508) π–alkyl (Val509) π–alkyl (Hem580) π–π stacked (Hem580)
**6b**	Tyr118, Leu121, Thr122, Tyr132, Phe228, Pro230, Phe233, Met306, Gly307, His310, Thr311, Leu376, His377, Ser378, Ile379, Phe380, Met508, Val509, Hem580.	O^… …^H-O (Tyr118) O^… …^H-O (Tyr132) N^… …^H-N (Met508)	π–sulfur (Tyr132) π–π T-shaped (Phe228, Phe233) halogen (Met306. Gly307, Thr311) π–alkyl (Ile376) π–π T-shaped (His377) π–alkyl (met508, Val509) π–sulfur (Met508) π–alkyl (Hem580) π–π stacked (Hem580)
**6c**	Tyr118, Leu121, Thr122, Phe126, Ile131, Tyr132, Phe228, Pro230, Phe233, Gly307, His310, Thr311, Leu376, Ser378, Phe380, Ser507, Met508, Val509, Hem580.		π–π T-shaped (Tyr118) π–alkyl (Leu121) halogen (Phe126, Ile131, Tyr132) alkyl (Ile131) π–π T-shaped (Phe228, Phe233) π–alkyl (Pro230) π–sigma (Leu376) alkyl (Hem580) π–cation (Met508)
**6d**	Ala114, Tyr118, Tyr132, Phe228, Pro230, Phe233, Gly307, His310, Thr311, Leu376, Ser378, Phe380, Ser507, Met508, Val509, Hem580.		π–alkyl (Tyr118) π–π T-shaped (Phe228) π–alkyl (Pro230) alkyl (Pro230) π–sigma (Phe233) π–alkyl (Ile376, His468) π–alkyl (Met508, Val509)
**6e**	Tyr118, Leu121, Tyr132, Phe228, Pro230, Phe233, Met306, Gly307, His310, Thr311, Leu376, His377, Ser378, Ile379, Phe380, Arg381, Ser507, Met508, Val509, Hem580.	N^… …^H-O (Tyr118) C-H^… …^O (Ser378) N^… …^H-N (Met508)	π–alkyl (Pro230) halogen (Pro230. His377) π–alkyl (Ile376) π–π T-shaped (His377) π–alkyl (met508, Val509) π–π sigma (Hem580) π–π stacked (Hem580)
**6f**	Tyr118, Leu121, Thr122, Phe126, Ile131, Tyr132, Phe228, Pro230, Phe233, Gly307, His310, Thr311, Leu376, Ser378, Phe380, Ser507, Met508, Val509, Hem580.		π–π T-shaped (Tyr118) alkyl (Ile131) π–π T-shaped (Phe228, Phe233) π–alkyl (Pro230) π–sigma (Leu376) π–sulfur (Met508) alkyl (Hem580) π–cation (Met508)
**6g**	Tyr118, Leu121, Thr122, Phe126, Ile131, Tyr132, Phe228, Pro230, Phe233, Met306, Gly307, His310, Thr311, Leu376, Ser378, Phe380, Met508, Val509, Hem580.	C-H^… …^O (Met508)	π–π T-shaped (Tyr118) π–alkyl (Tyr118) π–sigma (Thr122) π–alkyl (Tyr132) π–alkyl (Pro230) π–π T-shaped (Phe233) π–sigma (Leu376) π–sulfur (Met508) alkyl (Hem580) π–cation (Met508)
**6h**	Tyr118, Leu121, Thr122, Phe126, Ile131, Tyr132, Phe228, Pro230, Phe233, Met306, Gly307, His310, Thr311, Leu376, Ser378, Phe380, Met508, Val509, Hem580.	C-H^… …^O (Met508)	π–π T-shaped (Tyr118) π–alkyl (Leu121) π–sigma (Thr122) π–alkyl (Phe126) alkyl (Ile131) π–alkyl (Tyr132) π–π T-shaped (Phe228, Phe233) π–alkyl (Pro230) π–sigma (Leu376) π–sulfur (Met508) alkyl (Hem580) π–cation (Met508)
**6i**	Tyr118, Leu121, Thr122, Phe126, Ile131, Tyr132, Phe228, Pro230, Phe233, Met306, Gly307, His310, Thr311, Leu376, His377, Ser378, Met508, Val509, Hem580.	C-H^… …^O (Met508)	π–π T-shaped (Tyr118) π–alkyl (Tyr118) π–alkyl (Leu121, Phe126) alkyl (Ile131) π–alkyl (Tyr132) π–π T-shaped (Phe228, Phe233) π–alkyl (Pro230) π–alkyl (Ile376) π–sigma (Leu376) π–sulfur (Met508) π–alkyl (Hem580) π–cation (Met508)
**6j**	Tyr118, Leu121, Thr122, Phe126, Ile131, Tyr132, Phe228, Pro230, Phe233, Gly307, His310, Thr311, Leu376, Ser378, Phe380, Met508, Val509, Hem580.	C-H^… …^O (Met508)	Π–π T-shaped (Tyr118) alkyl (Ile131) π–alkyl (Pro230) π–π T-shaped (His377) π–sulfur (Met508) alkyl (Hem580) π–cation (Met508)

**Table 10:** MIC_70_ and MIC_90_ values of the series of 1,3-diaryl-1*H*-pyrazole-4-carbaldehydes **4a-j** against *Candida* species.

Compound	*C. albicans*		*C. auris*		*C. dubliniensis*		*C. glabrata*		*C. haemulonii*		*C. krusei*	
	MIC_70_	MIC_90_	MIC_70_	MIC_90_	MIC_70_	MIC_90_	MIC_70_	MIC_90_	MIC_70_	MIC_90_	MIC_70_	MIC_90_
	µg/mL		µg/mL		µg/mL		µg/mL		µg/mL		µg/mL	
**Fluconazole**	1.4	1.8	>44.8	>57.6	1.4	1.8	5.6	7.2	>44.8	>57.6	5.6	7.2
**4a**	0.39	0.50	10.80	14.0	0.34	0.45	0.39	0.52	5.76	7.36	0.17	0.22
**4b**	0.61	0.79	2.80	3.60	0.10	0.14	0.32	0.43	1.42	1.85	0.17	0.22
**4c**	0.25	0.29	0.64	0.83	0.09	0.14	0.20	0.26	2.76	3.57	0.08	0.11
**4d**	0.17	0.22	11.27	14.37	0.43	0.62	0.15	0.20	2.72	3.62	0.08	0.11
**4e**	0.17	0.22	5.45	6.9	0.24	0.35	0.24	0.32	2.75	3.62	0.08	0.11
**4f**	0.65	0.86	4.2	5.25	0.20	0.28	0.26	0.30	2.70	3.52	0.08	0.11
**4g**	0.29	0.37	2.81	3.63	0.21	0.31	0.23	0.30	5.60	7.20	0.17	0.22
**4h**	0.64	0.82	11.2	14.4	0.20	0.30	0.21	0.28	2.92	3.69	0.17	0.22
**4i**	0.17	0.22	3.4	4.37	0.99	1.27	0.69	0.88	3.62	4.65	0.11	0.14
**4j**	0.42	0.54	10.43	13.92	0.21	0.30	0.25	0.32	5.60	7.30	0.08	0.11

MIC_70_: the lowest concentration of the compound at which 70% of yeast growth was inhibited (compared to the control well). MIC_90_: the lowest concentration of the compound at which 90% of yeast growth was inhibited (compared to the control well).

**Table 11:** MIC_70_ and MIC_90_ values of the series of (4S*, 5S*)-5-(1,3-diaryl-1*H*-pyrazol-4-yl)-4-tosyl-4,5-dihydrooxazoles **6a-j** against *Candida* species.

Compound	*C. albicans*		*C. auris*		*C. dubliniensis*		*C. glabrata*		*C. haemulonii*		*C. krusei*	
	MIC_70_	MIC_90_	MIC_70_	MIC_90_	MIC_70_	MIC_90_	MIC_70_	MIC_90_	MIC_70_	MIC_90_	MIC_70_	MIC_90_
	µg/mL		µg/mL		µg/mL		µg/mL		µg/mL		µg/mL	
**Fluconazole**	1.4	1.8	>44.8	>57.6	1.4	1.8	5.6	7.2	>44.8	>57.6	5.6	7.2
**6a**	0.25	0.32	2.04	2.62	0.13	0.16	0.25	0.32	16.45	21.15	0.32	0.41
**6b**	0.12	0.16	16.45	21.15	0.58	0.74	0.16	0.21	8.21	10.56	0.25	0.32
**6c**	0.12	0.16	16.45	21.15	0.34	0.43	0.16	0.21	4.10	5.27	0.50	0.64
**6d**	0.12	0.16	0.50	0.64	0.04	0.05	0.28	0.36	2.04	2.68	0.05	0.07
**6e**	0.12	0.16	4.10	5.27	0.42	0.54	0.14	0.18	16.45	21.15	0.54	0.70
**6f**	0.25	0.32	0.25	0.32	0.34	0.43	0.61	0.79	16.45	21.15	1.00	1.29
**6g**	0.25	0.32	0.50	0.64	0.34	0.43	0.056	0.072	16.45	21.15	0.23	0.30
**6h**	0.50	0.64	32.9	42.3	0.18	0.23	0.07	0.09	32.9	42.3	0.21	0.27
**6i**	0.25	0.32	32.9	42.3	0.58	0.74	0.22	0.28	16.45	21.15	0.23	0.30
**6j**	0.12	0.16	2.04	2.62	0.10	0.12	0.39	0.50	32.9	42.3	0.7	0.9

## Data Availability

The data supporting this article will be shared upon reasonable request to the corresponding author.

## Supplementary Materials

The following supporting information can be downloaded at: https://www.mdpi.com/article/10.3390/ijms25105091/s1. References [76,77,78,79,80,81,82,83,84] are cited in the Supplementary Materials.
