# Supplementary material for: Synthesis, In Silico Study, and In Vitro Antifungal Activity of New 5-(1,3-Diphenyl-1H-Pyrazol-4-yl)-4-Tosyl-4,5-Dihydrooxazoles
Source: Int J Mol Sci. 2024 May 7;25(10):5091. doi: 10.3390/ijms25105091 (PMC11120875; doi:10.3390/ijms25105091)
Supplement: Supplementary file 1 [file ijms-25-05091-s001.zip › ijms-2931875-supplementary.pdf]

Synthesis, In Silico Study, and In Vitro Antifungal Activity of New 5-(1,3-Diphenyl-1*H*-Pyrazol-4-yl)-4-Tosyl-4,5-Dihydrooxazoles

Neively Tlapale-Lara [1], Julio López [1], Elizabeth Gómez [2], Lourdes Villa-Tanaca [3], Edson Barrera [1], Carlos H. Escalante [2], Joaquín Tamariz [1], Francisco Delgado [1], Dulce Andrade-Pavón [3,4,*] and Omar Gómez-García [1,*]

[1] Departamento de Química Orgánica, Escuela Nacional de Ciencias Biológicas, Instituto Politécnico Nacional, Prol. Carpio y Plan de Ayala S/N, Mexico City 11340, Mexico; neively.tlapale@gmail.com (N.T.-L.); jclmtz@hotmail.com (J.L.); ebarrerac0800@alumno.ipn.mx (E.B.); jtamarizm@gmail.com (J.T.); jfdelgador@gmail.com (F.D.)

[2] Instituto de Química, Universidad Nacional Autónoma de México, Circuito Exterior, Ciudad Universitaria, Coyoacán, Mexico City 04510, Mexico; eligom@iquimica.unam.mx (E.G.); cescalantep1700@alumno.ipn.mx (C.H.E.)

[3] Departamento de Microbiología, Laboratorio de Biología Molecular de Bacterias y Levaduras, Escuela Nacional de Ciencias Biológicas, Instituto Politécnico Nacional, Prolongación de Carpio y Plan de Ayala S/N, Colonia Santo Tomás, Mexico City 11340, Mexico; mvillat@ipn.mx

[4] Departamento de Fisiología, Escuela Nacional de Ciencias Biológicas, Instituto Politécnico Nacional, Av. Wilfrido Massieu S/N, Unidad Adolfo López Mateos, Mexico City 07738, Mexico

* Correspondence: dandradep@ipn.mx (D.A.-P.); jogomezga@ipn.mx (O.G.-G.)

Index

Characterization of series **3a-j** 3-23

Characterization of series **4a-j** 23-69

Characterization of series **6a-j** 70-124

Reaction mechanisms 125

In silico supplements 126-161

Characterization of series **3a-j**

Table S1. Reported sources addressing the characterization of hidrazones **3a-j** [76–84].

Compound	Reference
**3a** and **3h**	Hu, X., Martin, D., & Bertrand, G. (2016). Room temperature hydroamination of alkynes with anilines catalyzed by anti-Bredt di(amino)carbene gold(I) complexes. *New Journal of Chemistry*, *40*(7), 5993–5996. https://doi.org/10.1039/c6nj00980h
**3b**	Desai, N. C., Joshi, V. V., Rajpara, K. M., Vaghani, H. V., & Satodiya, H. M. (2012). Facile synthesis of novel fluorine containing pyrazole based thiazole derivatives and evaluation of antimicrobial activity. *Journal of Fluorine Chemistry*, *142*, 67–78. https://doi.org/10.1016/j.jfluchem.2012.06.021
**3c**	Yadlapalli, R. K., Chourasia, O. P., Vemuri, K., Sritharan, M., & Perali, R. S. (2012). Synthesis and in vitro anticancer and antitubercular activity of diarylpyrazole ligated dihydropyrimidines possessing lipophilic carbamoyl group. *Bioorganic and Medicinal Chemistry Letters*, *22*(8), 2708–2711. https://doi.org/10.1016/j.bmcl.2012.02.101
**3d**	Sharma, J., Tuli, H. S., Kinger, M., Pal, R., Abbas, Z., & Kumar, M. (2022). Synthesis, Characterization and Biological Screening of Novel Imidazolylpyrazole Scaffolds. *Asian Journal of Chemistry*, *34*(3), 614–618. https://doi.org/10.14233/ajchem.2022.23570
**3e**	Homes, T. P., Mattner, F., Keller, P. A., & Katsifis, A. (2006). Synthesis and in vitro binding of N,N-dialkyl-2-phenylindol-3-yl-glyoxylamides for the peripheral benzodiazepine binding sites. *Bioorganic and Medicinal Chemistry*, *14*(11), 3938–3946. https://doi.org/10.1016/j.bmc.2006.01.039
**3f**	Zhang, G., Miao, J., Zhao, Y., & Ge, H. (2012). Copper-catalyzed aerobic dehydrogenative cyclization of N-methyl-N-phenylhydrazones: Synthesis of cinnolines. *Angewandte Chemie - International Edition*, *51*(33), 8318–8321. https://doi.org/10.1002/anie.201204339
**3g**	Sandhya, P. V., Kumar, K. V. S., & Haridas, K. R. (2022). Synthesis, Molecular Docking and DFT Studies of Biologically Active N-((3-(4-Nitrophenyl)-1-phenyl-1H-pyrazol-4-yl)methylene)aniline Derivatives. *Asian Journal of Chemistry*, *34*(2), 297–304. https://doi.org/10.14233/ajchem.2022.23500
**3i**	Kishk, S. M., McLean, K. J., Sood, S., Smith, D., Evans, J. W. D., Helal, M. A., Gomaa, M. S., Salama, I., Mostafa, S. M., de Carvalho, L. P. S., Levy, C. W., Munro, A. W., & Simons, C. (2019). Design and Synthesis of Imidazole and Triazole Pyrazoles as Mycobacterium Tuberculosis CYP121A1 Inhibitors. *ChemistryOpen*, *8*(7), 995–1011. https://doi.org/10.1002/open.201900227
**3j**	La Regina, G., Bai, R., Rensen, W. M., Di Cesare, E., Coluccia, A., Piscitelli, F., Famiglini, V., Reggio, A., Nalli, M., Pelliccia, S., Da Pozzo, E., Costa, B., Granata, I., Porta, A., Maresca, B., Soriani, A., Iannitto, M. L., Santoni, A., Li, J., … Silvestri, R. (2013). Toward highly potent cancer agents by modulating the C-2 group of the arylthioindole class of tubulin polymerization inhibitors. *Journal of Medicinal Chemistry*, *56*(1), 123–149. https://doi.org/10.1021/jm3013097

The complete spectroscopic data of **3g** is presented as an illustrative example.

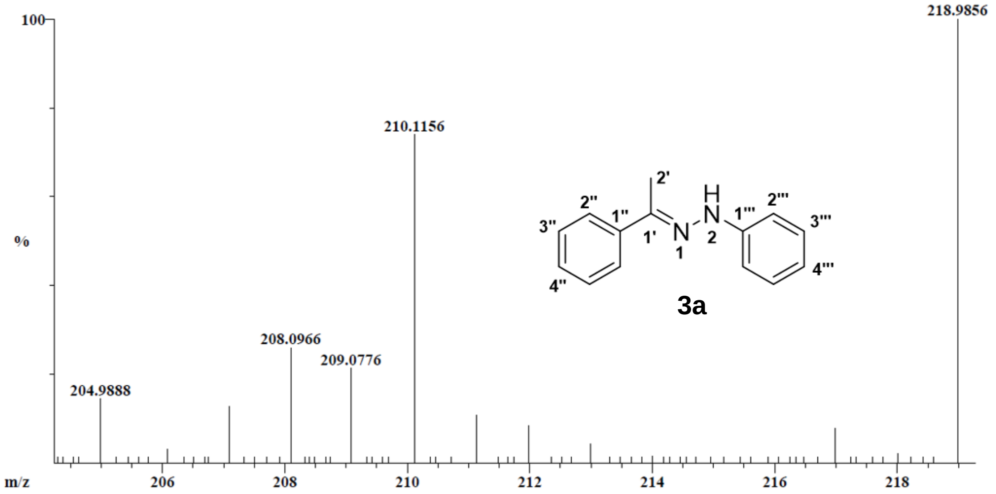

Selected Isotopes : $H_{0-14}C_{0-14}N_{0-2}$ Error Limit : 5 ppm Unsaturation Limits : 0 to 50

Measured Mass	*% Base*	*Formula*	*Calculated Mass*	*Error*	*Unsaturation*
210.1156	74.0%	$C_{14}H_{14}N_2$	210.1157	-0.5	9.0

Figure S1. HRMS of (*E*)-1-phenyl-2-(1-phenylethylidene)hydrazone **3a**.

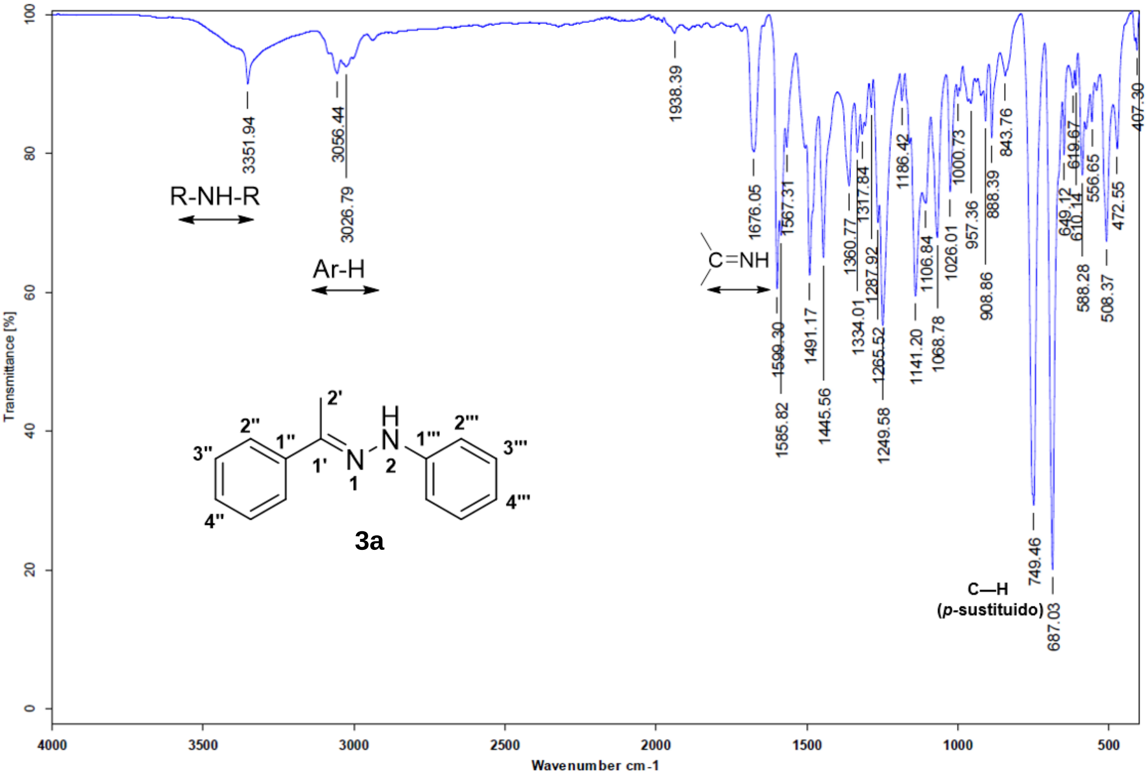

Figure S2. FT-IR of (*E*)-1-phenyl-2-(1-phenylethylidene)hydrazone **3a**.

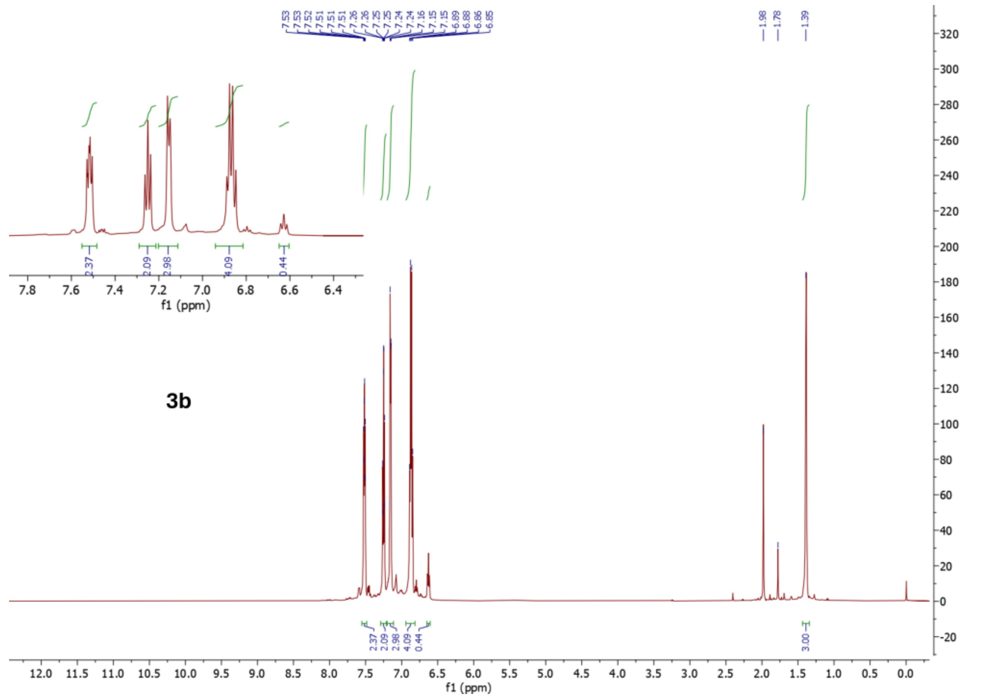

Figure S3. ^{1}H NMR (600 MHz, C_6D_6) of (*E*)-1-(1-(4-fluorophenyl)ethylidene)-2-phenylhydrazone **3b**.

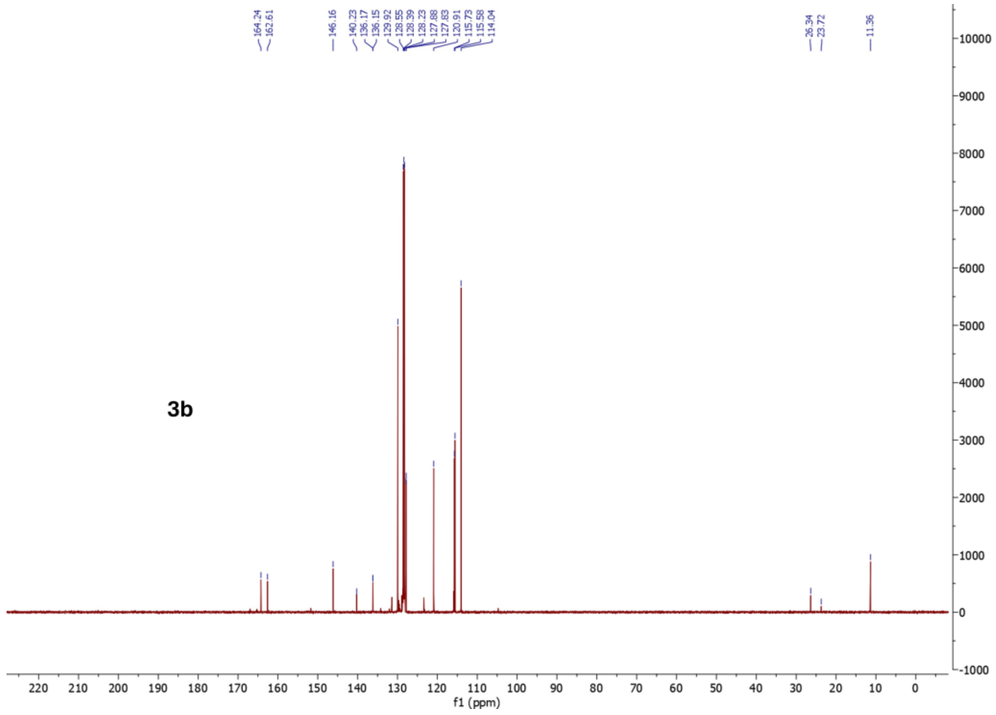

Figure S4. ^{13}C NMR (150 MHz, C_6D_6) of (*E*)-1-(1-(4-fluorophenyl)ethylidene)-2-phenylhydrazone **3b**.

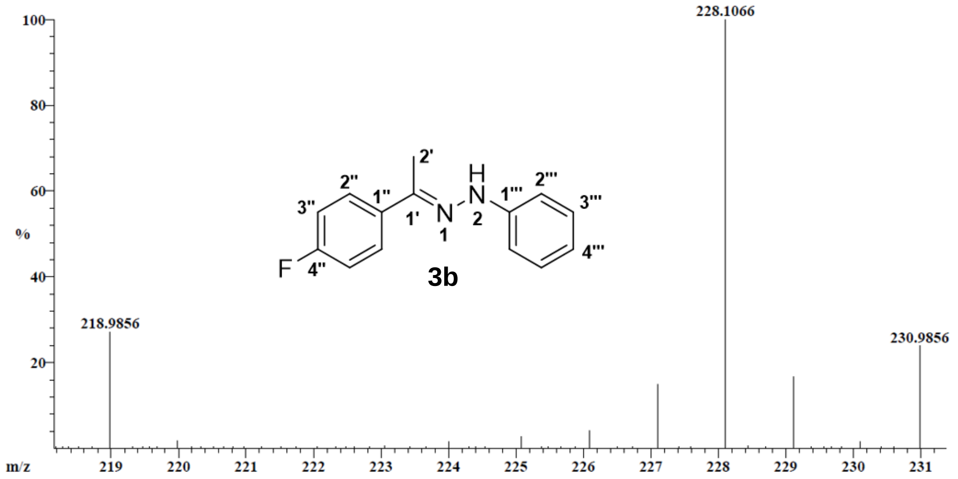

Selected Isotopes : $H_{0-13}C_{0-14}N_{0-2}F_{0-1}$ Error Limit : 5 ppm   Unsaturation Limits : 0 to 50

Measured Mass	*% Base*	*Formula*	*Calculated Mass*	*Error*	*Unsaturation*
228.1066	100.0%	$C_{14}H_{13}N_2F$	228.1063	1.4	9.0

Figure S5. HRMS of (*E*)-1-(1-(4-fluorophenyl)ethylidene)-2-phenylhydrazone **3b**.

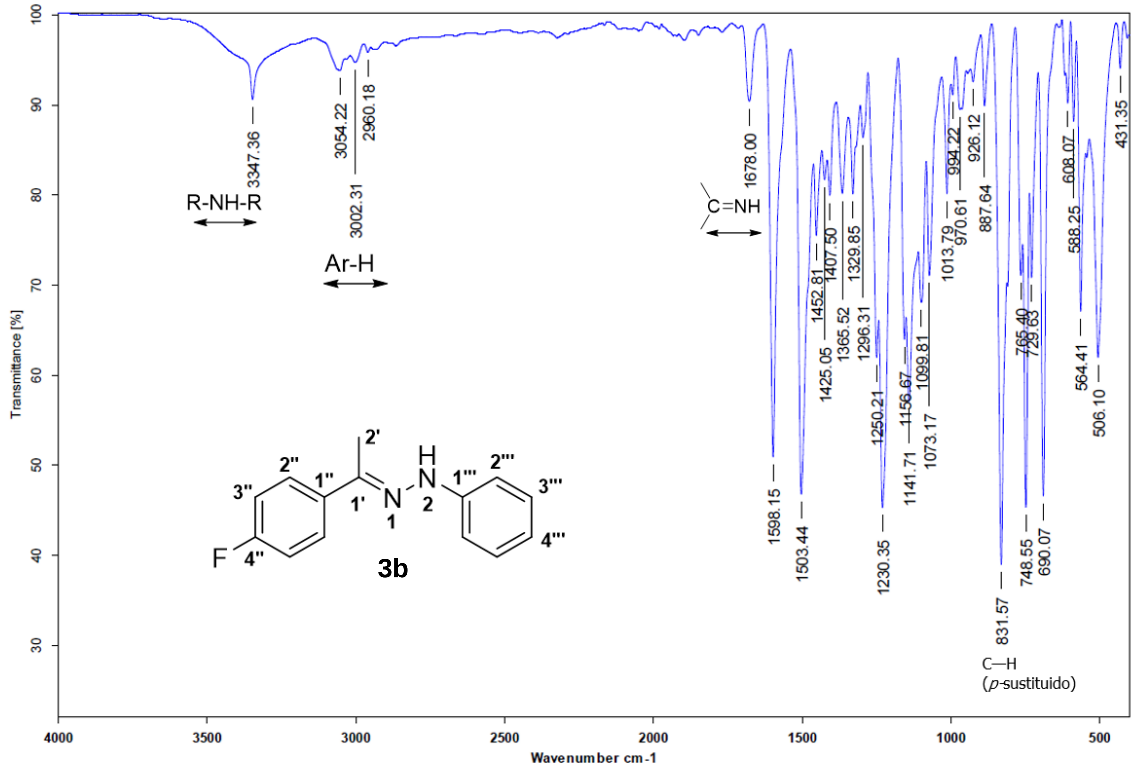

Figure S6. FT-IR of (*E*)-1-(1-(4-fluorophenyl)ethylidene)-2-phenylhydrazone **3b**.

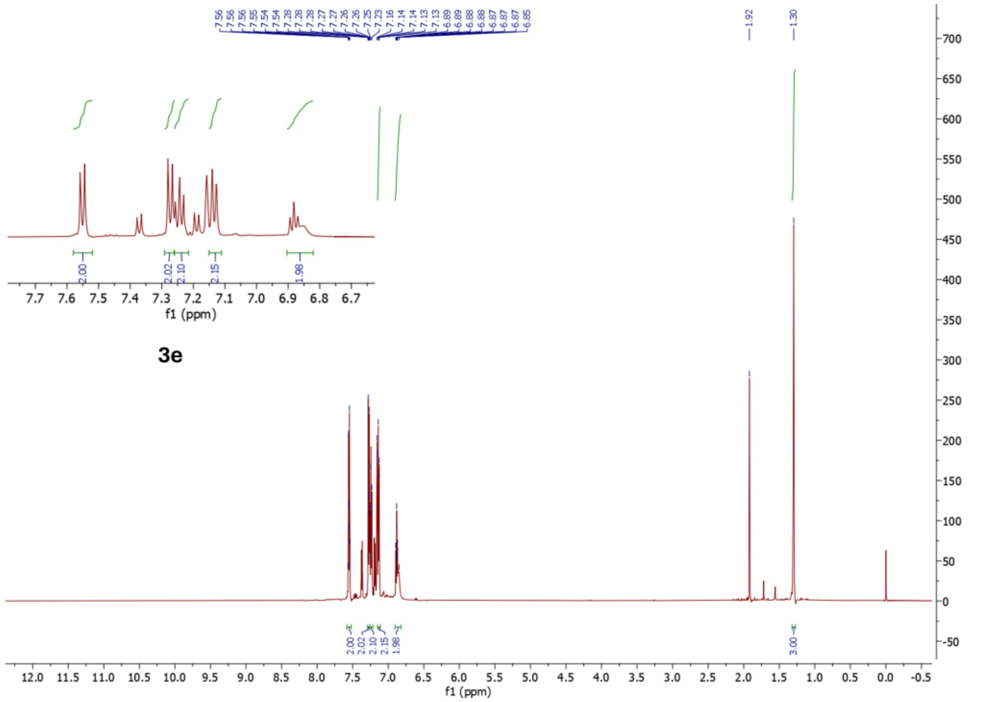

Figure S7. ^{1}H NMR (600 MHz, C_6D_6) of (*E*)-1-(1-(4-chlorophenyl)ethylidene)-2-phenylhydrazone **3c**.

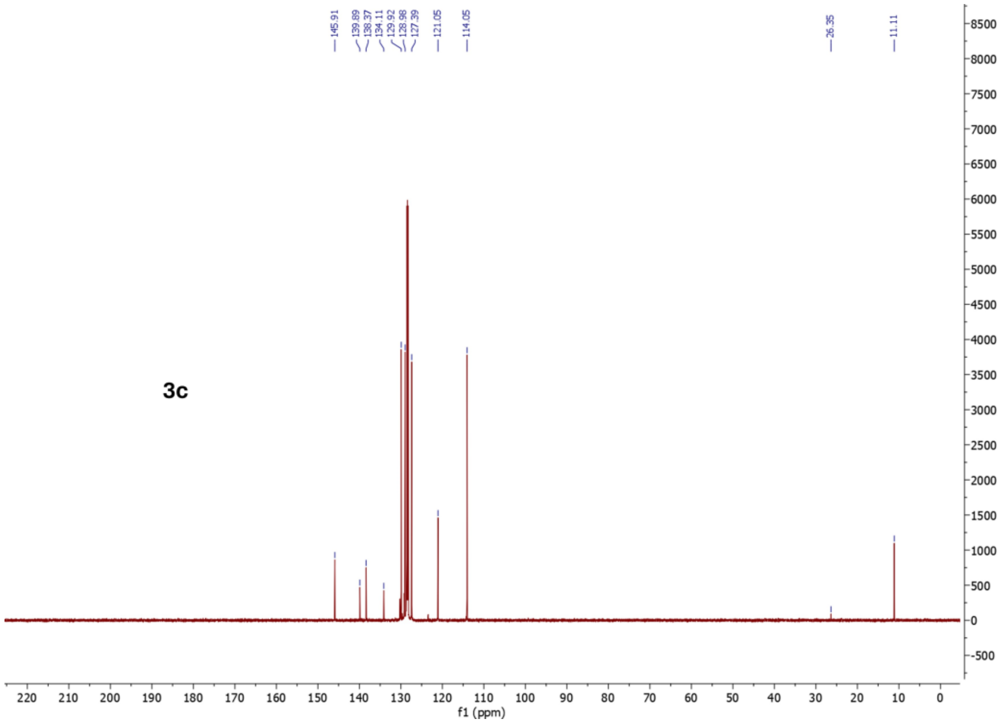

Figure S8. ^{13}C NMR (150 MHz, C_6D_6) of (*E*)-1-(1-(4-chlorophenyl)ethylidene)-2-phenylhydrazone **3c**.

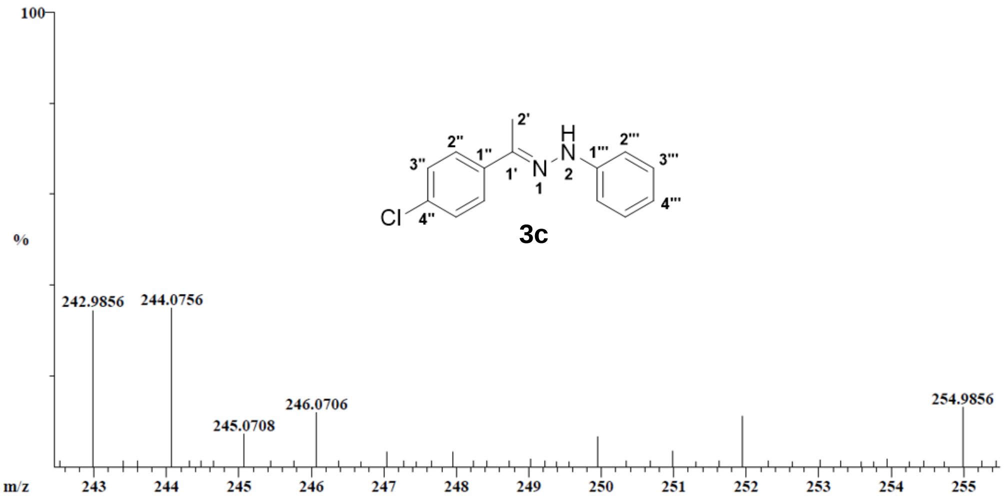

Selected Isotopes : $H_{0-13}C_{0-14}N_{0-2}Cl_{0-1}$ Error Limit : 5 ppm Unsaturation Limits : 0 to 50

Measured Mass	*% Base*	*Formula*	*Calculated Mass*	*Error*	*Unsaturation*
244.0756	34.9%	$C_{14}H_{13}N_2Cl$	244.0767	-4.6	9.0

Figure S9. HRMS of (*E*)-1-(1-(4-chlorophenyl)ethylidene)-2-phenylhydrazone **3c**.

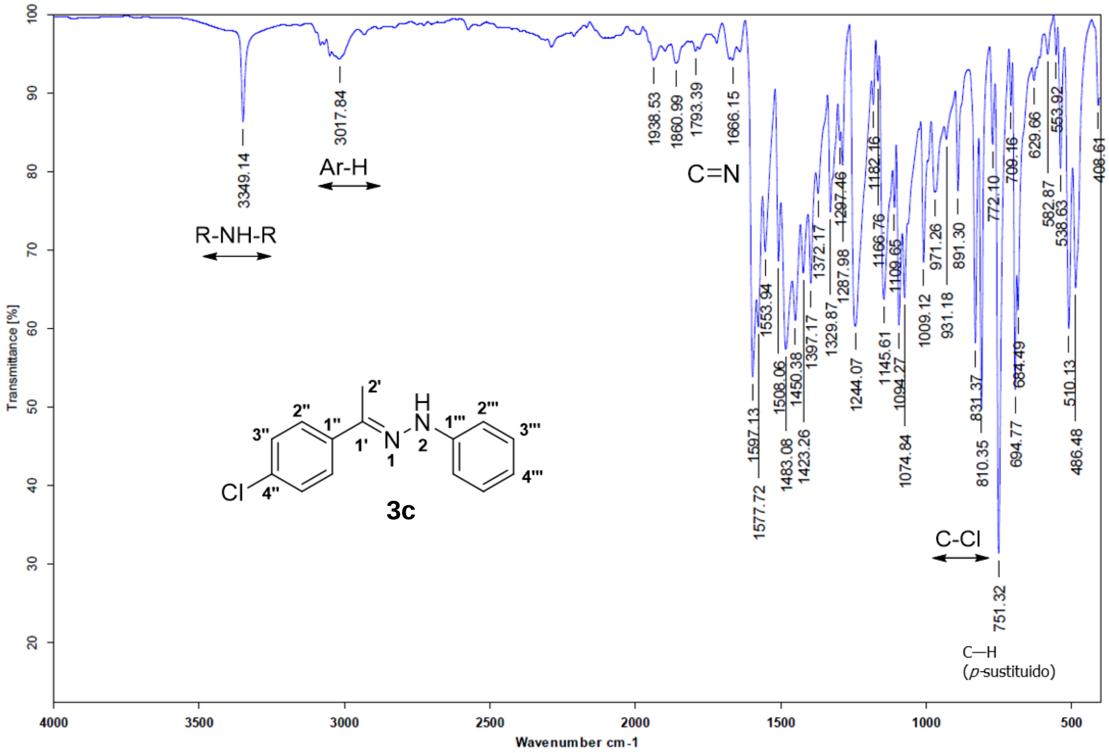

Figure S10. FT-IR of (*E*)-1-(1-(4-chlorophenyl)ethylidene)-2-phenylhydrazone **3c**.

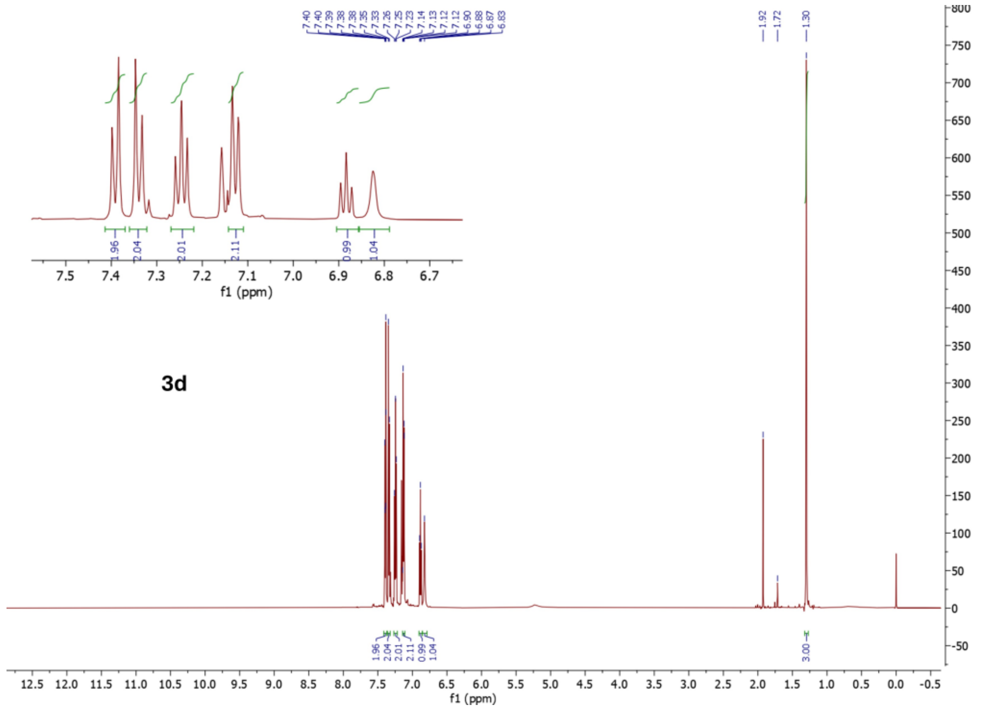

Figure S11. ^{1}H NMR (600 MHz, C_6D_6) of (*E*)-1-(1-(4-bromophenyl)ethylidene)-2-phenylhydrazone **3d**.

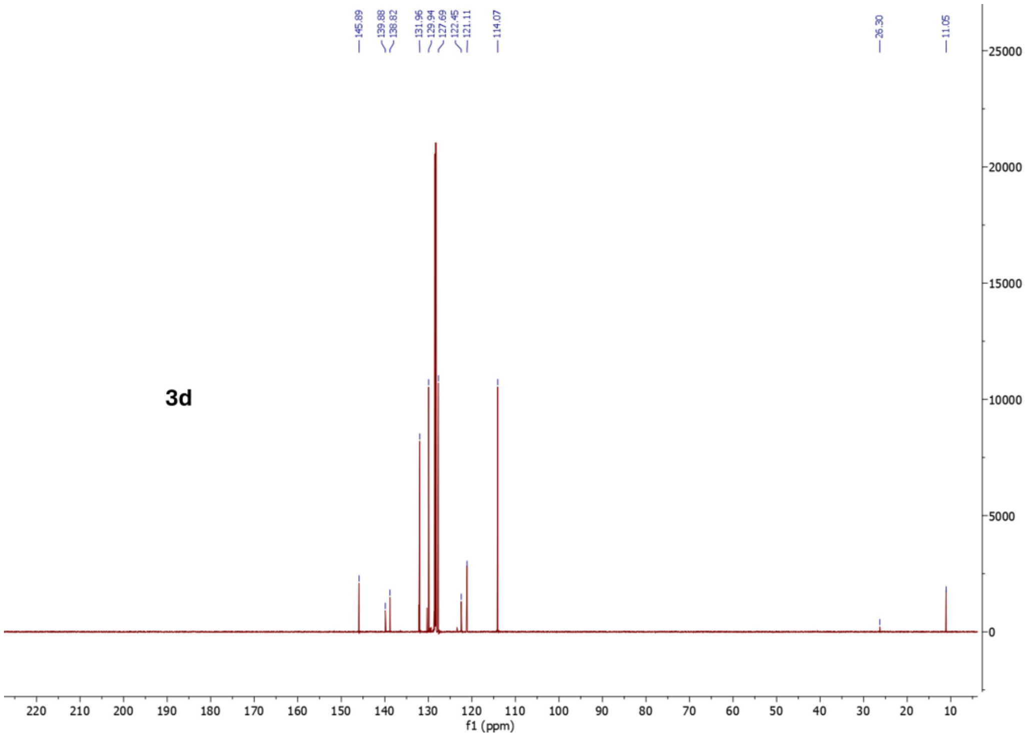

Figure S12. ^{13}C NMR (150 MHz, C_6D_6) of (*E*)-1-(1-(4-bromophenyl)ethylidene)-2-phenylhydrazone **3d**.

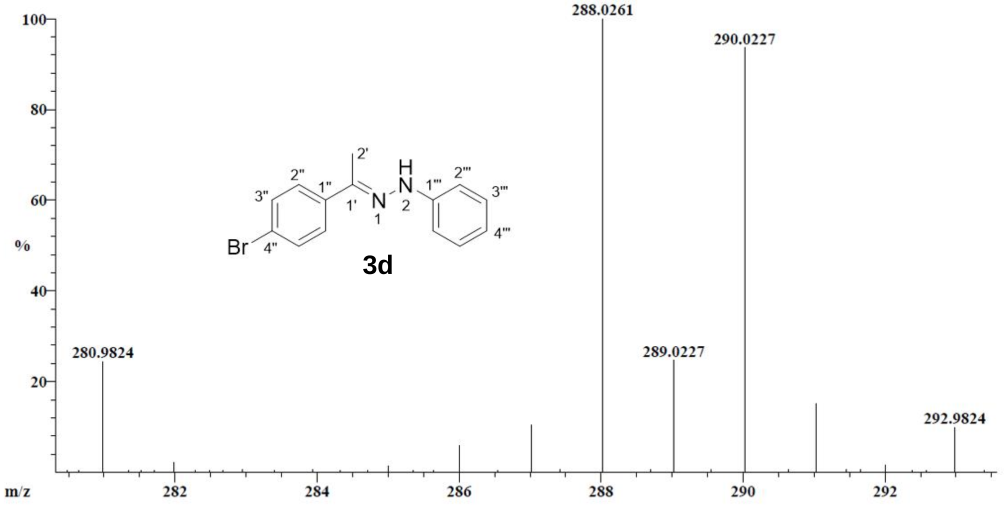

Selected Isotopes : $H_{0-13}C_{0-14}N_{0-2}Br_{0-1}$ Error Limit : 5 ppm Unsaturation Limits : 0 to 50

Measured Mass	% Base	Formula	Calculated Mass	Error	Unsaturation
288.0261	100.0%	$C_{14}H_{13}N_2Br$	288.0262	-0.4	9.0

*

Figure S13. HRMS of (*E*)-1-(1-(4-bromophenyl)ethylidene)-2-phenylhydrazone **3d**.

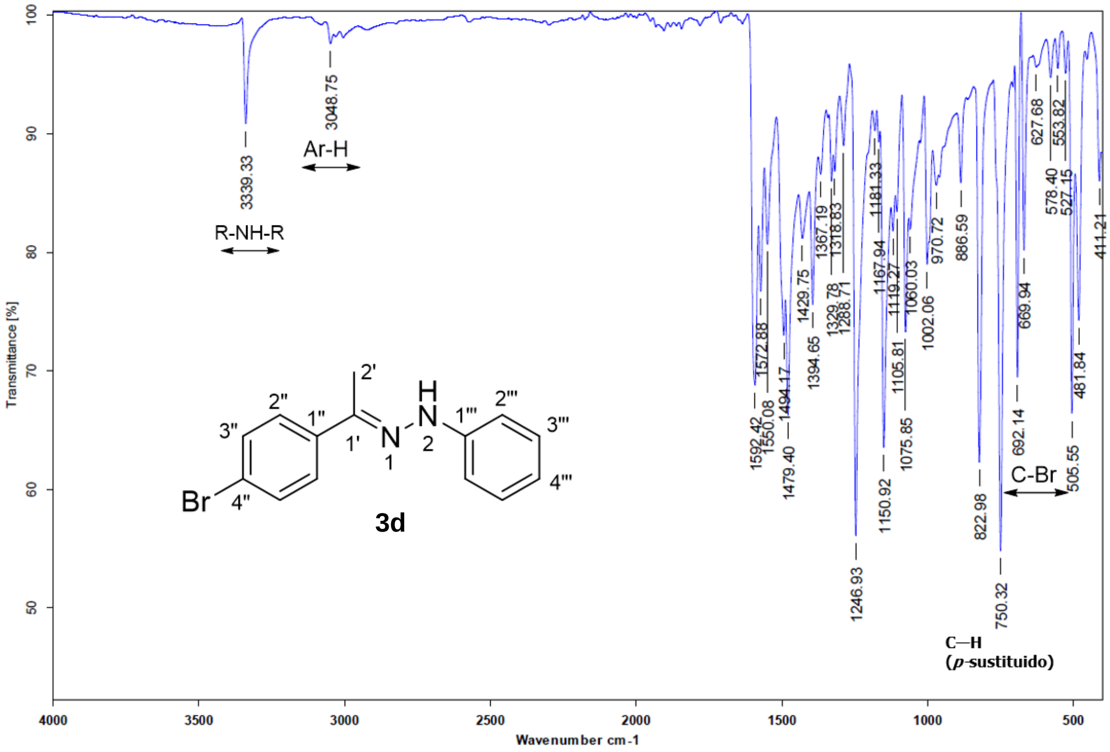

Figure S14. FT-IR of (*E*)-1-(1-(4-bromophenyl)ethylidene)-2-phenylhydrazone **3d**.

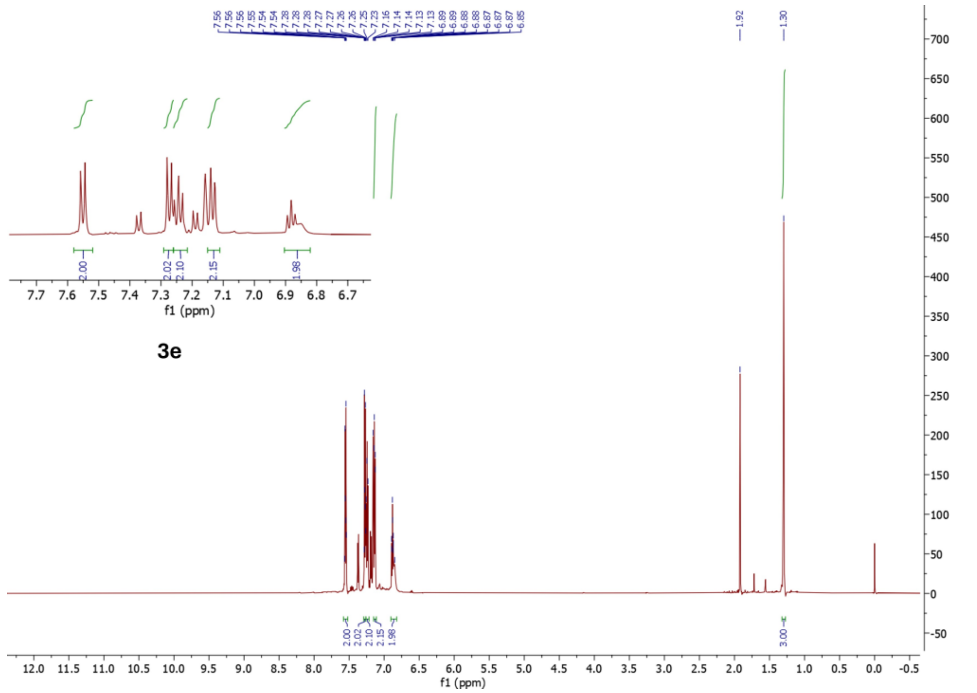

Figure S15. ^{1}H NMR (600 MHz, C_6D_6) of (*E*)-1-(1-(4-iodophenyl)ethylidene)-2-phenylhydrazone **3e.**

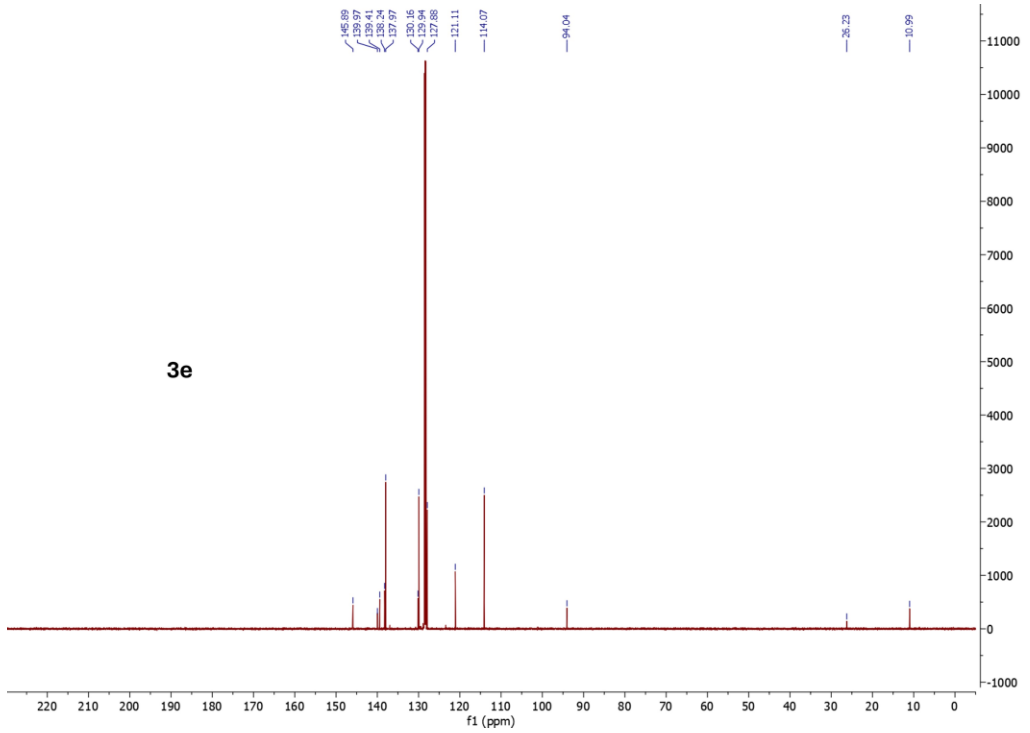

Figure S16. ^{13}C NMR (150 MHz, C_6D_6) of (*E*)-1-(1-(4-iodophenyl)ethylidene)-2-phenylhydrazone **3e.**

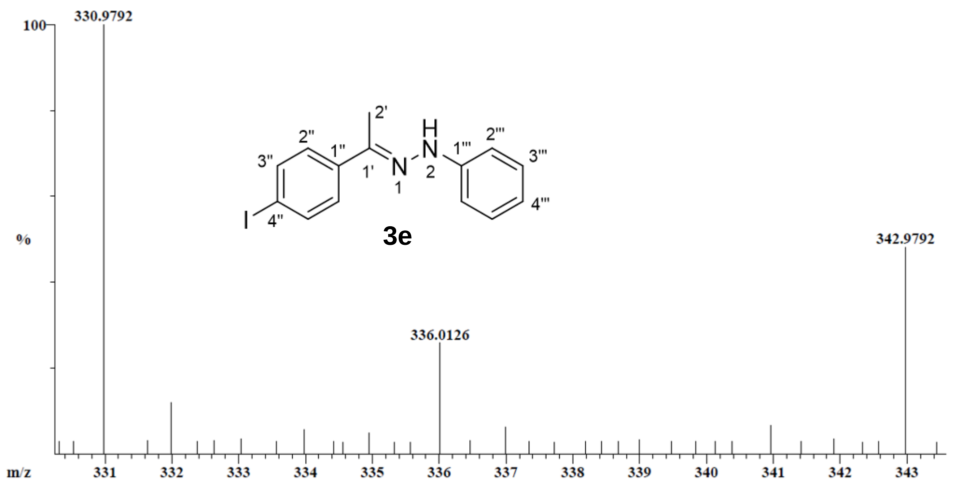

Selected Isotopes : $H_{0-13}C_{0-14}N_{0-2}I_{0-1}$ Error Limit : 5 ppm Unsaturation Limits : 0 to 50

Measured Mass	% Base	Formula	Calculated Mass	Error	Unsaturation
336.0126	25.9%	$C_{14}H_{13}N_2I$	336.0124	0.7	9.0

Figure S17. HRMS of (*E*)-1-(1-(4-iodophenyl)ethylidene)-2-phenylhydrazone **3e**.

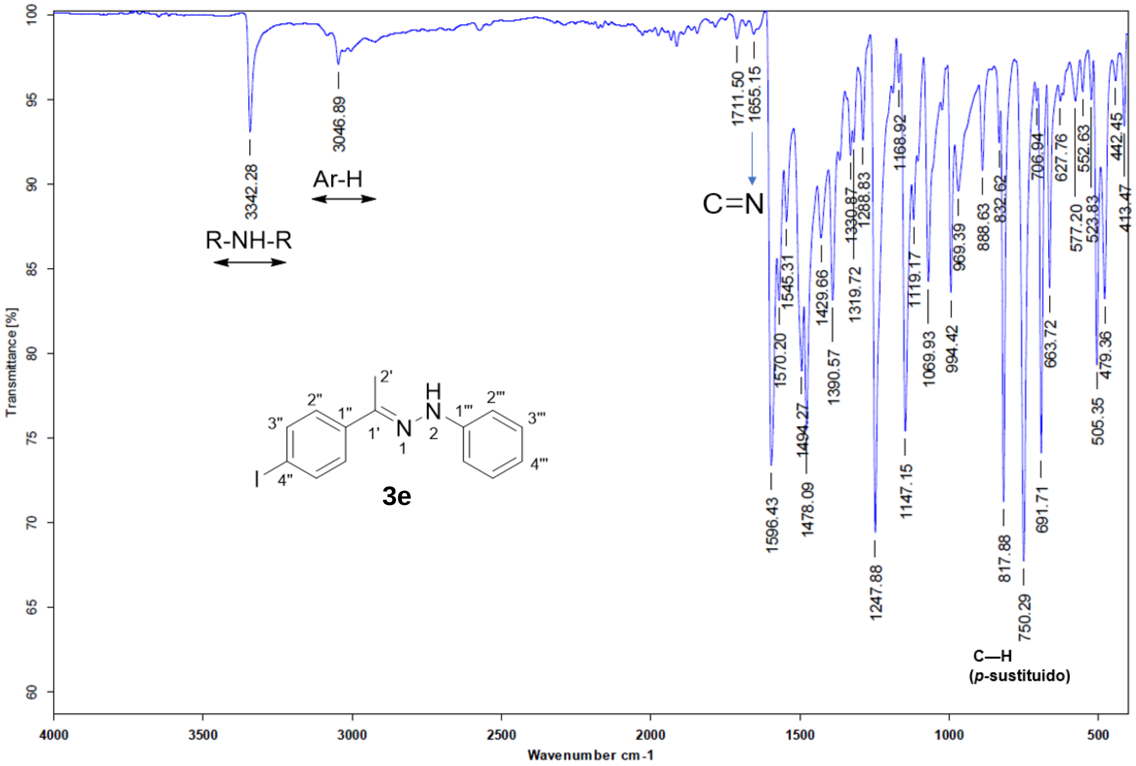

Figure S18. FT-IR of 5 (*E*)-1-(1-(4-iodophenyl)ethylidene)-2-phenylhydrazone **3e**.

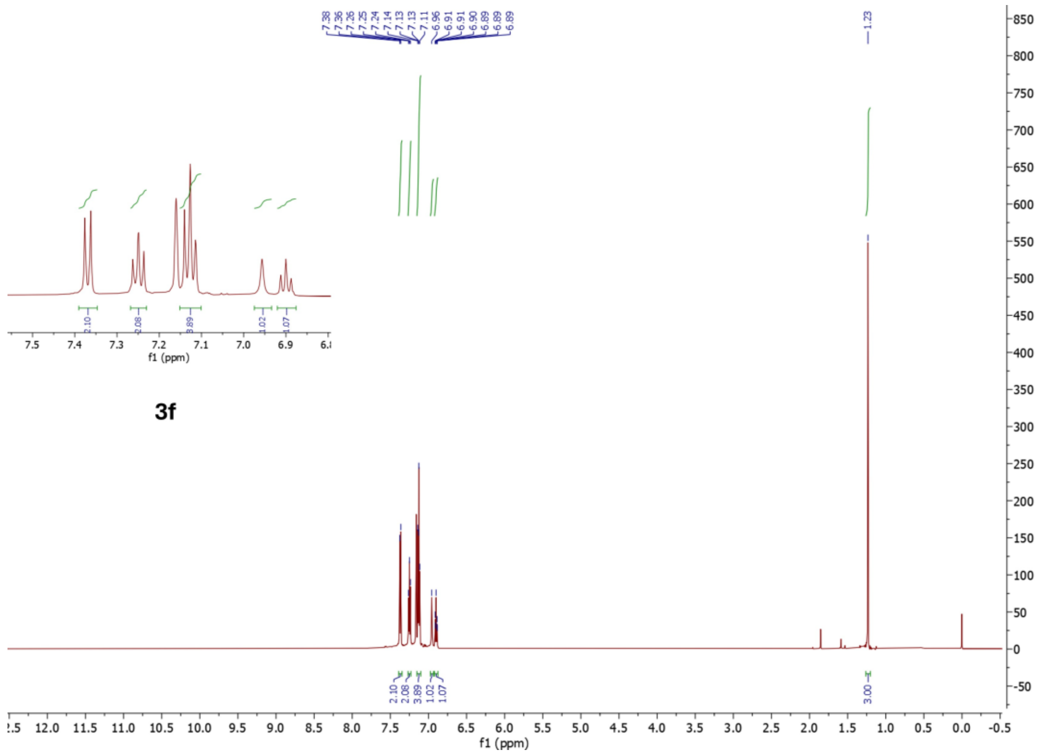

Figure S19. ^{1}H NMR (600 MHz, C_6D_6) of (*E*)-4-(1-(2-phenylhydrazono)ethyl)benzonitrile **3f**.

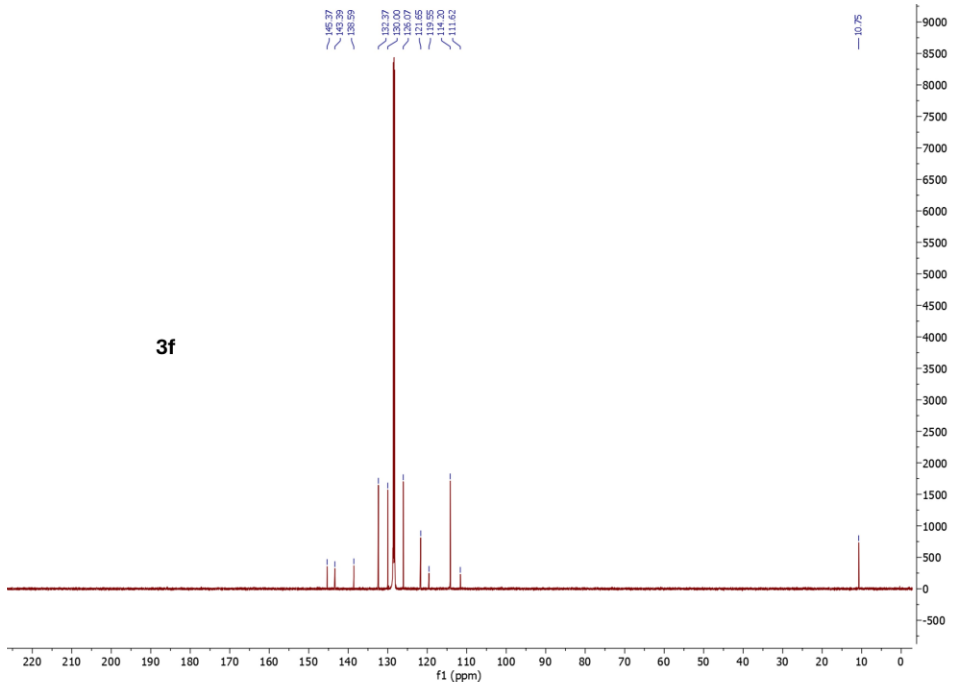

Figure S20. ^{13}C NMR (150 MHz, C_6D_6) of (*E*)-4-(1-(2-phenylhydrazono)ethyl)benzonitrile **3f**.

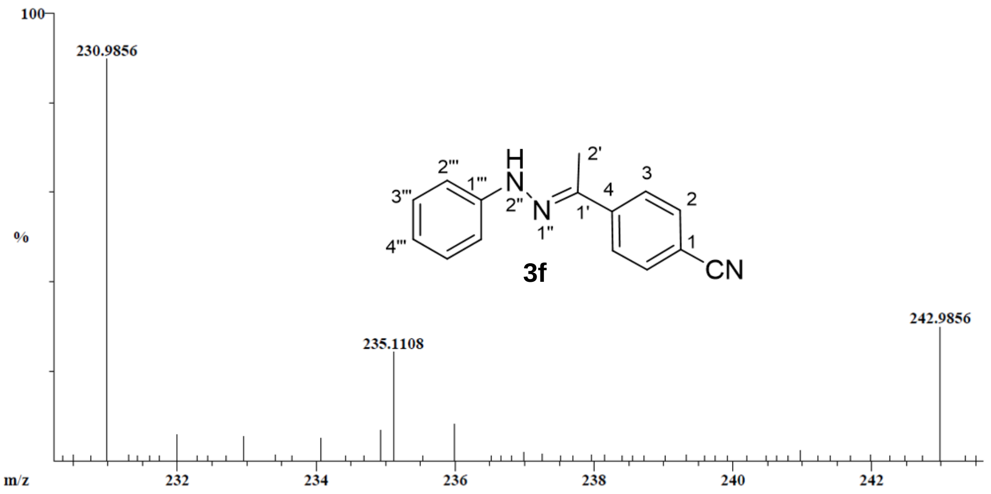

Selected Isotopes : $H_{0-13}C_{0-15}N_{0-3}$ | Error Limit : 5 ppm | Unsaturation Limits : 0 to 50

Measured Mass	*% Base*	*Formula*	*Calculated Mass*	*Error*	*Unsaturation*
235.1108	24.3%	$C_{15}H_{13}N_3$	235.1109	-0.6	11.0

Figure S21. HRMS of (*E*)-4-(1-(2-phenylhydrazono)ethyl)benzonitrile **3f**.

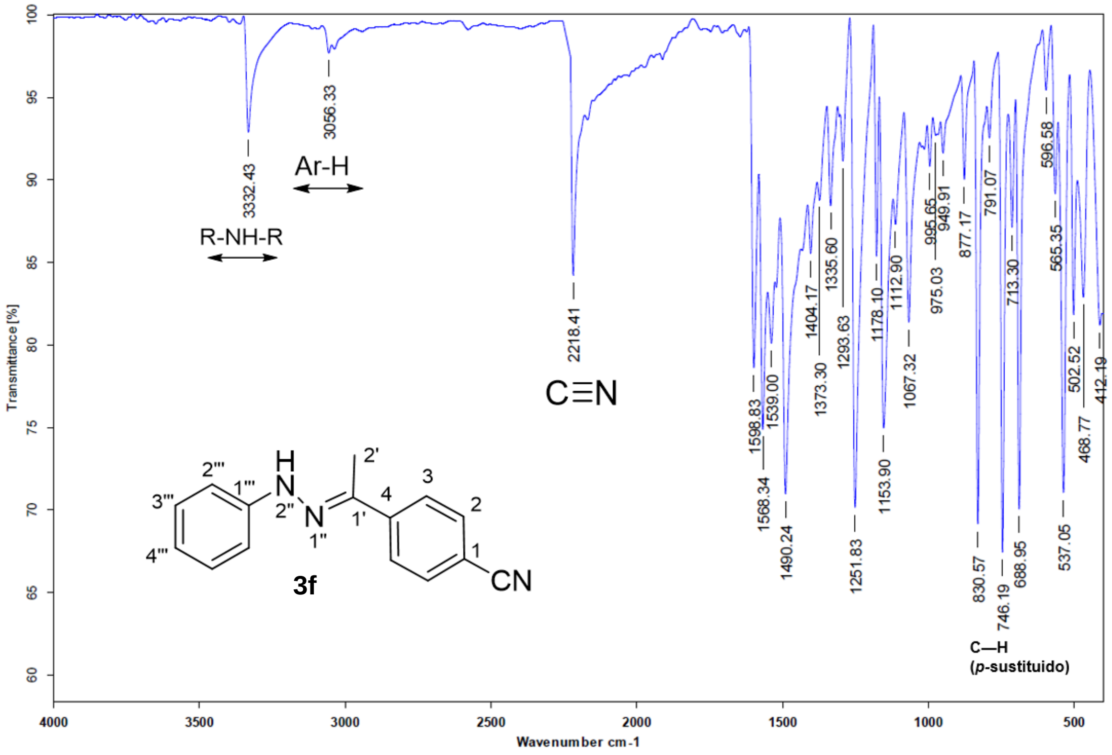

Figure S22. FT-IR of (*E*)-4-(1-(2-phenylhydrazono)ethyl)benzonitrile **3f**.

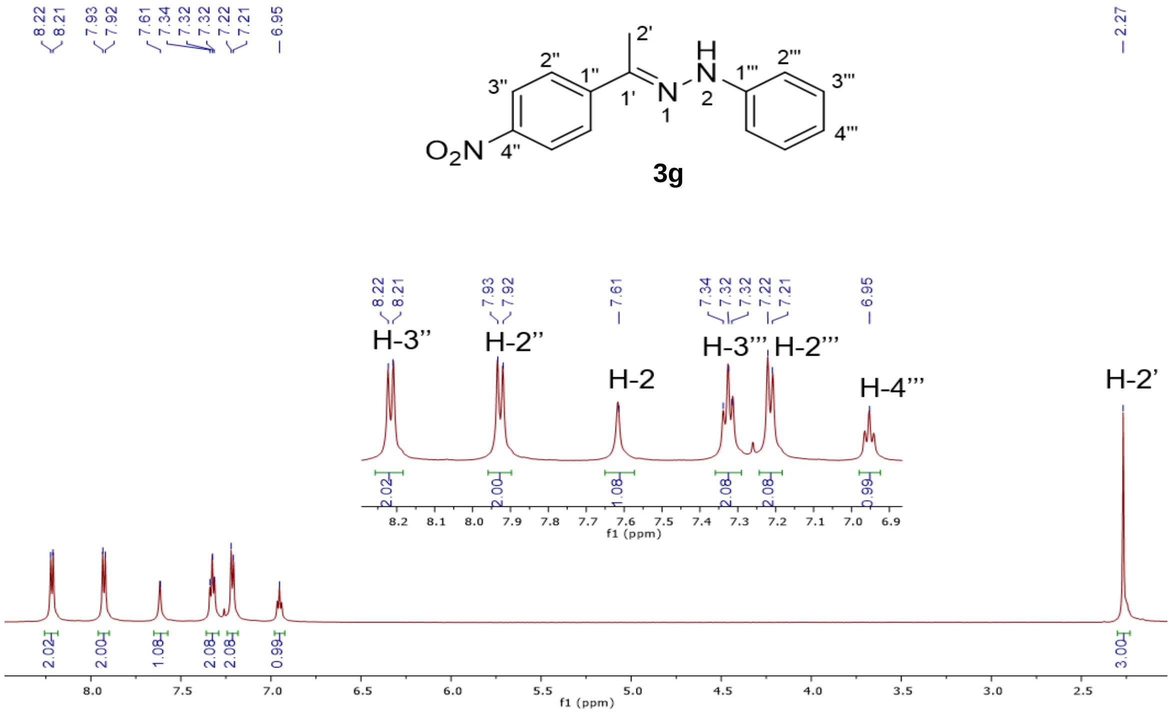

Figure S23. ^{1}H NMR (500 MHz, $CDCl_3$) of (*E*)-1-(1-(4-nitrophenyl)ethylidene)-2-phenylhydrazone **3g**.

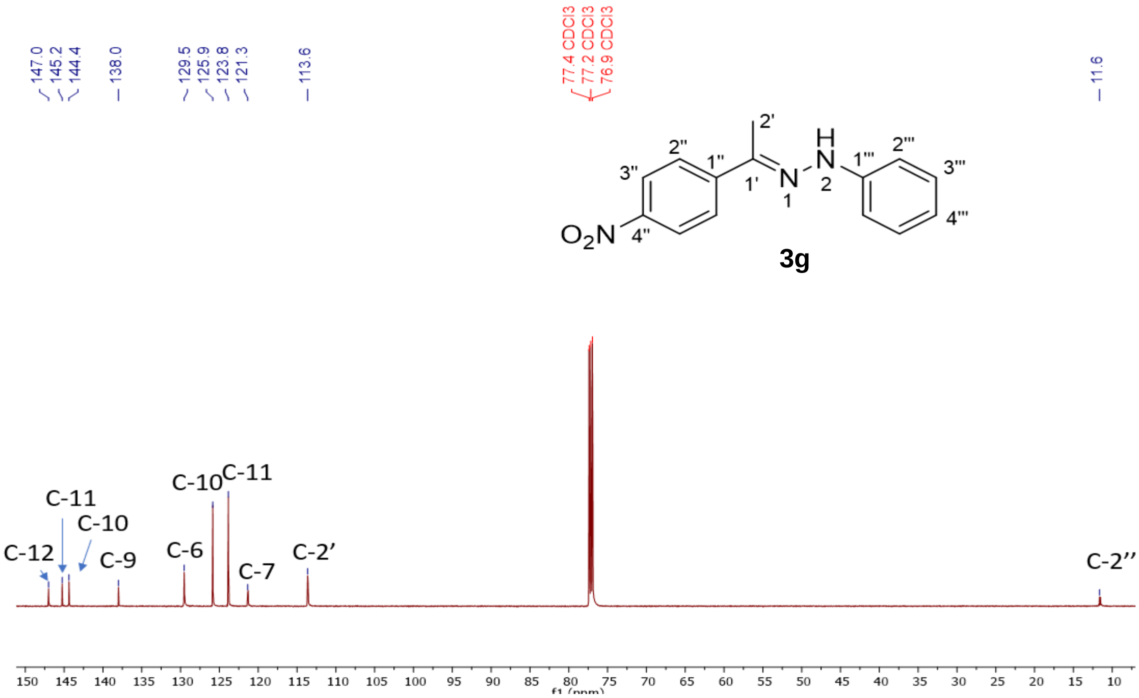

Figure S24. ^{13}C NMR (125 MHz, $CDCl_3$) of (*E*)-1-(1-(4-nitrophenyl)ethylidene)-2-phenylhydrazone **3g**.

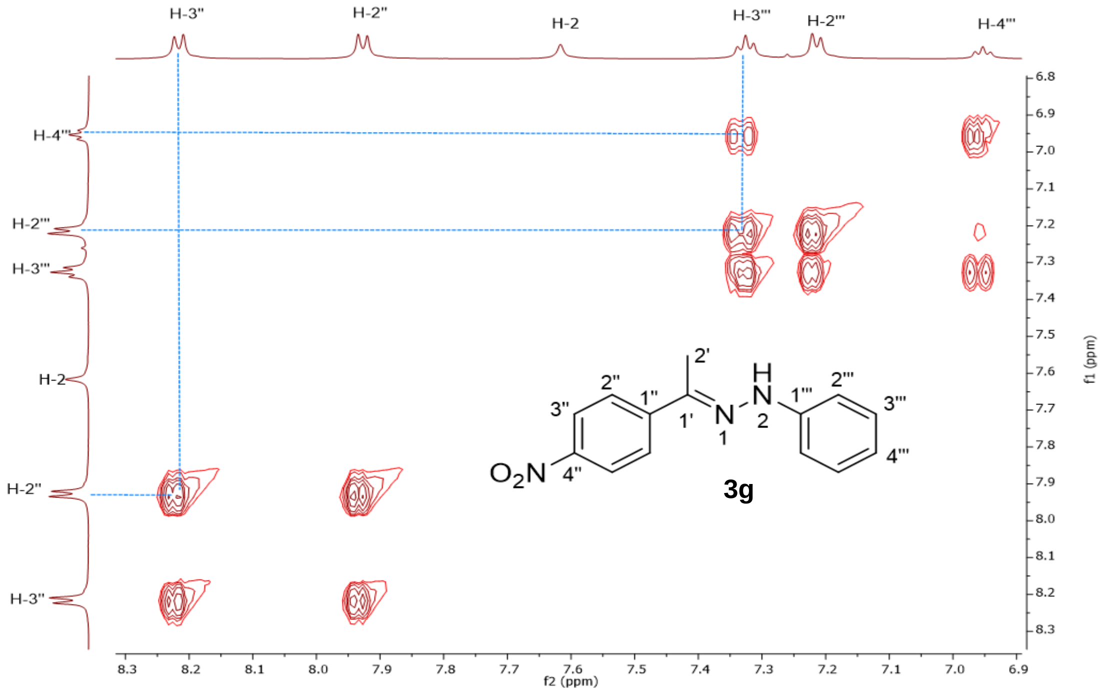

Figure S25. COSY experiment of (*E*)-1-(1-(4-nitrophenyl)ethylidene)-2-phenylhydrazone **3g**.

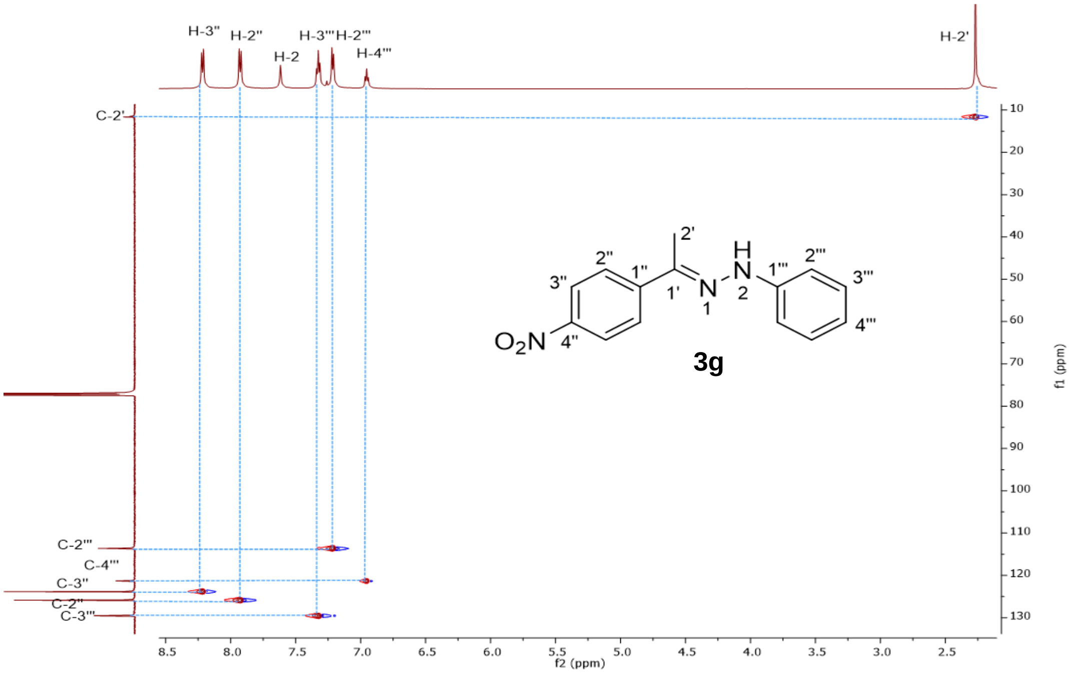

|**Figure S26**. HSQC experiment of (*E*)-1-(1-(4-nitrophenyl)ethylidene)-2-phenylhydrazone **3g**.

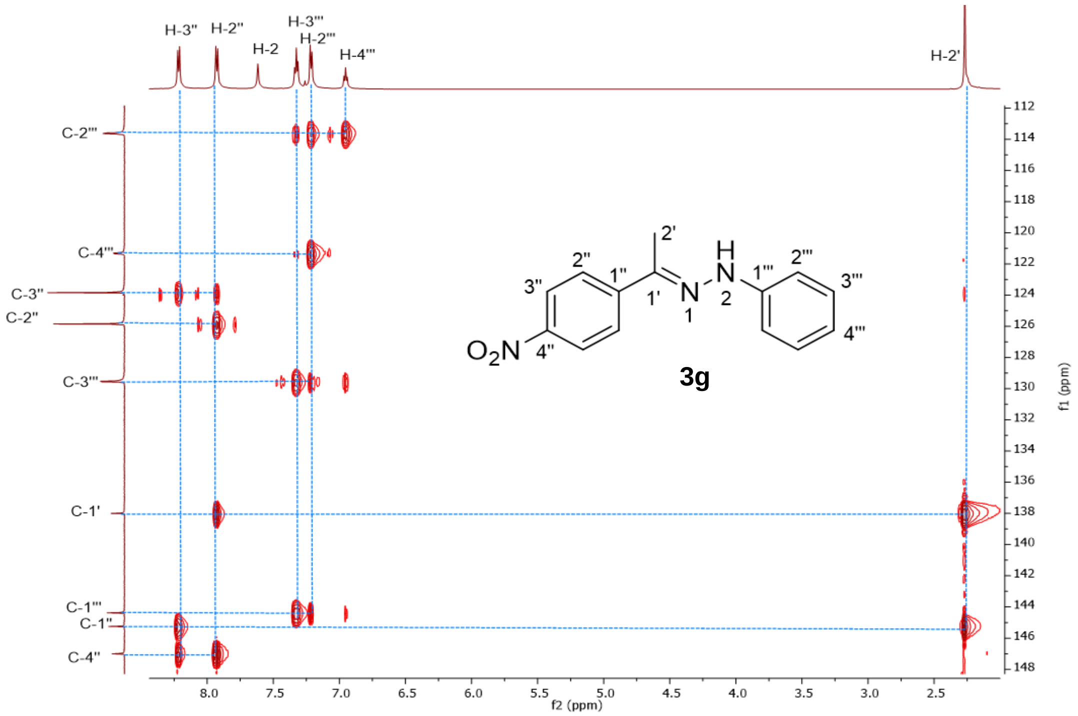

Figure S27. HMBC experiment of (*E*)-1-(1-(4-nitrophenyl)ethylidene)-2-phenylhydrazone **3g**.

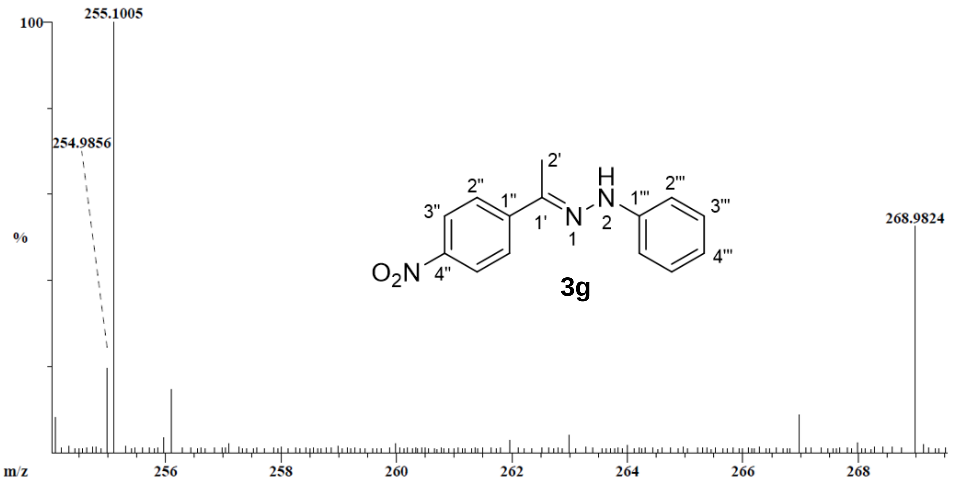

Selected Isotopes : $H_{0\text{-}13}C_{0\text{-}14}N_{0\text{-}3}O_{0\text{-}2}$ Error Limit : 5 ppm Unsaturation Limits : 0 to 50

Measured Mass	% Base	Formula	Calculated Mass	Error	Unsaturation
255.1005	100.0%	$C_{14}H_{13}N_3O_2$	255.1008	-1.1	10.0

Figure S28. HRMS of (*E*)-1-(1-(4-nitrophenyl)ethylidene)-2-phenylhydrazone **3g**.

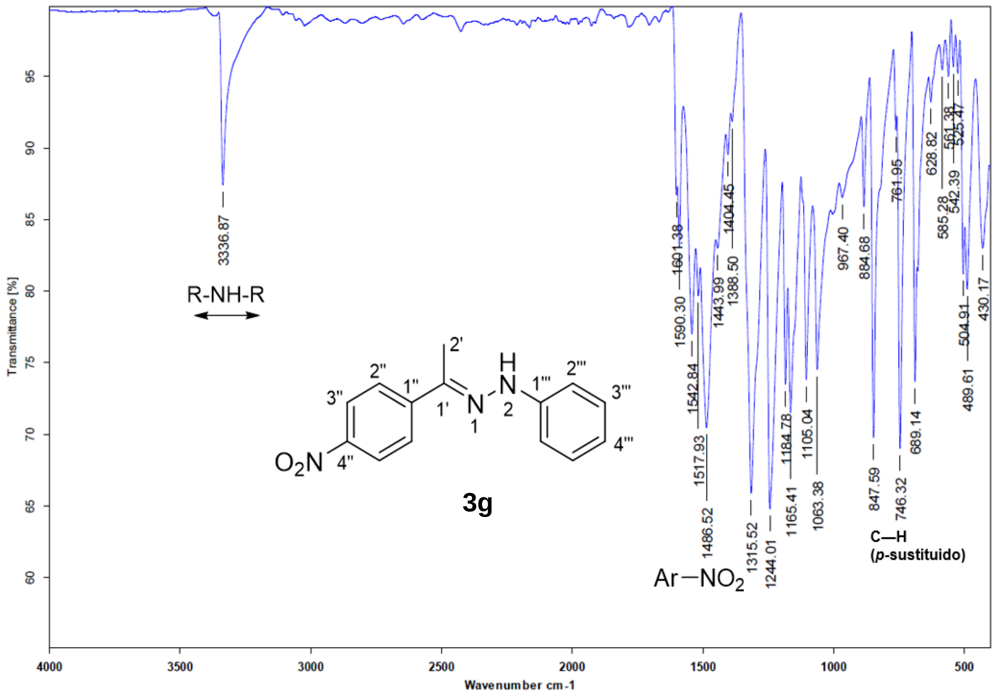

Figure S29. FT-IR of (*E*)-1-(1-(4-nitrophenyl)ethylidene)-2-phenylhydrazone **3g**.

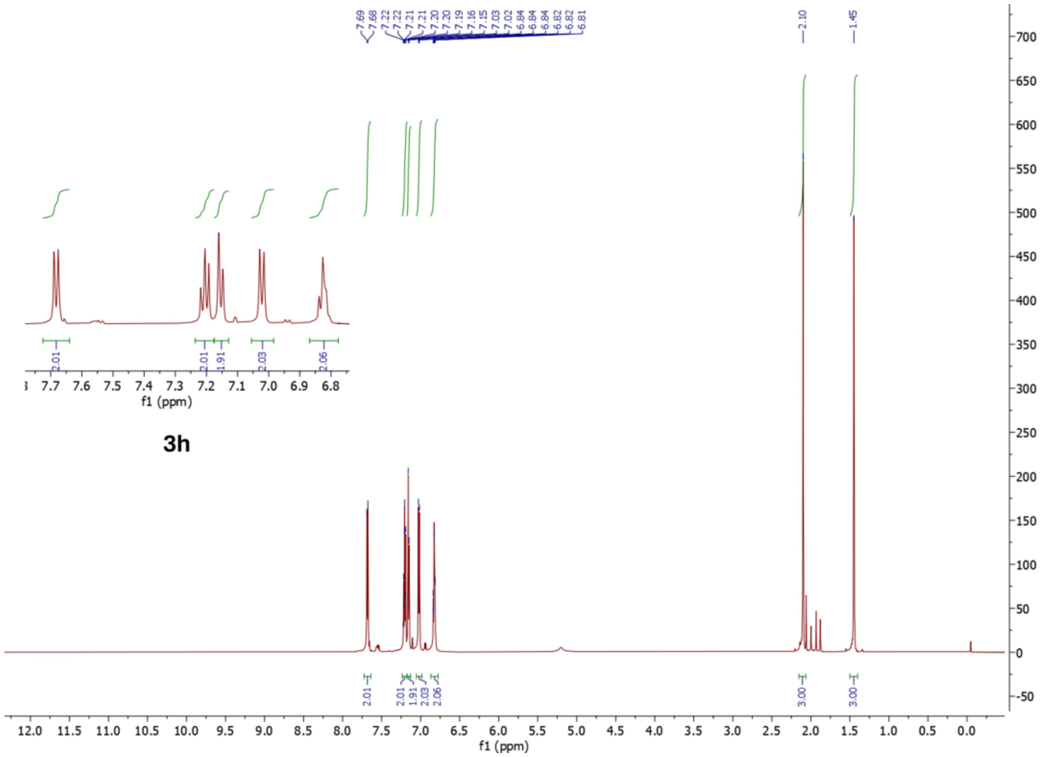

Figure S30. ^{1}H NMR (600 MHz, CD_6) of (*E*)-1-phenyl-2-(1-*p*-tolyl)ethylidene)hydrazone **3h**

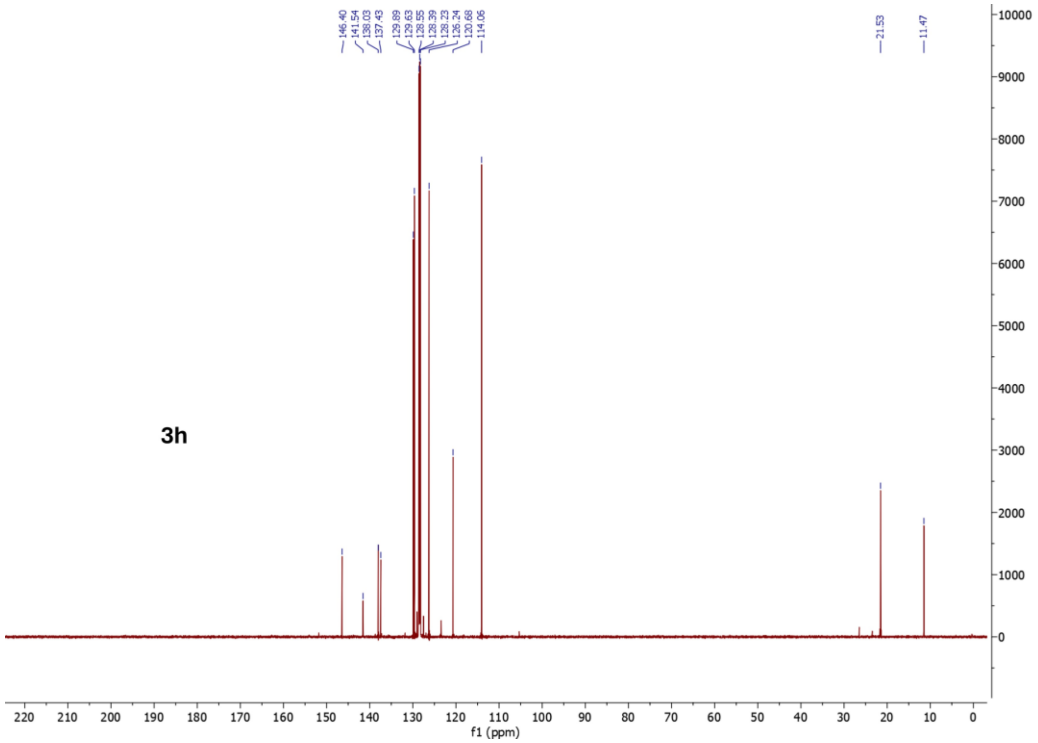

Figure S31. ^{13}C NMR (150 MHz, CD_6) of (*E*)-1-phenyl-2-(1-*p*-tolyl)ethylidene)hydrazone **3h**

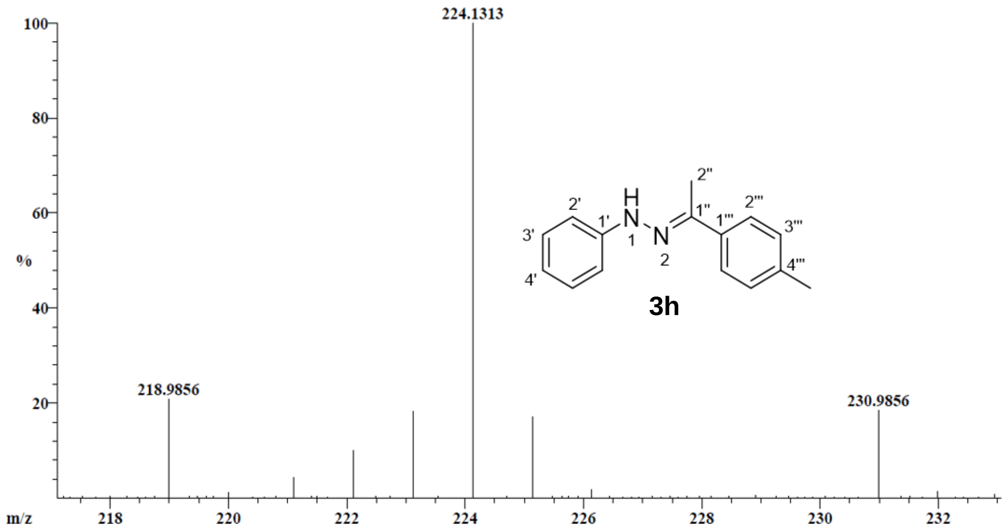

Selected Isotopes : $H_{0\text{-}16}C_{0\text{-}15}N_{0\text{-}2}$ Error Limit : 5 ppm   Unsaturation Limits : 0 to 50

Measured Mass	% Base	Formula	Calculated Mass	Error	Unsaturation
224.1313	100.0%	$C_{15}H_{16}N_2$	224.1314	-0.2	9.0

Figure S32. HRMS of (*E*)-1-phenyl-2-(1-*p*-tolyl)ethylidene)hydrazone **3h**.

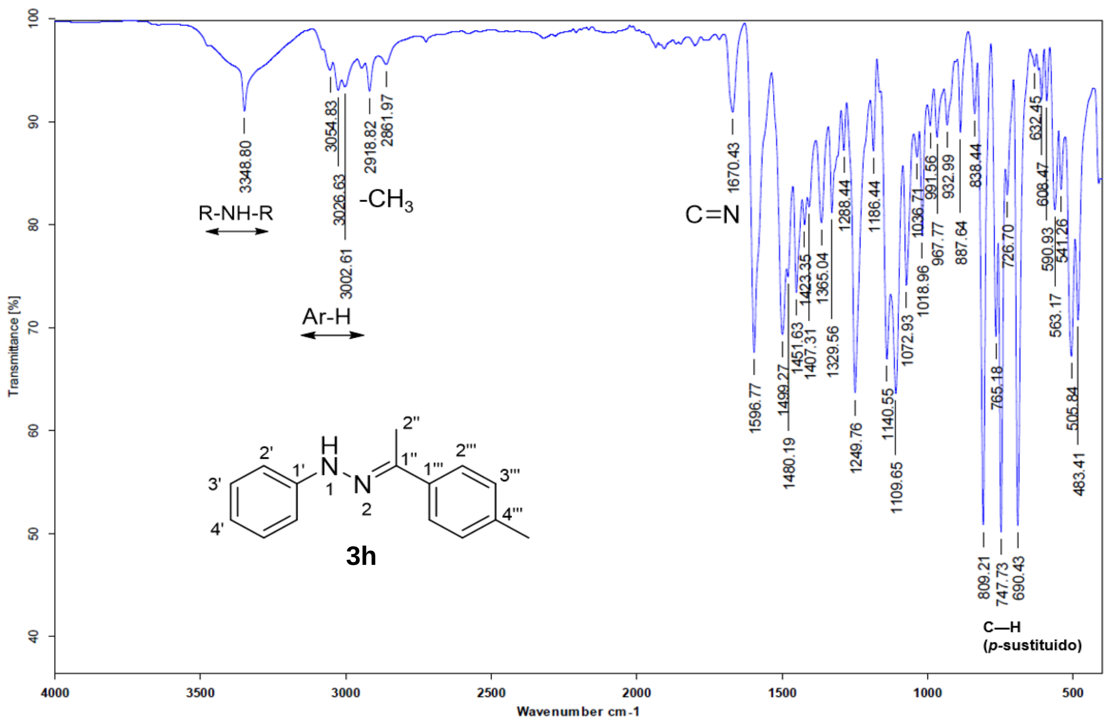

Figure S33. FT-IR of (*E*)-1-phenyl-2-(1-*p*-tolyl)ethylidene)hydrazone **3h**.

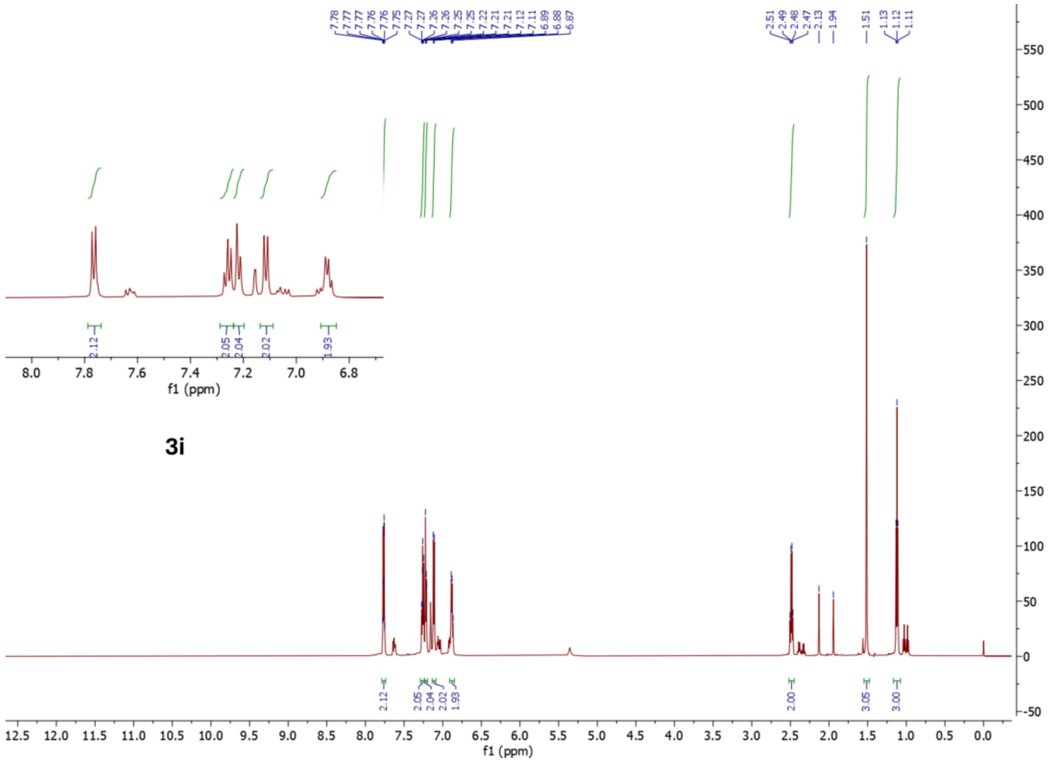

Figure S34. ^{1}H NMR (600 MHz, CD_6) of (*E*)-1-(1-(4-ethylphenyl)ethylidene)-2-phenylhydrazone **3i**.

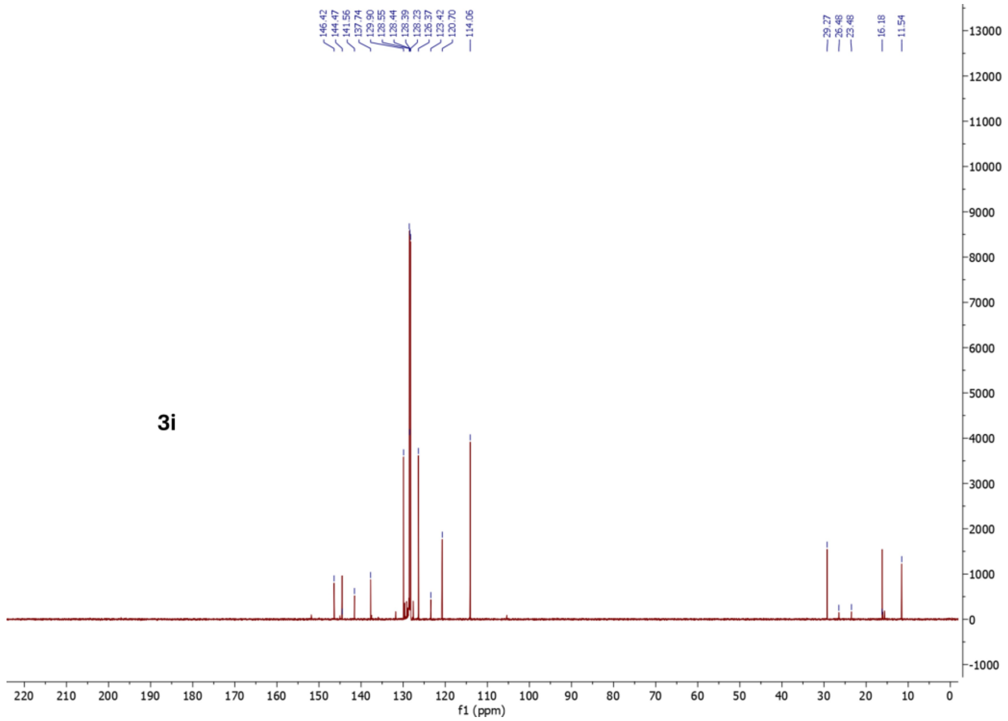

Figure S35. ^{13}C NMR (150 MHz, CD_6) of (*E*)-1-(1-(4-ethylphenyl)ethylidene)-2-phenylhydrazone **3i**.

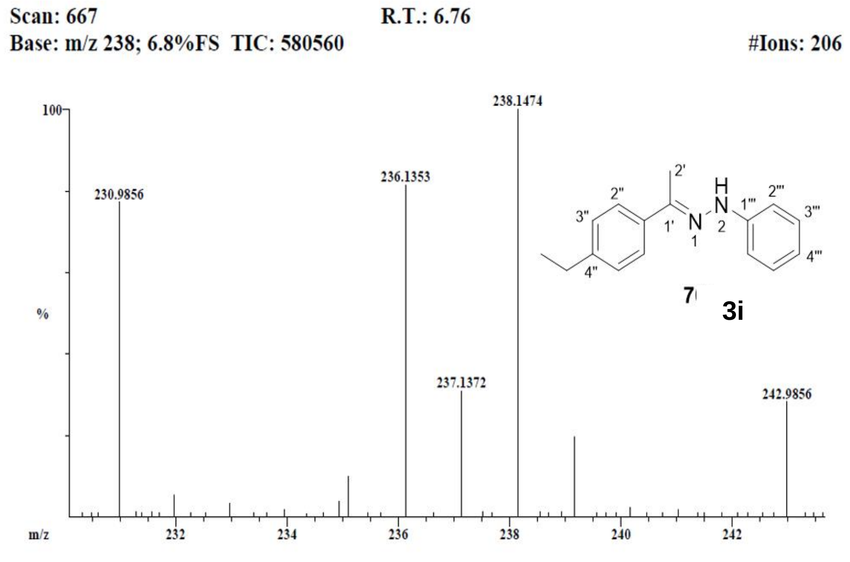

Selected Isotopes : $H_{0-18}C_{0-16}N_{0-2}$ Error Limit : 5 ppm Unsaturation Limits : 0 to 50

Measured Mass	% Base	Formula	Calculated Mass	Error	Unsaturation
238.1474	100.0%	$C_{16}H_{18}N_2$	238.1470	1.7	9.0

Figure S36. HRMS of (*E*)-1-(1-(4-ethylphenyl)ethylidene)-2-phenylhydrazone **3i**.

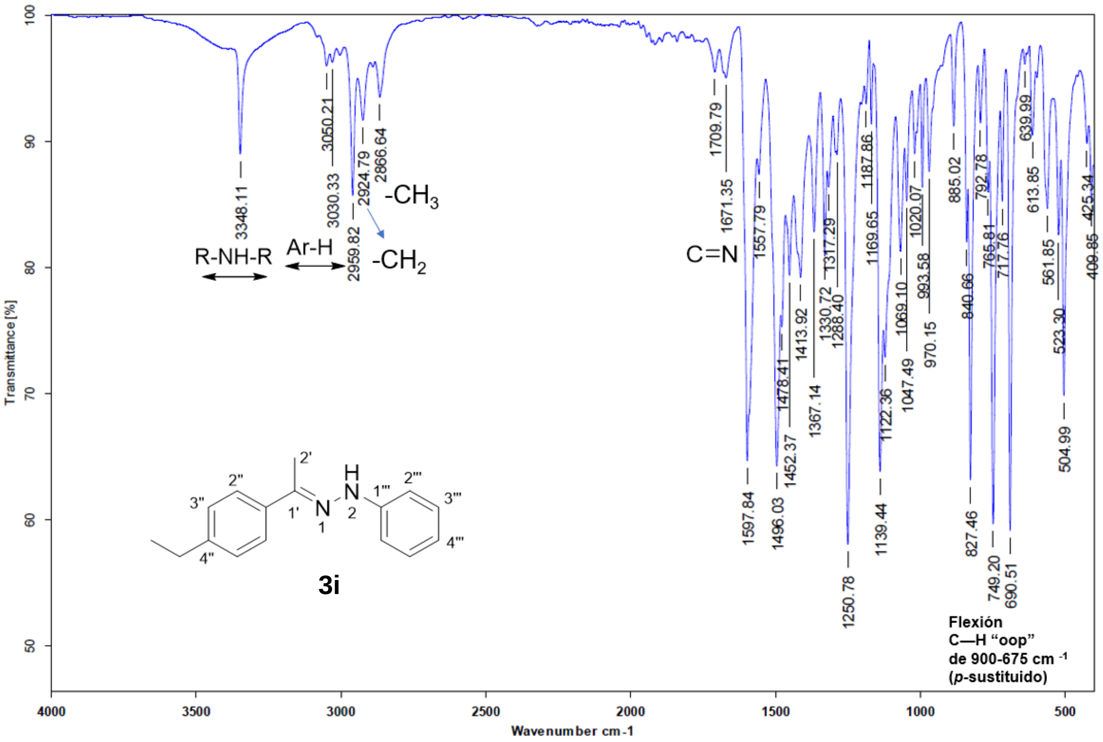

Figure S37. FT-IR of (*E*)-1-(1-(4-ethylphenyl)ethylidene)-2-phenylhydrazone **3i**.

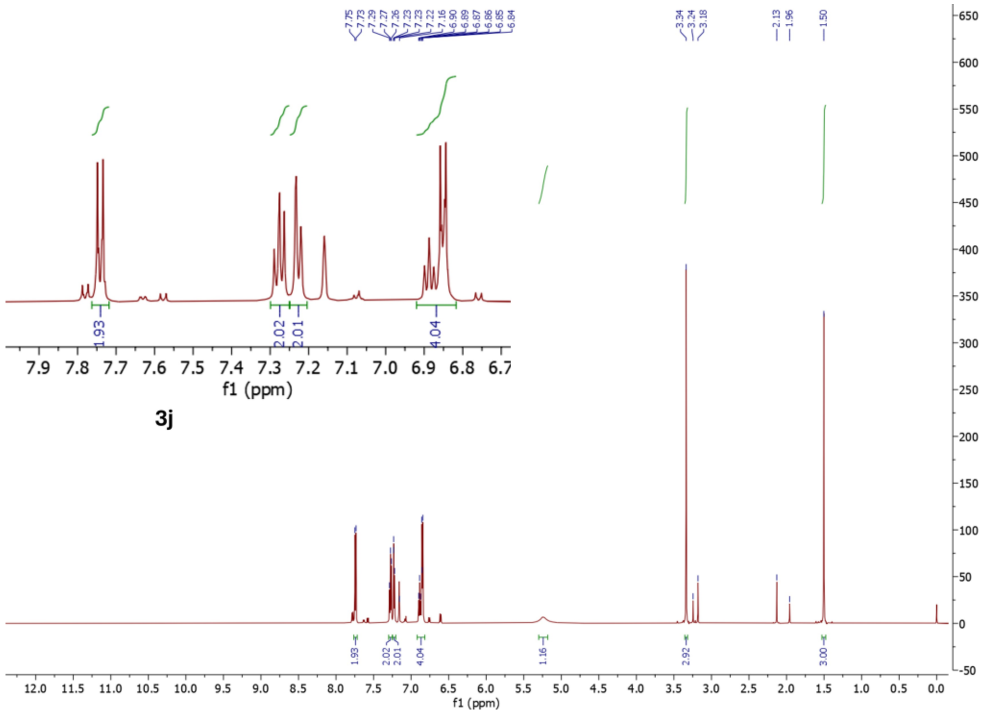

Figure S38. ^{1}H NMR (600 MHz, CD_6) of (*E*)-1-(1-(4-methoxyphenyl)ethylidene)-2-phenylhydrazone **3j**.

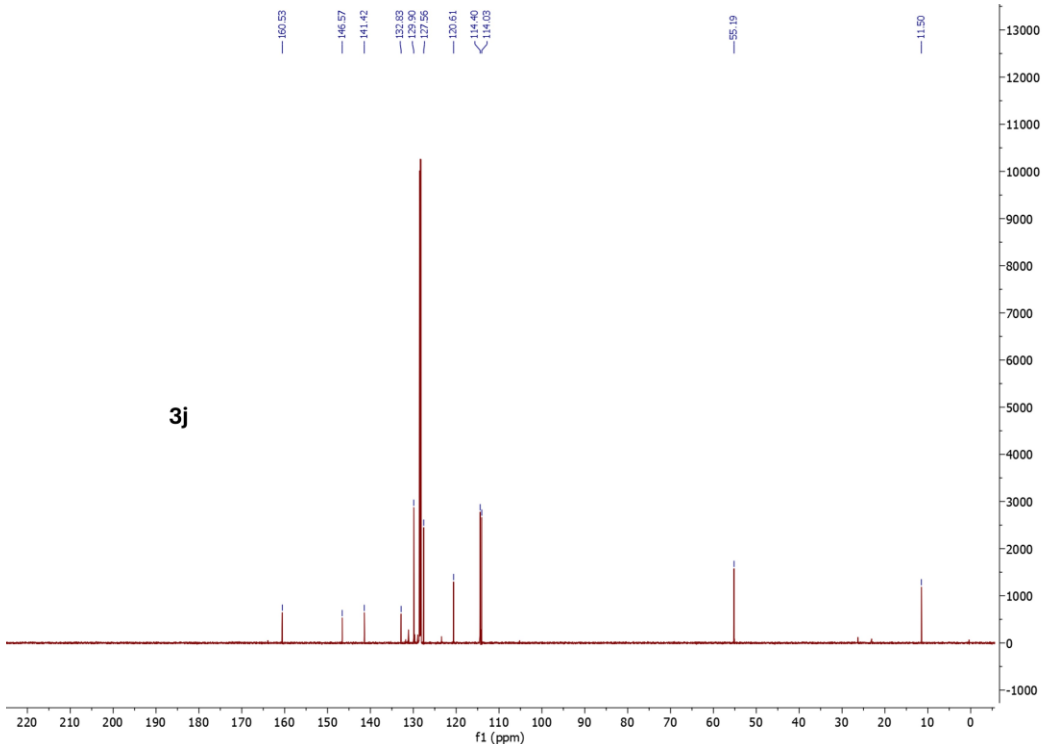

Figure S39. ^{13}C NMR (150 MHz, CD_6) of (*E*)-1-(1-(4-methoxyphenyl)ethylidene)-2-phenylhydrazone **3j**.

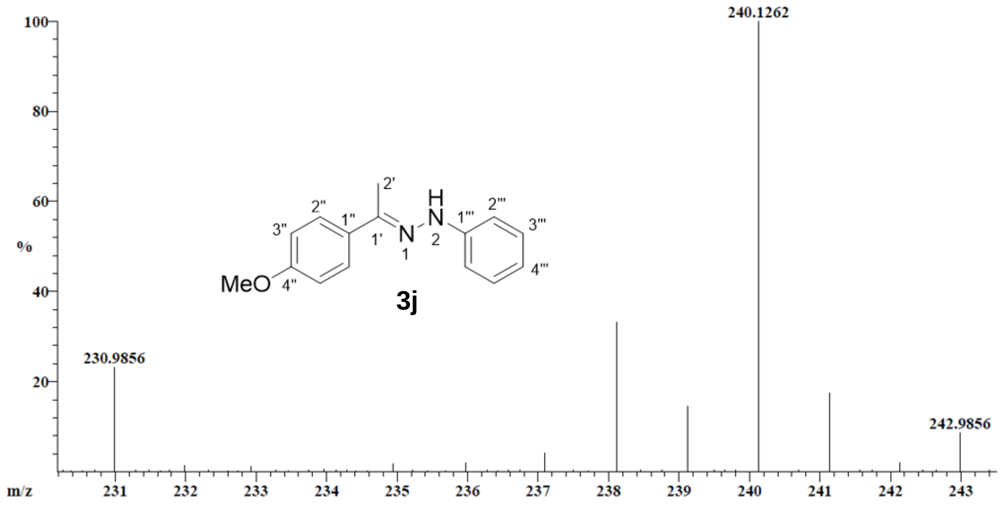

Selected Isotopes : $H_{0-16}C_{0-15}N_{0-2}O_{0-1}$ Error Limit : 5 ppm Unsaturation Limits : 0 to 50

Measured Mass	*% Base*	*Formula*	*Calculated Mass*	*Error*	*Unsaturation*
240.1262	100.0%	$C_{15}H_{16}N_2O$	240.1263	-0.3	9.0

Figure S40. HRMS of (*E*)-1-(1-(4-methoxyphenyl)ethylidene)-2-phenylhydrazone **3j**.

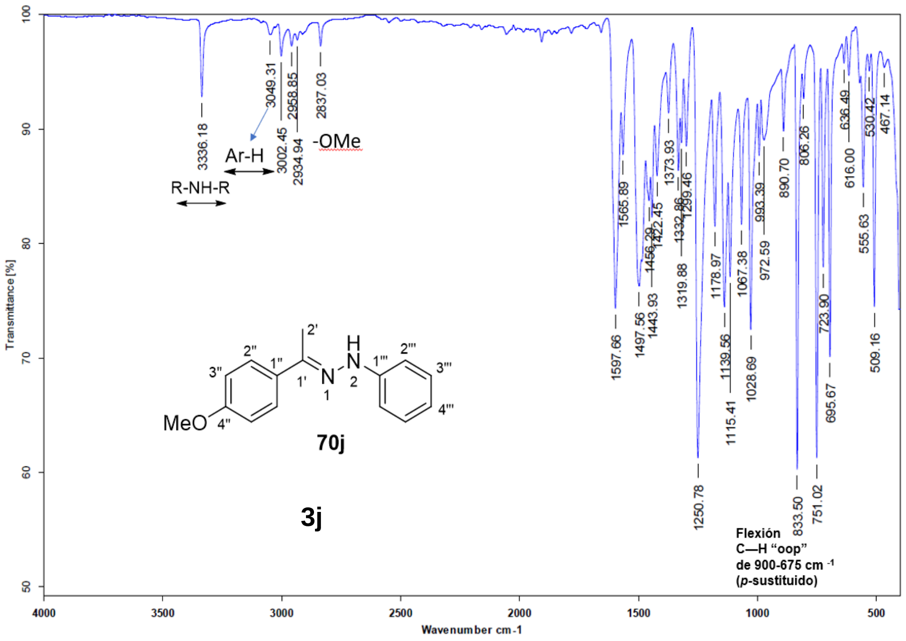

Figure S41. FT-IR of (*E*)-1-(1-(4-methoxyphenyl)ethylidene)-2-phenylhydrazone **3j**.

Characterization of series **4a-j**

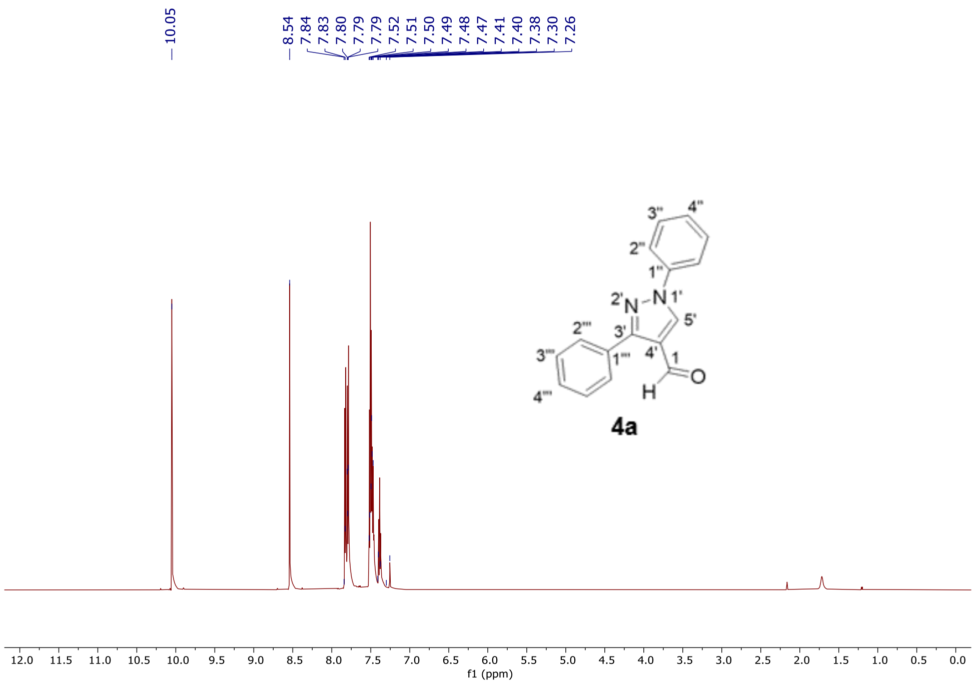

Figure S42. ^{1}H NMR (500 MHz, $CDCl_3$) of 1,3-diphenyl-1*H*-pyrazole-4-carbaldehyde **4a**.

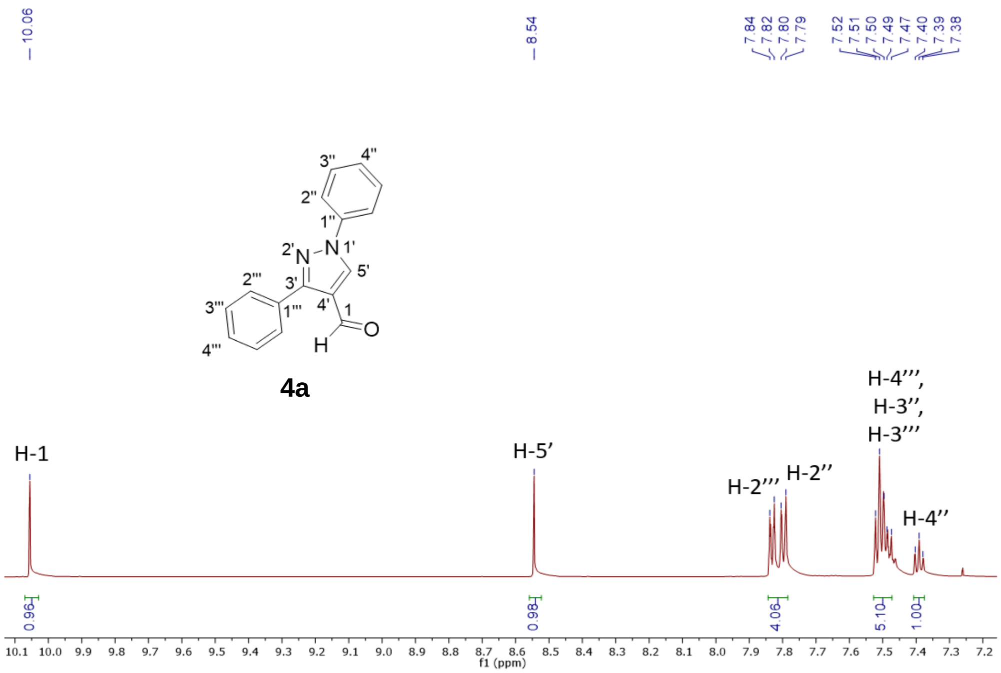

Figure S43. Expansion of ^{1}H NMR (500 MHz, $CDCl_3$) of 1,3-diphenyl-1*H*-pyrazole-4-carbaldehyde **4a**.

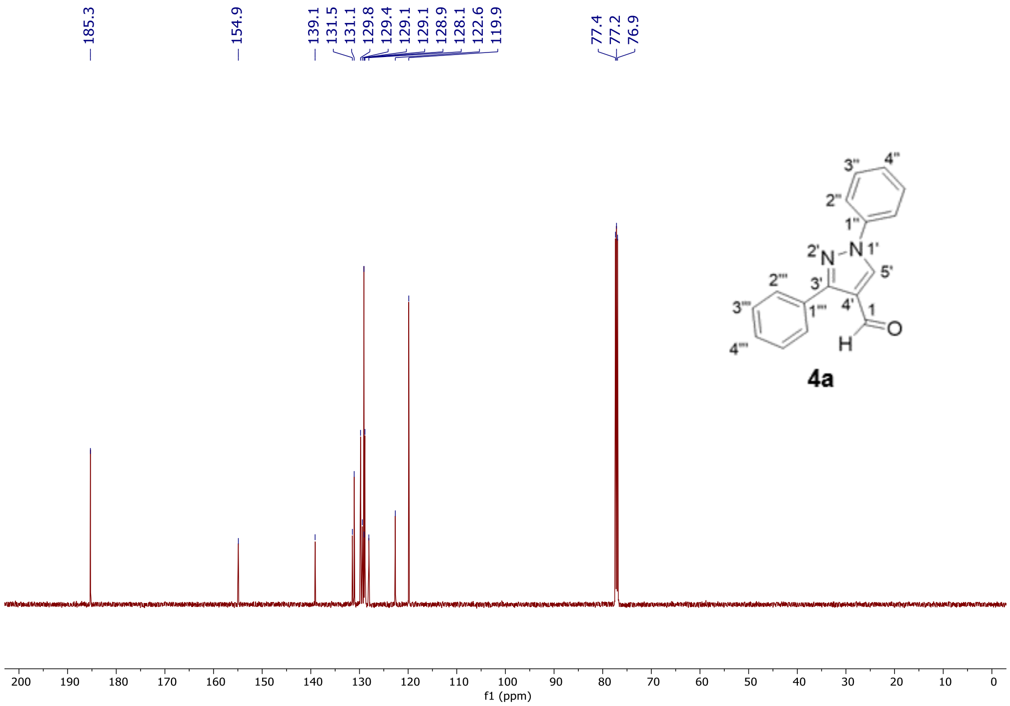

Figure S44. ^{13}C NMR (125 MHz, $CDCl_3$) of 1,3-diphenyl-1*H*-pyrazole-4-carbaldehyde **4a**.

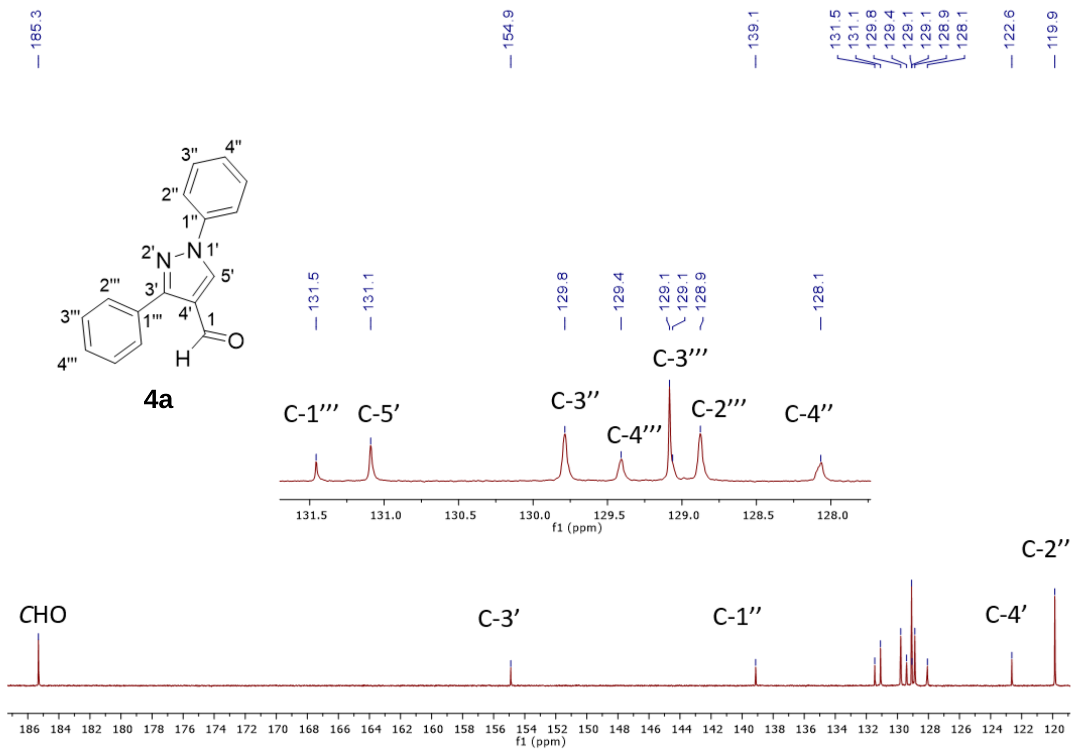

Figure S45. Expansion of ^{13}C NMR (125 MHz, $CDCl_3$) of 1,3-diphenyl-1*H*-pyrazole-4-carbaldehyde **4a**.

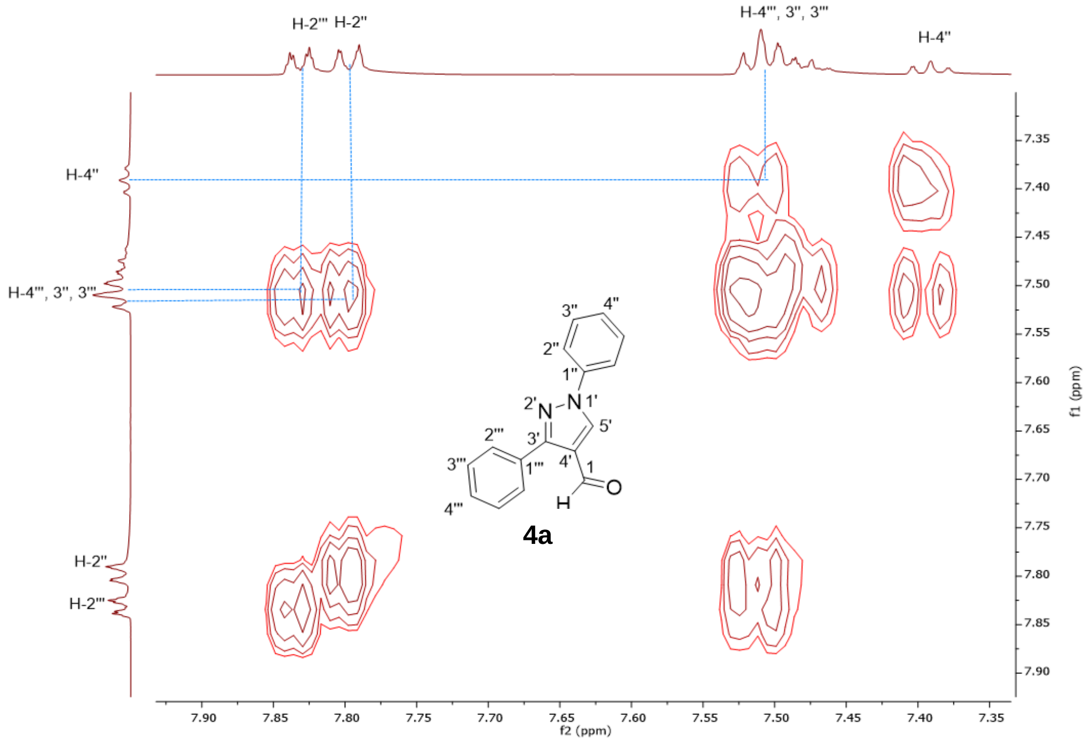

Figure S46. COSY experiment of 1,3-diphenyl-1*H*-pyrazole-4-carbaldehyde **4a**.

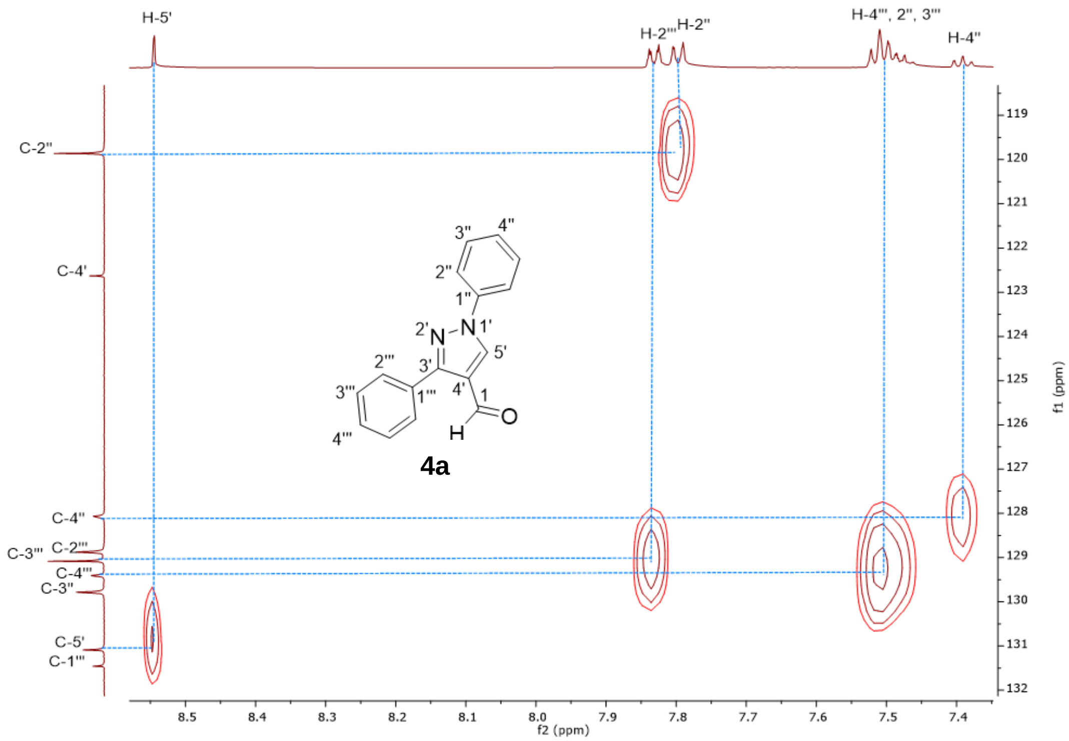

Figure S47. HSQC experiment of 1,3-diphenyl-1*H*-pyrazole-4-carbaldehyde **4a**.

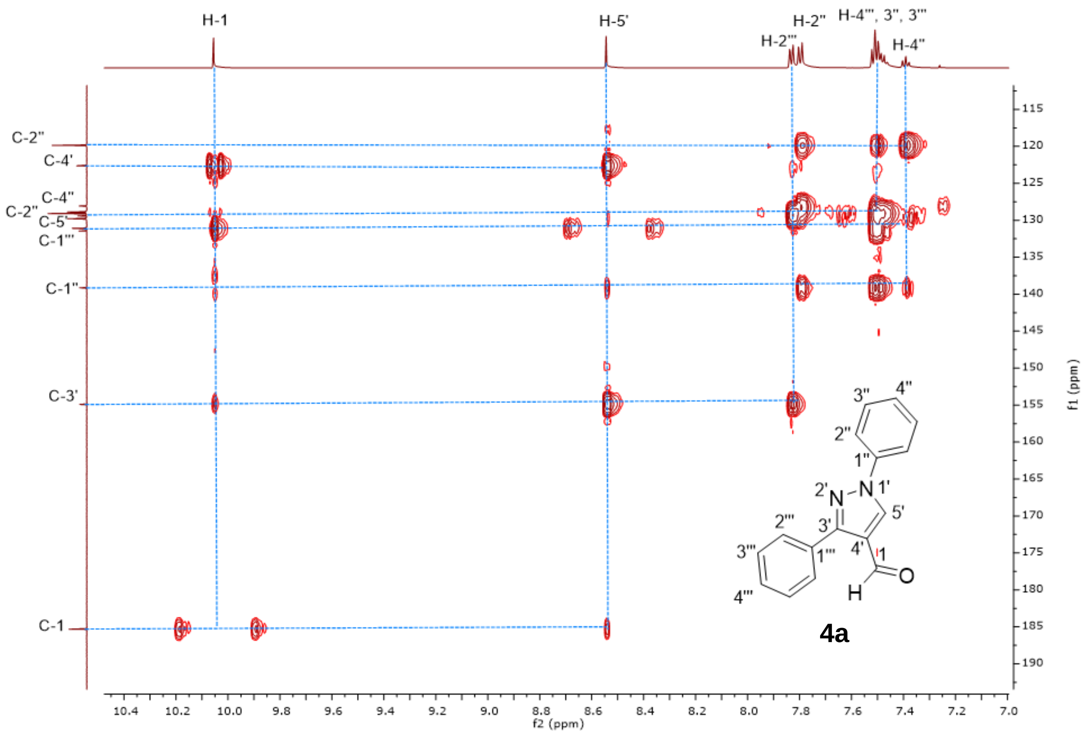

Figure S48. HMBC experiment of 1,3-diphenyl-1*H*-pyrazole-4-carbaldehyde **4a**.

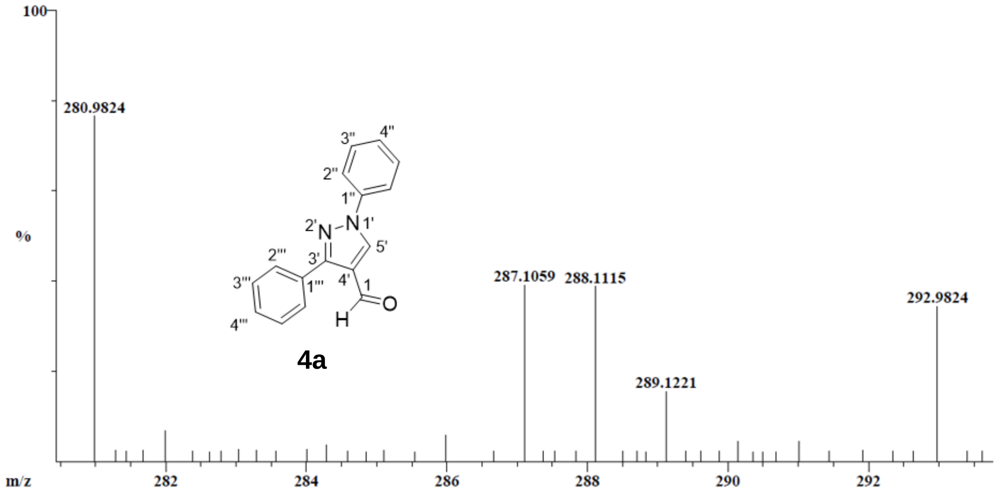

Selected Isotopes : $C_{0-18}N_{0-3}O_{0-1}H_{0-13}$ Error Limit : 5 ppm Unsaturation Limits : 0 to 50

Measured Mass	*% Base*	*Formula*	*Calculated Mass*	*Error*	*Unsaturation*
287.1059	39.1%	$C_{18}H_{13}N_3O$	287.1059	0.1	14.0

Figure S49. HRMS of 1,3-diphenyl-1*H*-pyrazole-4-carbaldehyde **4a**.

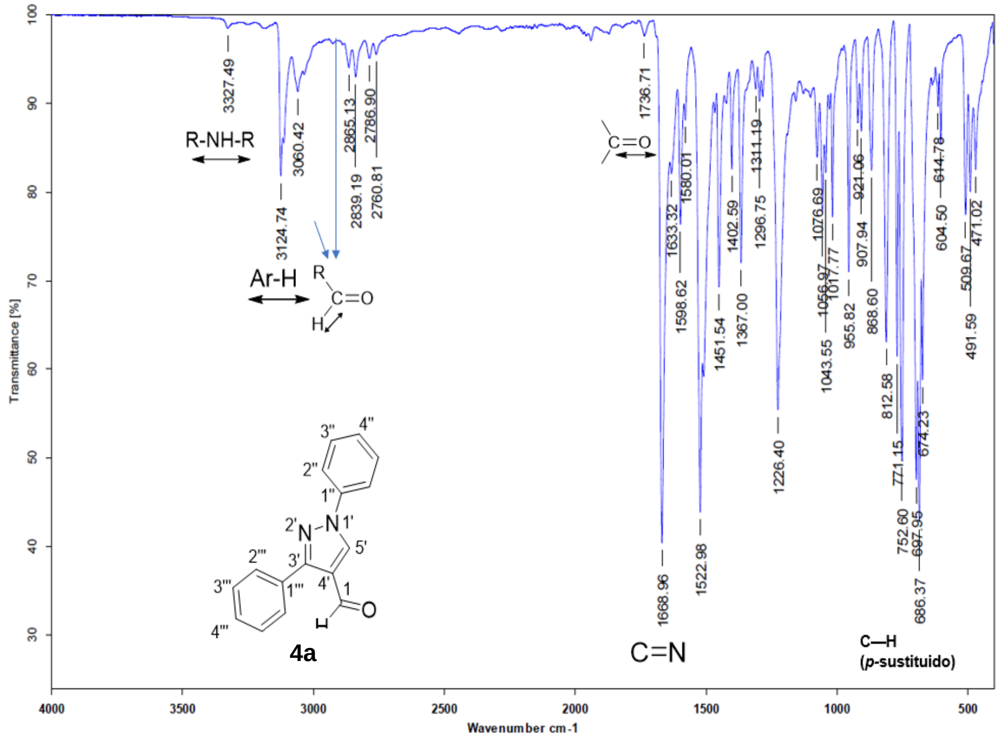

Figure S50. FT-IR of 1,3-diphenyl-1*H*-pyrazole-4-carbaldehyde **4a**.

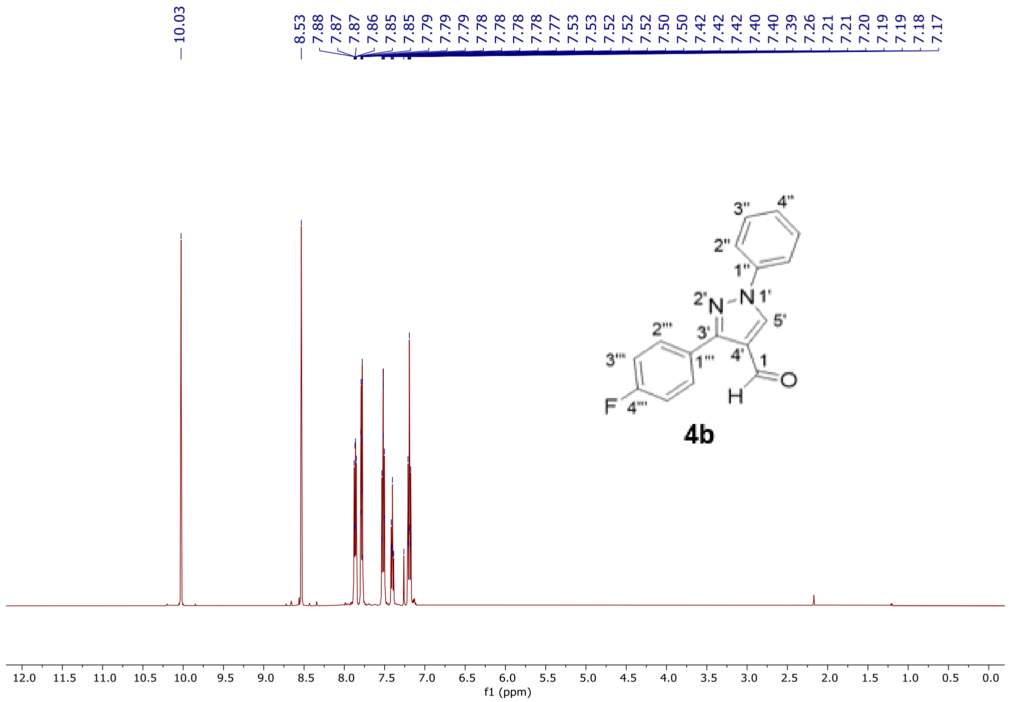

Figure S51. ^{1}H NMR (500 MHz, $CDCl_3$) of 3-(4-fluorophenyl)-1-phenyl-1*H*-pyrazole-4-carbaldehyde **4b**.

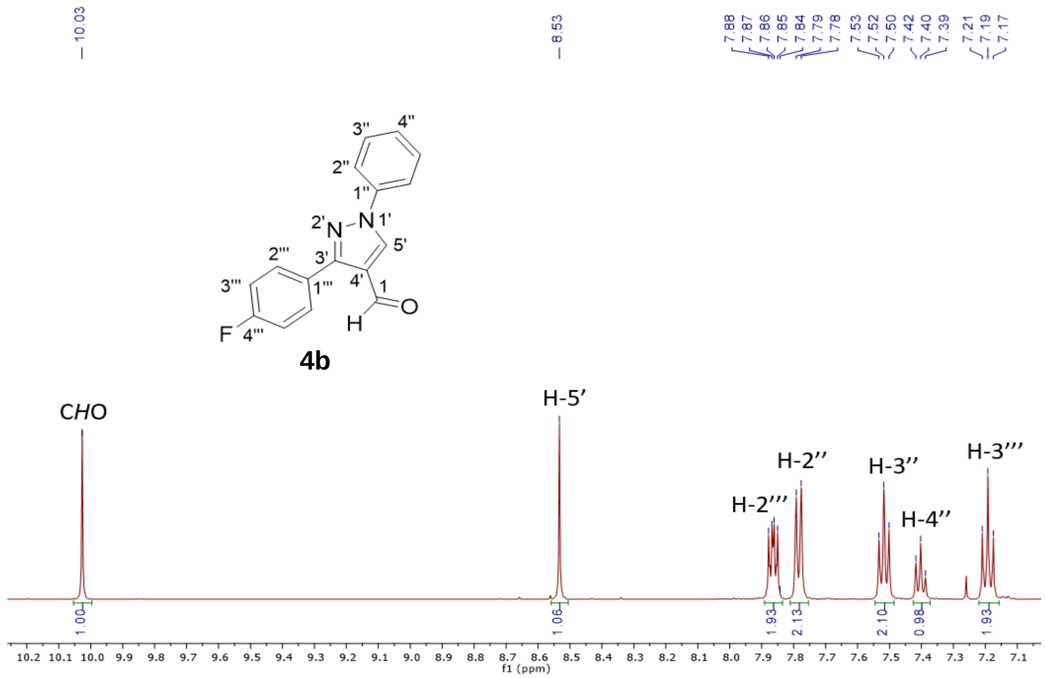

Figure S52. Expansion of ^{1}H NMR (500 MHz, $CDCl_3$) of 3-(4-fluorophenyl)-1-phenyl-1*H*-pyrazole-4-carbaldehyde **4b**.

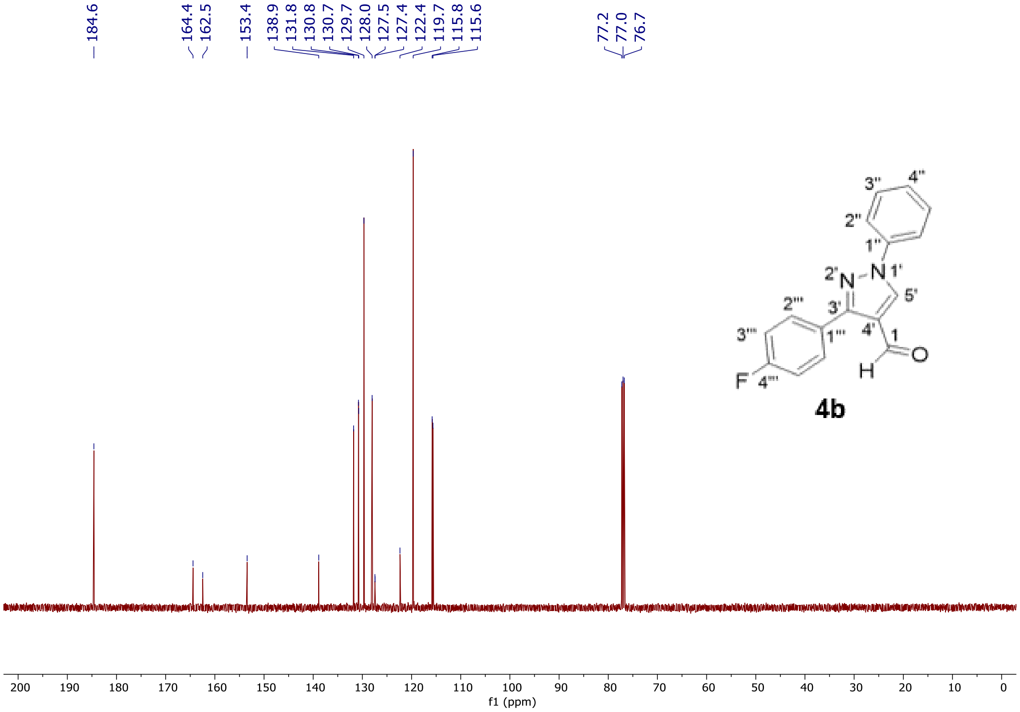

Figure S53. ^{13}C NMR (125 MHz, $CDCl_3$) of 3-(4-fluorophenyl)-1-phenyl-1*H*-pyrazole-4-carbaldehyde **4b**.

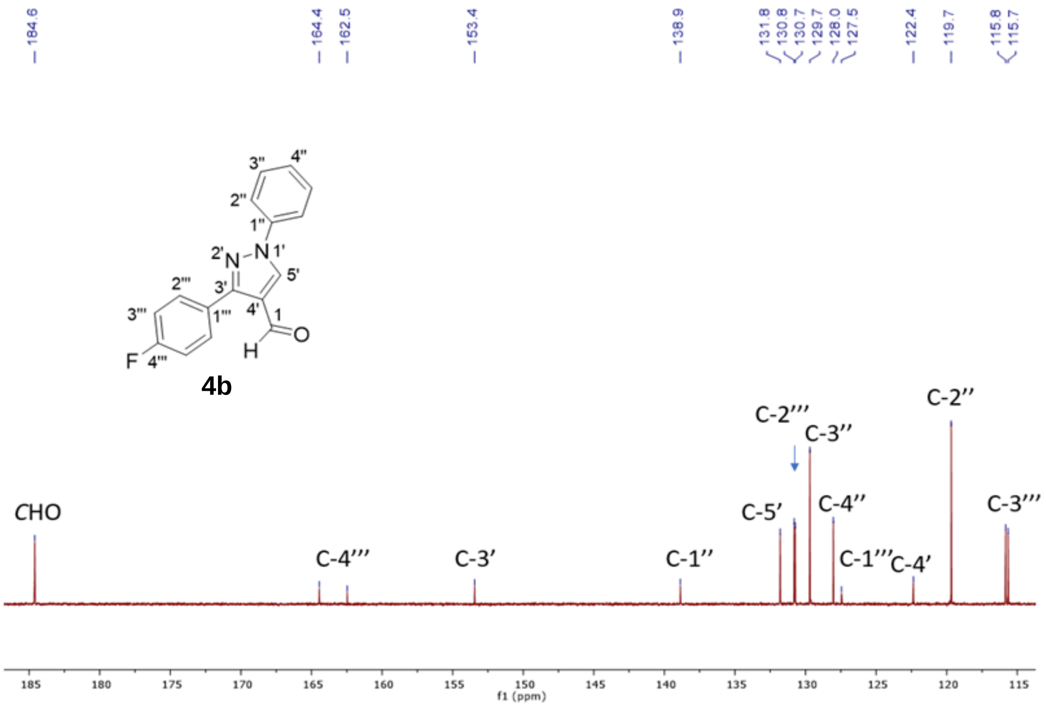

Figure S54. Expansion of ^{13}C NMR (125 MHz, $CDCl_3$) of 3-(4-fluorophenyl)-1-phenyl-1*H*-pyrazole-4-carbaldehyde **4b**.

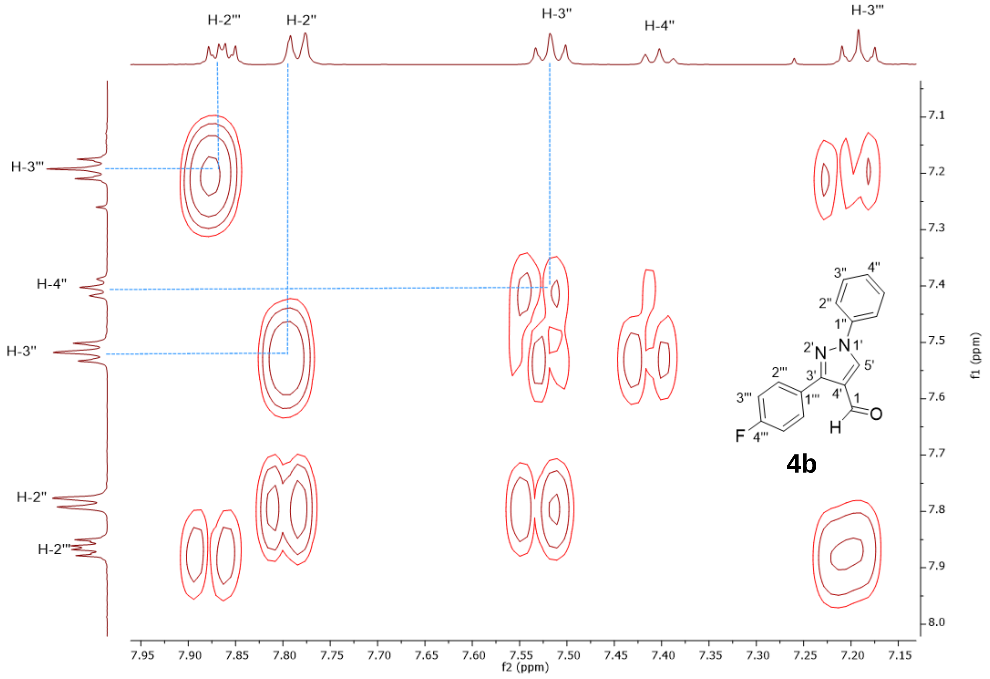

Figure S55. COSY experiment of 3-(4-fluorophenyl)-1-phenyl-1*H*-pyrazole-4-carbaldehyde **4b**.

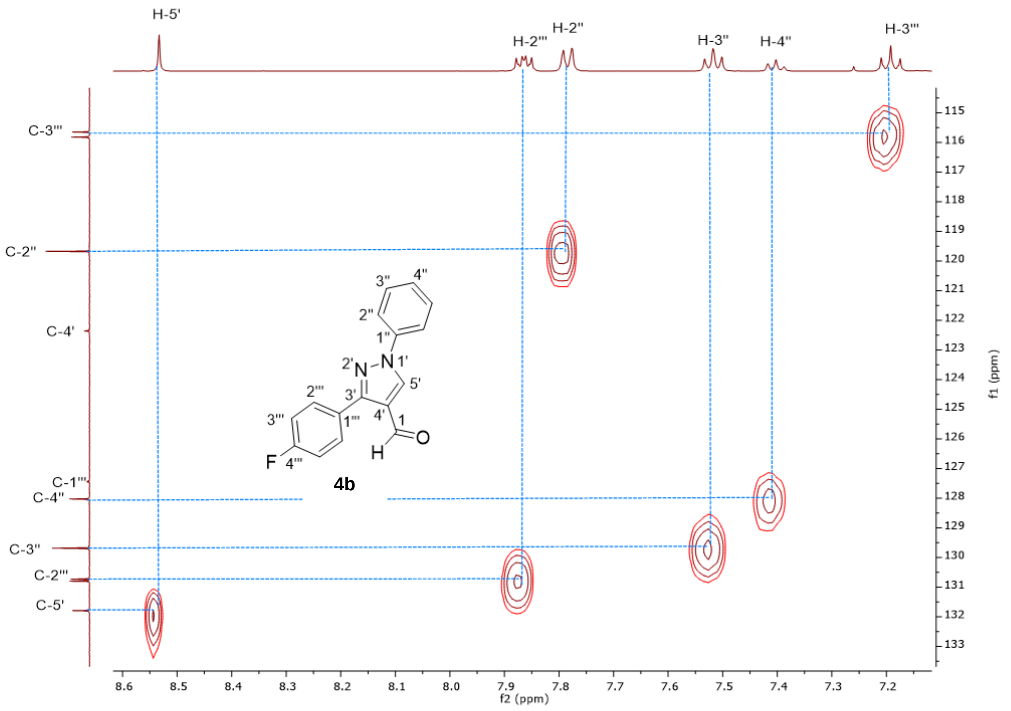

Figure S56. Expansion of HSQC experiment of 3-(4-fluorophenyl)-1-phenyl-1*H*-pyrazole-4-carbaldehyde **4b**.

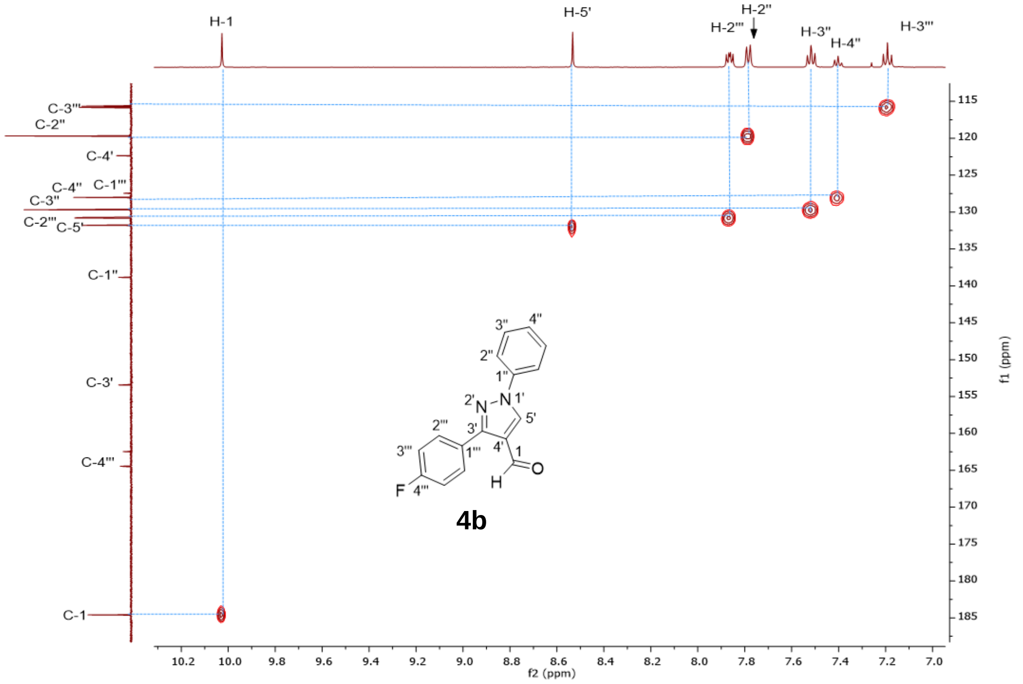

Figure S57. HSQC experiment of 3-(4-fluorophenyl)-1-phenyl-1*H*-pyrazole-4-carbaldehyde **4b**.

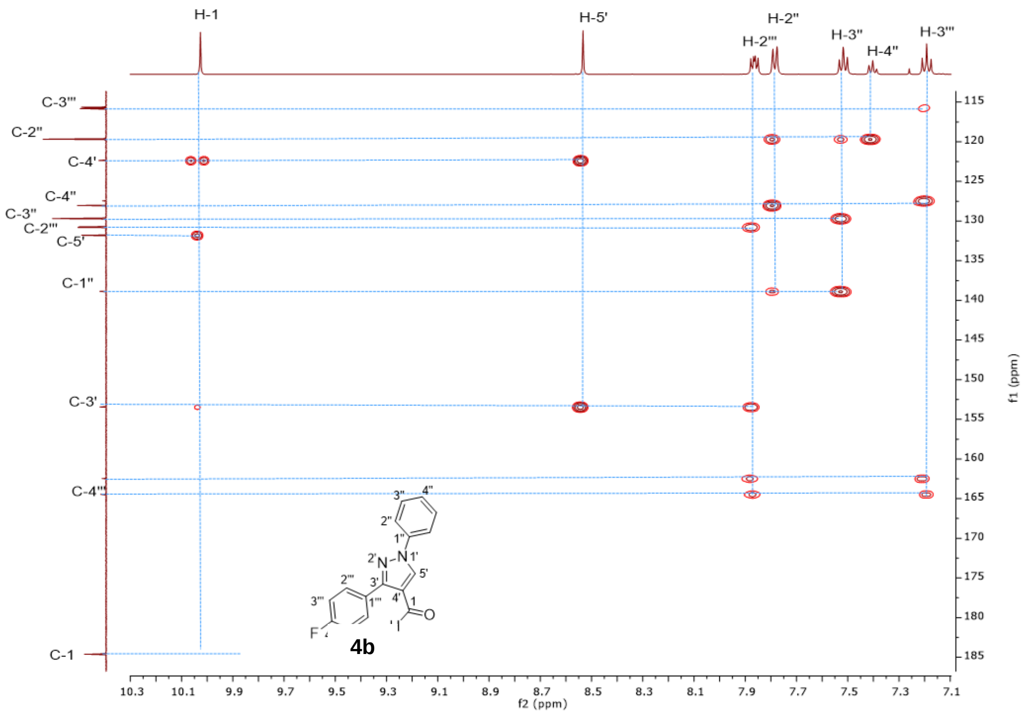

Figure S58. HMBC experiment of 3-(4-fluorophenyl)-1-phenyl-1*H*-pyrazole-4-carbaldehyde **4b**.

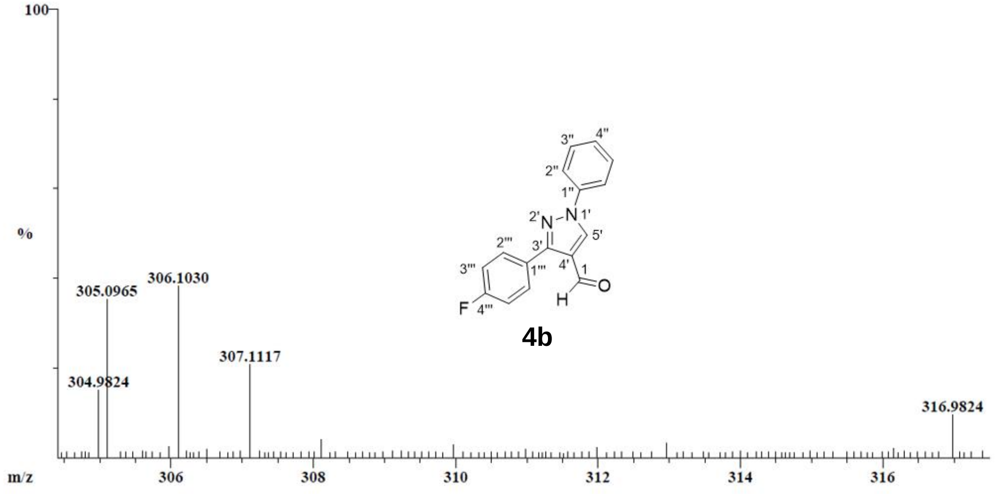

Selected Isotopes : $H_{0-12}C_{0-18}N_{0-3}O_{0-1}F_{0-1}$ Error Limit : 5 ppm Unsaturation Limits : 0 to 50

Measured Mass	% Base	Formula	Calculated Mass	Error	Unsaturation
305.0965	35.3%	$C_{18}H_{12}N_3O F$	305.0964	0.2	14.0

Figure S59. HRMS of 3-(4-fluorophenyl)-1-phenyl-1*H*-pyrazole-4-carbaldehyde **4b**.

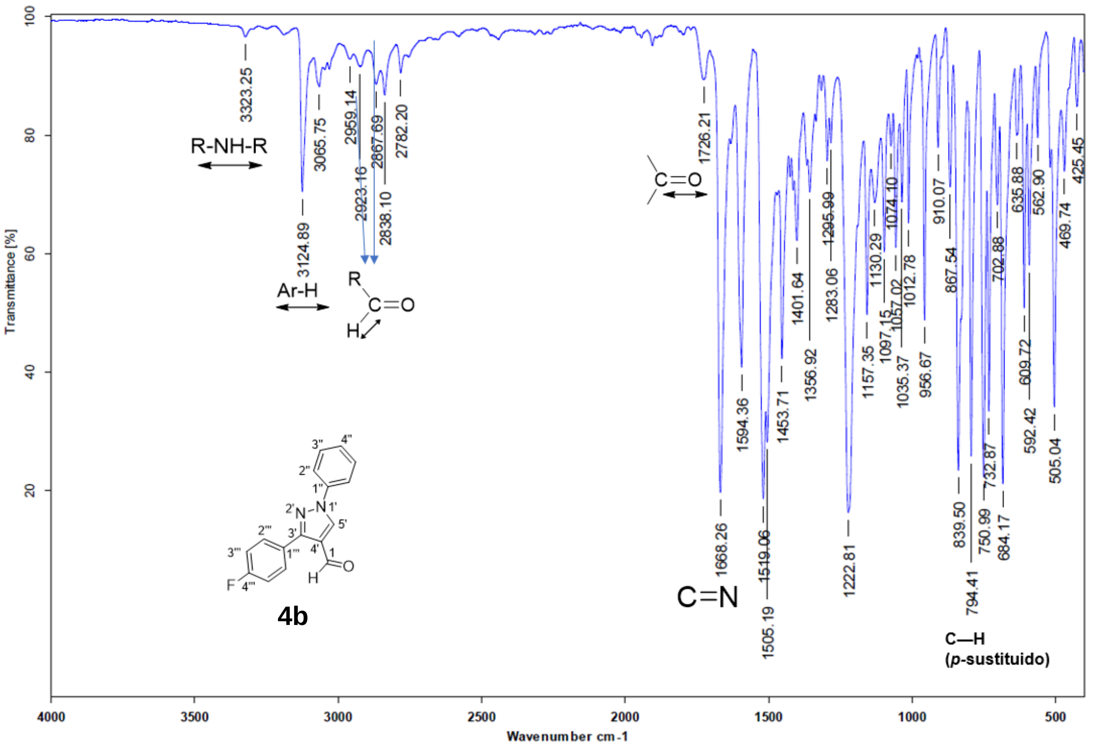

Figure S60. FT-IR of 3-(4-fluorophenyl)-1-phenyl-1*H*-pyrazole-4-carbaldehyde **4b**.

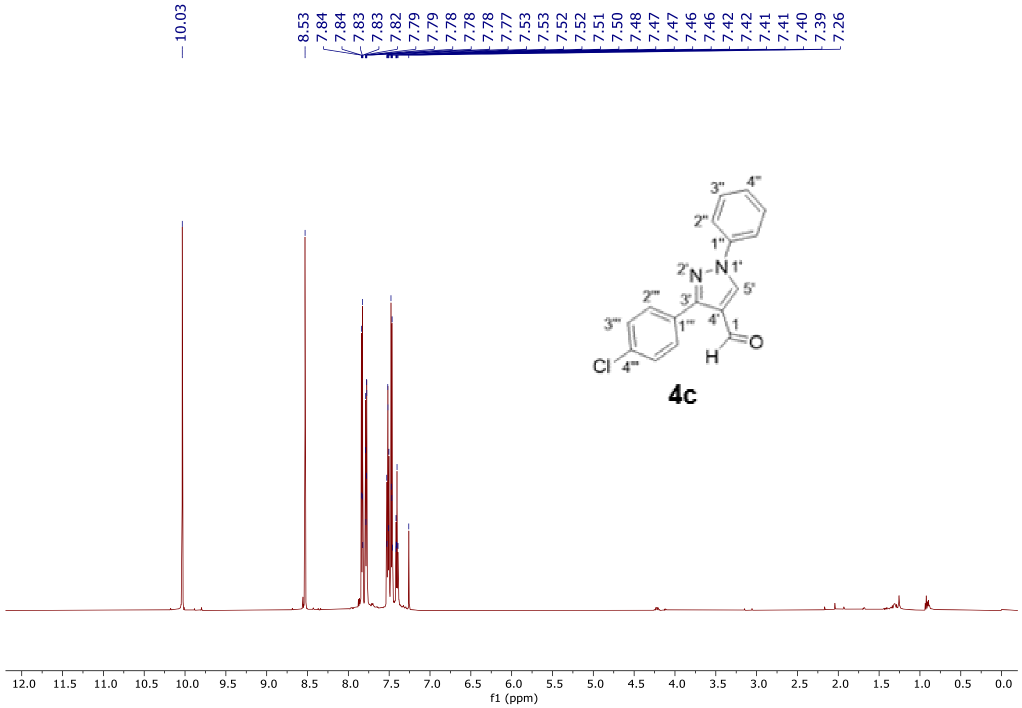

Figure S61. ¹H NMR (500 MHz, $CDCl_3$) of 3-(4-chlorophenyl)-1-phenyl-1*H*-pyrazole-4-carbaldehyde **4c**.

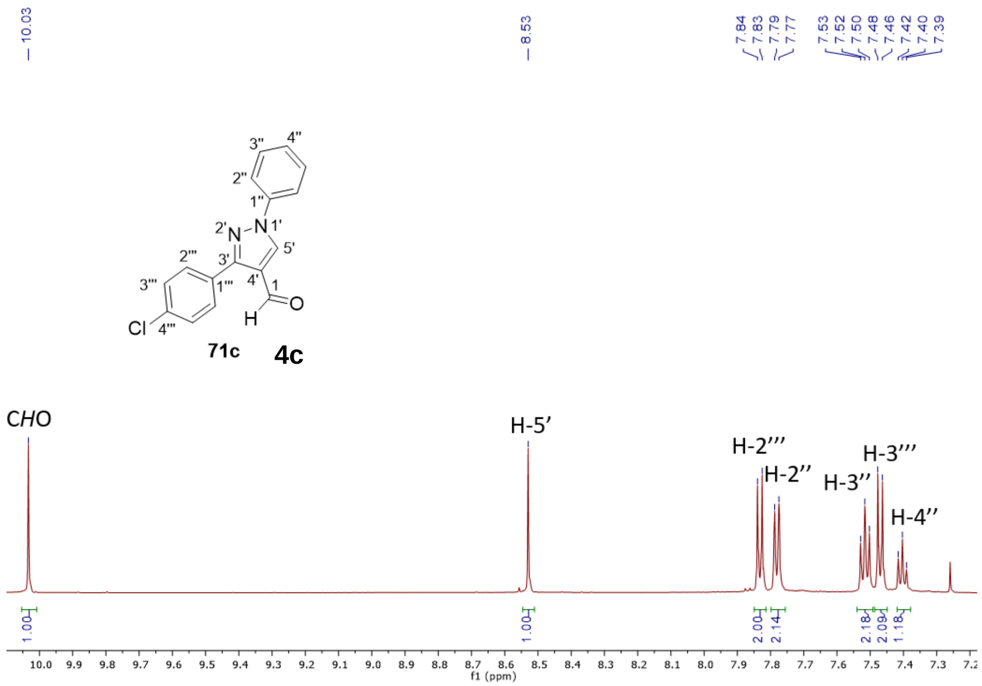

Figure S62. Expansion of ^{1}H NMR (500 MHz, $CDCl_3$) of 3-(4-chlorophenyl)-1-phenyl-1*H*-pyrazole-4-carbaldehyde **4c**.

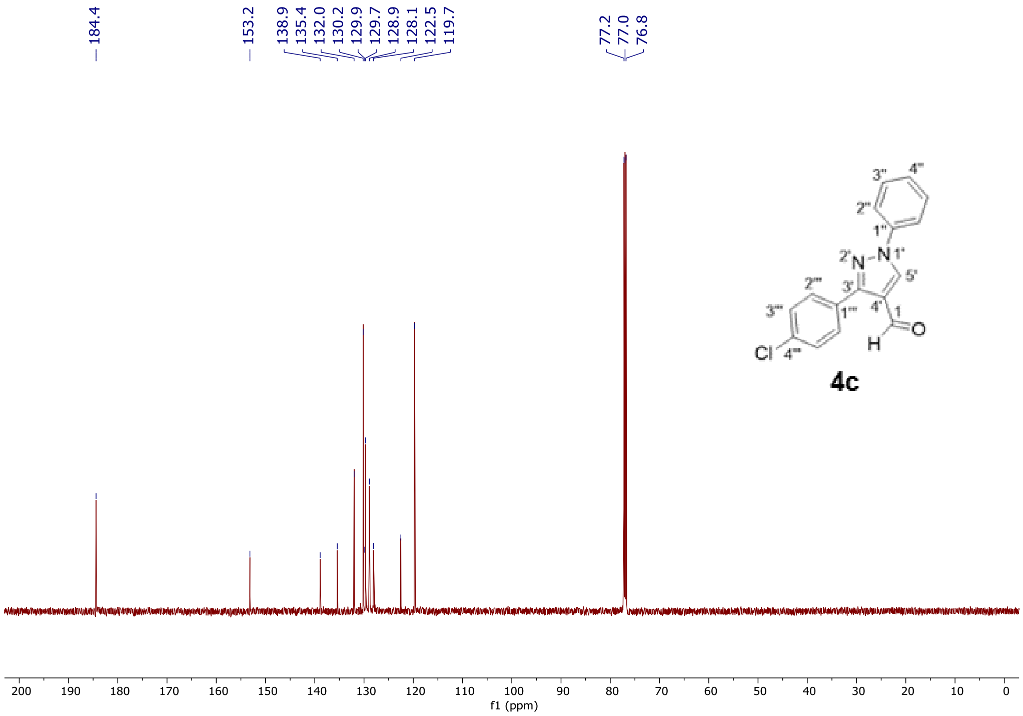

Figure S63. ^{13}C NMR (125 MHz, $CDCl_3$) of 3-(4-chlorophenyl)-1-phenyl-1*H*-pyrazole-4-carbaldehyde **4c**.

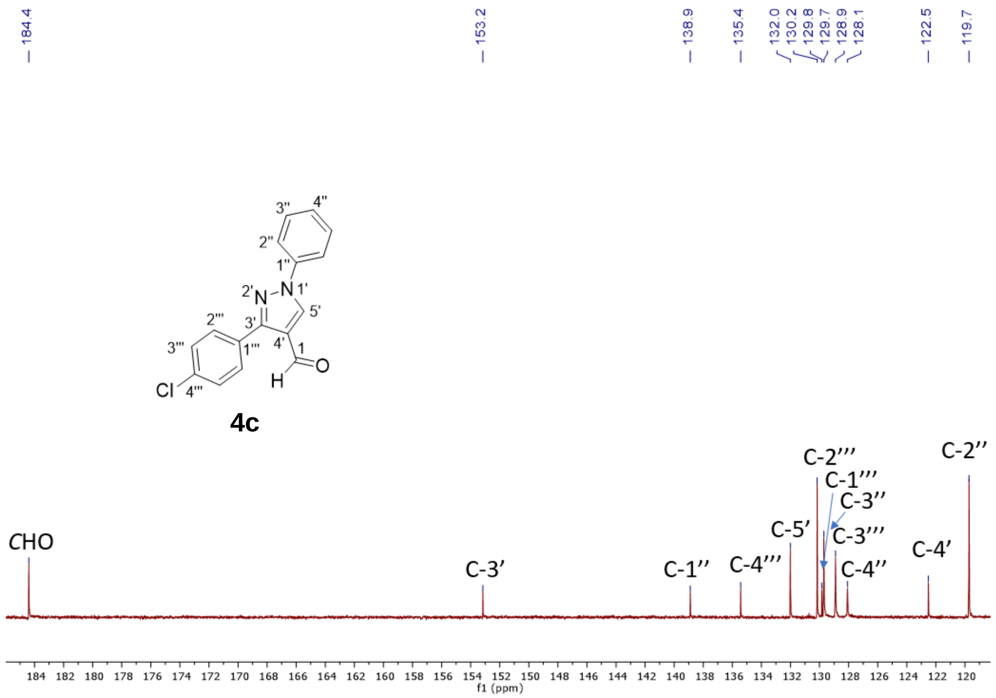

Figure S64. Expansion of ^{13}C NMR (125 MHz, CDCl$_3$) of 3-(4-chlorophenyl)-1-phenyl-1*H*-pyrazole-4-carbaldehyde **4c**.

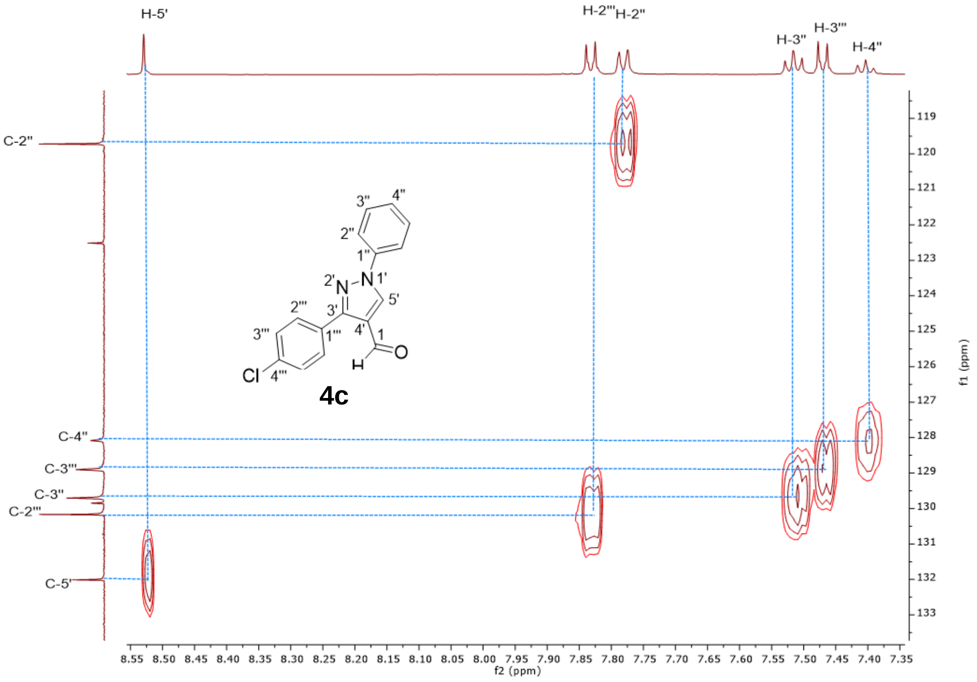

Figure S65. Expansion of HSQC experiment of 3-(4-chlorophenyl)-1-phenyl-1*H*-pyrazole-4-carbaldehyde **4c**.

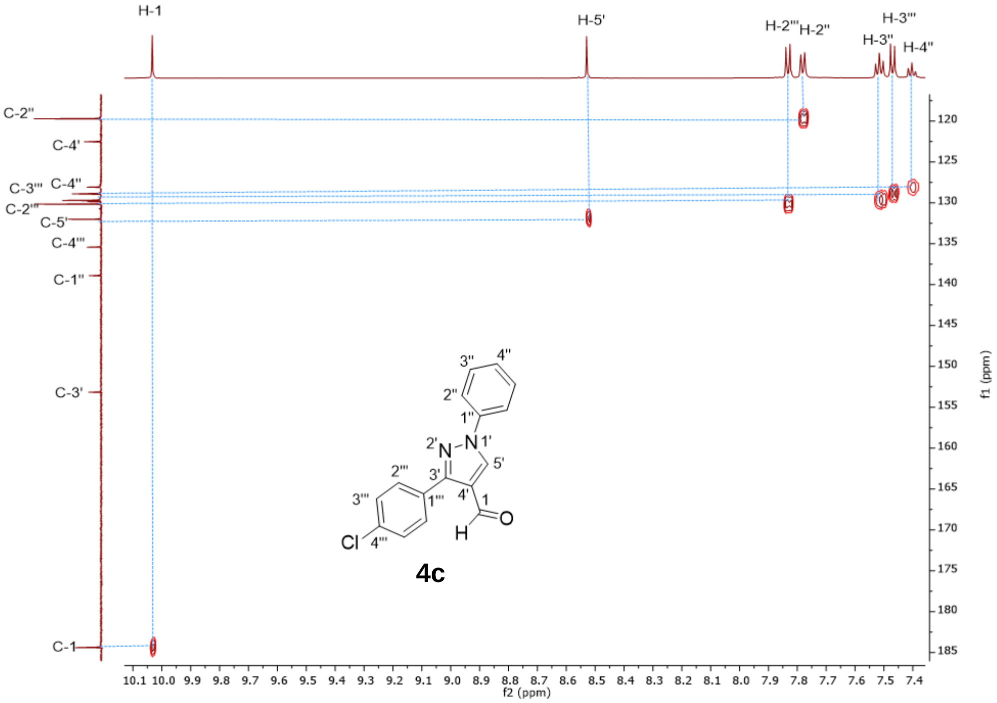

Figure S66. HSQC experiment of 3-(4-chlorophenyl)-1-phenyl-1*H*-pyrazole-4-carbaldehyde **4c**.

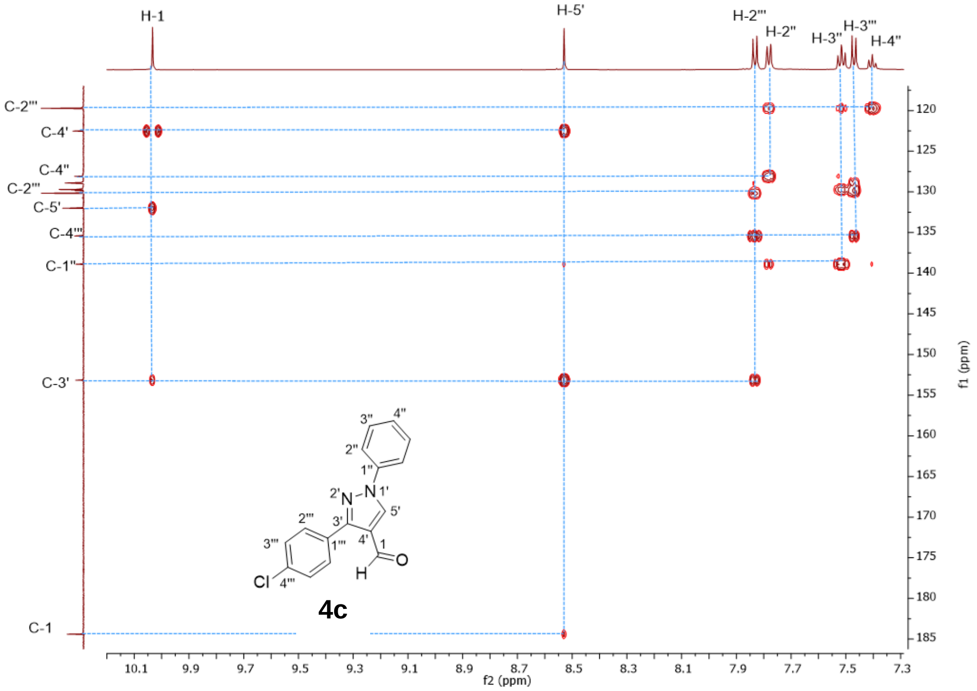

Figure S67. HMBC experiment of 3-(4-chlorophenyl)-1-phenyl-1*H*-pyrazole-4-carbaldehyde **4c**.

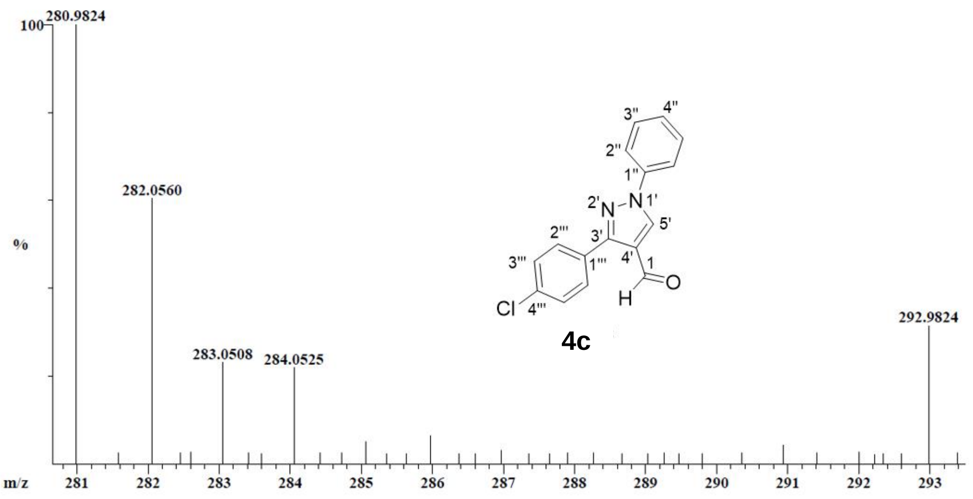

Selected Isotopes : $H_{0-11}C_{0-16}N_{0-2}O_{0-1}Cl_{0-1}$ Error Limit : 5 ppm Unsaturation Limits : 0 to 50

Measured Mass	% Base	Formula	Calculated Mass	Error	Unsaturation
282.0560	60.4%	$C_{16}H_{11}N_2O\,Cl$	282.0560	0.0	12.0

Figure S68. HRMS of 3-(4-chlorophenyl)-1-phenyl-1*H*-pyrazole-4-carbaldehyde **4c**.

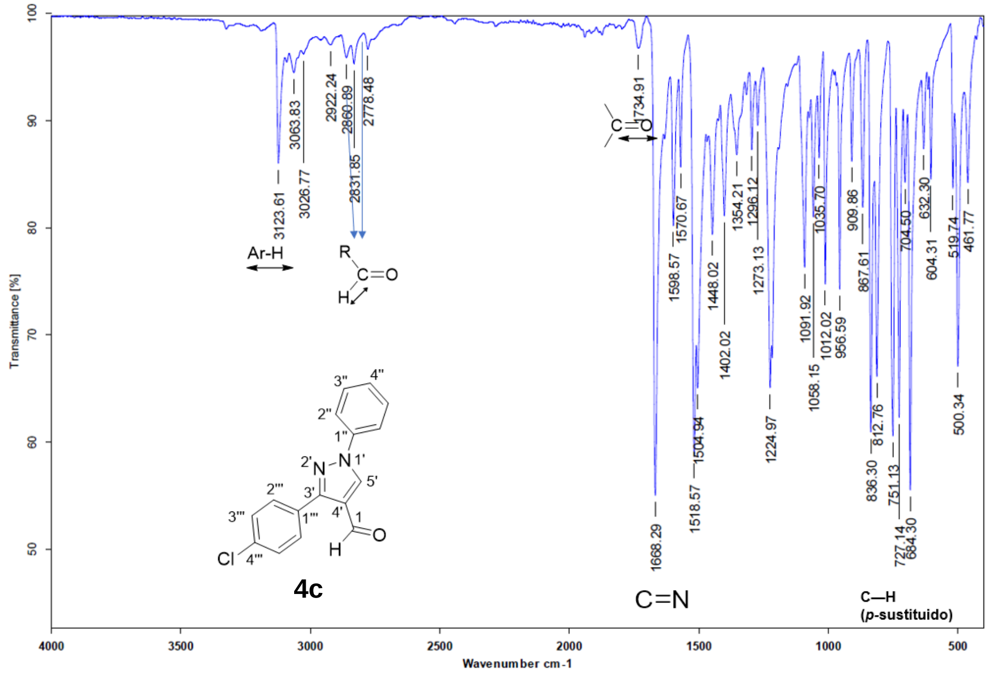

Figure S69. FT-IR of 3-(4-chlorophenyl)-1-phenyl-1*H*-pyrazole-4-carbaldehyde **4c**.

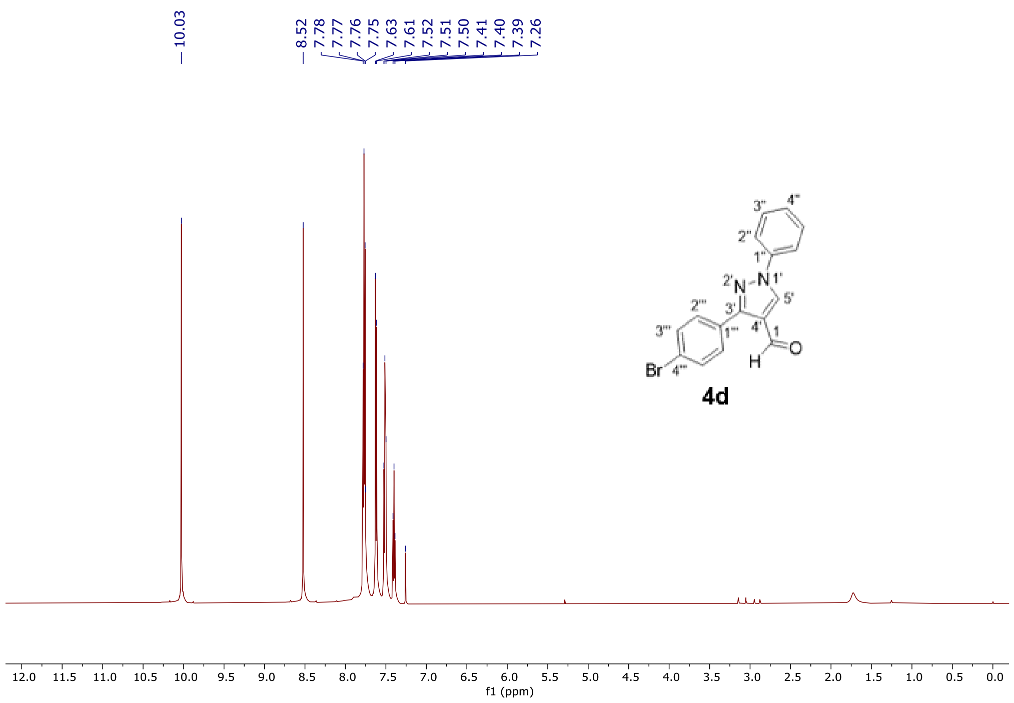

Figure S70. ^{1}H NMR (500 MHz, $CDCl_3$) of 4 3-(4-bromophenyl)-1-phenyl-1*H*-pyrazole-4-carbaldehyde **4d**.

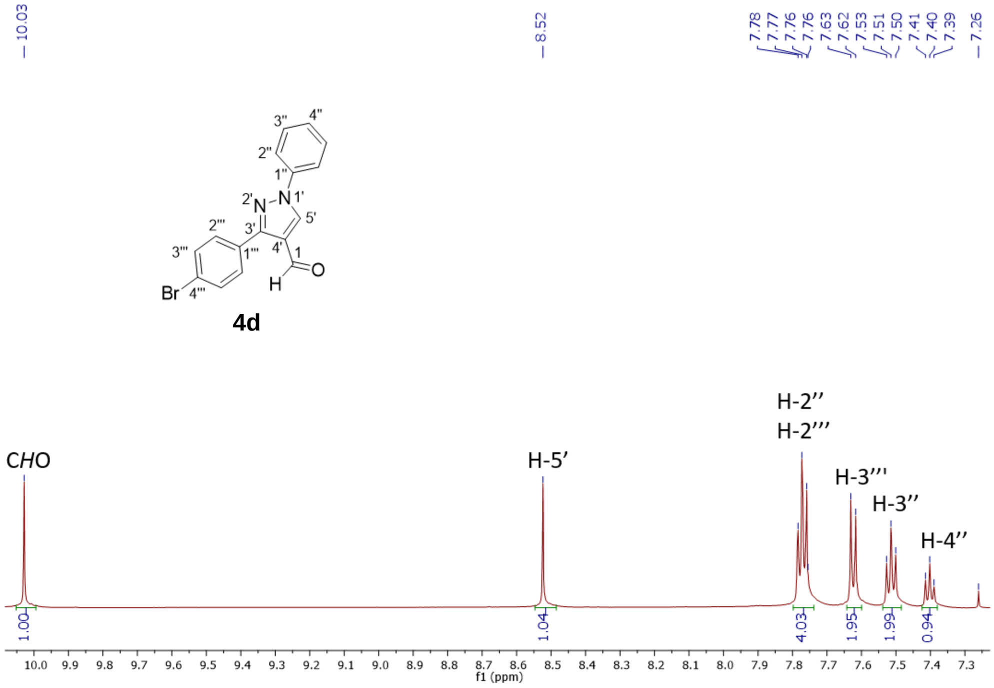

Figure S71. Expansion of ^{1}H NMR (500 MHz, $CDCl_3$) of 4 3-(4-bromophenyl)-1-phenyl-1*H*-pyrazole-4-carbaldehyde **4d**.

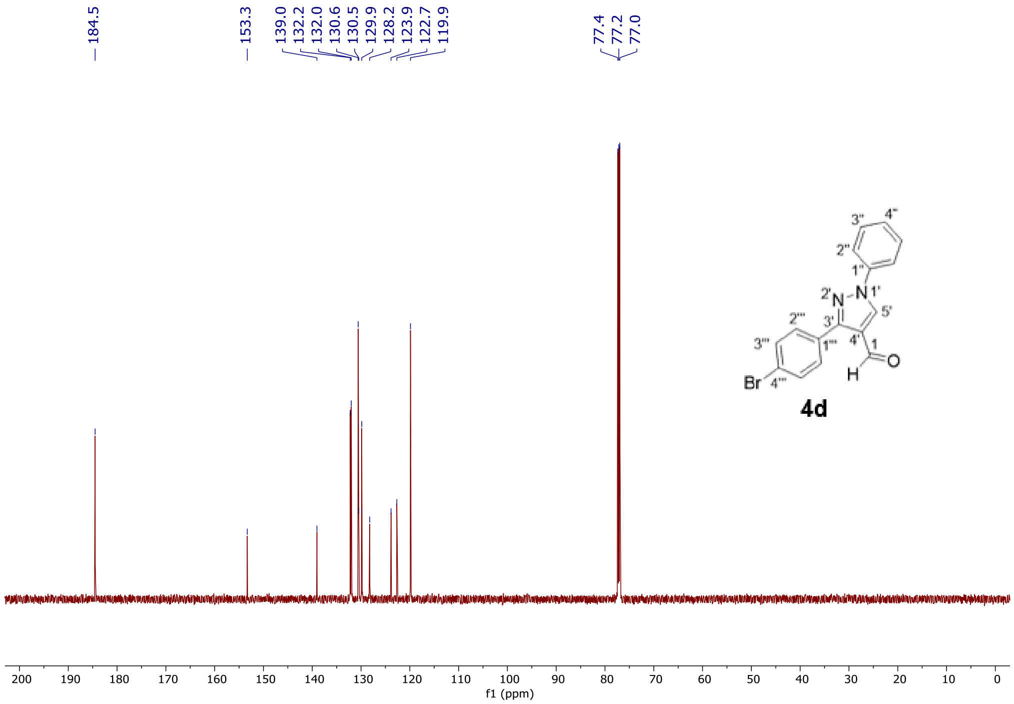

Figure S72. ^{13}C NMR (125 MHz, $CDCl_3$) of 4 3-(4-bromophenyl)-1-phenyl-1*H*-pyrazole-4-carbaldehyde **4d**.

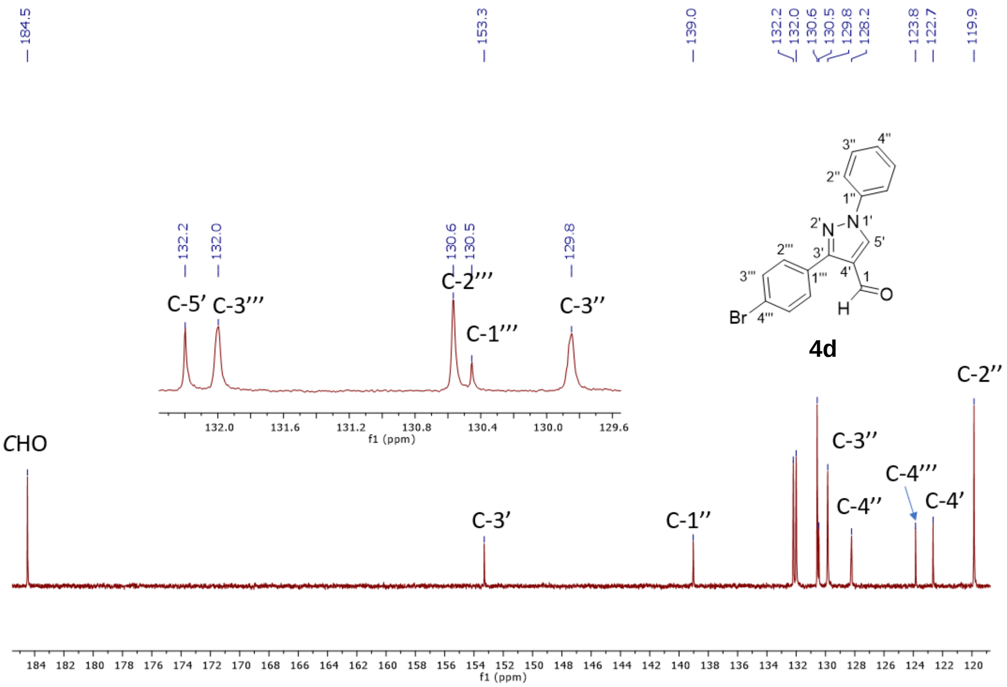

Figure S73. Expansion of ^{13}C NMR (125 MHz, $CDCl_3$) of 4 3-(4-bromophenyl)-1-phenyl-1*H*-pyrazole-4-carbaldehyde **4d**.

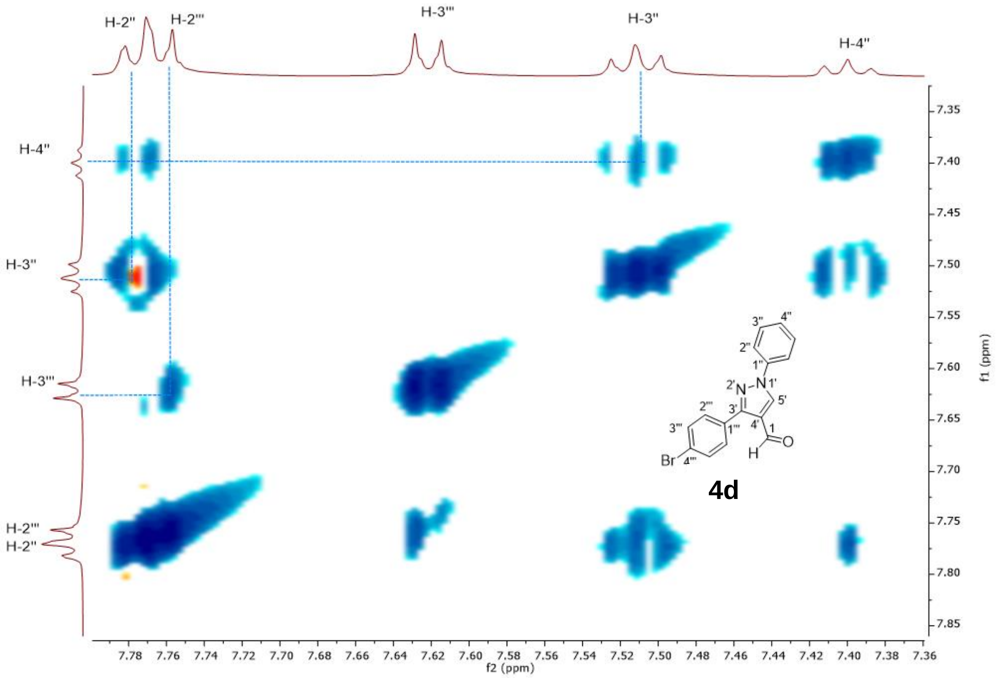

Figure S74. COSY experiment of 4 3-(4-bromophenyl)-1-phenyl-1*H*-pyrazole-4-carbaldehyde **4d**.

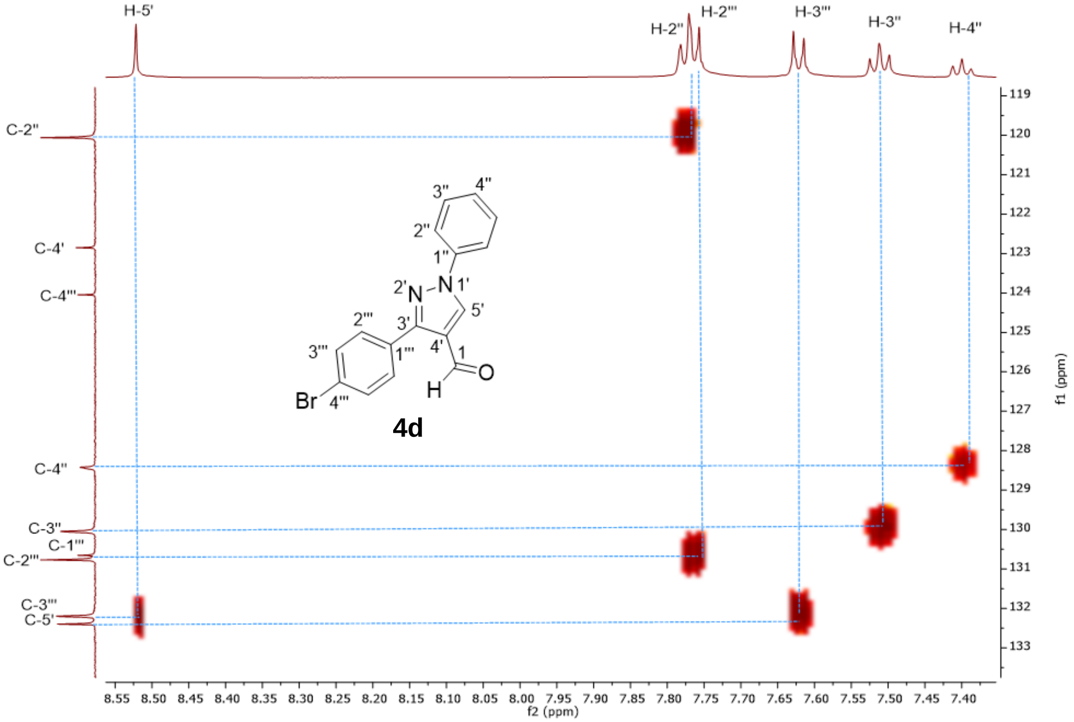

Figure S75. HSQC experiment of 4 3-(4-bromophenyl)-1-phenyl-1*H*-pyrazole-4-carbaldehyde **4d**.

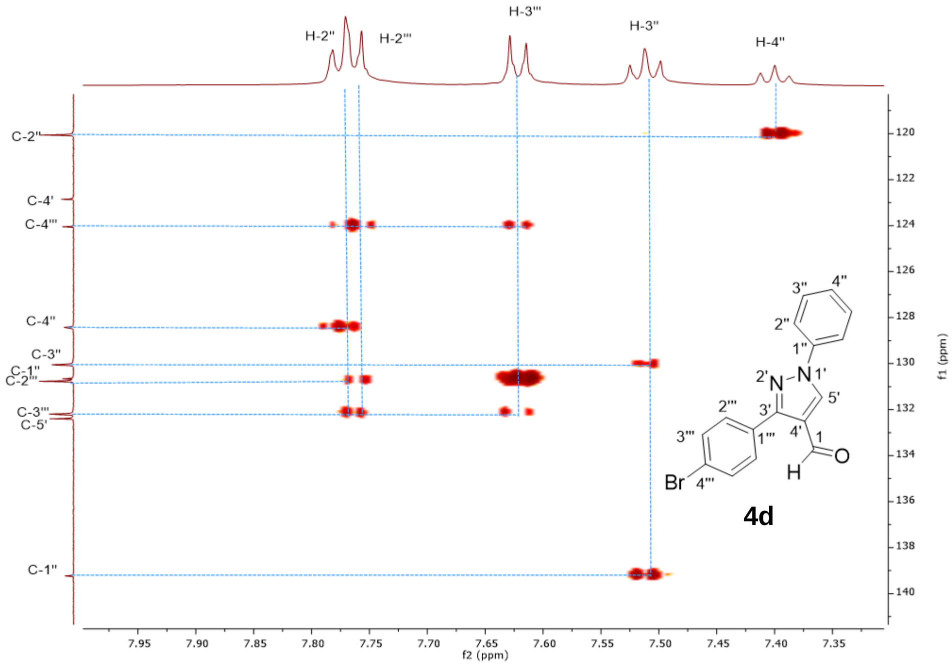

Figure S76. HMBC experiment of 4 3-(4-bromophenyl)-1-phenyl-1*H*-pyrazole-4-carbaldehyde **4d**.

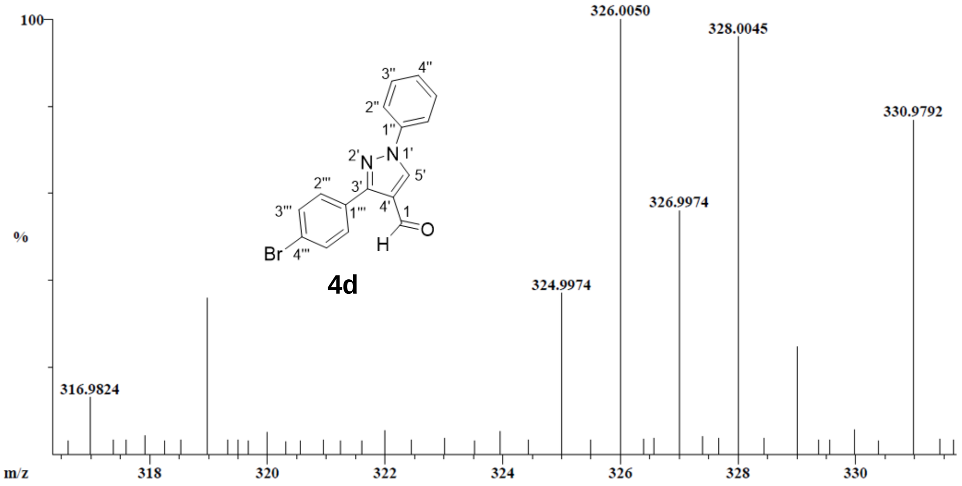

Selected Isotopes : $H_{0-11}C_{0-16}N_{0-2}O_{0-1}Br_{0-1}$			Error Limit : 5 ppm	Unsaturation Limits : 0 to 50	
Measured Mass	% Base	Formula	Calculated Mass	Error	Unsaturation
326.0050	100.0%	$C_{16}H_{11}N_2O$ Br	326.0055	-1.5	12.0

Figure S77. HRMS of 4 3-(4-bromophenyl)-1-phenyl-1*H*-pyrazole-4-carbaldehyde **4d**.

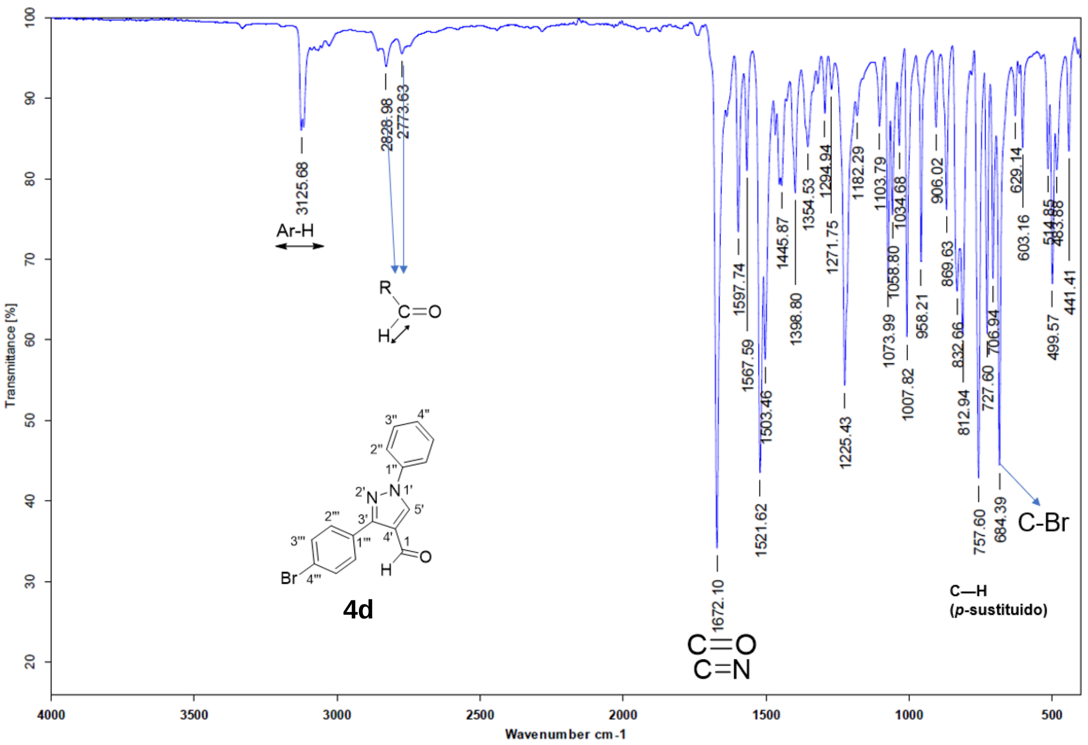

Figure S78. FT-IR of 4 3-(4-bromophenyl)-1-phenyl-1*H*-pyrazole-4-carbaldehyde **4d**.

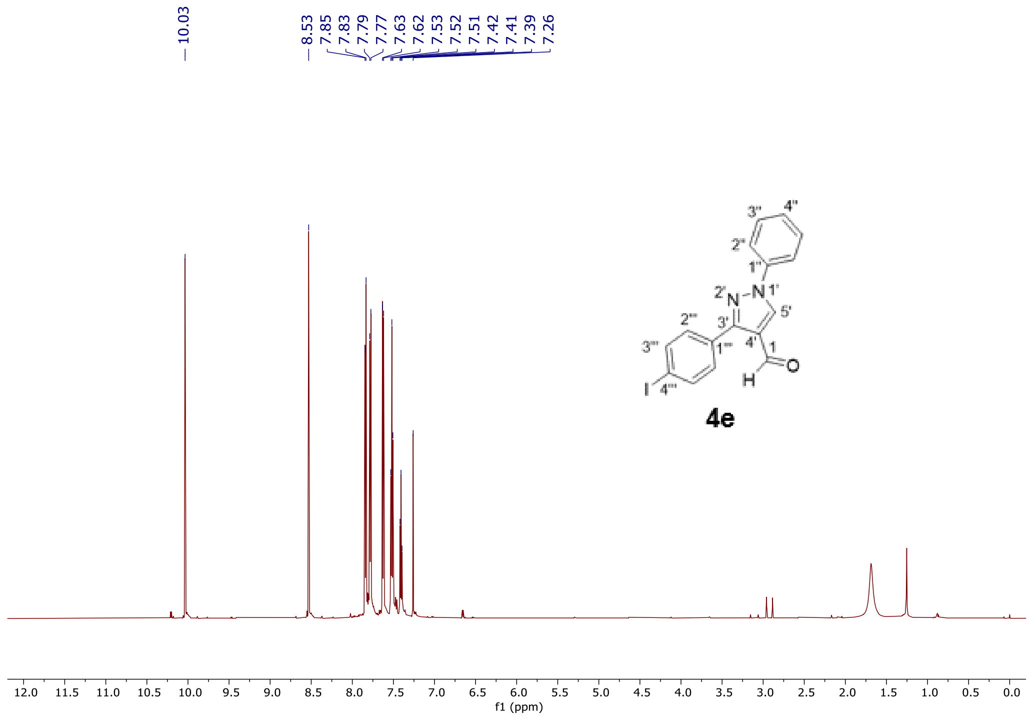

Figure S79. ^{1}H NMR (500 MHz, $CDCl_3$) of 4 3-(4-iodophenyl)-1-phenyl-1*H*-pyrazole-4-carbaldehyde **4e**.

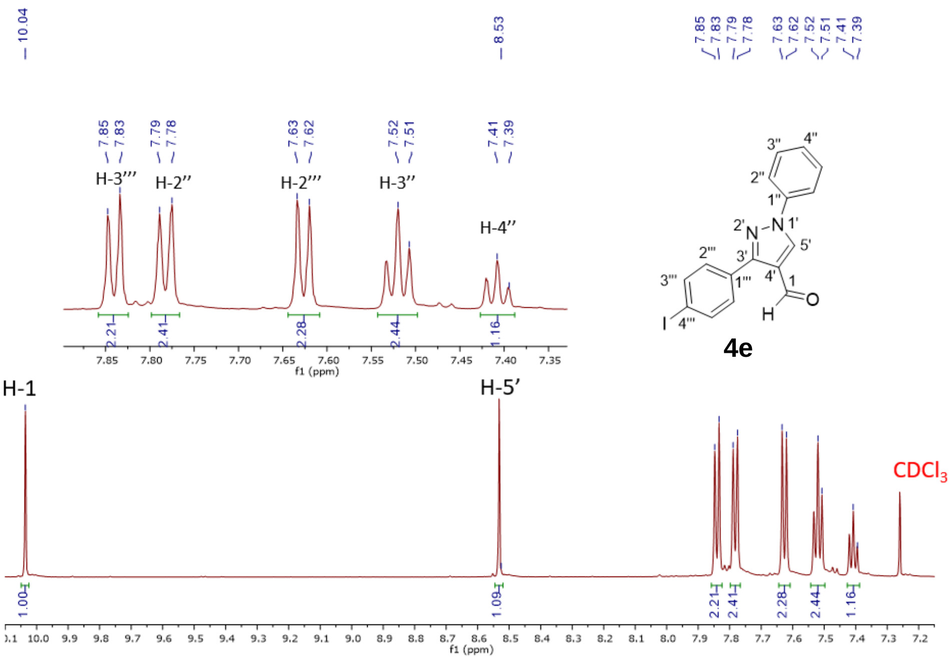

Figure S80. Expansion of ^{1}H NMR (500 MHz, CDCl$_3$) of 4 3-(4-iodophenyl)-1-phenyl-1*H*-pyrazole-4-carbaldehyde **4e**.

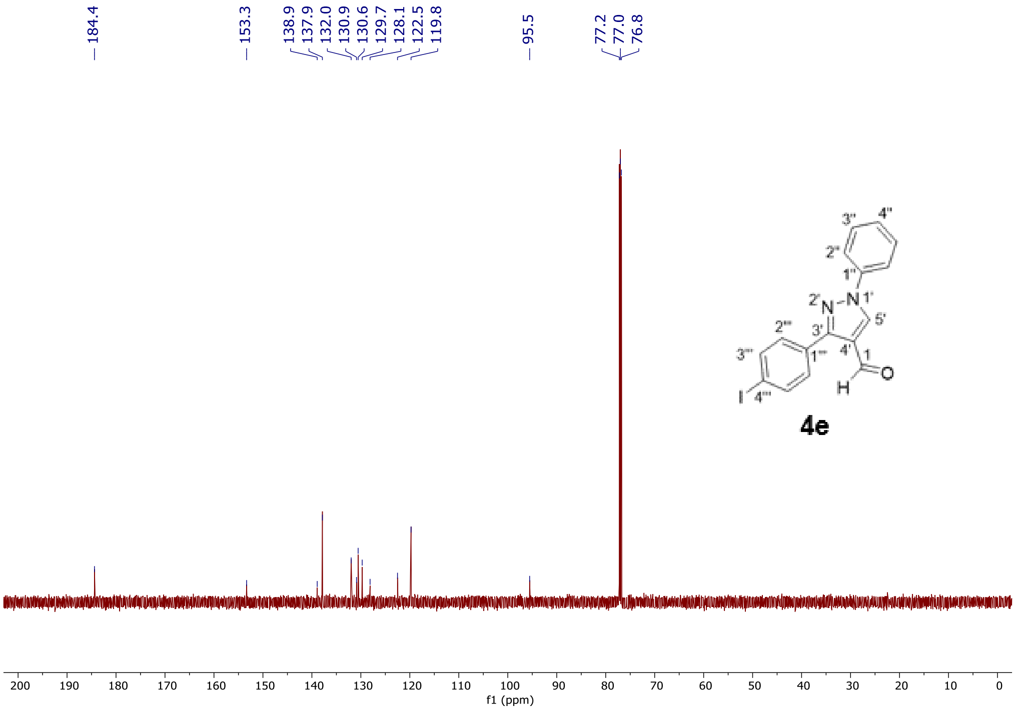

Figure S81. ^{13}C NMR (125 MHz, CDCl$_3$) of 4 3-(4-iodophenyl)-1-phenyl-1*H*-pyrazole-4-carbaldehyde **4e**.

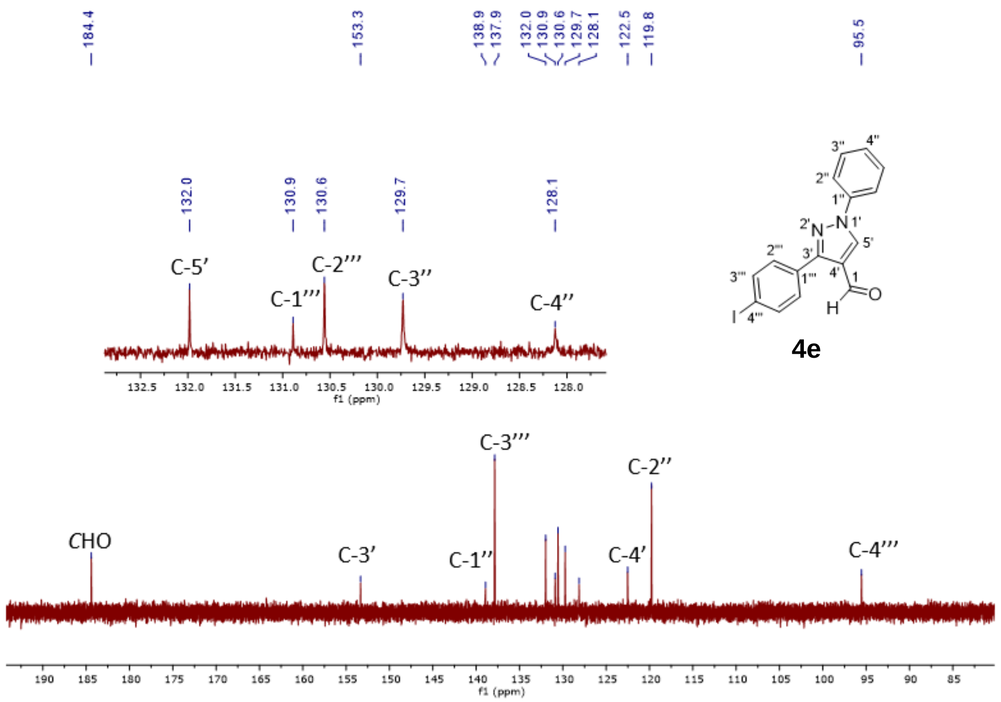

Figure S82. Expansion of ^{13}C NMR (125 MHz, $CDCl_3$) of 4 3-(4-iodophenyl)-1-phenyl-1*H*-pyrazole-4-carbaldehyde **4e**.

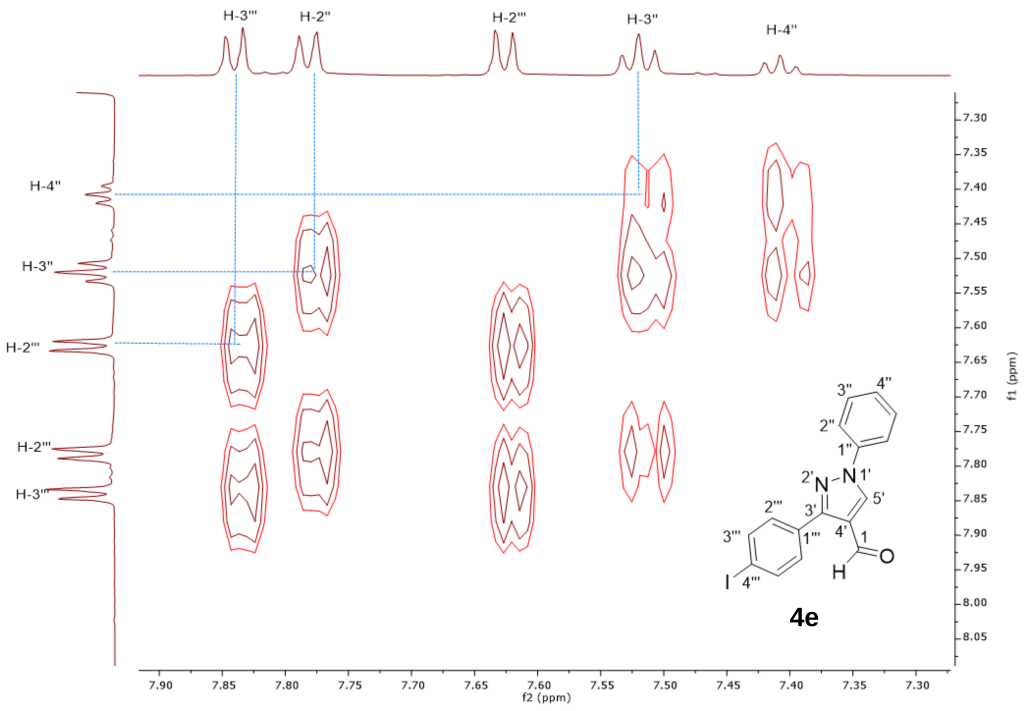

Figure S83. COSY experiment of 4 3-(4-iodophenyl)-1-phenyl-1*H*-pyrazole-4-carbaldehyde **4e**.

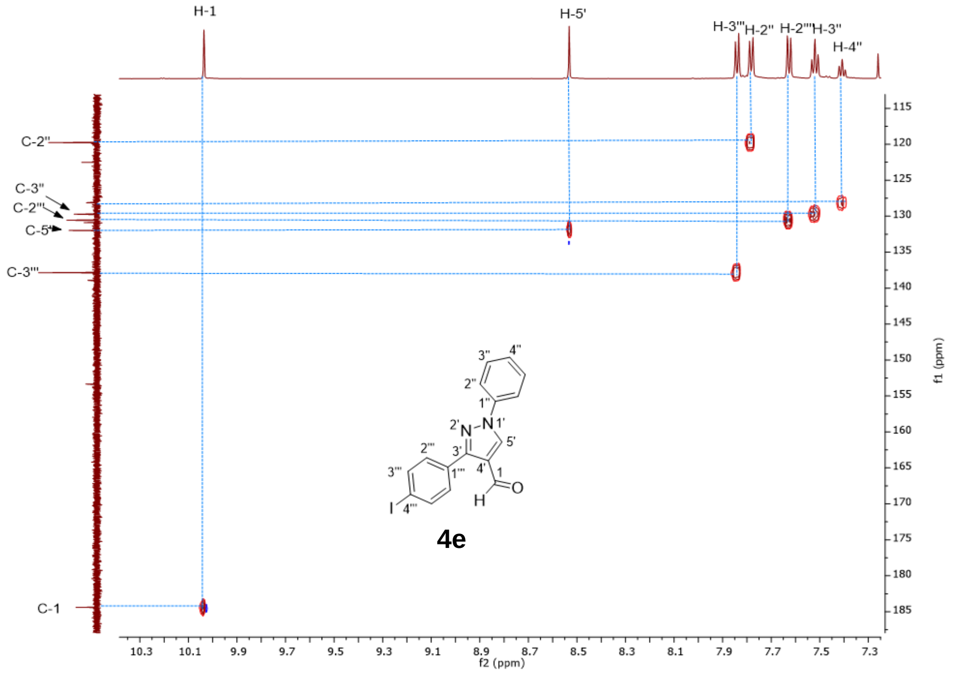

Figure S84. HSQC experiment of 4 3-(4-iodophenyl)-1-phenyl-1*H*-pyrazole-4-carbaldehyde **4e**.

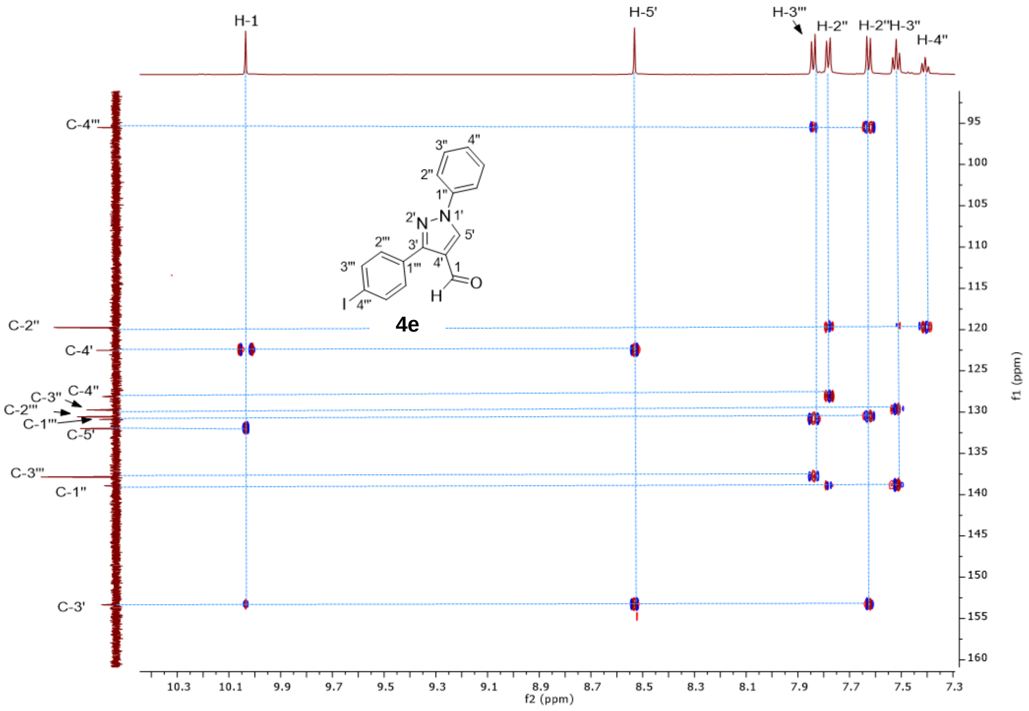

Figure S85. HMBC experiment of 4 3-(4-iodophenyl)-1-phenyl-1*H*-pyrazole-4-carbaldehyde **4e**.

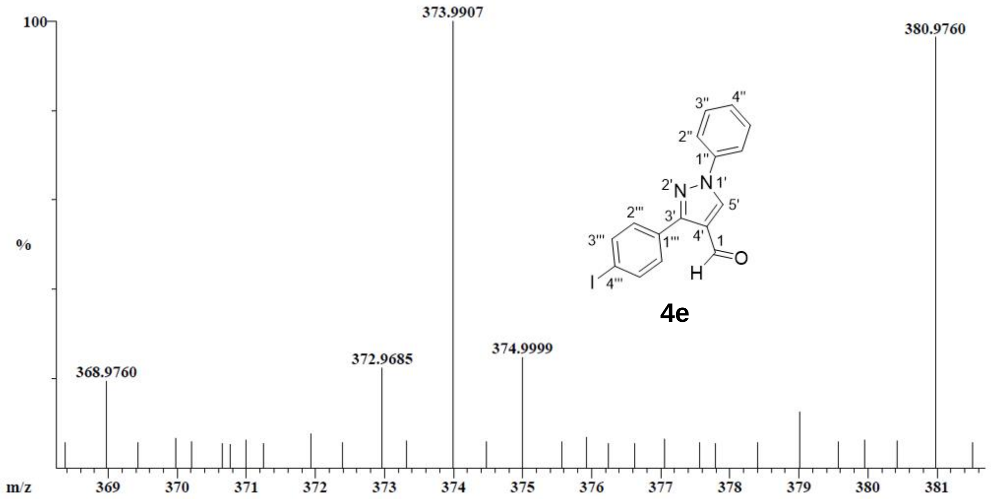

Selected Isotopes : $H_{0-11}C_{0-16}N_{0-2}O_{0-1}I_{0-1}$ Error Limit : 5 ppm Unsaturation Limits : 0 to 50

Measured Mass	% Base	Formula	Calculated Mass	Error	Unsaturation
373.9907	100.0%	$C_{16}H_{11}N_2OI$	373.9916	-2.5	12.0

Figure S86. HRMS of 3-(4-iodophenyl)-1-phenyl-1*H*-pyrazole-4-carbaldehyde **4e**.

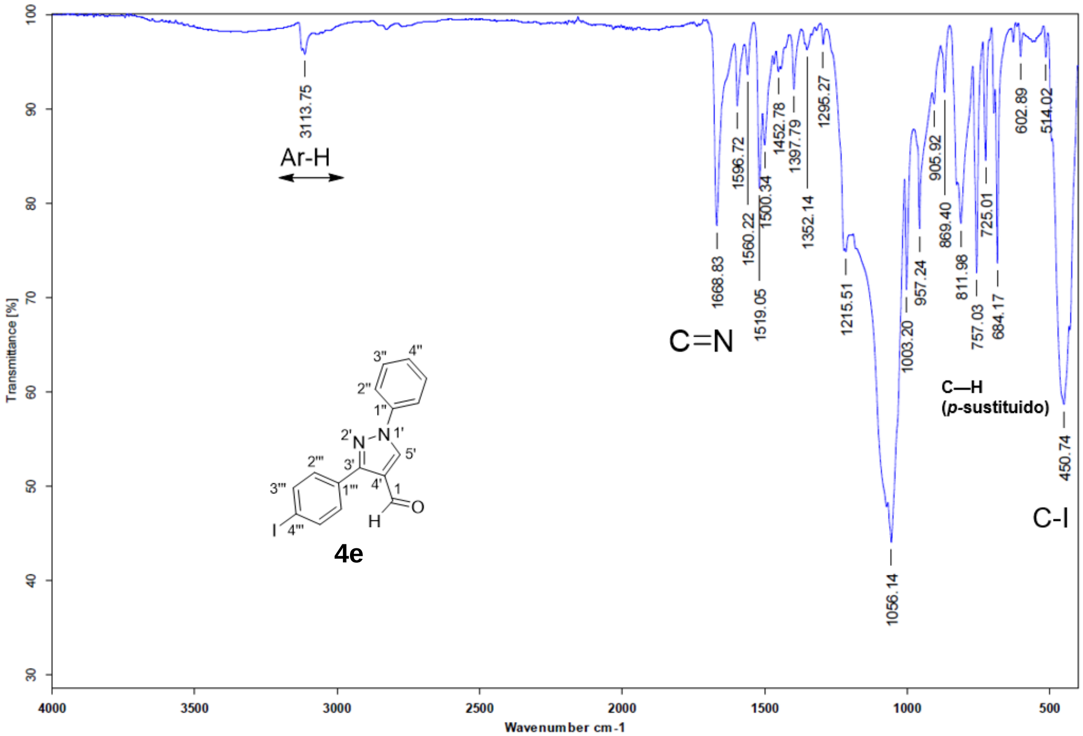

Figure S87. FT-IR of 4 3-(4-iodophenyl)-1-phenyl-1*H*-pyrazole-4-carbaldehyde **4e**.

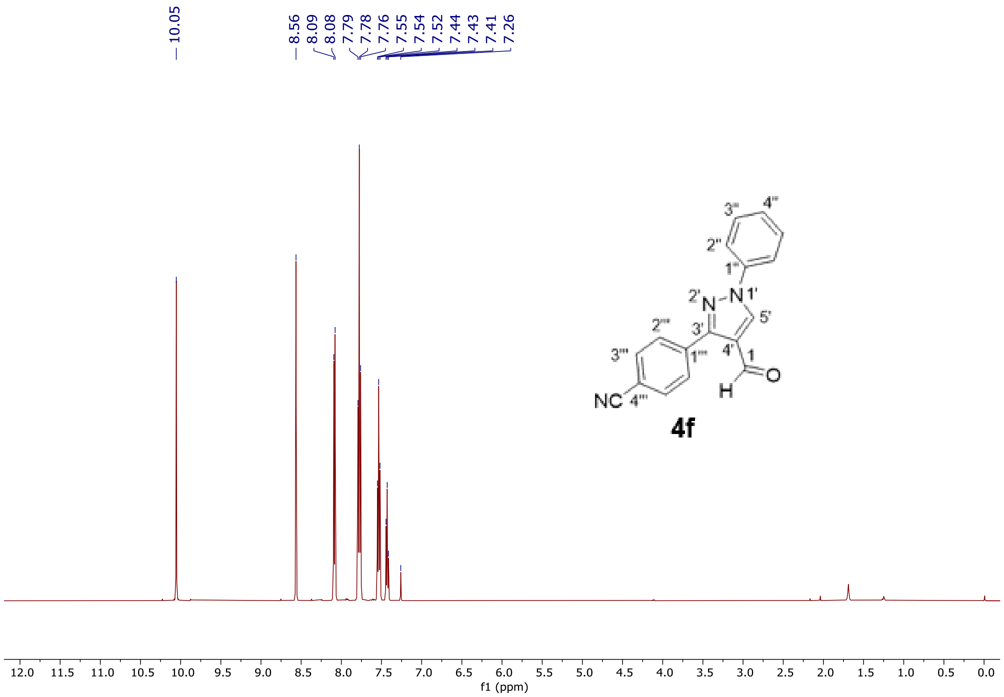

Figure S88. ^{1}H NMR (500 MHz, $CDCl_3$) of 4-(4-formyl-1-phenyl-1*H*-pyrazol-3-yl)benzonitrile **4f**.

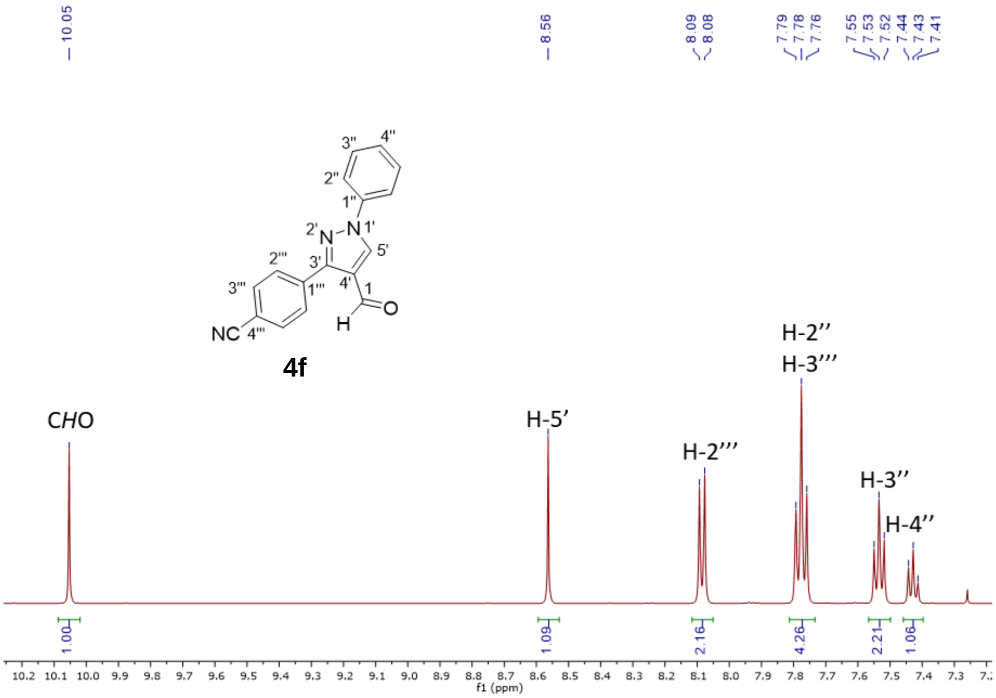

Figure S89. Expansion of ^{1}H NMR (500 MHz, $CDCl_3$) of 4-(4-formyl-1-phenyl-1*H*-pyrazol-3-yl)benzonitrile **4f**.

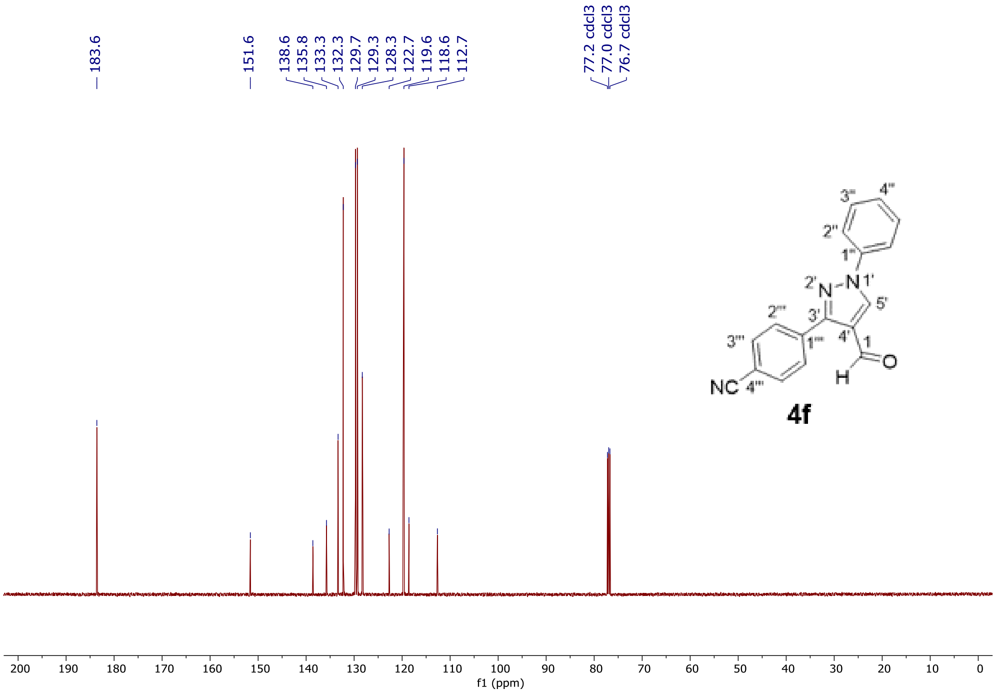

Figure S90. ^{13}C NMR (125 MHz, $CDCl_3$) of 4-(4-formyl-1-phenyl-1*H*-pyrazol-3-yl)benzonitrile **4f**.

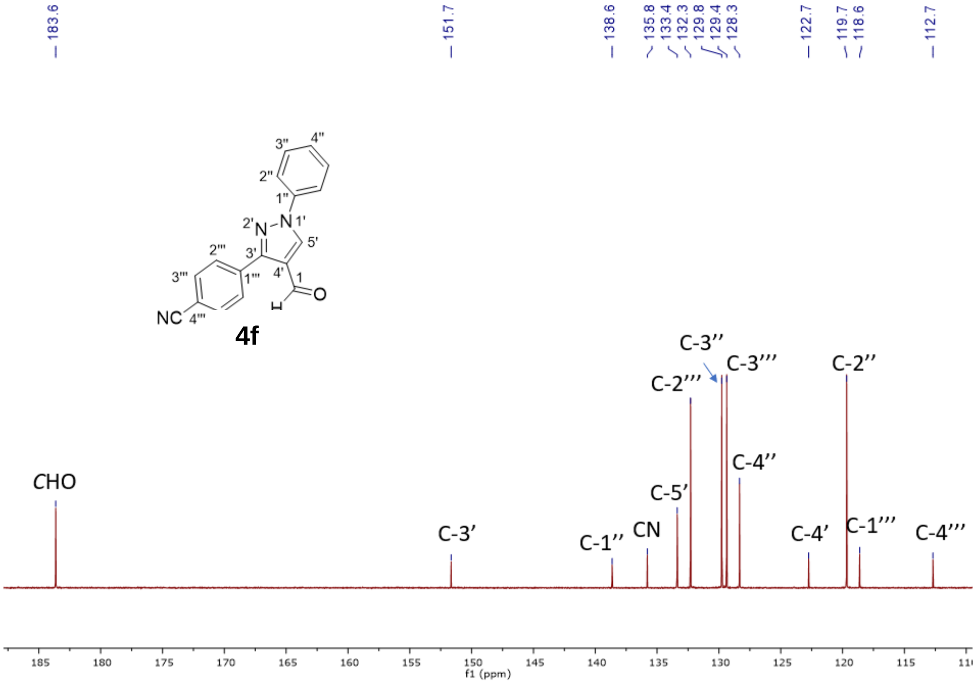

Figure S91. Expansion of ^{13}C NMR (125 MHz, $CDCl_3$) of 4-(4-formyl-1-phenyl-1*H*-pyrazol-3-yl)benzonitrile **4f**.

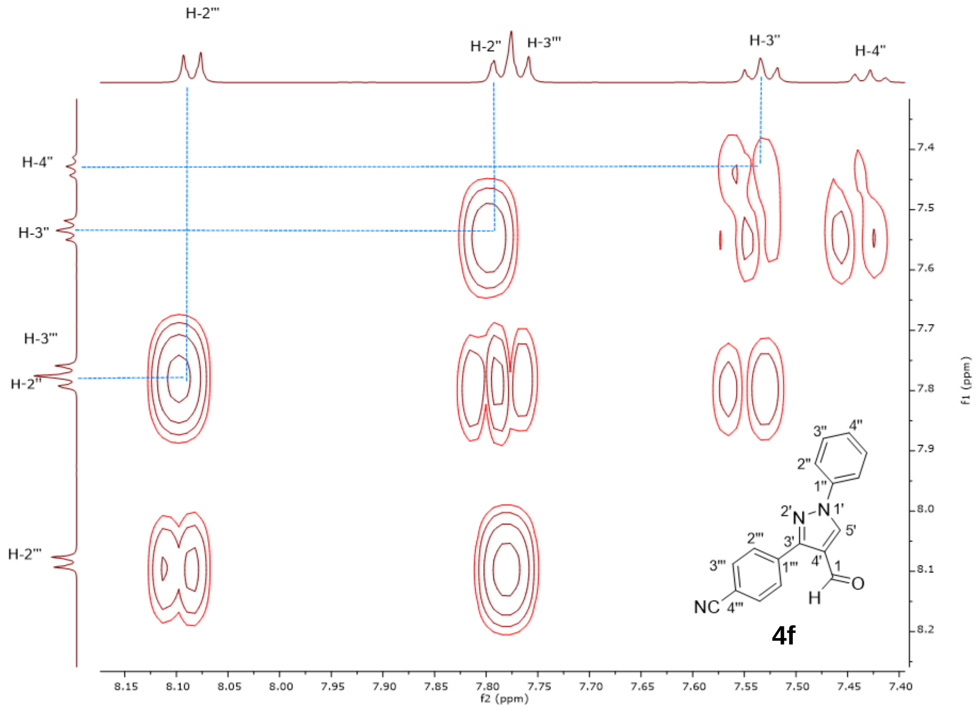

Figure S92. COSY experiment of 4-(4-formyl-1-phenyl-1*H*-pyrazol-3-yl)benzonitrile **4f**.

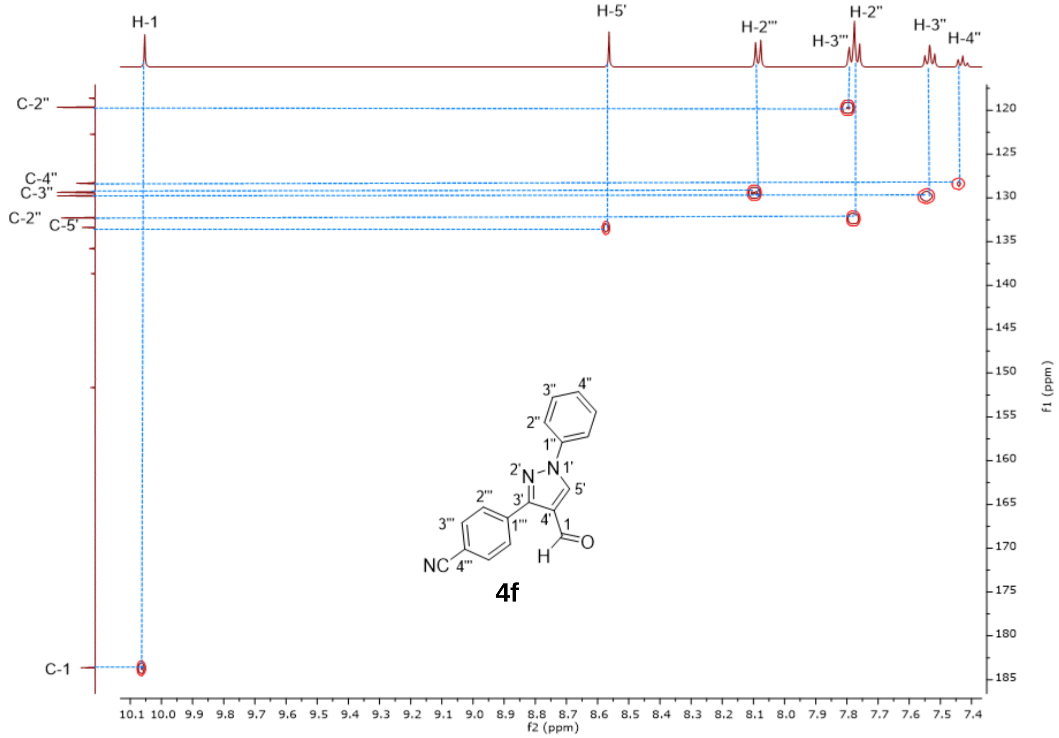

Figure S93. HSQC experiment of 4-(4-formyl-1-phenyl-1*H*-pyrazol-3-yl)benzonitrile **4f**.

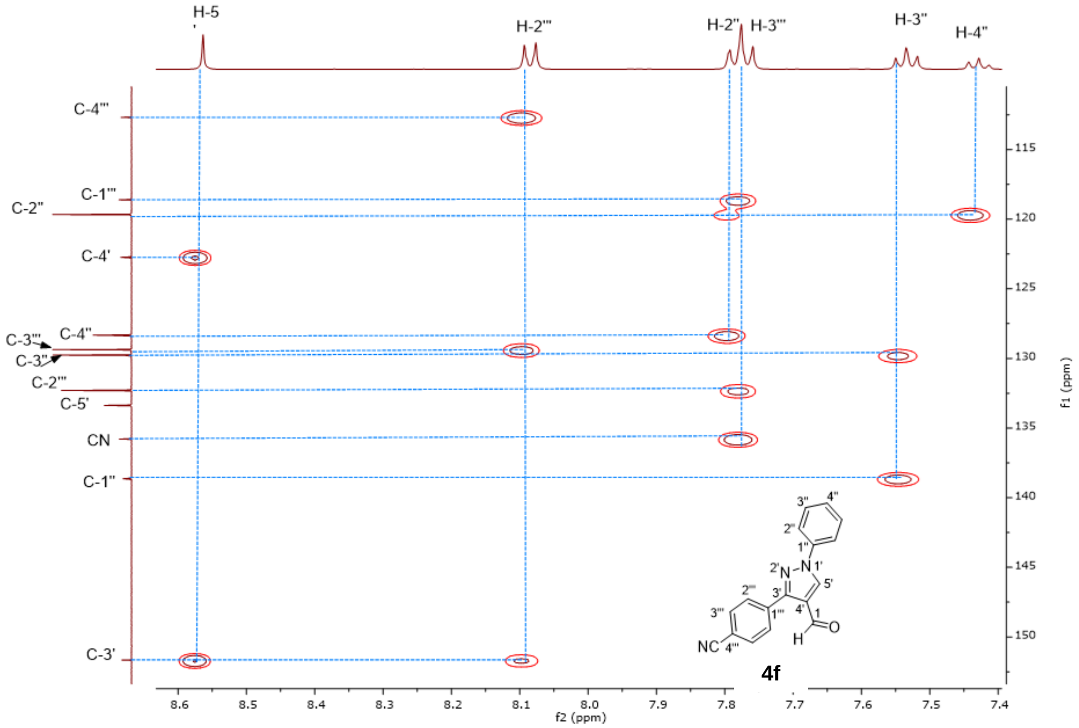

Figure S94. HMBC experiment of 4-(4-formyl-1-phenyl-1*H*-pyrazol-3-yl)benzonitrile **4f**.

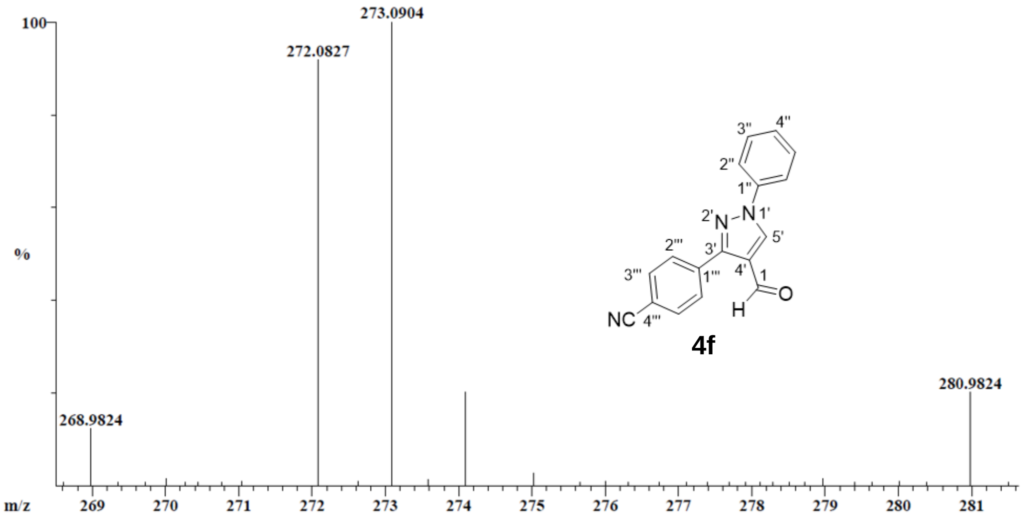

Selected Isotopes : $H_{0-11}C_{0-17}N_{0-3}O_{0-1}$ Error Limit : 5 ppm Unsaturation Limits : 0 to 50

Measured Mass	% Base	Formula	Calculated Mass	Error	Unsaturation
273.0904	100.0%	$C_{17}H_{11}N_3O$	273.0902	0.7	14.0

Figure S95. HRMS of 4-(4-formyl-1-phenyl-1*H*-pyrazol-3-yl)benzonitrile **4f**.

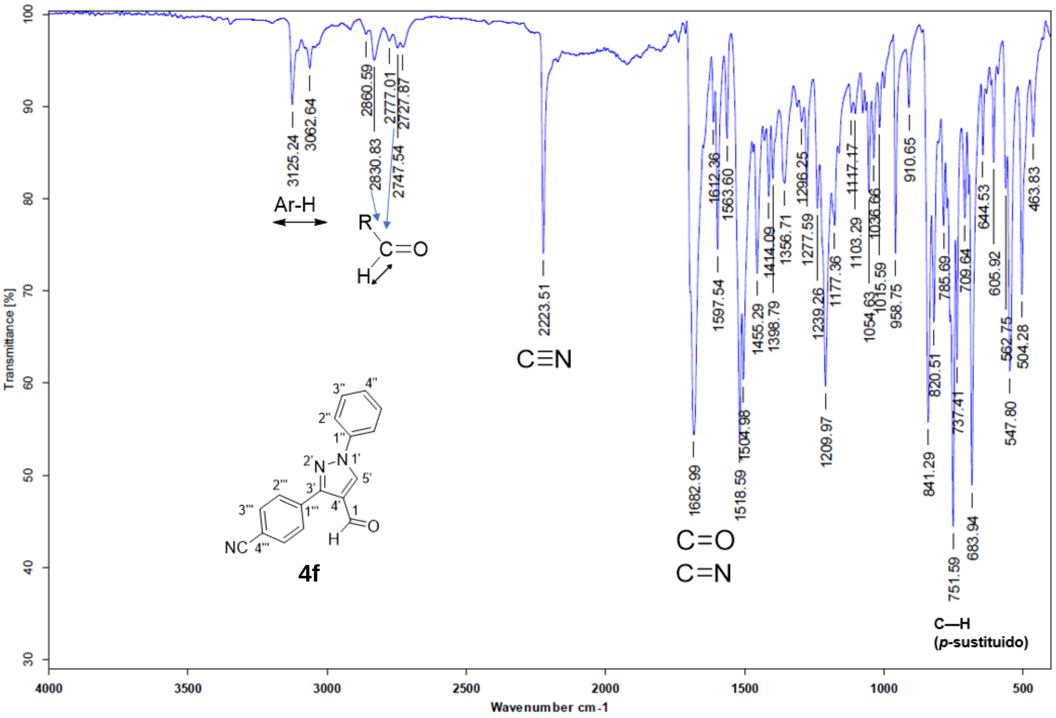

Figure S96. FT-IR of 4-(4-formyl-1-phenyl-1*H*-pyrazol-3-yl)benzonitrile **4f**.

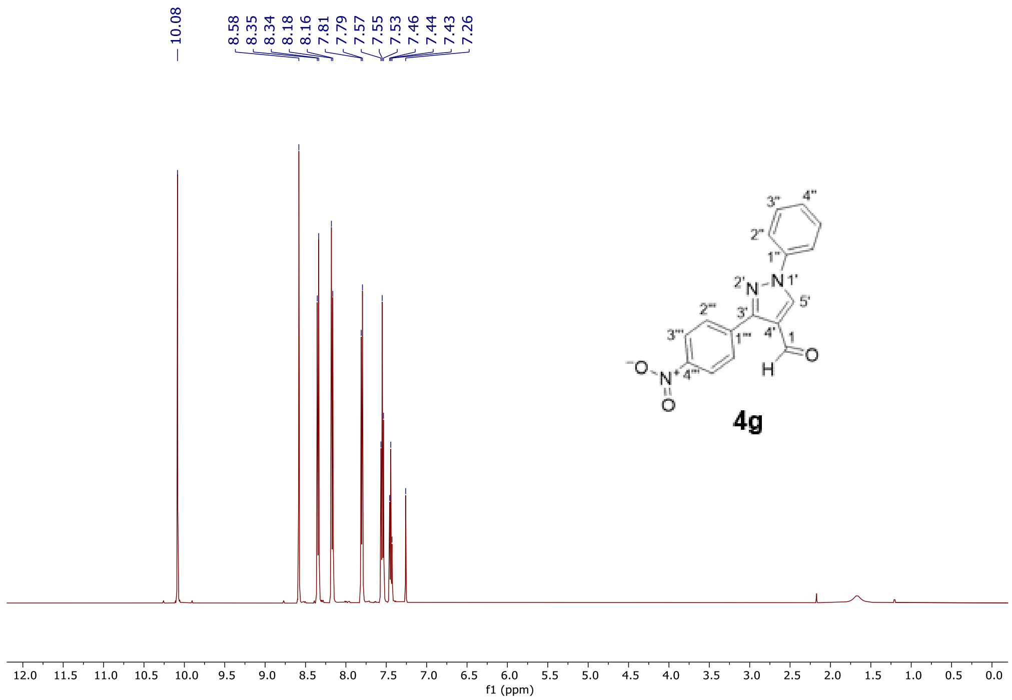

Figure S97. ^{1}H NMR (500 MHz, $CDCl_3$) of 3-(4-nitrophenyl)-1-phenyl-1*H*-pyrazole-4-carbaldehyde **4g**.

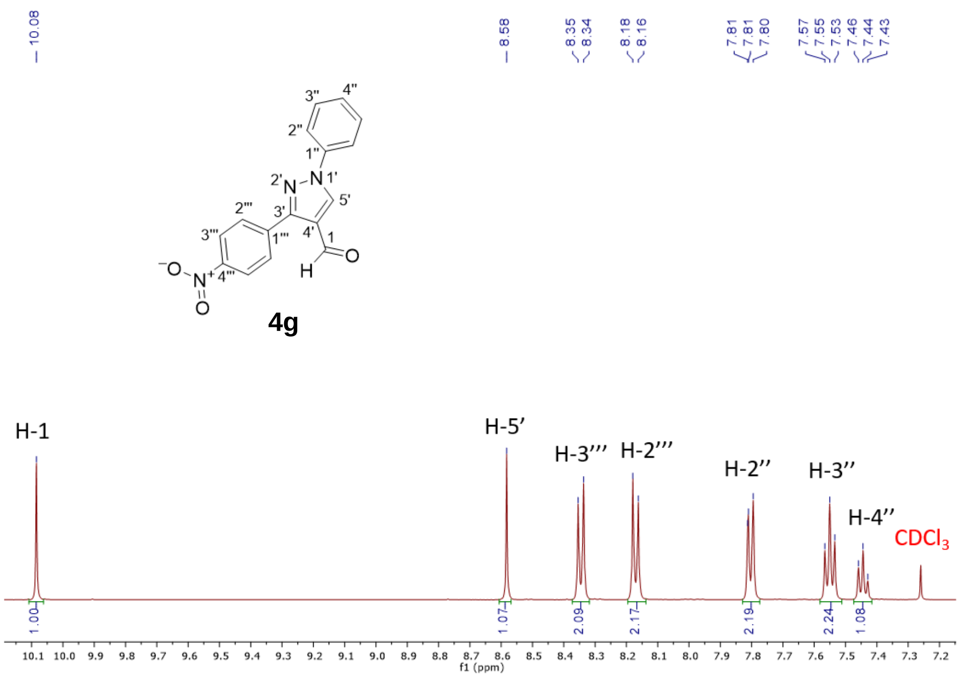

Figure S98. Expansion of ^{1}H NMR (500 MHz, $CDCl_3$) of 3-(4-nitrophenyl)-1-phenyl-1*H*-pyrazole-4-carbaldehyde **4g**.

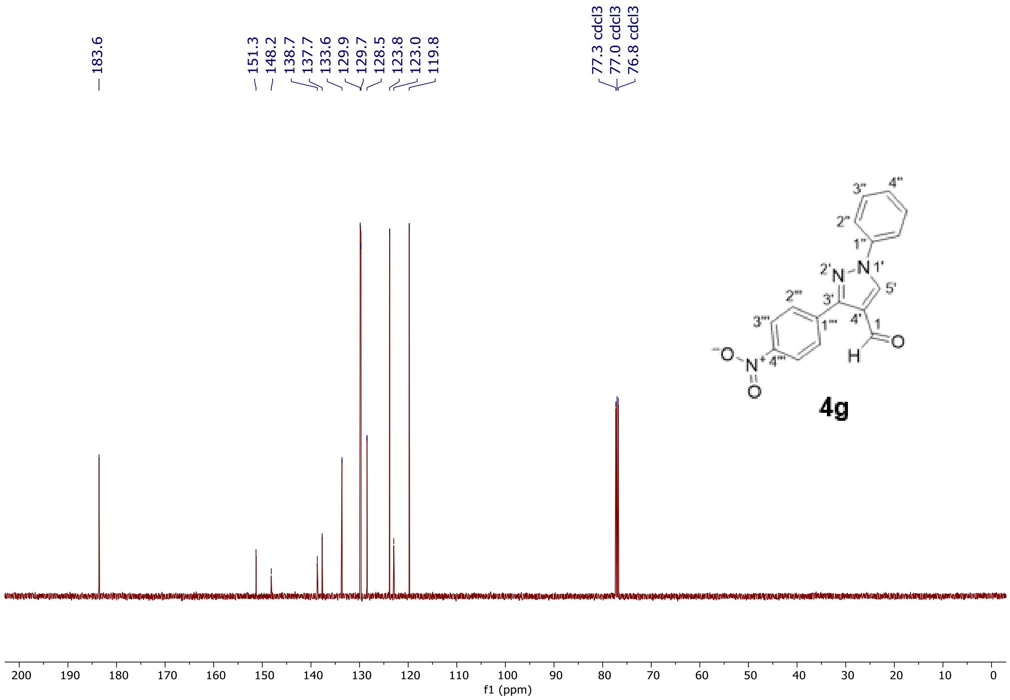

Figure S99. ^{13}C NMR (125 MHz, $CDCl_3$) of 3-(4-nitrophenyl)-1-phenyl-1*H*-pyrazole-4-carbaldehyde **4g**.

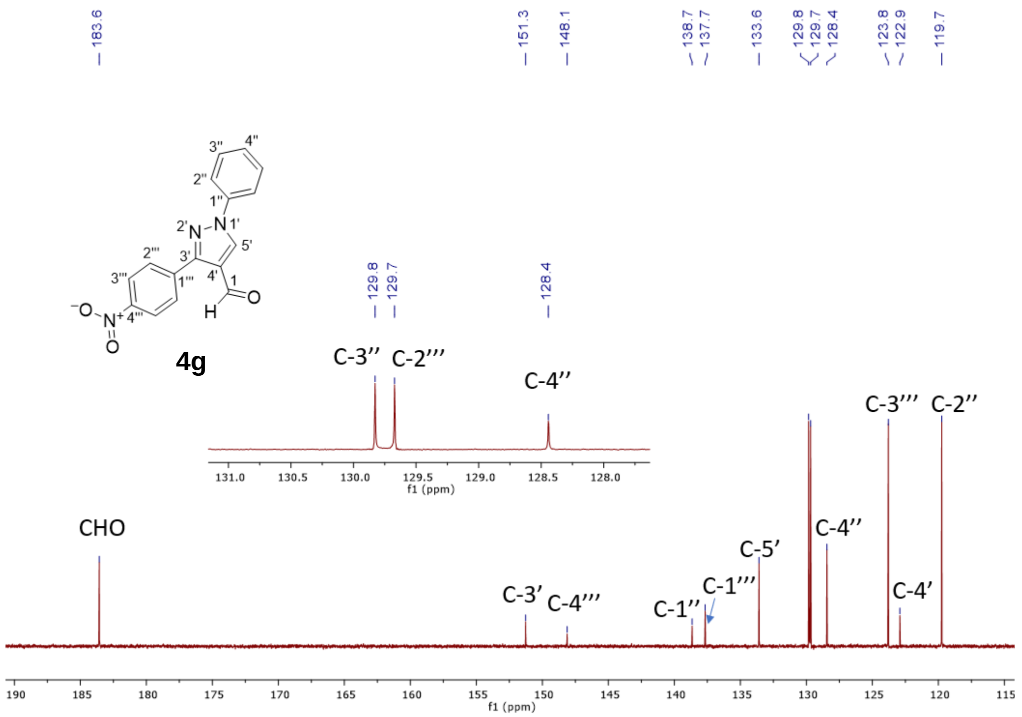

Figure S100. Expansion of ^{13}C NMR (125 MHz, $CDCl_3$) of 3-(4-nitrophenyl)-1-phenyl-1*H*-pyrazole-4-carbaldehyde **4g**.

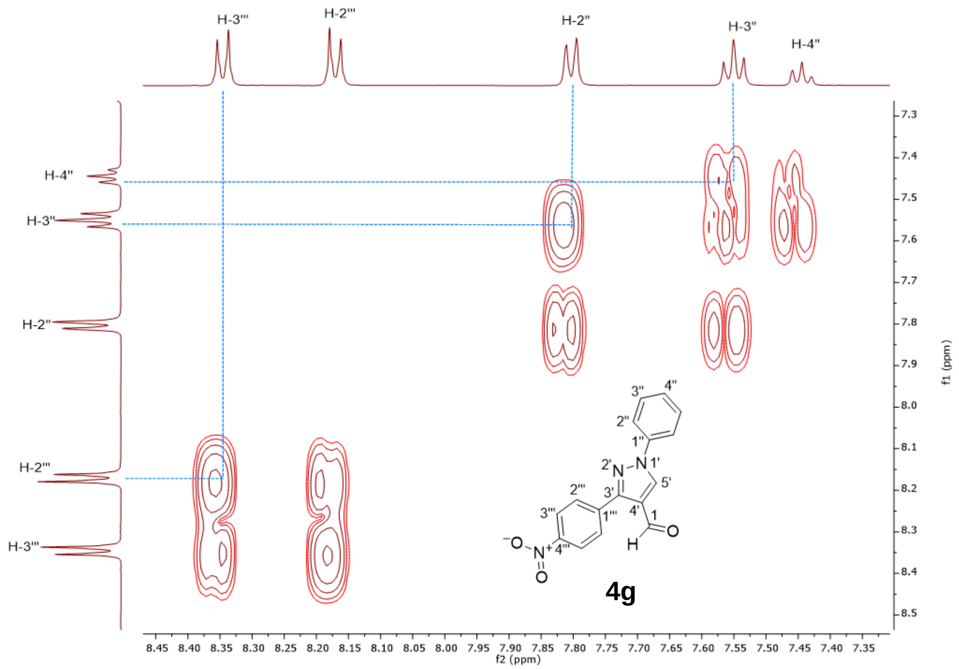

Figure S101. COSY experiment of 3-(4-nitrophenyl)-1-phenyl-1*H*-pyrazole-4-carbaldehyde **4g**.

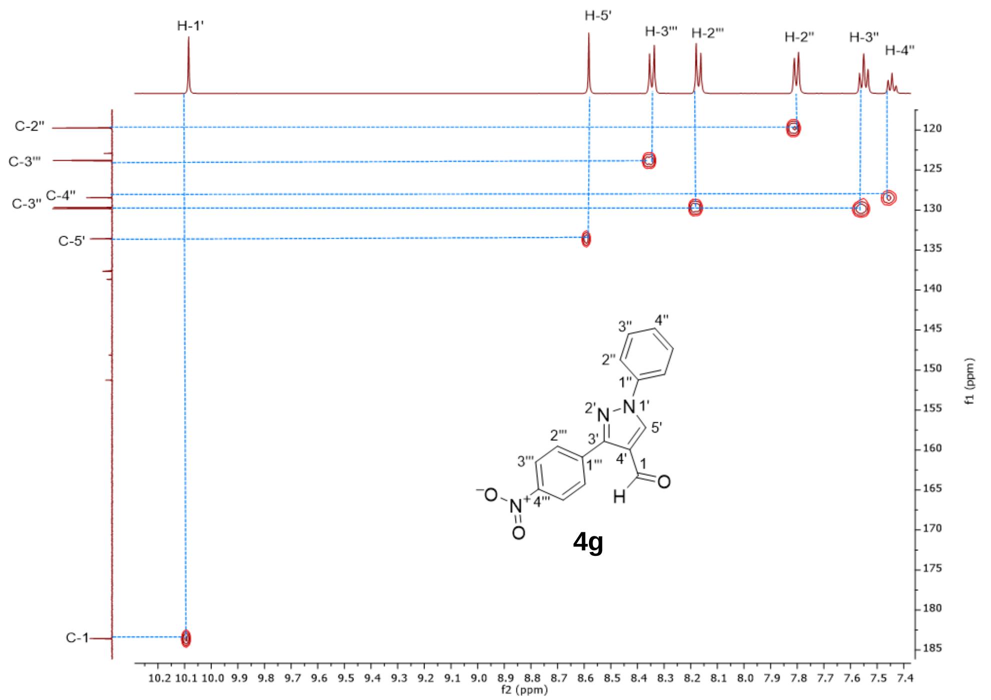

Figure S102. HSQC experiment of 3-(4-nitrophenyl)-1-phenyl-1*H*-pyrazole-4-carbaldehyde **4g**.

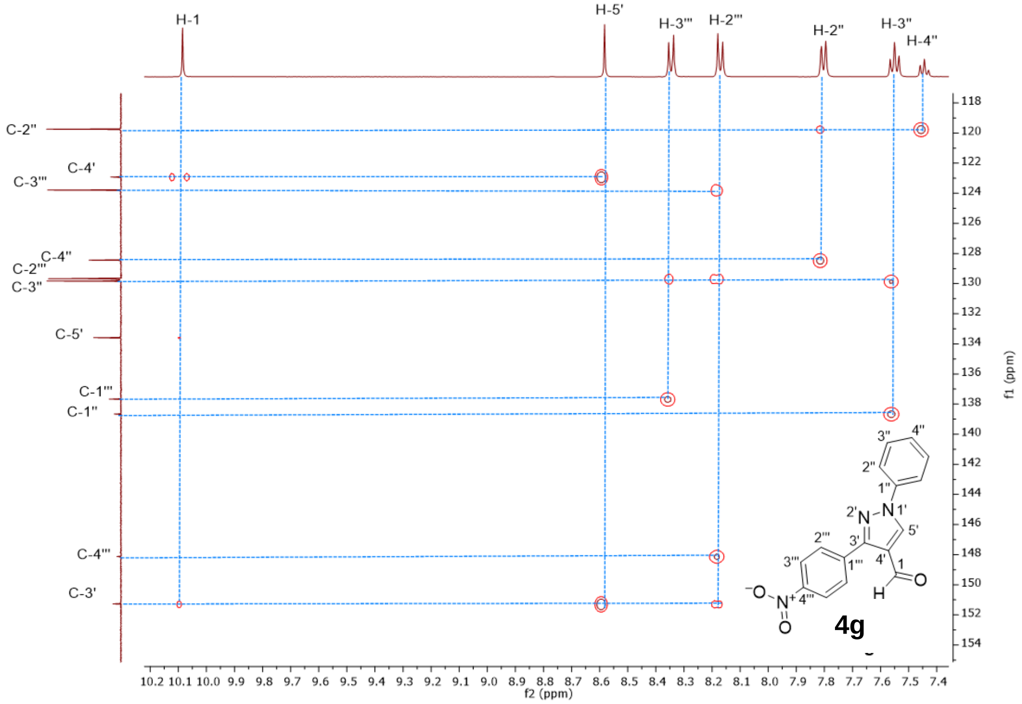

Figure S103. HMBC experiment of 3-(4-nitrophenyl)-1-phenyl-1*H*-pyrazole-4-carbaldehyde **4g**.

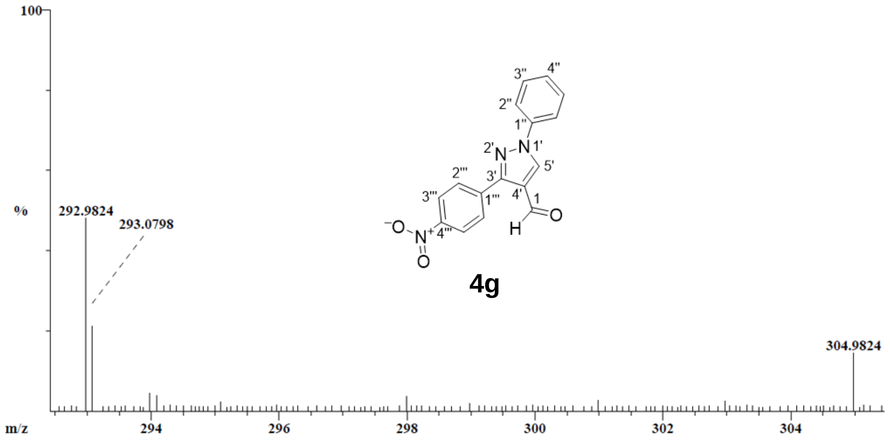

Selected Isotopes : $H_{0-11}C_{0-16}N_{0-3}O_{0-3}$ Error Limit : 5 ppm Unsaturation Limits : 0 to 50

Measured Mass	*% Base*	*Formula*	*Calculated Mass*	*Error*	*Unsaturation*
293.0798	21.3%	$C_{16}H_{11}N_3O_3$	293.0800	-0.8	13.0

Figure S104. HRMS of 3-(4-nitrophenyl)-1-phenyl-1*H*-pyrazole-4-carbaldehyde **4g**.

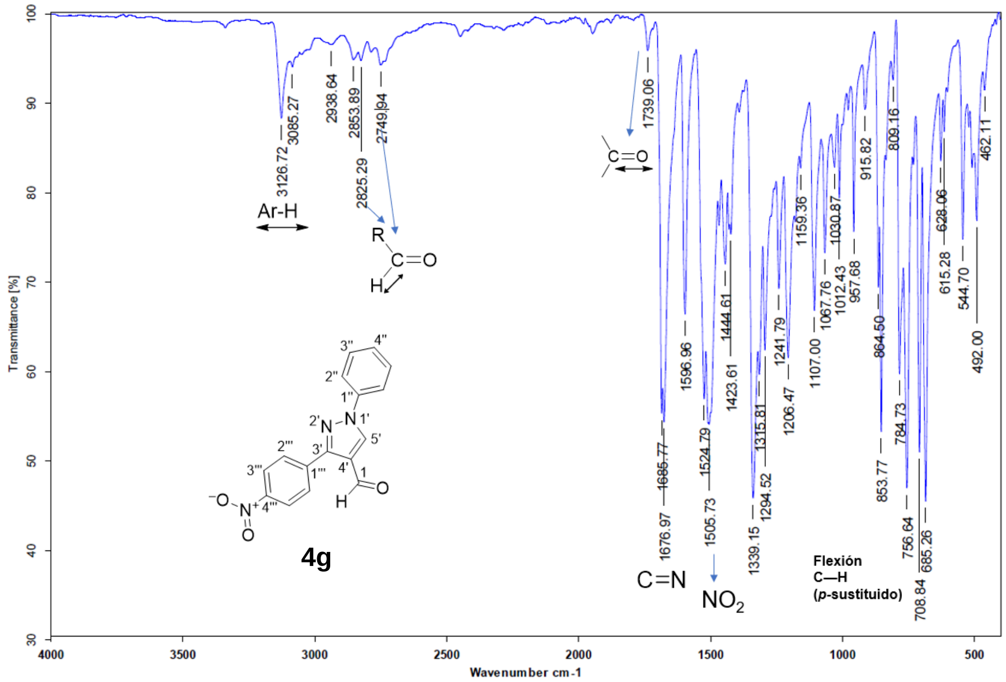

Figure S105. FT-IR of 3-(4-nitrophenyl)-1-phenyl-1*H*-pyrazole-4-carbaldehyde **4g**.

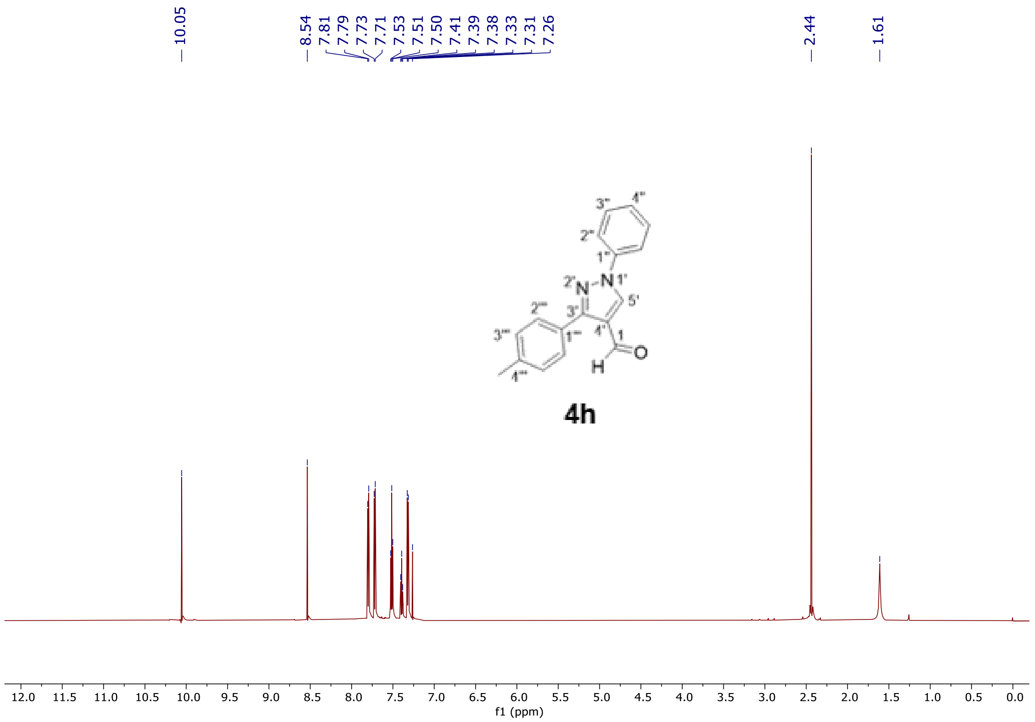

Figure S106. ^{1}H NMR (500 MHz, $CDCl_3$) of 1-phenyl-3-(*p*-tolyl)-1*H*-pyrazole-4-carbaldehyde **4h**.

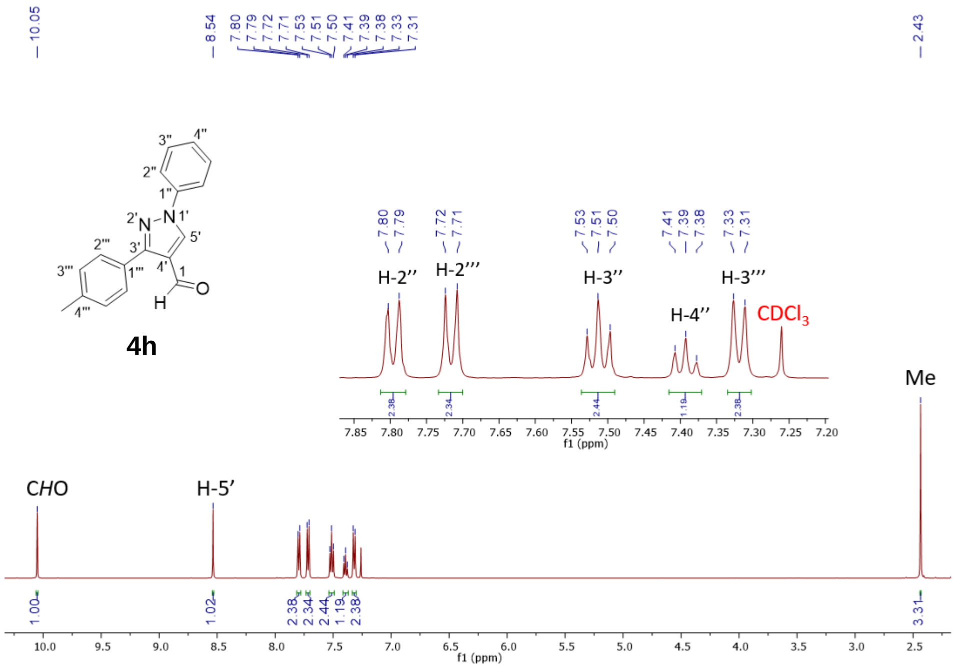

Figure S107. Expansion of ^{1}H NMR (500 MHz, $CDCl_3$) of 1-phenyl-3-(*p*-tolyl)-1*H*-pyrazole-4-carbaldehyde **4h**.

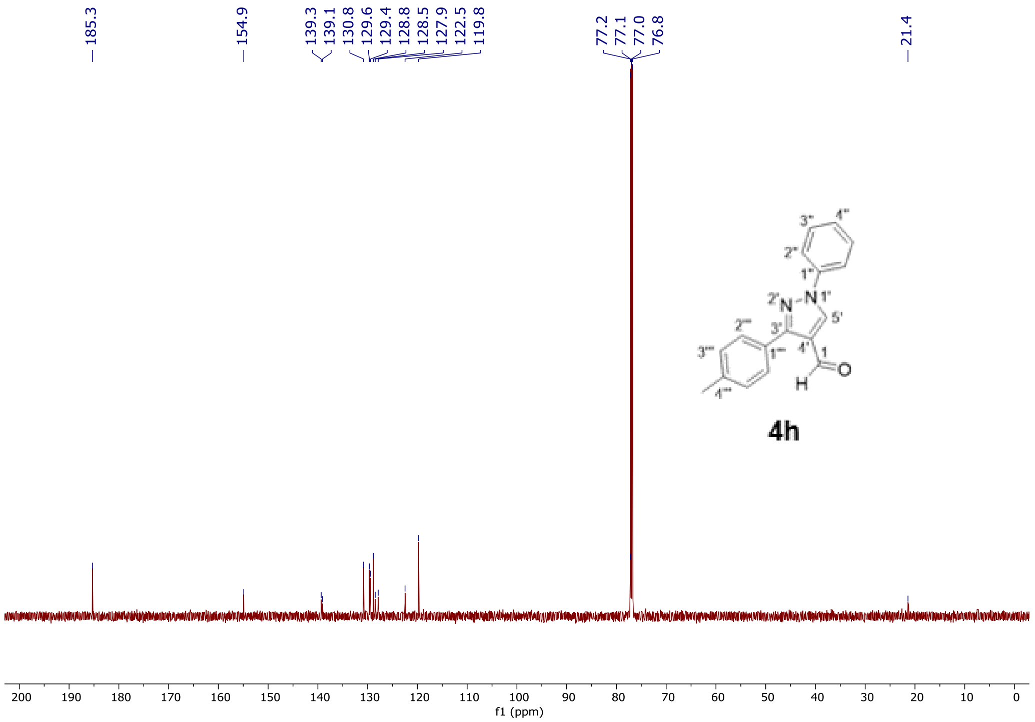

Figure S108. ^{13}C NMR (125 MHz, $CDCl_3$) of 1-phenyl-3-(*p*-tolyl)-1*H*-pyrazole-4-carbaldehyde **4h**.

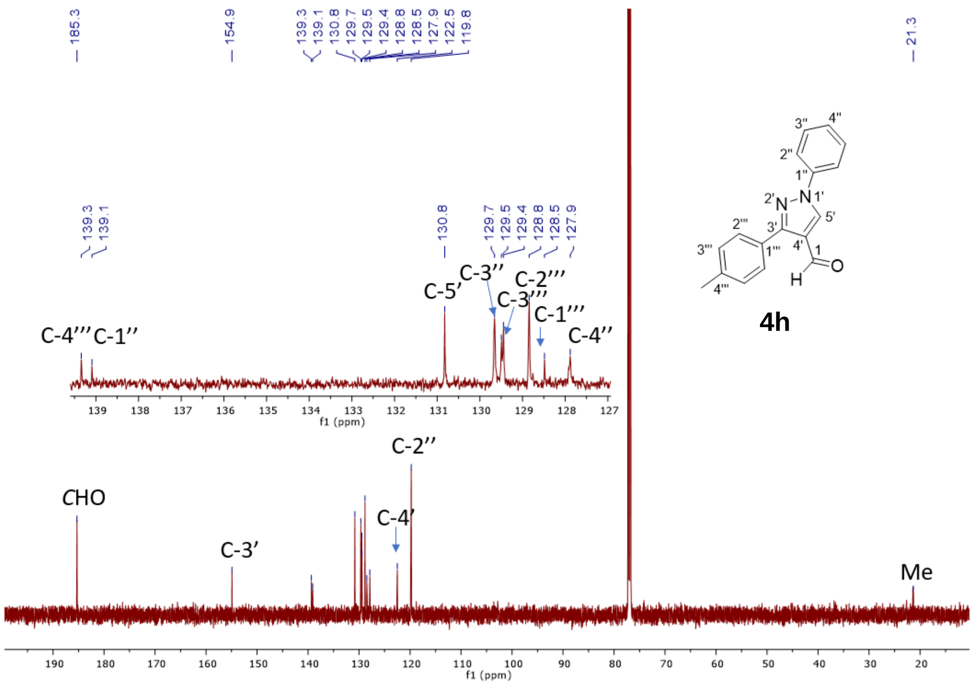

Figure S109. Expansion of ^{13}C NMR (125 MHz, $CDCl_3$) of 1-phenyl-3-(*p*-tolyl)-1*H*-pyrazole-4-carbaldehyde **4h**.

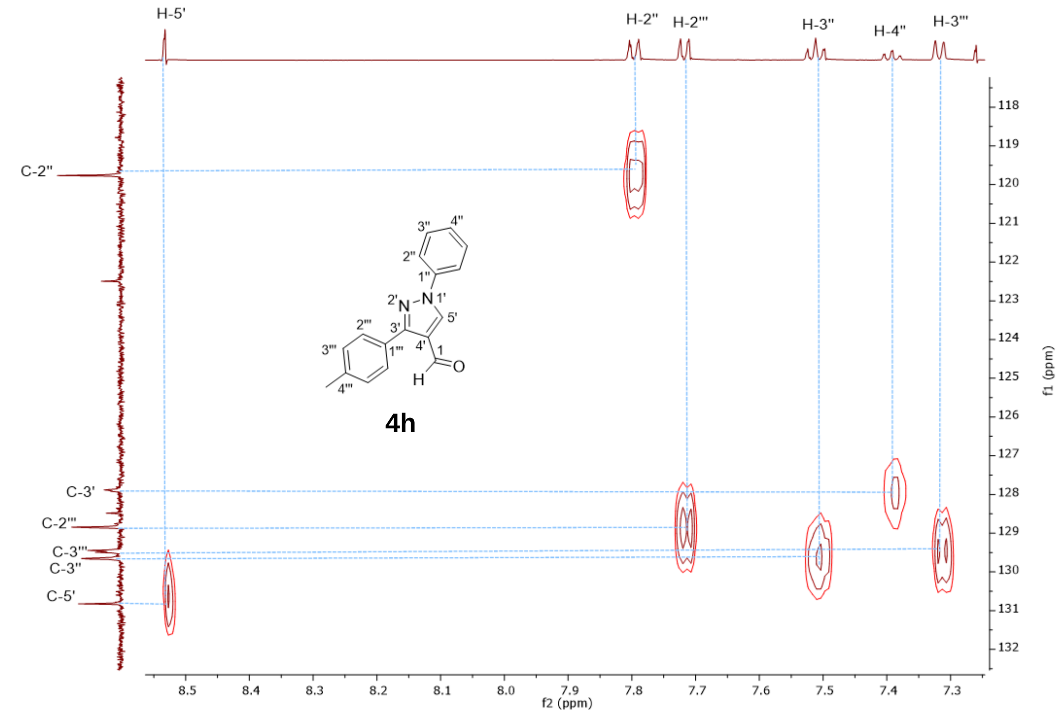

Figure S110. Expansion of HSQC experiment of 1-phenyl-3-(*p*-tolyl)-1*H*-pyrazole-4-carbaldehyde **4h**.

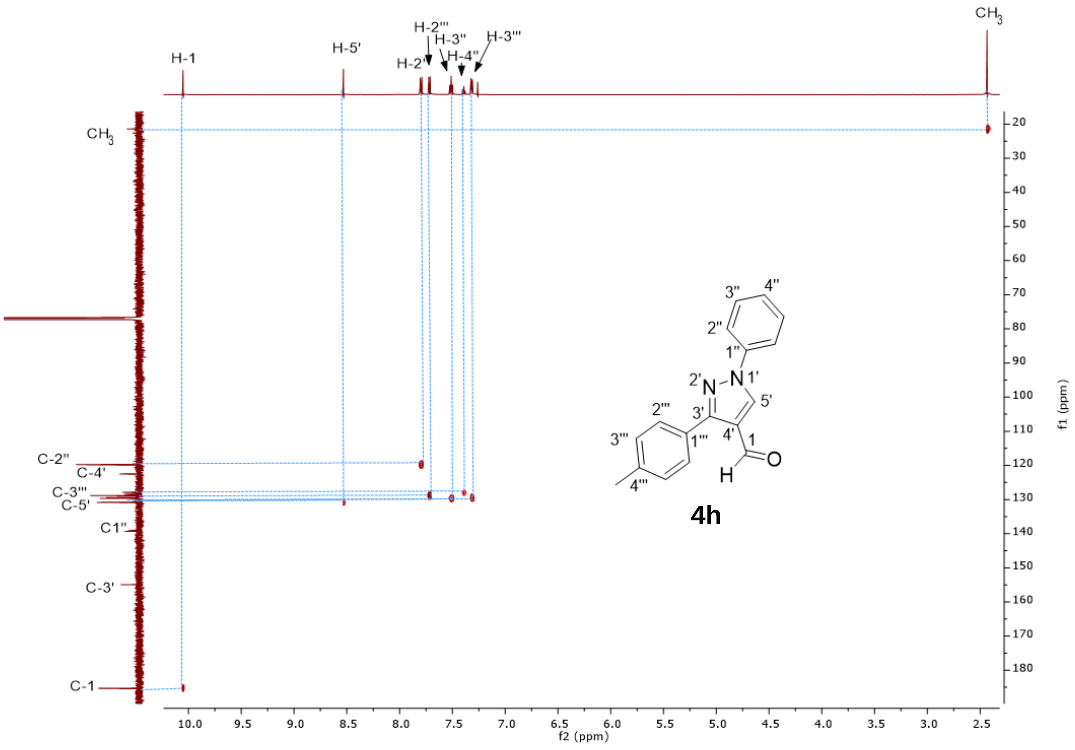

Figure S111. HSQC experiment of 1-phenyl-3-(*p*-tolyl)-1*H*-pyrazole-4-carbaldehyde **4h**.

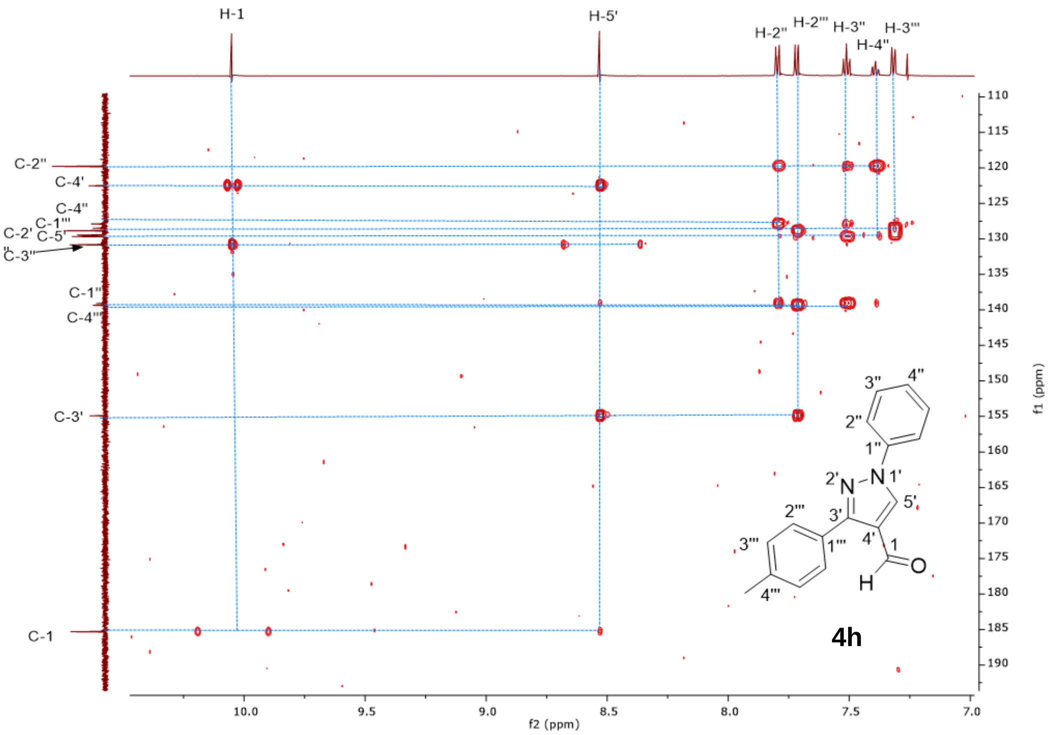

Figure S112. HMBC experiment of 1-phenyl-3-(*p*-tolyl)-1*H*-pyrazole-4-carbaldehyde **4h**.

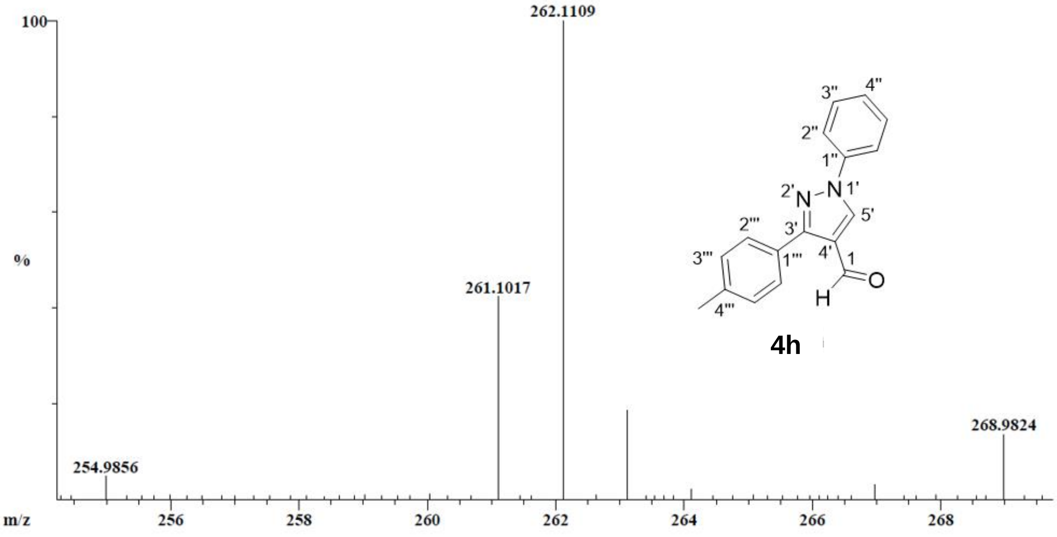

Selected Isotopes : $H_{0-14}C_{0-17}N_{0-2}O_{0-1}$ **Error Limit : 5 ppm** **Unsaturation Limits : 0 to 50**

Measured Mass	% Base	Formula	Calculated Mass	Error	Unsaturation
262.1109	100.0%	$C_{17}H_{14}N_2O$	262.1106	1.1	12.0

Figure S113. HRMS of 1-phenyl-3-(*p*-tolyl)-1*H*-pyrazole-4-carbaldehyde **4h**.

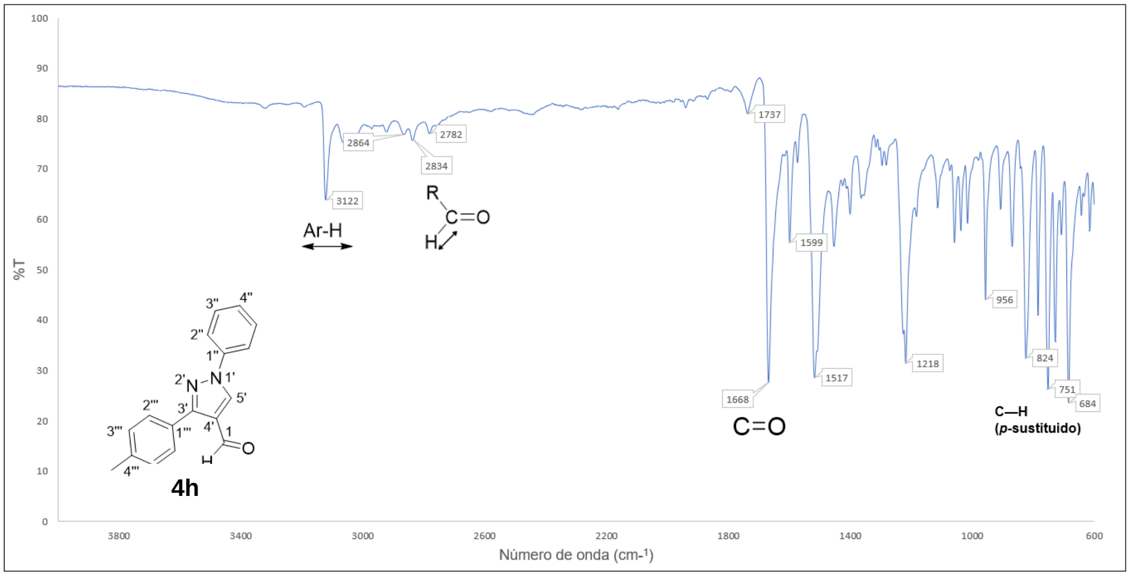

Figure S114. FT-IR of 1-phenyl-3-(*p*-tolyl)-1*H*-pyrazole-4-carbaldehyde **4h**.

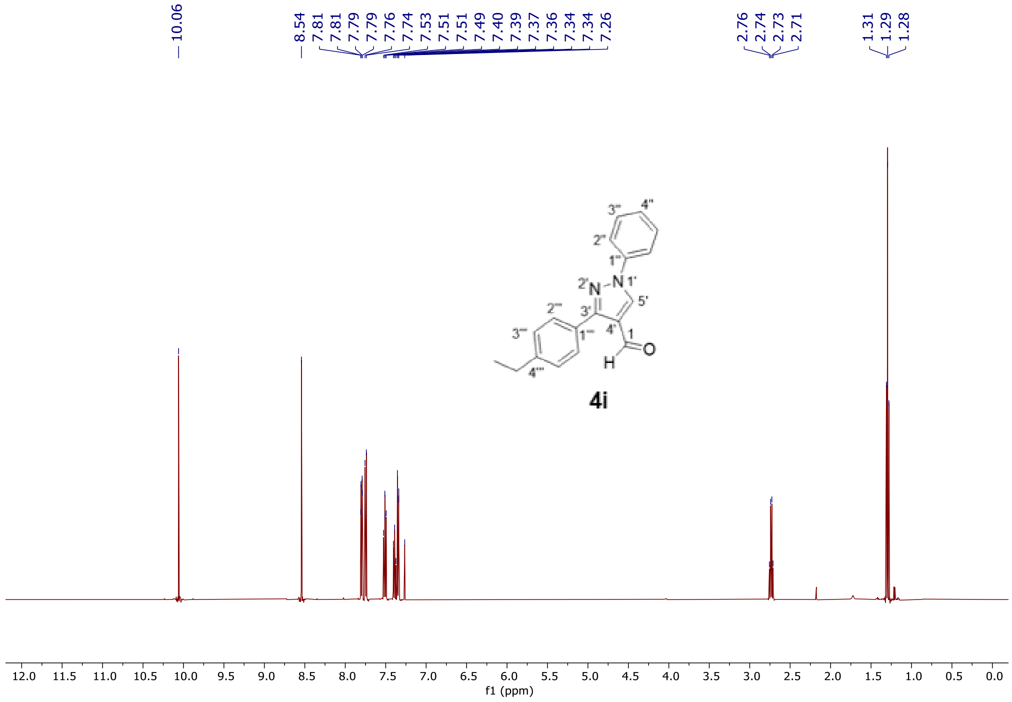

Figure S115. ^{1}H NMR (500 MHz, $CDCl_3$) of 3-(4-ethylphenyl)-1-phenyl-1*H*-pyrazole-4-carbaldehyde **4i**.

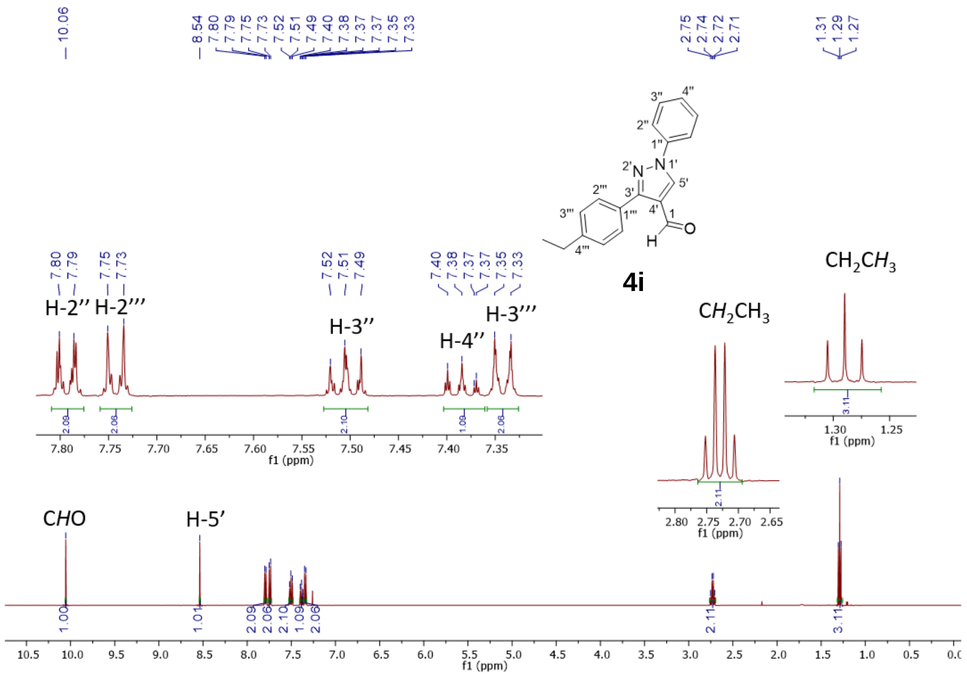

Figure S116. Expansion of ^{1}H NMR (500 MHz, $CDCl_3$) of 3-(4-ethylphenyl)-1-phenyl-1*H*-pyrazole-4-carbaldehyde **4i**.

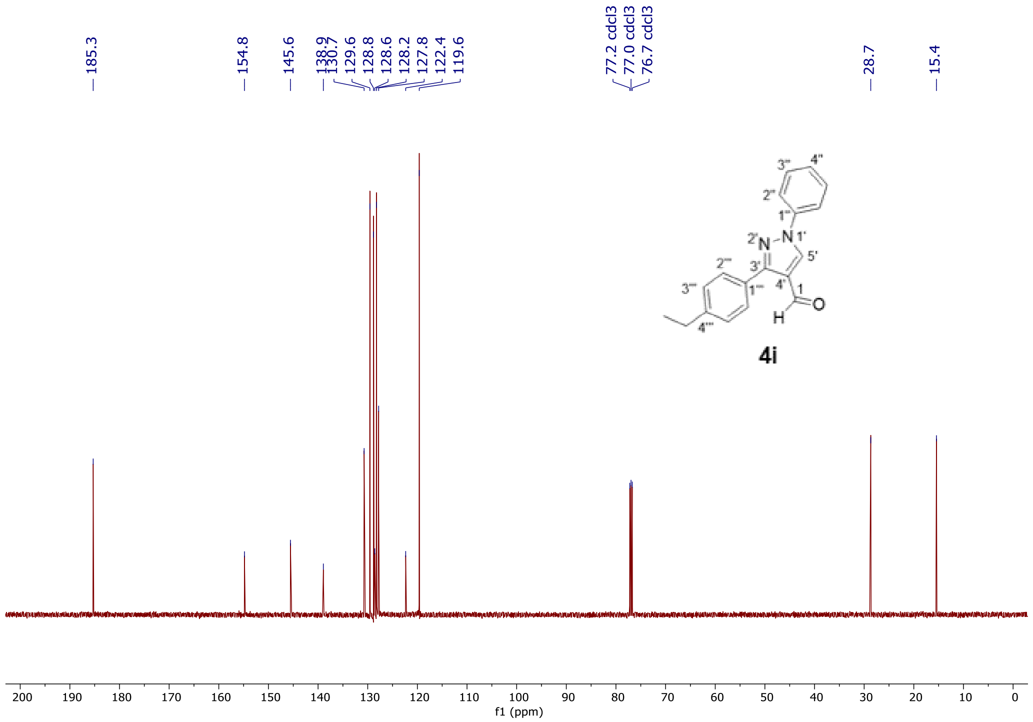

Figure S117. ^{13}C NMR (125 MHz, $CDCl_3$) of 3-(4-ethylphenyl)-1-phenyl-1*H*-pyrazole-4-carbaldehyde **4i**.

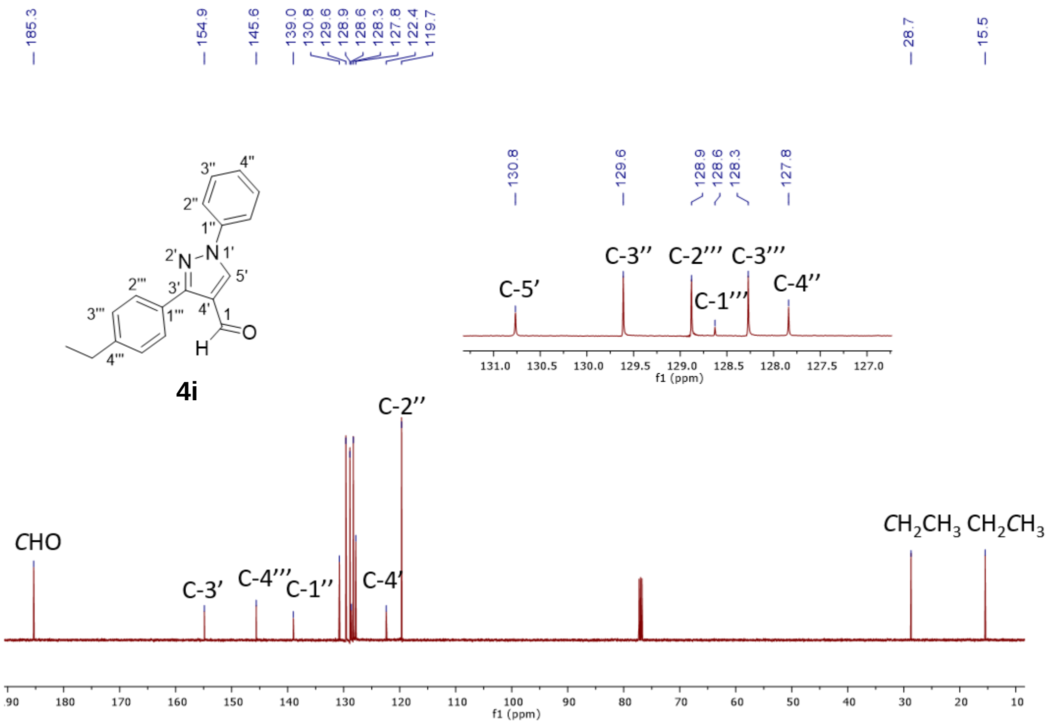

Figure S118. Expansion of ^{13}C NMR (125 MHz, $CDCl_3$) of 3-(4-ethylphenyl)-1-phenyl-1*H*-pyrazole-4-carbaldehyde **4i**.

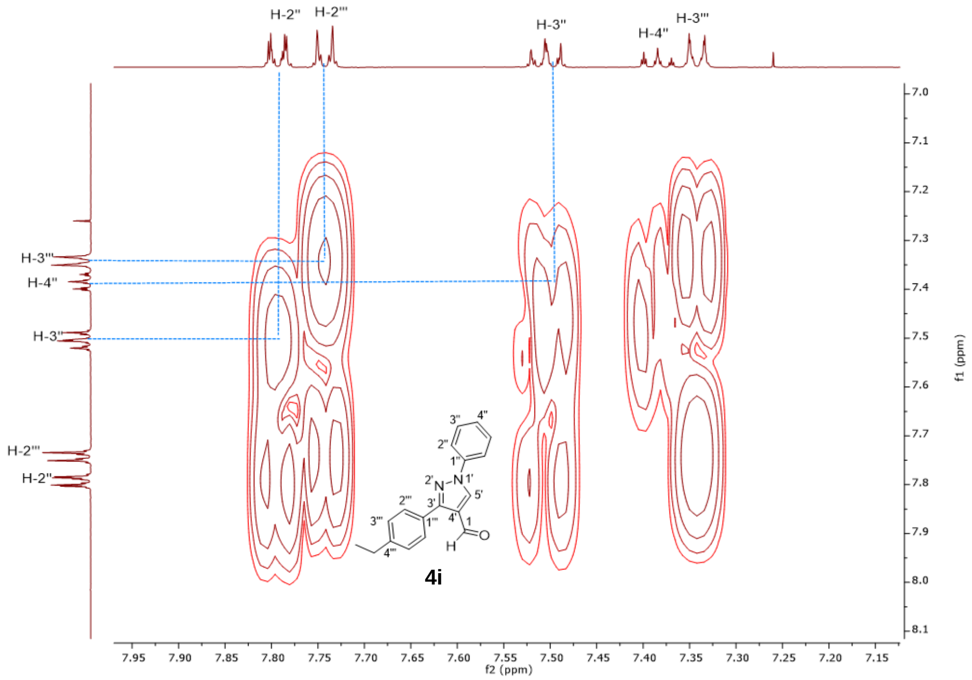

Figure S119. COSY experiment of 3-(4-ethylphenyl)-1-phenyl-1*H*-pyrazole-4-carbaldehyde **4i**.

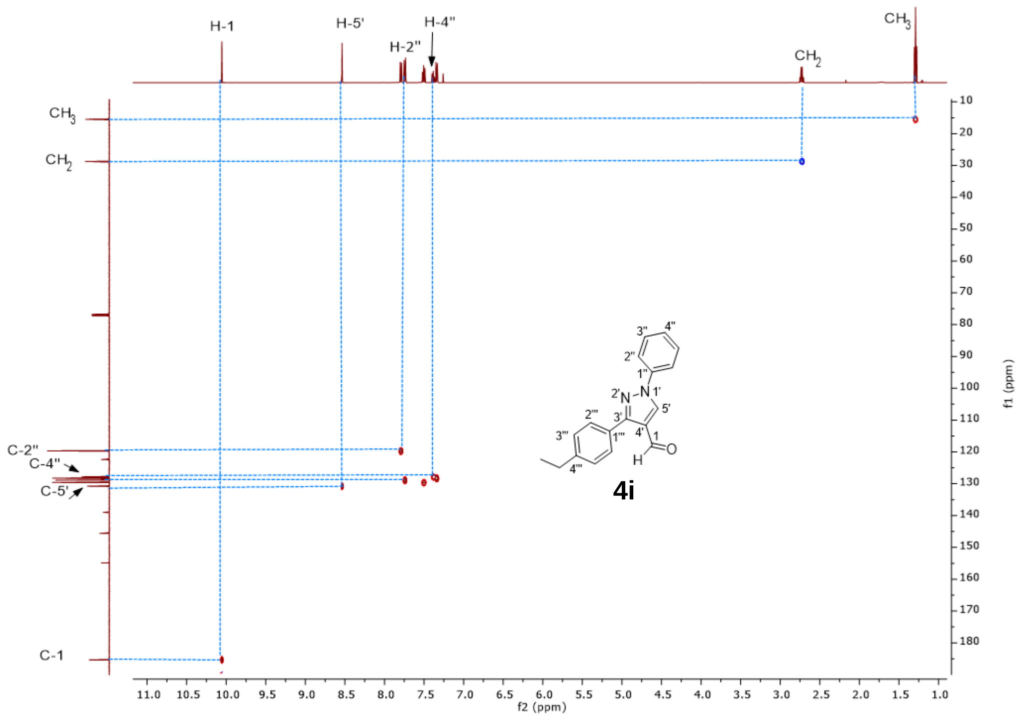

Figure S120. HSQC experiment of 3-(4-ethylphenyl)-1-phenyl-1*H*-pyrazole-4-carbaldehyde **4i**.

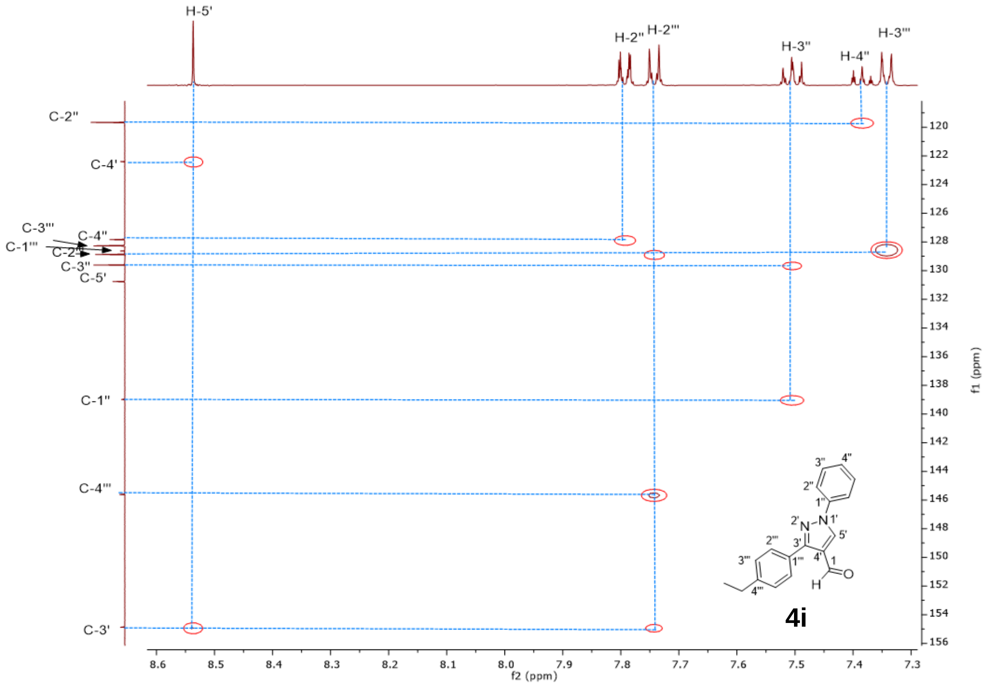

Figure S121. HMBC experiment of 3-(4-ethylphenyl)-1-phenyl-1*H*-pyrazole-4-carbaldehyde **4i**.

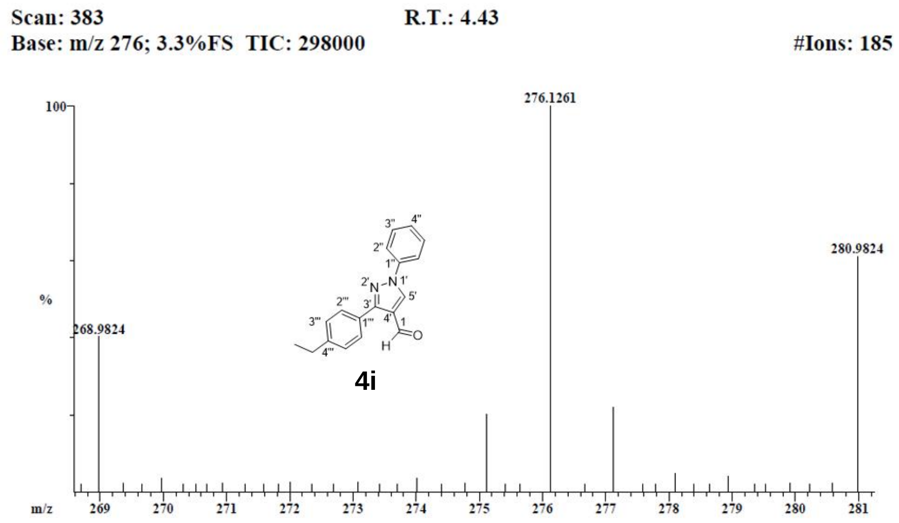

Selected Isotopes : $H_{0-16}C_{0-18}N_{0-2}O_{0-1}$ Error Limit : 5 ppm Unsaturation Limits : 0 to 50

Measured Mass	*% Base*	*Formula*	*Calculated Mass*	*Error*	*Unsaturation*
276.1261	100.0%	$C_{18}H_{16}N_2O$	276.1263	-0.6	12.0

Figure S122. HRMS of 3-(4-ethylphenyl)-1-phenyl-1*H*-pyrazole-4-carbaldehyde **4i**.

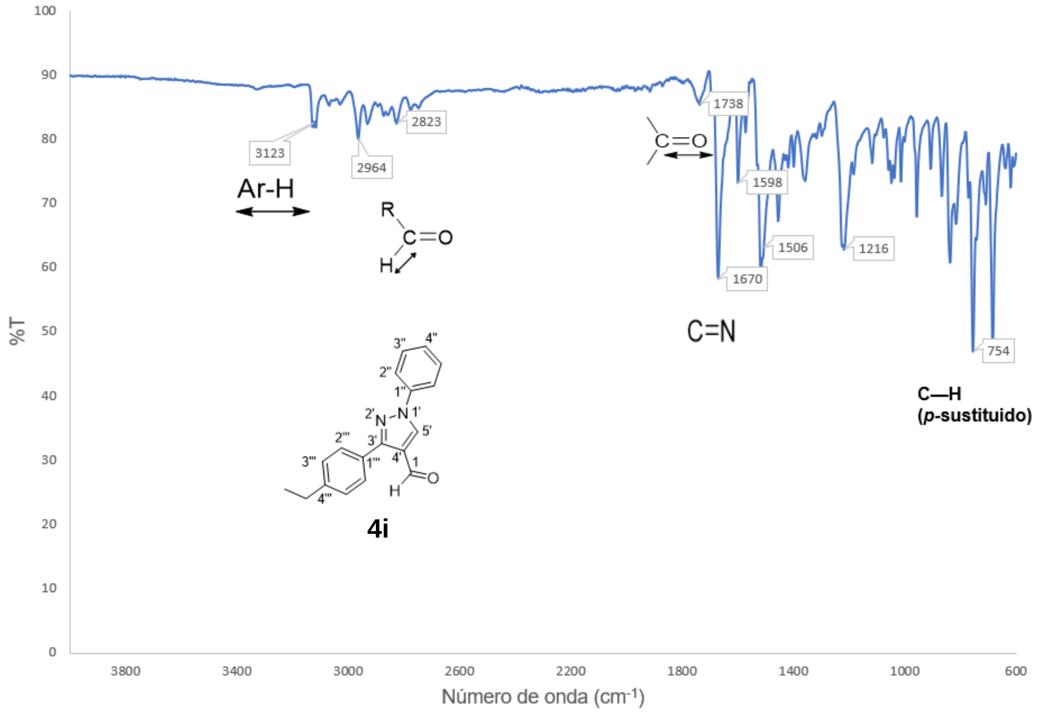

Figure S123. FT-IR of 3-(4-ethylphenyl)-1-phenyl-1*H*-pyrazole-4-carbaldehyde **4i**.

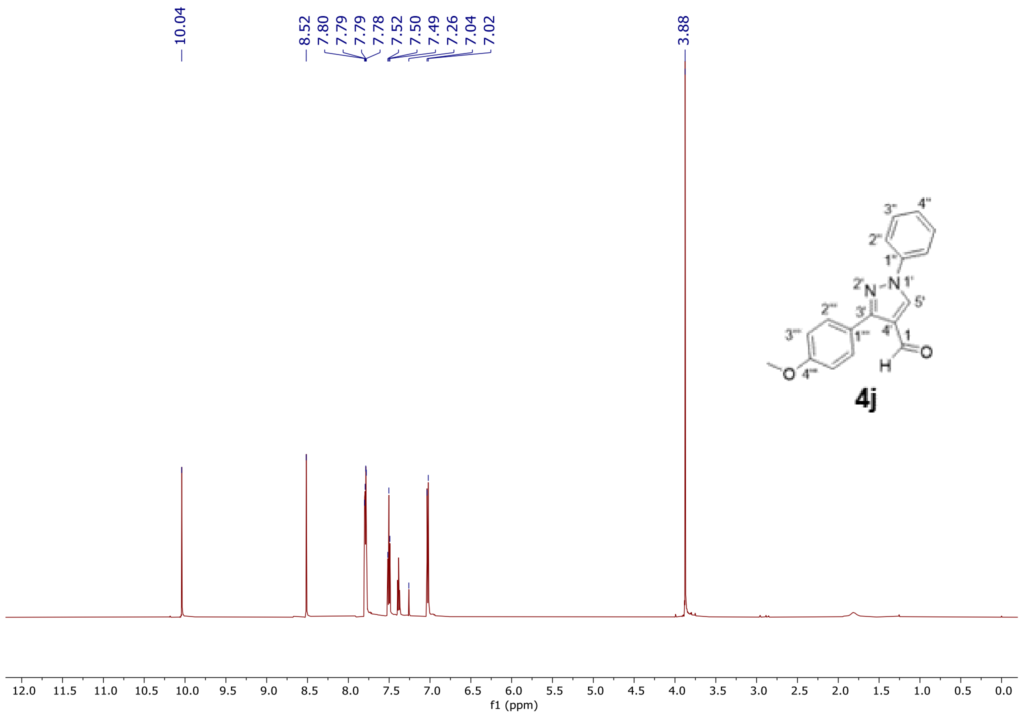

Figure S124. ^{1}H NMR (500 MHz, $CDCl_3$) of 3-(4-methoxyphenyl)-1-phenyl-1*H*-pyrazole-4-carbaldehyde **4j**.

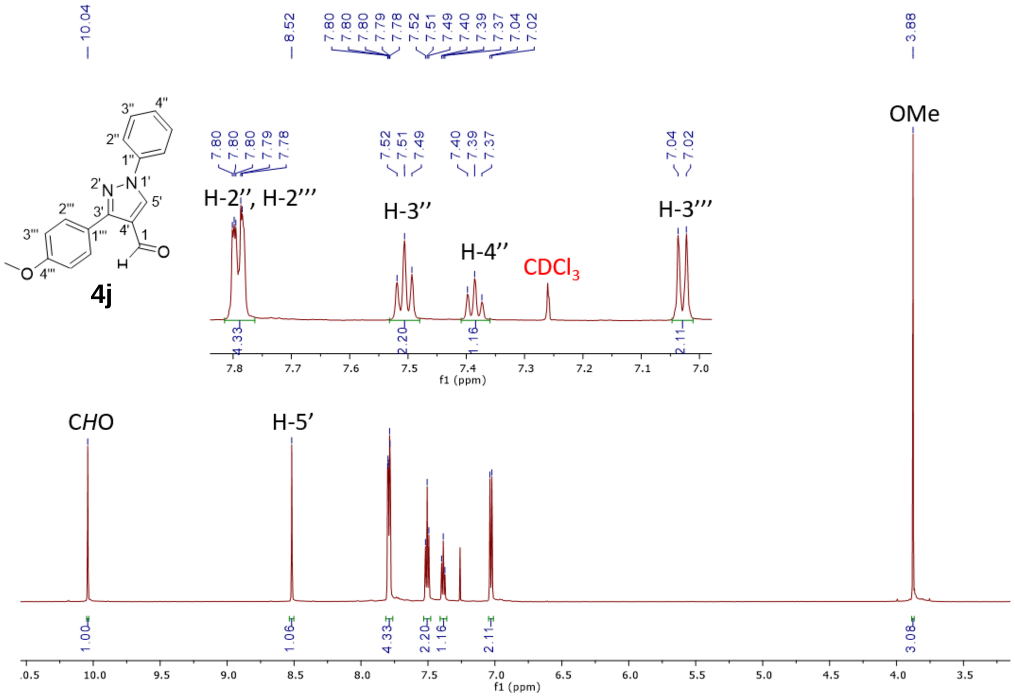

Figure S125. Expansion of ^{1}H NMR (500 MHz, $CDCl_3$) of 3-(4-methoxyphenyl)-1-phenyl-1*H*-pyrazole-4-carbaldehyde **4j**.

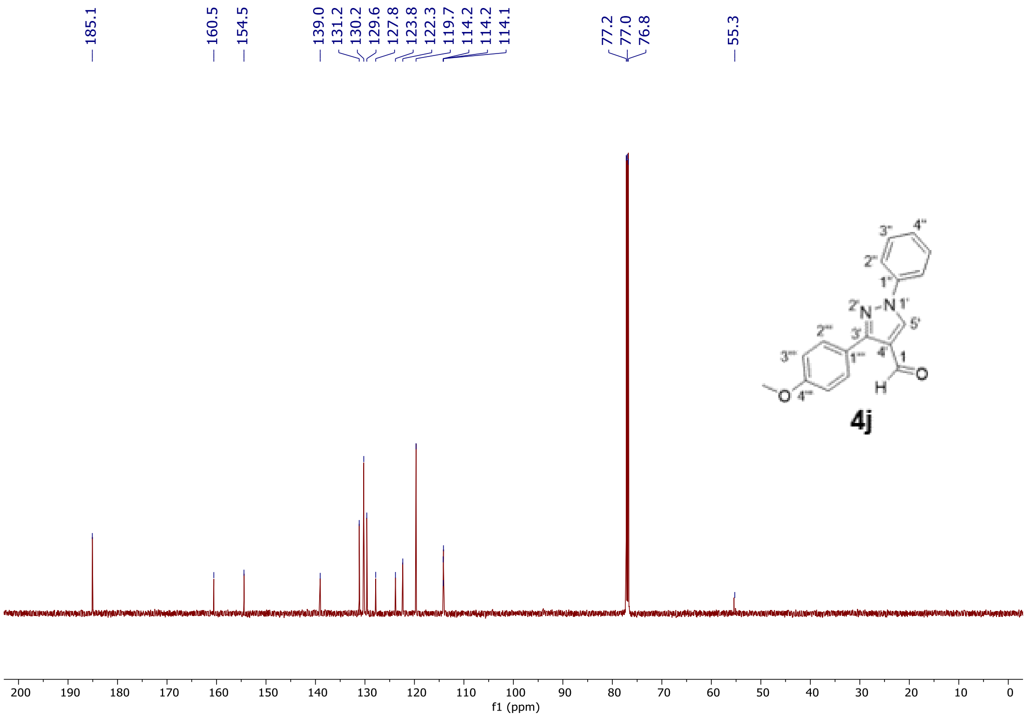

Figure S126. ^{13}C NMR (125 MHz, $CDCl_3$) of 3-(4-methoxyphenyl)-1-phenyl-1*H*-pyrazole-4-carbaldehyde **4j**.

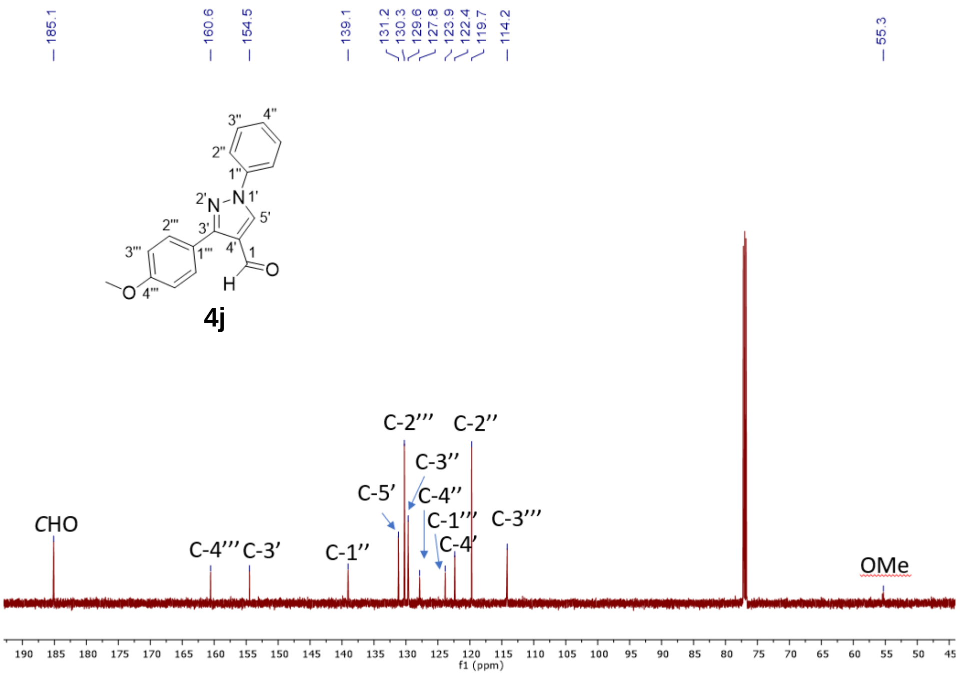

Figure S127. Expansion of ^{13}C NMR (125 MHz, $CDCl_3$) of 3-(4-methoxyphenyl)-1-phenyl-1*H*-pyrazole-4-carbaldehyde **4j**.

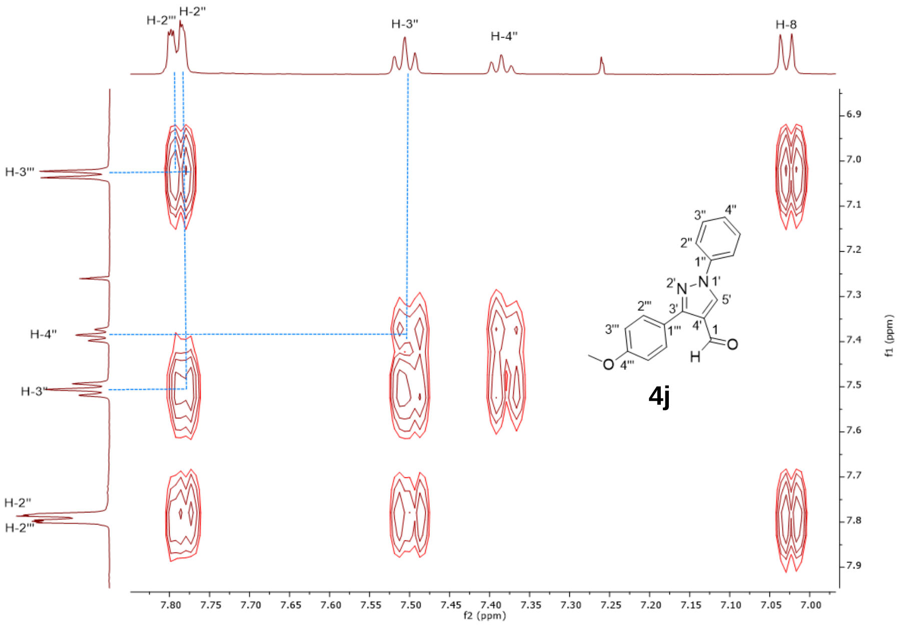

Figure S128. COSY experiment of 3-(4-methoxyphenyl)-1-phenyl-1*H*-pyrazole-4-carbaldehyde **4j**.

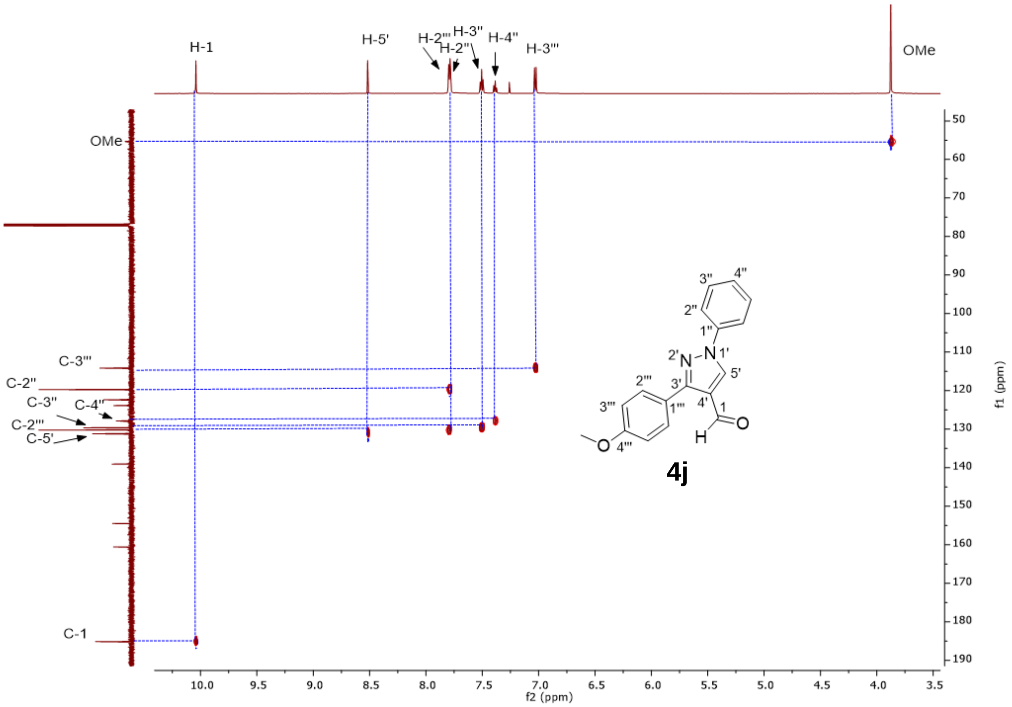

Figure S129. HSQC experiment of 3-(4-methoxyphenyl)-1-phenyl-1*H*-pyrazole-4-carbaldehyde **4j**.

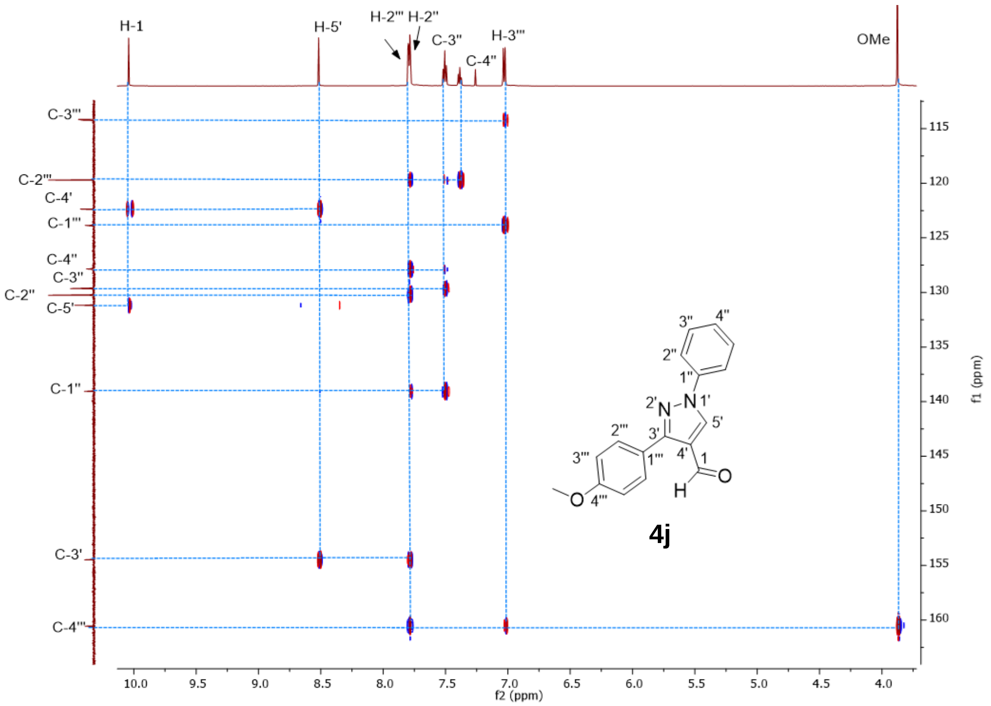

Figure S130. HMBC experiment of 3-(4-methoxyphenyl)-1-phenyl-1*H*-pyrazole-4-carbaldehyde **4j**.

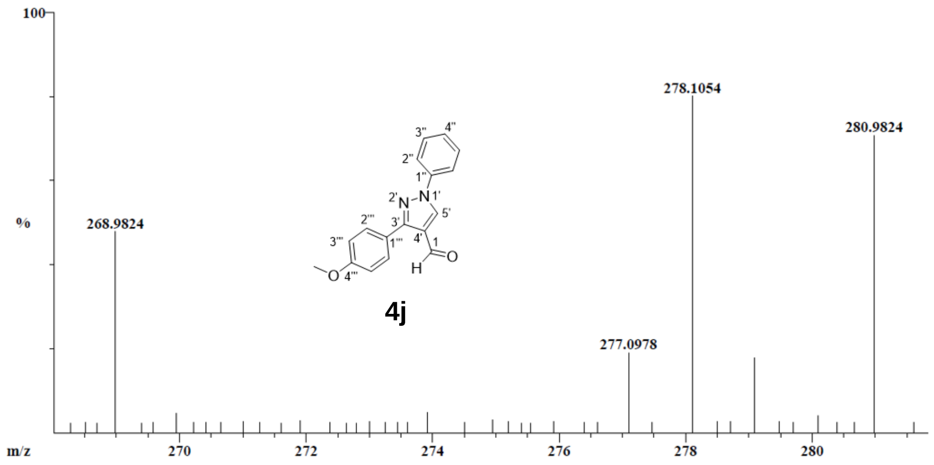

Selected Isotopes : $H_{0-14}C_{0-17}N_{0-2}O_{0-2}$ Error Limit : 5 ppm Unsaturation Limits : 0 to 50

Measured Mass	% Base	Formula	Calculated Mass	Error	Unsaturation
278.1054	80.0%	$C_{17}H_{14}N_2O_2$	278.1055	-0.5	12.0

Figure S131. HRMS of 3-(4-methoxyphenyl)-1-phenyl-1*H*-pyrazole-4-carbaldehyde **4j**.

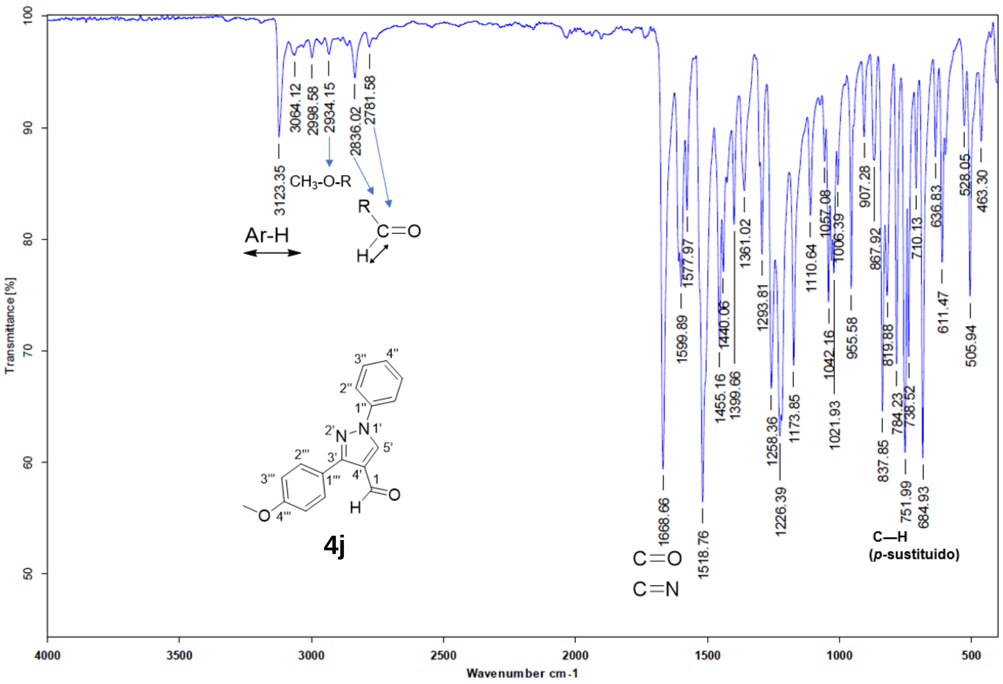

Figure S132. FT-IR of 3-(4-methoxyphenyl)-1-phenyl-1*H*-pyrazole-4-carbaldehyde **4j**.

Characterization of series **6a-j**

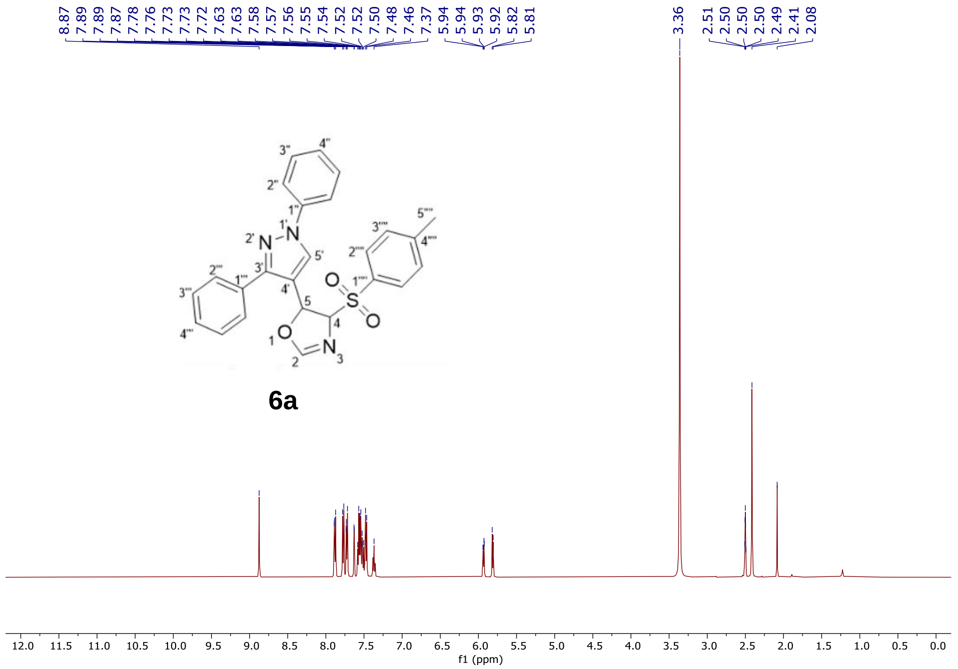

Figure S133. ^{1}H NMR (500 MHz, DMSO-d$_6$) of (4*S**, 5*S**)-5-(1,3-diphenyl-1*H*-pyrazol-4-yl)-4-tosyl-4,5-dihydrooxazole **6a**.

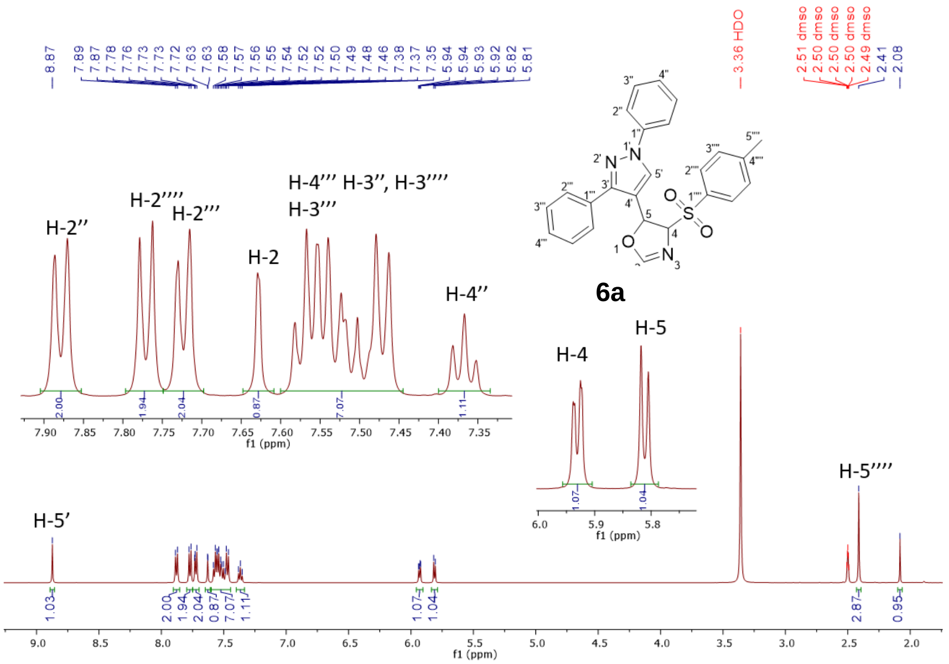

Figure S134. Expansion of ^{1}H NMR (500 MHz, DMSO-d$_6$) of (4*S**, 5*S**)-5-(1,3-diphenyl-1*H*-pyrazol-4-yl)-4-tosyl-4,5-dihydrooxazole **6a**.

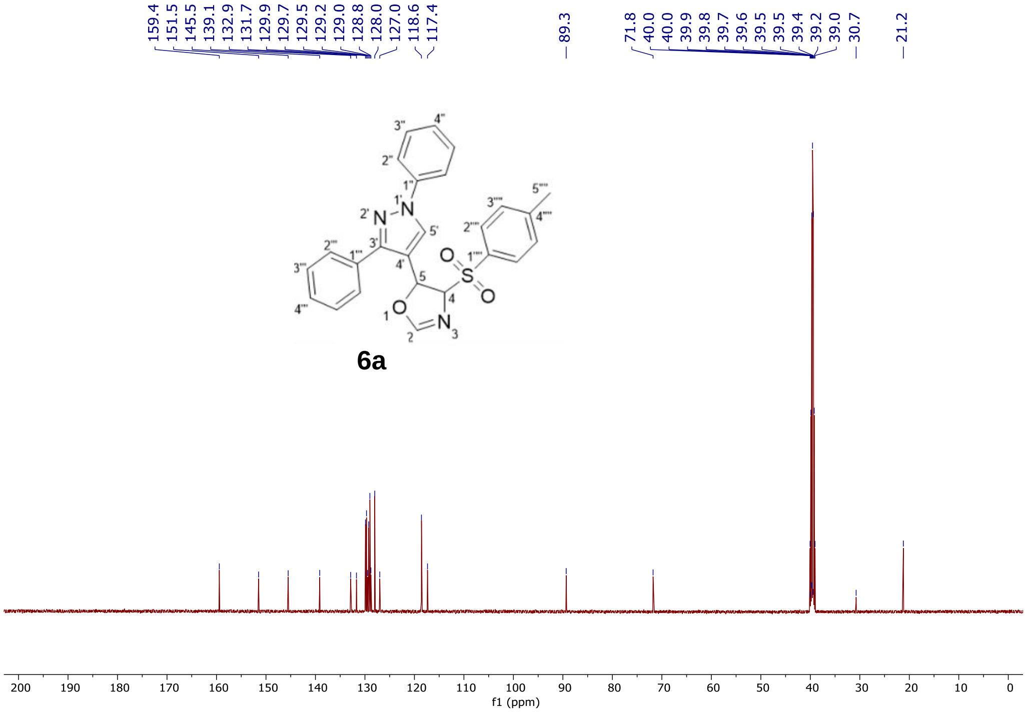

Figure S135. ^{13}C NMR (125 MHz, DMSO-d$_6$) of (4*S**, 5*S**)-5-(1,3-diphenyl-1*H*-pyrazol-4-yl)-4-tosyl-4,5-dihydrooxazole **6a**.

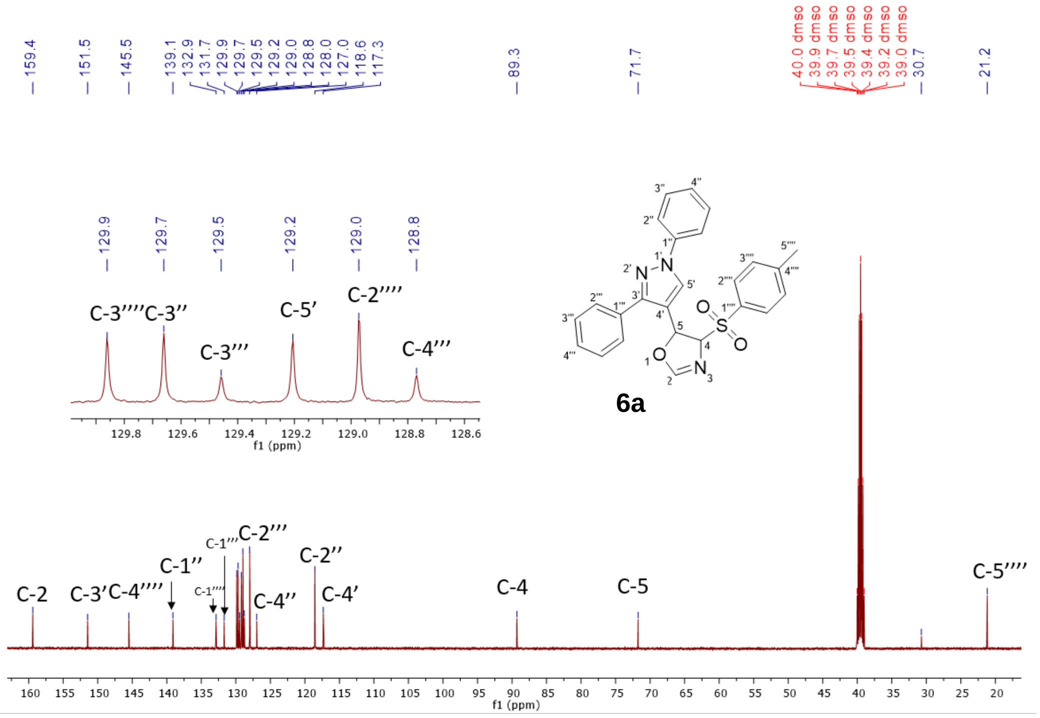

Figure S136. Expansion of ^{13}C NMR (125 MHz, DMSO-d$_6$) of (4*S**, 5*S**)-5-(1,3-diphenyl-1*H*-pyrazol-4-yl)-4-tosyl-4,5-dihydrooxazole **6a**.

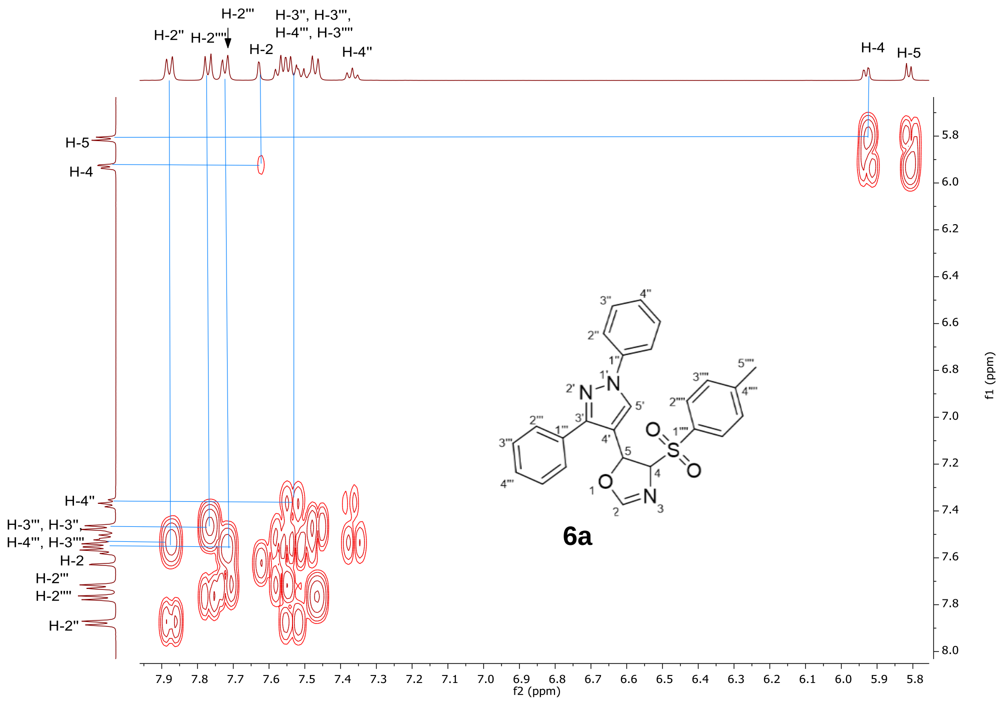

Figure S137. COSY experiment of (4*S**, 5*S**)-5-(1,3-diphenyl-1*H*-pyrazol-4-yl)-4-tosyl-4,5-dihydrooxazole **6a**.

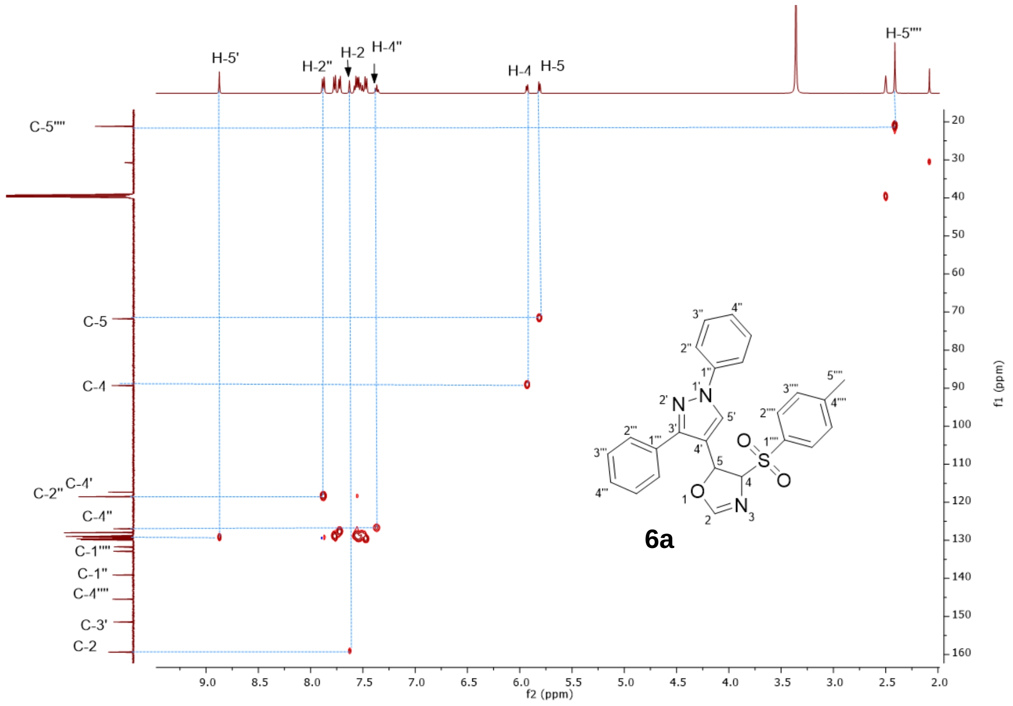

Figure S138. HSQC experiment of (4*S**, 5*S**)-5-(1,3-diphenyl-1*H*-pyrazol-4-yl)-4-tosyl-4,5-dihydrooxazole **6a**.

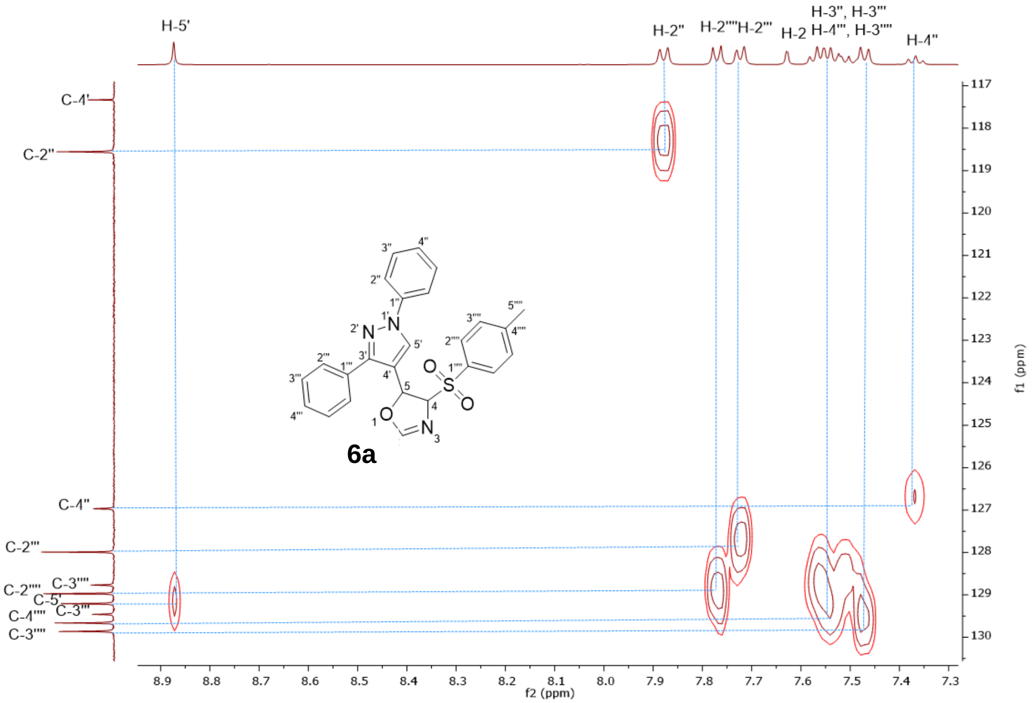

Figure S139. Expansion of HSQC experiment of (4*S**, 5*S**)-5-(1,3-diphenyl-1*H*-pyrazol-4-yl)-4-tosyl-4,5-dihydrooxazole **6a**.

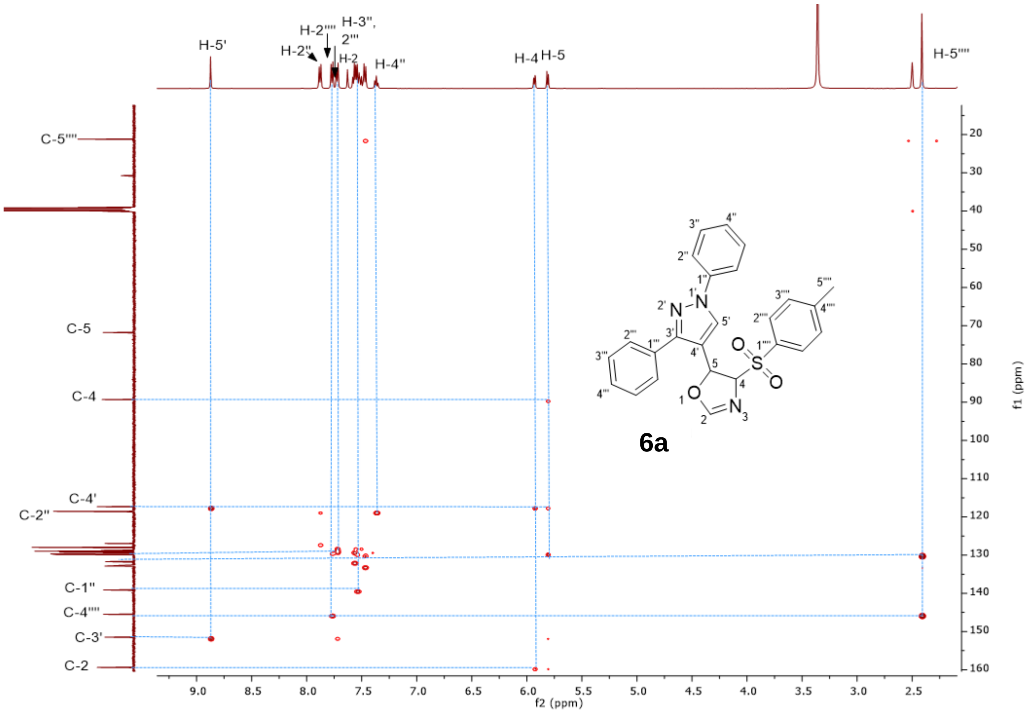

Figure S140. HMBC experiment of (4*S**, 5*S**)-5-(1,3-diphenyl-1*H*-pyrazol-4-yl)-4-tosyl-4,5-dihydrooxazole **6a**.

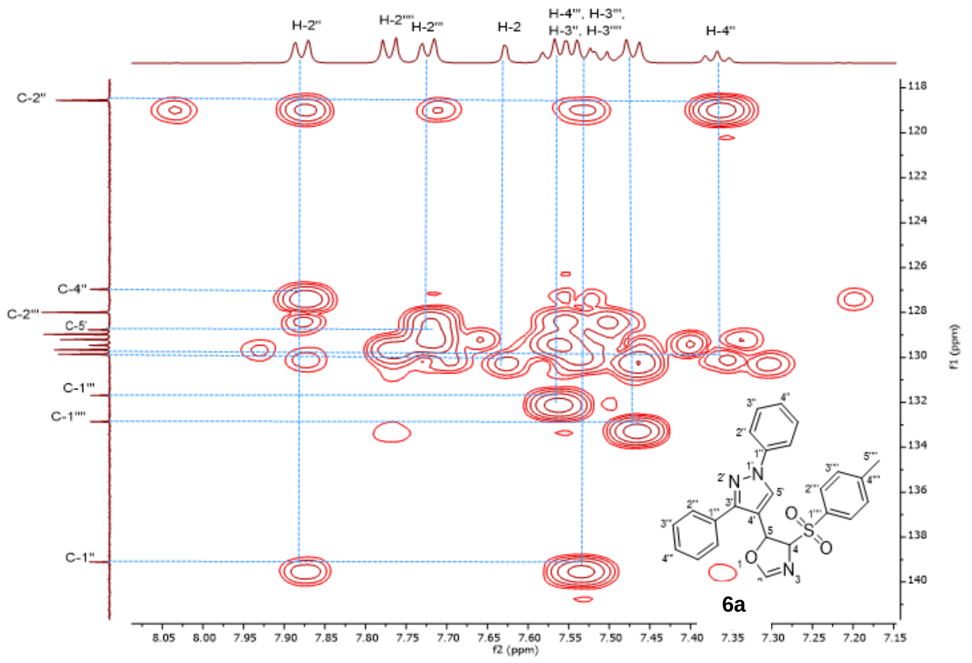

Figure S141. Expansion of HMBC experiment of (4*S**, 5*S**)-5-(1,3-diphenyl-1*H*-pyrazol-4-yl)-4-tosyl-4,5-dihydrooxazole **6a**.

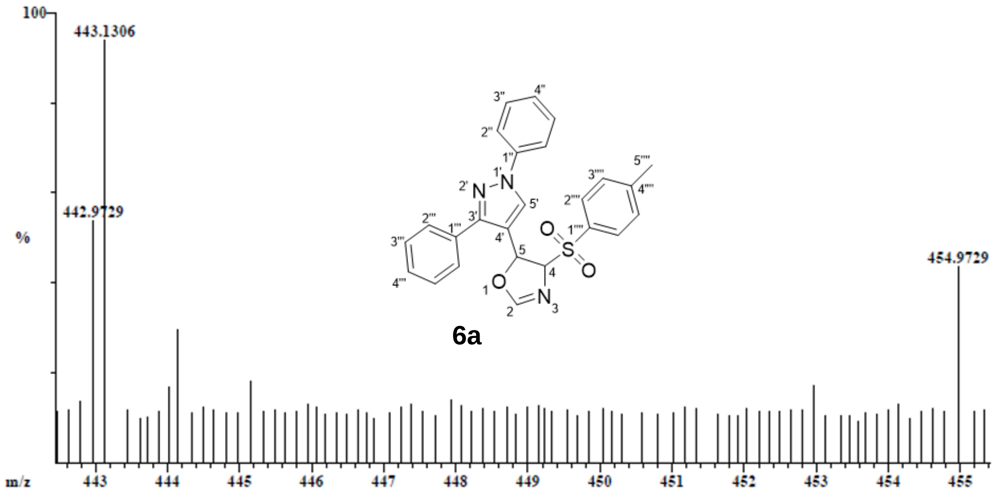

Selected Isotopes : $H_{0\text{-}21}C_{0\text{-}25}N_{0\text{-}3}O_{0\text{-}3}S_{0\text{-}1}$ Error Limit : 5 ppm Unsaturation Limits : 0 to 50

Measured Mass	% Base	Formula	Calculated Mass	Error	Unsaturation
443.1306	93.9%	$C_{25}H_{21}N_3O_3S$	443.1304	0.5	17.0

Figure S142. HRMS of (4*S**, 5*S**)-5-(1,3-diphenyl-1*H*-pyrazol-4-yl)-4-tosyl-4,5-dihydrooxazole **6a**.

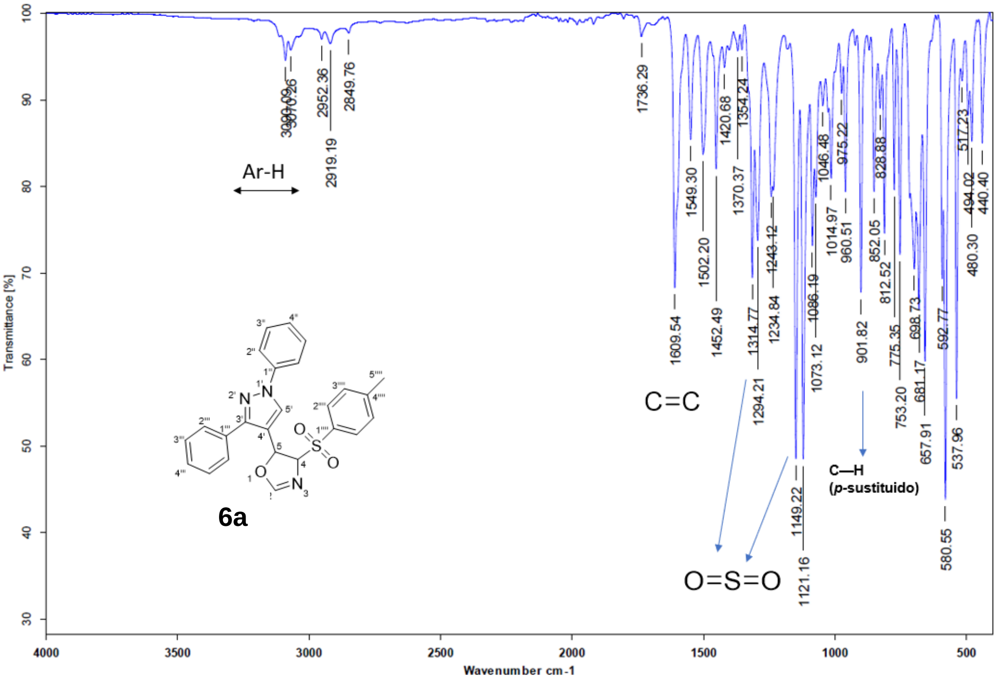

Figure S143. FT-IR of (4S*, 5S*)-5-(1,3-diphenyl-1H-pyrazol-4-yl)-4-tosyl-4,5-dihydrooxazole **6a**.

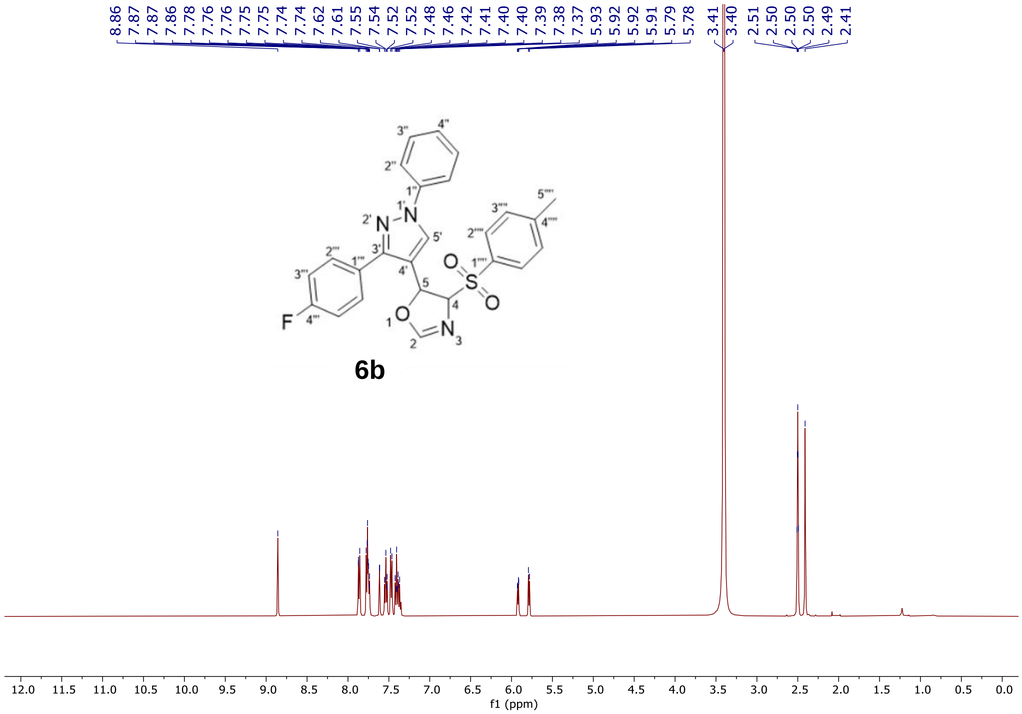

Figure S144. ^{1}H NMR (500 MHz, DMSO-d$_6$) of (4S*, 5S*)-5-(3-(4-fluorophenyl)-1-phenyl-1H-pyrazol-4-yl)-4-tosyl-4,5-dihydrooxazole **6b**.

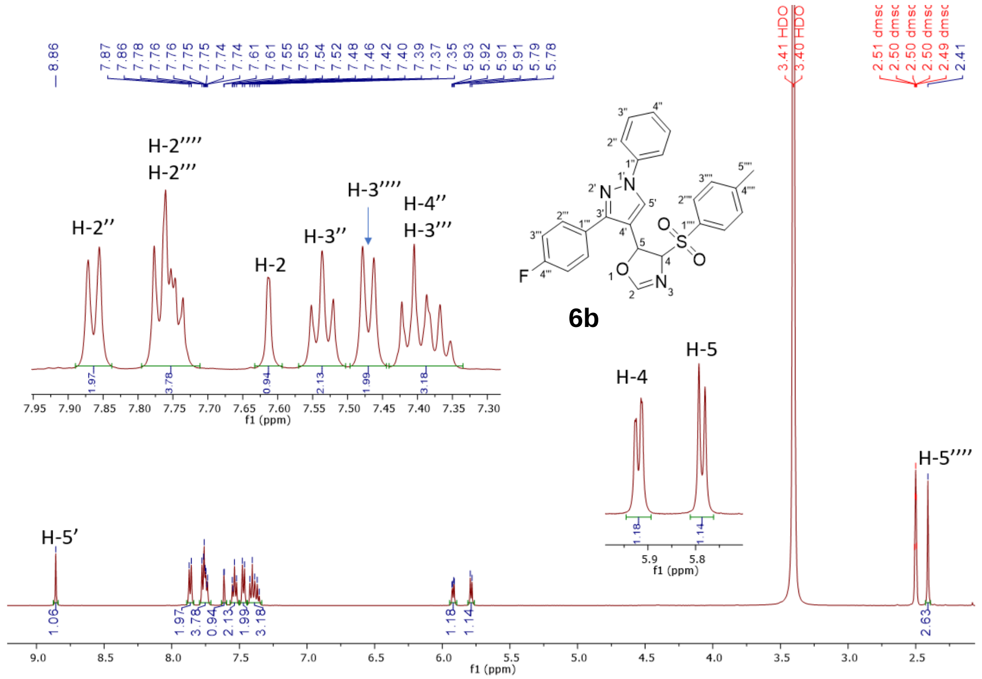

Figure S145. Expansion of ^{1}H NMR (500 MHz, DMSO-d$_6$) of (4*S**, 5*S**)-5-(3-(4-fluorophenyl)-1-phenyl-1*H*-pyrazol-4-yl)-4-tosyl-4,5-dihydrooxazole **6b**.

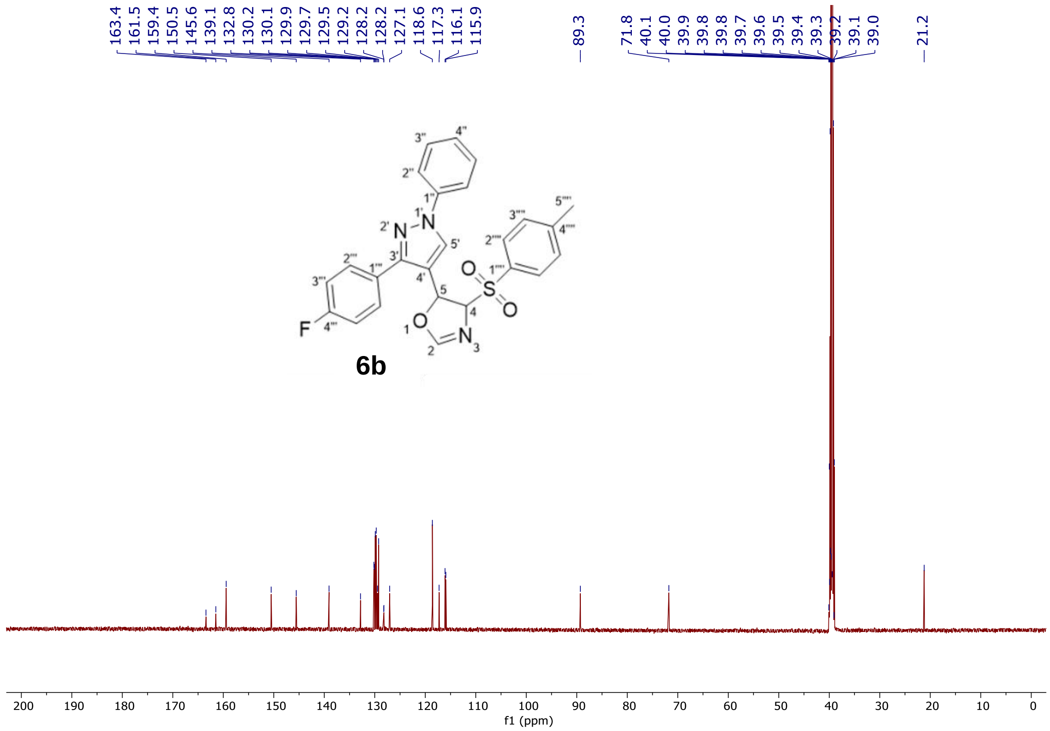

Figure S146. ^{13}C NMR (125 MHz, DMSO-d$_6$) of (4*S**, 5*S**)-5-(3-(4-fluorophenyl)-1-phenyl-1*H*-pyrazol-4-yl)-4-tosyl-4,5-dihydrooxazole **6b**.

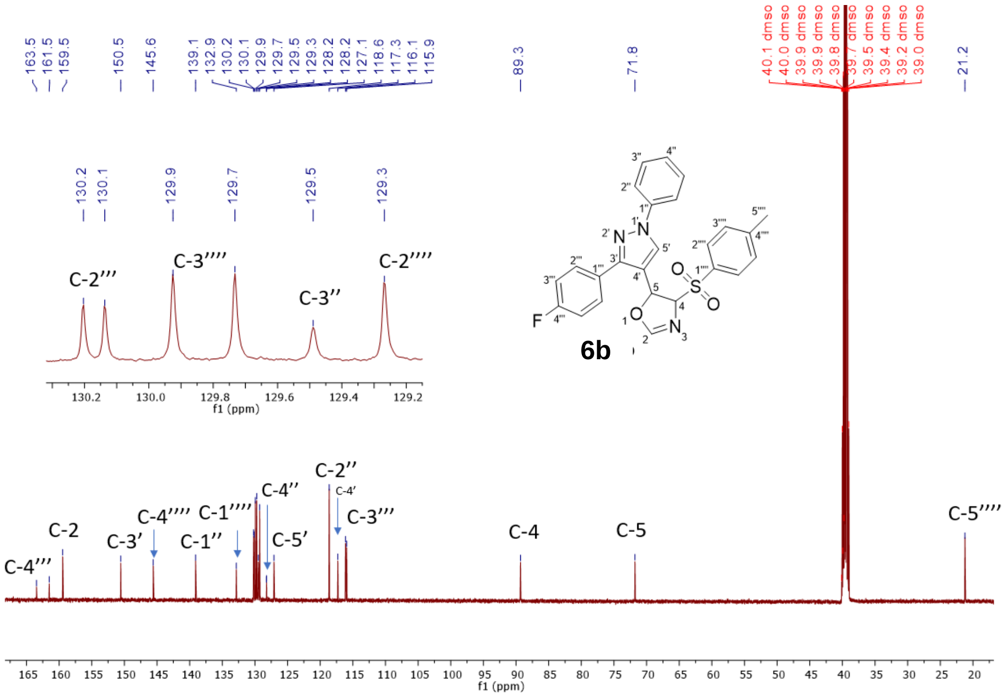

Figure S147. Expansion of ^{13}C NMR (125 MHz, DMSO-d$_6$) of (4*S**, 5*S**)-5-(3-(4-fluorophenyl)-1-phenyl-1*H*-pyrazol-4-yl)-4-tosyl-4,5-dihydrooxazole **6b**.

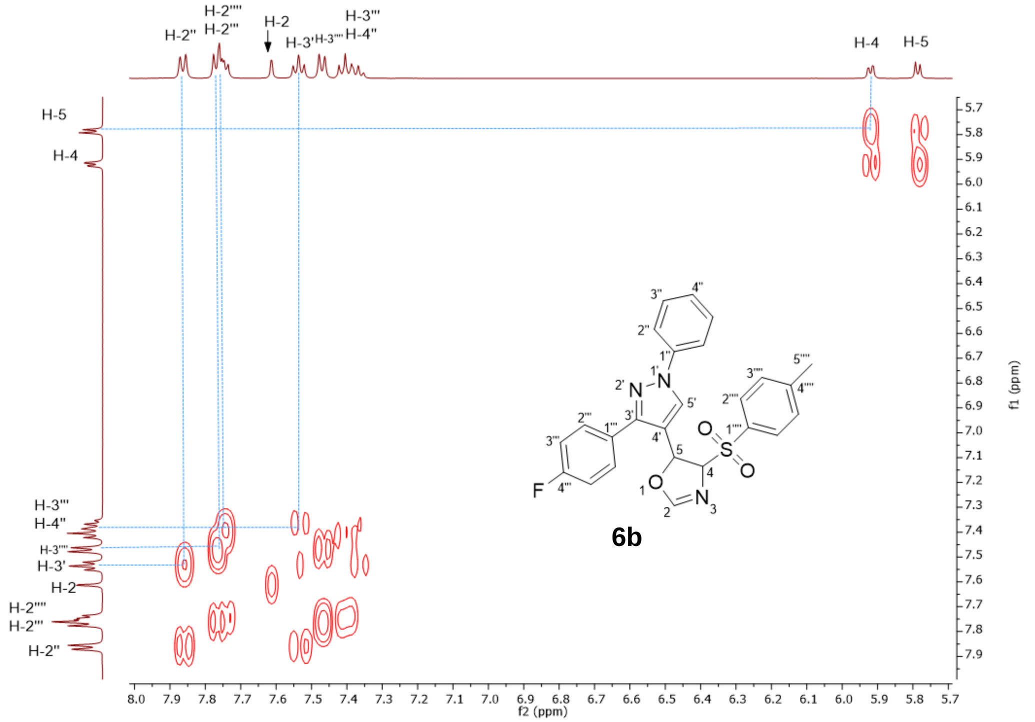

Figure S148. COSY experiment of (4*S**, 5*S**)-5-(3-(4-fluorophenyl)-1-phenyl-1*H*-pyrazol-4-yl)-4-tosyl-4,5-dihydrooxazole **6b**.

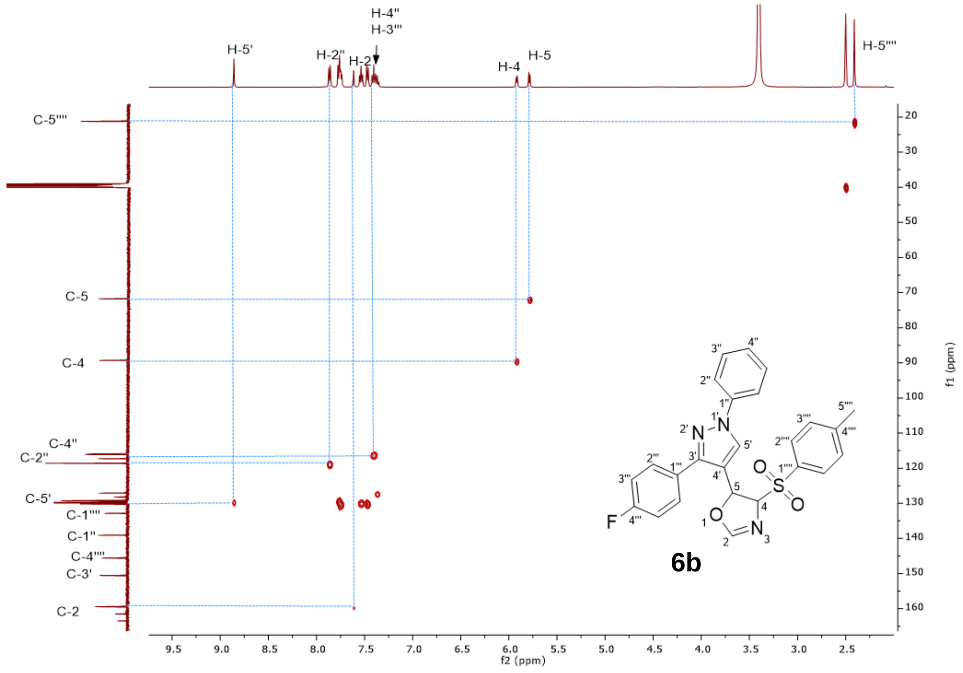

Figure S149. HSQC experiment of (4*S**, 5*S**)-5-(3-(4-fluorophenyl)-1-phenyl-1*H*-pyrazol-4-yl)-4-tosyl-4,5-dihydrooxazole **6b**.

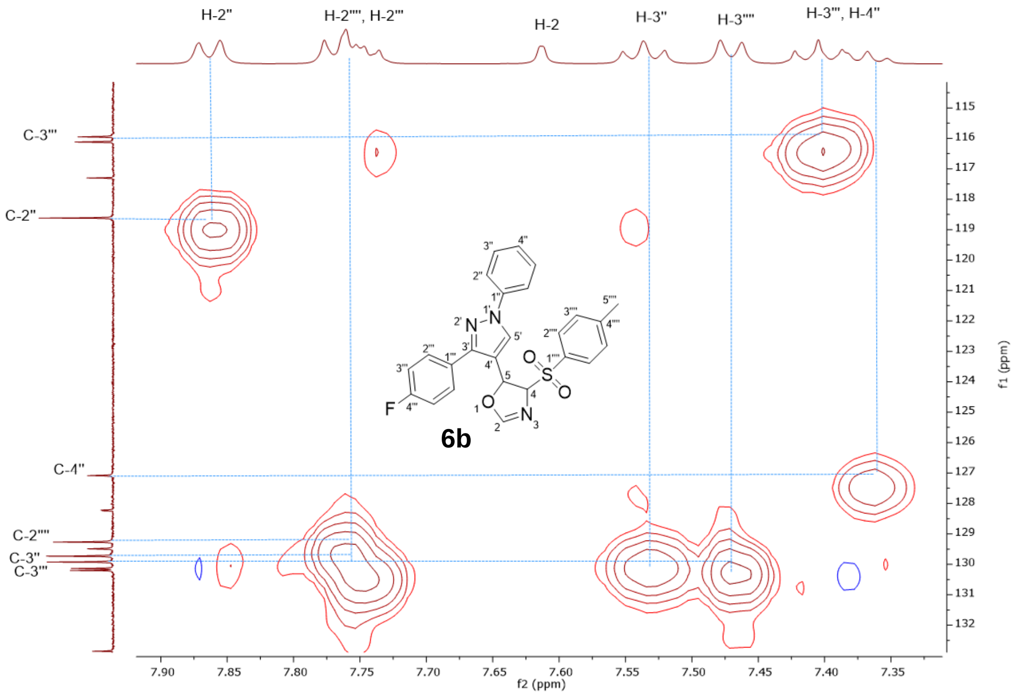

Figure S150. Expansion of HSQC experiment of (4*S**, 5*S**)-5-(3-(4-fluorophenyl)-1-phenyl-1*H*-pyrazol-4-yl)-4-tosyl-4,5-dihydrooxazole **6b**.

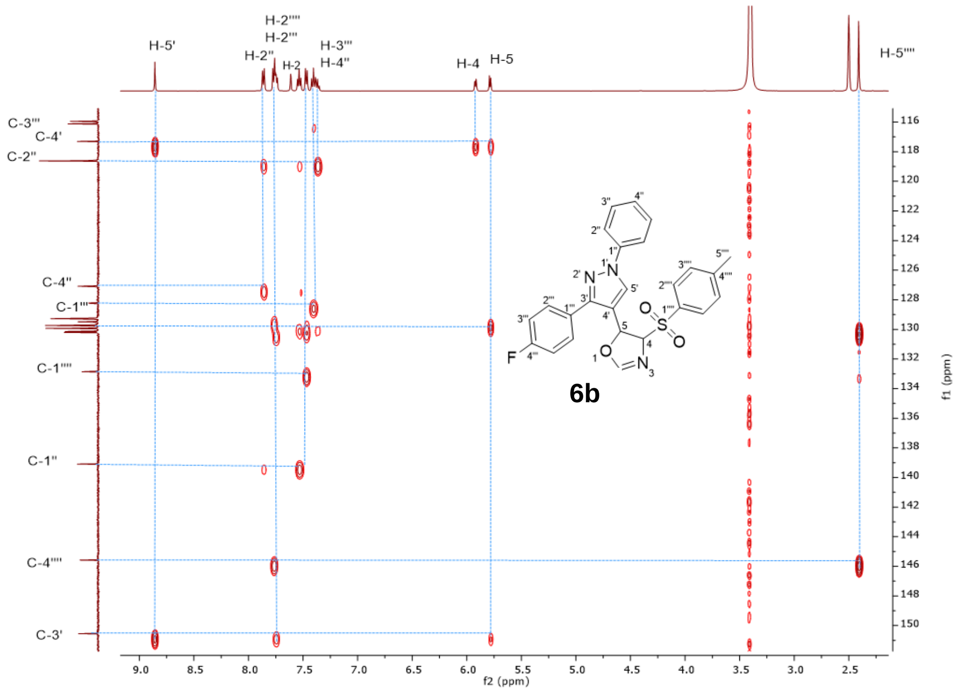

Figure S151. HMBC experiment of (4*S**, 5*S**)-5-(3-(4-fluorophenyl)-1-phenyl-1*H*-pyrazol-4-yl)-4-tosyl-4,5-dihydrooxazole **6b**.

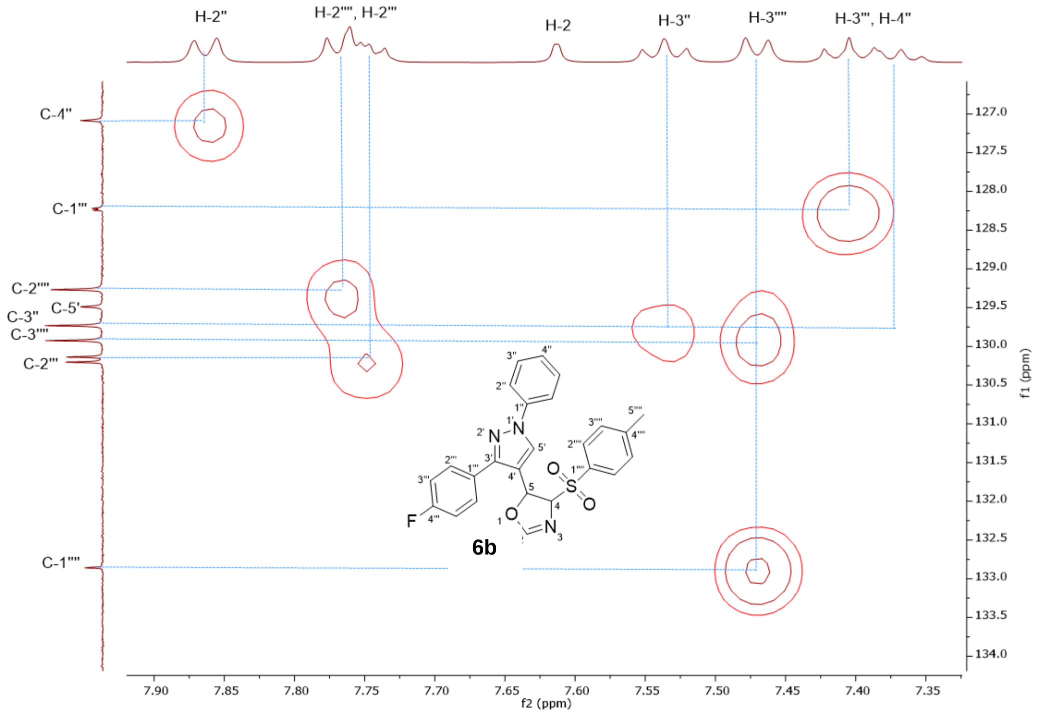

Figure S152. Expansion of HMBC experiment of (4*S**, 5*S**)-5-(3-(4-fluorophenyl)-1-phenyl-1*H*-pyrazol-4-yl)-4-tosyl-4,5-dihydrooxazole **6b**.

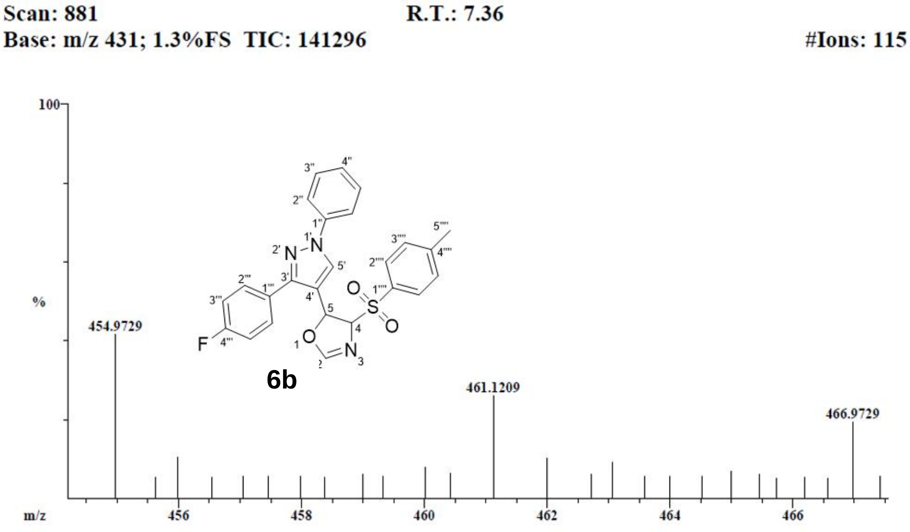

Selected Isotopes : $H_{0\text{-}20}C_{0\text{-}25}N_{0\text{-}3}O_{0\text{-}3}F_{0\text{-}1}S_{0\text{-}1}$ | Error Limit : 5 ppm | "Molecular" compositions | Unsaturation Limits : 0 to 50

Measured Mass	% Base	Formula	Calculated Mass	Error	Unsaturation
461.1209	26.1%	$C_{25}H_{20}N_3O_3F\,S$	461.1209	-0.1	17.0

Figure S153. HRMS of (4*S**, 5*S**)-5-(3-(4-fluorophenyl)-1-phenyl-1*H*-pyrazol-4-yl)-4-tosyl-4,5-dihydrooxazole **6b**.

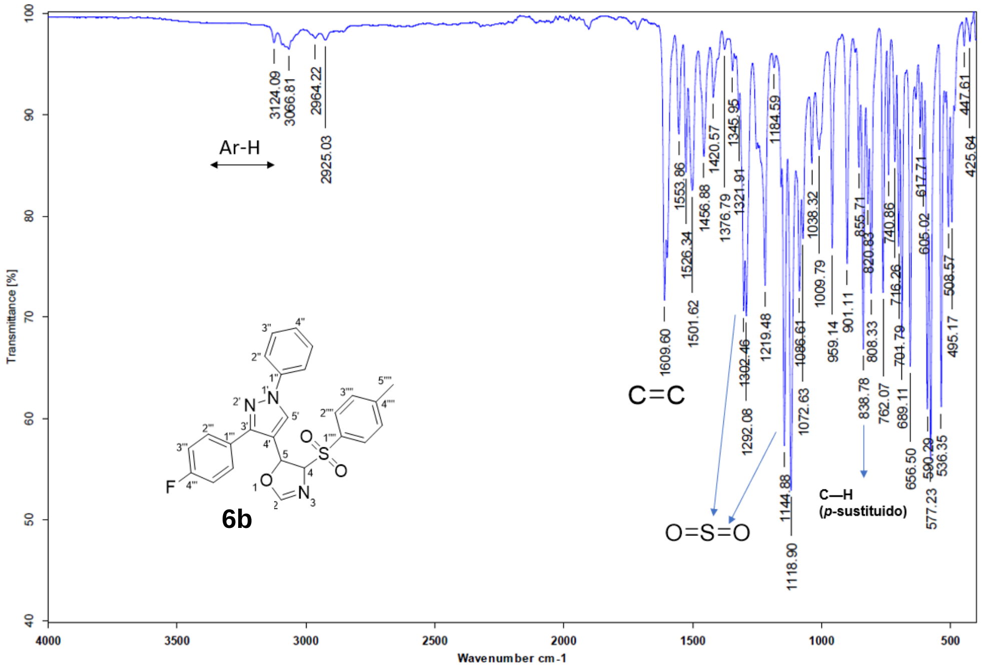

Figure S154. FT-IR of (4*S**, 5*S**)-5-(3-(4-fluorophenyl)-1-phenyl-1*H*-pyrazol-4-yl)-4-tosyl-4,5-dihydrooxazole **6b**.

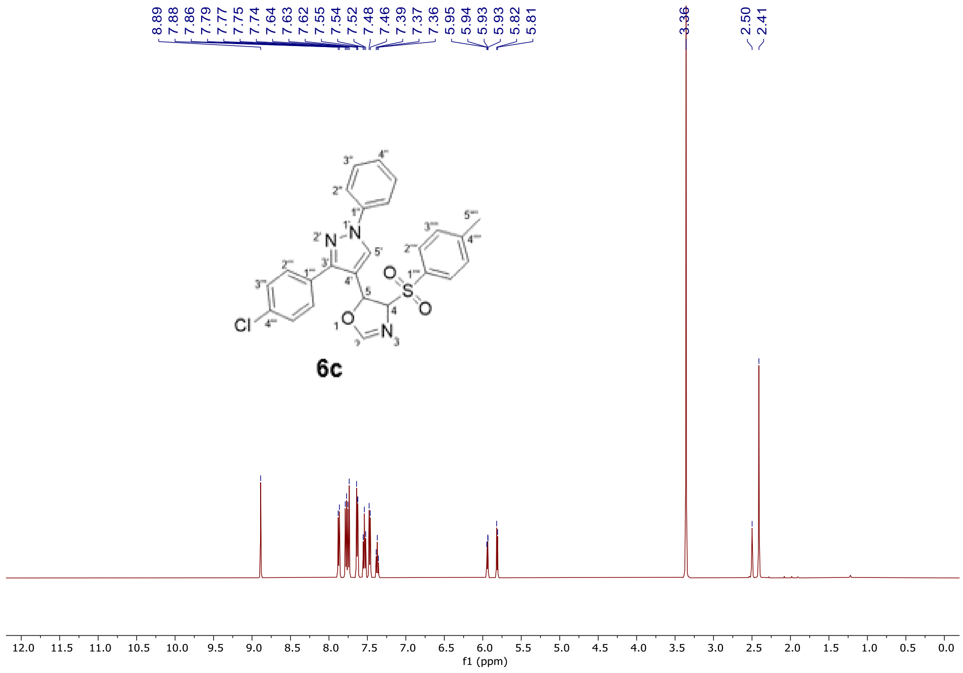

Figure S155. ^{1}H NMR (500 MHz, DMSO-d$_6$) of (4*S**, 5*S**)-5-(3-(4-chlorophenyl)-1-phenyl-1*H*-pyrazol-4-yl)-4-tosyl-4,5-dihydrooxazole **6c**.

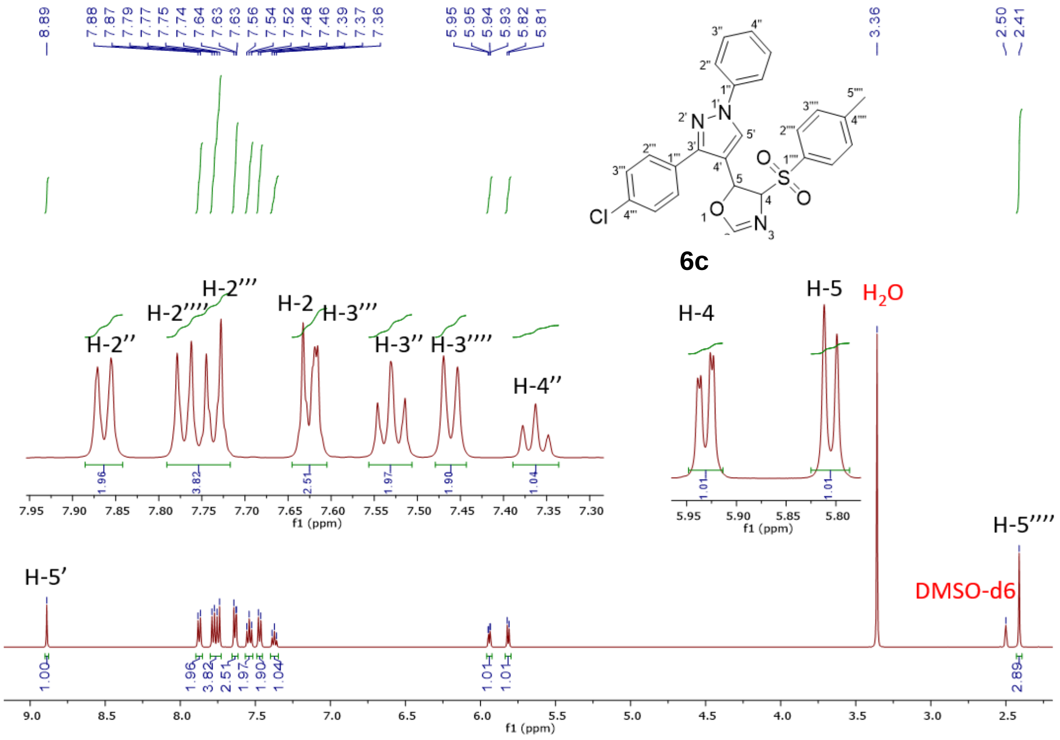

Figure S156. Expansion of ^{1}H NMR (500 MHz, DMSO-d$_6$) of (4*S**, 5*S**)-5-(3-(4-chlorophenyl)-1-phenyl-1*H*-pyrazol-4-yl)-4-tosyl-4,5-dihydrooxazole **6c**.

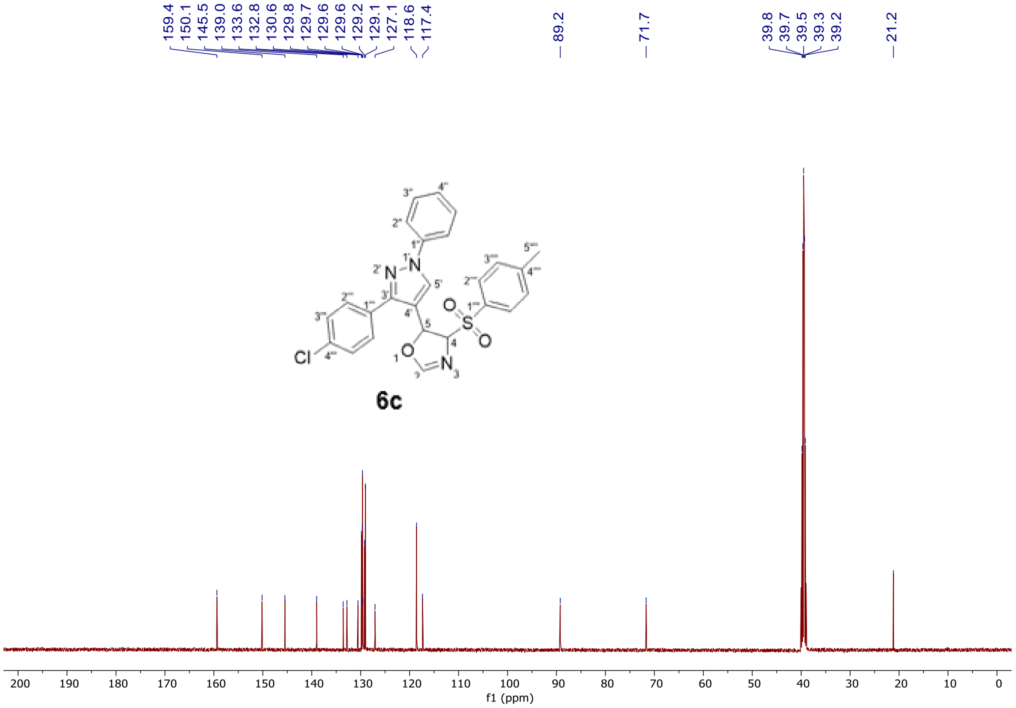

Figure S157. ^{13}C NMR (125 MHz, DMSO-d$_6$) of (4*S**, 5*S**)-5-(3-(4-chlorophenyl)-1-phenyl-1*H*-pyrazol-4-yl)-4-tosyl-4,5-dihydroxazole **6c**.

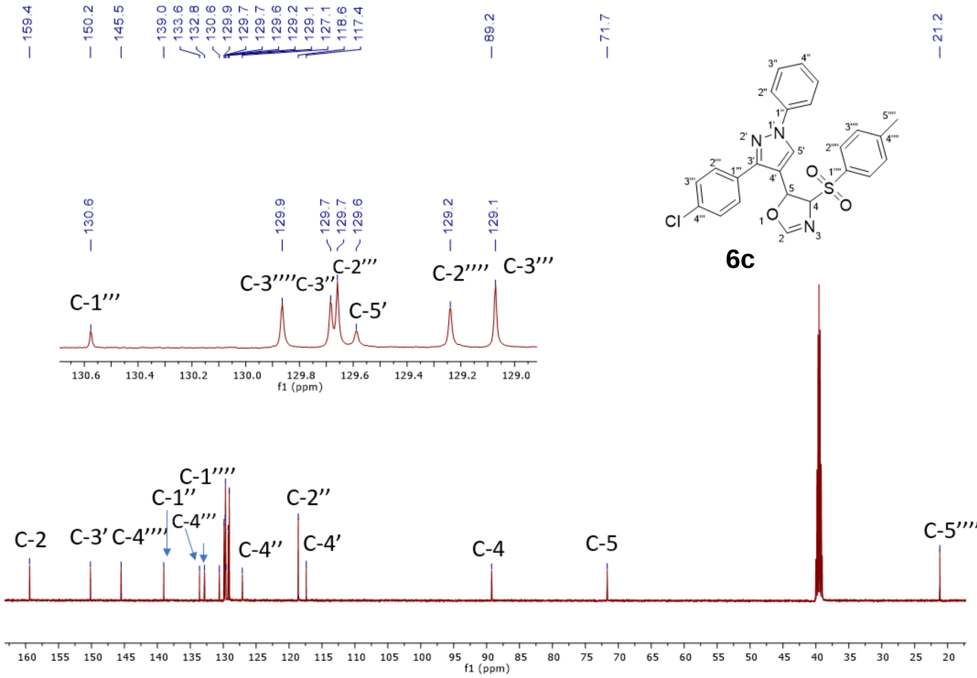

Figure S158. Expansion of ^{13}C NMR (125 MHz, DMSO-d$_6$) of (4*S**, 5*S**)-5-(3-(4-chlorophenyl)-1-phenyl-1*H*-pyrazol-4-yl)-4-tosyl-4,5-dihydroxazole **6c**.

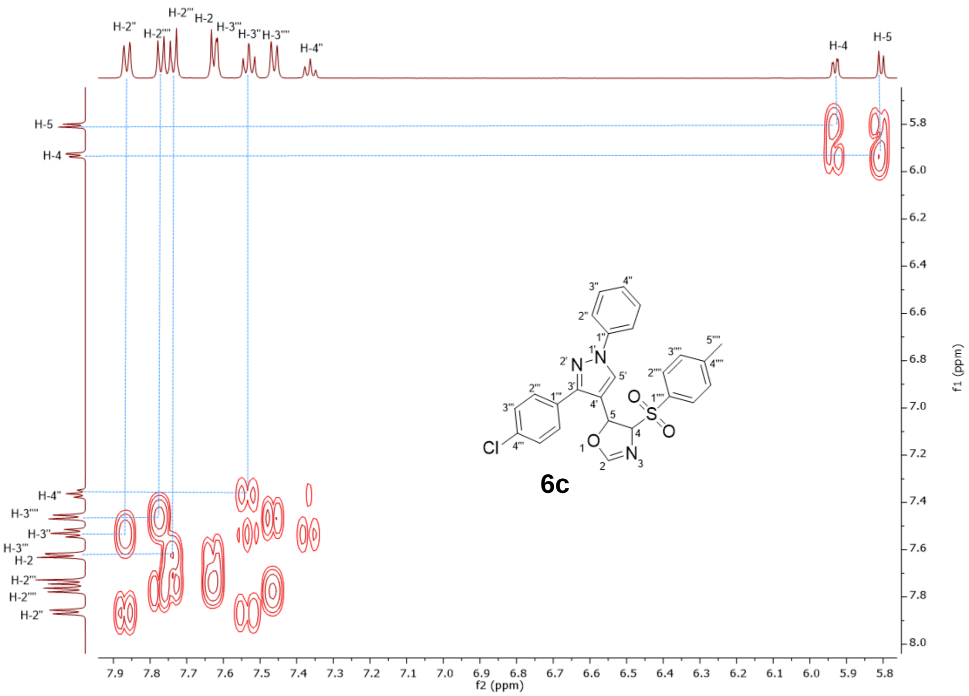

Figure S159. COSY experiment of (4*S**, 5*S**)-5-(3-(4-chlorophenyl)-1-phenyl-1*H*-pyrazol-4-yl)-4-tosyl-4,5-dihydrooxazole **6c**.

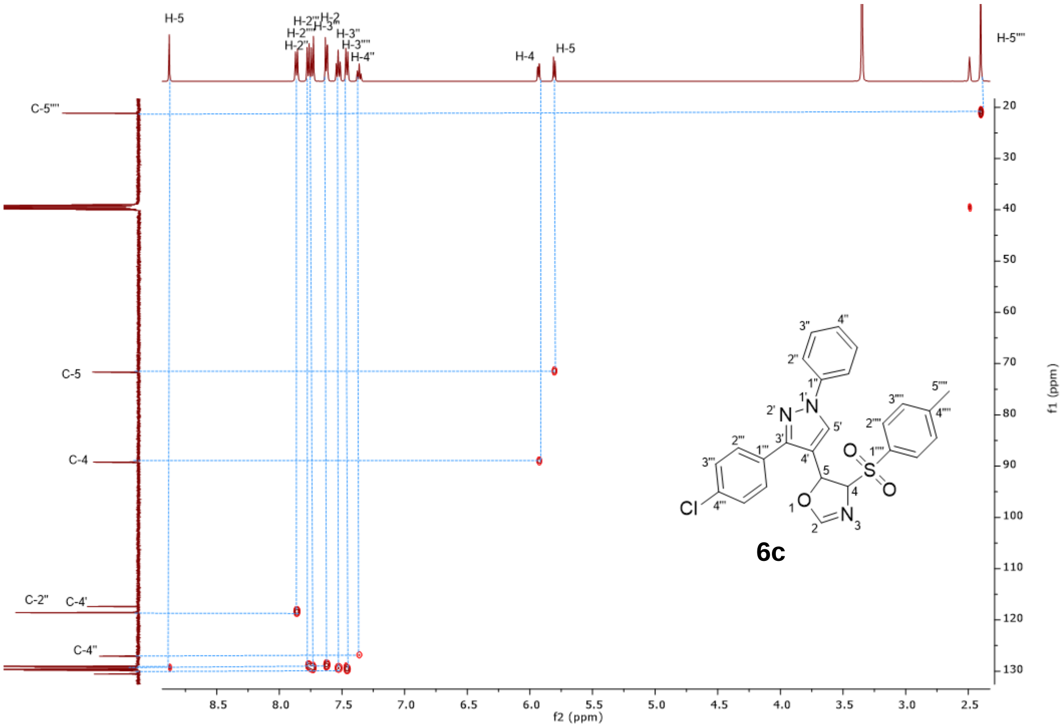

Figure S160. HSQC experiment of (4*S**, 5*S**)-5-(3-(4-chlorophenyl)-1-phenyl-1*H*-pyrazol-4-yl)-4-tosyl-4,5-dihydrooxazole **6c**.

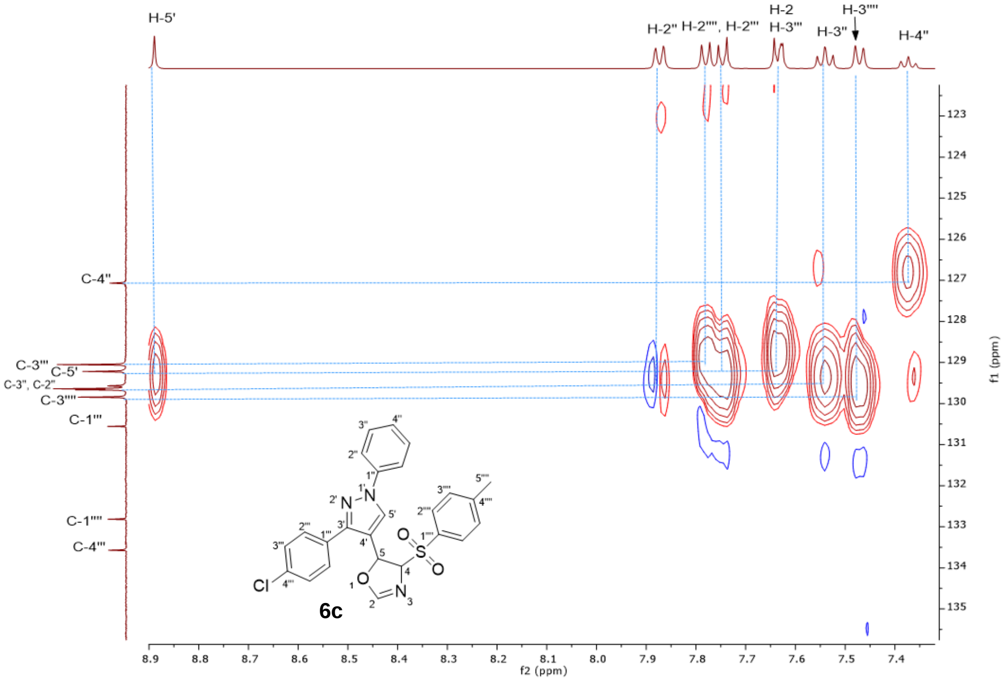

Figure S161. Expansion of HSQC experiment of (4*S**, 5*S**)-5-(3-(4-chlorophenyl)-1-phenyl-1*H*-pyrazol-4-yl)-4-tosyl-4,5-dihydrooxazole **6c**.

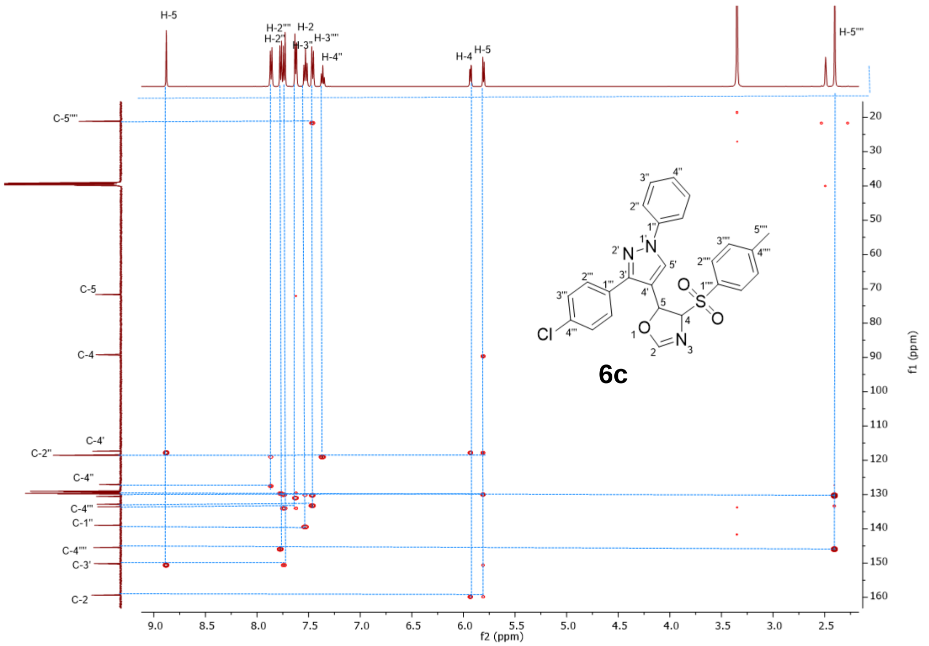

Figure S162. HMBC experiment of (4*S**, 5*S**)-5-(3-(4-chlorophenyl)-1-phenyl-1*H*-pyrazol-4-yl)-4-tosyl-4,5-dihydrooxazole **6c**.

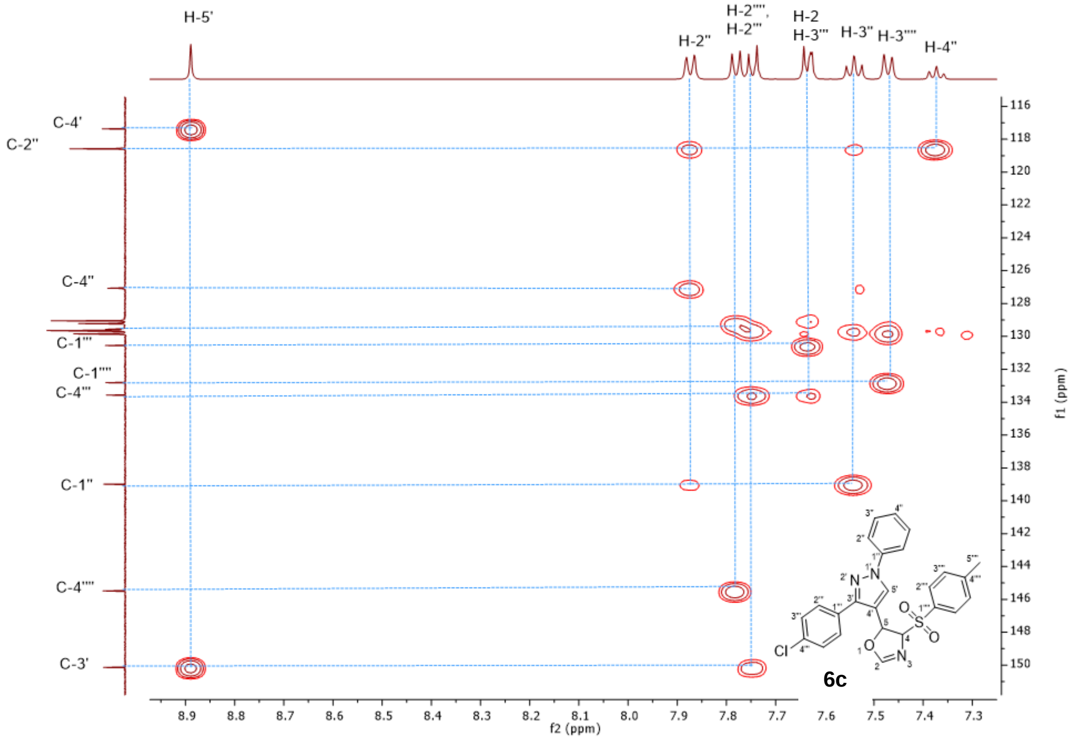

Figure S163. Expansion of HMBC experiment of (4*S**, 5*S**)-5-(3-(4-chlorophenyl)-1-phenyl-1*H*-pyrazol-4-yl)-4-tosyl-4,5-dihydrooxazole **6c**.

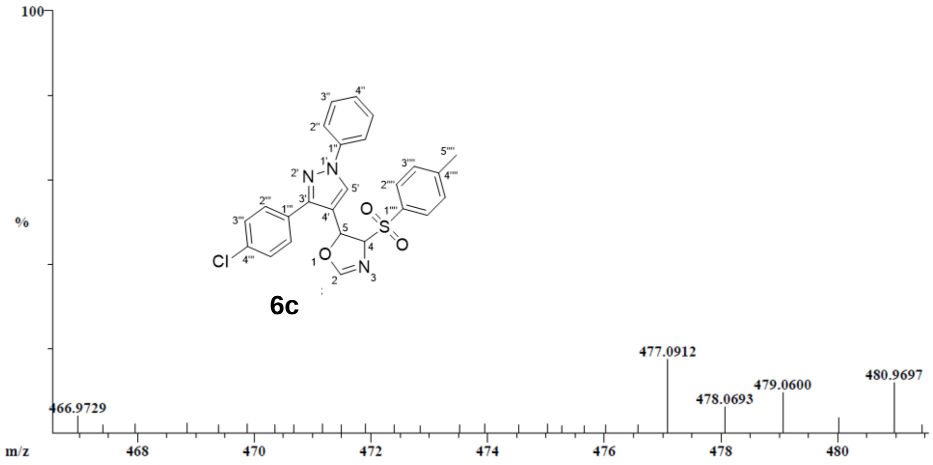

Selected Isotopes : $H_{0-20}C_{0-25}N_{0-3}O_{0-3}Cl_{0-1}S_{0-1}$ Error Limit : 5 ppm Unsaturation Limits : 0 to 50

Measured Mass	% Base	Formula	Calculated Mass	Error	Unsaturation
477.0912	17.5%	$C_{25}H_{20}N_3O_3ClS$	477.0914	-0.4	17.0

Figure S164. HRMS of (4*S**, 5*S**)-5-(3-(4-chlorophenyl)-1-phenyl-1*H*-pyrazol-4-yl)-4-tosyl-4,5-dihydrooxazole **6c**.

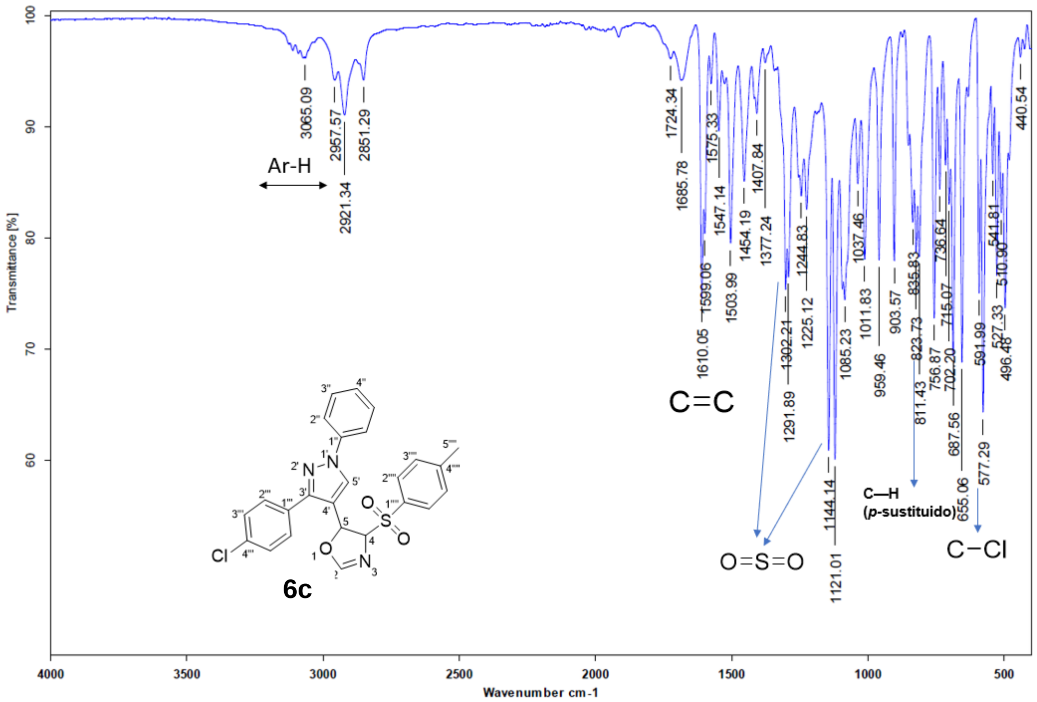

Figure S165. FT-IR of (4S*, 5S*)-5-(3-(4-chlorophenyl)-1-phenyl-1H-pyrazol-4-yl)-4-tosyl-4,5-dihydrooxazole **6c**.

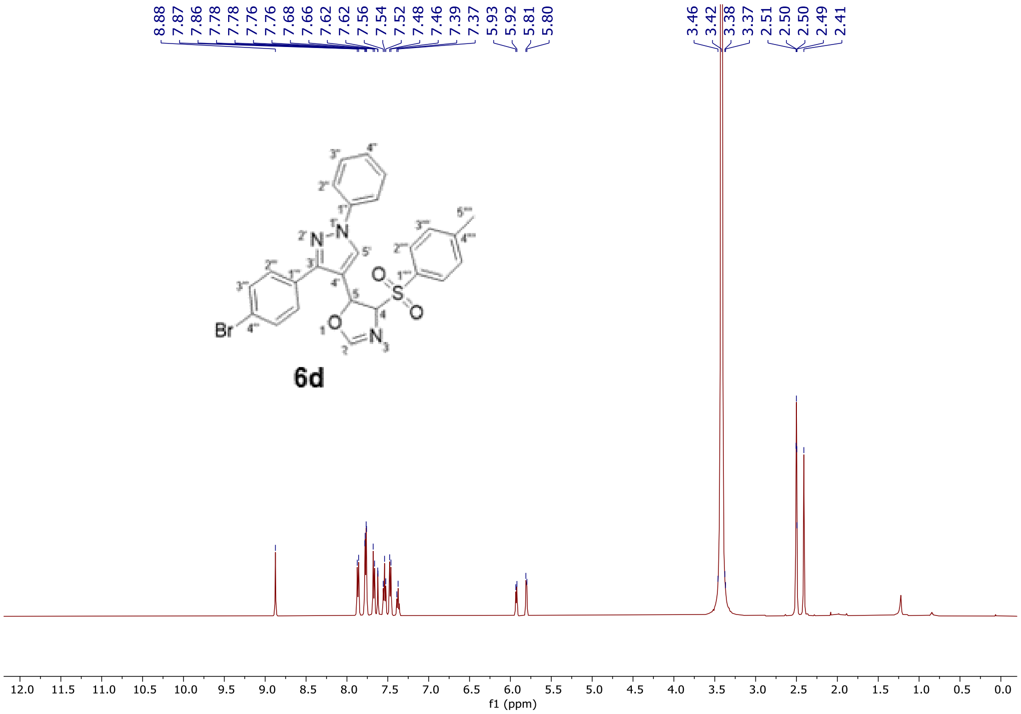

Figure S166. ^{1}H NMR (500 MHz, DMSO-d$_6$) of (4S*, 5S*)-5-(3-(4-bromophenyl)-1-phenyl-1H-pyrazol-4-yl)-4-tosyl-4,5-dihydrooxazole **6d**.

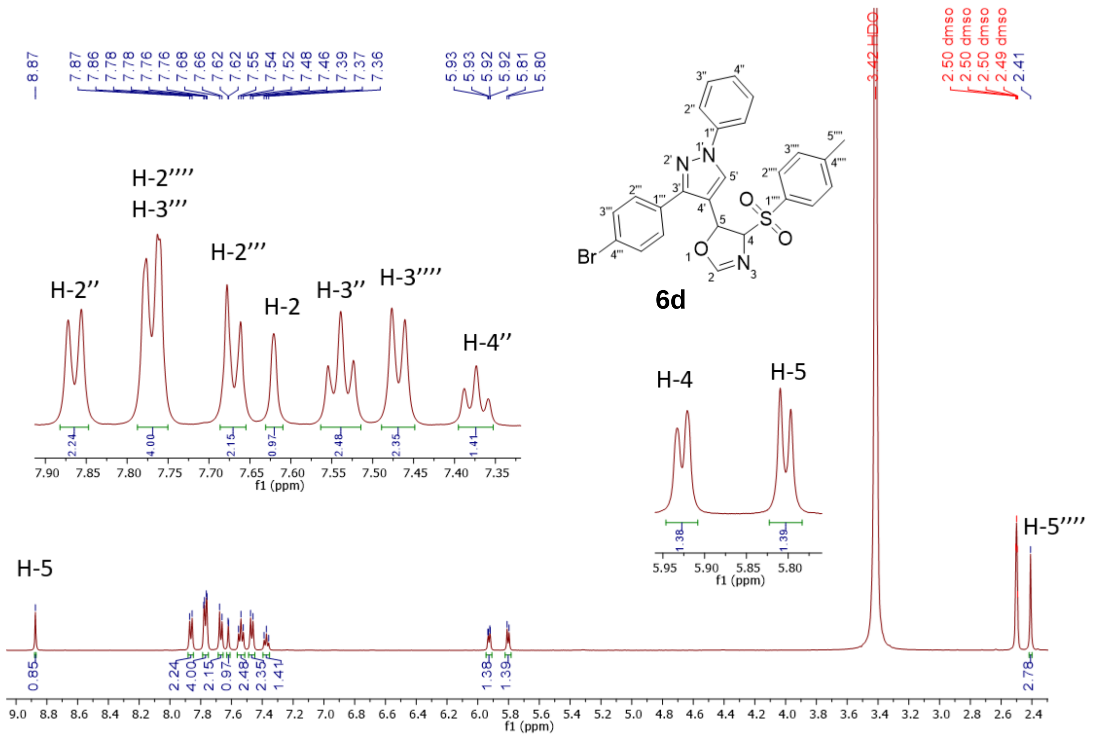

Figure S167. Expansion of ^{1}H NMR (500 MHz, DMSO-d$_6$) of (4*S**, 5*S**)-5-(3-(4-bromophenyl)-1-phenyl-1*H*-pyrazol-4-yl)-4-tosyl-4,5-dihydrooxazole **6d**.

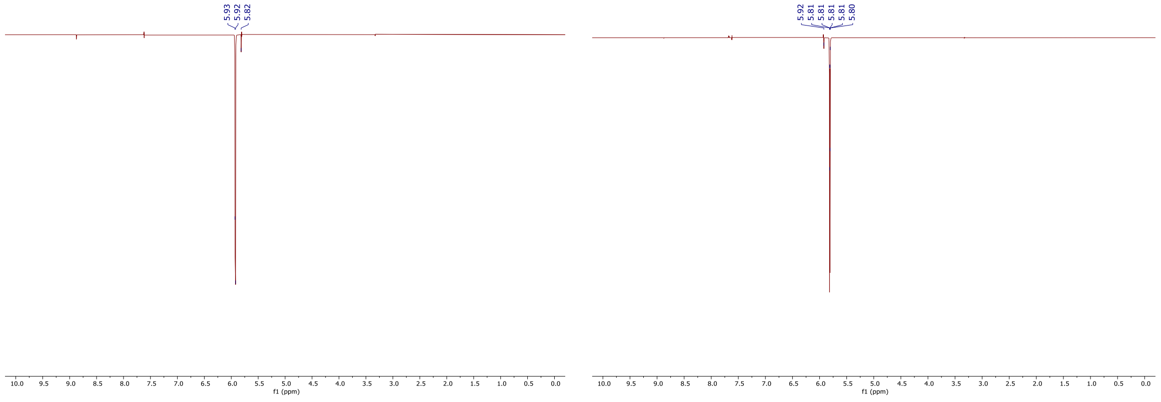

Figure S168. NOESY experiments of (4*S**, 5*S**)-5-(3-(4-bromophenyl)-1-phenyl-1*H*-pyrazol-4-yl)-4-tosyl-4,5-dihydrooxazole **6d** (H-4 and H-5 were irradiated).

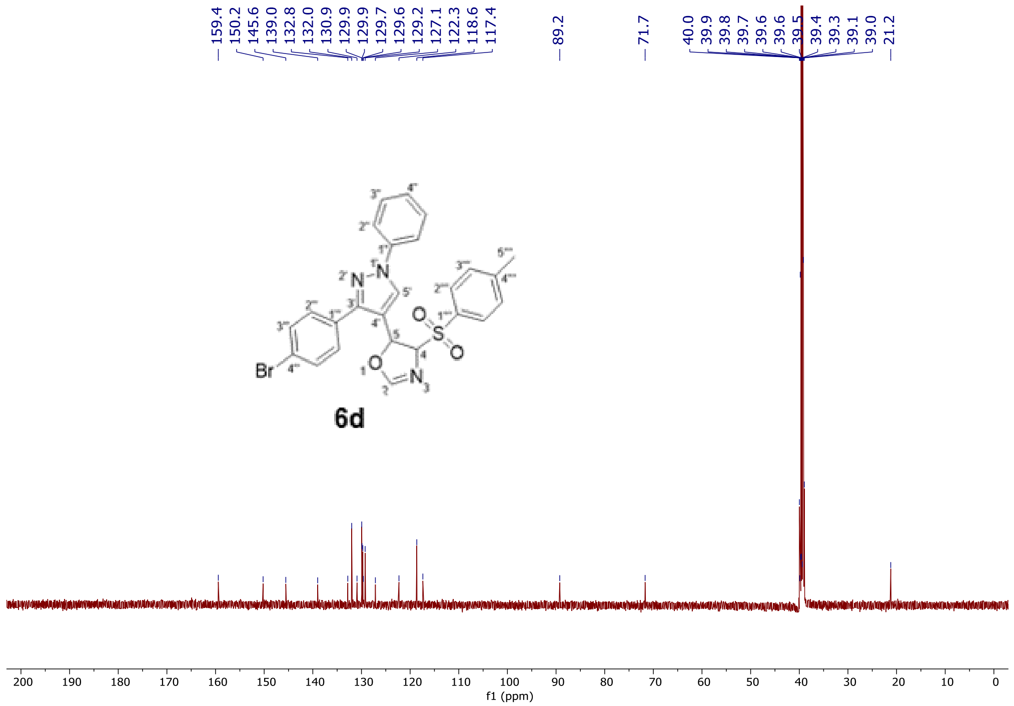

Figure S169. ^{13}C NMR (125 MHz, DMSO-d$_6$) of (4*S**, 5*S**)-5-(3-(4-bromophenyl)-1-phenyl-1*H*-pyrazol-4-yl)-4-tosyl-4,5-dihydrooxazole **6d**.

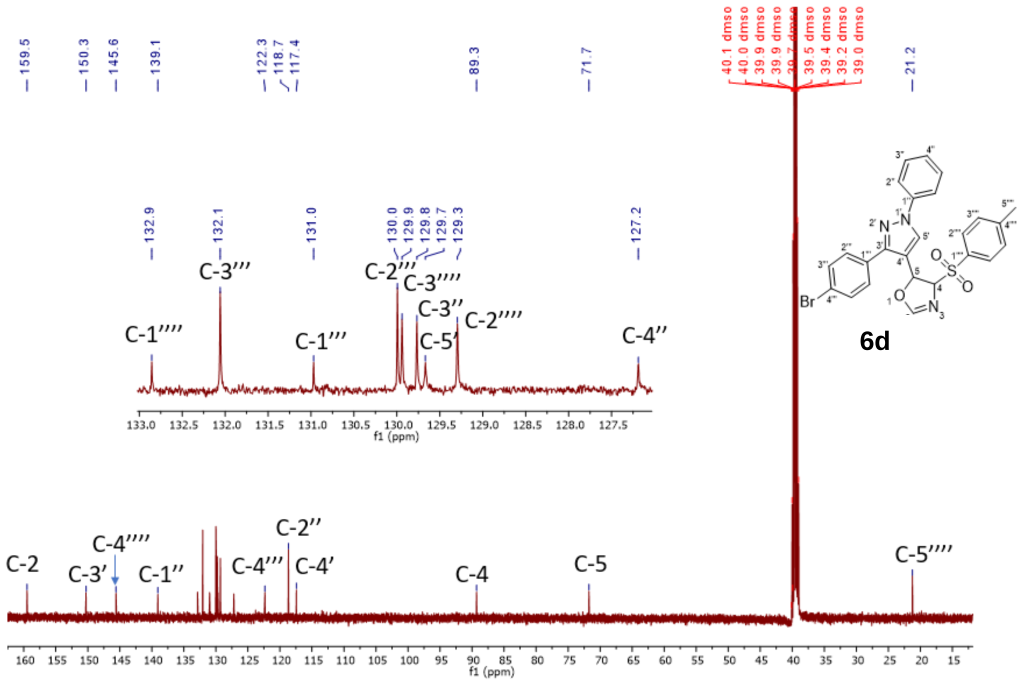

Figure S170. Expansion of ^{13}C NMR (125 MHz, DMSO-d$_6$) of (4*S**, 5*S**)-5-(3-(4-bromophenyl)-1-phenyl-1*H*-pyrazol-4-yl)-4-tosyl-4,5-dihydrooxazole **6d**.

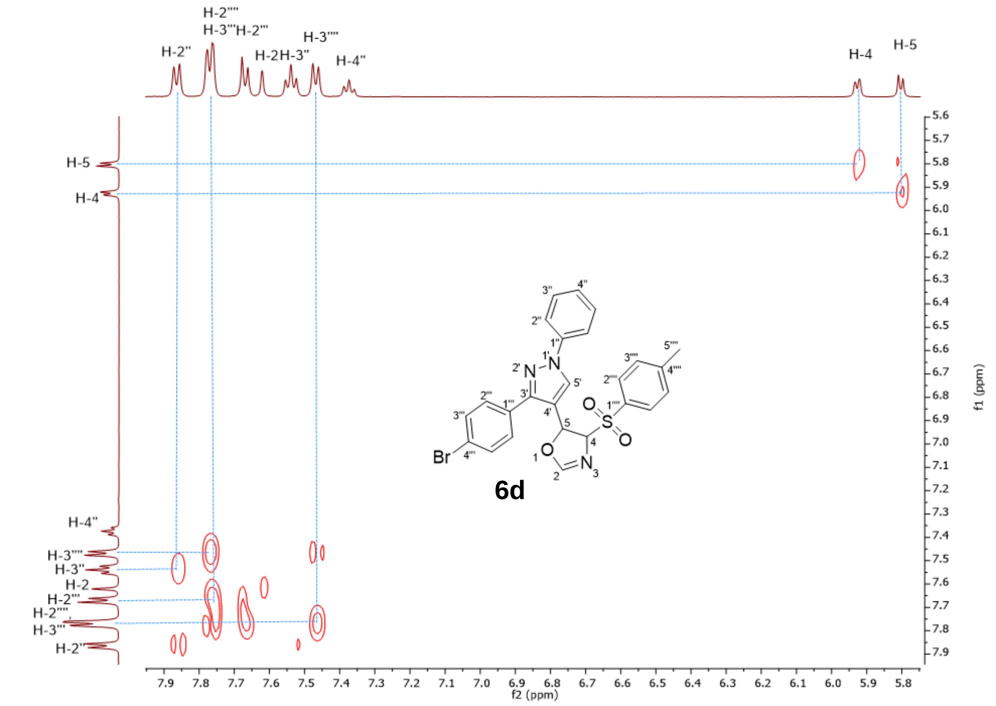

Figure S171. COSY experiment of (4*S**, 5*S**)-5-(3-(4-bromophenyl)-1-phenyl-1*H*-pyrazol-4-yl)-4-tosyl-4,5-dihydrooxazole **6d**.

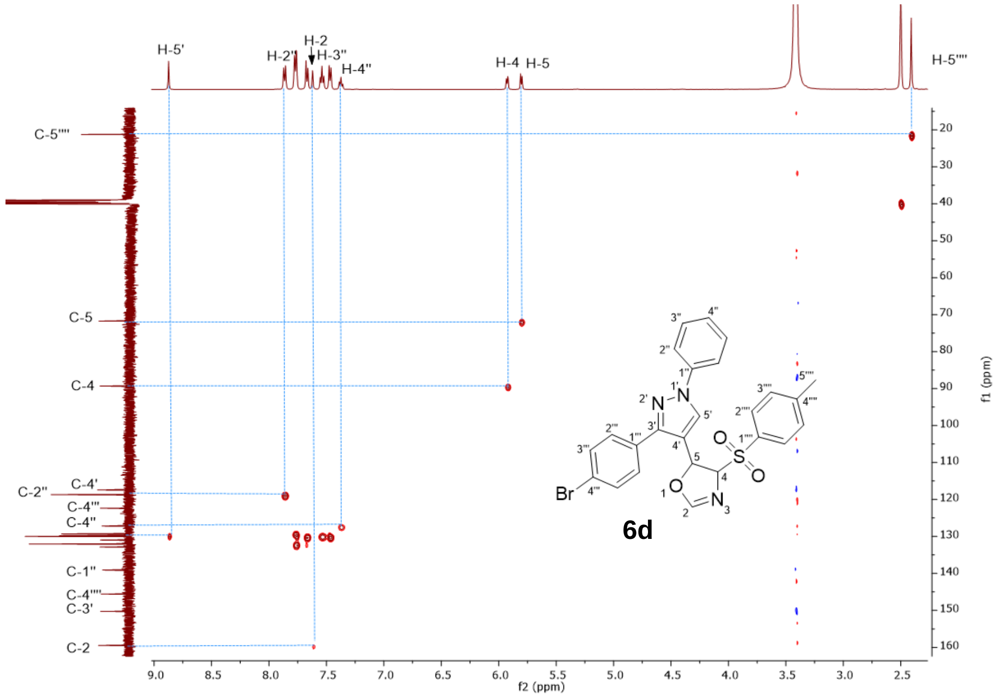

Figure S172. HSQC experiment of (4*S**, 5*S**)-5-(3-(4-bromophenyl)-1-phenyl-1*H*-pyrazol-4-yl)-4-tosyl-4,5-dihydrooxazole **6d**.

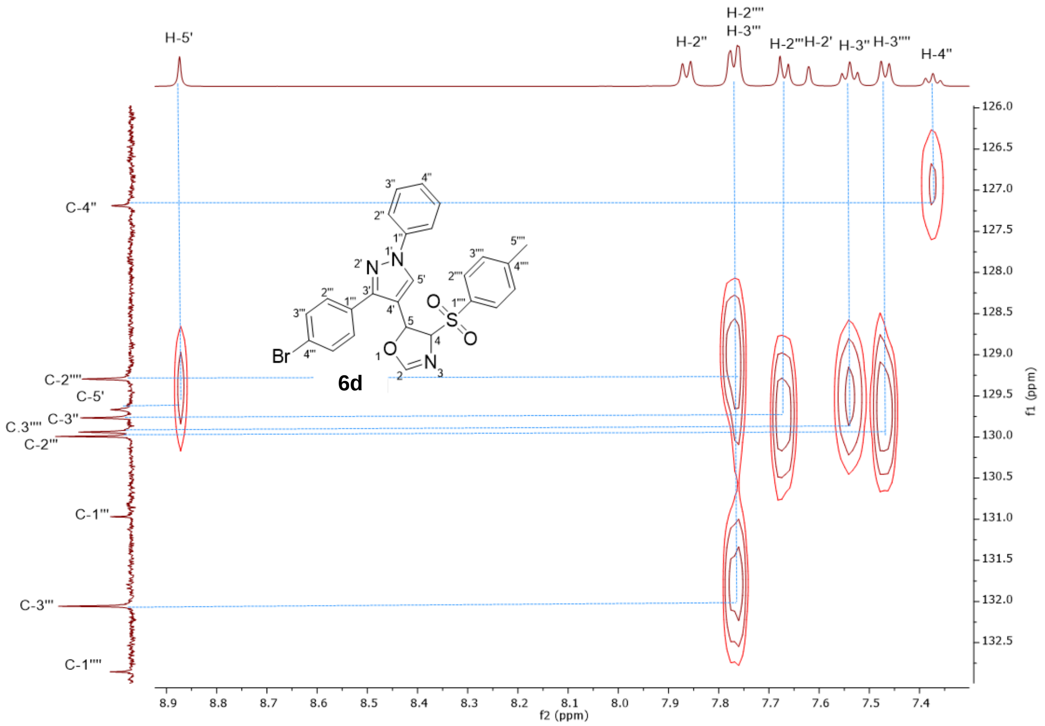

Figure S173. Expansion of HSQC experiment of (4*S**, 5*S**)-5-(3-(4-bromophenyl)-1-phenyl-1*H*-pyrazol-4-yl)-4-tosyl-4,5-dihydrooxazole **6d**.

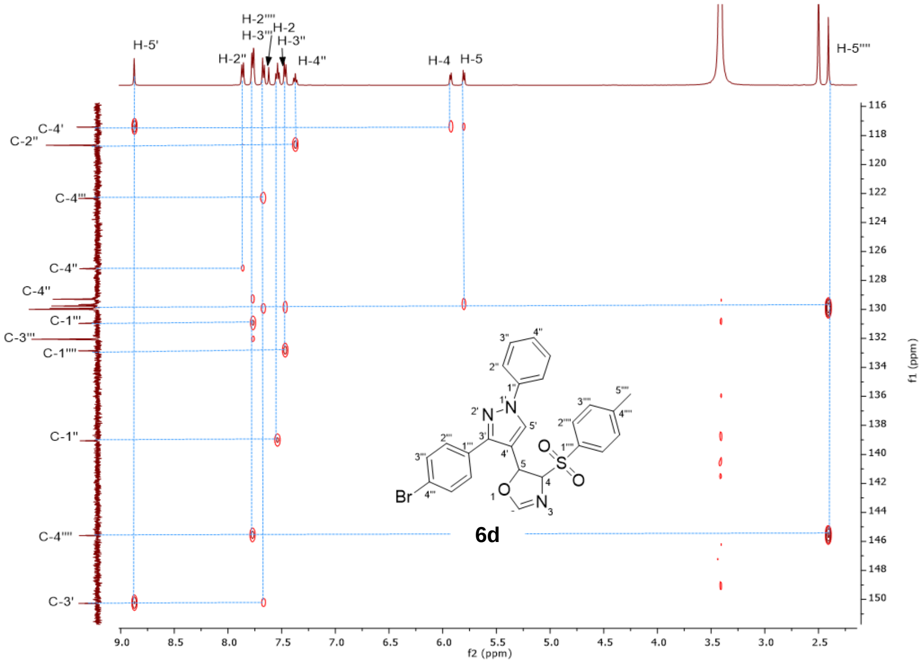

Figure S174. HMBC experiment of (4*S**, 5*S**)-5-(3-(4-bromophenyl)-1-phenyl-1*H*-pyrazol-4-yl)-4-tosyl-4,5-dihydrooxazole **6d**.

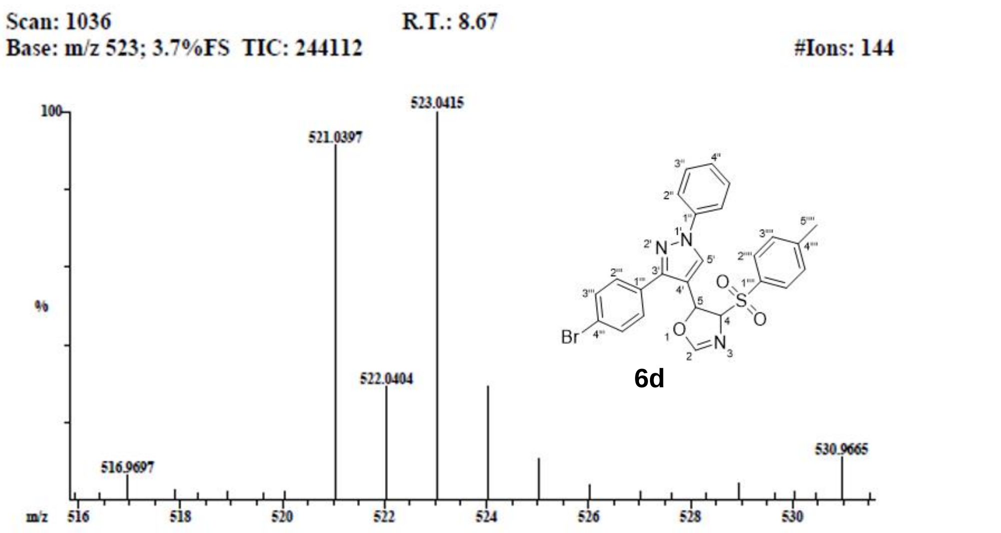

Selected Isotopes : $H_{0\text{-}20}C_{0\text{-}25}N_{0\text{-}3}O_{0\text{-}3}S_{0\text{-}1}Br_{0\text{-}1}$ Error Limit : 5 ppm Unsaturation Limits : 0 to 50

Measured Mass	% Base	Formula	Calculated Mass	Error	Unsaturation
521.0397	91.4%	$C_{25}H_{20}N_3O_3S$ Br	521.0409	-2.2	17.0

Figure S175. HRMS of (4*S**, 5*S**)-5-(3-(4-bromophenyl)-1-phenyl-1*H*-pyrazol-4-yl)-4-tosyl-4,5-dihydrooxazole **6d**.

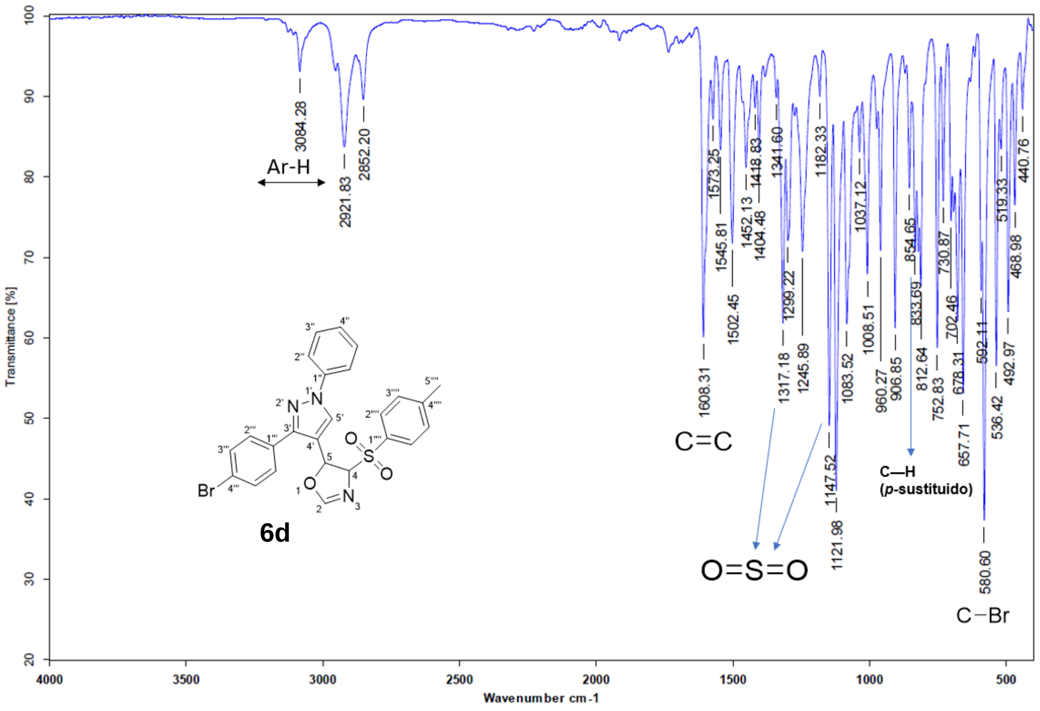

Figure S176. FT-IR of (4*S**, 5*S**)-5-(3-(4-bromophenyl)-1-phenyl-1*H*-pyrazol-4-yl)-4-tosyl-4,5-dihydrooxazole **6d**.

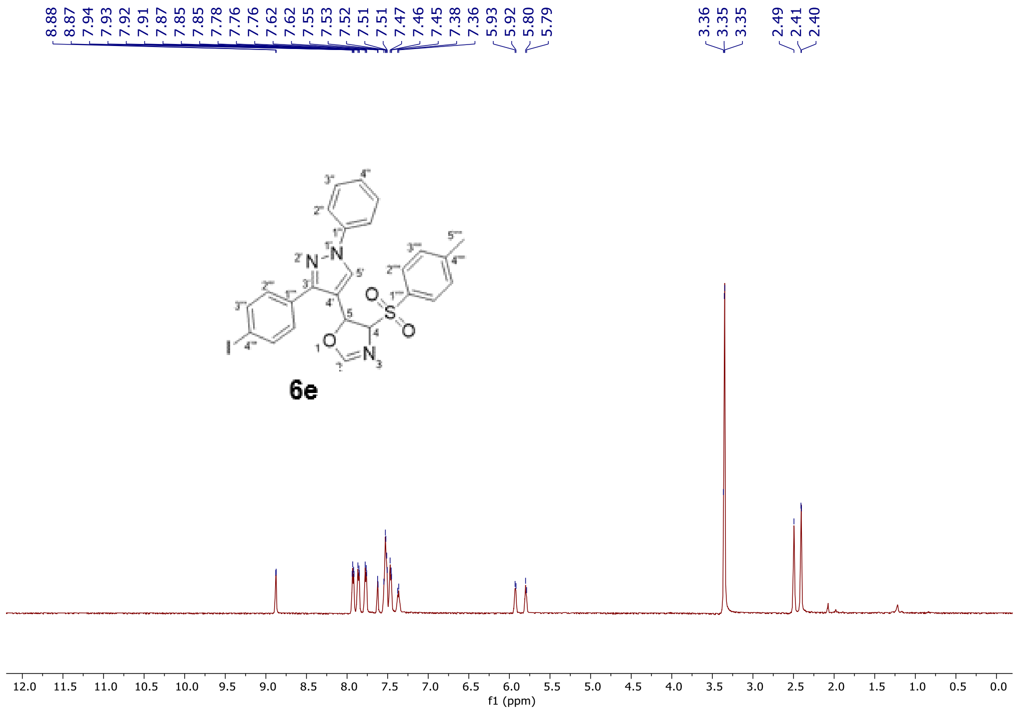

Figure S177. ^{1}H NMR (500 MHz, DMSO-d$_6$) of (4*S**, 5*S**)-5-(3-(4-iodophenyl)-1-phenyl-1*H*-pyrazol-4-yl)-4-tosyl-4,5-dihydrooxazole **6e**.

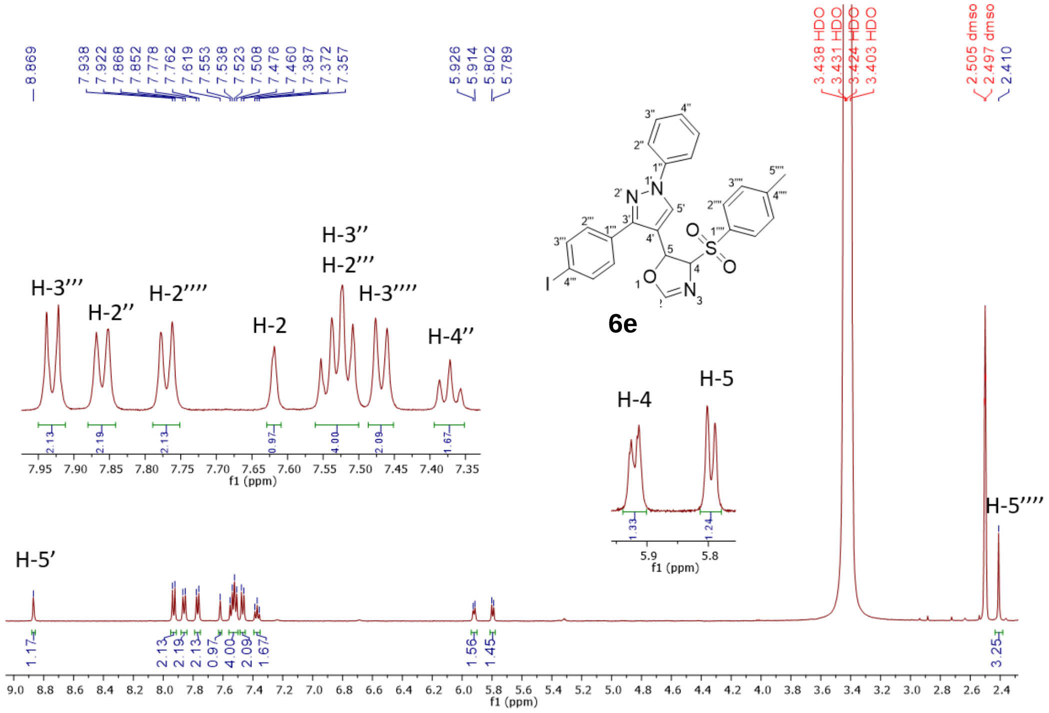

Figure S178. Expansion of ^{1}H NMR (500 MHz, DMSO-d$_6$) of (4*S**, 5*S**)-5-(3-(4-iodophenyl)-1-phenyl-1*H*-pyrazol-4-yl)-4-tosyl-4,5-dihydrooxazole **6e**.

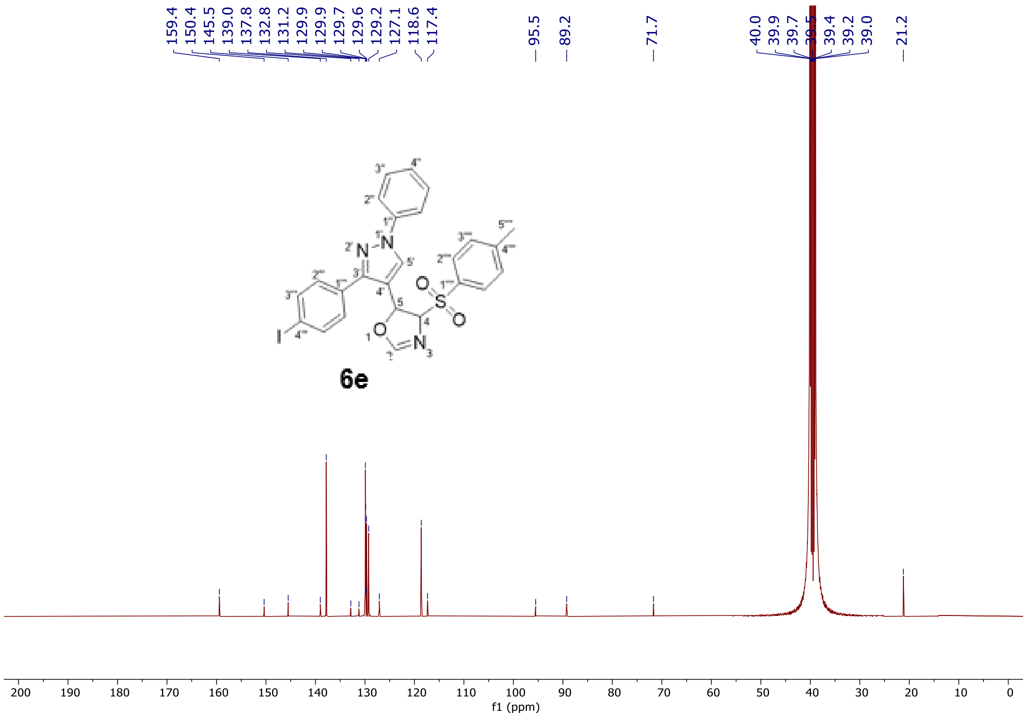

Figure S179. ^{13}C NMR (125 MHz, DMSO-d$_6$) of (4*S**, 5*S**)-5-(3-(4-iodophenyl)-1-phenyl-1*H*-pyrazol-4-yl)-4-tosyl-4,5-dihydrooxazole **6e**.

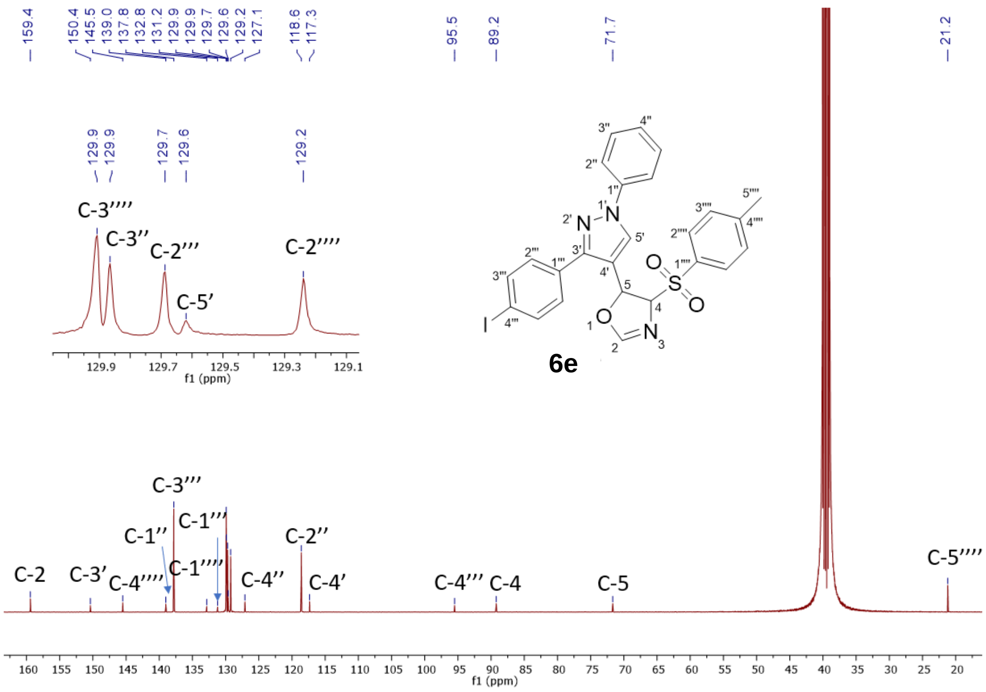

Figure S180. Expansion of ^{13}C NMR (125 MHz, DMSO-d$_6$) of (4*S**, 5*S**)-5-(3-(4-iodophenyl)-1-phenyl-1*H*-pyrazol-4-yl)-4-tosyl-4,5-dihydrooxazole **6e**.

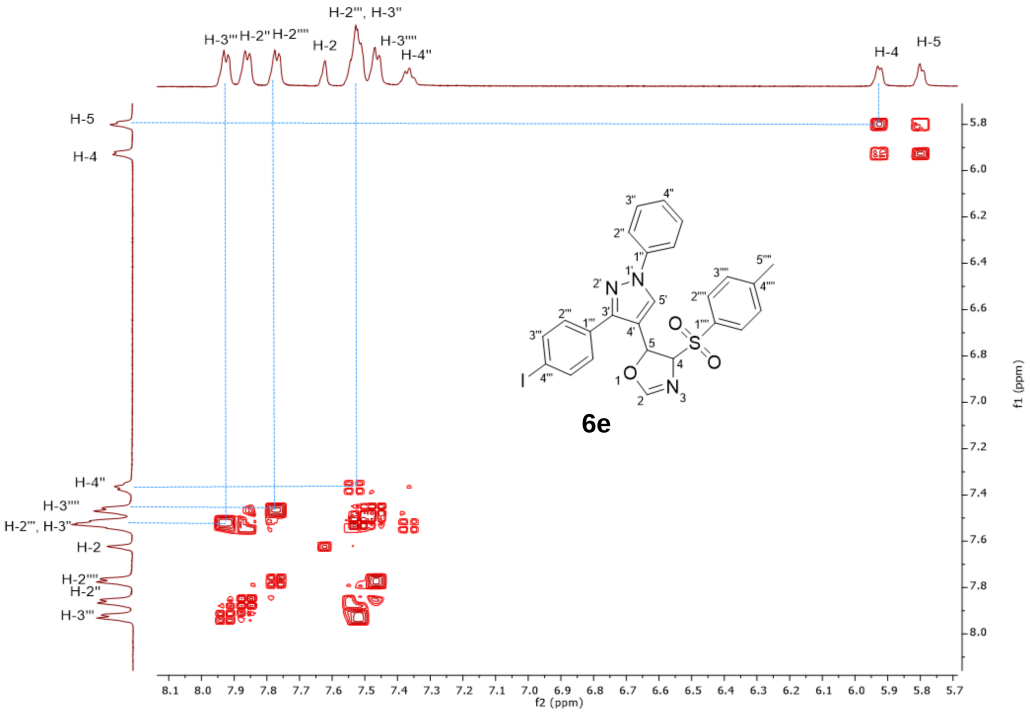

Figure S181. COSY experiment of (4*S**, 5*S**)-5-(3-(4-iodophenyl)-1-phenyl-1*H*-pyrazol-4-yl)-4-tosyl-4,5-dihydrooxazole **6e**.

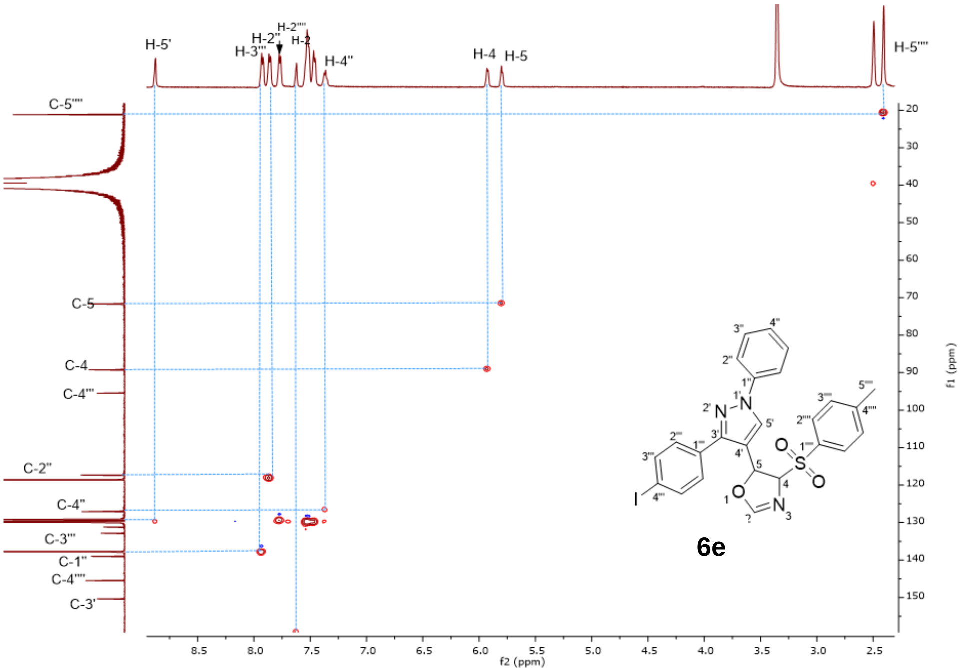

Figure S182. HSQC experiment of (4*S**, 5*S**)-5-(3-(4-iodophenyl)-1-phenyl-1*H*-pyrazol-4-yl)-4-tosyl-4,5-dihydrooxazole **6e**.

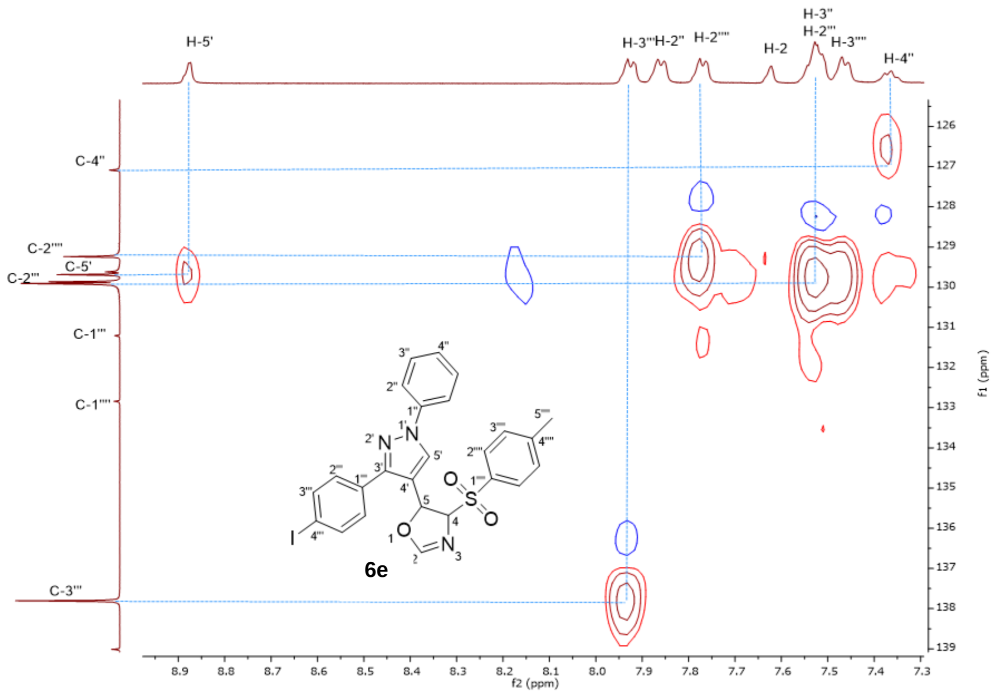

Figure S183. Expansion of HSQC experiment of (4*S**, 5*S**)-5-(3-(4-iodophenyl)-1-phenyl-1*H*-pyrazol-4-yl)-4-tosyl-4,5-dihydrooxazole **6e**.

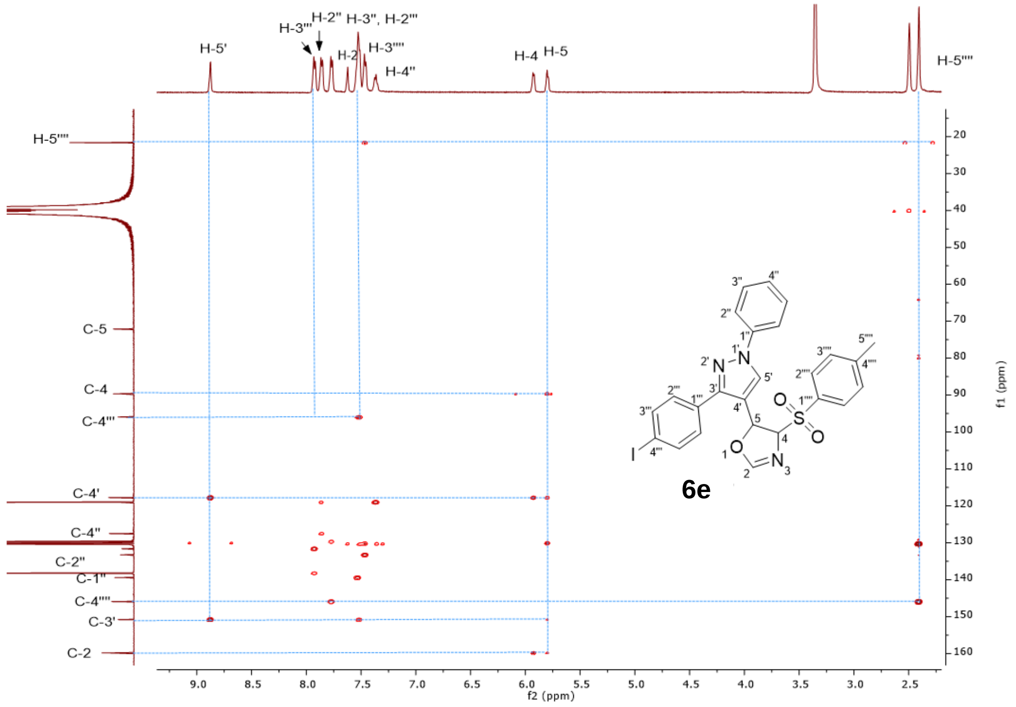

Figure S184. HMBC experiment of (4*S**, 5*S**)-5-(3-(4-iodophenyl)-1-phenyl-1*H*-pyrazol-4-yl)-4-tosyl-4,5-dihydrooxazole **6e**.

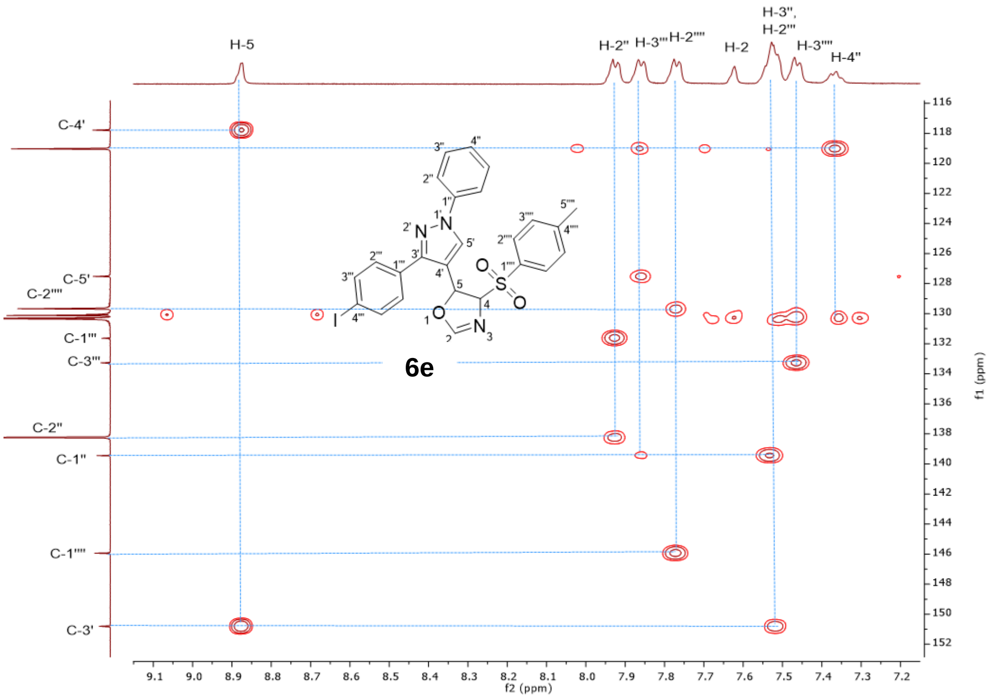

Figure S185. Expansion of HMBC experiment of (4*S**, 5*S**)-5-(3-(4-iodophenyl)-1-phenyl-1*H*-pyrazol-4-yl)-4-tosyl-4,5-dihydrooxazole **6e**.

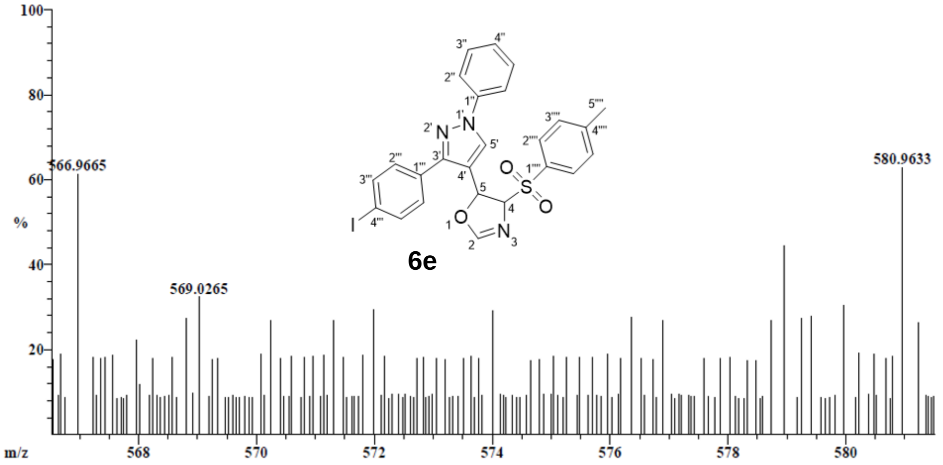

Selected Isotopes : $H_{0-20}C_{0-25}N_{0-3}O_{0-3}S_{0-1}I_{0-1}$ Error Limit : 5 ppm Unsaturation Limits : 0 to 50

Measured Mass	% Base	Formula	Calculated Mass	Error	Unsaturation
569.0265	32.3%	$C_{25}H_{20}N_3O_3SI$	569.0270	-0.9	17.0

Figure S186. HRMS of (4*S**, 5*S**)-5-(3-(4-iodophenyl)-1-phenyl-1*H*-pyrazol-4-yl)-4-tosyl-4,5-dihydrooxazole **6e**.

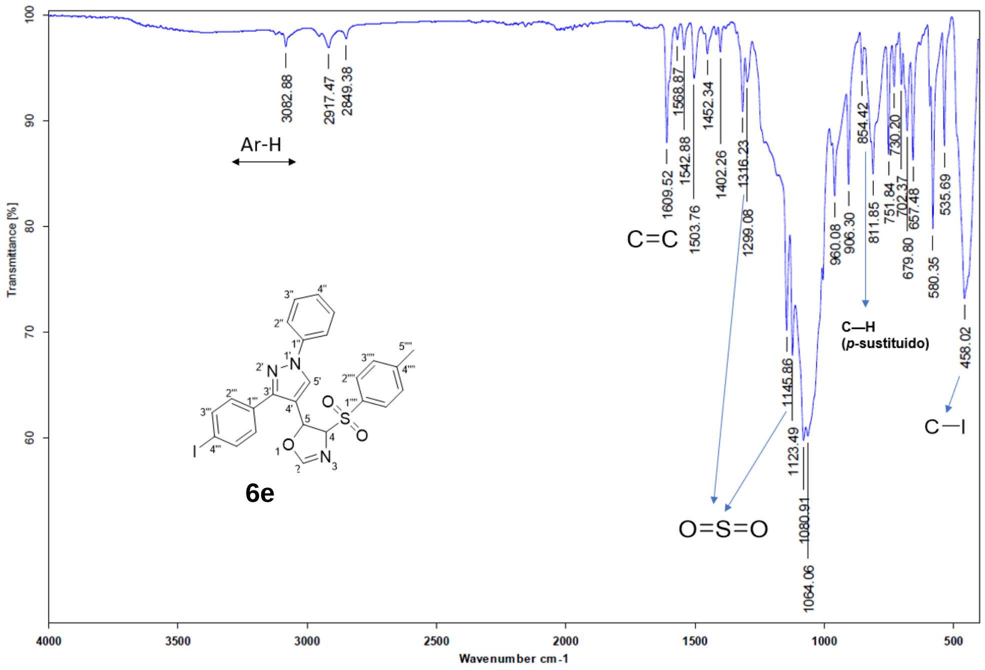

Figure S187. FT-IR of (4*S**, 5*S**)-5-(3-(4-iodophenyl)-1-phenyl-1*H*-pyrazol-4-yl)-4-tosyl-4,5-dihydrooxazole **6e**.

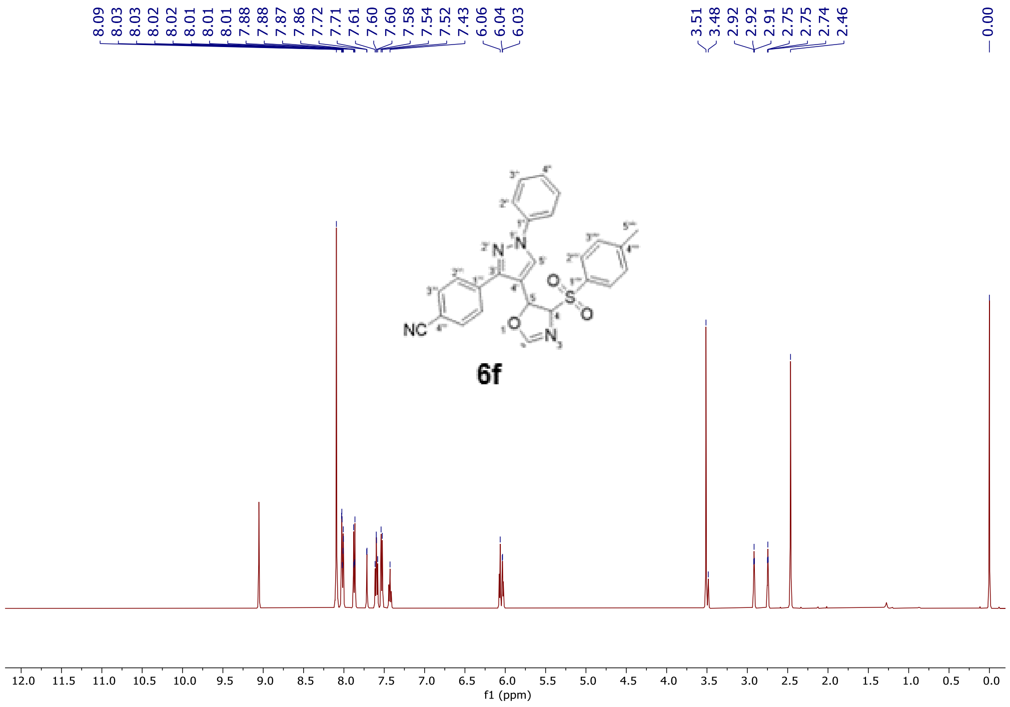

Figure S188. ^{1}H NMR (500 MHz, DMF-d$_7$) of (4*S**, 5*S**)-4-(1-phenyl-4-(4-tosyl-4,5-dihydrooxazol-5-yl)-1*H*-pyrazol-3-yl)benzonitrile **6f**.

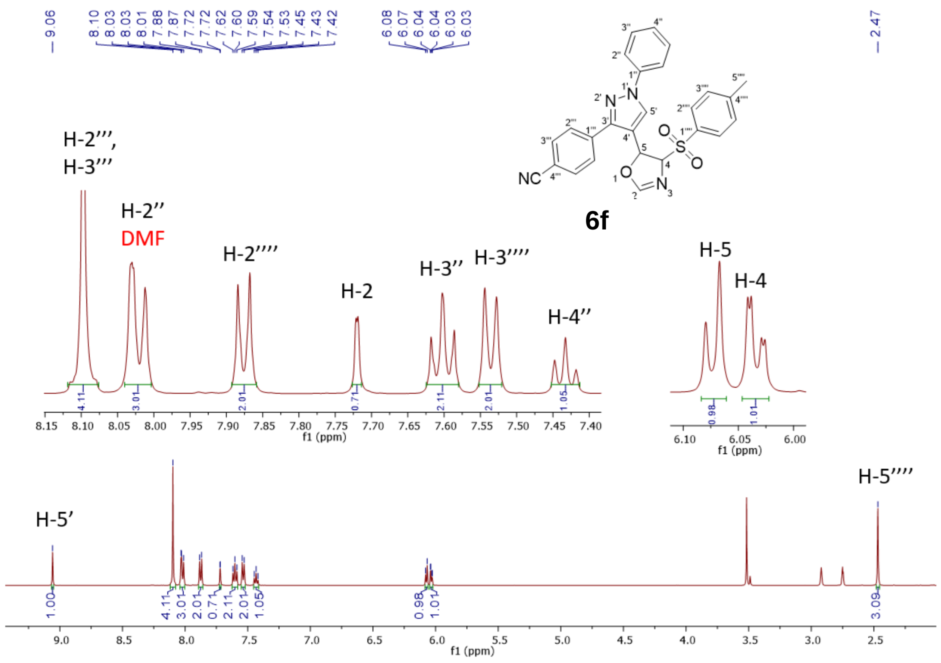

Figure S189. Expansion of ^{1}H NMR (500 MHz, DMF-d_7) of (4*S**, 5*S**)-4-(1-phenyl-4-(4-tosyl-4,5-dihydrooxazol-5-yl)-1*H*-pyrazol-3-yl)benzonitrile **6f**.

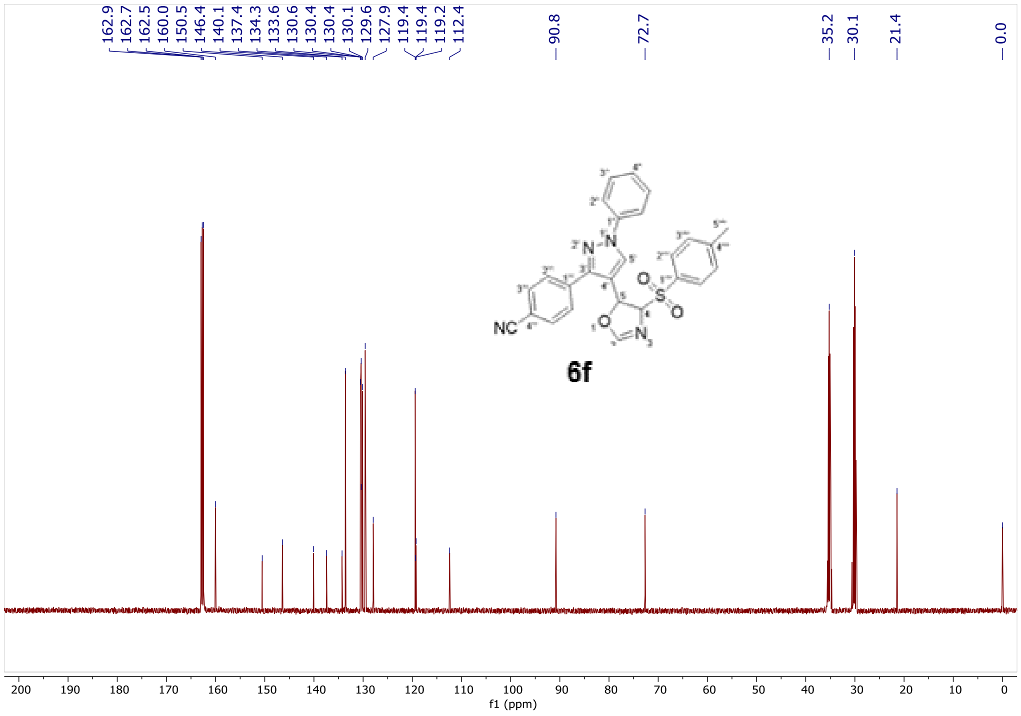

Figure S190. ^{13}C NMR (125 MHz, DMF-d_7) of (4*S**, 5*S**)-4-(1-phenyl-4-(4-tosyl-4,5-dihydrooxazol-5-yl)-1*H*-pyrazol-3-yl)benzonitrile **6f**.

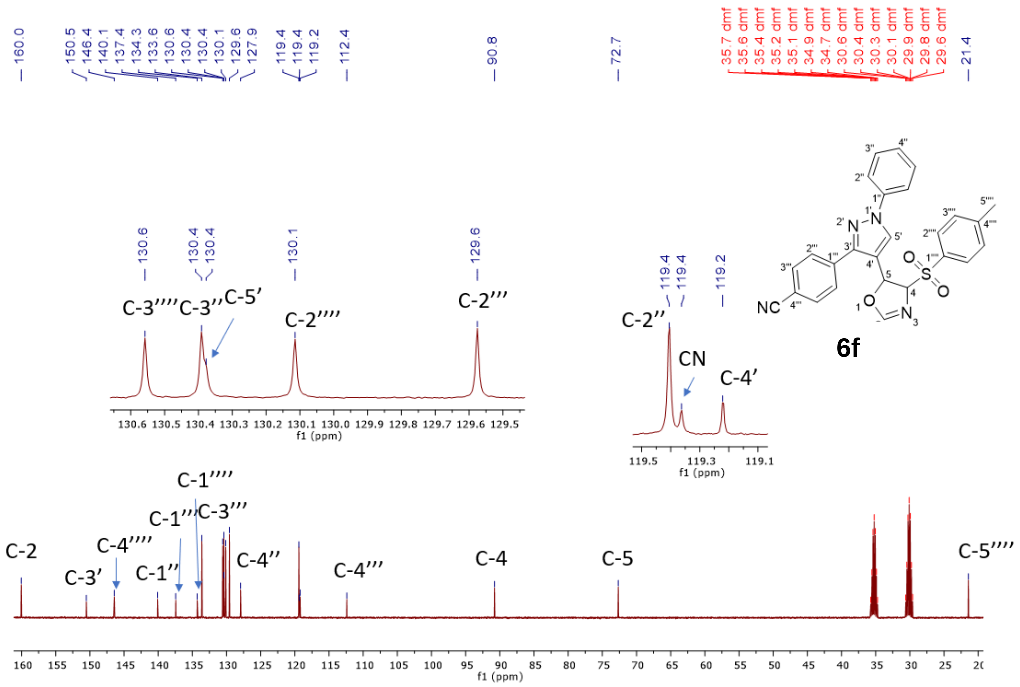

Figure S191. Expansion of ^{13}C NMR (125 MHz, DMF-d$_7$) of (4*S**, 5*S**)-4-(1-phenyl-4-(4-tosyl-4,5-dihydrooxazol-5-yl)-1*H*-pyrazol-3-yl)benzonitrile **6f**.

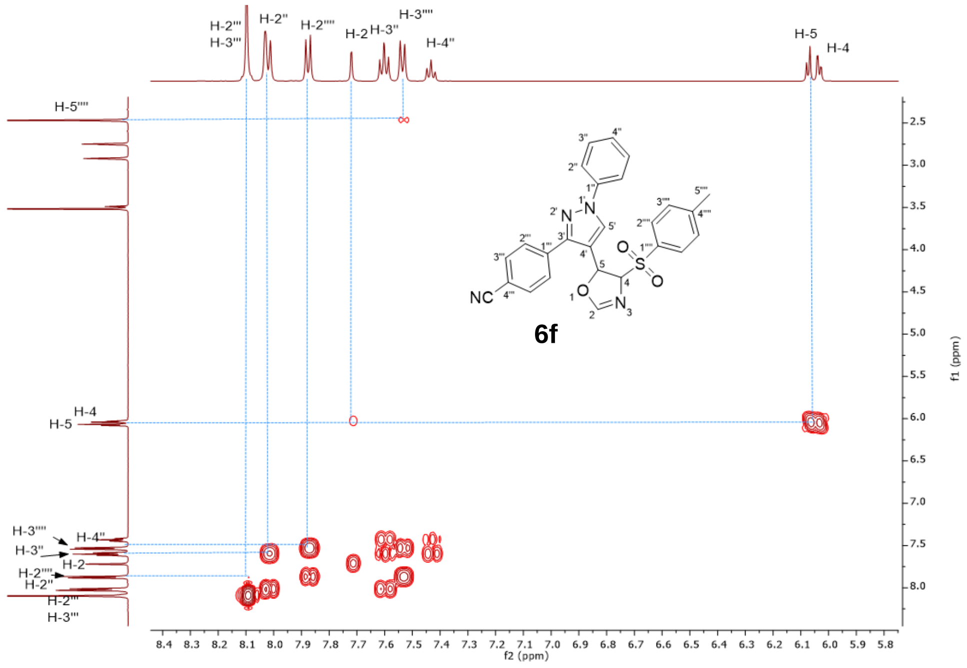

Figure S192. COSY experiment of (4*S**, 5*S**)-4-(1-phenyl-4-(4-tosyl-4,5-dihydrooxazol-5-yl)-1*H*-pyrazol-3-yl)benzonitrile **6f**.

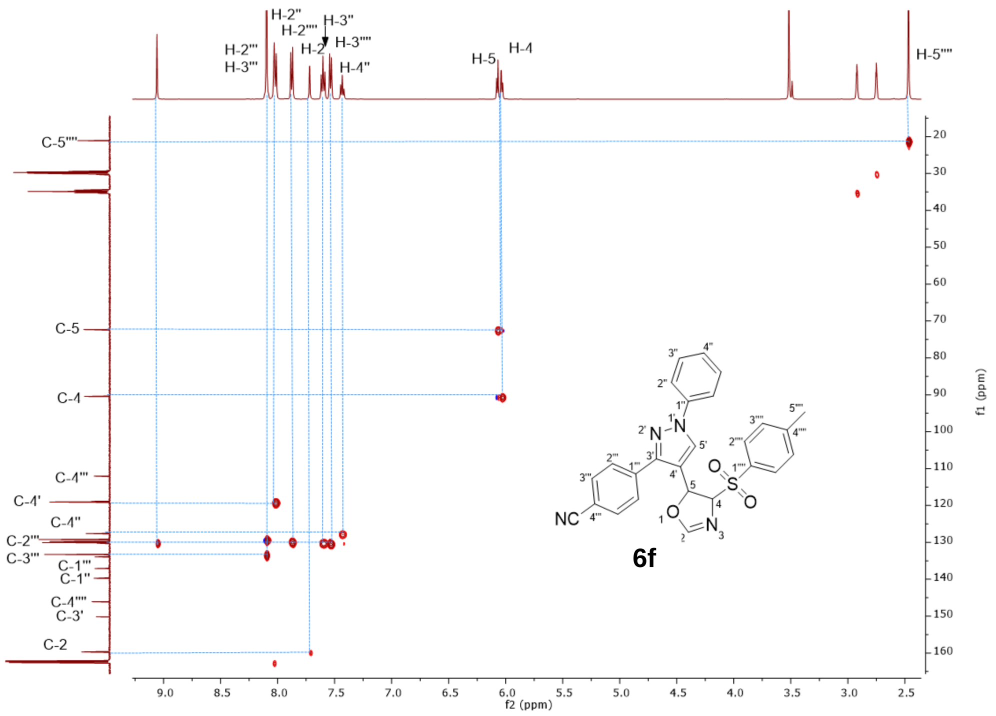

Figure S193. HSQC experiment of (4*S**, 5*S**)-4-(1-phenyl-4-(4-tosyl-4,5-dihydrooxazol-5-yl)-1*H*-pyrazol-3-yl)benzonitrile **6f**.

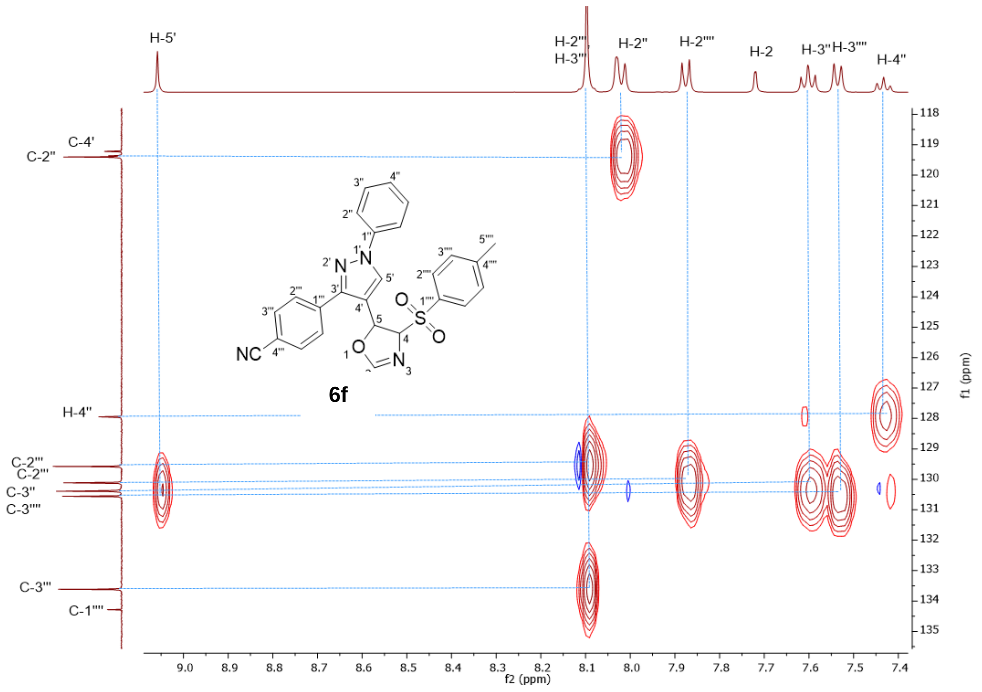

Figure S194. Expansion of HSQC experiment of (4*S**, 5*S**)-4-(1-phenyl-4-(4-tosyl-4,5-dihydrooxazol-5-yl)-1*H*-pyrazol-3-yl)benzonitrile **6f**.

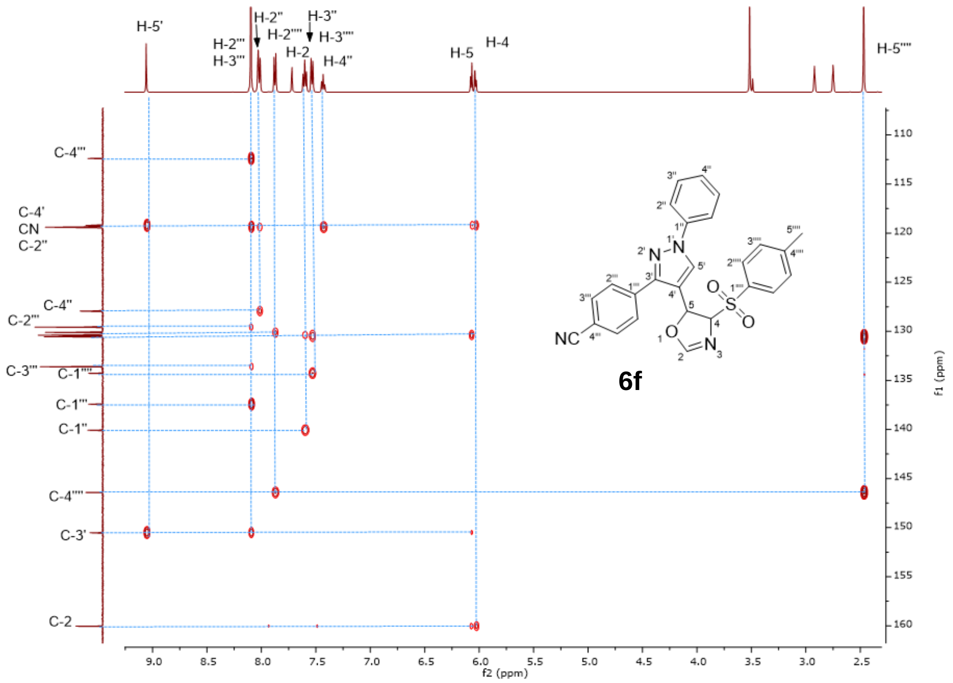

Figure S195. HMBC experiment of (4*S**, 5*S**)-4-(1-phenyl-4-(4-tosyl-4,5-dihydrooxazol-5-yl)-1*H*-pyrazol-3-yl)benzonitrile **6f**.

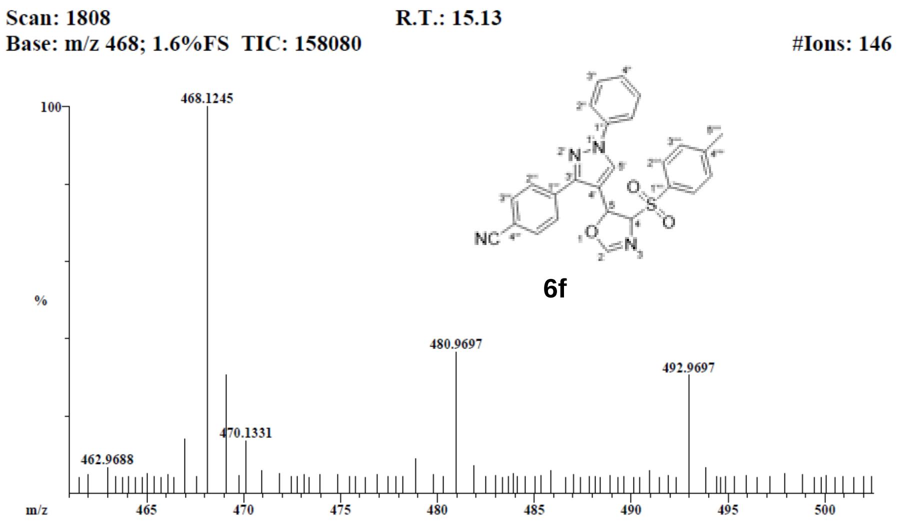

Selected Isotopes : $C_{0-26}H_{0-20}N_{0-4}O_{0-3}S_{0-1}$ Error Limit : 5 ppm Unsaturation Limits : 0 to 50

Measured Mass	% Base	Formula	Calculated Mass	Error	Unsaturation
468.1245	100.0%	$C_{26}H_{20}N_4O_3S$	468.1256	-2.4	19.0

Figure S196. HRMS of (4*S**, 5*S**)-4-(1-phenyl-4-(4-tosyl-4,5-dihydrooxazol-5-yl)-1*H*-pyrazol-3-yl)benzonitrile **6f**.

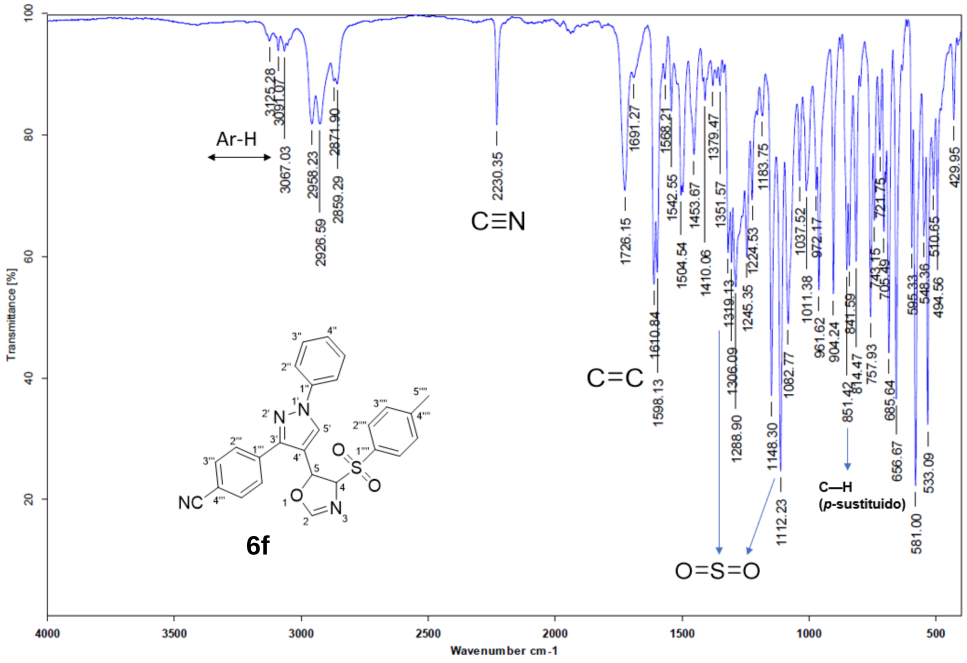

Figure S197. FT-IR of (4*S**, 5*S**)-4-(1-phenyl-4-(4-tosyl-4,5-dihydrooxazol-5-yl)-1*H*-pyrazol-3-yl)benzonitrile **6f**.

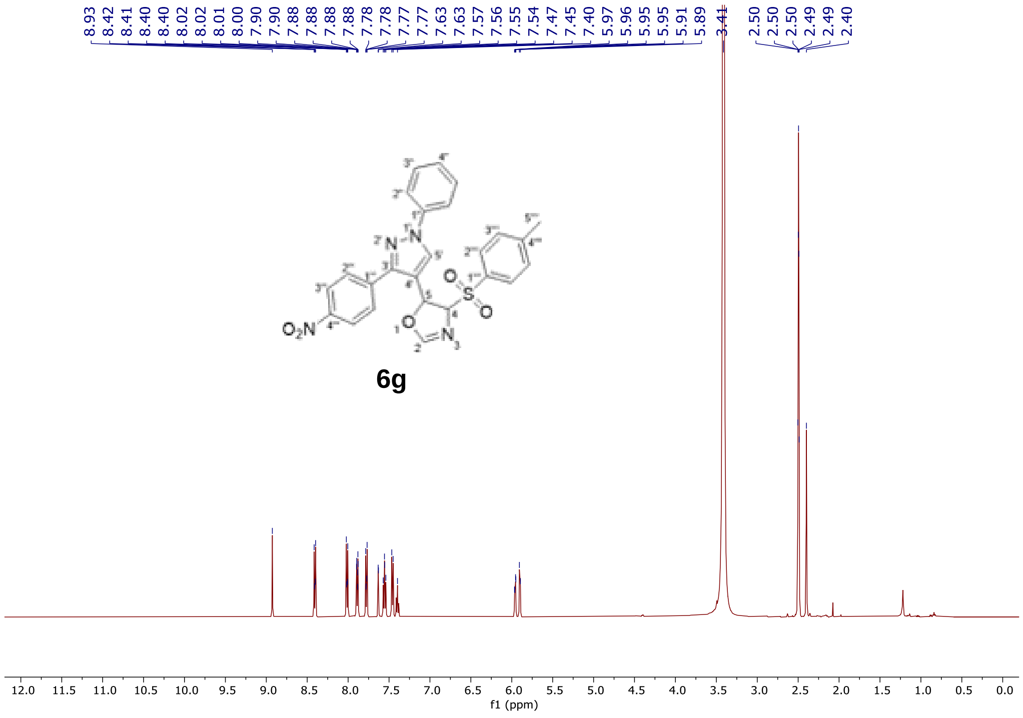

Figure S198. ^{1}H NMR (500 MHz, DMSO-d_6) of (4*S**, 5*S**)-5-(3-(4-nitrophenyl)-1-phenyl-1*H*-pyrazol-4-yl)-4-tosyl-4,5-dihydrooxazole **6g**.

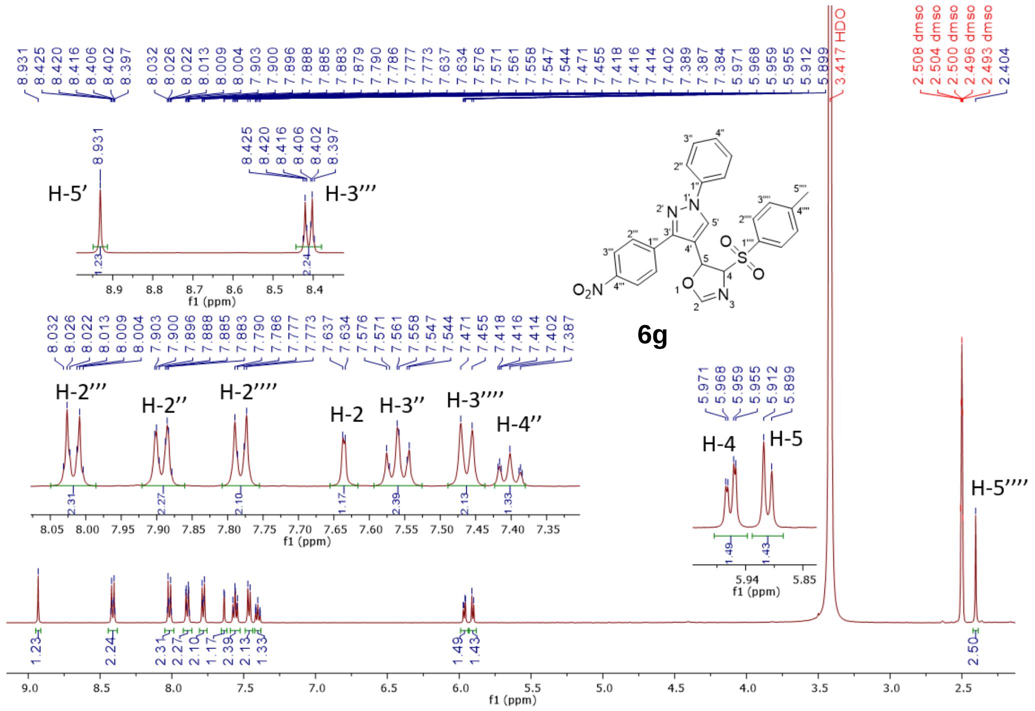

Figure S199. Expansion of ^{1}H NMR (500 MHz, DMSO-d$_6$) of (4*S**, 5*S**)-5-(3-(4-nitrophenyl)-1-phenyl-1*H*-pyrazol-4-yl)-4-tosyl-4,5-dihydrooxazole **6g**.

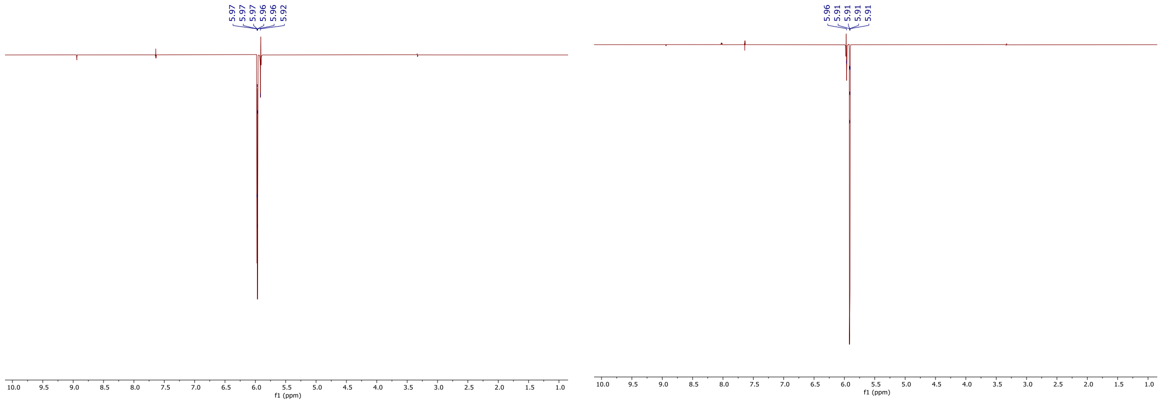

Figure S200. NOESY1D experiment of (4*S**, 5*S**)-5-(3-(4-nitrophenyl)-1-phenyl-1*H*-pyrazol-4-yl)-4-tosyl-4,5-dihydrooxazole **6g** (H-4 and H-5 were irradiated).

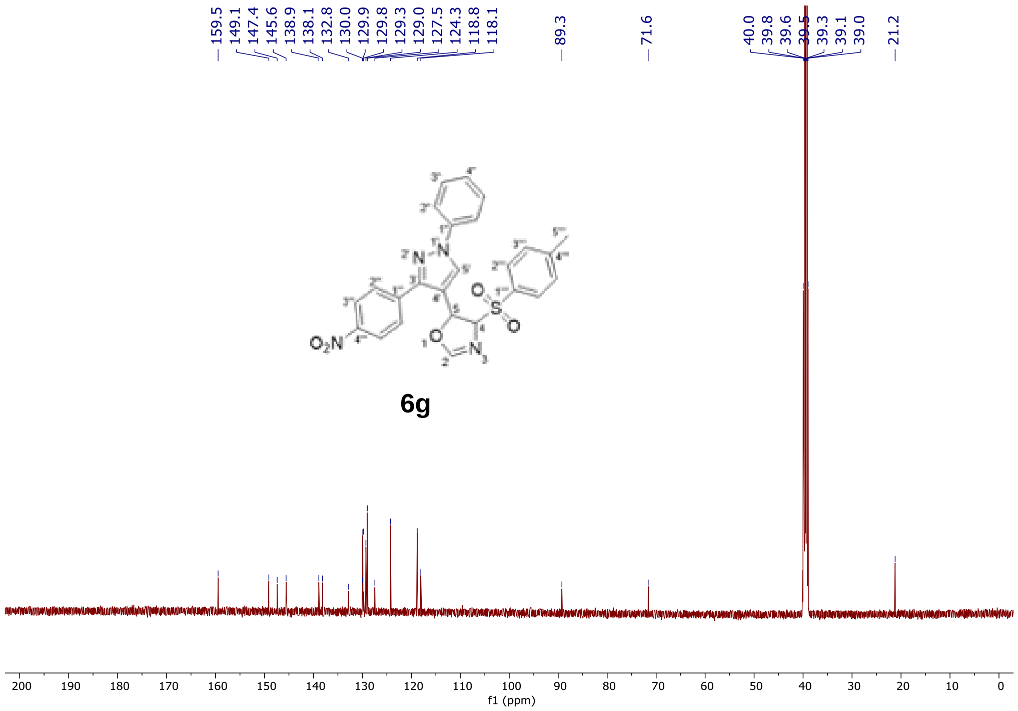

Figure S201. ^{13}C NMR (125 MHz, DMSO-d$_6$) of (4*S**, 5*S**)-5-(3-(4-nitrophenyl)-1-phenyl-1*H*-pyrazol-4-yl)-4-tosyl-4,5-dihydrooxazole **6g**.

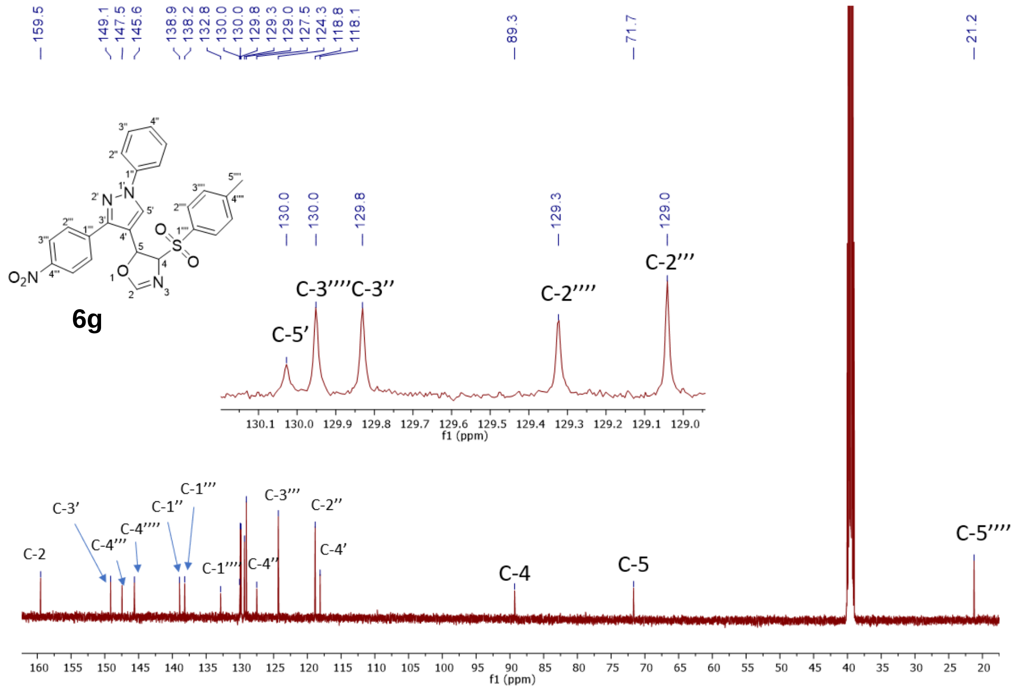

Figure S202. Expansion of ^{13}C NMR (125 MHz, DMSO-d$_6$) of (4*S**, 5*S**)-5-(3-(4-nitrophenyl)-1-phenyl-1*H*-pyrazol-4-yl)-4-tosyl-4,5-dihydrooxazole **6g**.

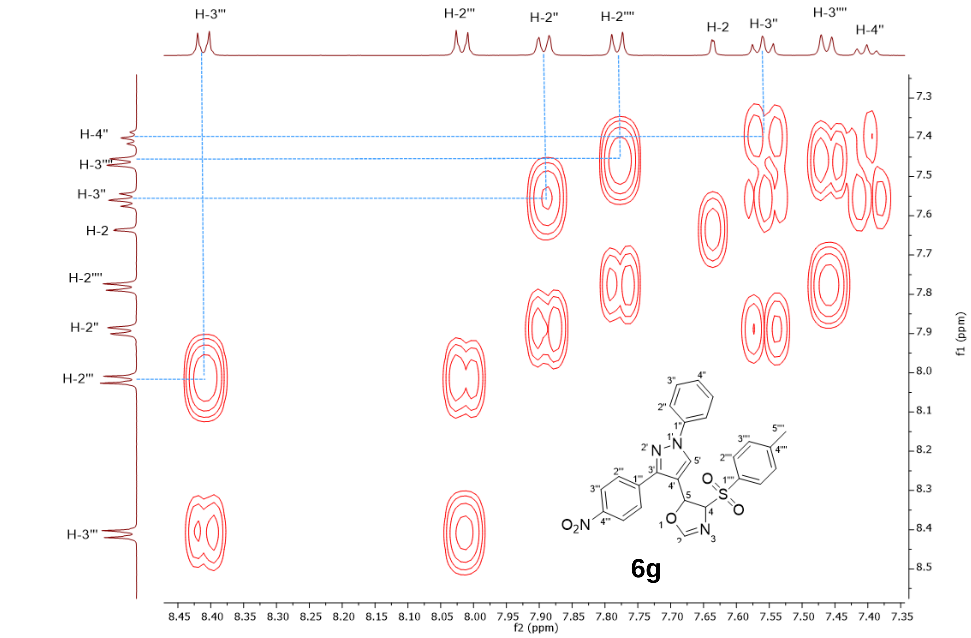

Figure S203. COSY experiment of (4*S**, 5*S**)-5-(3-(4-nitrophenyl)-1-phenyl-1*H*-pyrazol-4-yl)-4-tosyl-4,5-dihydrooxazole **6g**.

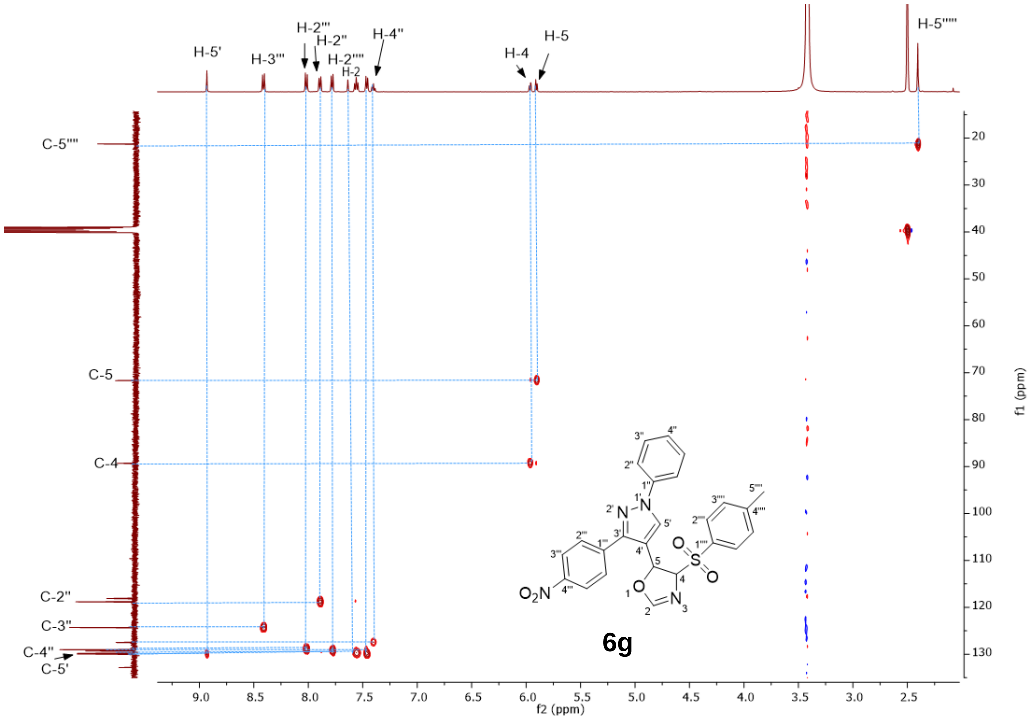

Figure S204. HSQC experiment of (4*S**, 5*S**)-5-(3-(4-nitrophenyl)-1-phenyl-1*H*-pyrazol-4-yl)-4-tosyl-4,5-dihydrooxazole **6g**.

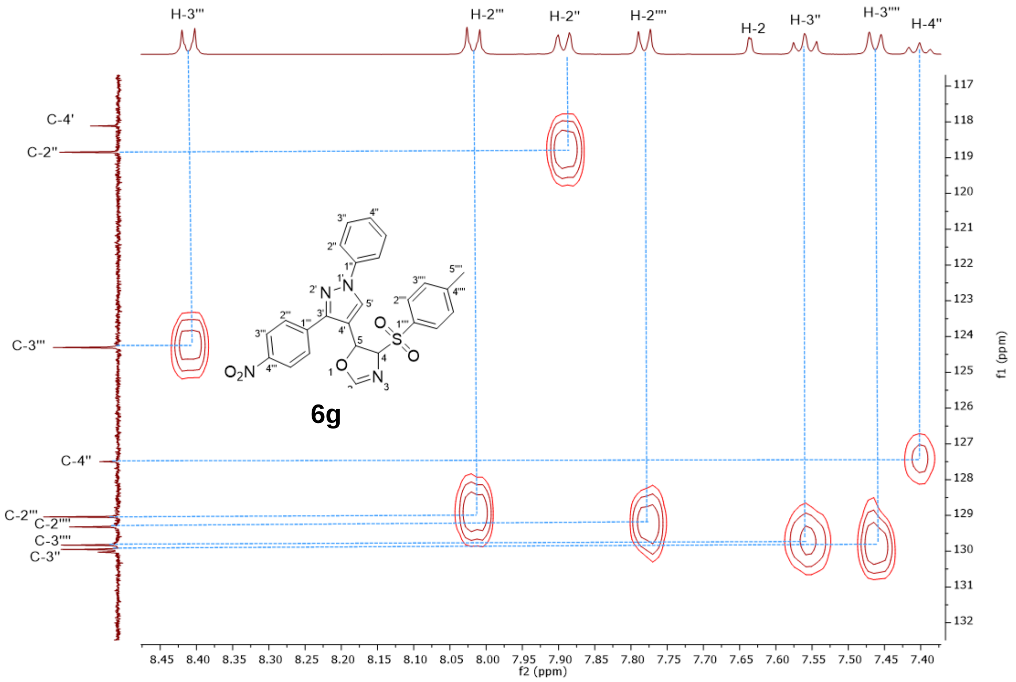

Figure S205. Expansion of HSQC experiment of (4*S**, 5*S**)-5-(3-(4-nitrophenyl)-1-phenyl-1*H*-pyrazol-4-yl)-4-tosyl-4,5-dihydrooxazole **6g**.

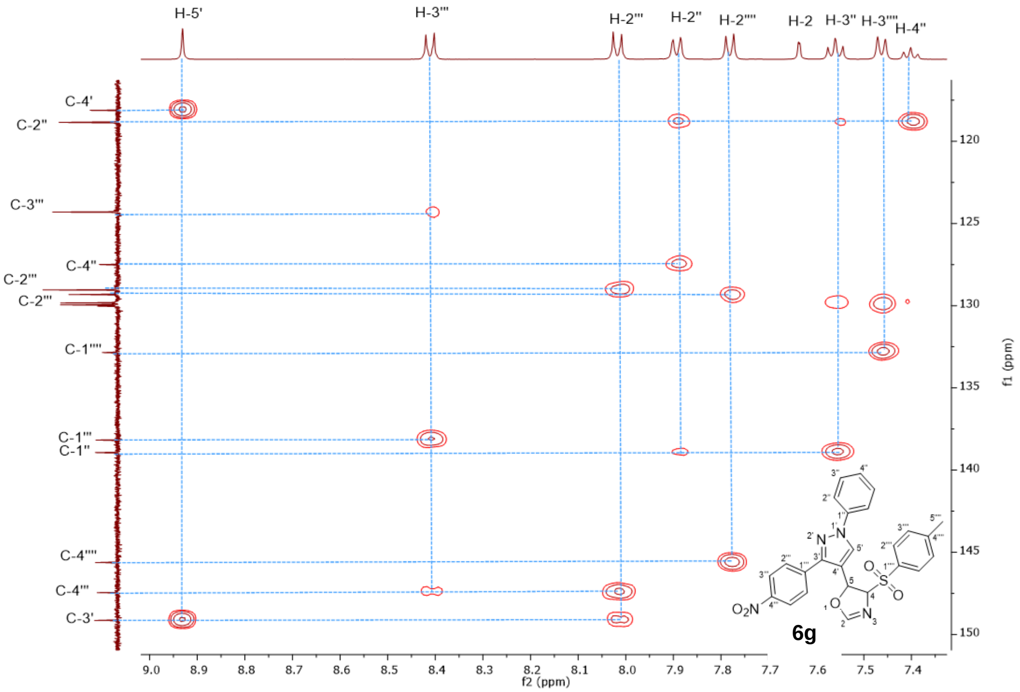

Figure S206. Expansion of HMBC experiment of (4*S**, 5*S**)-5-(3-(4-nitrophenyl)-1-phenyl-1*H*-pyrazol-4-yl)-4-tosyl-4,5-dihydrooxazole **6g**.

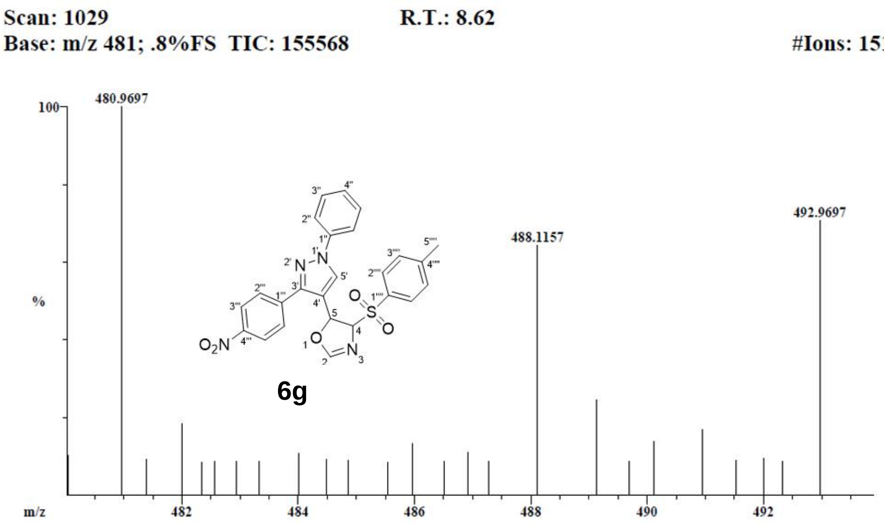

Selected Isotopes : $H_{0-20}C_{0-25}N_{0-4}O_{0-5}S_{0-1}$ Error Limit : 5 ppm Unsaturation Limits : 0 to 50

Measured Mass	% Base	Formula	Calculated Mass	Error	Unsaturation
488.1157	64.4%	$C_{25}H_{20}N_4O_5S$	488.1154	0.6	18.0

Figure S207. HRMS of (4*S**, 5*S**)-5-(3-(4-nitrophenyl)-1-phenyl-1*H*-pyrazol-4-yl)-4-tosyl-4,5-dihydrooxazole **6g**.

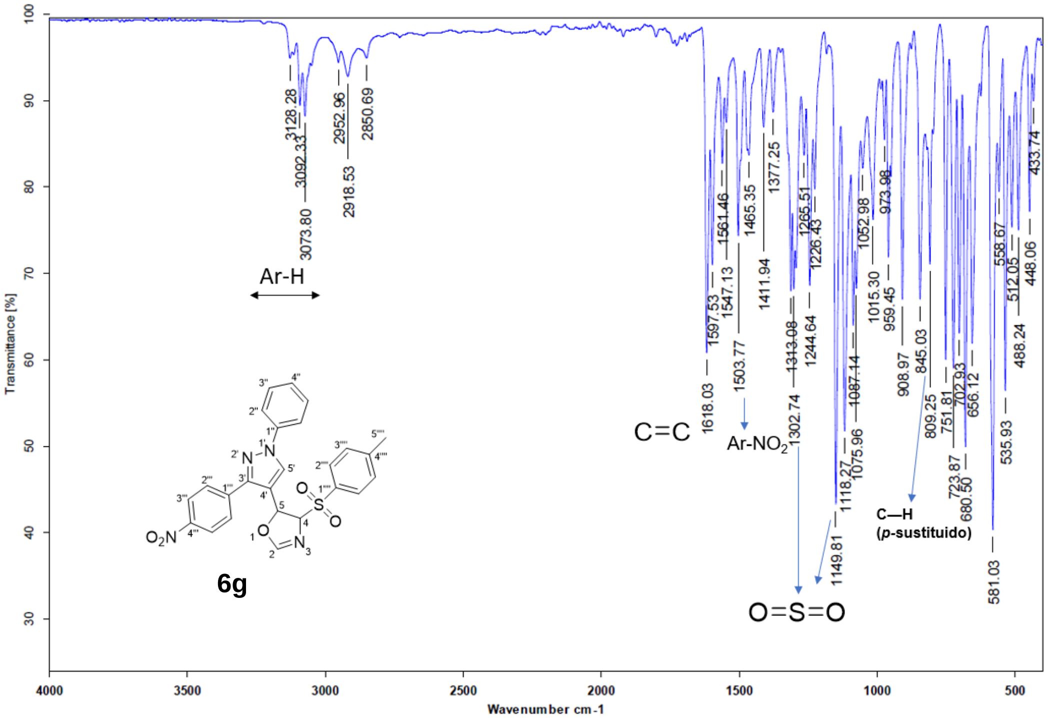

Figure S208. FT-IR of (4*S**, 5*S**)-5-(3-(4-nitrophenyl)-1-phenyl-1*H*-pyrazol-4-yl)-4-tosyl-4,5-dihydrooxazole **6g**.

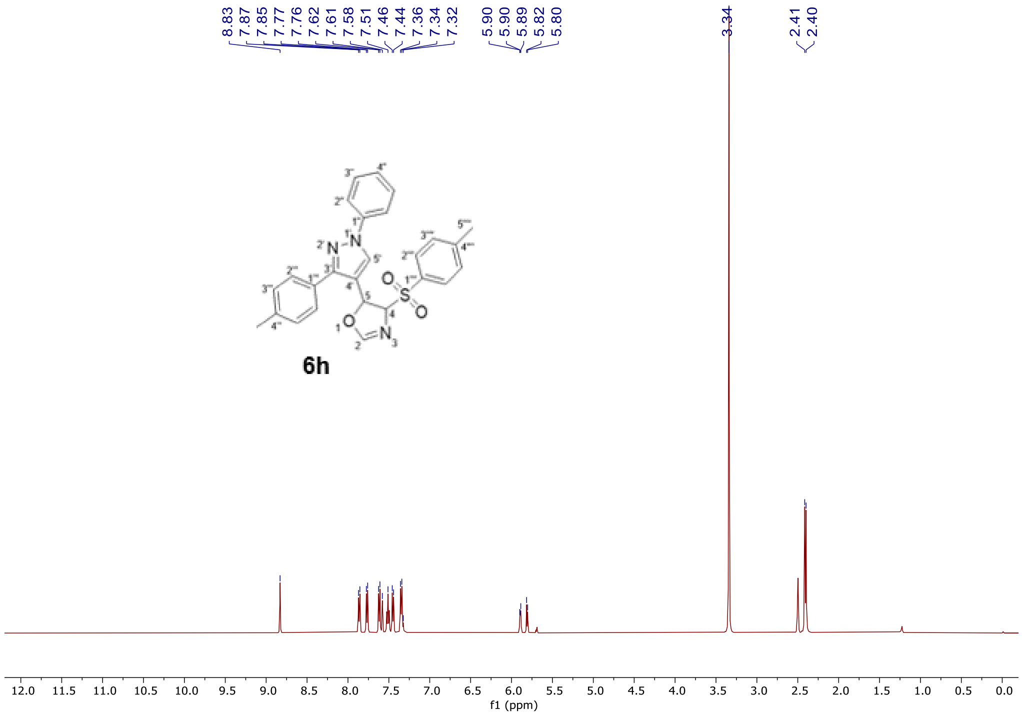

Figure S209. ^{1}H NMR (500 MHz, DMSO-d$_6$) of (4*S**, 5*S**)-5-(1-phenyl-3-(*p*-tolyl)-1*H*-pyrazol-4-yl)-4-tosyl-4,5-dihydrooxazole **6h**.

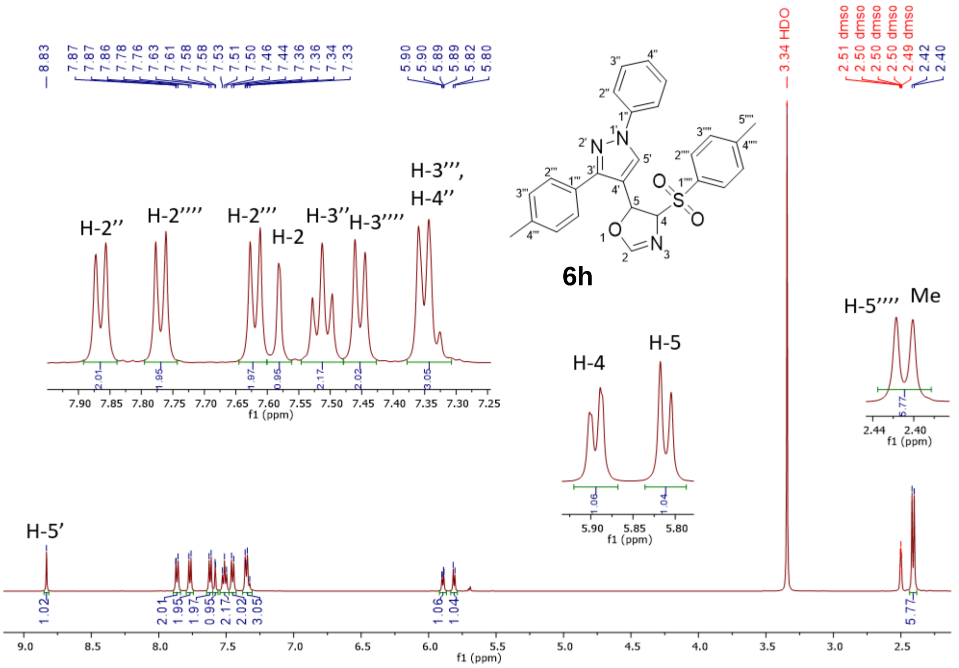

Figure S210. Expansion of ^{1}H NMR (500 MHz, DMSO-d$_6$) of (4*S**, 5*S**)-5-(1-phenyl-3-(*p*-tolyl)-1*H*-pyrazol-4-yl)-4-tosyl-4,5-dihydrooxazole **6h**.

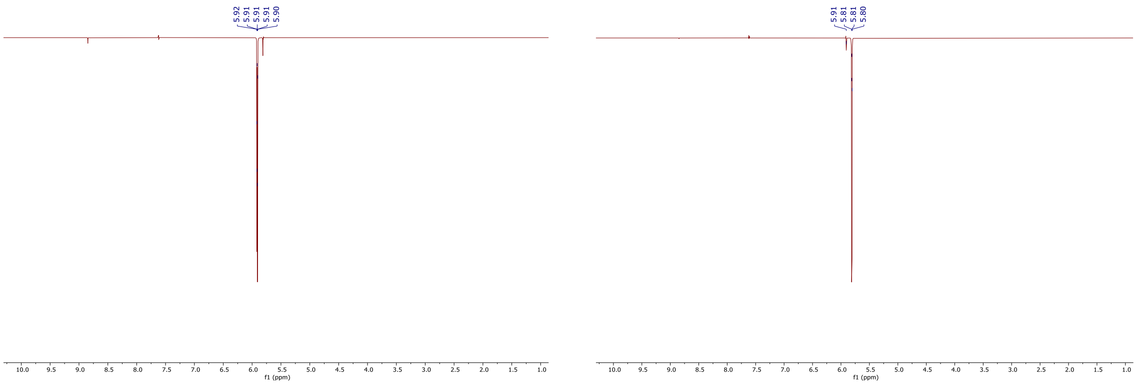

Figure S211. NOESY experiment of (4*S**, 5*S**)-5-(1-phenyl-3-(*p*-tolyl)-1*H*-pyrazol-4-yl)-4-tosyl-4,5-dihydrooxazole **6h** (H-4 and H-5 were irradiated).

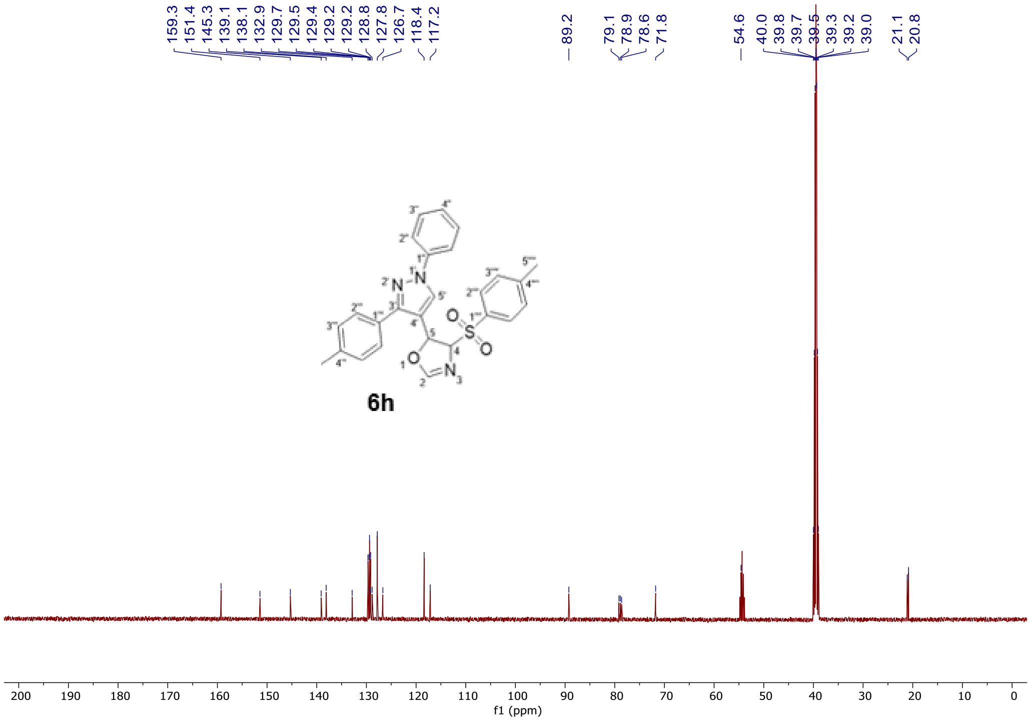

Figure S212. ^{13}C NMR (125 MHz, DMSO-d$_6$) of (4*S**, 5*S**)-5-(1-phenyl-3-(*p*-tolyl)-1*H*-pyrazol-4-yl)-4-tosyl-4,5-dihydrooxazole **6h**.

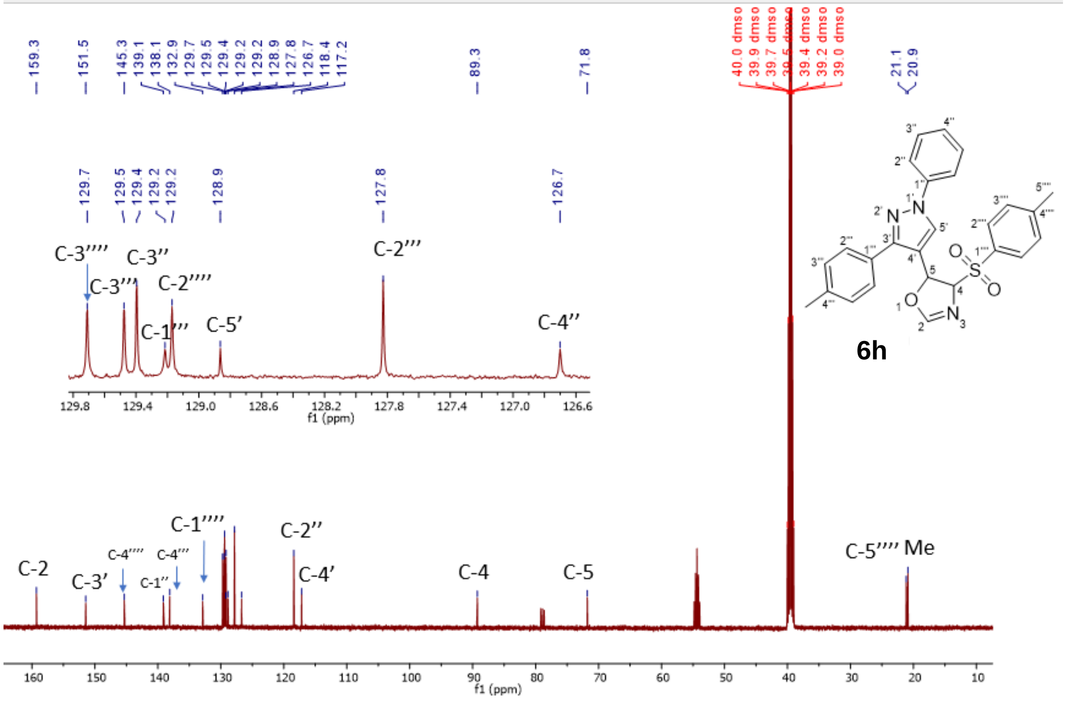

Figure S213. Expansion of ^{13}C NMR (125 MHz, DMSO-d_6) of (4*S**, 5*S**)-5-(1-phenyl-3-(*p*-tolyl)-1*H*-pyrazol-4-yl)-4-tosyl-4,5-dihydrooxazole **6h**.

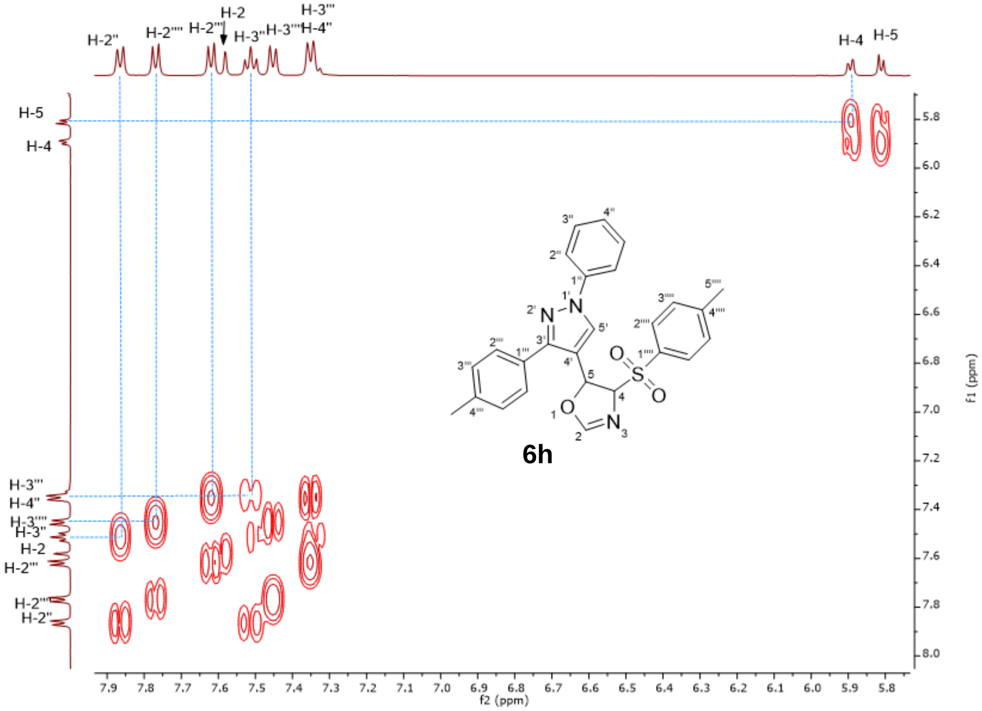

Figure S214. COSY experiment of (4*S**, 5*S**)-5-(1-phenyl-3-(*p*-tolyl)-1*H*-pyrazol-4-yl)-4-tosyl-4,5-dihydrooxazole **6h**.

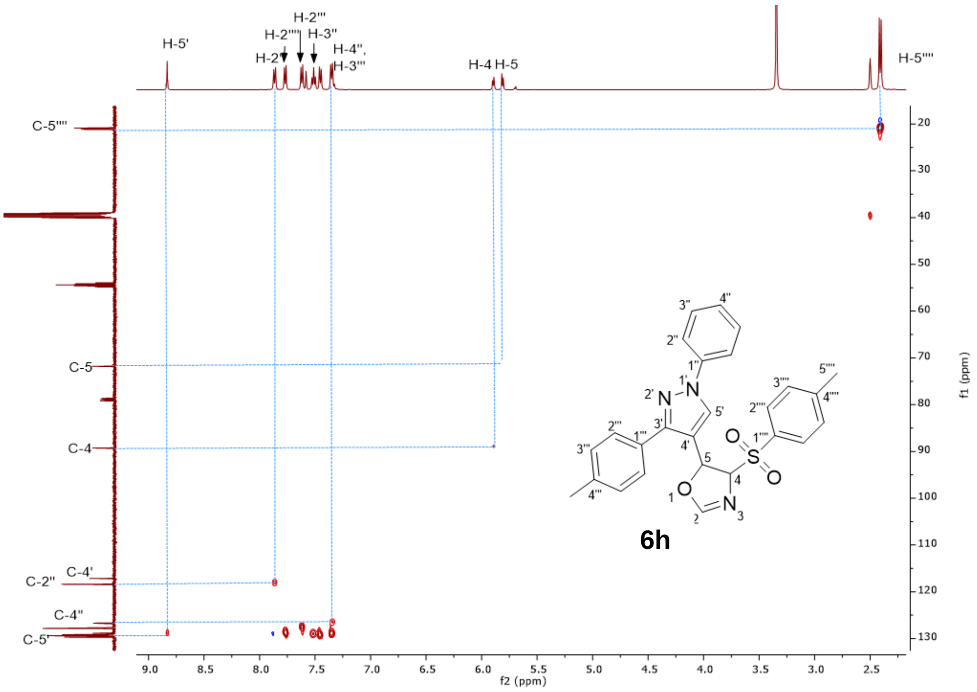

Figure S215. HSQC experiment of (4*S**, 5*S**)-5-(1-phenyl-3-(*p*-tolyl)-1*H*-pyrazol-4-yl)-4-tosyl-4,5-dihydrooxazole **6h**.

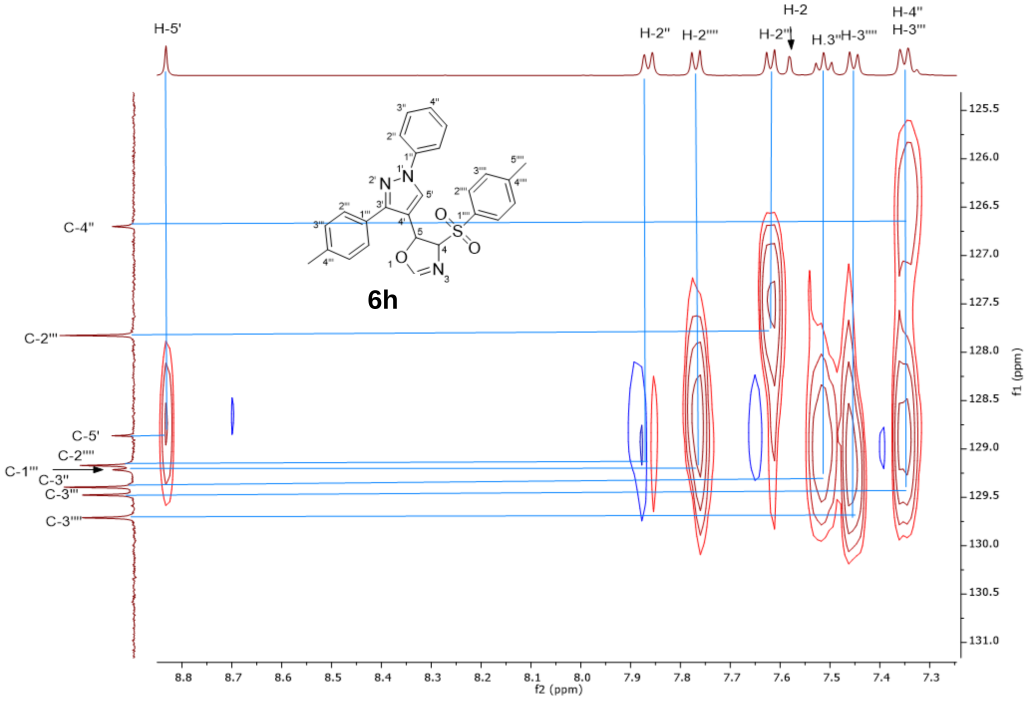

Figure S216. Expansion of HSQC experiment of (4*S**, 5*S**)-5-(1-phenyl-3-(*p*-tolyl)-1*H*-pyrazol-4-yl)-4-tosyl-4,5-dihydrooxazole **6h**.

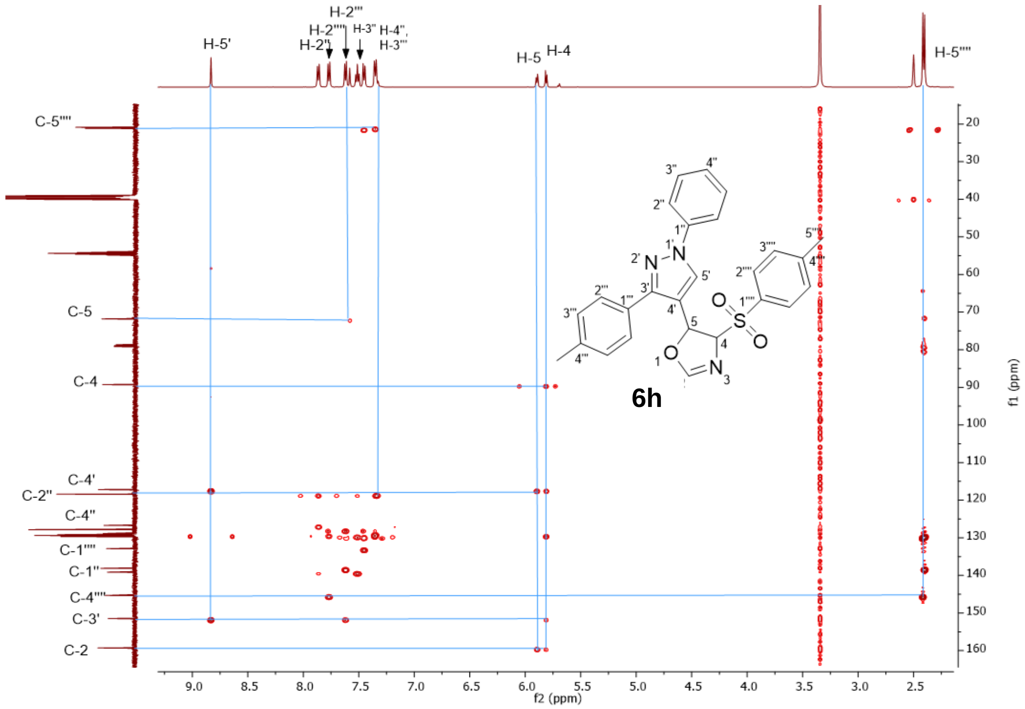

Figure S217. HMBC experiment of (4*S**, 5*S**)-5-(1-phenyl-3-(*p*-tolyl)-1*H*-pyrazol-4-yl)-4-tosyl-4,5-dihydrooxazole **6h**.

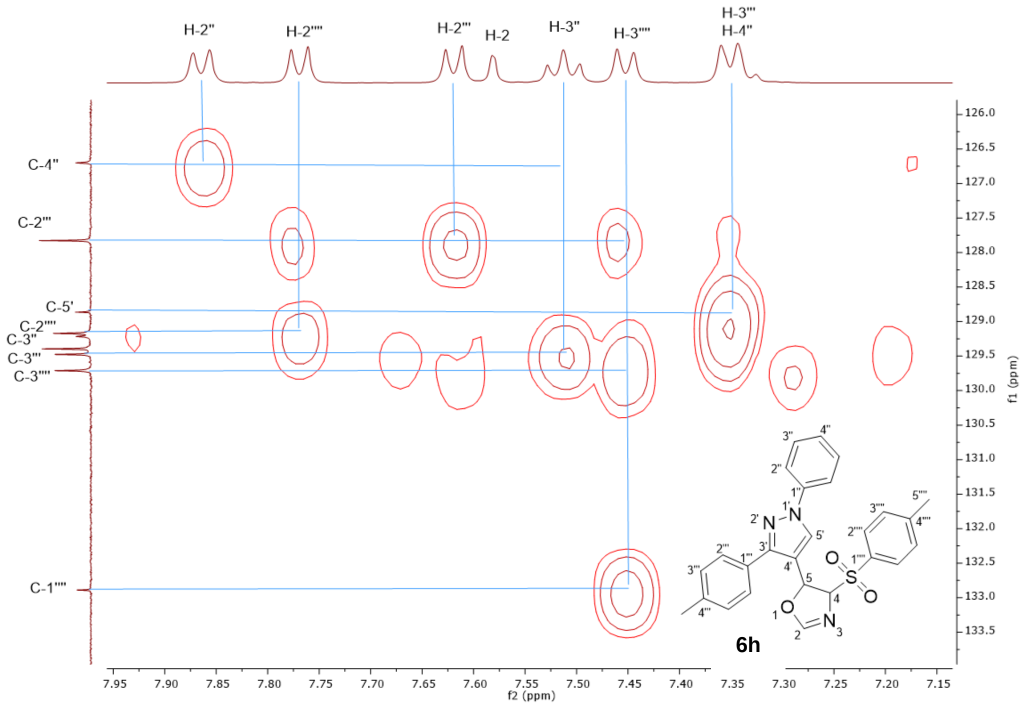

Figure S218. Expansion of HMBC experiment of (4*S**, 5*S**)-5-(1-phenyl-3-(*p*-tolyl)-1*H*-pyrazol-4-yl)-4-tosyl-4,5-dihydrooxazole **6h**.

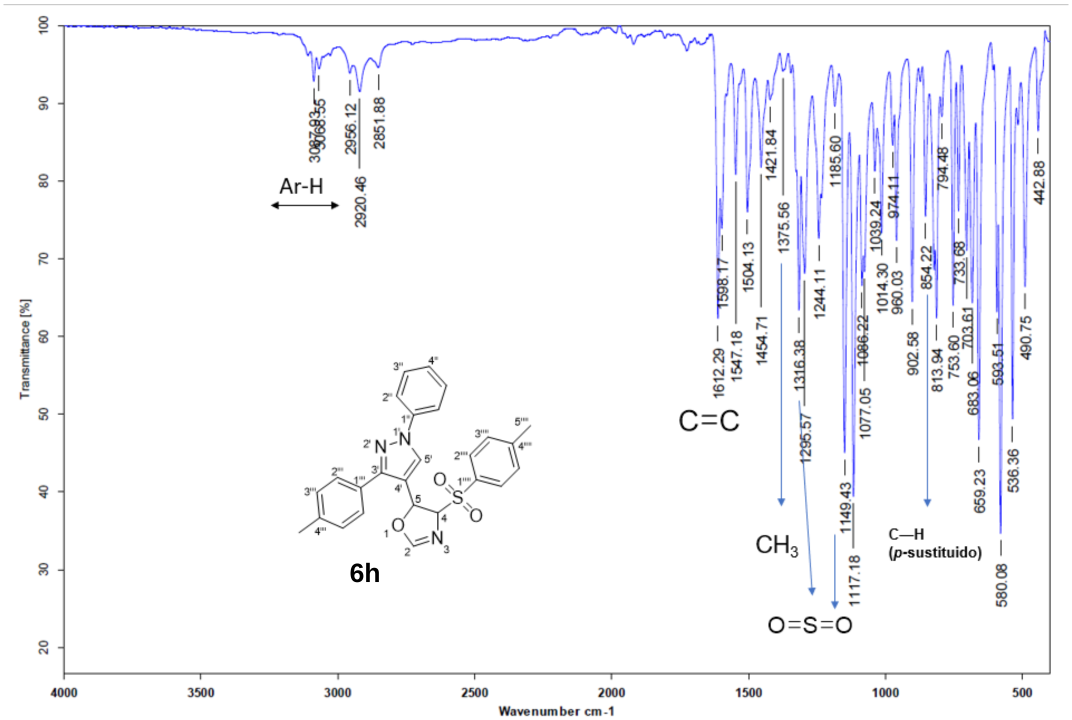

Figure S219. FT-IR of (4*S**, 5*S**)-5-(1-phenyl-3-(*p*-tolyl)-1*H*-pyrazol-4-yl)-4-tosyl-4,5-dihydrooxazole **6h**.

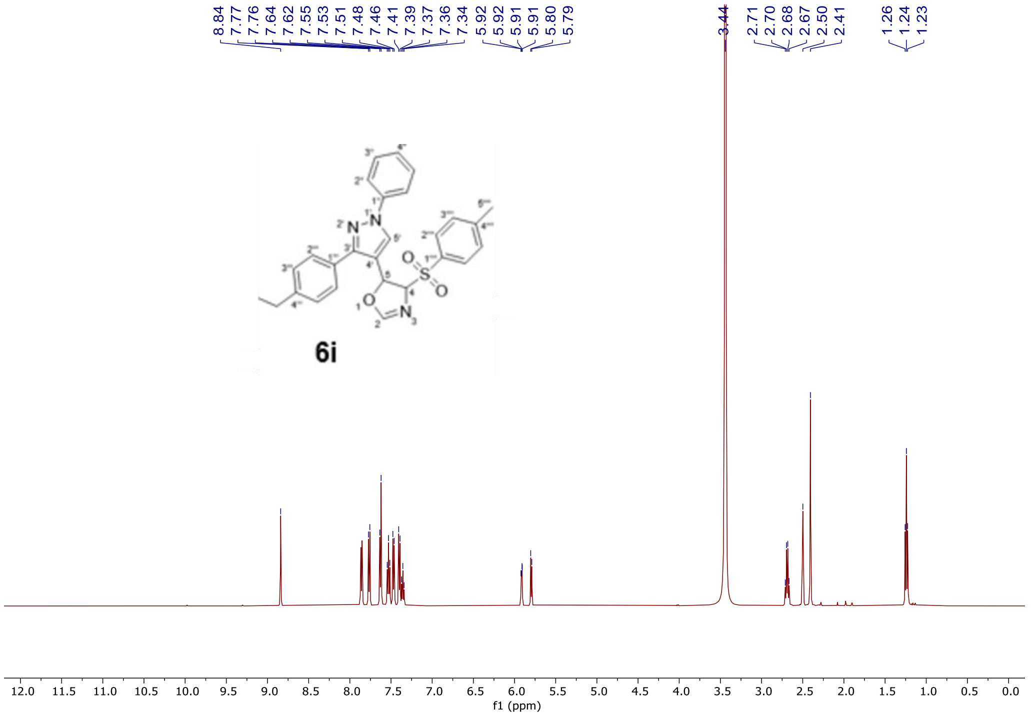

Figure S220. ^{1}H NMR (500 MHz, DMSO-d$_6$) of (4*S**, 5*S**)-5-(3-(4-ethylphenyl)-1-phenyl-1*H*-pyrazol-4-yl)-4-tosyl-4,5-dihydrooxazole **6i**.

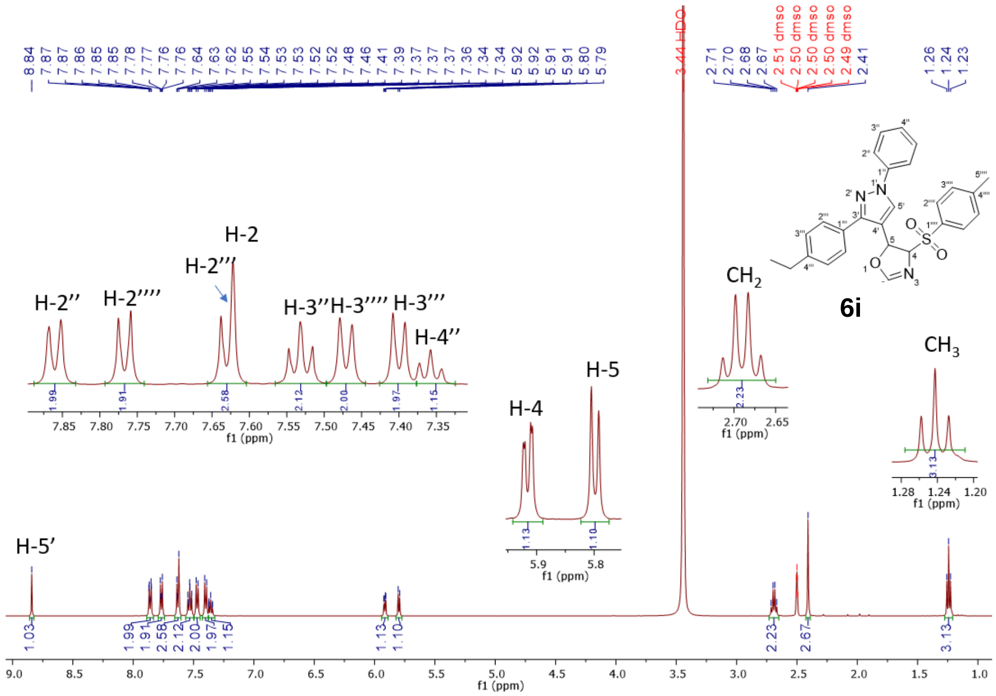

Figure S221. Expansion of ^{1}H NMR (500 MHz, DMSO-d$_6$) of (4*S**, 5*S**)-5-(3-(4-ethylphenyl)-1-phenyl-1*H*-pyrazol-4-yl)-4-tosyl-4,5-dihydrooxazole **6i**.

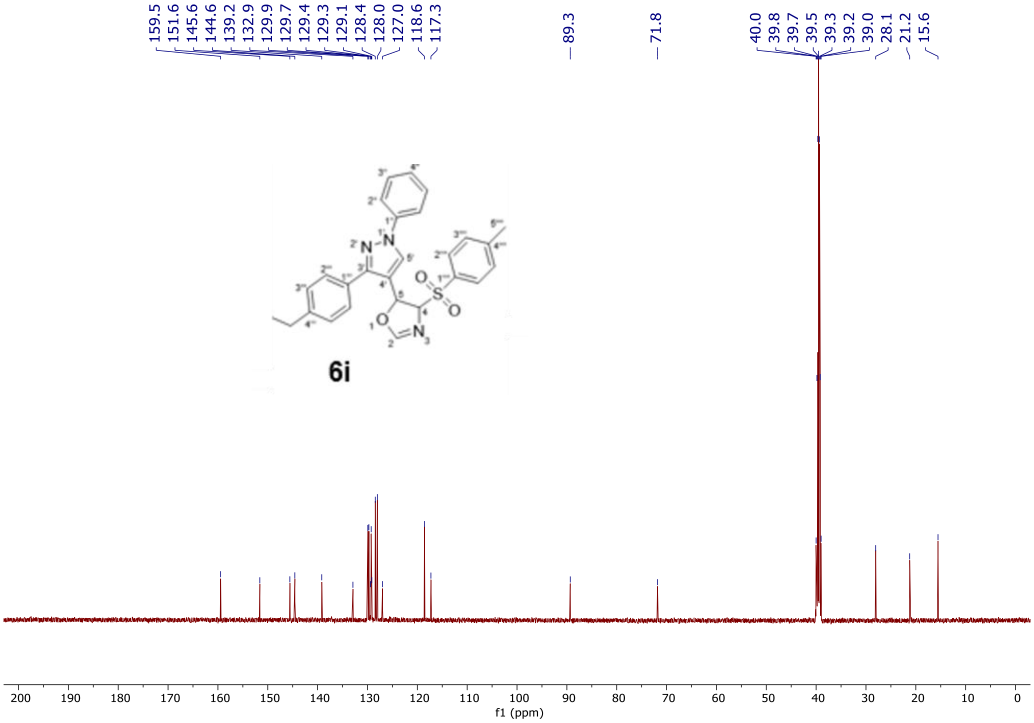

Figure S222. ^{13}C NMR (125 MHz, DMSO-d$_6$) of (4*S**, 5*S**)-5-(3-(4-ethylphenyl)-1-phenyl-1*H*-pyrazol-4-yl)-4-tosyl-4,5-dihydrooxazole **6i**.

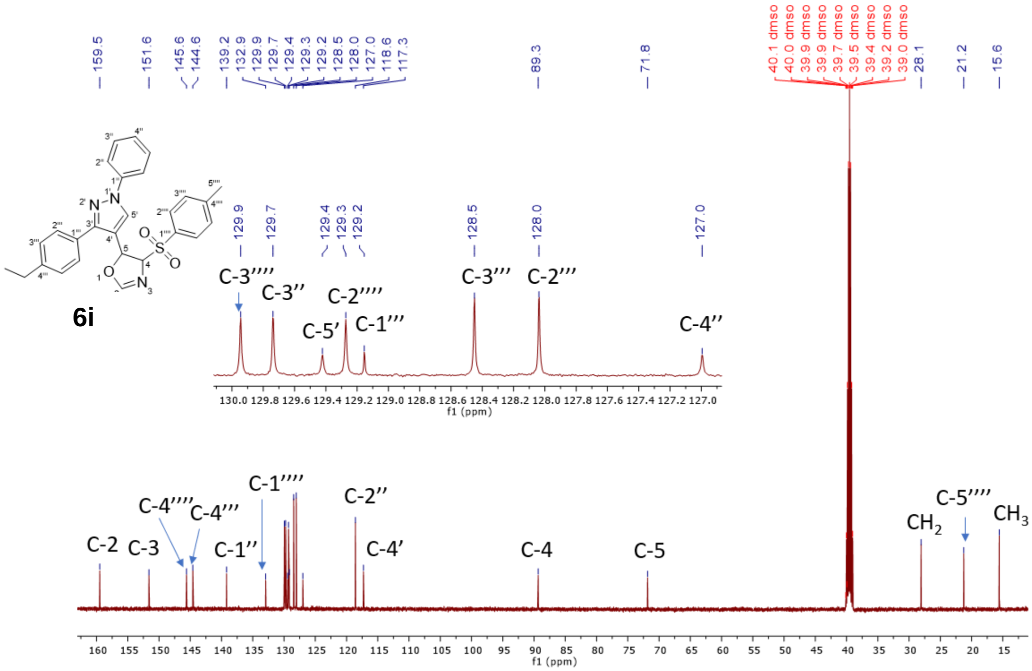

Figure S223. Expansion of ^{13}C NMR (125 MHz, DMSO-d$_6$) of (4*S**, 5*S**)-5-(3-(4-ethylphenyl)-1-phenyl-1*H*-pyrazol-4-yl)-4-tosyl-4,5-dihydrooxazole **6i**.

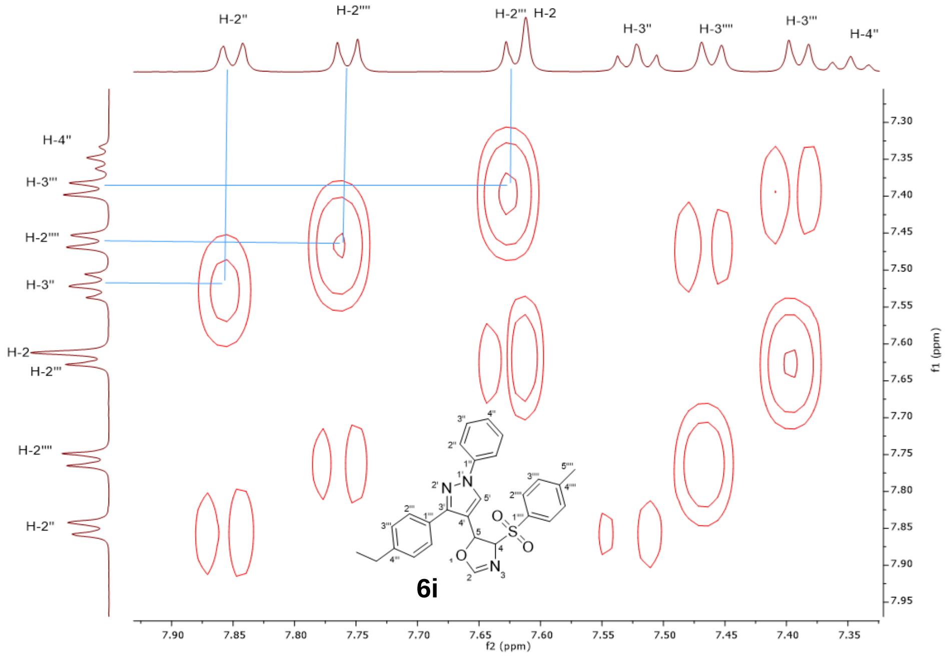

Figure S224. Expansion of COSY experiment of (4*S**, 5*S**)-5-(3-(4-ethylphenyl)-1-phenyl-1*H*-pyrazol-4-yl)-4-tosyl-4,5-dihydrooxazole **6i**.

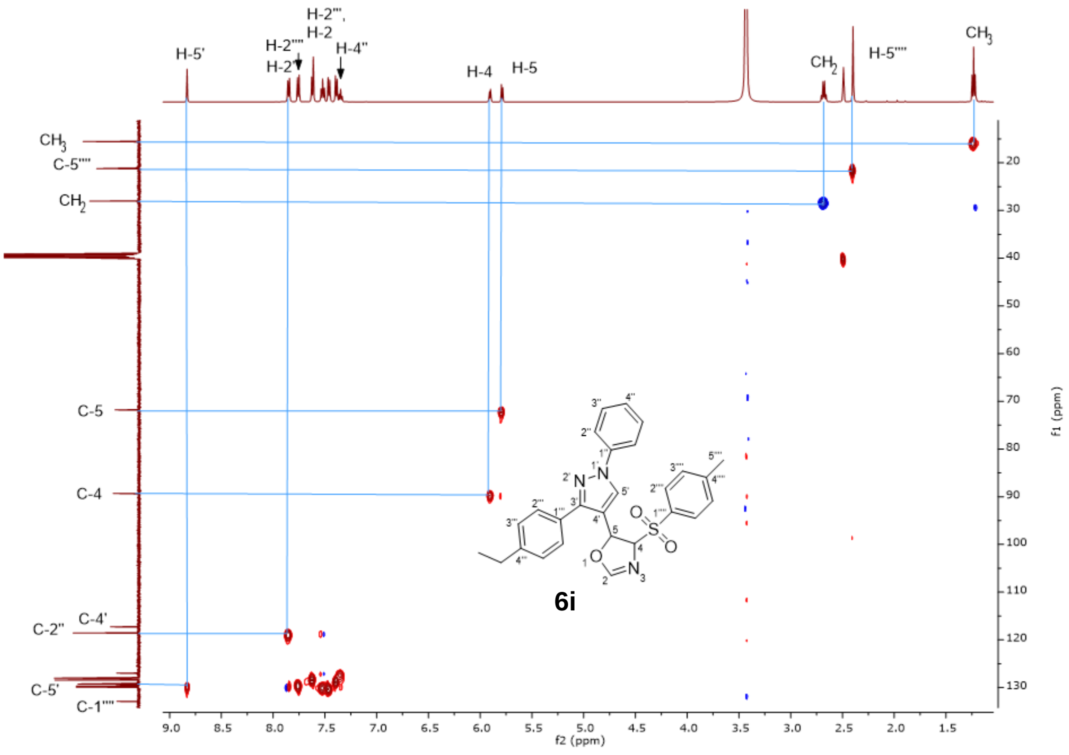

Figure S225. HSQC experiment of (4*S**, 5*S**)-5-(3-(4-ethylphenyl)-1-phenyl-1*H*-pyrazol-4-yl)-4-tosyl-4,5-dihydrooxazole **6i**.

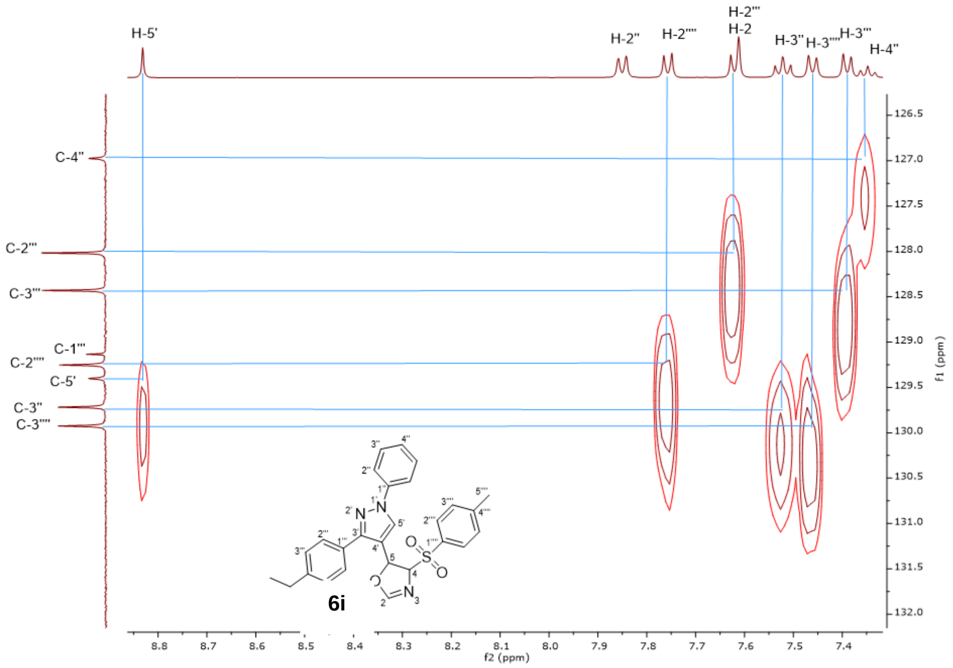

Figure S226. Expansion of HSQC experiment of (4*S**, 5*S**)-5-(3-(4-ethylphenyl)-1-phenyl-1*H*-pyrazol-4-yl)-4-tosyl-4,5-dihydrooxazole **6i**.

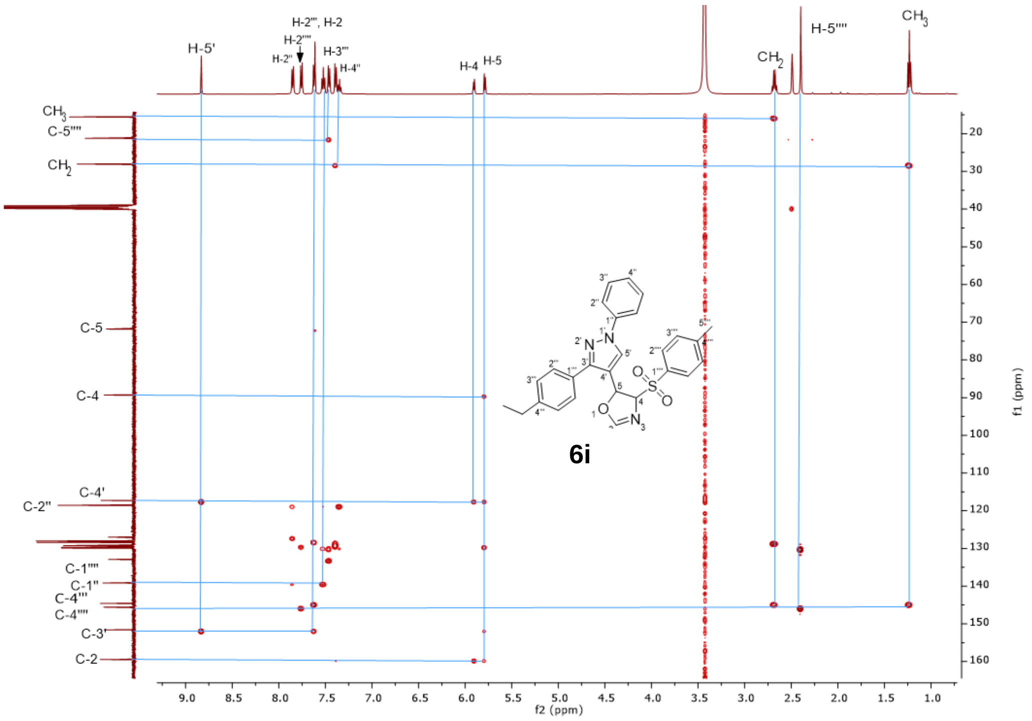

Figure S227. HMBC experiment of (4*S**, 5*S**)-5-(3-(4-ethylphenyl)-1-phenyl-1*H*-pyrazol-4-yl)-4-tosyl-4,5-dihydrooxazole **6i**.

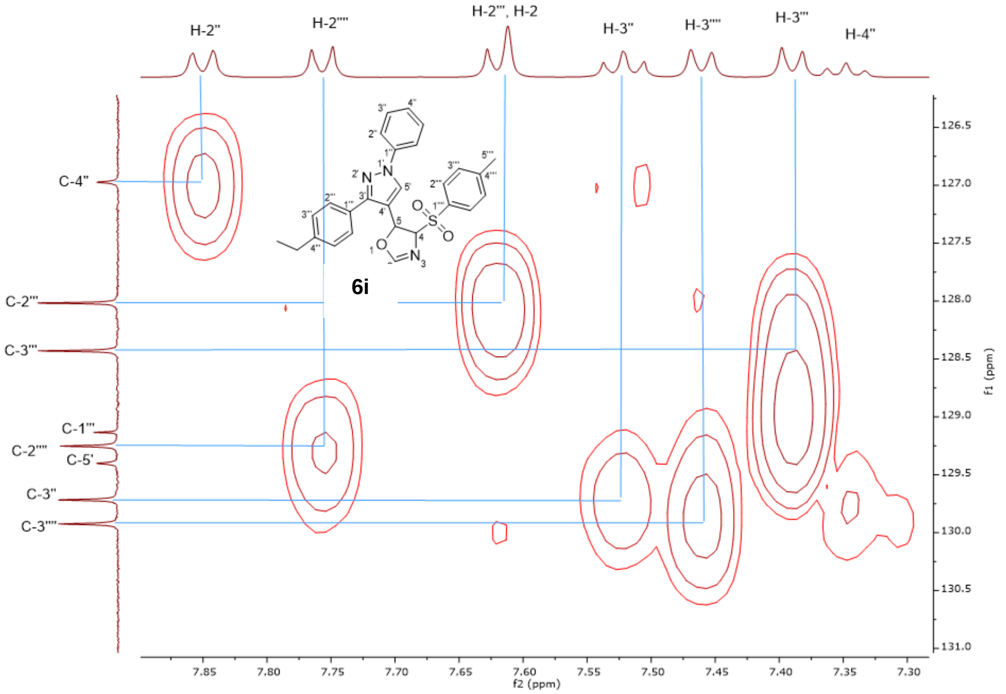

Figure S228. Expansion of HMBC experiment of (4*S**, 5*S**)-5-(3-(4-ethylphenyl)-1-phenyl-1*H*-pyrazol-4-yl)-4-tosyl-4,5-dihydrooxazole **6i**.

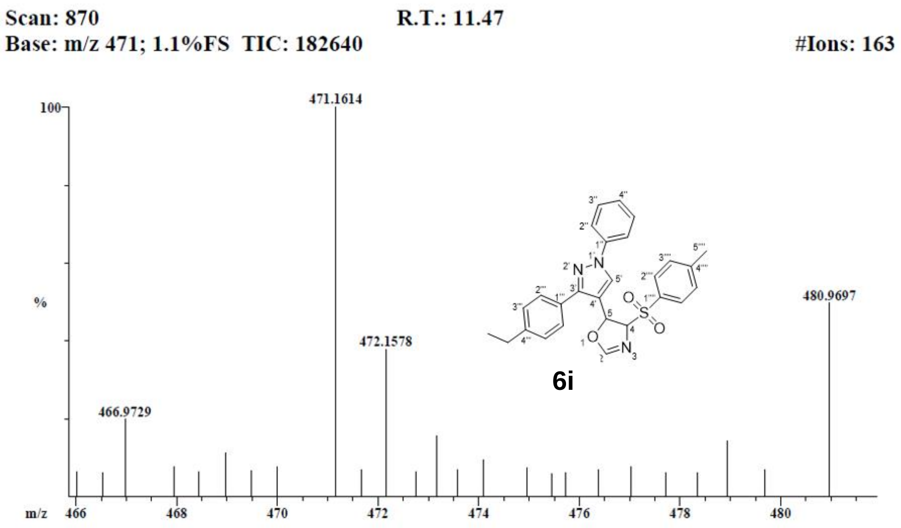

Selected Isotopes : $H_{0-25}C_{0-27}N_{0-3}O_{0-3}S_{0-1}$ Error Limit : 5 ppm Unsaturation Limits : 0 to 50

Measured Mass	% Base	Formula	Calculated Mass	Error	Unsaturation
471.1614	100.0%	$C_{27}H_{25}N_3O_3S$	471.1617	-0.6	17.0

Figure S229. HRMS of (4*S**, 5*S**)-5-(3-(4-ethylphenyl)-1-phenyl-1*H*-pyrazol-4-yl)-4-tosyl-4,5-dihydrooxazole **6i**.

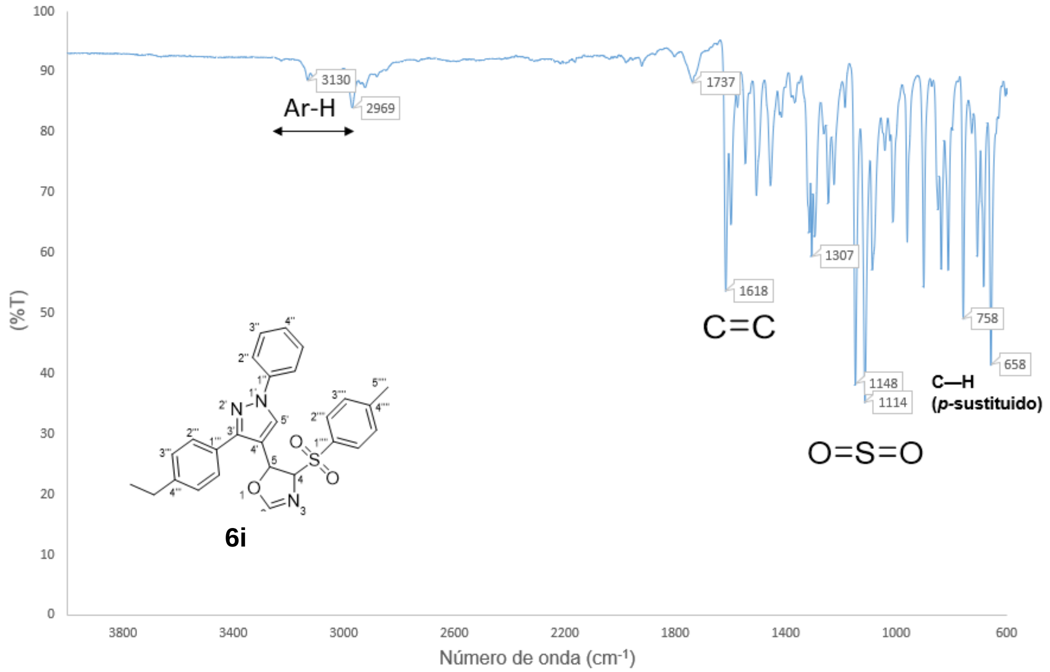

Figure S230. FT-IR of (4*S**, 5*S**)-5-(3-(4-ethylphenyl)-1-phenyl-1*H*-pyrazol-4-yl)-4-tosyl-4,5-dihydrooxazole **6i**.

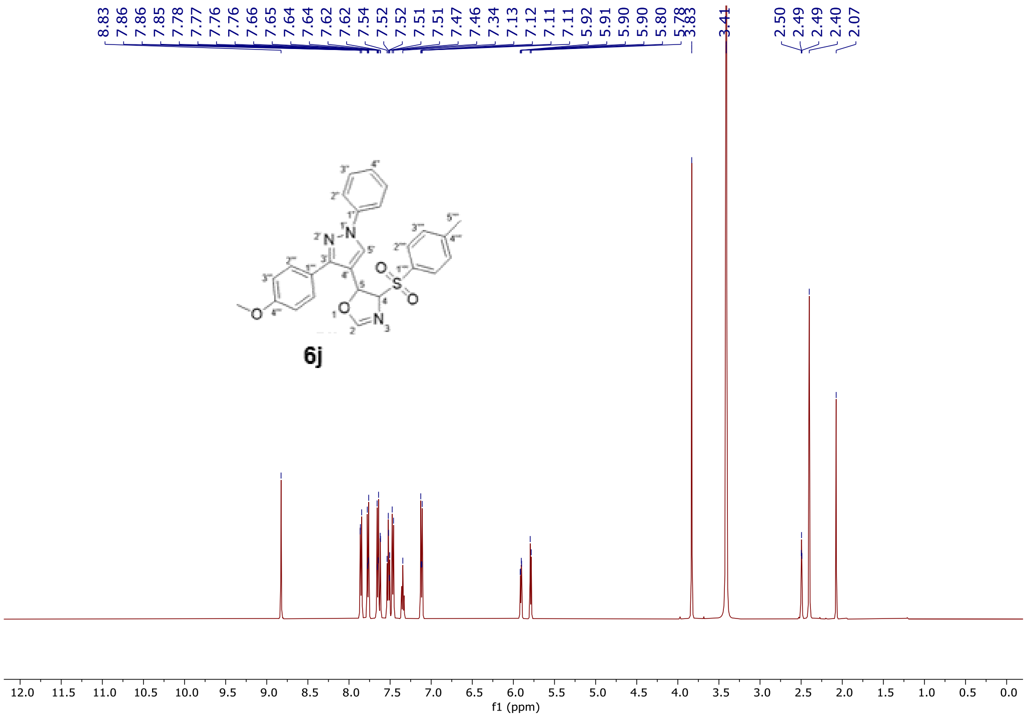

Figure S231. ^{1}H NMR (500 MHz, DMSO-d$_6$) of (4*S**, 5*S**)-5-(3-(4-methoxyphenyl)-1-phenyl-1*H*-pyrazol-4-yl)-4-tosyl-4,5-dihydrooxazole **6j**.

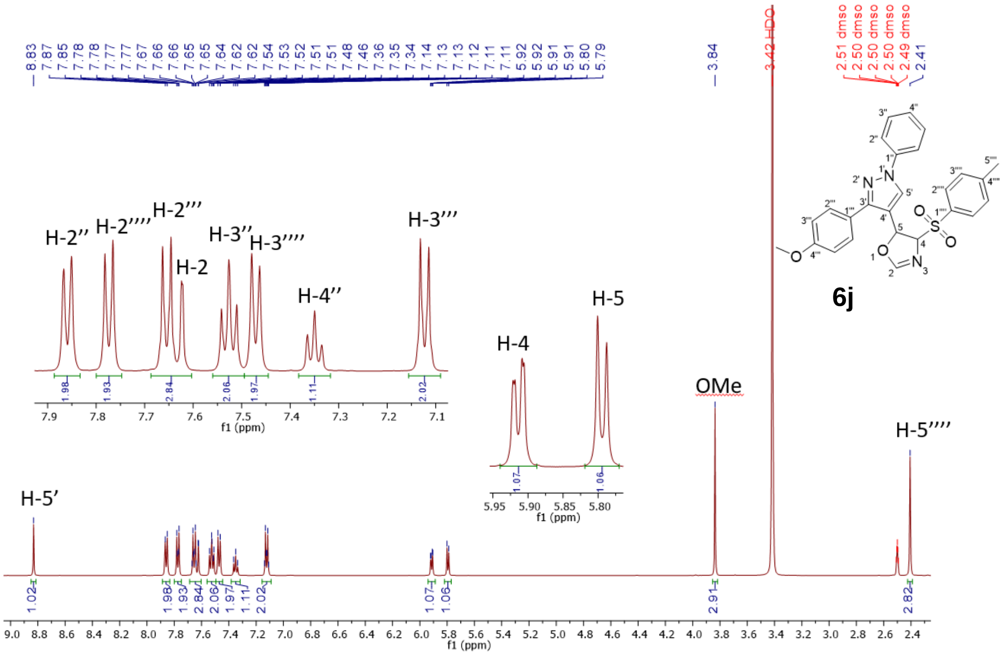

Figure S232. Expansion of ^{1}H NMR (500 MHz, DMSO-d$_6$) of (4*S**, 5*S**)-5-(3-(4-methoxyphenyl)-1-phenyl-1*H*-pyrazol-4-yl)-4-tosyl-4,5-dihydrooxazole **6j**.

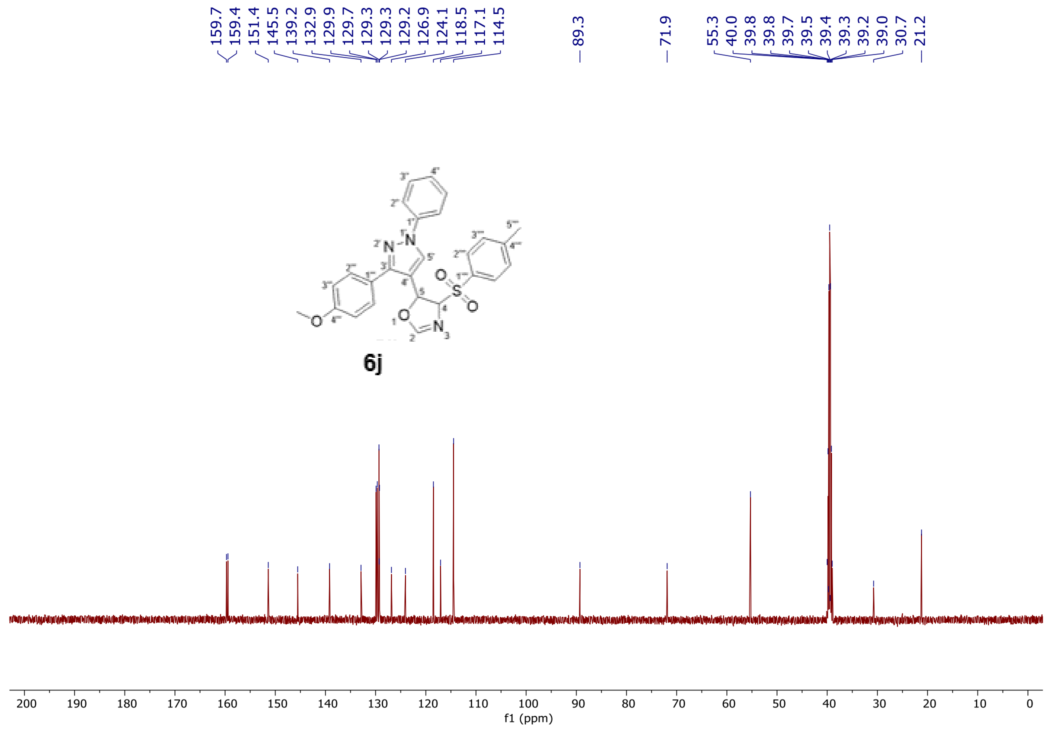

Figure S233. ^{13}C NMR (125 MHz, DMSO-d_6) of (4*S**, 5*S**)-5-(3-(4-methoxyphenyl)-1-phenyl-1*H*-pyrazol-4-yl)-4-tosyl-4,5-dihydrooxazole **6j**.

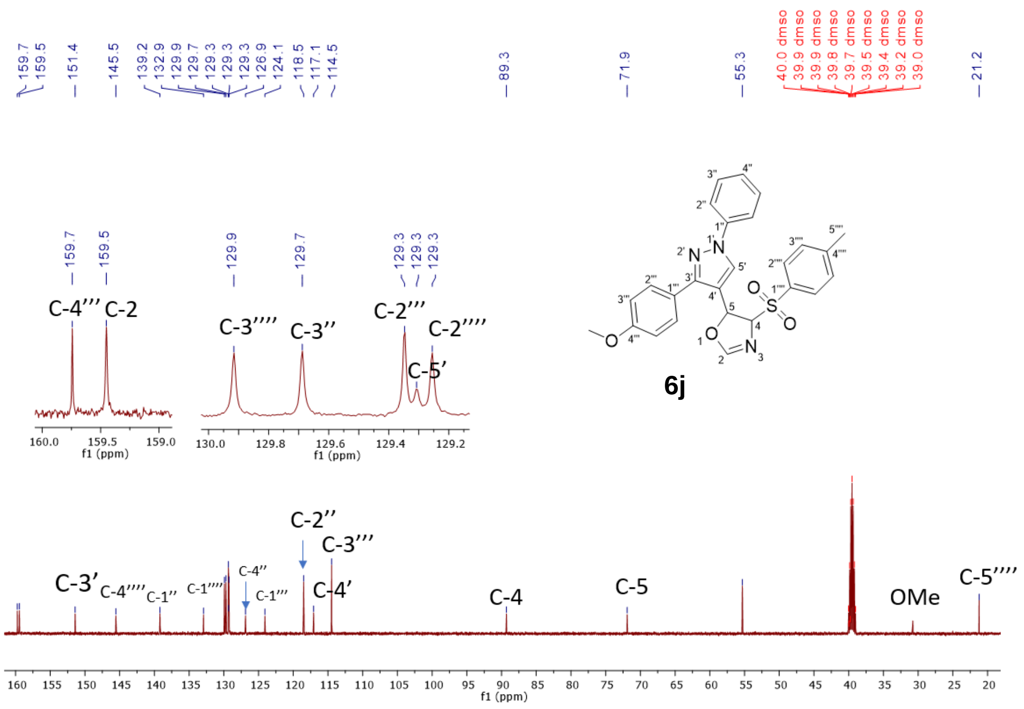

Figure S234. Expansion of ^{13}C NMR (125 MHz, DMSO-d_6) of (4*S**, 5*S**)-5-(3-(4-methoxyphenyl)-1-phenyl-1*H*-pyrazol-4-yl)-4-tosyl-4,5-dihydrooxazole **6j**.

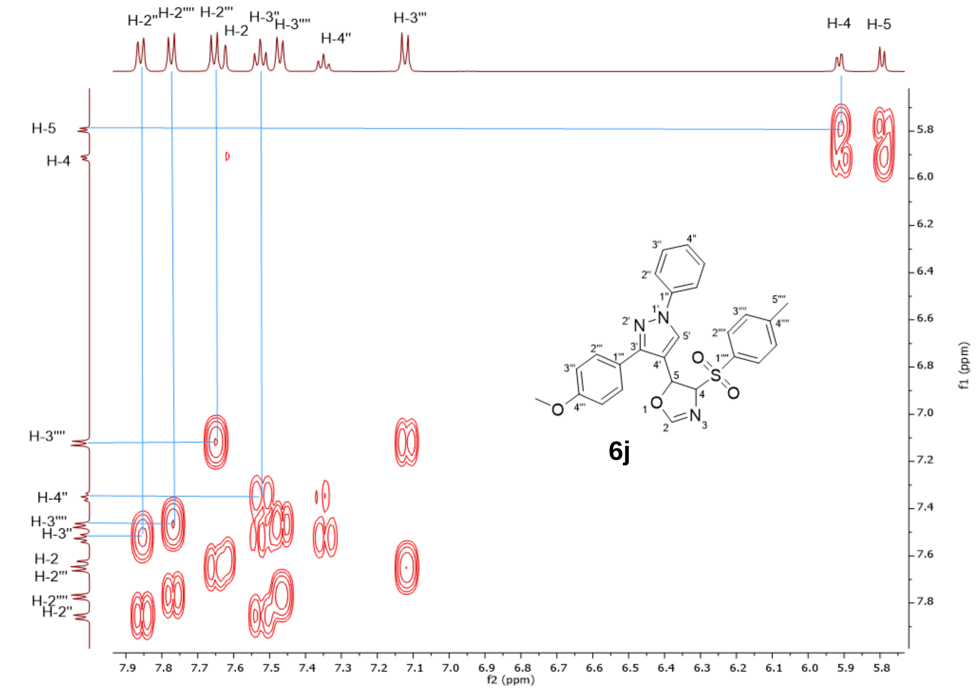

Figure S235. COSY experiment of (4*S**, 5*S**)-5-(3-(4-methoxyphenyl)-1-phenyl-1*H*-pyrazol-4-yl)-4-tosyl-4,5-dihydrooxazole **6j**.

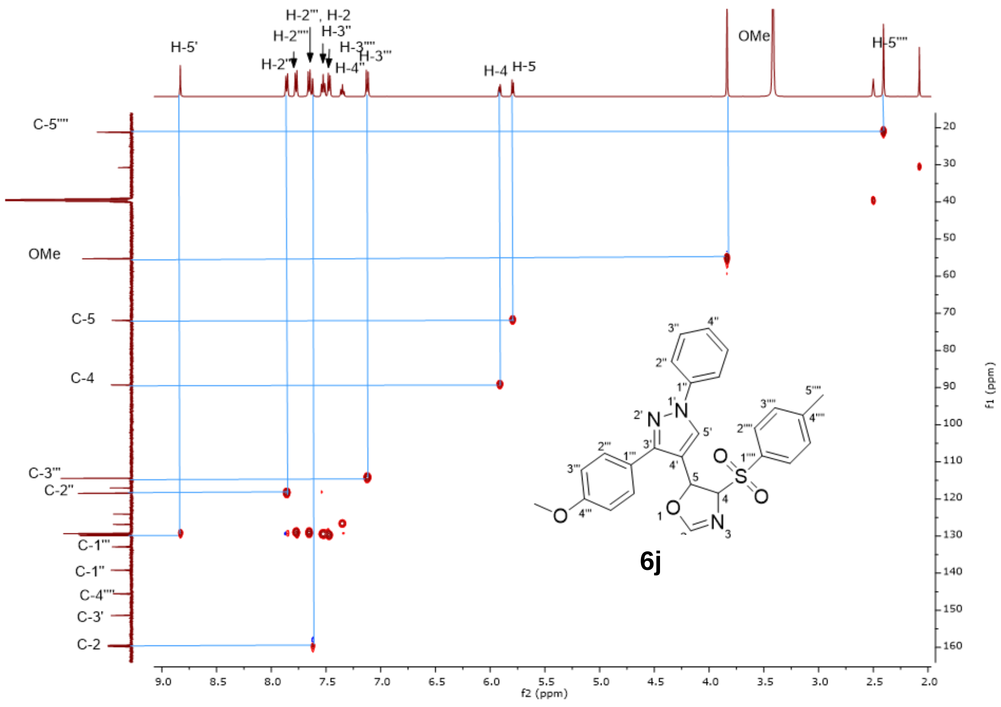

Figure S236. HSQC experiment of (4*S**, 5*S**)-5-(3-(4-methoxyphenyl)-1-phenyl-1*H*-pyrazol-4-yl)-4-tosyl-4,5-dihydrooxazole **6j**.

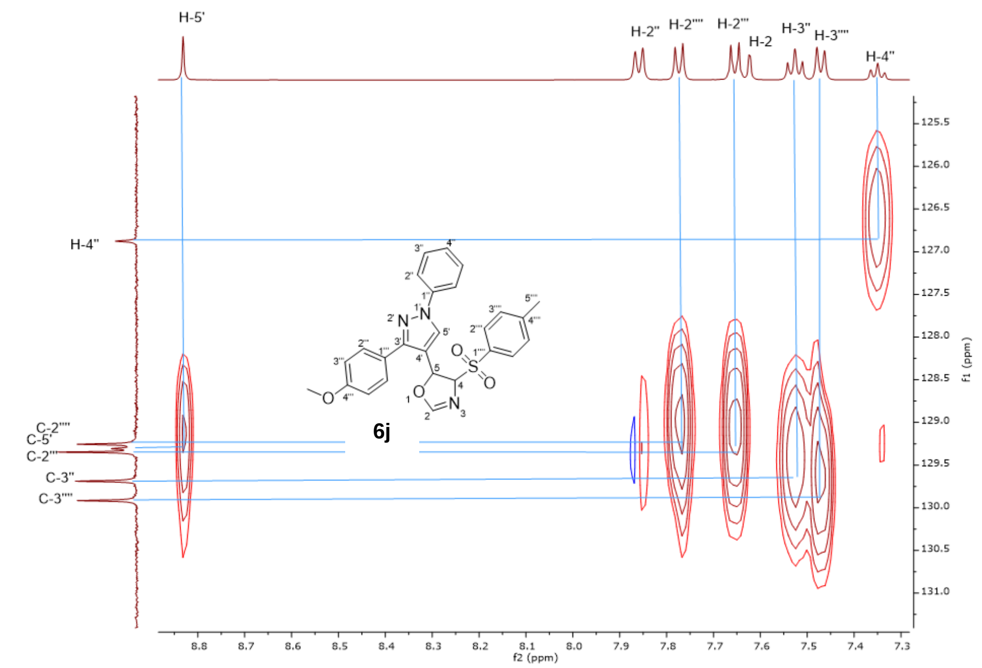

Figure S237. Expansion of HSQC experiment of (4*S**, 5*S**)-5-(3-(4-methoxyphenyl)-1-phenyl-1*H*-pyrazol-4-yl)-4-tosyl-4,5-dihydrooxazole **6j**.

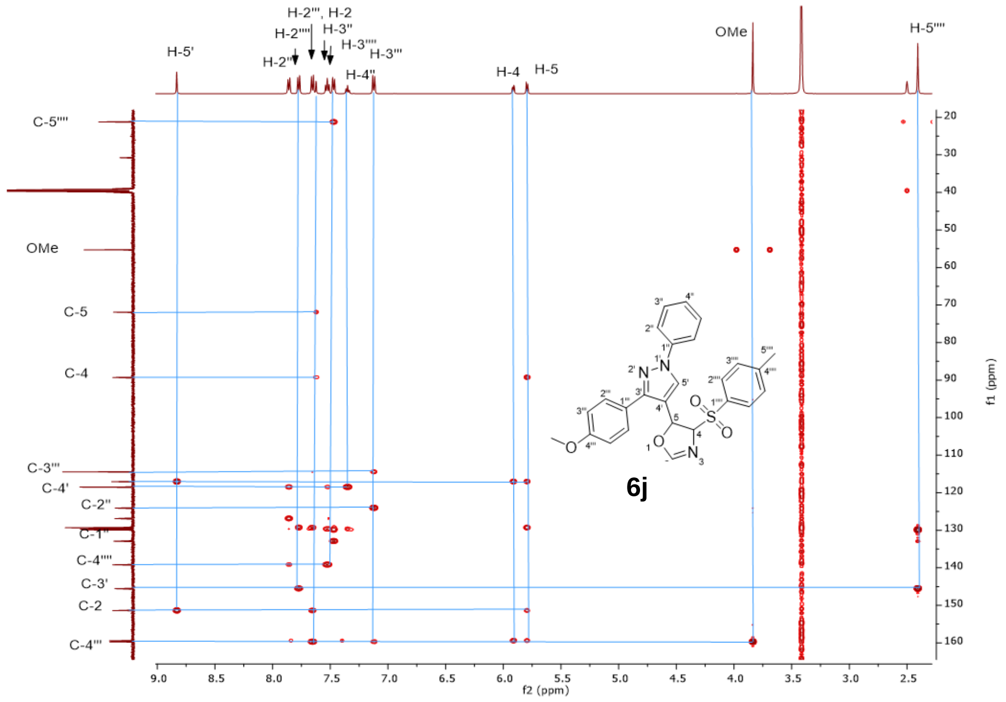

Figure S238. HMBC experiment of (4*S**, 5*S**)-5-(3-(4-methoxyphenyl)-1-phenyl-1*H*-pyrazol-4-yl)-4-tosyl-4,5-dihydrooxazole **6j**.

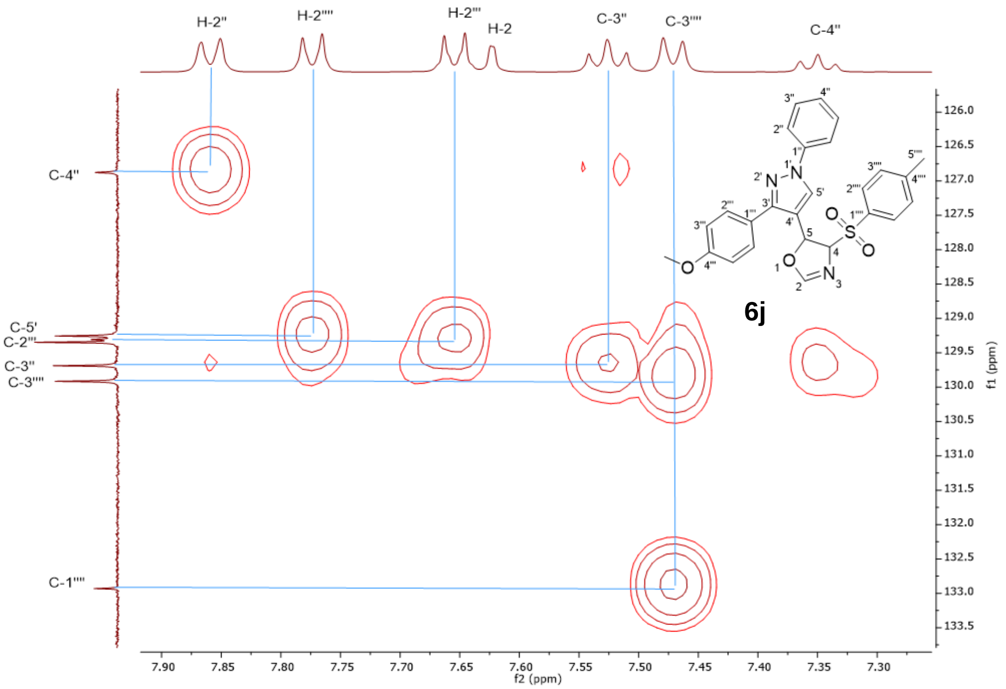

Figure S239. Expansion of HMBC experiment of (4*S**, 5*S**)-5-(3-(4-methoxyphenyl)-1-phenyl-1*H*-pyrazol-4-yl)-4-tosyl-4,5-dihydrooxazole **6j**.

Scan: 443 **R.T.: 7.32**
Base: m/z 473; 5.5%FS TIC: 161616 **#Ions: 92**

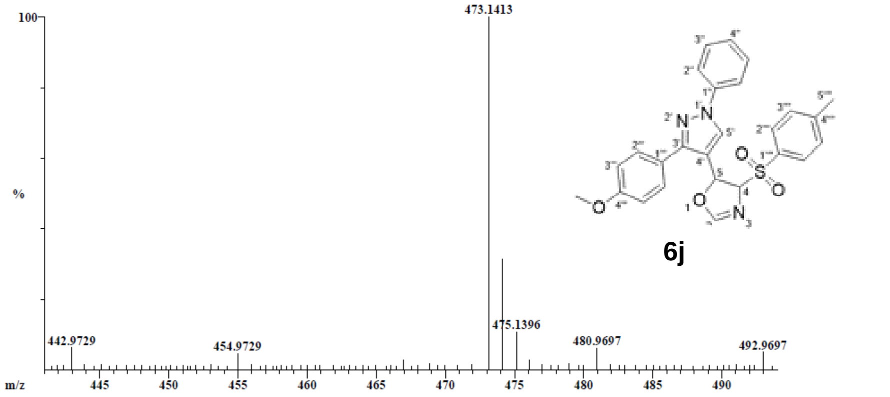

Selected Isotopes : $H_{0-23}C_{0-26}N_{0-3}O_{0-4}S_{0-1}$ Error Limit : 5 ppm Unsaturation Limits : 0 to 50

Measured Mass	% Base	Formula	Calculated Mass	Error	Unsaturation
473.1413	100.0%	$C_{26}H_{23}N_3O_4S$	473.1409	0.8	17.0

Figure S240. HRMS of (4*S**, 5*S**)-5-(3-(4-methoxyphenyl)-1-phenyl-1*H*-pyrazol-4-yl)-4-tosyl-4,5-dihydrooxazole **6j**.

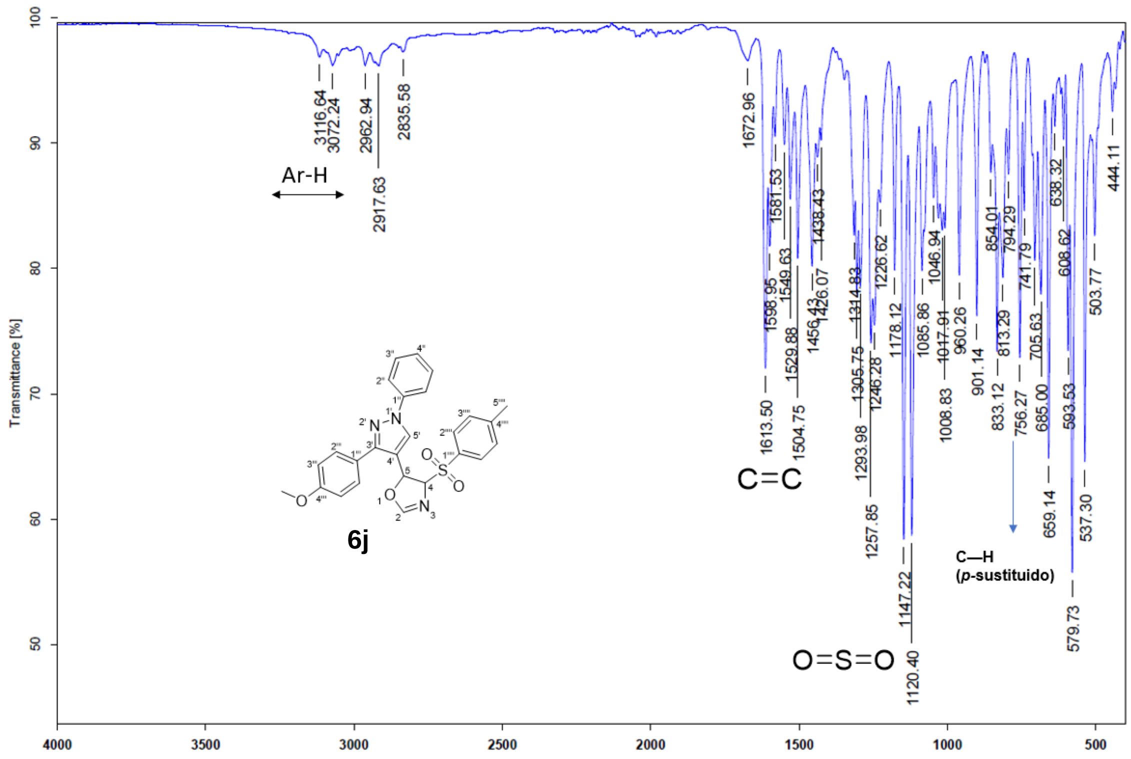

Figure S241. FT-IR of (4*S**, 5*S**)-5-(3-(4-methoxyphenyl)-1-phenyl-1*H*-pyrazol-4-yl)-4-tosyl-4,5-dihydrooxazole **6j**.

Reaction mechanisms

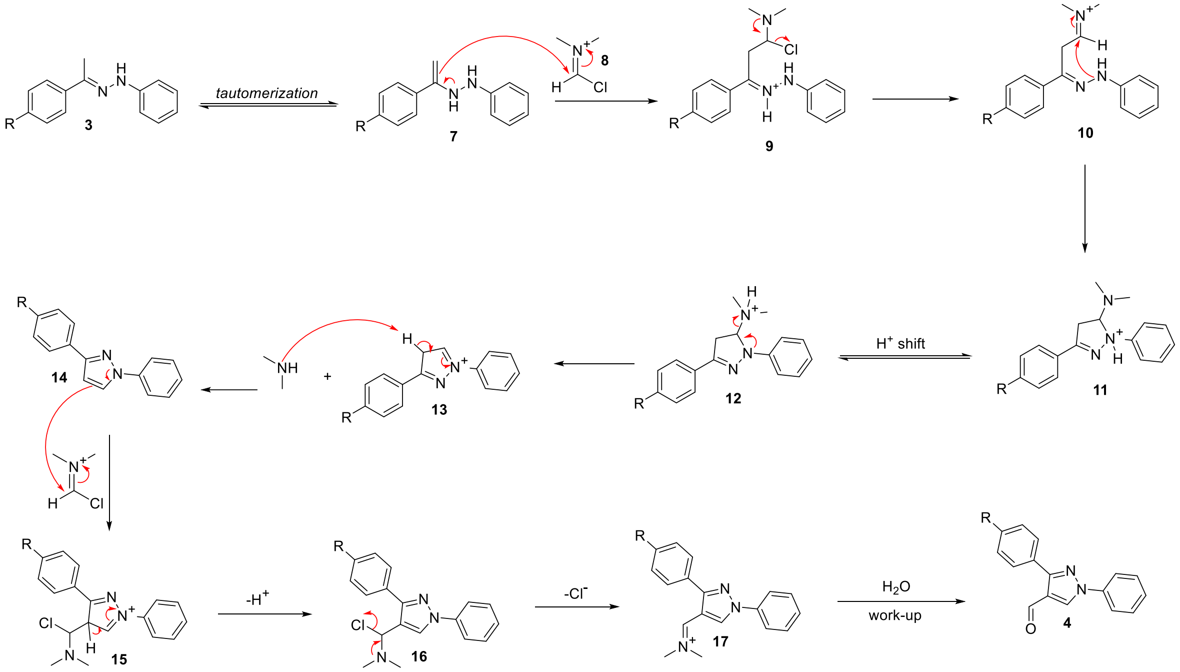

Scheme S1. Mechanistic proposal for the synthesis of 1,3-diphenyl-1*H*-pyrazole-4-carbaldehydes **4a-j**.

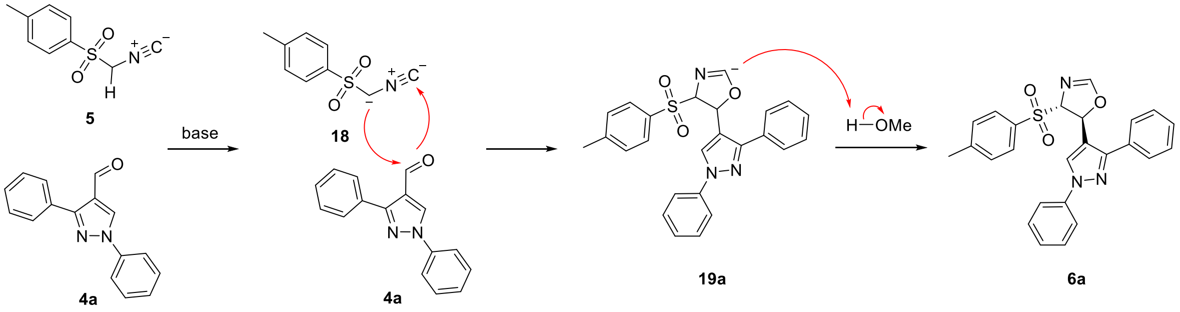

Scheme S2. Proposed mechanism of formation of (4*S**, 5*S**)-5-(1,3-diphenyl-1*H*-pyrazol-4-yl)-4-tosyl-4,5-dihydrooxazole **6a** from 1,3-diphenyl-1*H*-pyrazole-4-carbaldehyde **4a** and TosMIC **5**.

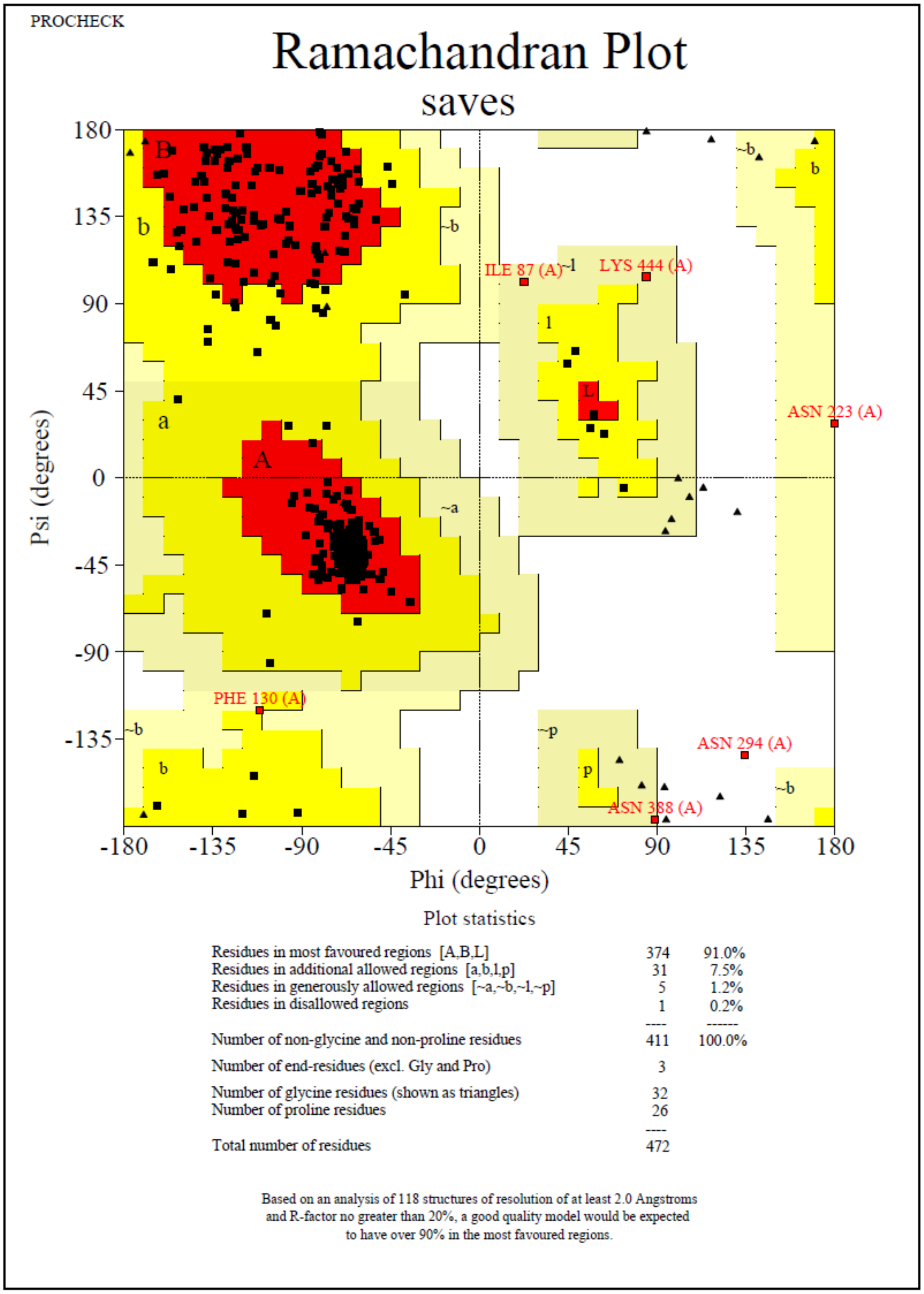

saves_01.ps

Figure S242. Ramachandran plot of the *C. auris* CYP51 model.

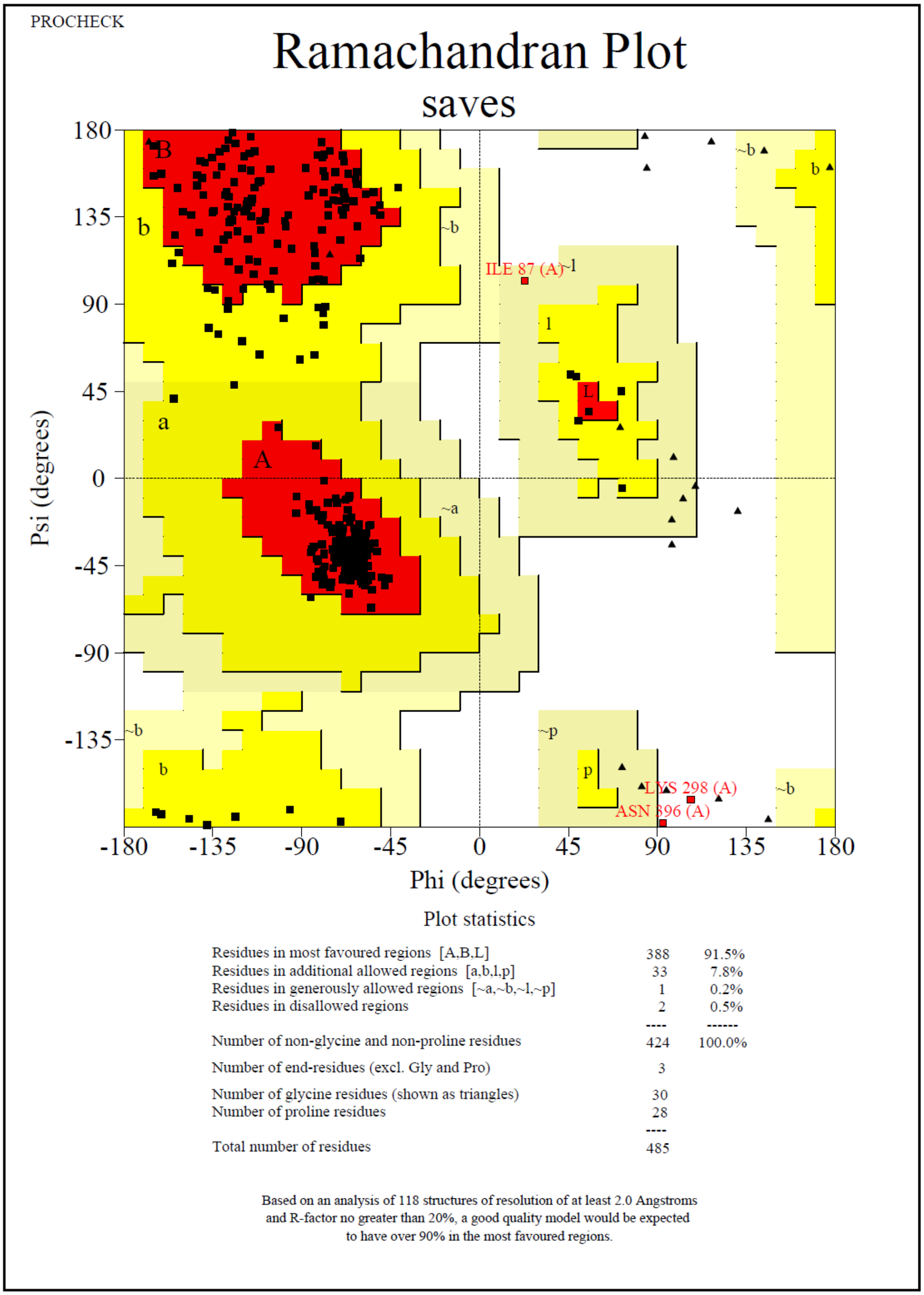

saves_01.ps

Figure S243. Ramachandran plot of the *C. dubliniensis* CYP51 model.

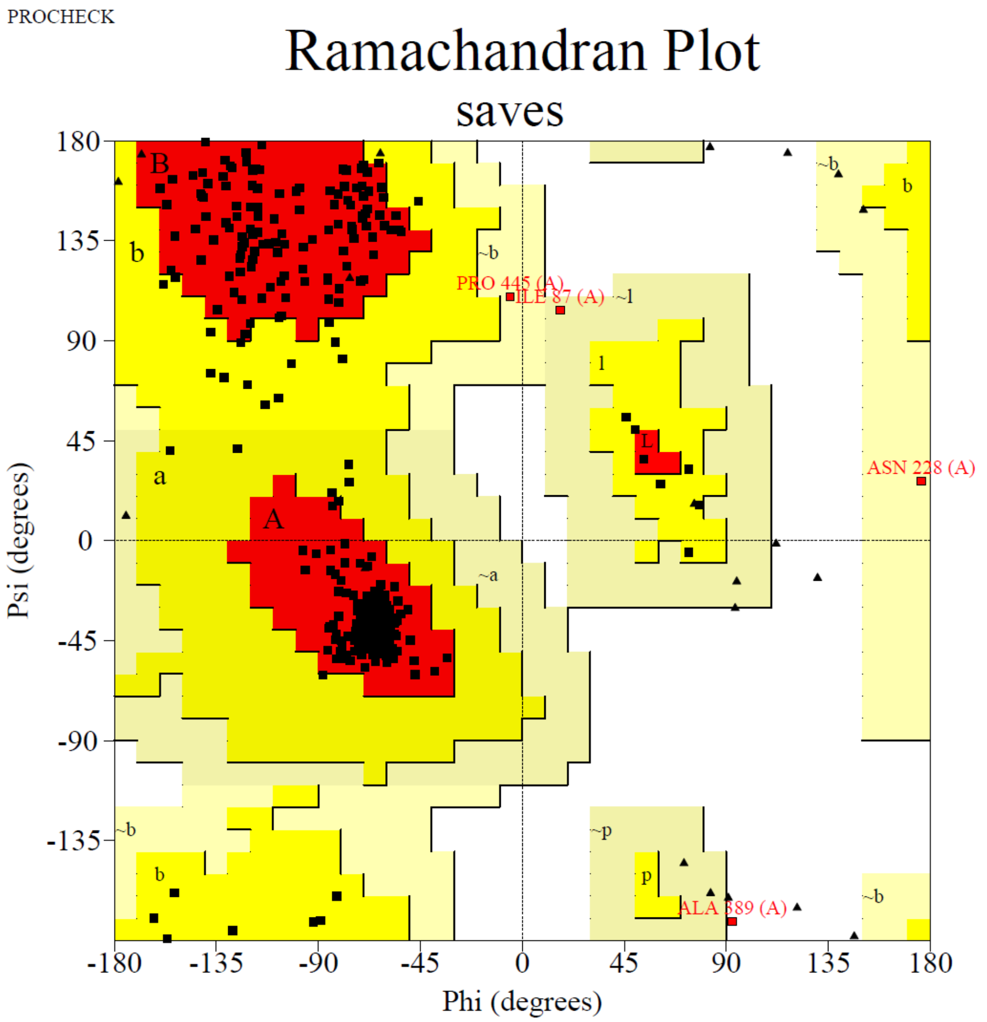

Plot statistics

Residues in most favoured regions [A,B,L]	381	91.4%
Residues in additional allowed regions [a,b,l,p]	33	7.9%
Residues in generously allowed regions [~a,~b,~l,~p]	2	0.5%
Residues in disallowed regions	1	0.2%
	----	------
Number of non-glycine and non-proline residues	417	100.0%
Number of end-residues (excl. Gly and Pro)	2	
Number of glycine residues (shown as triangles)	31	
Number of proline residues	28	

Total number of residues	478	

Based on an analysis of 118 structures of resolution of at least 2.0 Angstroms and R-factor no greater than 20%, a good quality model would be expected to have over 90% in the most favoured regions.

saves_01.ps

Figure S244. Ramachandran plot of the *C. glabrata* CYP51 model.

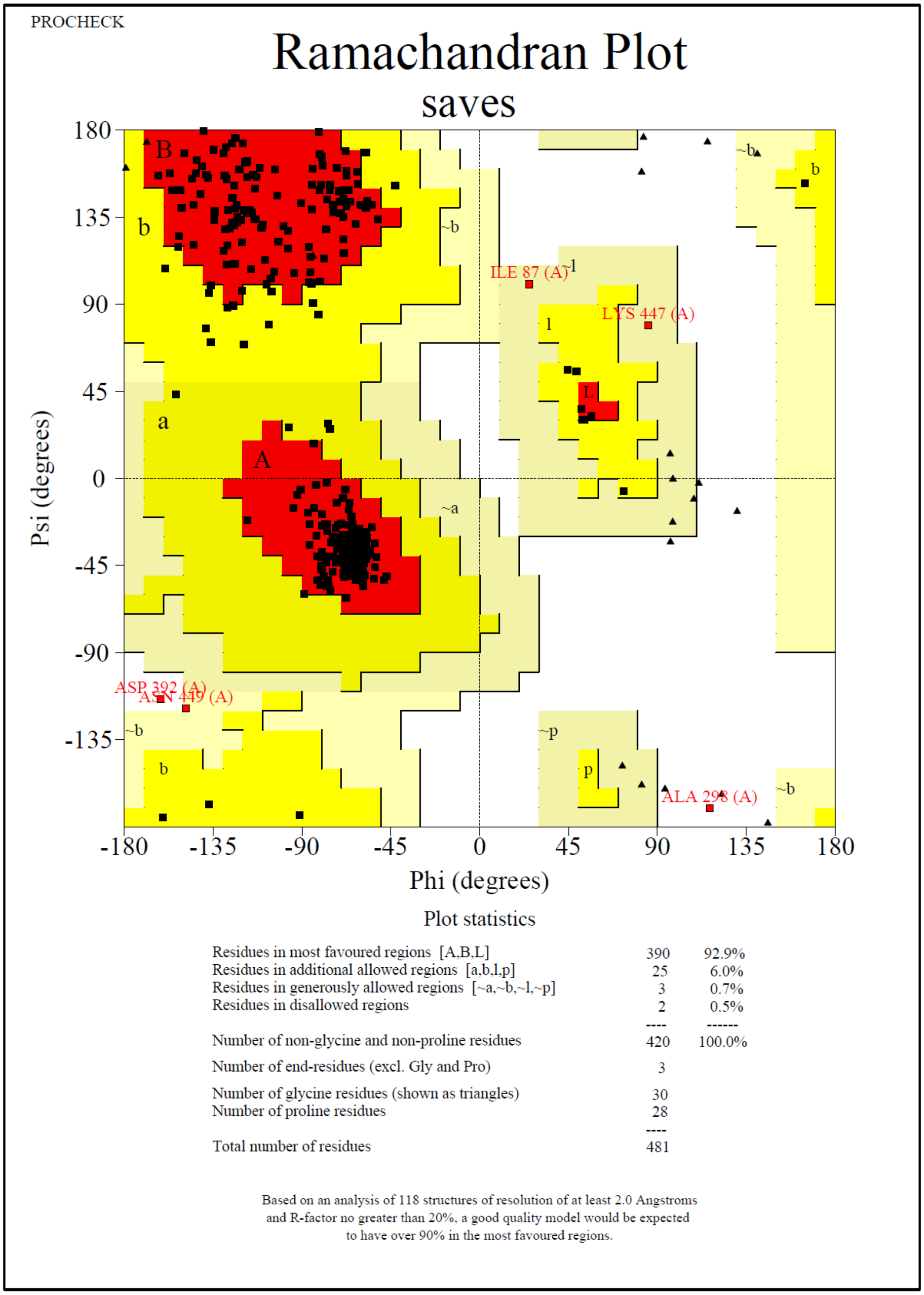

saves_01.ps

Figure S245. Ramachandran plot of the *C. haemulonii* CYP51 model.

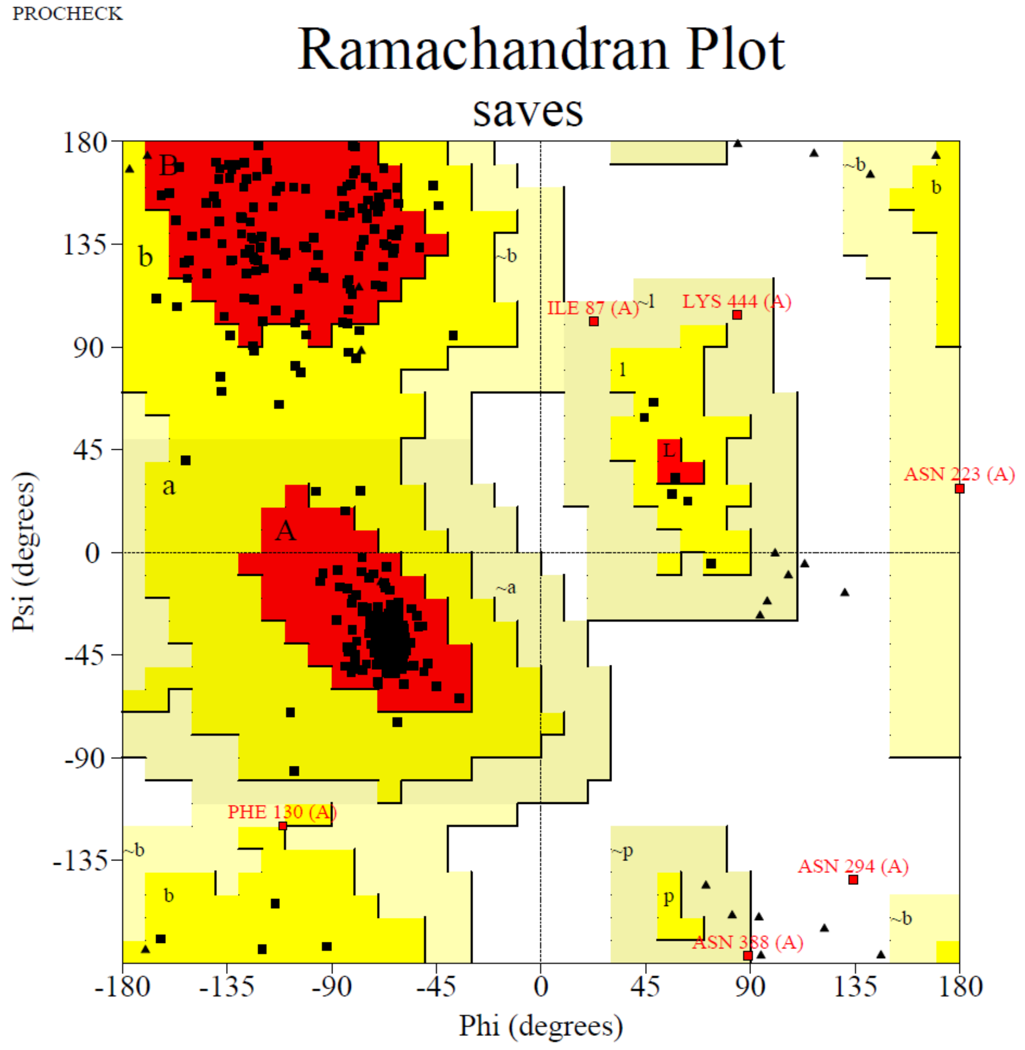

Plot statistics

Residues in most favoured regions [A,B,L]	374	91.0%
Residues in additional allowed regions [a,b,l,p]	31	7.5%
Residues in generously allowed regions [~a,~b,~l,~p]	5	1.2%
Residues in disallowed regions	1	0.2%
	----	------
Number of non-glycine and non-proline residues	411	100.0%
Number of end-residues (excl. Gly and Pro)	3	
Number of glycine residues (shown as triangles)	32	
Number of proline residues	26	

Total number of residues	472	

Based on an analysis of 118 structures of resolution of at least 2.0 Angstroms and R-factor no greater than 20%, a good quality model would be expected to have over 90% in the most favoured regions.

saves_01.ps

Figure S246. Ramachandran plot of the *C. krusei* CYP51 model.

Fluconazole 20

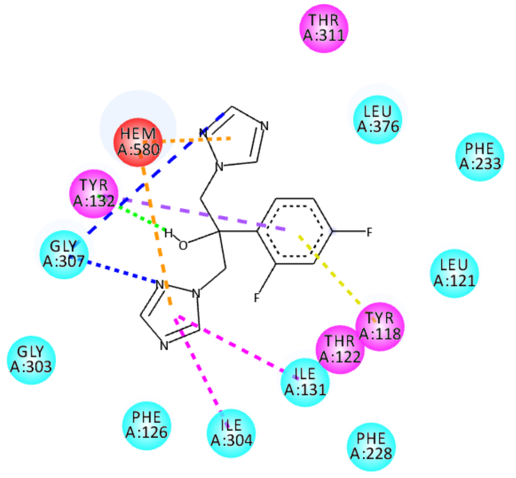

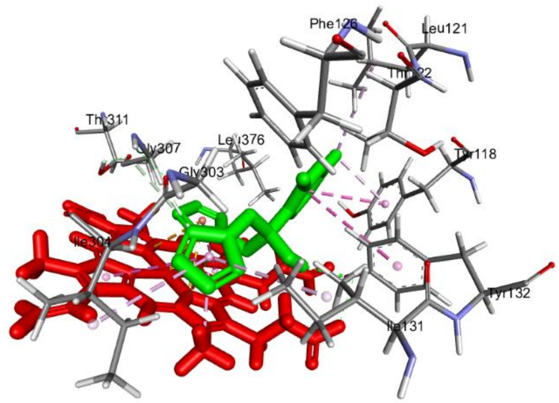

4a

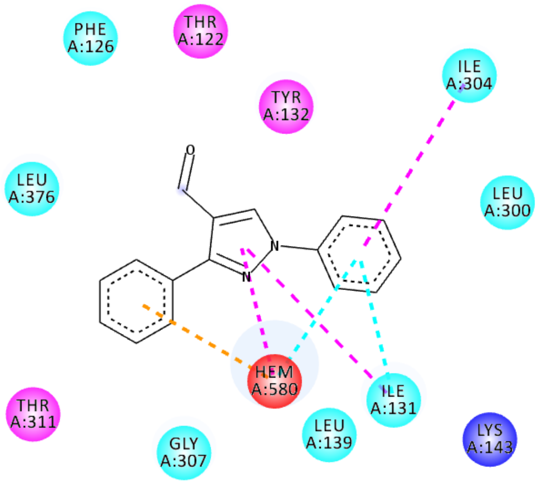

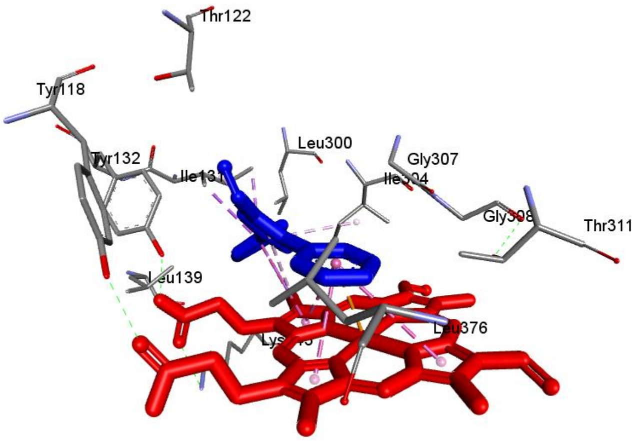

4b

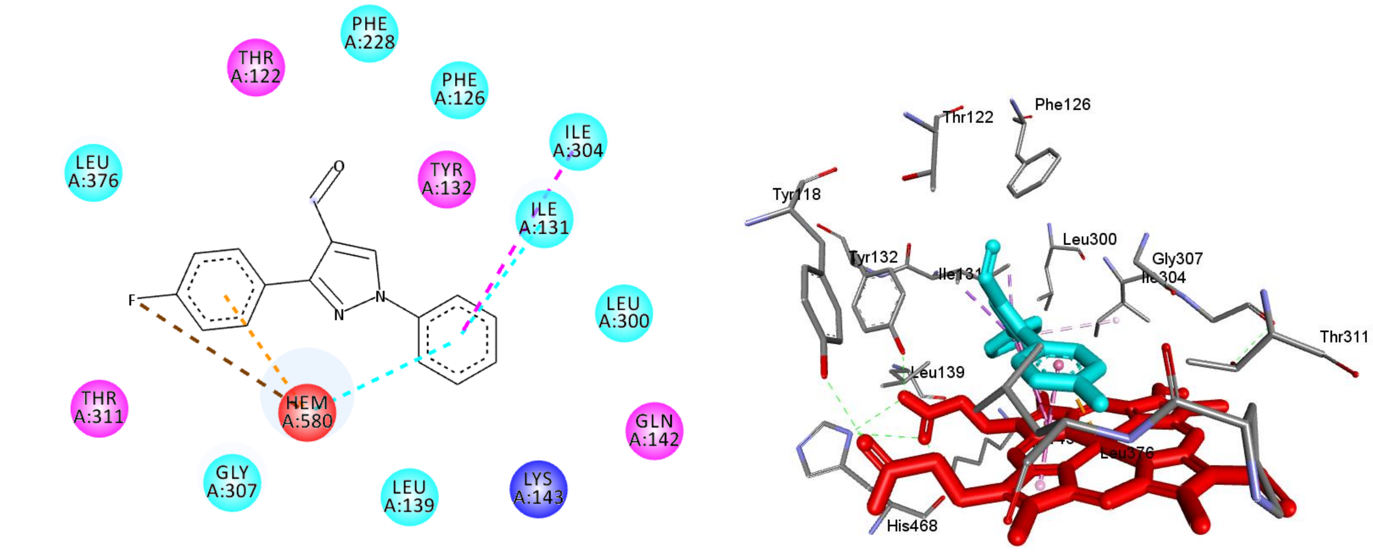
PHE A:228
THR A:122
PHE A:126
ILE A:304
LEU A:376
TYR A:132
ILE A:131
F
N
N
LEU A:300
THR A:311
HEM A:580
GLN A:142
GLY A:307
LEU A:139
LYS A:143
Phe126
Thr122
Tyr118
Leu300
Tyr132
Gly307
Ile304
Thr311
Leu139
Leu376
His468

4c

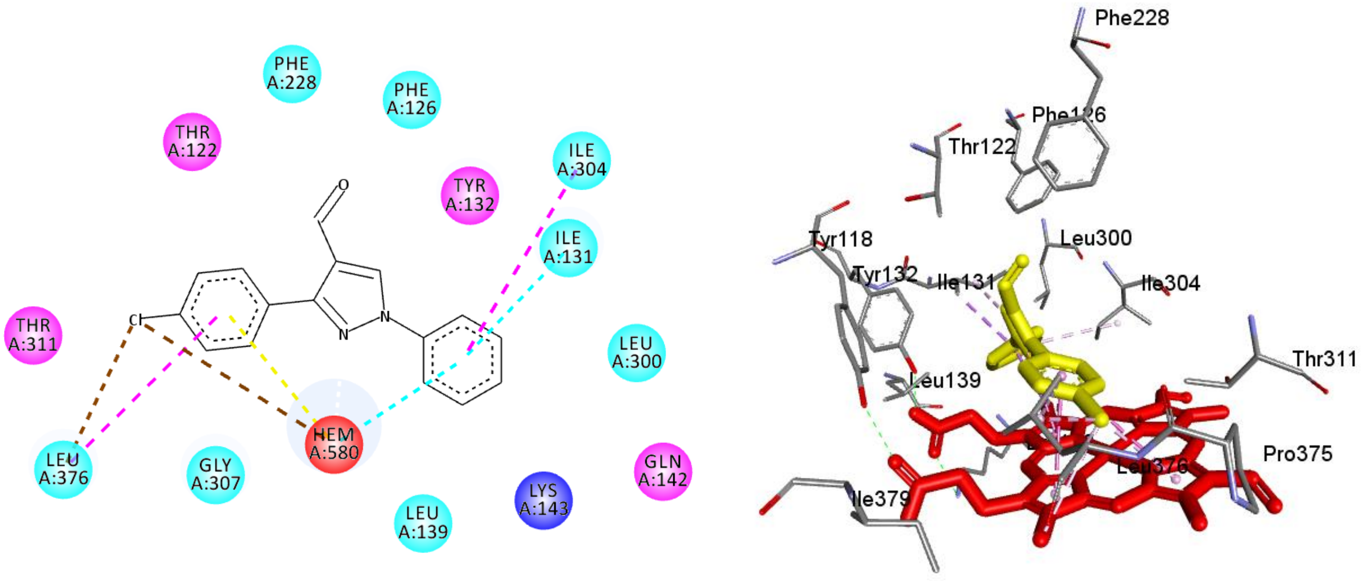
PHE A:228
PHE A:126
THR A:122
ILE A:304
TYR A:132
ILE A:131
Cl
N
N
THR A:311
LEU A:300
HEM A:580
LEU A:376
GLY A:307
GLN A:142
LYS A:143
LEU A:139
Phe228
Thr122
Tyr118
Leu300
Tyr132
Ile131
Ile304
Thr311
Leu139
Pro375
Ile379

4d

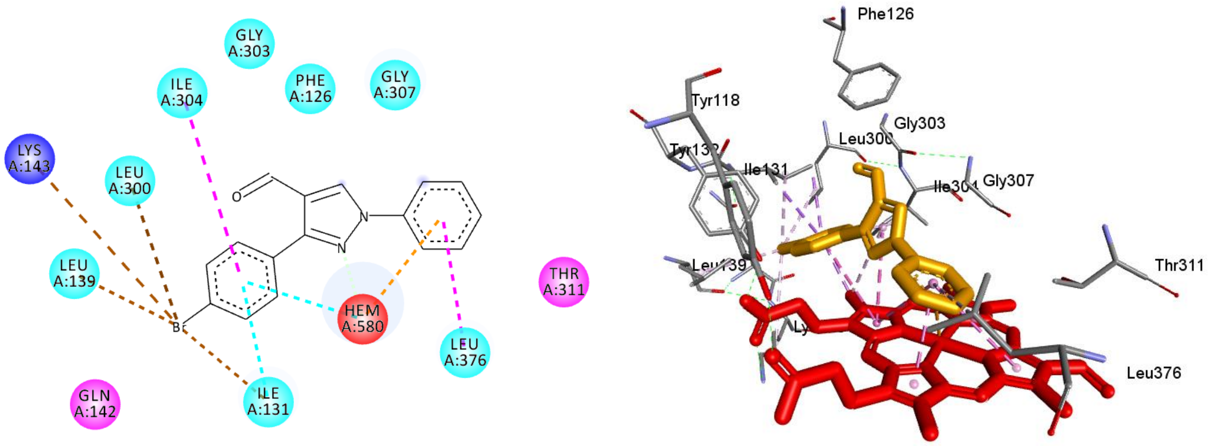
GLY A:303
ILE A:304
PHE A:126
GLY A:307
LYS A:143
LEU A:300
LEU A:139
HEM A:580
THR A:311
LEU A:376
GLN A:142
ILE A:131
O
N
Br
Phe126
Tyr118
Gly303
Leu300
Ile131
Gly307
Leu139
Thr311
Leu376

4e

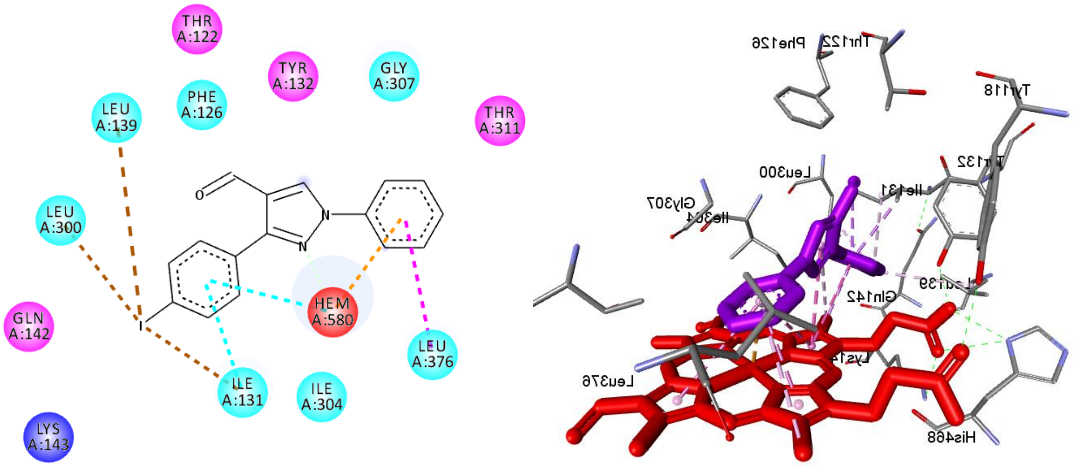
THR A:122
TYR A:132
GLY A:307
PHE A:126
LEU A:139
THR A:311
LEU A:300
GLN A:142
HEM A:580
LEU A:376
ILE A:131
ILE A:304
LYS A:143
O
N
I

4f

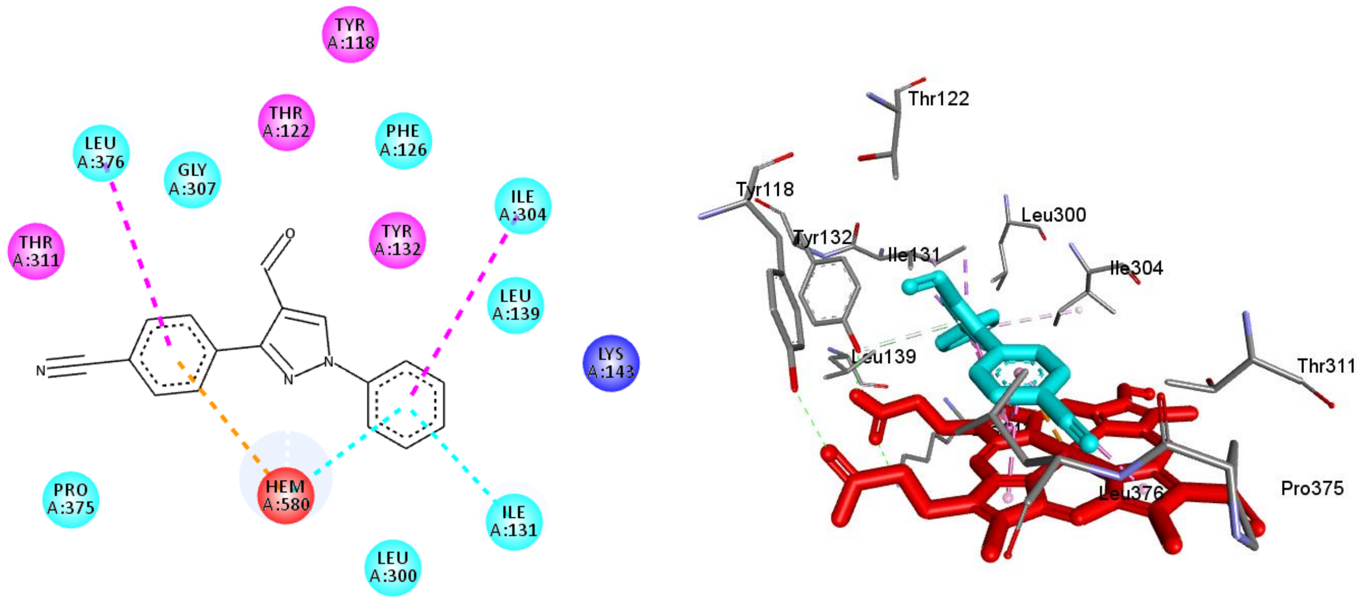
TYR A:118
THR A:122
PHE A:126
LEU A:376
GLY A:307
ILE A:304
THR A:311
TYR A:132
LEU A:139
LYS A:143
HEM A:580
PRO A:375
ILE A:131
LEU A:300
Thr122
Tyr118
Leu300
Tyr132
Ile131
Ile304
Thr311
Pro375

4g

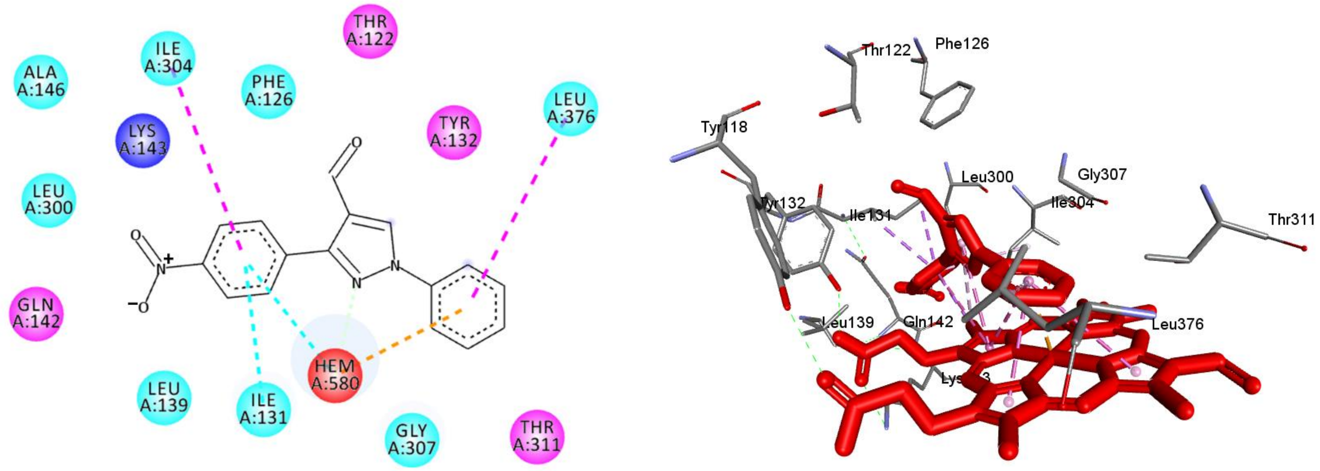
ALA A:146
ILE A:304
THR A:122
PHE A:126
LEU A:376
LYS A:143
TYR A:132
LEU A:300
GLN A:142
HEM A:580
LEU A:139
ILE A:131
GLY A:307
THR A:311
Thr122
Phe126
Leu300
Gly307
Ile304
Ile131
Thr311
Gln142
Leu376

4h

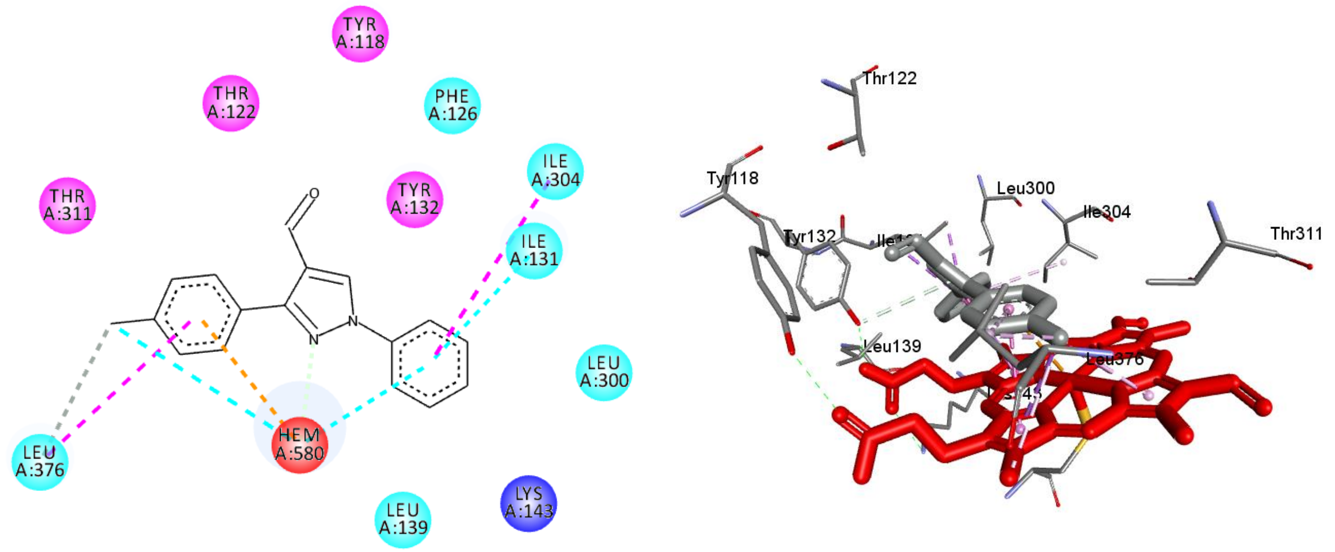

TYR A:118
THR A:122
PHE A:126
ILE A:304
THR A:311
TYR A:132
ILE A:131
LEU A:300
LEU A:376
HEM A:580
LEU A:139
LYS A:143
Thr122
Tyr118
Leu300
Ile304
Thr311
Tyr132
Leu139
Leu376

4i

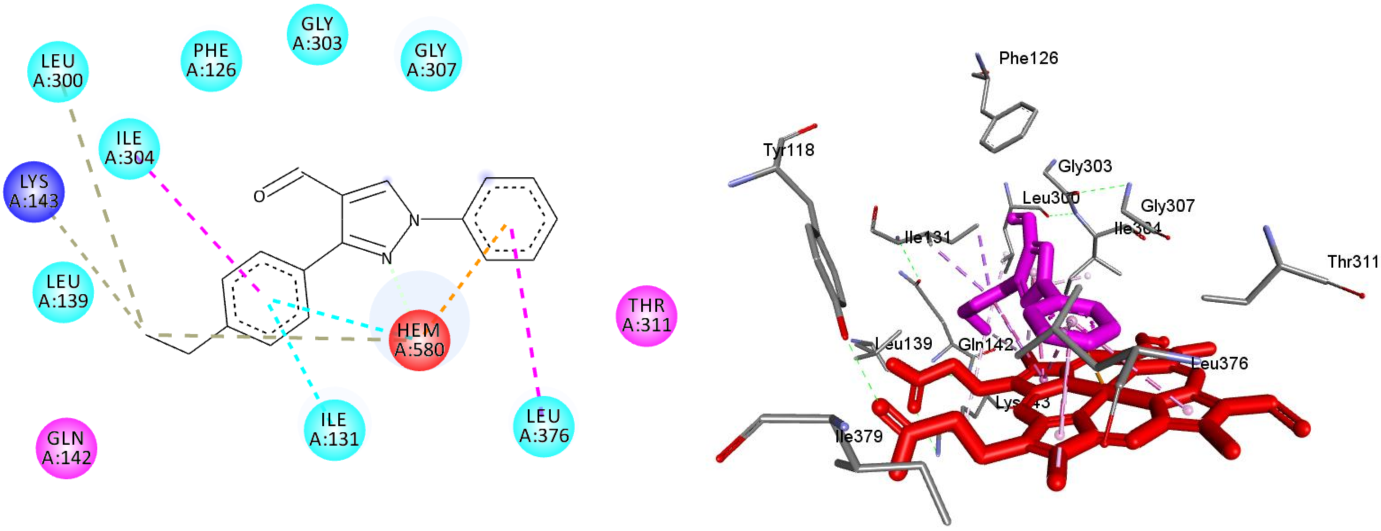

LEU A:300
PHE A:126
GLY A:303
GLY A:307
ILE A:304
LYS A:143
LEU A:139
THR A:311
HEM A:580
ILE A:131
LEU A:376
GLN A:142
Phe126
Tyr118
Gly303
Leu300
Gly307
Ile131
Thr311
Gln142
Leu376
Ile379

4j

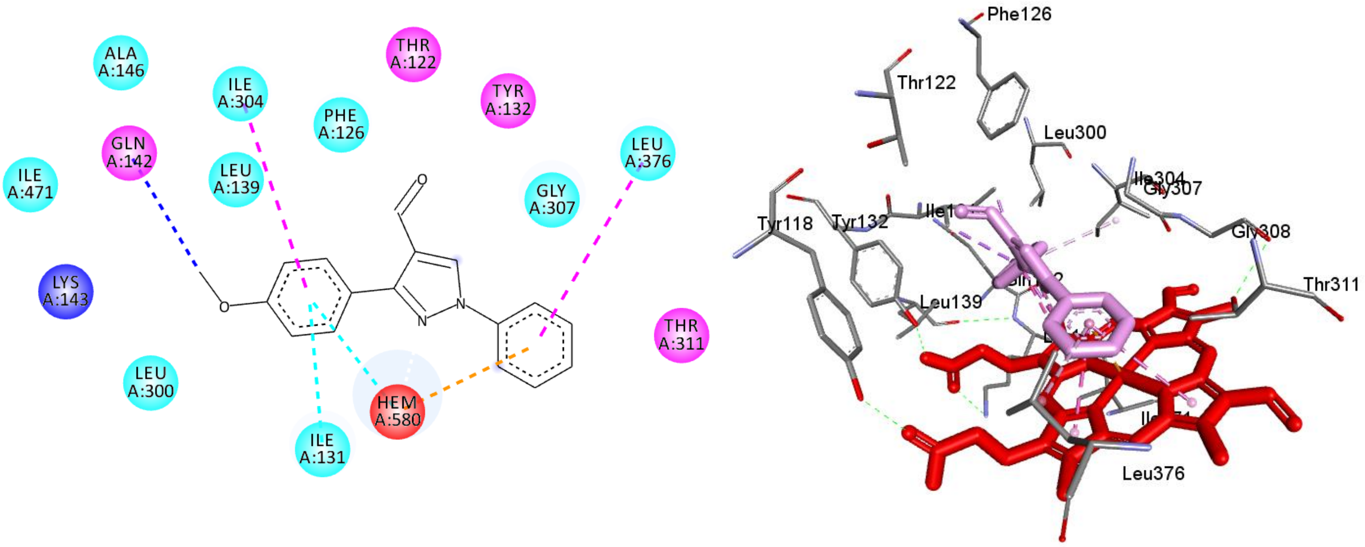

Figure S247. Schematic representation of binding modes of **fluconazole 20** and 1,3-diaryl-1*H*-pyrazole-4-carbaldehydes **4a-j**, at the active site of CYP51Ca (*C. albicans*). The 3D model depicts the hydrophilic bonds and amino acid residues that are part of the active site of CYP51Ca. In the 2D model, the interactions denoted with dotted lines are conventional hydrogen bonds (green), carbon-hydrogen (dark blue), π-cation (orange), π-sigma (cyan), π-alkyl (fuchsia), π-π T-shaped (purple), π-π stacked (yellow), alkyl (gray), π-sulfur (red) and halogen (brown). The heme group is in red, fluconazole **20** (green), **4a** (blue), **4b** (cyan), **4c** (yellow), **4d** (orange), **4e** (purple), **4f** (turquoise), **4g** (red), **4h** (gray), **4i** (fuchsia) and **4j** (pink). The solvent accessible surface is illustrated for the amino acid residues and ligands. The amino acids are highlighted in blue (basic), cyan (hydrophobic), and fuchsia (hydrophilic).

Fluconazole 20

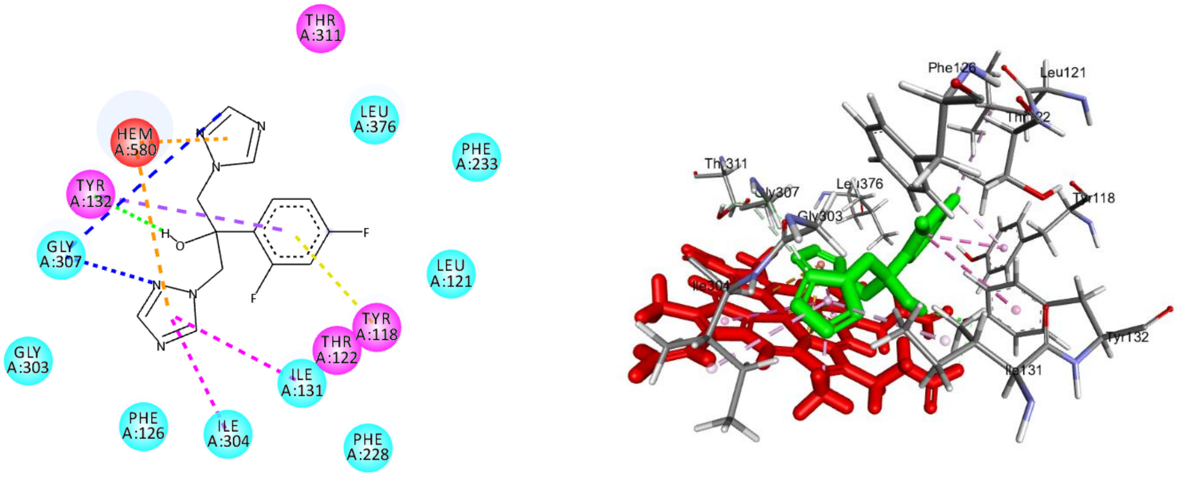

6a

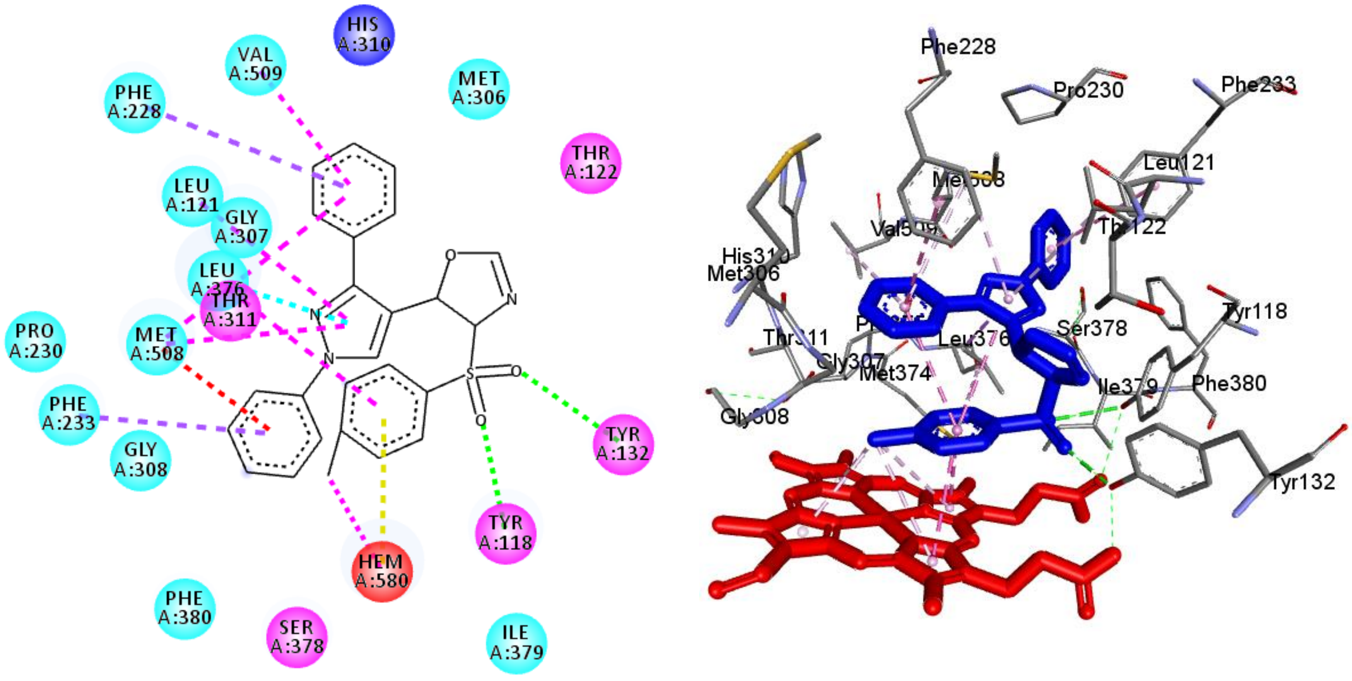

6b

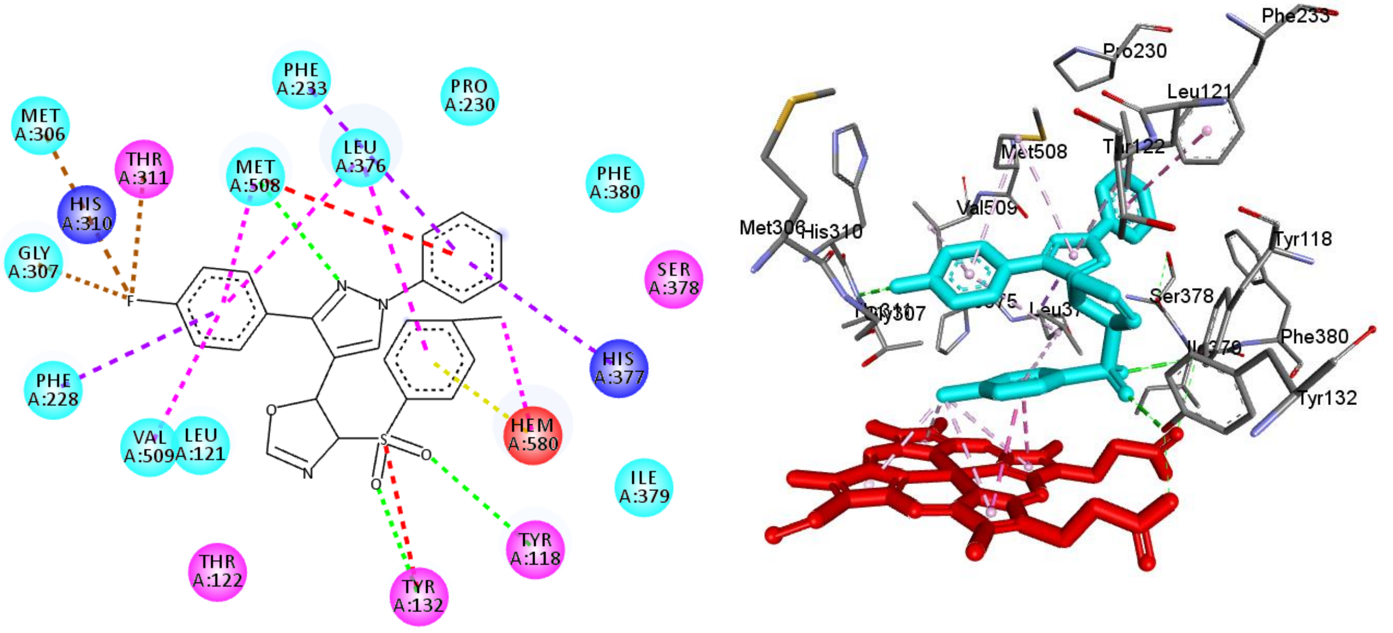
MET A:306
THR A:311
HIS A:310
GLY A:307
PHE A:228
VAL A:509
LEU A:121
MET A:508
PHE A:233
LEU A:376
PRO A:230
PHE A:380
SER A:378
HIS A:377
HEM A:580
ILE A:379
TYR A:118
TYR A:132
THR A:122
Phe233
Pro230
Leu121
Met508
Thr122
Val509
Met306
His310
Tyr118
Ser378
Phe380
Tyr132

6c

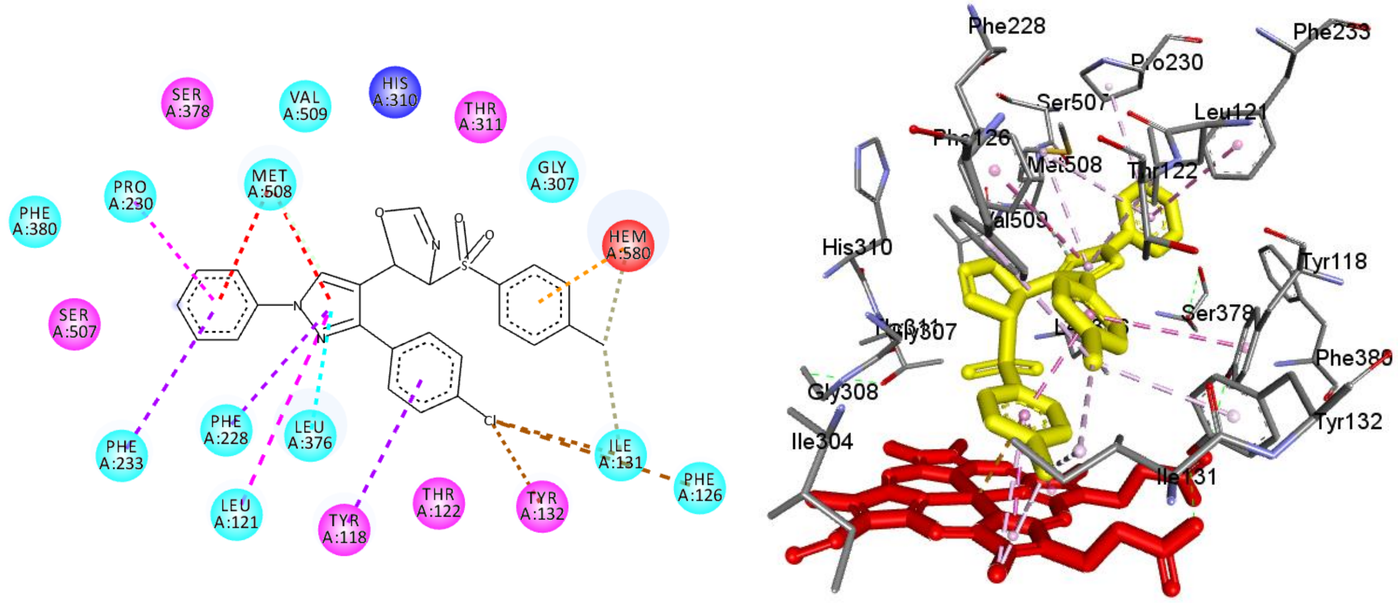
SER A:378
VAL A:509
HIS A:310
THR A:311
PRO A:230
MET A:508
GLY A:307
PHE A:380
HEM A:580
SER A:507
PHE A:233
PHE A:228
LEU A:376
ILE A:131
PHE A:126
LEU A:121
TYR A:118
THR A:122
TYR A:132
Cl
Phe228
Phe233
Pro230
Ser507
Leu121
Met508
Thr122
His310
Tyr118
Ser378
Gly308
Phe380
Ile304
Tyr132

6d

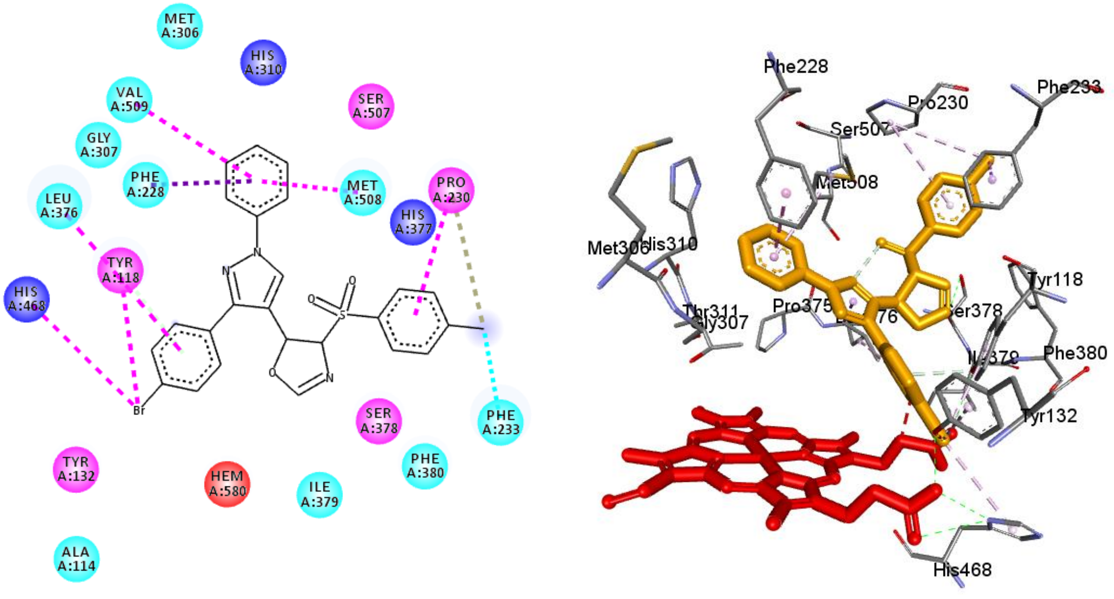
MET A:306
HIS A:310
VAL A:509
SER A:507
GLY A:307
PHE A:228
MET A:508
PRO A:230
LEU A:376
HIS A:377
TYR A:118
HIS A:468
SER A:378
PHE A:233
TYR A:132
HEM A:580
PHE A:380
ILE A:379
ALA A:114
Phe228
Pro230
Phe233
Ser507
Met508
Met306
His310
Tyr118
Thr311
Gly307
Pro375
Phe380
Tyr132
His468

6e

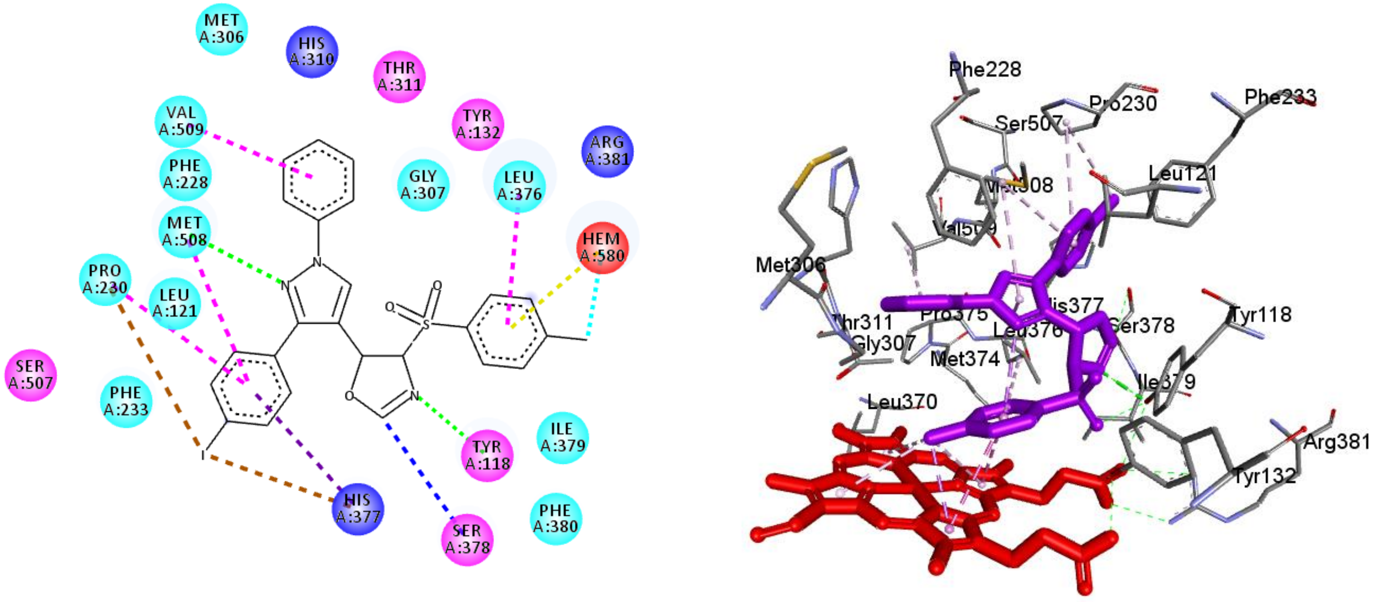
MET A:306
HIS A:310
THR A:311
TYR A:132
VAL A:509
ARG A:381
PHE A:228
GLY A:307
LEU A:376
MET A:508
HEM A:580
PRO A:230
LEU A:121
SER A:507
PHE A:233
TYR A:118
ILE A:379
HIS A:377
SER A:378
PHE A:380
Phe228
Pro230
Phe233
Ser507
Leu121
Met306
Thr311
Gly307
Met374
Ser378
Tyr118
Leu370
Arg381
Tyr132

6f

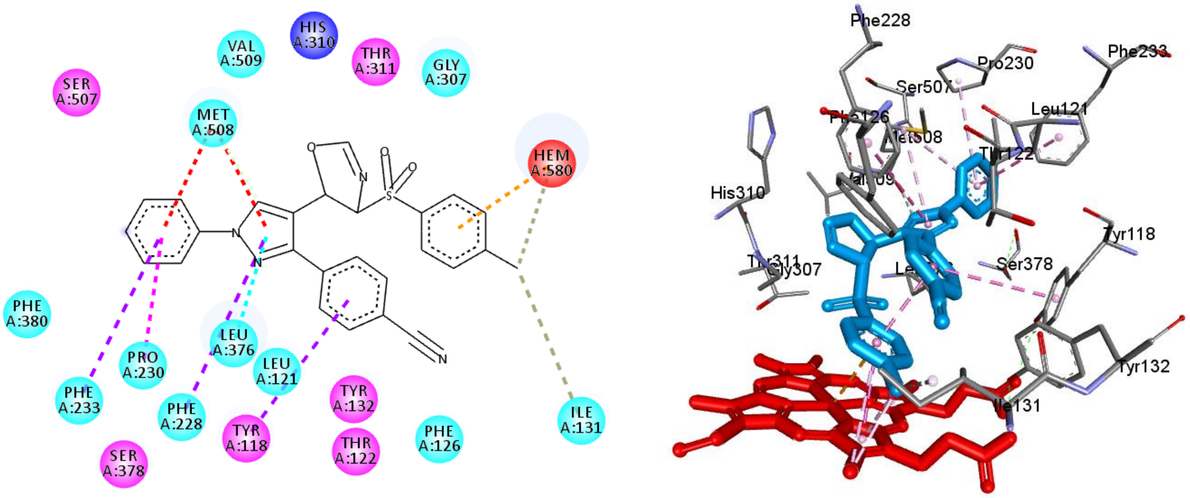

VAL A:509
HIS A:310
THR A:311
GLY A:307
SER A:507
MET A:508
HEM A:580
PHE A:380
LEU A:376
PRO A:230
LEU A:121
PHE A:233
PHE A:228
TYR A:118
TYR A:132
THR A:122
PHE A:126
ILE A:131
SER A:378
Phe228
Pro230
Phe233
Ser507
Leu121
Thr122
His310
Tyr118
Ser378
Tyr132

6g

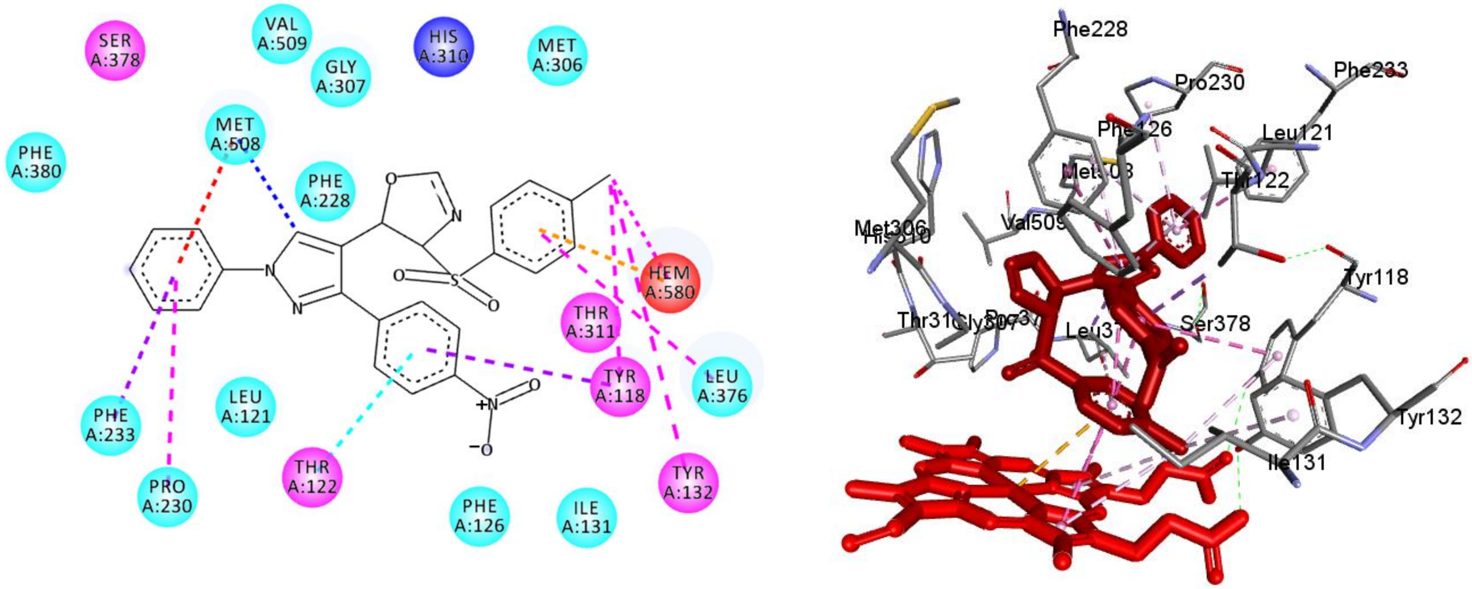

SER A:378
VAL A:509
GLY A:307
HIS A:310
MET A:306
PHE A:380
MET A:508
PHE A:228
HEM A:580
THR A:311
TYR A:118
LEU A:376
PHE A:233
LEU A:121
THR A:122
PRO A:230
TYR A:132
PHE A:126
ILE A:131
Phe228
Pro230
Phe233
Leu121
Thr122
Val509
Tyr118
Ser378
Tyr132
Ile131

6h

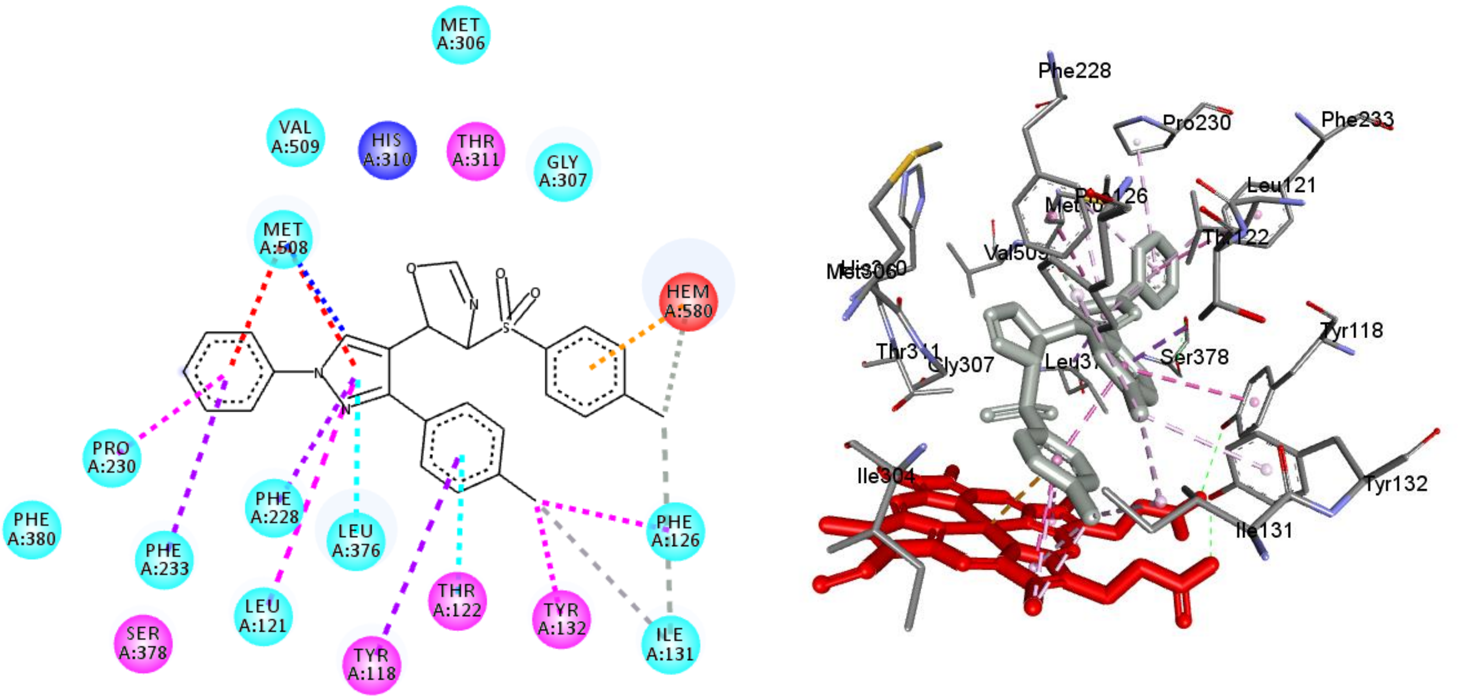
MET A:306
VAL A:509
HIS A:310
THR A:311
GLY A:307
MET A:508
HEM A:580
PRO A:230
PHE A:380
PHE A:228
PHE A:233
LEU A:376
PHE A:126
LEU A:121
THR A:122
TYR A:132
SER A:378
TYR A:118
ILE A:131
Phe228
Pro230
Phe233
Leu121
Thr122
Tyr118
Ser378
Gly307
Ile304
Tyr132
Ile131

6i

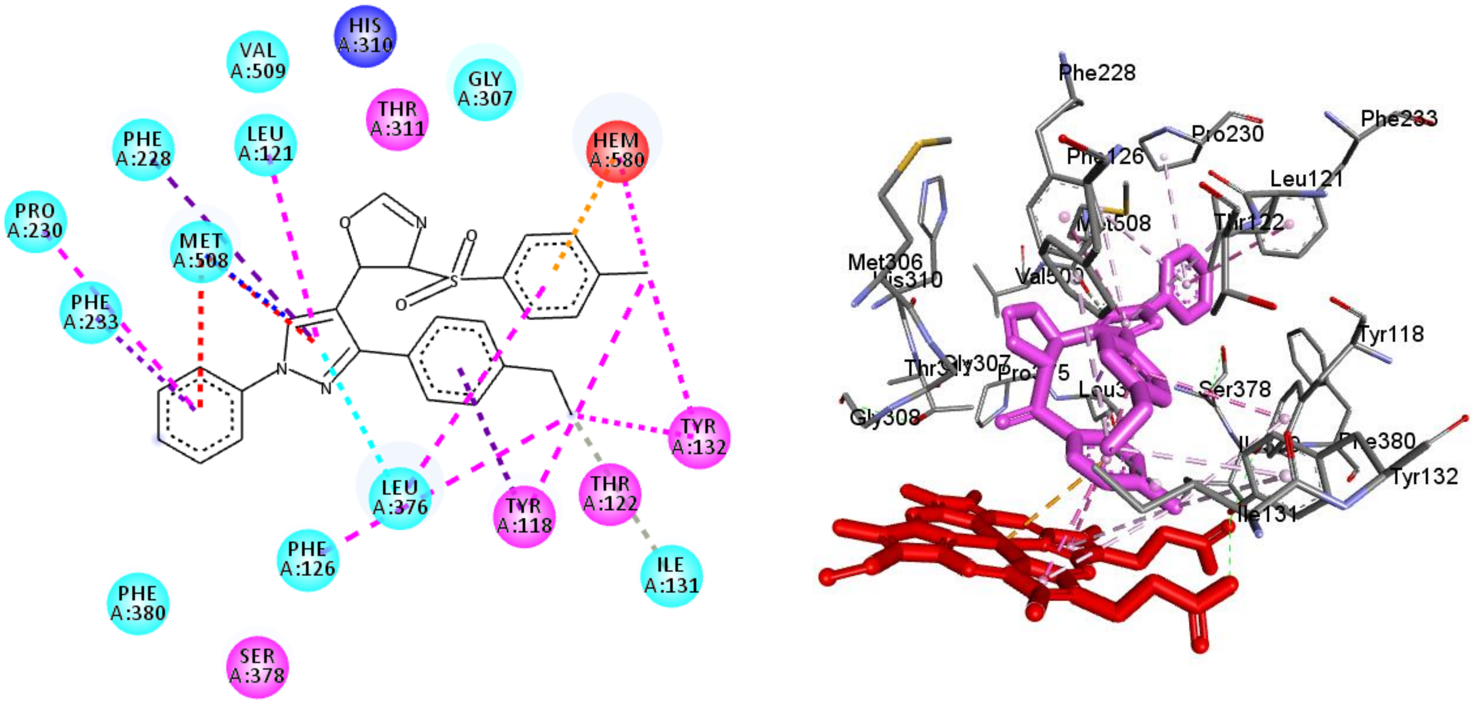
HIS A:310
VAL A:509
GLY A:307
THR A:311
PHE A:228
LEU A:121
HEM A:580
PRO A:230
MET A:508
PHE A:233
TYR A:132
LEU A:376
TYR A:118
THR A:122
PHE A:126
ILE A:131
PHE A:380
SER A:378
Phe228
Pro230
Phe233
Phe126
Leu121
Thr122
Met306
Tyr118
Ser378
Gly308
Tyr132
Ile131

6j

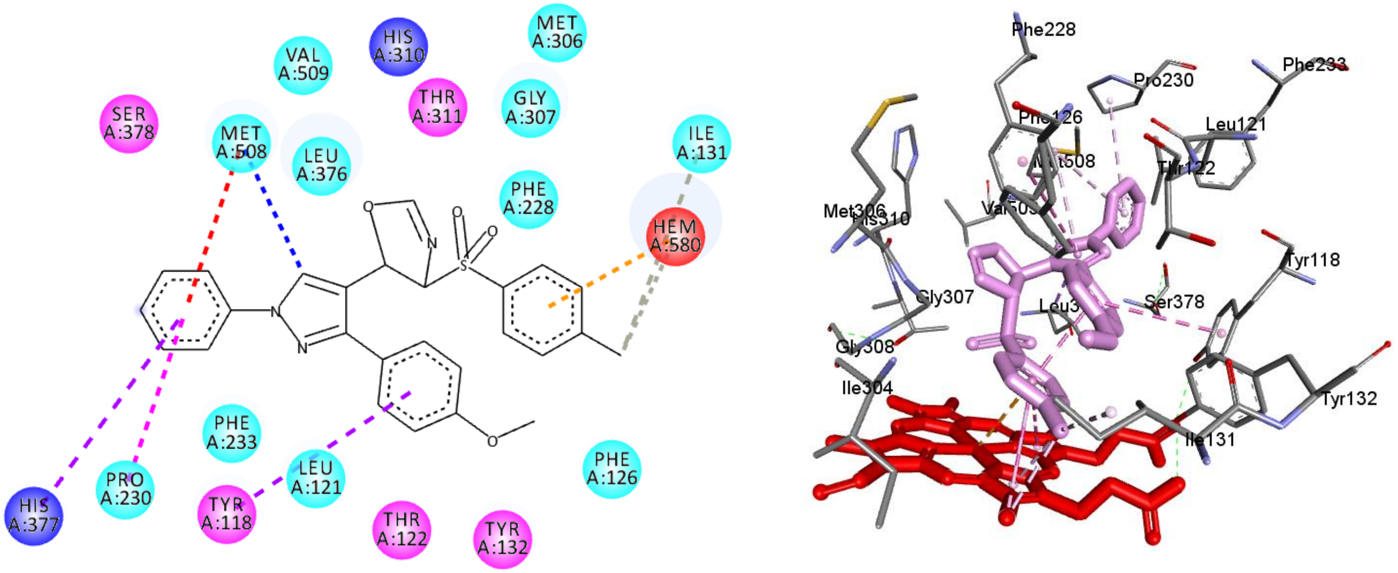

Figure S248. Schematic representation of binding modes of **fluconazole 20** and (4*S**, 5*S**)-5-(1,3-diphenyl-1*H*-pyrazol-4-yl)-4-tosyl-4,5-dihydrooxazoles **6a-j**, at the active site of CYP51Ca (*C. albicans*). The 3D model depicts the hydrophilic bonds and amino acid residues that are part of the active site of CYP51Ca. In the 2D model, the interactions denoted with dotted lines are conventional hydrogen bonds (green), carbon-hydrogen (dark blue), π-cation (orange), π-sigma (cyan), π-alkyl (fuchsia), π-π T-shaped (purple), π-π stacked (yellow), alkyl (gray), π-sulfur (red) and halogen (brown). The heme group is in red, fluconazoles **20** (green), **6a** (blue), **6b** (cyan), **6c** (yellow), **6d** (orange), **6e** (purple), **6f** (turquoise), **6g** (red), **6h** (gray), **6i** (fuchsia) and **6j** (pink). The solvent accessible surface is illustrated for the amino acid residues and ligands. The amino acids are highlighted in blue (basic), cyan (hydrophobic), and fuchsia (hydrophilic).

Table S2. Interactions of 1,3-diaryl-1*H*-pyrazole-4-carbaldehydes **4a-j**, (4*S**, 5*S**)-5-(1,3-diphenyl-1*H*-pyrazol-4-yl)-4-tosyl-4,5-dihydrooxazoles **6a-j** and fluconazole at the active site of lanosterol 14-alpha demethylase CYP51 from *C. glabrata.*

Compound	Interacting residues	Interactions	
		Polar	Hydrophobic
**Fluconazole**	Tyr74, Leu77, Thr78, Phe82, Tyr88, Phe184, Gly258, Val259, Gly262, Gly263, Leu328, Hem478.	O-H·····O (Gly258)<br>C-H·····O (Val259)<br>N·····H-C (Gly262)	π-alkyl (Leu77)<br>π-sigma (Val259)<br>π-sigma (Hem478)<br>halogen (Hem478)
**4a**	Tyr74, Leu77, Thr78, Phe82, Tyr88, Phe184, Gly258, Val259, Gly262, Gly263, Leu328, Hem478.		π-sigma (Ile87)<br>amide-π stacked (Gly258)<br>π- alkyl (Val259)<br>π-alkyl (Hem478)<br>π-π-stacked (Hem478)
**4b**	Thr78, Phe82, Ile87, Tyr88, Phe184, Leu255, Gly258, Val259, Gly262, Gly263, Thr266, Hem478.		π-sigma (Ile87)<br>halogen (Leu255)<br>amide-π stacked (Gly258)<br>π- alkyl (Val259)<br>π-alkyl (Hem478)<br>π-π-stacked (Hem478)
**4c**	Thr78, Phe82, Ile87, Tyr88, Val102, Leu255, Gly258, Val259, Gly262, Gly263, Thr266, Hem478.		π-sigma (Ile87)<br>alkyl (Val102)<br>alkyl (Leu255)<br>amide-π stacked (Gly258)<br>π-alkyl (Val259)<br>π-alkyl (Hem478)<br>π-π-stacked (Hem478)
**4d**	Thr78, Phe82, Ile87, Tyr88, Val102, Leu255, Gly258, Val259, Gly262, Thr266, Hem478.		π- alkyl (Ile87)<br>π- alkyl (Val102)<br>alkyl (Leu255)<br>amide-π stacked (Gly258)<br>π-π stacked (Val259)<br>π-alkyl (Hem478)<br>π-π-stacked (Hem478)<br>π-sigma (Hem478)

**4e**	Thr78, Phe82, Ile87, Tyr88, Val102, Leu255, Gly258, Val259, Gly262, Thr266, Leu328, Hem478.		π-alkyl (Ile87)<br>π-alkyl (Val102)<br>π-alkyl (Leu255)<br>amide-π stacked (Gly258)<br>π-alkyl (Val259)<br>π-alkyl (Leu328)<br>π-alkyl (Hem478)<br>π-π-stacked (Hem478)
**4f**	Thr78, Phe82, Ile87, Tyr88, Val102, Leu255, Gly258, Val259, Gly262, Gly263, Thr266, Hem478.		π- alkyl (Ile87)<br>amide-π stacked (Gly258)<br>π-alkyl (Val259)<br>π-alkyl (Hem478)<br>π-π-stacked (Hem478)
**4g**	Thr78, Phe82, Ile87, Tyr88, Gln98, Val102, Leu255, Gly258, Val259, Gly262, Gly263, Thr266, Hem478.	O·····H-O (Leu255)	π-alkyl (Ile87)<br>amide-π stacked (Gly258)<br>π-alkyl (Val259)<br>π-alkyl (Hem478)<br>π-π-stacked (Hem478)<br>π-π T-shaped (Hem478)
**4h**	Thr78, Phe82, Ile87, Tyr88, Val102, Leu255, Gly258, Val259, Gly262, Gly263, Thr266, Hem478.		π-alkyl (Ile87)<br>alkyl (Val102)<br>alkyl (Leu255)<br>amide-π stacked (Gly258)<br>alkyl (Val259)<br>π-alkyl (Val259)<br>π-alkyl (Hem478)<br>π-π-stacked (Hem478)
**4i**	Thr78, Phe82, Ile87, Tyr88, Val102, Leu255, Gly258, Val259, Gly262, Gly263, Thr266, Leu328, Hem478.		π-alkyl (Ile87)<br>π-alkyl (Val102)<br>π-alkyl (Leu255)<br>amide-π stacked (Gly258)<br>π-alkyl (Val259)<br>alkyl (Leu328)<br>π-alkyl (Hem478)<br>π-sigma (Hem478)<br>π-π-stacked (Hem478)
**4j**	Phe82, Ile87, Phe184, Leu255, Gly258, Val259, Gly262, Gly263, Thr266, Leu328, Hem478.	C-H·····O (Gln142)	π-alkyl (Ile87)<br>amide-π stacked (Gly258)<br>π-alkyl (Val259)

			π-sigma (Gly262)<br>alkyl (Leu328)<br>π-alkyl (Hem478)<br>π-π T-shaped (Hem478)<br>π-sigma (Hem478)<br>π-π-stacked (Hem478)
**6a**	Tyr74, Leu77, Thr78, Phe82, Ile87, Tyr88, Phe184, Leu255, Gly258, Val259, Gly262, Thr266, Leu328, Met457, Hem478.	N····H-N (Hem478)	π-alkyl (Leu77)<br>π-sigma (Ile87)<br>π-alkyl (Phe184)<br>amide-π stacked (Gly258)<br>π-alkyl (Val259)<br>π-sigma (Leu328)<br>alkyl (Met457)<br>π-alkyl (Hem478)<br>π-sulfur (Hem478)
**6b**	Tyr74, Leu77, Thr78, Phe82, Ile87, Tyr88, Phe184, Leu255, Gly258, Val259, Gly262, Gly263, His265, Thr266, Leu328, Met457, Val458, Hem478.	N····H-N (Hem478)	π-sigma (Ile87)<br>π-alkyl (Phe184)<br>amide-π stacked (Gly258)<br>π-alkyl (Val259)<br>π-alkyl (His265)<br>alkyl (Met457)<br>π-alkyl (Met457)<br>alkyl (Val458)<br>π-alkyl (Hem478)
**6c**	Tyr74, Leu77, Thr78, Phe82, Ile87, Tyr88, Phe184, Leu255, Gly258, Val259, Gly262, Thr266, Met457, Hem478.	O····H-C (Gly262)<br>C-H····N (Hem478)	π-π T-shaped (Tyr74)<br>π-alkyl (Ile87)<br>π-sulfur (Phe184)<br>amide-π stacked (Gly258)<br>π-alkyl (Val259)<br>alkyl (Met457)<br>π-alkyl (Met457)<br>π-alkyl (Hem478)
**6d**	Tyr74, Leu77, Thr78, Phe82, Ile87, Tyr88, Phe184, Leu255, Gly258, Val259, Gly262, Thr266, Leu328, Met457, Hem478.	O····H-C (Gly262)<br>O····H-C (Hem478)	π-alkyl (Tyr74)<br>alkyl (Leu77)<br>π-alkyl (Ile87)<br>π-sulfur (Phe184)<br>amide-π stacked (Gly258)<br>π-alkyl (Val259)<br>π-alkyl (Leu328)<br>π-alkyl (Met457)<br>π-alkyl (Hem478)
**6e**	Tyr74, Leu77, Thr78, Phe82, Ile87, Tyr88, Phe184, Leu255, Gly258, Val259, Gly262,	N····H-O (Tyr78)<br>N····H-N (Hem478)	π-alkyl (Tyr74)<br>π-π T-shaped (Tyr74)

	Gly263, His265, Thr266, Leu328, Met457, Val458, Hem478.		alkyl (Leu77)<br>π-alkyl (Ile87)<br>π-alkyl (Phe184)<br>amide-π stacked (Gly258)<br>π-alkyl (Val259)<br>π-alkyl (His265)<br>π-alkyl (Met457)<br>π-alkyl (Hem478)<br>π-sulfur (Hem478)
**6f**	Tyr74, Leu77, Thr78, Ile87, Tyr88, Phe184, Gly258, Val259, Thr266, His325, Pro327, Leu328, Met457, Hem478.	N····H-C (His326)<br>N····H-N (Leu328)	π-sigma (Leu77)<br>π-alkyl (Ile87)<br>π-sulfur (Phe184)<br>π-alkyl (Val259)<br>π-alkyl (Leu328)<br>π-sigma (Met457)<br>π-π T-shaped (Hem478)<br>π-sigma (Hem478)<br>π-π-stacked (Hem478)
**6g**	Tyr74, Leu77, Thr78, Phe82, Ile87, Tyr88, Phe184, Leu255, Gly258, Val259, Gly262, Gly263, Thr266, Leu328, Met457, Val458, Hem478.	O····H-O (Thr78)<br>N····H-N (Hem478)	π-sigma (Ile87)<br>alkyl (Phe184)<br>amide-π stacked (Gly258)<br>π-alkyl (Val259)<br>π-alkyl (Leu328)<br>alkyl (Met457)<br>alkyl (Val458)<br>π-alkyl (Hem478)<br>π-sulfur (Hem478)
**6h**	Tyr74, Leu77, Thr78, Phe82, Ile87, Tyr88, Phe184, Leu255, Gly258, Val259, Gly262, Gly263, His265, Thr266, Leu328, Met457, Val458, Hem478.	N····H-N (Hem478)	π-alkyl (Tyr74)<br>alkyl (Leu77)<br>π-alkyl (Ile87)<br>π-alkyl (Phe184)<br>amide-π stacked (Gly258)<br>π-alkyl (Val259)<br>π-alkyl (His265)<br>alkyl (Val458)<br>π-alkyl (Hem478)<br>π-sulfur (Hem478)
**6i**	Tyr74, Thr78, Ile87, Tyr88, Phe184, Leu255, Gly258, Val259, Gly262, His265, Thr266, Pro327, Leu328, Met457, Val458, Hem478.		π-alkyl (Ile87)<br>π-sigma (Ile87)<br>amide-π stacked (Gly258)<br>π-alkyl (Val259)<br>π-sigma (Leu328)<br>π-alkyl (Hem478)<br>π-π T-shaped (Hem478)

**6j**	Tyr74, Leu77, Thr78, Ile87, Tyr88, Phe184, Gly258, Val259, Gly262, Thr266, His326, Pro327, Leu328, Met457, Hem478.	O····H-O (Thr78)<br>C-H····O (Tyr88)	π-alkyl (Leu77)<br>π-sigma (Ile87)<br>π-sulfur (Phe184)<br>π-alkyl (Val259)<br>π-alkyl (His326)<br>alkyl (Pro327)<br>alkyl (Leu328)<br>π-alkyl (Leu328)<br>π-sigma (Leu328)<br>π-sigma (Met457)<br>alkyl (Hem478)<br>π-π T-shaped (Hem478)<br>π-π-stacked (Hem478)

Fluconazole

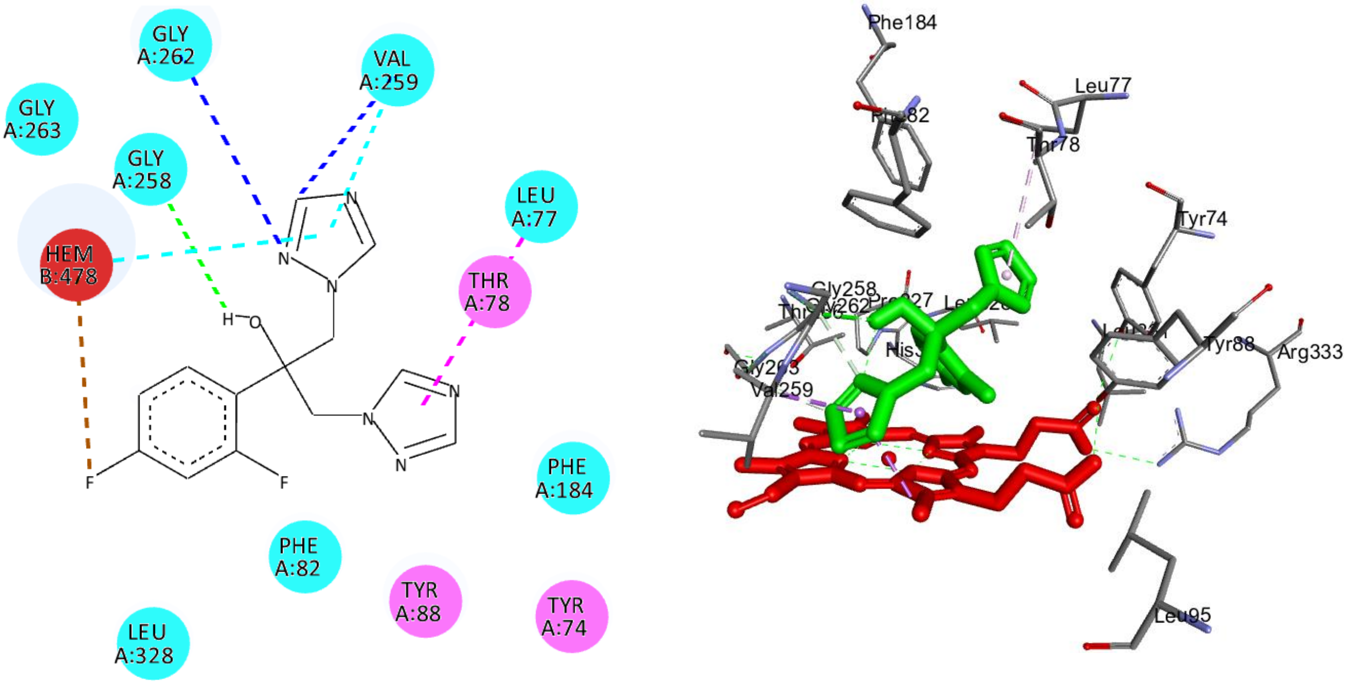

4a

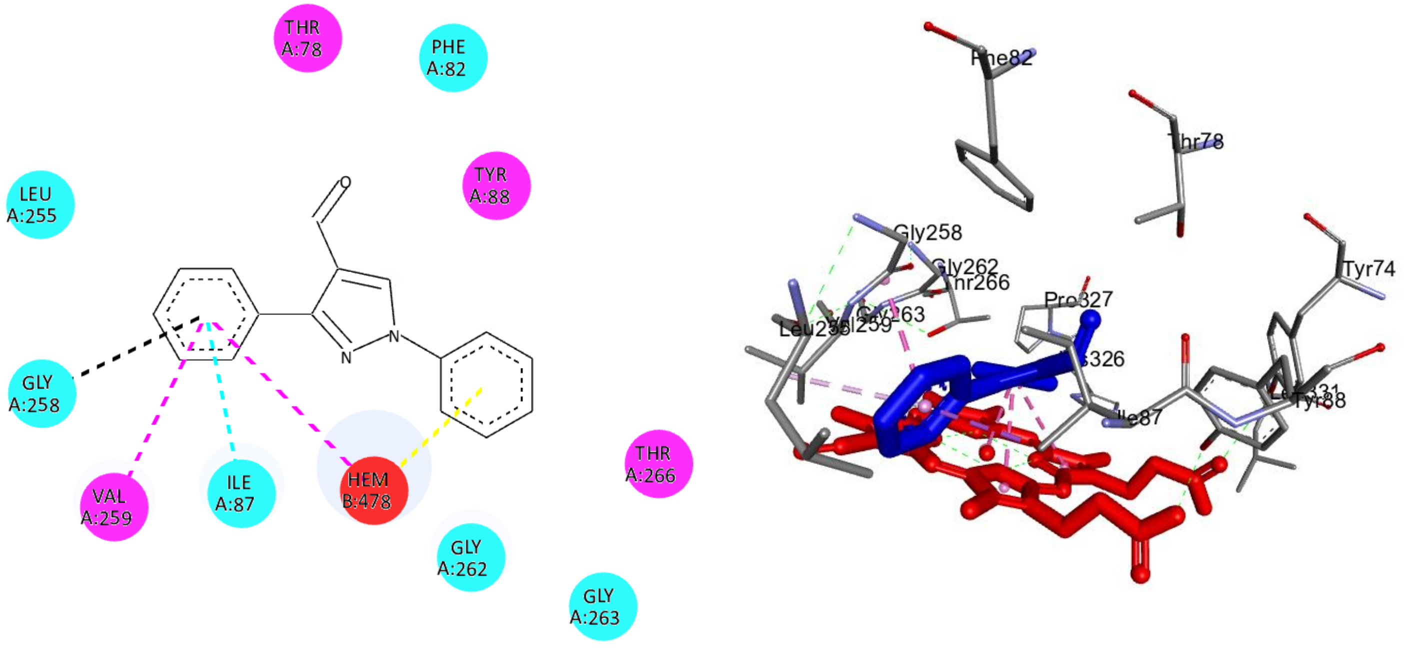
THR A:78
PHE A:82
LEU A:255
TYR A:88
GLY A:258
VAL A:259
ILE A:87
HEM B:478
THR A:266
GLY A:262
GLY A:263
O
N
N
Phe82
Thr78
Gly258
Gly262
Thr266
Gly263
Pro327
Tyr74
Ile87
Tyr88

4b

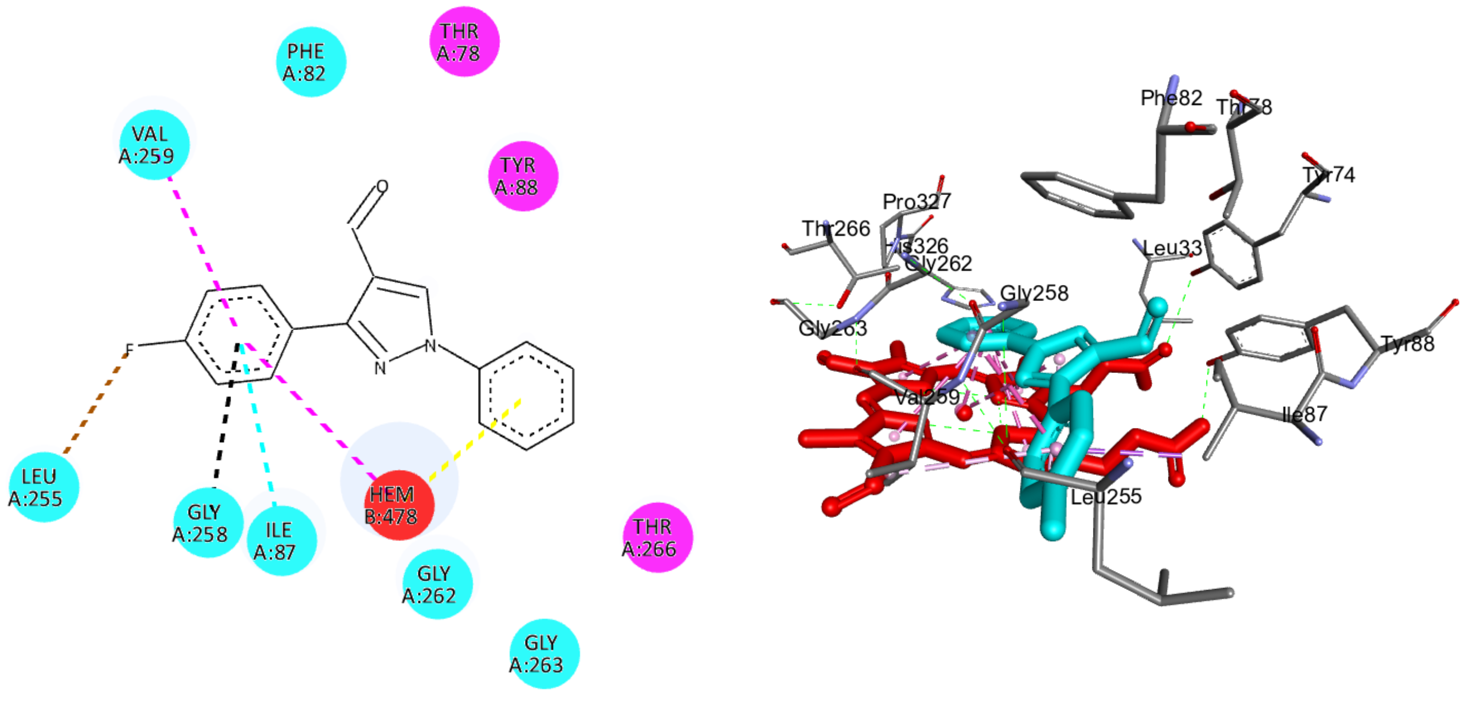
PHE A:82
THR A:78
VAL A:259
TYR A:88
O
F
N
N
LEU A:255
GLY A:258
ILE A:87
HEM B:478
THR A:266
GLY A:262
GLY A:263
Phe82
Thr78
Tyr74
Pro327
Thr266
His326
Gly262
Gly258
Gly263
Val259
Tyr88
Ile87
Leu255

4c

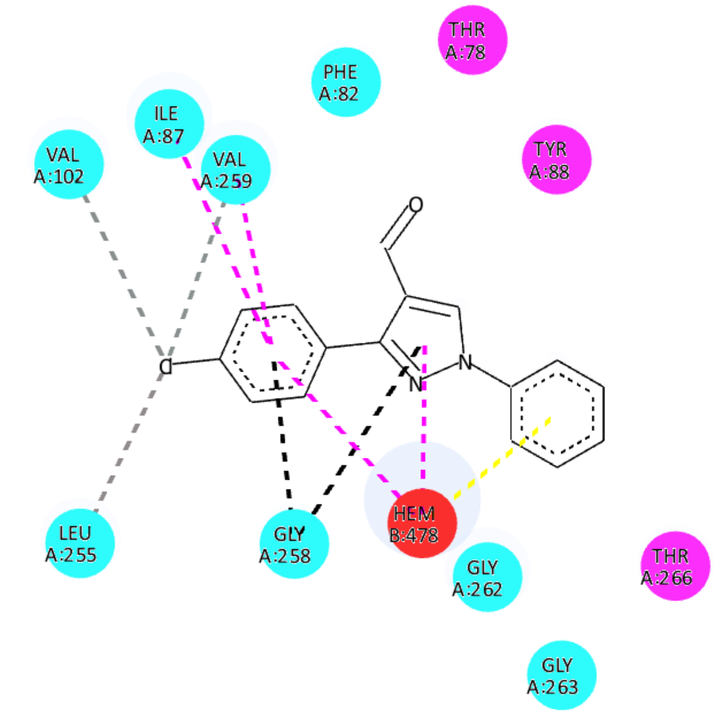
THR A:78
PHE A:82
ILE A:87
VAL A:102
VAL A:259
TYR A:88
Cl
N
N
O
LEU A:255
GLY A:258
HEM B:478
GLY A:262
THR A:266
GLY A:263

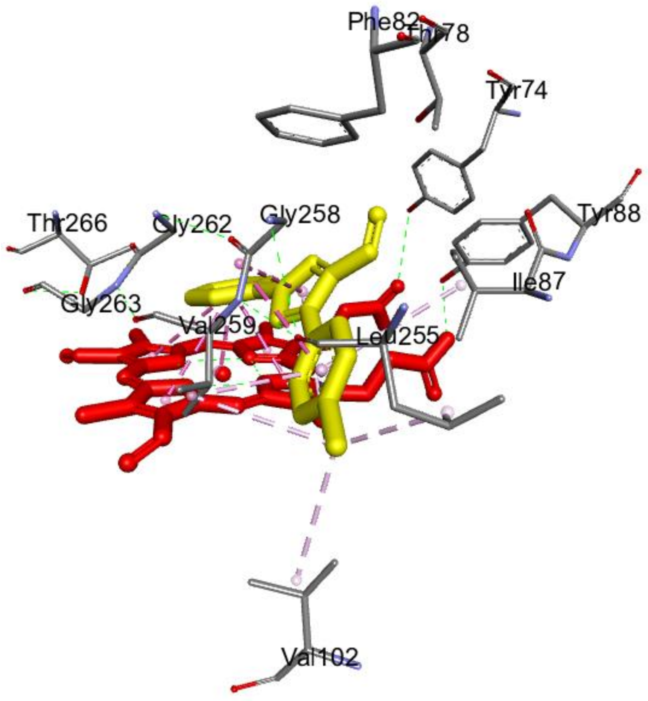
Phe82
Thr78
Tyr74
Thr266
Gly262
Gly258
Tyr88
Ile87
Gly263
Val259
Leu255
Val102

4d

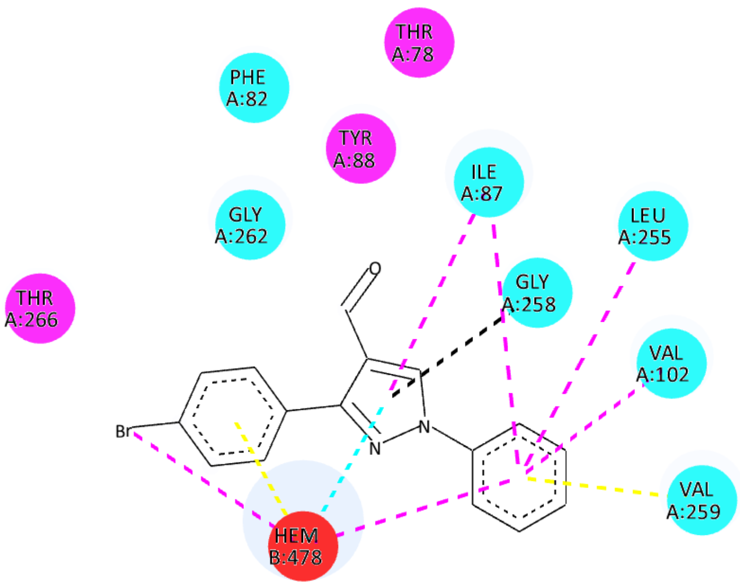
THR A:78
PHE A:82
TYR A:88
ILE A:87
GLY A:262
LEU A:255
THR A:266
GLY A:258
VAL A:102
Br
N
N
O
HEM B:478
VAL A:259

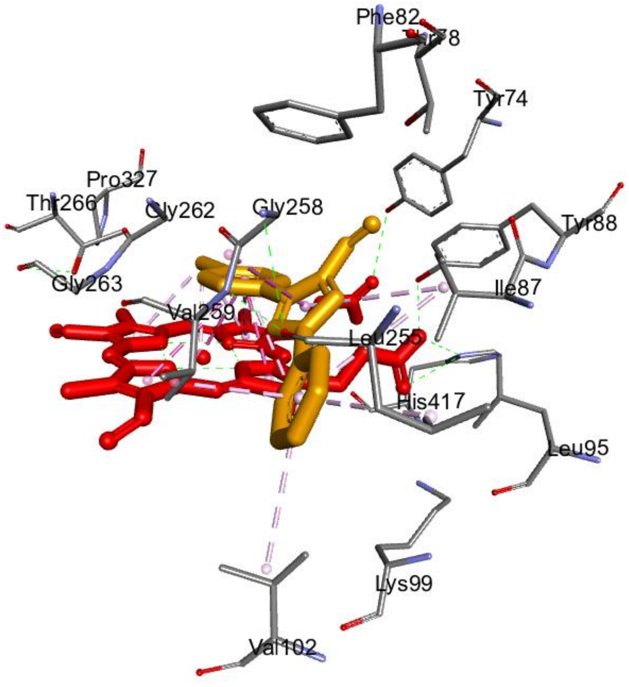
Phe82
Thr78
Tyr74
Pro327
Thr266
Gly262
Gly258
Tyr88
Gly263
Ile87
Val259
Leu255
His417
Leu95
Lys99
Val102

4e

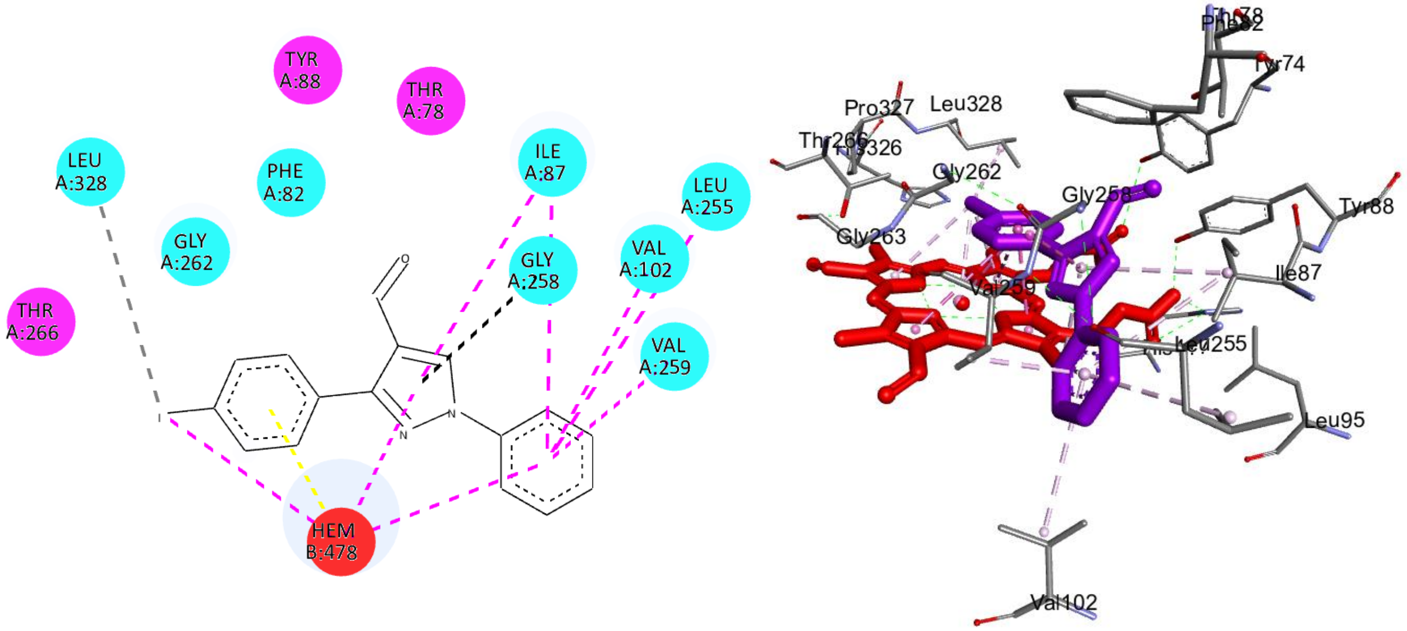
TYR A:88
THR A:78
LEU A:328
PHE A:82
ILE A:87
LEU A:255
GLY A:262
GLY A:258
VAL A:102
THR A:266
VAL A:259
HEM B:478
O
N
N
I
Phe82
Tyr74
Pro327
Leu328
Gly262
Gly258
Tyr88
Gly263
Ile87
Val259
Leu255
Leu95
Val102

4f

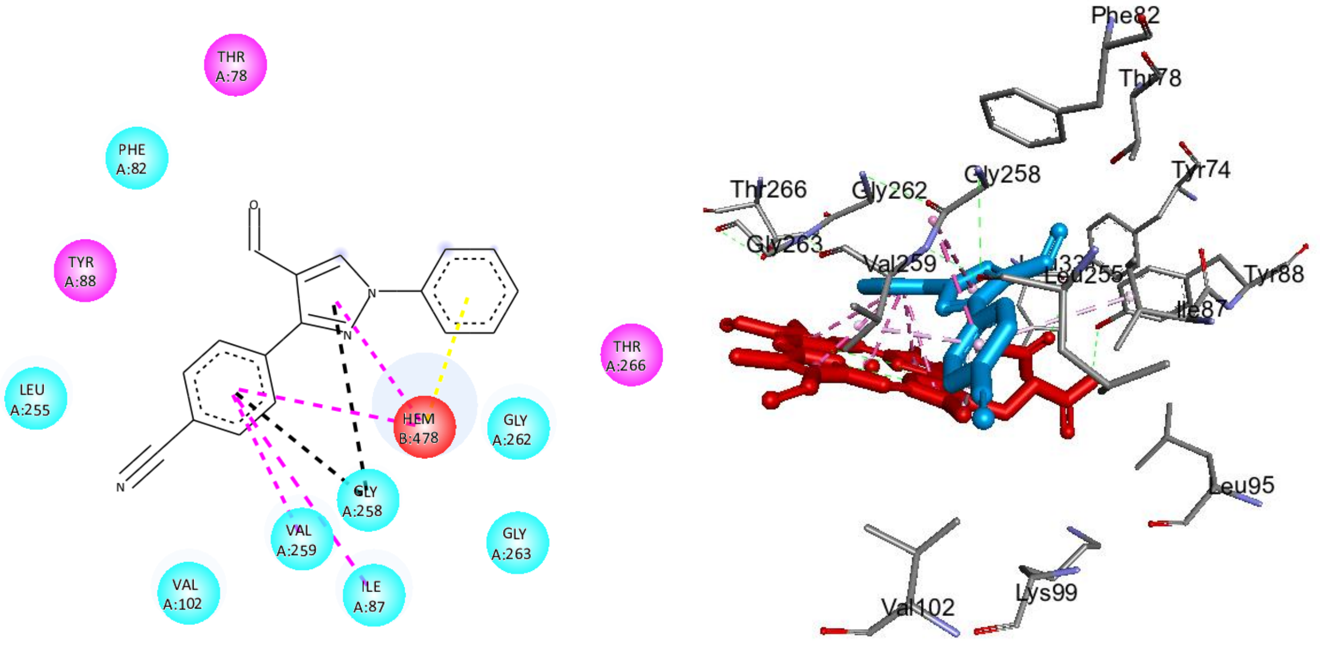
THR A:78
PHE A:82
TYR A:88
LEU A:255
THR A:266
HEM B:478
GLY A:262
GLY A:258
VAL A:259
GLY A:263
VAL A:102
ILE A:87
O
N
N
N
Phe82
Thr78
Thr266
Gly262
Gly258
Tyr74
Gly263
Val259
Leu255
Tyr88
Ile87
Leu95
Val102
Lys99

4g

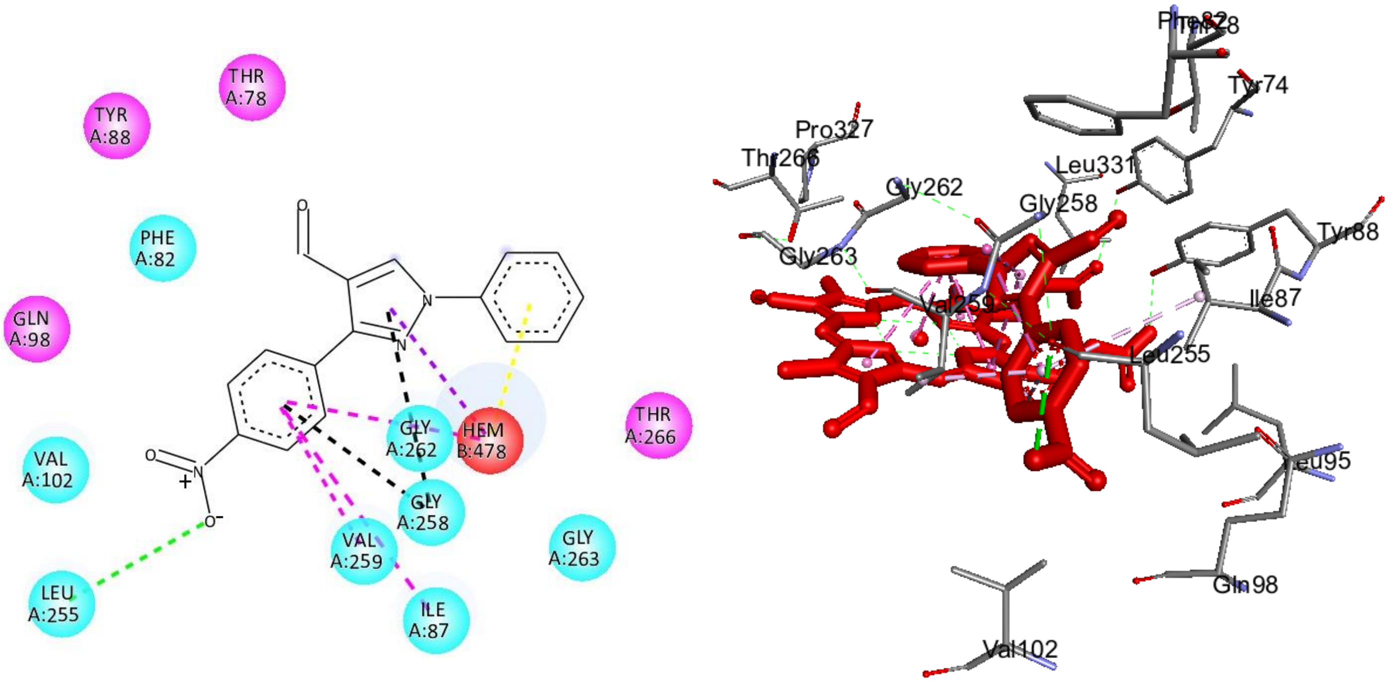
TYR A:88
THR A:78
PHE A:82
GLN A:98
VAL A:102
LEU A:255
VAL A:259
ILE A:87
GLY A:262
HEM B:478
GLY A:258
GLY A:263
THR A:266
Tyr74
Pro327
Thr266
Gly262
Leu331
Gly258
Tyr88
Gly263
Val259
Ile87
Leu255
Gln98
Val102

4h

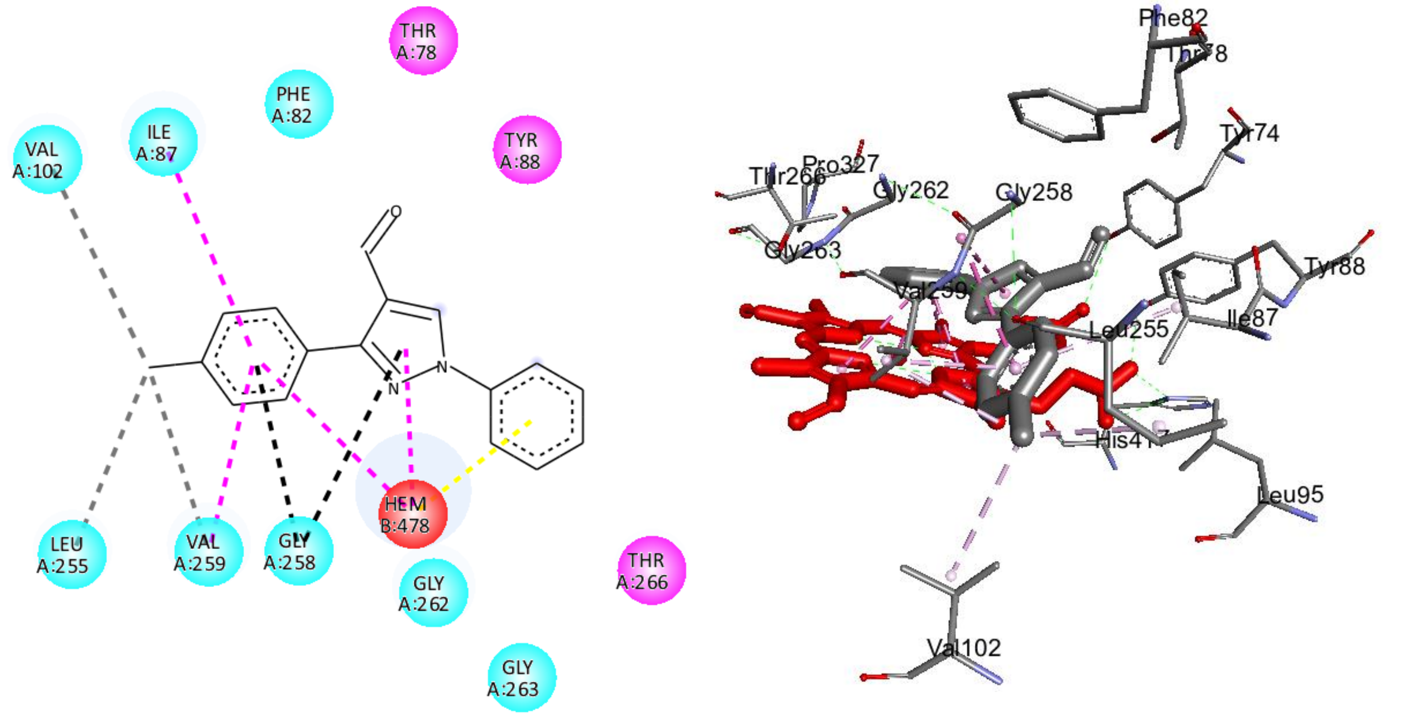
THR A:78
PHE A:82
VAL A:102
ILE A:87
TYR A:88
LEU A:255
VAL A:259
GLY A:258
HEM B:478
GLY A:262
GLY A:263
THR A:266
Phe82
Tyr74
Pro327
Gly262
Gly258
Gly263
Tyr88
Ile87
Leu255
Leu95
Val102

4i

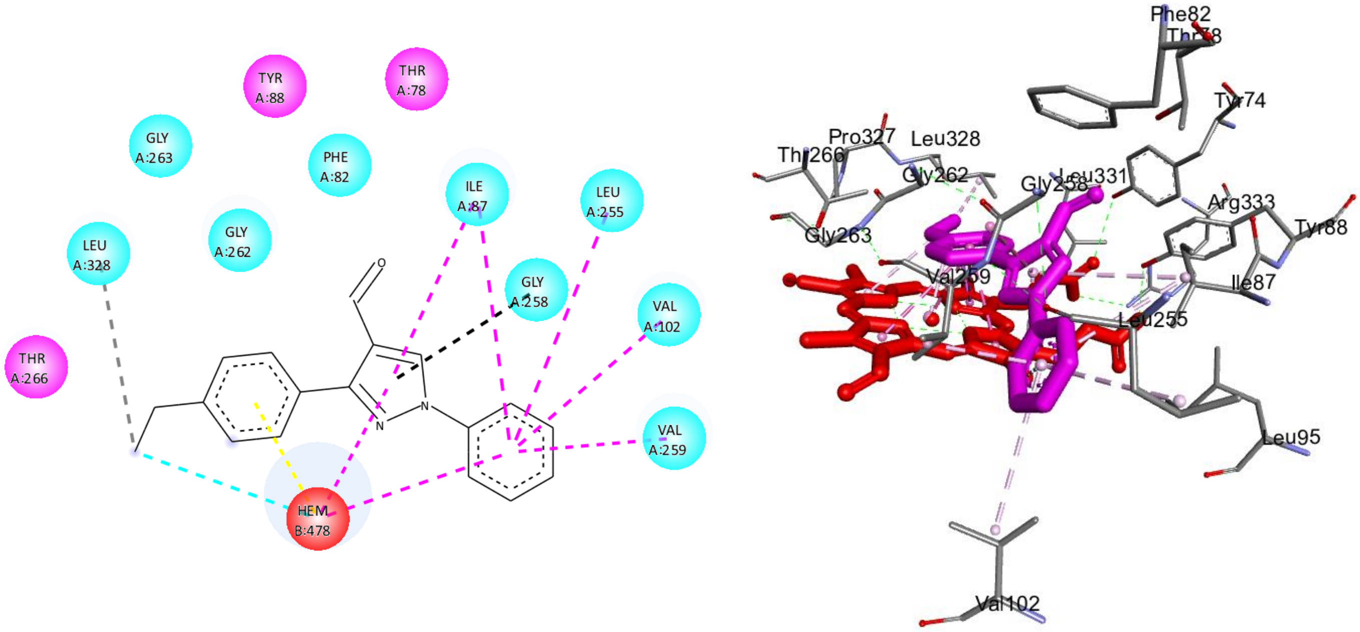

4j

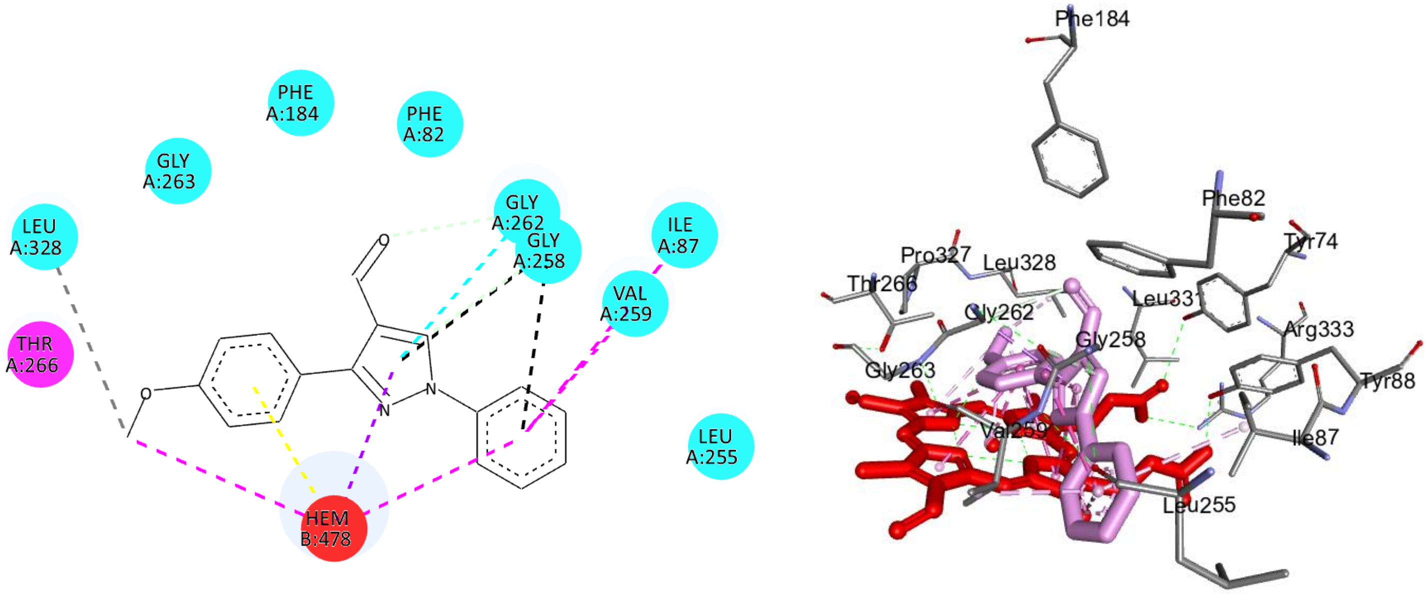

Figure S249. Schematic representation of binding modes of **fluconazole 20** and 1,3-diaryl-1*H*-pyrazole-4-carbaldehydes **4a-j**, at the active site of CYP51Cg (*C. glabrata*). The 3D

model depicts the hydrophilic bonds and amino acid residues that are part of the active site of CYP51Cg. In the 2D model, the interactions denoted with dotted lines are conventional hydrogen bonds (green), carbon-hydrogen (dark blue), π-cation (orange), π-sigma (cyan), π-alkyl (fuchsia), π-π T-shaped (purple), π-π stacked (yellow), alkyl (gray), π-sulfur (red) and halogen (brown). The heme group is in red, fluconazole **20** (green), **4a** (blue), **4b** (cyan), **4c** (yellow), **4d** (orange), **4e** (purple), **4f** (turquoise), **4g** (red), **4h** (gray), **4i** (fuchsia) and **4j** (pink). The solvent accessible surface is illustrated for the amino acid residues and ligands. The amino acids are highlighted in blue (basic), cyan (hydrophobic), and fuchsia (hydrophilic).

6a

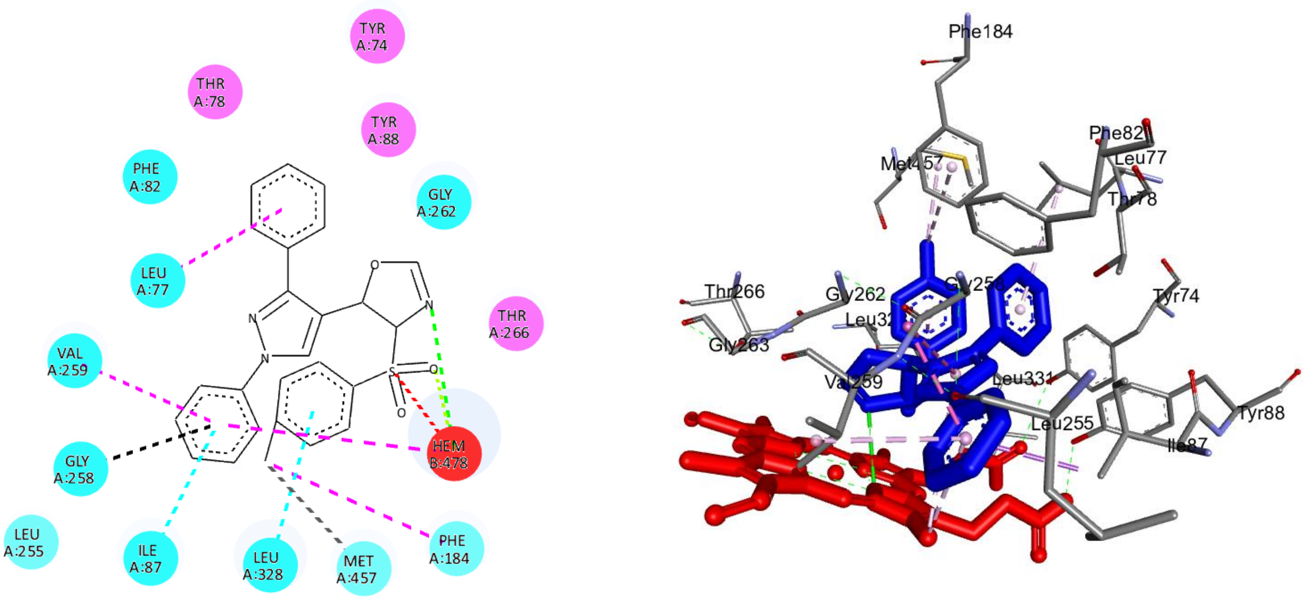

6b

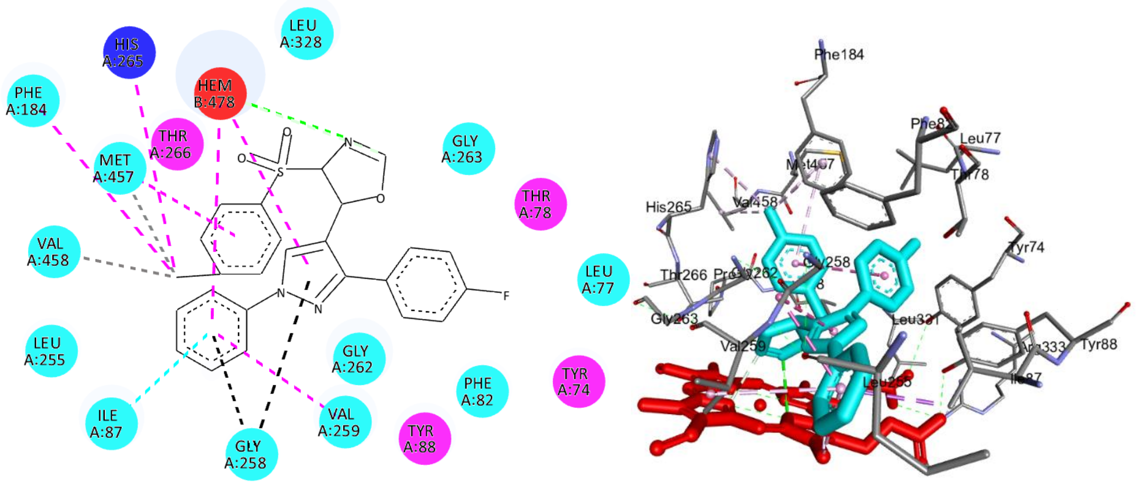
LEU A:328
HIS A:265
HEM B:478
PHE A:184
THR A:266
MET A:457
GLY A:263
THR A:78
VAL A:458
LEU A:77
LEU A:255
GLY A:262
PHE A:82
TYR A:74
ILE A:87
VAL A:259
TYR A:88
GLY A:258
Phe184
Leu77
His265
Val458
Tyr74
Thr266
Gly263
Val259
Tyr88

6c

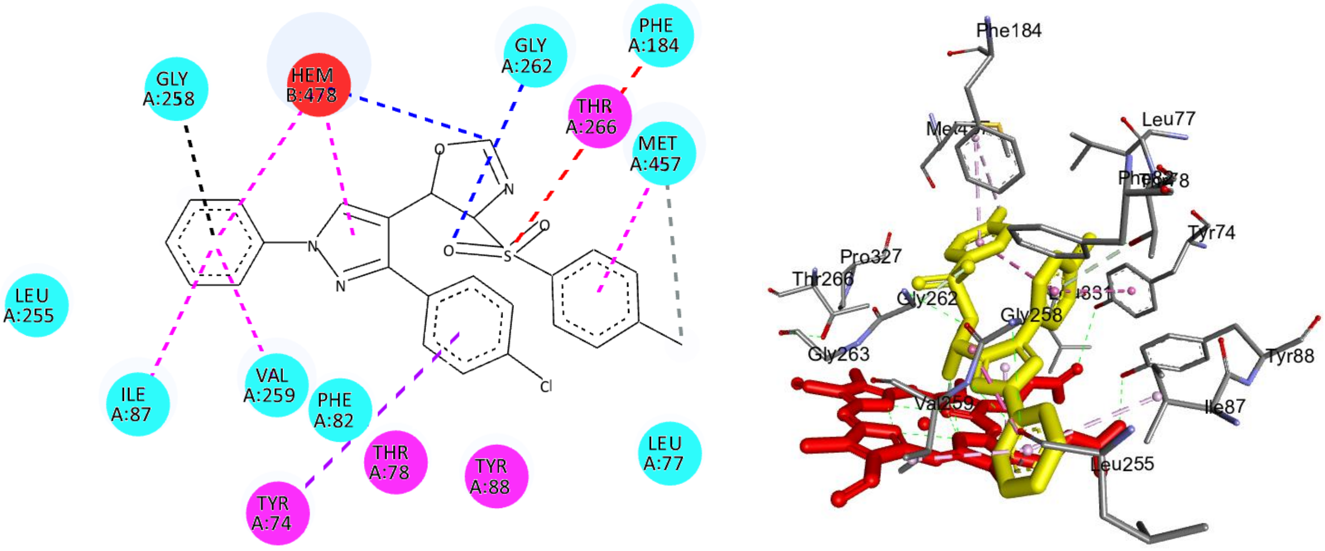
GLY A:258
HEM B:478
GLY A:262
PHE A:184
THR A:266
MET A:457
LEU A:255
ILE A:87
VAL A:259
PHE A:82
THR A:78
TYR A:88
LEU A:77
TYR A:74
Cl
Phe184
Leu77
Tyr74
Pro327
Thr266
Gly262
Gly258
Gly263
Tyr88
Ile87
Val259
Leu255

6d

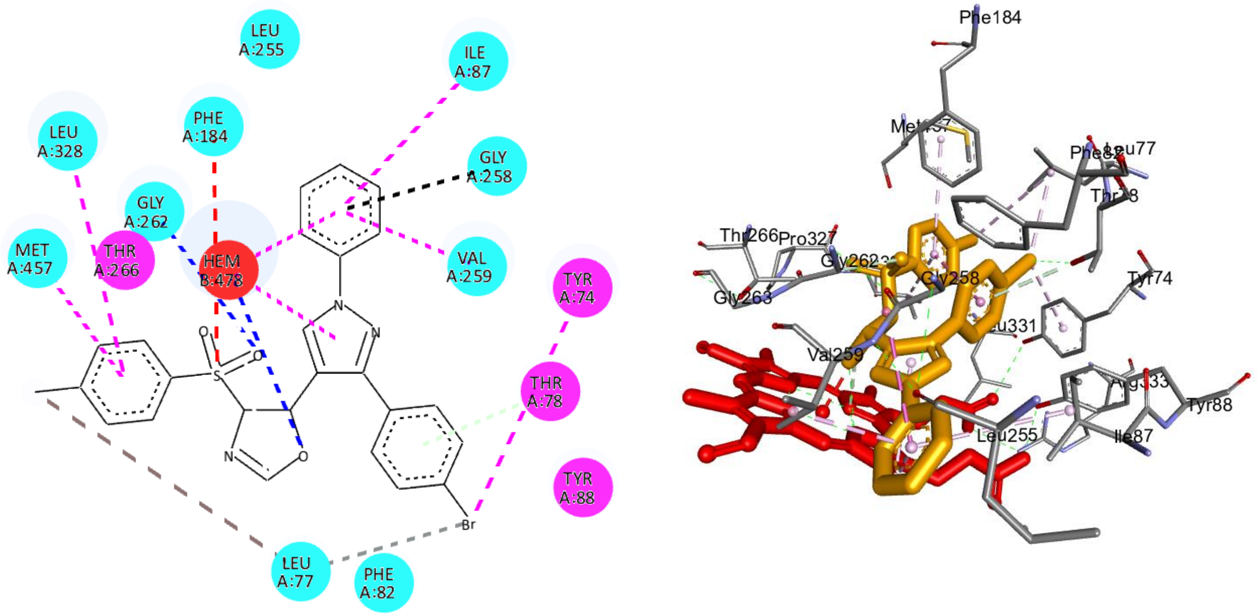
LEU A:255
ILE A:87
LEU A:328
PHE A:184
GLY A:258
GLY A:262
MET A:457
THR A:266
HEM B:478
VAL A:259
TYR A:74
THR A:78
TYR A:88
LEU A:77
PHE A:82
Br

6e

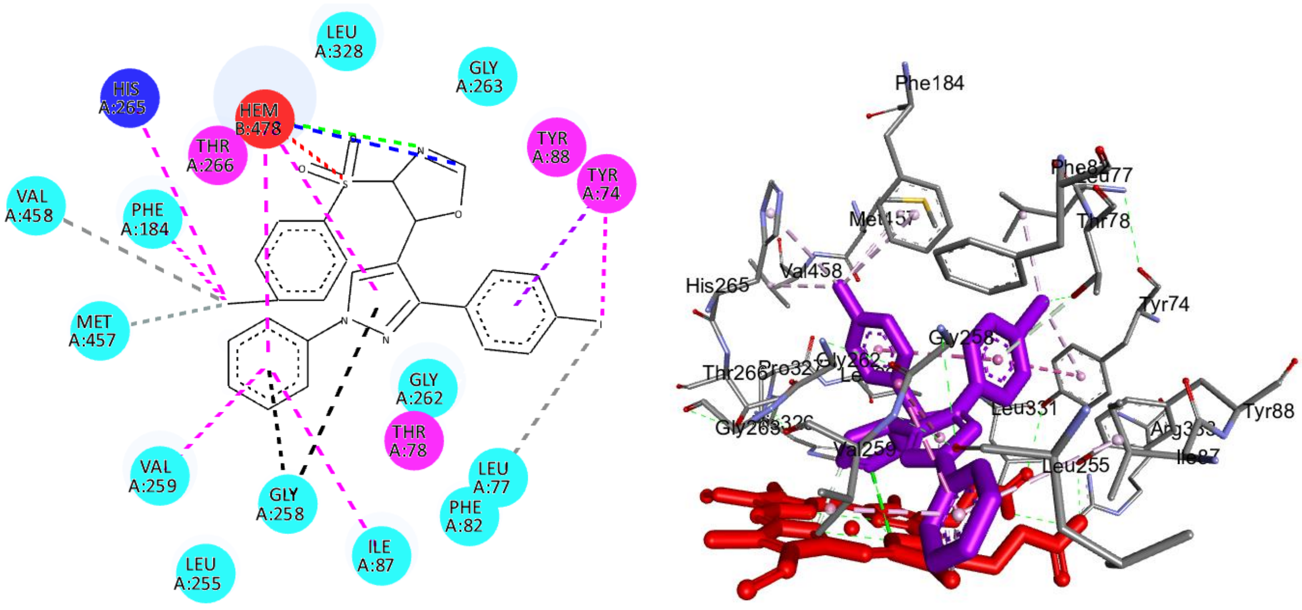
LEU A:328
GLY A:263
HIS A:265
HEM B:478
THR A:266
TYR A:88
TYR A:74
VAL A:458
PHE A:184
MET A:457
GLY A:262
THR A:78
LEU A:77
PHE A:82
VAL A:259
GLY A:258
ILE A:87
LEU A:255

6f

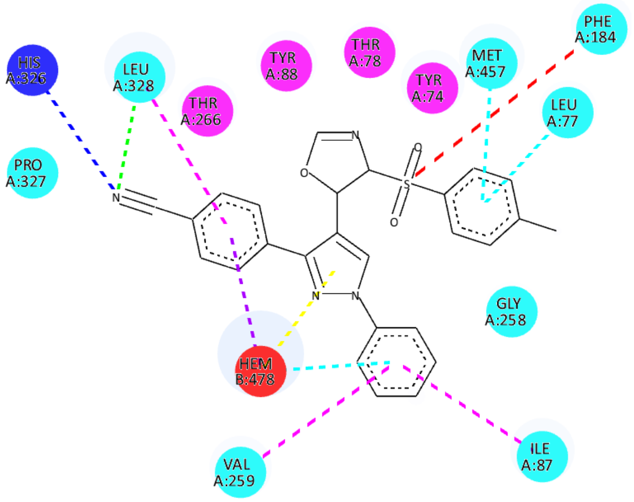
HIS A:326
LEU A:328
TYR A:88
THR A:78
TYR A:74
MET A:457
PHE A:184
LEU A:77
THR A:266
PRO A:327
GLY A:258
HEM B:478
VAL A:259
ILE A:87

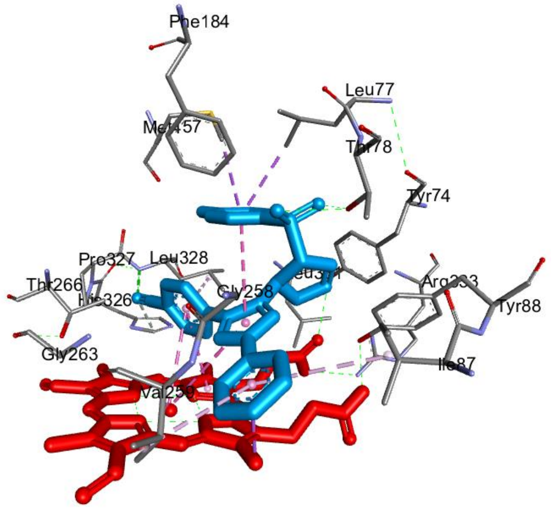
Phe184
Leu77
Tyr74
Pro327
Leu328
Thr266
Gly258
Tyr88
Gly263
Ile87

6g

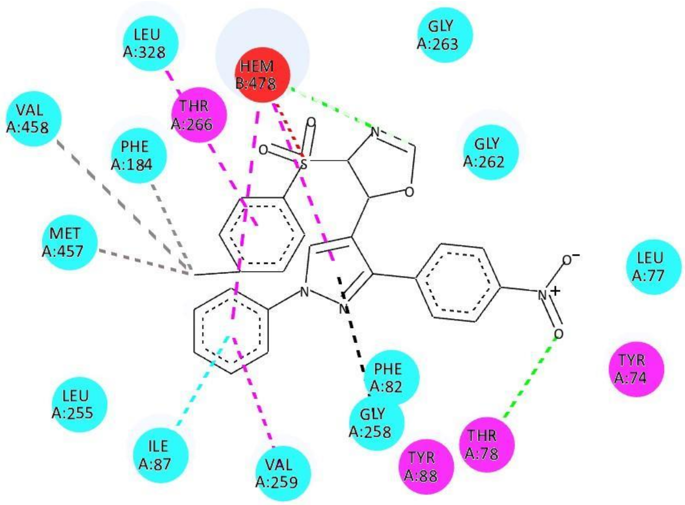
LEU A:328
HEM B:478
GLY A:263
VAL A:458
THR A:266
PHE A:184
GLY A:262
MET A:457
LEU A:77
LEU A:255
PHE A:82
GLY A:258
TYR A:74
ILE A:87
VAL A:259
TYR A:88
THR A:78

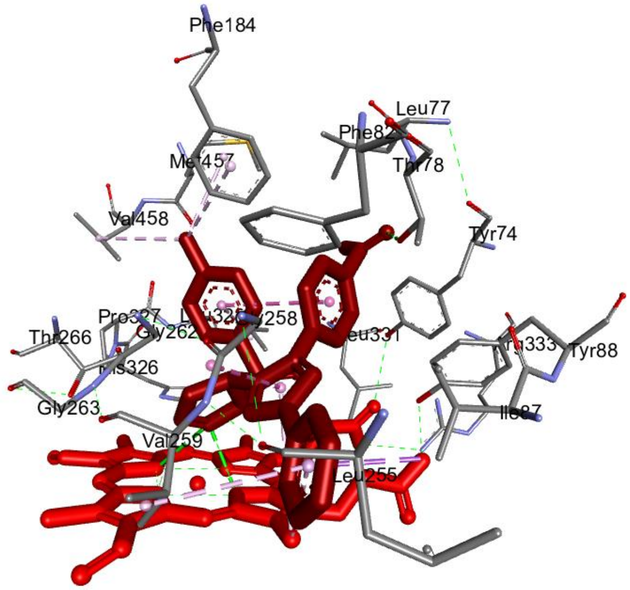
Phe184
Leu77
Phe82
Val458
Tyr74
Thr266
Pro327
Tyr88
Gly263
Val259
Leu255

6h

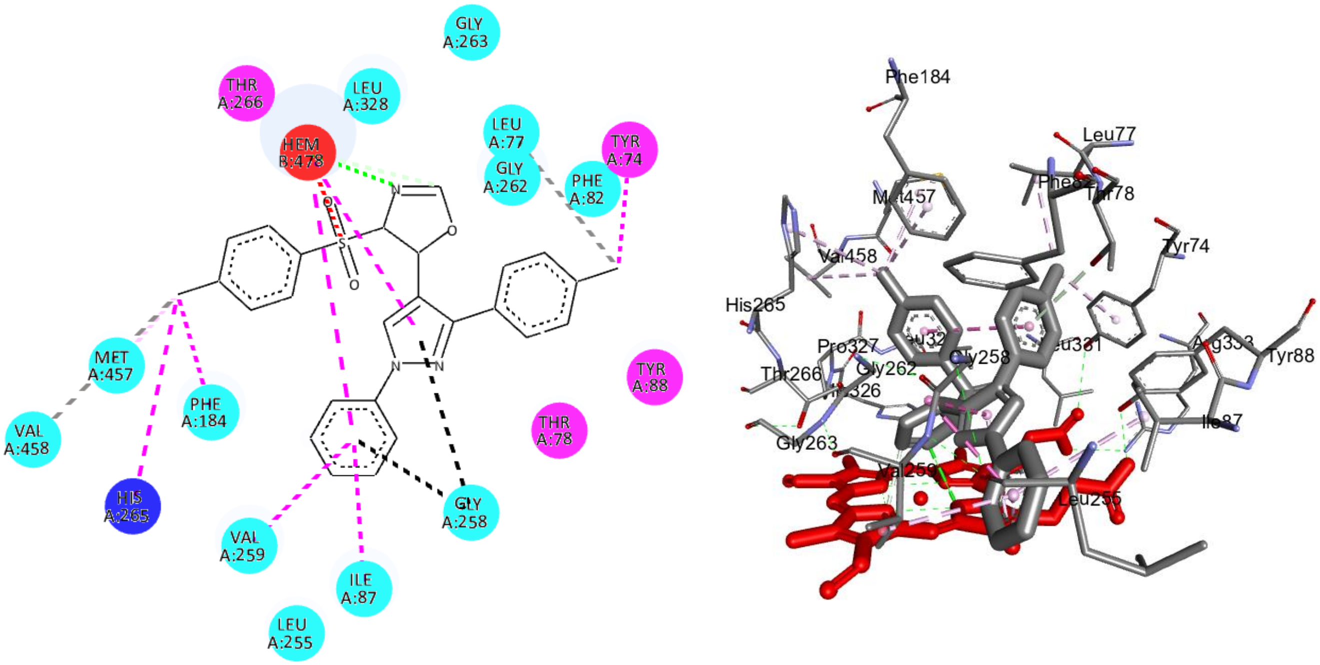
GLY A:263
THR A:266
LEU A:328
HEM B:478
LEU A:77
GLY A:262
PHE A:82
TYR A:74
MET A:457
VAL A:458
PHE A:184
HIS A:265
TYR A:88
THR A:78
VAL A:259
GLY A:258
ILE A:87
LEU A:255
Phe184
Leu77
Met457
Thr78
Tyr74
Val458
His265
Pro327
Gly262
Gly258
Thr266
Arg333
Tyr88
Gly263
Ile87
Leu255

6i

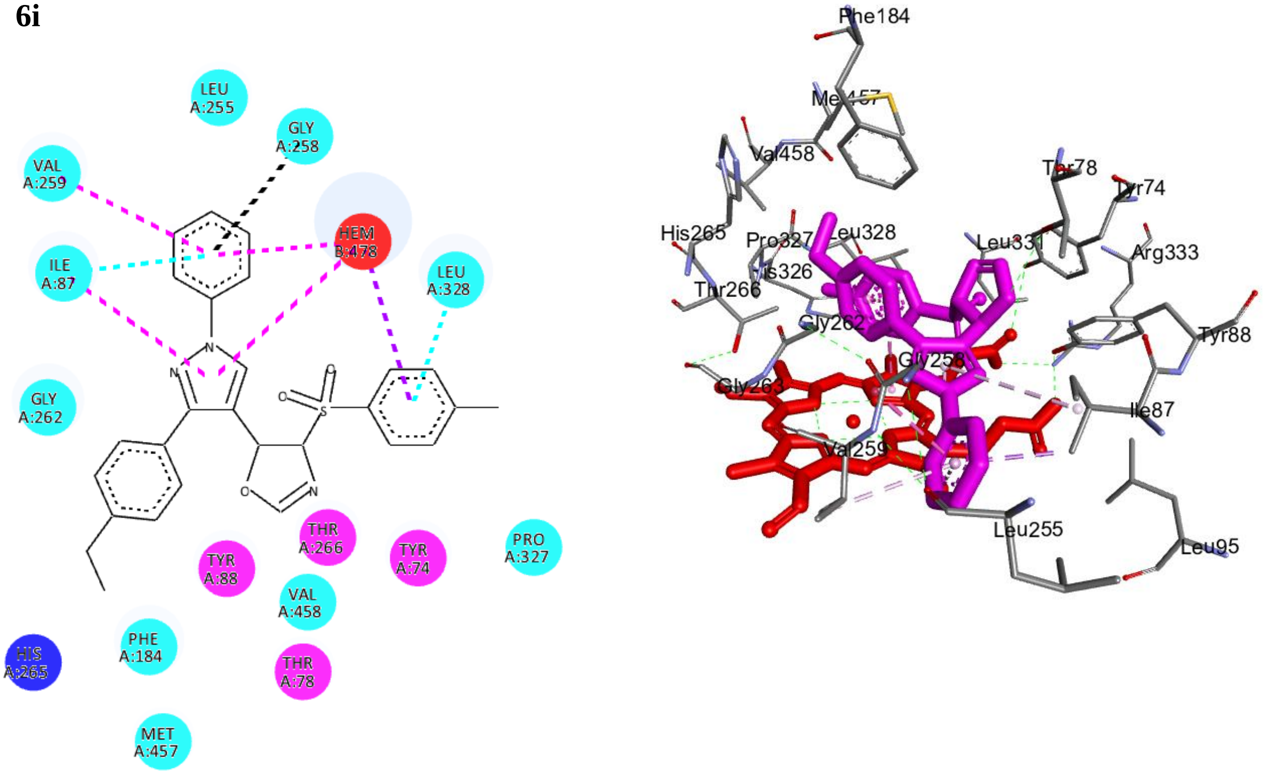
LEU A:255
GLY A:258
VAL A:259
HEM B:478
ILE A:87
LEU A:328
GLY A:262
THR A:266
TYR A:88
TYR A:74
PRO A:327
VAL A:458
HIS A:265
PHE A:184
THR A:78
MET A:457
Phe184
Val458
Thr78
Tyr74
His265
Pro327
Leu328
Leu331
Arg333
His326
Thr266
Gly262
Tyr88
Gly258
Ile87
Val259
Leu255
Leu95

6j

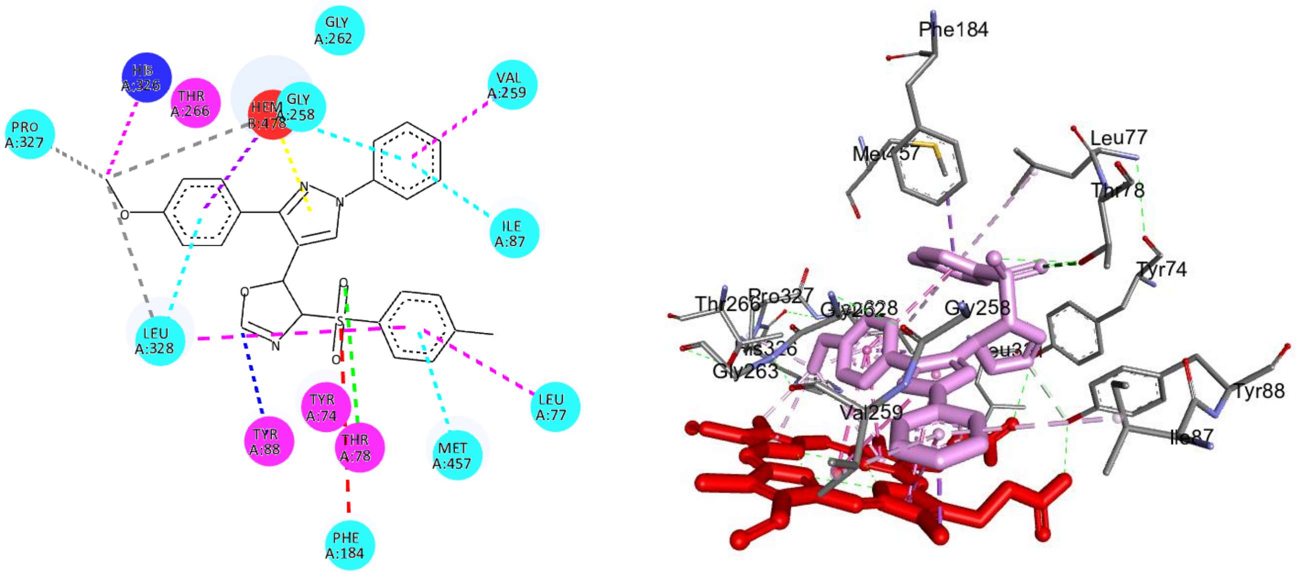

Figure S250. Schematic representation of binding modes of (4*S**, 5*S**)-5-(1,3-diphenyl-1*H*-pyrazol-4-yl)-4-tosyl-4,5-dihydrooxazoles **6a-j**, at the active site of CYP51Cg (*C. glabrata*). The 3D model depicts the hydrophilic bonds and amino acid residues that are part of the active site of CYP51Cg. In the 2D model, the interactions denoted with dotted lines are conventional hydrogen bonds (green), carbon-hydrogen (dark blue), π-cation (orange), π-sigma (cyan), π-alkyl (fuchsia), π-π T-shaped (purple), π-π stacked (yellow), alkyl (gray), π-sulfur (red) and halogen (brown). The heme group is in red, fluconazoles **20** (green), **6a** (blue), **6b** (cyan), **6c** (yellow), **6d** (orange), **6e** (purple), **6f** (turquoise), **6g** (red), **6h** (gray), **6i** (fuchsia) and **6j** (pink). The heme group is in red. The solvent accessible surface is illustrated for the amino acid residues and ligands. The amino acids are highlighted in blue (basic), cyan (hydrophobic), and fuchsia (hydrophilic).

Table S3. ChemScore fitness DG of 1,3-diaryl-1*H*-pyrazole-4-carbaldehydes **4a-j**, 5-(1,3-diphenyl-1*H*-pyrazol-4-yl)-4-tosyl-4,5-dihydrooxazoles **6a-j**, and fluconazole **20** at the active site of lanosterol 14-alpha demethylase CYP51 from *C. albicans* determined in GOLD.

Compound	ChemScore fitness ΔG (kcal/mol)						
	R	*C. albicans*	*C. auris*	*C. dubliniensis*	*C. glabrata*	*C. haemulonii*	*C. krusei*
**Fluconazole**	-	-20.43	-20.43	-17.05	-15.71	-20.27	-20.27
**4a**	H	-43.61	-42.71	-39.98	-44.08	-42.71	-42.71
**4b**	F	-41.25	-41.65	-39.81	-44.36	-50.36	-41.65
**4c**	Cl	-44.91	-53.80	-41.81	-46.05	-50.48	-44.91
**4d**	Br	-48.86	-47.02	-41.69	-46.68	-56.68	-47.02
**4e**	I	-49.27	-48.16	-41.96	-47.14	-58.13	-48.99
**4f**	CN	-43.07	-53.07	-39.86	-43.53	-47.82	-45.06
**4g**	NO_2	-41.87	-46.04	-38.79	-30.01	-38.46	-38.80
**4h**	Me	-46.25	-46.48	-41.41	-45.90	-52.99	-46.48
**4i**	Et	-40.76	-44.90	-40.88	-42.26	-52.41	-44.90
**4j**	OMe	-40.48	-42.28	-38.80	-37.29	-42.28	-42.28
**6a**	H	--51.90	-52.12	-51.85	-40.16	-55.41	-53.19
**6b**	F	-52.98	-52.38	-52.98	-50.36	-54.02	-51.98
**6c**	Cl	-59.05	-54.72	-54.04	-50.48	-55.81	-54.15
**6d**	Br	-60.51	-58.28	-54.53	-56.68	-57.11	-54.26
**6e**	I	-54.45	-59.02	-53.56	-58.13	-54.30	-54.30
**6f**	CN	-51.20	-45.06	-51.20	-47.82	-51.98	-50.61
**6g**	NO_2	-50.40	-48.91	-49.39	-38.80	-52.78	-53.66
**6h**	Me	-54.26	-54.43	-54.02	-52.99	-54.48	-53.05
**6i**	Et	-55.83	-58.04	-53.08	-52.41	-55.58	-55.58
**6j**	OMe	-54.34	-52.00	-49.36	-36.92	-52.74	-49.47

Table S4. RMSD determination on the CYP51 enzyme of *Candida* spp.

CYP51 yeast	RMSD (Å)
C. auris	0.168
C. dubliniensis	0.171
C. glabrata	0.180
C. haemulonii	0.170
C. krusei	0.176

Table S5. Distances of hydrogen bond interactions observed between **fluconazole 20** and 1,3-diaryl-1*H*-pyrazole-4-carbaldehydes **4a-j** and (4*S**, 5*S**)-5-(1,3-diphenyl-1*H*-pyrazol-4-yl)-4-tosyl-4,5-dihydrooxazoles **6a-j** at the active site of CYP51 enzyme of *C. albicans***.**

Compound	Interaction	Distance (Å)
Fluconazole	O-H·····O (Tyr132)	3.71
	C-H·····O (Gly307)	3.45
	N·····H-C (Gly307)	3.52
**4j**	C-H·····O (Gln142)	3.05
**6a**	O·····H-O (Tyr118)	3.08
	O·····H-O (Tyr132)	2.73
**6b**	O·····H-O (Tyr118)	3.74
	O·····H-O (Tyr132)	3.79
	N·····H-N (Met508)	3.65
**6e**	N·····H-O (Tyr118)	2.45
	C-H·····O (Ser378)	3.13
	N·····H-N (Met508)	2.85
**6g**	C-H·····O (Met508)	3.22
**6h**	C-H·····O (Met508)	3.43
**6i**	C-H·····O (Met508)	3.04
**6j**	C-H·····O (Met508)	3.14

Table S6. Distances of hydrogen bond interactions observed between **fluconazole 20** and 1,3-diaryl-1*H*-pyrazole-4-carbaldehydes **4a-j** and (4*S**, 5*S**)-5-(1,3-diphenyl-1*H*-pyrazol-4-yl)-4-tosyl-4,5-dihydrooxazoles **6a-j** at the active site of CYP51 enzyme of *C. glabrata***.**

Compound	Interaction	Distance (Å)
Fluconazole	O-H·····O (Gly258)	3.70
	C-H·····O (Val259)	3.59
	N·····H-C (Gly262)	3.33
**4g**	O·····H-O (Leu255)	4.01
**4j**	C-H·····O (Gln142)	3.30
**6a**	N·····H-N (Hem478)	2.51
**6b**	N·····H-N (Hem478)	2.45
**6c**	O·····H-C (Gly262)	3.37
	C-H·····N (Hem478)	2.79
**6d**	O·····H-C (Gly262)	3.24
	O·····H-C (Hem478)	3.00
**6e**	N·····H-O (Tyr78)	3.71
	N·····H-N (Hem478)	2.26
**6f**	N·····H-C (His326)	3.71
	N·····H-N (Leu328)	2.86

**6g**	O····H-O (Thr78)	4.42
	N····H-N (Hem478)	2.26
**6h**	N····H-N (Hem478)	2.78
**6j**	O····H-O (Thr78)	3.09
	C-H····O (Tyr88)	2.81
